# Characterization of *CRN-Like* Genes From *Plasmopara viticola*: Searching for the Most Virulent Ones

**DOI:** 10.3389/fmicb.2021.632047

**Published:** 2021-03-22

**Authors:** Gaoqing Xiang, Xiao Yin, Weili Niu, Tingting Chen, Ruiqi Liu, Boxing Shang, Qingqing Fu, Guotian Liu, Hui Ma, Yan Xu

**Affiliations:** ^1^State Key Laboratory of Crop Stress Biology in Arid Areas, College of Horticulture, Northwest A&F University, Yangling, China; ^2^Key Laboratory of Horticultural Plant Biology and Germplasm Innovation in Northwest China, Ministry of Agriculture, College of Horticulture, Northwest A&F University, Yangling, China; ^3^College of Horticulture, Northwest A&F University, Yangling, China

**Keywords:** Plasmopara viticola, Vitis, CRN effectors, cell death, virulence

## Abstract

Grapevine downy mildew is an insurmountable disease that endangers grapevine production and the wine industry worldwide. The causal agent of the disease is the obligate biotrophic oomycete *Plasmopara viticola*, for which the pathogenic mechanism remains largely unknown. Crinkling and necrosis proteins (CRN) are an ancient class of effectors utilized by pathogens, including oomycetes, that interfere with host plant defense reactions. In this study, 27 *CRN-like* genes were cloned from the *P. viticola* isolate YL genome, hereafter referred to as *PvCRN* genes, and characterized *in silico* and *in planta. PvCRN* genes in ‘YL’ share high sequence identities with their ortholog genes in the other three previously sequenced *P. viticola* isolates. Sequence divergence among the genes in the *PvCRN* family indicates that different *PvCRN* genes have different roles. Phylogenetic analysis of the PvCRN and the CRN proteins encoded by genes in the *P. halstedii* genome suggests that various functions might have been acquired by the *CRN* superfamily through independent evolution of *Plasmopara* species. When transiently expressed in plant cells, the PvCRN protein family shows multiple subcellular localizations. None of the cloned PvCRN proteins induced hypersensitive response (HR)-like cell death on the downy mildew-resistant grapevine *Vitis riparia*. This was in accordance with the result that most PvCRN proteins, except PvCRN11, failed to induce necrosis in *Nicotiana benthamiana*. Pattern-triggered immunity (PTI) induced by INF1 was hampered by several PvCRN proteins. In addition, 15 PvCRN proteins prevented Bax-induced plant programmed cell death. Among the cell death-suppressing members, PvCRN17, PvCRN20, and PvCRN23 were found to promote the susceptibility of *N. benthamiana* to *Phytophthora capsici*, which is a semi-biotrophic oomycete. Moreover, the nucleus-targeting member, PvCRN19, promoted the susceptibility of *N. benthamiana* to *P. capsici*. Therefore, these PvCRN proteins were estimated to be virulent effectors involved in the pathogenicity of *P. viticola* YL. Collectively, this study provides comprehensive insight into the CRN effector repertoire of *P. viticola* YL, which will help further elucidate the molecular mechanisms of the pathogenesis of grapevine downy mildew.

## Introduction

Grapevine (*Vitis* spp.) is one of the most widely distributed and economically important fruit crops globally. In humid weather, grapevine downy mildew occurs in vineyards, causing severe losses in the yield and quality of grapes and economic losses to the grape and wine industries (Gessler et al., 2011). The causal agent of grapevine downy mildew is the obligate biotrophic pathogen *Plasmopara viticola* (Berk. & M. A. Curtis) Berl. & De Toni, which belongs to the Oomycota. Chemical fungicides are currently used to control grapevine downy mildew but have high costs and negative environmental impacts. An alternative to the use of chemical fungicides is the incorporation of genes that could confer resistance to *P. viticola* into grapevine varieties, which is an environmentally friendly and cost-efficient approach. Discovery of novel genes related to pathogen resistance should be based on the elucidation of the mechanisms underlying the interactions between host plants and pathogens (Mestre et al., 2012).

It is well known that plants and pathogens co-evolve following the “*Zig-zag*” model. In this model, to impede the PTI (pattern-triggered immunity) responses of the host, the pathogen secretes apoplastic effectors and cytoplasmic effectors to the interface of the “battlefield” to improve its pathogenicity. However, the plant activates defense responses called ETI (effector-triggered immunity) with resistance (R) genes, which recognize a certain effector, to suppress the infection progress of the pathogen. Notably, there would be more pathogen effectors and plant R genes contributed to the interaction as both the host and the pathogen are evolving under natural selection. The arms race between host plants and pathogens is continuous and never ending (Jones and Dangl, 2006).

With the discovery of resistance-related genes in some plant species and effector genes in some pathogens, a better understanding of the molecular mechanisms underlying the interactions between plants and pathogens has been achieved (Wanderley-Nogueira et al., 2017; Qin et al., 2020). Among the oomycetes, the genome sequences of many species from the hemibiotrophic *Phytophthora* genus and the biotrophic *Plasmopara* genus are available, including those of *Phytophthora ramorum*, *Phytophthora sojae* (Tyler et al., 2006), *Phytophthora infestans* (Haas et al., 2009), *Phytophthora capsici* (Lamour et al., 2012a), *Phytophthora lateralis* (Quinn et al., 2013), *Plasmopara viticola* (Dussert et al., 2016, 2019; Yin L. et al., 2017; Brilli et al., 2018), and *Plasmopara halstedii* (Sharma et al., 2015). Analysis of these genome sequences has shown that these pathogens possess genes encoding proteins known as effectors, including the cytoplasmic effector RXLR and CRN families. The RXLR effectors have the conserved amino acid sequence R(arginine)-X (any amino acid)-L (leucine)-R (arginine), whereas CRN effectors are characterized by a conserved amino (N)-terminal motif LXLFLAK (Yin L. et al., 2017). Another conserved motif, HVLVXXP, is also shared among CRN proteins and is followed by variegated carboxyl (C)-terminal sequences. It has been well documented that RXLR effectors serve as avirulent proteins when recognized by host plant R proteins, whereas they act as virulent effectors when host defense responses are successfully hampered by them (Oh et al., 2009; Bos et al., 2010). However, the function of CRN proteins is unresolved, even though some CRN proteins cause a crinkling and necrosis phenotype when transiently expressed *in planta* (Torto et al., 2003). Studies have indicated that CRN proteins may have additional functions besides cell death-inducing activity (Amaro et al., 2017). For example, *Phytophthora sojae* effectors PsCRN63 and PsCRN115 synergistically interact with host plant catalases (CAT1) to suppress H_2_O_2_ accumulation and promote pathogenicity (Zhang et al., 2015). Another virulence effector, PsCRN108, contains a helix-hairpin-helix (HhH) motif and inhibits transcription of plant HSP genes by interacting with the HSE elements in the promoters of HSP genes (Song et al., 2015). The characteristics and functions of CRN effectors from *Plasmopara viticola* remain undefined despite the identification of *CRN* genes in the genomes of several *P. viticola* strains. In the present study, a group of candidate *CRN* genes from another *P. viticola* strain, *P. viticola* isolate YL (Yin X. et al., 2017), were identified and transiently expressed *in planta* to analyze their properties. The subcellular localization of the PvCRN proteins in plant cells and their transcription patterns during *P. viticola* infection were determined. Moreover, the virulence of several PvCRN proteins was revealed. This study provides basic information on the *CRN* genes in *P. viticola* YL, which will help identify the vital effectors involved in the interaction between *P. viticola* and *Vitis.* spp.

## Materials and Methods

### Microbial Strains, Plants, and Culture Conditions

*Escherichia coli* Top10 used for DNA cloning was selectively cultured in solid or liquid Luria-Bertani (LB) media containing kanamycin (50 mg/L) at 37°C. *Agrobacterium tumefaciens* GV3101 was cultured at 28°C in solid or liquid LB media containing kanamycin (50 mg/L), gentamycin (30 mg/L), and rifampicin (50 mg/L). Transformations of DNA into Top10 and GV3101 were conducted following standard protocols for heat shock treatment and freezing-thawing transformation, respectively.

*Plasmopara viticola* YL was preserved in the lab by inoculating a sporangium suspension of the strain onto young leaves of *V. vinifera* Thompson Seedless every 5–7 days. *Phytophthora capsici* was maintained by subculturing the mycelia on V8 juice medium (2.5% V8, 1.5% agar) at 25°C in the dark.

*In vitro* grown plantlets of grapevine *V. riparia* and *V. vinifera* Thompson Seedless, which were intended for use in the agrobacterium-mediated gene transient expression experiments, were primarily cultured in jars with medium containing half-strength Murashige and Skoog medium (MS), 30 g/L sucrose, 0.1 mg/L IBA (indol-3-butyric acid), 0.3 mg/L 6-BA (6-benzylaminopurine), and 3 g/L phytagel. The *in vitro* plants were propagated in the medium with half-strength MS, 15 g/L sucrose, 0.15 mg/L IBA, and 3 g/L phytagel. The environmental temperature of the tissue culture system was 24 ± 2°C, and the photoperiod was set to 16 h illumination: 8 h dark. Plants of *V. vinifera* Pinot Noir were grown in the Grape Germplasm Resources Repository at Northwest A&F University, Yangling, China. Detached grapevine leaves inoculated with *P. viticola* YL were incubated in a climate chamber with same culture conditions as the *in vitro* plants. *Nicotiana benthamiana* seedlings were grown in pots with a matrix composed of peat and vermiculite at 22 ± 2°C under a 16 h photoperiod and 75% relative humidity in a greenhouse.

### Bioinformatic Analysis

The protein sequences encoded by the *CRN* genes cloned from the *P. viticola* YL genome were translated using the ExPASy-Translate tool^1^. Then, protein physical and chemical parameters were predicted using ExPASy-ProtParam^2^. Signal peptide prediction of PvCRN proteins was performed using the SignalP 3.0 server^3^ and Phobius^4^. For prediction with SignalP 3.0, both neural networks (NN) and hidden Markov models (HMM) methods were used. The prediction criteria were as follows: the sequence was labeled “yes” when the *S*-score was greater than the default threshold 0.47 under SignalP-NN prediction, and for SignalP-HMM prediction, the sequence was labeled “yes” when the S-probability was greater than the default threshold 0.5 (Sperschneider et al., 2015). The presence of signal peptides was finally determined by combining the prediction results of the two software programs. The presence of importin α-dependent nuclear localization signal (NLS) peptides was predicted by the NLS Mapper server^5^. Transmembrane helices were predicted using Predictprotein^6^ and TMpred server^7^. Multiple sequence alignment of PvCRN proteins was conducted in ClustalX-2.1, and the results were converted into colored images using CLC Sequence Viewer 8.0. The most conserved N-terminal motifs among the PvCRN proteins were identified, and the corresponding sequence logos were generated by Weblogo^8^. The presence of the effector subdomains defined in *Phytophthora* species (Haas et al., 2009; Stam et al., 2013) in the C-terminal region of PvCRN proteins were identified by multiple sequence alignment with CLC Sequence Viewer 8.0. Phylogenetic analysis was performed using MEGA7.0, using the maximum likelihood method with the best-fit substitution model WAG + G (Zuckerkandl and Pauling, 1965; Kumar et al., 2016). Phylogenetic trees for display were drawn with the iTOL server^9^. Conserved functional domains and motifs of PvCRN proteins were predicted using the online software Pfam 33.1^10^, SMART^11^, and ExPASy-ScanProsite^12^.

### DNA and RNA Preparation

A sporangium suspension of 50,000 sporangia/mL of *P. viticola* YL was sprayed on detached leaves of *V. vinifera* Pinot Noir, which had been sterilized with 1% sodium hypochlorite, for propagation (Mestre et al., 2012). After the sporangiophores of *P. viticola* YL reached out from the abaxial surface of grapevine leaves, the sporangia were collected into sterilized water using a soft brush, centrifuged, and stored at –80°C. Genomic DNA was extracted from *P. viticola* YL using the CTAB method (Xin and Chen, 2012). For gene expression analysis, *P. viticola* YL sporangia were inoculated onto detached Pinot Noir leaves in the same manner described above. Different batches of inoculated grapevine leaves were collected at 0, 24, 48, 72, 96, and 120 h post inoculation. The whole experiment was repeated three times. All samples collected were stored at –80°C before use. The hybrid total RNA of grapevine leaves and *P. viticola* YL mycelia was collected at different time points and isolated using the E.Z.N.A.^®^ Plant RNA Kit (Omega Bio-tek, United States).

### DNA Cloning, Vector Construction, and Reverse Transcription-PCR

The coding sequences of the *PvCRN* genes, excluding the fragments that were predicted with high probability to be related to signal peptides, were amplified from the genomic DNA of *P. viticola* YL using a KOD-Plus-Neo DNA Polymerase Kit (TOYOBO, Japan) with specific primer pairs (Supplementary Table 1). The DNA sequences predicted to encode signal peptides were cloned using the same method. *PvCRN* genes without signal peptide coding sequences were inserted into the plant virus-based expression binary vector PVX1 that was modified from the pGR106/107 vector with two restriction enzyme sites (*Sma*I and *Not I*). After confirmation by sequencing, the corresponding DNA was subcloned into the plant expression vector pCAMBIA2300-GFP (Su et al., 2018). The signal peptide coding sequences were introduced to the signal peptide trap vector pSUC2 (pSUC2T7M13ORI) and confirmed by sequencing. The first-strand cDNA was synthesized using a PrimeScript^TM^ II 1st Strand cDNA Synthesis Kit (Takara, Japan) with mixed total RNA (2 μg) as the template. Then, the cDNA was diluted with an equal volume of nuclease-free water. Expression of each *PvCRN* gene at different time points during the development of downy mildew was detected by performing reverse transcription-PCR (RT-PCR) on the diluted cDNA templates (Liu et al., 2018). The PCR procedure was as follows: a pre-denaturing step at 94°C for 3 min, a 33-cycle reaction consisting of denaturation at 94°C for 30 s, annealing at 54°C for 30 s, and extension at 72°C for 2 min, followed by a final elongation at 72°C for 8 min. The PCR products were separated by 1% agarose gel electrophoresis and detected on a gel imaging system.

### Transient Expression of *PvCRN* Genes *in planta*

The plasmid constructs harboring PVX1-*PvCRN* and pCAMBIA2300-*PvCRN-GFP* were transformed into *Agrobacterium tumefaciens* strain GV3101 using the freezing-thawing method. After confirmation by selective culture and PCR testing, the transformants were propagated in a shaking culture in LB liquid media with appropriate antibiotics at 28°C. The bacterial cells were collected by centrifugation (5000 rpm × 3 min), washed twice with 10 mM MgCl_2_ solution, and resuspended in the infiltration buffer (10 mM MES, pH 5.7, 10 mM MgCl_2_, and 200 μM acetosyringone), followed by incubation at 28°C for 3 h before infiltration. The bacterial suspension was diluted to an OD_600_ of 0.4 with the infiltration buffer for each plasmid construct and then injected into *N. benthamiana* leaves from the abaxial surface with a 1 mL syringe without a needle. For cell death suppression assays, the left and right sides of the *N. benthamiana* leaves were agroinfiltrated by *A. tumefaciens* containing either *GFP* or *PvCRN* genes, respectively, and the infiltrated areas were marked. Twelve h after the first infiltration, the marked areas of the leaves were re-infiltrated by *A. tumefaciens* carrying either the PVX-*INF1* or PVX-*Bax* constructs. For subcellular localization analysis of the PvCRN proteins, the OD_600_ of the agrobacteria transformed with the pCAMBIA2300-*PvCRN*-*GFP* construct was adjusted to 0.3. For measurements of the transient expression of each *PvCRN* gene in *Vitis* spp., *A. tumefaciens* cells were resuspended in grapevine infiltration buffer (10 mM MES, pH 5.7, 10 mM MgCl_2_, and 500 μM acetosyringone), and the OD_600_ for each plasmid construct was adjusted to 1.0. The bacteria were injected into the top right and bottom left parts of the abaxial surfaces of the *in vitro* grapevine leaves with a needleless 1 mL syringe (Zottini et al., 2008). *A. tumefaciens* cells containing pCAMBIA2300-*GFP* or pCAMBIA2300-*RXLR77*-*GFP* were injected into the lower right and upper left parts of the abaxial surface, respectively, of the same leaves expressing each *PvCRN* to serve as controls. The infiltrated grapevine leaves were monitored for the expression of genes of interest for 10 days post infiltration, and the phenotypes of the leaves expressing genes of interest were recorded.

### Fluorescence Imaging

The subcellular localization of the PvCRN proteins in plant cell were determined by observing the fluorescence of transiently expressed PvCRN-GFP proteins in *N. benthamiana* leaves 48–72 h after agroinfiltration with confocal microscopy, following the standard protocol. The nuclear localization of the proteins was marked by co-expression of the construct pYJ-*NLS-mCherry* with PvCRN-GFP proteins. The plasma membrane-localized marker PM-RK (Zhu et al., 2014) was co-expressed with each PvCRN protein to validate the plasma membrane localization of proteins. For each transiently expressed PvCRN-GFP protein that had been introduced to *in vitro* grapevine leaves by means of agroinfiltration, expression was validated by observing the GFP fluorescence of the recombinant protein using fluorescence microscopy, as described in a previous study (Zottini et al., 2008).

### Yeast Signal Sequence Trap Assay

The secretion function of the putative signal peptide sequences in the PvCRN proteins was verified using a previously described yeast signal sequence trap assay (Oh et al., 2009). Briefly, plasmid constructs of the putative signal peptide coding sequence (pSUC2-*PvCRNXSP*) were transformed into the invertase-negative yeast strain YTK12 using the lithium acetate method, and the transformants were obtained by selective culture on CMD-W medium (0.67% yeast nitrogen base without amino acids, 0.075% minus Trp dropout Supplement, 2% sucrose, 0.1% glucose, and 2% agar). The positive clones were then transferred to YPRAA medium (1% yeast extract, 2% peptone, 2% raffinose, and 2% agar) to verify invertase secretion. The invertase activity was determined by monitoring the reduction of 2,3,5-triphenyl tetrazolium chloride (TTC) to the insoluble, red-colored triphenylformazan. Signal peptide sequences of the effectors Avr1b and Mg87 were included in the experiment as positive and negative controls, respectively.

### Inoculation of *Phytophthora capsici* on *N. benthamiana*

Fresh *P. capsici* mycelia were cut into small pieces and cultured in liquid V8 medium for approximately 4 days to produce sporangia. Then, the mycelia containing sporangia were washed twice with cold sterilized water (4°C) and incubated at 4°C in water to induce zoospore release. The concentration of zoospores was measured with a hemocytometer, and the final zoospore concentration used in inoculations was adjusted to 50 zoospores/μL. *N. benthamiana* leaves that had been infiltrated with agrobacteria containing the indicated plasmid constructs were detached at 48 h post infiltration (hpi), inoculated with 10 μL droplets of *P. capsici* zoospores on their abaxial surfaces, and then incubated in plastic trays with sufficient moisture at 25°C for 48 h. The symptoms and lesion lengths for all lesions were recorded for 9–12 infected leaves, and the experiment was repeated at least three times (Song et al., 2015). Statistical analysis was conducted in Excel and GraphPad Prism 6.

### Immunoblotting

*Nicotiana benthamiana* leaves infiltrated with agrobacteria carrying the genes of interest were collected at 48–72 hpi, immediately frozen in liquid nitrogen, and stored at –80°C before western blot analysis. After being ground to a powder, the samples were transferred into microtubes with protein extraction buffer (50 mM Tris-HCl [pH 7.4], 150 mM NaCl, 10% glycerol, 0.2% Triton X-100, 0.1% NP-40, 0.25% sodium deoxycholate, 2 mM ethylenediaminetetraacetic acid [pH 8.0], 5 mM dithiothreitol, and 2 mM phenylmethylsulphonyl fluoride) and gently vortexed intermittently for 40 min. The samples were then centrifuged at room temperature (12000 rpm × 10 min) to collect the supernatants. The supernatants were boiled with 5 × SDS-loading buffer and loaded into 12% SDS-PAGE gels for separation. Gels were blotted onto a PVDF membrane (Merck, Germany) with a semi-dry gel transfer instrument (Bio-Rad, United States), followed by blocking the membrane in 5% skimmed milk dissolved in TBST (20 mM Tris-HCl [pH 8.0], 150 mM NaCl, and 0.05% (v/v) Tween 20). Membranes were incubated with the primary antibody mouse anti HA-Tag mAb or anti-GFP mAb (ABclonal) at a ratio of 1: 3000 at 4°C overnight. After washing three times with TBST, the membrane was incubated with the corresponding horseradish peroxidase (HRP)-conjugated goat anti-mouse IgG (H + L) secondary antibody (ABclonal) at a ratio of 1:5000 at room temperature for 2 h. Finally, the target proteins were detected by enhanced chemiluminescence (Beyotime, China) after the membrane was washed four times with TBST.

## Results

### Conserved Modules Coupled With Differentiation Events Were Found in the PvCRN Family

In *P. viticola* isolate YL, 35 functional *CRN*-*like* genes were predicted from its genome sequence data^13^, and 27 out of 35 *CRN-like* gene coding sequences (*PvCRN*) were successfully cloned and verified. The NCBI accession number and the DNA sequence associated with each *PvCRN* gene are provided in Supplementary Table 2. All cloned *PvCRN* sequences were aligned with the genome sequences of other *P. viticola* isolates, including those of JL-7-2, INRA-PV221, and PVitFEM01 (Dussert et al., 2016; Yin X. et al., 2017; Brilli et al., 2018) using the NCBI-BLAST program. All the *PvCRN* genes in YL shared high similarities with the *CRN-like* DNA sequences of the other three *P. viticola* isolates (data not shown), but a few *CRN-like* genes showed 100% identity with the cognate DNA sequences from the other three genomes (Table 1). The molecular weights of the PvCRN proteins ranged from 16.51 to 84.22 kDa, with most members having molecular weights below 50 kDa (Table 2). All PvCRN proteins have the conserved motifs L (/V) -X-L-Y (/F)-L-A-K (/R/H) and I-H-V-L-V-X-X-P present at their N-terminal regions (Supplementary Figures 1, 2). In addition, two conserved motifs, V-X-L-X-C-A-X-V (/Y)-G and W-L (/M) were also found at the N-terminal regions of the PvCRN proteins (Supplementary Figures 1, 2). In contrast, the C-terminal sequences of the PvCRN family proteins were diverse, and no new CRN-related motif was found (Supplementary Figure 2). Only a few C-terminal subdomains reported in *Phytophthora* spp. were matched to a few PvCRN proteins (Table 2).

**TABLE 1:** DNA sequence identity analysis of *PvCRN* genes in four *P. viticola* isolates.

***PvCRN* genes in *P. viticola* YL**		**Presence of orthologs with 100% identity**		
**Gene Name**	**NCBI Accession Number**	**JL-7-2**	**INRA-PV221**	**PVitFEM01**
*PvCRN1*	MW567423	No	No	No
*PvCRN2*	MW567424	No	Yes (1 hit)	Yes (1 hit)
*PvCRN4*	MW567426	No	No	No
*PvCRN6*	MW567428	No	Yes (1 hit)	No
*PvCRN7*	MW567429	No	No	No
*PvCRN9*	MW567431	No	No	No
*PvCRN10*	MW567432	No	No	Yes (1 hit)
*PvCRN11*	MW567433	No	No	Yes (1 hit)
*PvCRN12*	MW567434	No	No	No
*PvCRN14*	MW567436	No	No	No
*PvCRN15*	MW567437	No	Yes (1 hit)	No
*PvCRN16*	MW567438	No	Yes (1 hit)	No
*PvCRN17*	MW567439	No	No	No
*PvCRN18*	MW567440	No	Yes (1 hit)	No
*PvCRN19*	MW567441	No	No	No
*PvCRN20*	MW567442	No	No	Yes (1 hit)
*PvCRN21*	MW567443	No	No	No
*PvCRN22*	MW567444	No	No	Yes (1 hit)
*PvCRN23*	MW567445	No	No	No
*PvCRN24*	MW567446	No	No	No
*PvCRN25*	MW567447	No	Yes	Yes
*PvCRN26*	MW567448	Yes (1 hit)	Yes (1 hit)	No
*PvCRN27*	MW567449	No	No	No
*PvCRN29*	MW567451	Yes (1 hit)	Yes (1 hit)	No
*PvCRN30*	MW567452	Yes (1 hit)	Yes (1 hit)	No
*PvCRN31*	MW567453	No	No	No
*PvCRN35*	MW567457	Yes (1 hit)	No	No

**TABLE 2:** Characteristics and predicted features of PvCRN effectors in *P. viticola* isolate YL.

**PvCRN**	**MW (kDa)**	**Subdomains^*a*^ in C - terminus**	**Signal peptide^*b*^ (region)**	**NLS^*c*^**	**TMH ^*d*^ (region)**	**Conserved domain or motif^*e*^**
PvCRN1	65.20	New DXV	Yes (1–15)	Yes	Yes (186-209)	No
PvCRN2	19.71	No	Yes (1–14)	No	No	No
PvCRN4	65.50	New DXV	No	Yes	Yes (193-214)	No
PvCRN6	41.44	No	No	Yes	Yes (233-252)	No
PvCRN7	84.22	DC	Yes (1–17)	Yes	Yes (280-298;313-342)	No
PvCRN9	69.77	No	Yes (1–17)	Yes	Yes (196-218)	No
PvCRN10	24.47	No	Yes (1–17)	No	Yes (178-203)	No
PvCRN11	24.20	No	No	Yes	Yes (177-199)	No
PvCRN12	19.57	No	No	Yes	No	No
PvCRN14	16.51	No	No	Yes	No	No
PvCRN15	33.17	No	No	Yes	Yes (185-205)	No
PvCRN16	42.69	DBE	No	Yes	Yes (171-190)	HNH_2 (HNH endonuclease)
PvCRN17	21.27	No	No	Yes	Yes (144-163)	Ubiquitin_2
PvCRN18	32.65	No	No	Yes	Yes (262-284)	No
PvCRN19	19.00	No	No	Yes	Yes (10-33)	No
PvCRN20	18.18	No	No	Yes	Yes (21-40)	No
PvCRN21	54.50	No	Yes (1–13)	Yes	Yes (200-223)	No
PvCRN22	23.07	No	Yes (1–17)	Yes	No	No
PvCRN23	47.78	DBE	Yes (1–17)	No	Yes (386-408)	HNH_2
PvCRN24	18.40	No	No	No	Yes (71-92)	No
PvCRN25	21.32	No	No	No	No	No
PvCRN26	32.19	No	No	Yes	Yes (188-204)	No
PvCRN27	34.23	No	No	Yes	Yes (217-243)	No
PvCRN29	34.06	No	No	Yes	Yes (217-243)	No
PvCRN30	25.00	No	No	No	Yes (161-184)	No
PvCRN31	65.70	No	No	Yes	No	Protein kinase domain (PS50011)
PvCRN35	24.70	No	No	Yes	No	No

*^a^“No” means no subdomains reported in *Phytophthora* spp. are found in the C-terminus of the indicated PvCRN protein.*

*^b^“Yes” means a classical signal peptide has been predicted by SignalP 3.0 and Phobius. “No” means no classical signal peptide has been predicted.*

*^c^“Yes” means at least one classical NLS has been predicted by the NLS Mapper server. “No” means no NLS has been predicted.*

*^d^TMH is the abbreviation of transmembrane helix. “Yes” means TMH has been predicted by Predictprotein and TMpred server. “No” means noTMH has been predicted.*

*^e^“No” means no conserved domains or motifs have been found by Pfam 33.1, SMART, and ExPASy-ScanProsite.*

There are eight PvCRN proteins predicted to have classical signal peptide sequences, whereas most PvCRN proteins showed no classical secretion features (Table 2). Most PvCRN proteins (21 out of 27) were predicted to have classical NLS. Meanwhile, most PvCRN proteins (20 out of 27) seem to form transmembrane helix structures. Some PvCRN proteins also contain some conserved functional domains or motifs in their C-termini; for example, PvCRN31 contains the protein kinase domain (Prosite entry PS50011). The HNH_2 motif (PF13391), which is related to DNA binding, was found both in PvCRN16 and PvCRN23 in the C-terminal region (Table 2).

High sequence similarities were found within several groups of *PvCRN* genes, indicating that gene duplication has occurred during the evolution of *PvCRN* genes. *PvCRN18*, *PvCRN27*, and *PvCRN29* probably developed from one ancestral gene (Supplementary Figure 3). In particular, *PvCRN27* and *PvCRN29* could be paralogs produced from gene tandem duplication as the distance between these two loci is less than 100 kb (data not shown). Similarly, *PvCRN10* and *PvCRN11* could also be paralogs developed from gene duplication (Supplementary Figure 4).

In addition, gene recombination was also found among *PvCRN* genes. Three types of gene recombination phenomena were significantly detected by RDP5. First, *PvCRN* genes shared a highly conserved N-terminal coding sequence but differed in their C-terminal coding sequences. Second, *PvCRN* genes shared a highly conserved C-terminal coding sequence but displayed diversity in the N-terminal coding sequence (Table 3). Third, one *PvCRN* gene was a recombinant gene composed of different fragments from at least two other *PvCRN* genes. For example, *PvCRN15* is a recombinant gene of *PvCRN26* and *PvCRN23*, with the former providing most of the 5’ terminal fragment and the entire 3′ terminal coding sequence, and the latter providing the remainder of the *PvCRN15* N-terminal coding sequence. *PvCRN16* is derived from the partial 5′ end sequence and the entire 3′ terminal sequence of *PvCRN23*, with the rest of the 5′ terminal sequence derived from *PvCRN26*. For *PvCRN21*, the major parent was *PvCRN9*, while the minor parent was predicted to be *PvCRN31*(Table 3 and Supplementary Table 3).

**TABLE 3:** Gene recombination events found in the *PvCRN* gene family.

**Gene recombination type**	**Phenomena**
Conserved N-terminus + different C-terminus	*PvCRN31 and PvCRN11 PvCRN12/PvCRN35 and PvCRN17 PvCRN21 and PvCRN22*
Different N + conserved C	*PvCRN1*and *PvCRN4 PvCRN1/PvCRN4* and *PvCRN30*
Gene A is a recombinant of B and C	*PvCRN15* (major parent: *PvCRN26*, minor parent: *PvCRN23*) *PvCRN16* (major parent: *PvCRN23*, minor parent: *PvCRN26*) *PvCRN9* (major parent: *PvCRN21*, minor parent: *PvCRN31*)

**FIGURE 1:**
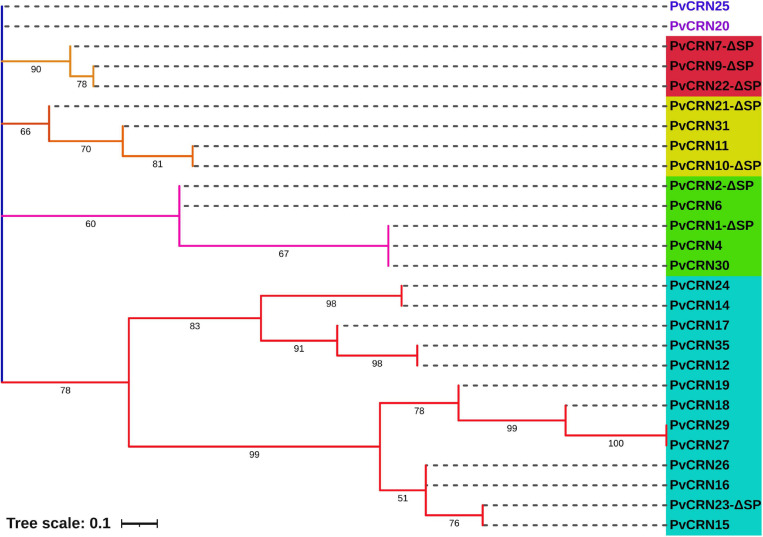
Phylogenetic relationships among the *PvCRN* genes of *P. viticola* isolate YL. The phylogenetic tree was originally generated in MEGA7.0 using the maximum likelihood method with 1,000 bootstrap replicates. The optimal substitution model was WAG + G. Different subgroups are highlighted with different colors, and bootstrap values greater than 50 are indicated on the nodes.

**FIGURE 2:**
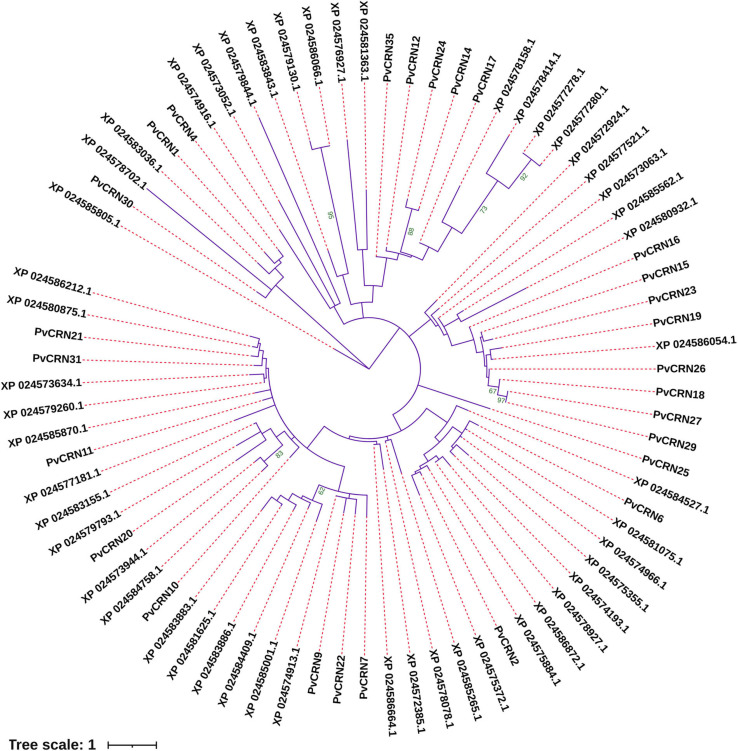
Phylogenetic relationships among the CRN proteins from *P. viticola* YL and *P. halstedii*. The phylogenetic tree was generated in MEGA7.0 using the maximum likelihood method with 1,000 bootstrap replicates. The optimal substitution model was WAG + G. Bootstrap values greater than 50 are indicated in green on the nodes.

The mature protein sequences of all 27 PvCRNs (the predicted classical signal peptide sequences were excluded for the eight PvCRNs mentioned above) were aligned with ClustalX-2.1; the alignment is available in Supplementary Figure 5. The alignment was then submitted to MEGA 7.0 for phylogenetic analysis. A phylogenetic tree (Figure 1) with 50% bootstrap cutoffs showed that PvCRN proteins cloned from *P. viticola* YL were divided into seven clades, including five groups and two singletons, suggesting that *PvCRN* genes are actively evolving new features and functions despite retaining several conserved motifs. Compared to the CRN-like proteins of the sunflower downy mildew causal agent *P. halstedii* (hereafter referred to as PhCRN proteins, and the sequence information is available at https://www.ncbi.nlm.nih.gov/genome/?term=txid4781[Organism:noexp]), no PvCRN proteins clustered together with PhCRN proteins in a phylogenetic tree with 50% bootstrap cutoffs, indicating that the phylogenetic relationships between most *PvCRN* genes and *PhCRN* genes are distant (Figure 2). The complete alignment of CRN proteins from *P. viticola* YL and *P. halstedii* is available in Supplementary Figure 6.

### Functional Validation of Predicted Signal Peptides in PvCRN Proteins

**FIGURE 3:**
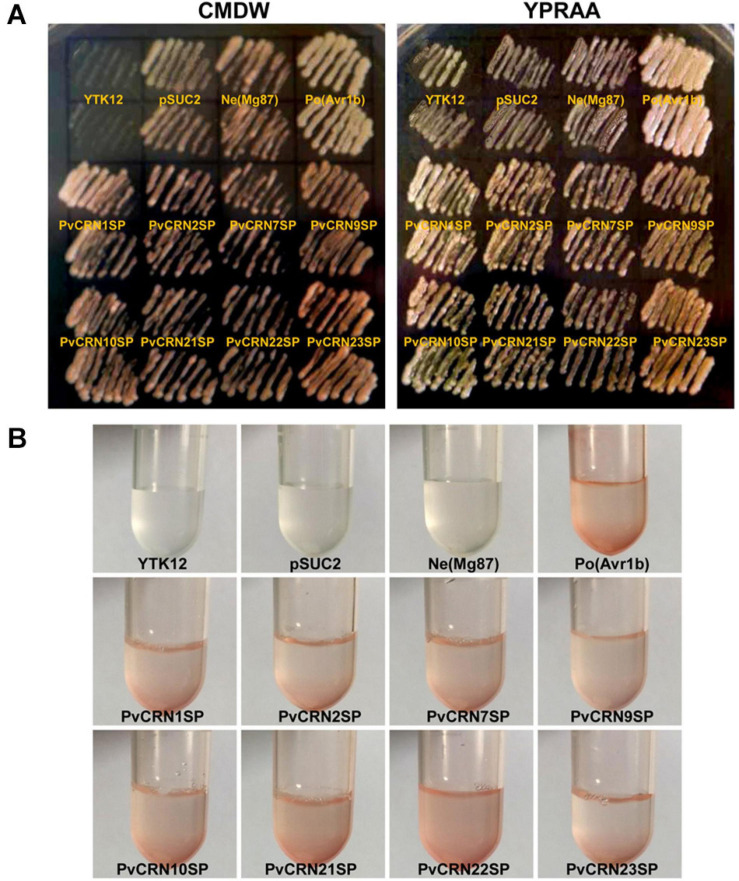
Functional validation of the predicted signal peptides of the PvCRN proteins. **(A)** Yeast growth assay on CMD-W media (left) and YPRAA media (right). **(B)** Yeast invertase secretion assay for the predicted signal peptides of the indicated PvCRN proteins.

Eight predicted signal peptide coding sequences of the *PvCRN* gene family were cloned into the yeast invertase vector pSUC2 and were subsequently transformed into the invertase-negative yeast strain YTK12. Yeast transformants expressing PvCRN signal peptides (PvCRN-SP), including PvCRN1-SP, PvCRN9-SP, PvCRN10-SP, and PvCRN23-SP, were able to grow rapidly on CMD-W and YPRAA media; the positive control (Avr1b) was also able to grow well on these media. Compared to the positive control, the transformants expressing PvCRN2-SP, PvCRN7-SP, PvCRN21-SP, PvCRN22-SP, and the negative control exhibited negligible growth (Figure 3A). However, in the invertase activity assay, all the predicted signal peptides of the PvCRN proteins transported invertase from yeast transformants into the sucrose solution, leading to the color reaction (Figure 3B). Overall, at least four of the eight predicted PvCRN-SPs were validated to secrete proteins.

### Subcellular Localization in the Plant Cell of PvCRN Proteins

**FIGURE 4:**
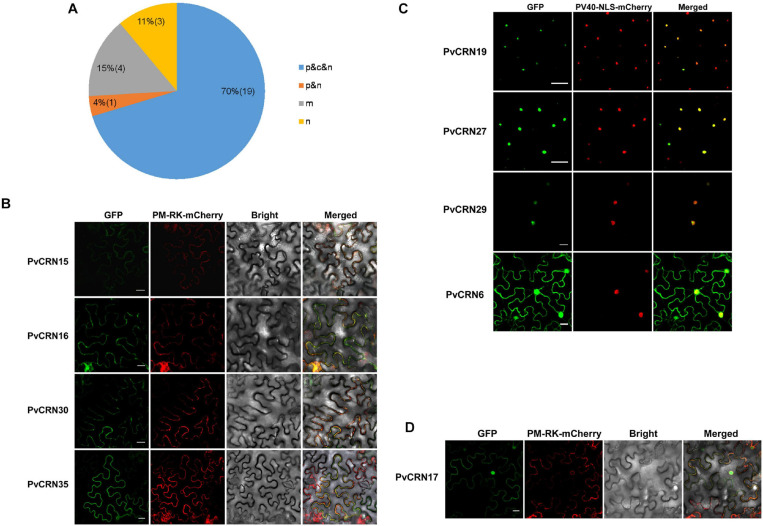
PvCRN proteins are distributed among different plant cell compartments. PvCRN proteins labeled with GFP were transiently expressed in *N. benthamiana* leaves to determine their subcellular localization in the plant cell by confocal microscopy at 48–72 h post agroinfiltration. p, plasma membrane; c, cytoplasm; n, nucleus; m, membrane. **(A)** A summary of statistics of subcellular localizations of PvCRN proteins. **(B)** PvCRN15, PvCRN16, PvCRN30, and PvCRN35 were localized in the membrane system of the host cell, and PM-RK was used as a membrane localization marker. **(C)** PvCRN19, PvCRN27, and PvCRN29 were localized in the nucleus of the plant cell. PvCRN6 was one of the PvCRN proteins that were localized in the plasma membrane, cytoplasm, and nucleus of the plant cell. **(D)** PvCRN17 was distributed among both the plasma membrane and nucleus of the plant cell. The scale bar indicates 20 μm.

With GFP fused to the C-terminus of each PvCRN protein (excluding the signal peptide) expressed in *N. benthamiana* leaves, the subcellular localization of PvCRN proteins was determined by fluorescence microscopic imaging. The results showed that most PvCRN proteins (19 out of 27) were diffusely localized in the plasma membrane, cytoplasm, and nucleus of the plant cell (Figure 4A and Supplementary Figure 7). Moreover, four PvCRN proteins were mainly distributed in the plant cell plasma membrane, including PvCRN15, PvCRN16, PvCRN30, and PvCRN35. In addition, PvCRN15 and PvCRN35 were probably localized in both the plasma membrane and the nuclear envelope (Figure 4B). Only three PvCRN proteins (PvCRN19, PvCRN27, and PvCRN29) with high probabilities of NLS presence (Table 2) were specifically localized in the plant cell nucleus (Figure 4C). Particularly, PvCRN17 was mainly localized in the plasma membrane and nucleus of the plant cell (Figure 4D). To confirm the membrane and nuclear localization of the proteins, some PvCRN proteins were selected to verify their co-localization with the membrane protein PM-RK linked with mCherry or the PV40-NLS-guided mCherry protein, respectively. Most PvCRN proteins were localized in the plasma membrane and nucleus, in agreement with the presence of transmembrane helices and predicted NLS.

### Cell Death-Inducing Activities of the PvCRN Proteins

**FIGURE 5:**
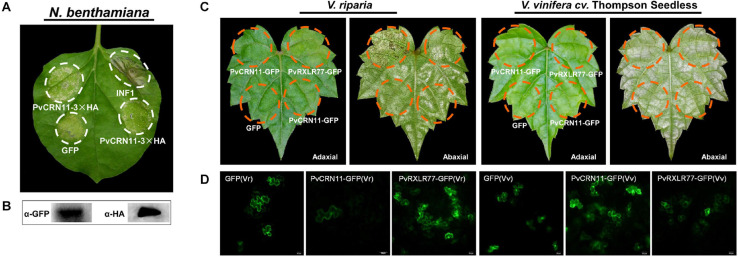
PvCRN11 induced cell death in *N. benthamiana* leaves but failed to induce cell death in *V. riparia* and *V. vinifera* cv. Thompson seedless leaves. **(A)** PvCRN11 induced spotted necrosis on *N. benthamiana* leaves. Constructs PVX-*GFP* and PVX-*INF1* were transiently expressed along with PVX1-*PvCRN11* in *N. benthamiana* leaves as the negative and positive controls for plant cell death induction, respectively. The expression of each construct was verified by western blot, except for INF1 since the plant cell death phenomena induced by PVX-*INF1* were typical, even though there was no accessible corresponding antibody **(B)**. Photographs were taken 5–8 days after agroinfiltration. **(C)** PvCRN11 did not induce HR-like cell death on *V. riparia* (left) or *V. vinifera* cv. Thompson seedless (right) leaves. PvRXLR77-GFP and GFP were expressed in grapevine leaves simultaneously with PvCRN11-GFP, and their corresponding resulting phenomena served as the positive and negative controls, respectively. The expression of each recombinant protein was confirmed through observing GFP fluorescence in the infiltrated leaves 72–120 h post agroinfiltration **(D)**. Photographs of grapevine leaves were taken 7–10 days after agroinfiltration. The experiment was repeated at least three times with at least six leaves tested in each independent experiment.

Pathogen effectors are recognized by host plant R proteins directly or indirectly during long-term co-evolution between plants and their pathogens. Upon recognition of the avirulent effector (Avr) by the plant NB-LRR protein (R), hypersensitive response (HR) is induced and usually coupled with local cell death in the host plant, preventing further biotrophic pathogen propagation (Petit-Houdenot and Fudal, 2017). Hence, it is important to identify plant disease resistance-related components or R genes to identify the cell death-inducing effectors of biotrophic pathogens. The North American *Vitis* species, *V. riparia*, is partially resistant to *P. viticola*, and most of its accessions show HR-related necrosis after infection by *P. viticola* (Cadle-Davidson, 2008; Gómez-Zeledón et al., 2013). The cell death-inducing activity of PvCRN proteins was tested on leaves of *N. benthamiana* and *V. riparia* with the aim of identifying some Avr-like effectors. As shown in Table 4 and Supplementary Figure 8, most PvCRN proteins (26 out of 27) did not induce plant cell death in *N. benthamiana* leaves, whereas INF1, the positive control protein, did induce cell death. No PvCRN proteins were able to induce veritable cell death in *V. riparia* leaves besides PvRXLR77 (Table 4 and Supplementary Figure 9), which is the ortholog of RXLR_PVITv1008311 in *P. viticola* isolate PvitFEM01 and reported to elicit HR-like cell death in *V. riparia* (Brilli et al., 2018). In particular, PvCRN11 induced spotted necrosis on *N. benthamiana* leaves but left no apparent phenotypic changes on *V. riparia* and *V. vinifera* cv. Thompson Seedless leaves (Figure 5). To confirm that PvCRN proteins were expressed in grapevine leaves, the GFP fluorescence of each PvCRN-GFP recombinant protein was verified prior to recording the phenotype of the infiltrated area (Supplementary Figure 10). Overall, the result of the test for cell death-inducing activity on *V. riparia* among the PvCRN proteins was in accordance with the result that most PvCRN proteins failed to induce necrosis on *N. benthamiana*. Even when overexpressed in grapevine leaves, no PvCRN protein induced apparent phenotypic changes in the host.

**TABLE 4:** A summary of plant cell death regulation by PvCRN proteins.

**Regulatory effect on plant cell death (CD)**	**On *N. benthamiana***	**On *V. riparia***
Induce CD	1	None
Suppress INF1-CD	1	_
Suppress Bax-CD	15	_
Neither induce nor suppress CD	12	_

*“_” means not detected.*

### Cell Death-Suppression Activity of the PvCRN Proteins

It is beneficial for plants to execute autonomous cell death to prevent the colonization of biotrophic and semi-biotrophic pathogens. For example, local necrosis or hypersensitive cell death is observed when PTI or ETI is triggered in a plant. Despite the fact that different plants are infected by different pathogens, the PTI responses in different plants share conserved molecular mechanisms (Boller and Felix, 2009; Faulkner and Robatzek, 2012; Bigeard et al., 2015), which are aimed at achieving similar outcomes. Programmed cell death (PCD) in plants is one of the manifestations of hypersensitive response and shares conserved hallmarks and some conserved regulators with PCD in animals (Reape and McCabe, 2008, 2010). For example, Bax, a proapoptotic protein from mice, activates PCD in some plant species, such as *Nicotiana* species and *Arabidopsis thaliana* (Lacomme and Santa Cruz, 1999; Kawai-Yamada et al., 2001; Yoshinaga et al., 2005). However, *V. riparia* leaves transiently overexpressing Bax-GFP recombinant protein simply turned brown on the abaxial side but did not show strong necrosis (Supplementary Figures 9, 10), probably because Bax-induced PCD in *Vitis* species depends on additional factors such as sufficient light (Yoshinaga et al., 2005) or biotrophic pathogen challenge. To overcome these plant defense responses and ensure sustainable access to nutrients, biotrophic pathogens secrete virulent effectors to inhibit cell death processes, keeping the host tissue alive.

It has been well documented that HR-like cell death is highly important for plant resistance to biotrophic pathogens (Glazebrook, 2005; Zebell and Dong, 2015). In accordance with that, biotrophs produce virulent effectors that deactivate host cell death responses (Chaudhari et al., 2014). To determine the virulence of the PvCRN proteins, each *PvCRN* gene was co-expressed with one of the cell death inducers: INF1or Bax. With INF1 expressed slightly later than each PvCRN protein in *N. benthamiana* leaves, the ability of PvCRN proteins to suppress PTI-related cell death was measured. Most PvCRN proteins failed to suppress cell death triggered by INF1 (INF1-CD); only PvCRN20 could completely suppress INF1-CD in *N. benthamiana* leaves (Table 4 and Supplementary Figure 11). In addition, PvCRN2, PvCRN16, and PvCRN17 partially suppressed or delayed INF1-CD (Supplementary Figure 11).

As for the suppressive effects of PvCRN proteins on Bax-induced plant cell death (Bax-CD), each *PvCRN* gene was delivered by *A. tumefaciens* at the same time as the *Bax* gene and 12 h before *Bax*. All PvCRN proteins failed to block Bax-CD when each of them was expressed simultaneously with Bax. However, when each PvCRN protein was able to accumulate earlier than Bax, some showed an inhibitory effect on Bax-CD. Altogether, 15 PvCRN proteins significantly suppressed plant Bax-induced PCD (Table 4 and Supplementary Figure 11). Overall, PvCRN20 fully prevented cell death induced by either INF1 or Bax in *N. benthamiana* leaves. Meanwhile, both PvCRN16 and PvCRN17 attenuated cell death induced by INF1or Bax in *N. benthamiana* leaves.

### Effect of PvCRN Proteins on the Susceptibility of *N. benthamiana* to *P. capsici*

**FIGURE 6:**
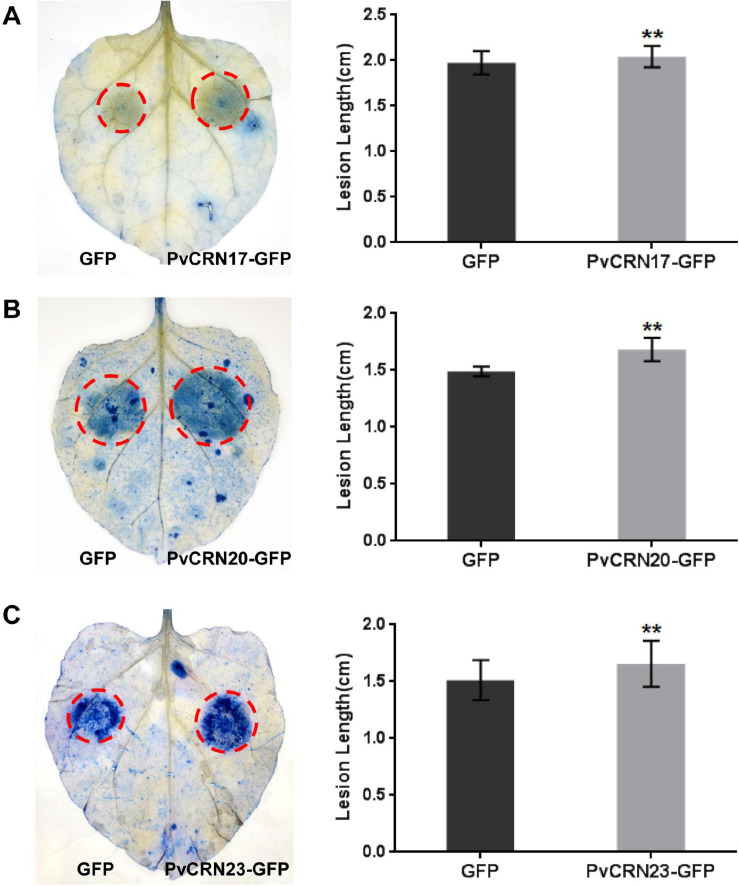
Transient expression of *PvCRN17*, *PvCRN20* and *PvCRN23* in *N. benthamiana* leaves promoted *P. capsici* colonization. **(A–C)** indicate the results for *PvCRN17*, *PvCRN20*, and *PvCRN23*, respectively. **Left panel**: representative inoculated *N. benthamiana* leaves stained with trypan blue. The lesions are outlined with red circles. **Right panel**: mean lesion lengths of *N. benthamiana* leaf regions in which each PvCRN or the control GFP were expressed. The heights of rectangles represent the mean lesion lengths. Error bars represent SD of 9–12 samples. Asterisks indicate significant differences from the control groups (paired *t*-test; **p < 0.01). Similar results were obtained from at least three independent experiments.

**FIGURE 7:**
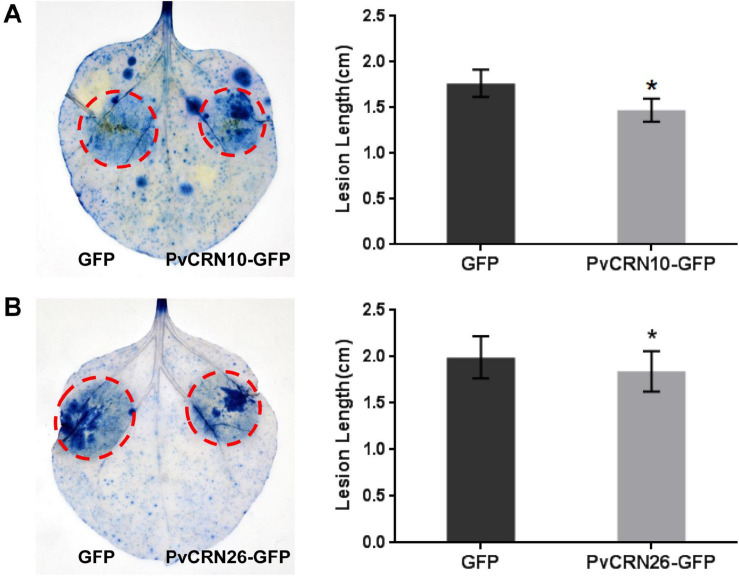
*PvCRN10* and *PvCRN26* enhanced the resistance of *N. benthamiana* to *P. capsici* when transiently expressed in *N. benthamiana.* **(A)** Results for *PvCRN10*, **(B)** Results for *PvCRN26*. **Left panel**: representative inoculated *N. benthamiana* leaves stained with trypan blue. The lesions are outlined with red circles. **Right panel**: mean lesion lengths of *N. benthamiana* leaf regions that were expressing the control GFP plus either PvCRN10 or PvCRN26. The heights of rectangles represent the mean lesion lengths. Error bars represent SD of 9–12 samples. Asterisks indicate significant differences from the control groups (paired *t*-test; *p < 0.05). Similar results were obtained in three independent experiments.

As 15 PvCRN proteins showed the ability to suppress plant cell death, thus contributing to plant resistance to (semi-) biotrophic pathogens, their effects on the susceptibility of *N. benthamiana* to the semi-biotrophic pathogen *P. capsici* were measured to further assess their virulence. Among the PvCRN proteins that suppressed Bax-CD, PvCRN17, PvCRN20, and PvCRN23 produced lesions with lengths that were significantly greater than those of the control GFP (Figure 6 and Table 5), thus promoting *P. capsici* colonization of *N. benthamiana* leaves. In contrast, PvCRN10 and PvCRN26 enhanced the resistance of *N. benthamiana* leaves to *P. capsici* (Figure 7 and Table 5). The other 10 PvCRN proteins showed no effect on the resistance of *N. benthamiana* leaves to *P. capsici* (Table 5 and Supplementary Figure 12). In addition, PvCRN1, a PvCRN protein that neither induced plant cell death nor suppressed INF1- or Bax-induced plant cell death, promoted resistance to *P. capsici* in *N. benthamiana* leaves. In addition, PvCRN31, which contains a protein kinase domain (Prosite entry PS50011) at its C-terminus, did not affect the susceptibility of *N. benthamiana* leaves to *P. capsici* (Supplementary Figure 13).

**TABLE 5:** The effect on the resistance of *N. benthamiana* to *P. capsici* of selected *PvCRNs.*

***PvCRN Gene***	**Significantly constrains colonization by *P. capsici* on *N. benthamiana***	**Significantly promotes colonization by *P. capsici* on *N. benthamiana***
*PvCRN10*	Yes	No
*PvCRN12*	No	No
*PvCRN14*	No	No
*PvCRN15*	No	No
*PvCRN16*	No	No
***PvCRN17***	No	Yes
*PvCRN18*	No	No
***PvCRN20***	No	Yes
*PvCRN22*	No	No
***PvCRN23***	No	Yes
*PvCRN24*	No	No
*PvCRN25*	No	No
*PvCRN26*	Yes	No
*PvCRN30*	No	No
*PvCRN35*	No	No

*PvCRN genes found to significantly promote the susceptibility of *N. benthamiana* to *P. capsici* are highlighted with their names in bold.*

**FIGURE 8:**
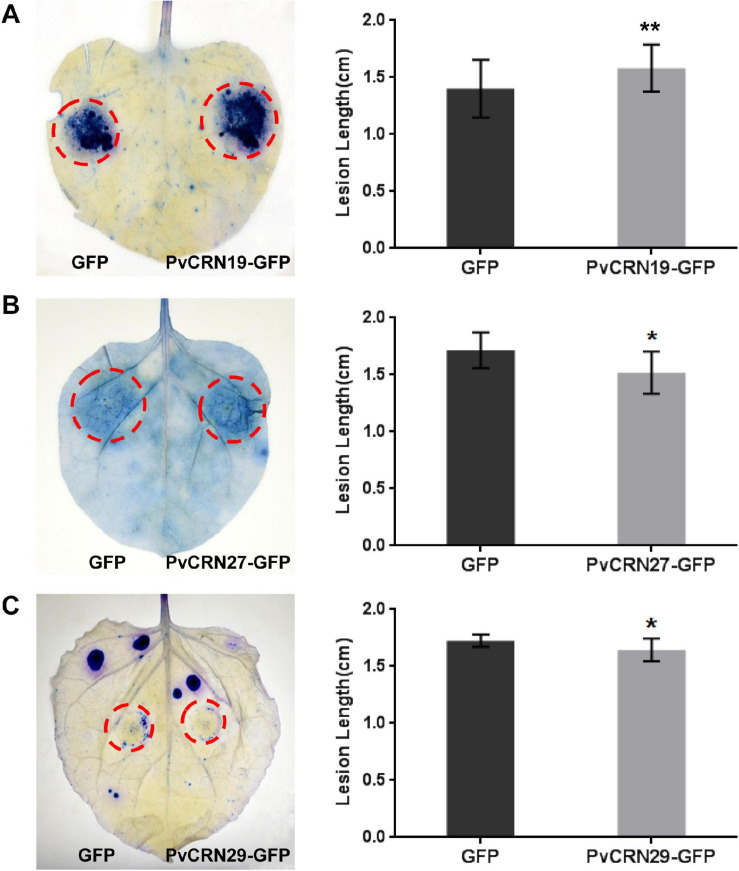
Plant nuclear localized proteins PvCRN19, PvCRN27, and PvCRN29 have different effects on the colonization of *N. benthamiana* by *P. capsici.* PvCRN19 made *N. benthamiana* leaves more susceptible to *P. capsici*, whereas PvCRN27 and PvCRN29 showed the opposite effect. **(A–C)** indicate the results for PvCRN19, PvCRN27, and PvCRN29, respectively. **Left panel**: representative inoculated *N. benthamiana* leaves stained with trypan blue. The lesions are outlined with red circles. **Right panel**: mean lesion lengths of *N. benthamiana* leaf regions in which each PvCRN or the control GFP were expressed. The heights of rectangles represent the mean lesion lengths. Error bars represent SD of 9–12 samples. Asterisks indicate significant differences from the control groups (paired *t*-test; *p < 0.05; **p < 0.01). Similar results were obtained in three independent experiments.

The nucleus-localized PvCRN proteins PvCRN19, PvCRN27, and PvCRN29 had different effects on disease resistance in *N. benthamiana*. PvCRN19 significantly enhanced the susceptibility of *N. benthamiana* leaves to *P. capsici*, whereas PvCRN27 and PvCRN29 improved the resistance level of *N. benthamiana* to the pathogen (Figure 8). This suggests that they differed in virulence, although these proteins were all translocated to the plant nucleus.

**FIGURE 9:**
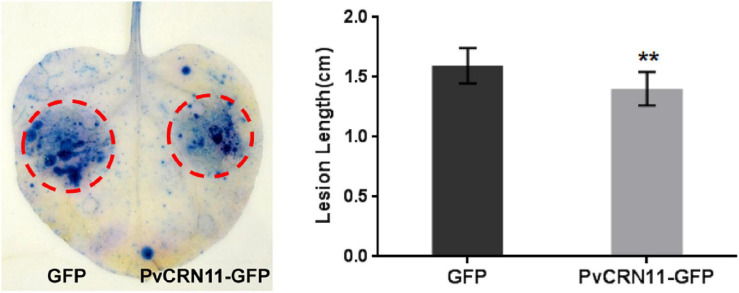
The *PvCRN11*gene enhanced the resistance of *N. benthamiana* to *P. capsici* when transiently expressed in *N. benthamiana.* **Left panel**: representative inoculated *N. benthamiana* leaves stained with trypan blue. The lesions are outlined with red circles. **Right panel**: mean lesion lengths of *N. benthamiana* leaf regions in which PvCRN11 or the control GFP were expressed. The heights of rectangles represent the mean lesion lengths. Error bars represent SD of nine samples. Asterisks indicate significant differences from the control groups (paired *t*-test; **p < 0.01). Similar results were obtained in three independent experiments.

In addition, PvCRN11, which is the only one that induced necrosis in *N. benthamiana*, repressed the extension of *P. capsici* lesions on *N. benthamiana* leaves (Figure 9). This indicates that PvCRN11 is recognized by host plants and triggers host defense responses.

### Transcription Levels of *PvCRN* Genes During *P. viticola* YL Infection of Grapevines

**FIGURE 10:**
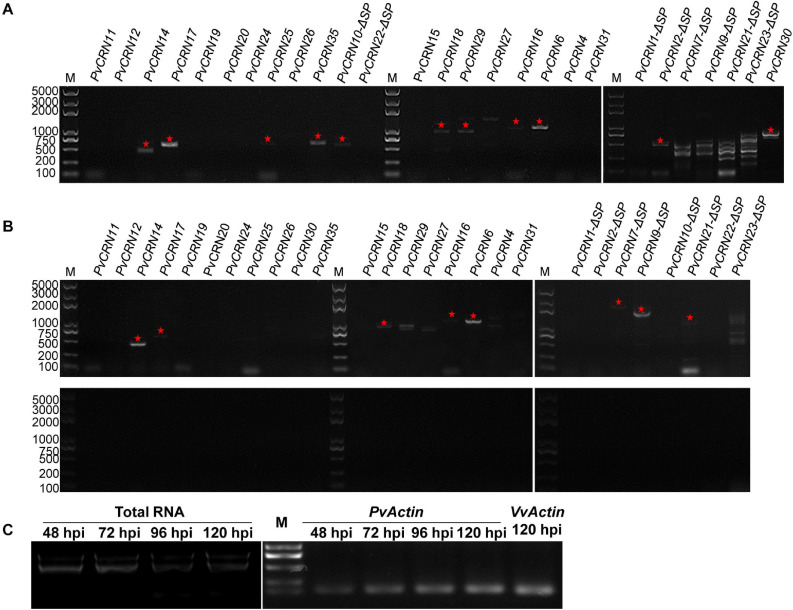
Detection of the transcription level of *PvCRN* genes during infection of *P. viticola* YL on *V. vinifera* Pinot Noir by RT-PCR. **(A)** Transcriptional analysis of the *PvCRN* family in *P. viticola* YL sporangia 96 h post inoculation (96 hpi) on Pinot Noir leaves. The corresponding DNA band of each detectable *PvCRN* gene was marked with a pentagram. **(B)** Transcriptional analysis of *PvCRN* genes at 72 hpi (upper) and 120 hpi (lower). **(C)** Detection of the total RNA and cDNA prepared from Pinot Noir leaves inoculated with *P. viticola* YL. Experiments were repeated three times with similar results.

The transcription pattern of each *PvCRN* gene likely reflects the involvement of these genes in the interaction between *P. viticola* and the grapevine. The transcription levels of the PvCRN genes were detected by RT-PCR at 96 hpi, which was selected because the hyphae and haustoria of *P. viticola* were anticipated to have already formed by 48–72 hpi (Unger et al., 2007; Yin X. et al., 2017). As shown in Figure 10, at 96 hpi, 11 out of 27 *PvCRN* genes were expressed at relatively high levels, and these included *PvCRN10*, *PvCRN14*, *PvCRN16*, *PvCRN17*, *PvCRN18*, *PvCRN30*, *PvCRN35, PvCRN2*, *PvCRN6*, *PvCRN25*, and *PvCRN29*. To identify more *PvCRN* genes that were significantly transcribed during infection, RT-PCR was conducted with the mixed mRNA from inoculated grapevine leaves at 72 and 120 hpi. The mRNAs for a few *PvCRN* genes were detectable by RT-PCR at 72 hpi. Transcripts of *PvCRN7*, *PvCRN9*, and *PvCRN21*, which are PvCRNs with signal peptides, were detectable at 72 hpi rather than 96 hpi. *PvCRN14*, *PvCRN16*, *PvCRN17*, and *PvCRN6* continued to be expressed between 72 and 96 hpi. It is intriguing that none of the 27 *PvCRN* genes studied showed evident accumulation of their mRNAs at 120 hpi (Figure 10).

Taken together, PvCRN14, PvCRN16, and PvCRN17 may be the strategic effectors of *P. viticola*, as evidenced by their high expression levels at 72–96 hpi and their suppression of Bax-induced PCD. PvCRN23 suppressed Bax-CD and promoted the susceptibility of *N. benthamiana* to a semi-biotrophic pathogen *P*. *capsici*, which encodes CRN proteins with low similarities to those of *P. viticola*, suggesting these contribute to the pathogenicity of *P. viticola*. PvCRN20 is another impressive effector that suppressed plant HR-like cell death triggered by INF1 or Bax and promoted *P. capsici* colonization of *N. benthamiana*. PvCRN19 increased the pathogenicity of *P. capsici* despite its inability to suppress the plant HR-like cell death induced by INF1 and Bax. PvCRN10 and PvCRN26 increased resistance in *N. benthamiana*, which was at odds with their abilities to prevent cell death associated with plant defense responses to pathogens. Therefore, the virulence of PvCRN proteins is quite complicated to determine based on the metrics above.

## Discussion

CRN-like proteins are a large ancient class of vital proteins conserved in many eukaryotic organisms, including oomycetes, such as *Phytophthora* species and *Plasmopara* species, some fungal pathogens (such as *Batrachochytrium dendrobatidis* and *Rhizophagus irregularis*), and some members of *Viridiplantae*, such as chlorophytes, cryptophyte algae, and choanoflagellates. However, to date, little has been reported regarding the detailed roles of most *CRN* genes in these organisms. As for *CRN* genes of *Plasmopara viticola*, the number of *CRN-like* genes was reported to be 90 in the isolate JL-7-2 collected from Jilin province, China (Yin L. et al., 2017), 68 in the isolate PvitFEM01 from Northern Italy (Brilli et al., 2018), and 35 in the isolate YL collected in Yangling, China in this study. However, a comprehensive characterization of the *PvCRN* genes in each of the previous two isolates was not performed. In this study, the 27 *CRN-like* genes of strain YL were cloned and characterized. Sequence alignment showed that all *PvCRN* genes in YL share high similarities with the genomic DNA sequences of the other three previously sequenced *P. viticola* isolates (Dussert et al., 2016; Yin L. et al., 2017; Brilli et al., 2018). Meanwhile, there were single nucleotide differences between most *PvCRNs* in ‘YL’ and their orthologs in the other isolates mentioned above. This is believed to reflect pathogen adaption to different grapevine genotypes in different geographic areas (Yin L. et al., 2017). Several pairs of *PvCRN* genes were determined to be generated by gene duplication, which has increased the number of *PvCRN-like* genes. Some *PvCRN* genes were recombinant products of the recombination of at least two other *PvCRN* genes, which has led to the diversification of the *PvCRN* family. Therefore, different clades of *PvCRN* genes have been formed in ‘YL.’ With the evolution of *PvCRN* genes, additional features and functions could be acquired by this family. In a phylogenetic comparison with CRN-like proteins from *P. halstedii*, the most closely related species to *P. viticola*, no PvCRN proteins clustered with PhCRN proteins. This agrees with a previous comparative analysis of CRN effectors from *P. viticola* and *P. halstedii* (Mestre et al., 2016). Therefore, there appear to be significant differences in the functions of *CRN* superfamily genes between the two *Plasmopara* species.

Compared with CRN proteins in *Phytophthora* spp., PvCRN proteins show only two subdomains (new DXV and DBE) in their C-terminal region from the collection of the C-terminal subdomains in *Phytophthora* species (Haas et al., 2009; Stam et al., 2013). The CRN C-terminal subdomains are thought to be correlated with hemi-biotrophy and necrotrophy of oomycetes. These effector-associated domains were found to reside widely in hemi-biotrophic *Phytophthora* spp. and the necrotrophic pathogen *Pythium ultimum*, but were not found in *Hyaloperonospora arabidopsis*, a biotrophic pathogen (Stam et al., 2013). These discoveries may explain why few subdomains were found in *P. viticola* YL, which is an obligate biotrophic pathogen. In addition, several PvCRN proteins contain some known conserved functional domains or patterns in their C-terminal regions, such as a protein kinase domain (Prosite entry PS50011) and HNH_2 motif, which may contribute to certain effector activities. The yeast signal sequence trap assay showed that at least four different predicted signal peptides, in PvCRN1, PvCRN9, PvCRN10, and PvCRN23, were validated signal peptides. In agreement with the characterization of CRN proteins in the previously sequenced organisms, most PvCRN proteins did not have canonical signal peptides. Considering that CRN proteins are also found in pathogens that do not form haustoria and that the conserved N terminuses of CRN proteins can deliver the effector domain of AVR3a into plant cells (Schornack et al., 2010; Zhang et al., 2016), this study provides further evidence that the conserved N-terminal region of CRN proteins possesses the ability to translocate pathogen-produced effectors into host plant cells.

It is interesting that most PvCRN proteins were probably distributed in the plasma membrane, cytosol, and nucleus of the *N. benthamiana* cells (Figure 4). This suggests that there is more than one target molecule or more than one destination in the host cell for these effectors. However, it was difficult to distinguish between recombinant protein localization in the plasma membrane and in the cytosol using fluorescence imaging, even with co-expressed localization marker genes. The amount of each transiently expressed protein that had accumulated could not be ensured to be the same, which could introduce error in the determination of the bona localization of PvCRN proteins in the plant cell. Notably, 10 members of the PvCRN protein family mentioned above are equal to or smaller than the tag protein eGFP in size (Table 2), which would make it possible for the distribution of the recombinant proteins to be guided by the widely dispersed eGFP. In fact, some PvCRN proteins could not be detected by western blot if the corresponding total protein samples were extracted by protein extraction buffer without sodium deoxycholate (data not shown). This requires further validation of the membrane localization of PvCRN proteins. With NLS predicted and GFP fluorescence observed in the nucleus of *N. benthamiana* and *V. riparia* cells (Table 2, Figure 4, and Supplementary Figure 10), PvCRN19, PvCRN27, and PvCRN29 were localized in the nucleus of the plant cell. In addition, several PvCRN proteins without classical NLS, such as PvCRN10, PvCRN23, and PvCRN25, were also found to target the plant nucleus, suggesting alternative means for the nuclear translocation of PvCRN proteins (Amaro et al., 2017). In summary, it is conducive to determine the distributions of PvCRN effectors in the plant cell to estimate their potential targets in the host and their possible functions.

Findings in this study make it more convincing that CRN proteins are cell death regulators rather than inducers, as cell death-inducing activity is not a prevalent feature of these proteins (Amaro et al., 2017). That PvCRN proteins in grapevine did not induce obvious cell death is likely due to variation in the *PvCRN* genes driven by positive selection during pathogen evolution (Shen et al., 2013), as well as their low expression levels (Supplementary Figure 10). In addition, a direct correlation between necrosis induction and effector virulence has not been demonstrated for CRN proteins. To date, most Avr proteins identified in oomycetes belong to RXLR effectors (Tyler and Rouxel, 2012), such as ATR39-1 (Goritschnig et al., 2012), ATR1^*NdWsB*^ (Rehmany et al., 2005), ATR13 (Allen et al., 2004), ATR5 (Bailey et al., 2011), Avr2 (Lokossou et al., 2009), Avr3a (Armstrong et al., 2005), Avr4 (Van Poppel et al., 2009), Avrblb2 (Oh et al., 2009), Avr1b-1 (Shan et al., 2004), and Avr4/6 (Dou et al., 2010). However, it remains unclear whether PvCRN proteins are recognized by host R proteins directly or indirectly, depending on the changes in appearance of grapevine leaves. The approach to identifying resistance-related components of grapevine by overexpression of PvCRN proteins *in planta* remains to be improved at the molecular level.

Pathogen effectors are thought to interfere with the PTI of the host, promoting colonization by the pathogen. PTI is induced by pathogen-associated molecular patterns (PAMPs), such as flagellin (or its derivative flg22) and EF-Tu from some bacterial pathogens and INF1 and XEG1 from oomycete pathogens (Gómez-Gómez and Boller, 2000; Zipfel et al., 2006; Du et al., 2015; Ma et al., 2015; Wang et al., 2019). Moreover, plant-derived molecular patterns, known as damage-associated molecular patterns (DAMPs), such as plant elicitor peptides (Peps), also elicit immune responses similar to those of PTI (Bigeard et al., 2015). It has been demonstrated that PTI triggered by these PAMPs and DAMPs share conserved signal transduction pathways, as plant BAK1/SERK3 serves as the indispensable co-receptor of the pattern-recognition receptors (PRRs) (Heese et al., 2007; Chaparro-Garcia et al., 2011; Wang et al., 2019). Furthermore, reactive oxygen species (ROS) production, Ca^2+^ signaling, and activation of a mitogen-activated protein kinase (MAPK) cascade are the general responses following PAMP recognition (Bigeard et al., 2015). Based on the above knowledge, INF1-triggered plant cell death was conducted to mimic the PTI of the host plant, and the PTI suppression activity of each PvCRN protein was determined in *N. benthamiana*. This indicates that PvCRN20, PvCRN2, PvCRN16, and PvCRN17 are likely powerful effectors for *P. viticola* to counteract PTI in grapevines.

Programmed cell death is a common strategy utilized by animals and plants to regulate defense and development. The PCD process has been presumed to be conserved among eukaryotes as the participation of caspase-like proteases, the involvement of mitochondria for cytochrome c release, and the accumulation of ROS are common features of eukaryotic PCD (Jones, 2000; Reape and McCabe, 2008, 2010). PCD can be induced by the mammalian proapoptotic protein Bax in both animals and plants (Yoshinaga et al., 2005). Therefore, Bax-CD was introduced to mimic the PCD of the host plants, and the effects of the PvCRN proteins on PCD were measured. Remarkably, PvCRN20 was able to suppress Bax-CD and INF1-CD, suggesting that PvCRN20 inhibit various defense responses of the host plant. In addition, some PvCRN proteins that neither partially nor completely inhibited INF1-CD were capable of inhibiting PCD (Supplementary Figure 11), these PvCRN proteins may interfere with a limited number of signaling pathways of plant defense responses to the biotrophic organism *P. viticola*.

As it is difficult to genetically manipulate *P. viticola* and grapevine (Dubresson et al., 2008; Reustle and Buchholz, 2009), high-throughput characterization of genes of interest in the two species may not be possible. The effect of PvCRN proteins on the virulence of *P. viticola* was explored by transient expression of each PvCRN gene in *N. benthamiana* leaves following inoculation with *Phytophthora capsici*, which is hemi-biotrophic (Lamour et al., 2012b). Sequence alignment showed that CRN effectors of *P. capsici* shared low identities with PvCRN proteins (data not shown). The virulence of PvCRN proteins was assessed indirectly by their effects on the susceptibility of *N. benthamiana* to *P. capsici*. This measurement system worked in this study, as PvCRN17, PvCRN19, PvCRN20, and PvCRN23 promoted susceptibility in *N. benthamiana*, whereas PvCRN1, PvCRN10, and PvCRN26 seemed to enhance the disease resistance of *N. benthamiana* to *P. capsici*. Therefore, the potential principal PvCRN effectors involved in the interaction between *P. viticola* and grapevine might be screened in this way.

The transcription levels of the *PvCRN* genes were roughly determined by RT-PCR in this study, considering the difficulty in precisely measuring the expression level of genes of interest from a population with continuous proliferation. *PvCRN14*, *PvCRN16*, *PvCRN17*, and *PvCRN6* showed obvious transcription at 72 and 96 hpi (Figure 10). However, this may merely result from the complex interaction between *P. viticola* and grapevine (*V. vinifera* Pinot Noir). Thus, the quantity of mRNA accumulating for each *PvCRN* may not fully reflect its importance to the progress of *P. viticola* infection. On the other hand, it has been reported that *P. viticola* generates small RNAs derived from CRN genes, as *Phytophthora infestans* did, including one that was predicted to target some *V. vinifera* genes (Vetukuri et al., 2012; Brilli et al., 2018). Combined with the results of this study, this indicates another way in which *PvCRN* genes function in the interaction between *P. viticola* and grapevine.

This study provides an overview of the basic characteristics of *PvCRN* genes in *P. viticola* YL, which provides a foundation for a comprehensive understanding of the role and biology of *CRN-like* genes. For *PvCRN* genes, their virulence remains to be determined in the *P. viticola* – grapevine system. Nevertheless, a rough screen of *PvCRN* genes mainly in model plant *N. benthamiana* still has value as a reference for narrowing down the search range for candidate *PvCRN* genes with crucial roles. This study found that PvCRN17, PvCRN19, PvCRN20, and PvCRN23 may be pathogenesis-related effectors against PvCRN11, which was ineffective for pathogenicity. The contribution to the pathogenicity of *P. viticola* of the ‘seeded’ *PvCRN* genes mentioned above and the targets of these PvCRN proteins in *Vitis* species remain to be discovered in future research aimed at elucidating the pathogenic mechanisms of *P. viticola*.

## Data Availability Statement

The datasets presented in this study can be found in online repositories. The names of the repository/repositories and accession number(s) can be found in the article/Supplementary Material.

## Author Contributions

YX conceived the study. GX, XY, WN, TC, and RL conducted the experiments. BS contributed to the *in vitro* grapevine plantlets cultivation. QF contributed to the fluorescence observation. GX wrote the manuscript. YX, GL, and HM revised the manuscript. All the authors contributed to the article and approved the submitted version.

## Conflict of Interest

The authors declare that the research was conducted in the absence of any commercial or financial relationships that could be construed as a potential conflict of interest.

## Footnotes

1 https://web.expasy.org/translate/

2 https://web.expasy.org/protparam/

3 http://www.cbs.dtu.dk/services/SignalP/

4 http://phobius.sbc.su.se/

5 http://nls-mapper.iab.keio.ac.jp/cgi-bin/NLS_Mapper_form.cgi

6 https://www.predictprotein.org/

7 https://embnet.vital-it.ch/software/TMPRED_form.html

8 http://weblogo.berkeley.edu/logo.cgi

9 https://itol.embl.de/

10 http://pfam.xfam.org/

11 http://smart.embl-heidelberg.de/smart/set_mode.cgi?NORMAL=1

12 https://prosite.expasy.org/scanprosite/

13 https://www.ncbi.nlm.nih.gov/bioproject/488341
