# Supplementary material for: Characterization of CRN-Like Genes From Plasmopara viticola: Searching for the Most Virulent Ones
Source: Front Microbiol. 2021 Mar 22;12:632047. doi: 10.3389/fmicb.2021.632047 (PMC8044898; doi:10.3389/fmicb.2021.632047)
Supplement: Supplementary file 4 [file Data_Sheet_1.PDF]

Supplementary Material

1 Supplementary Figures

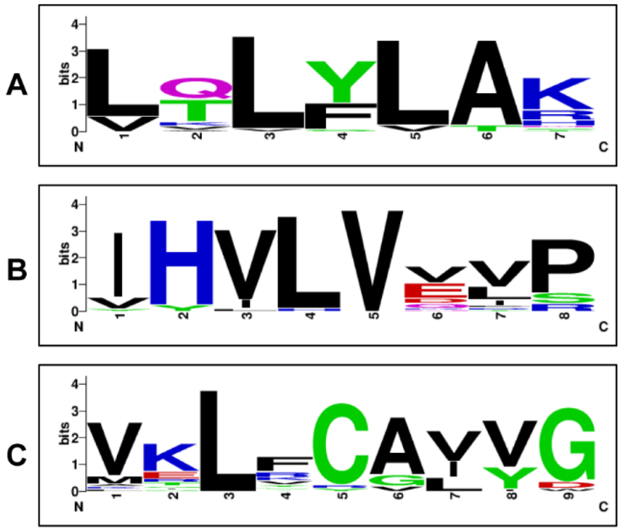

Figure S1. Conserved motifs conserved in the N-terminal sequences of PvCRN proteins. **(A)** The L (/V) -X-L-Y (/F)-L-A-K (/R/H) motif, **(B)** The I-H-V-L-V-X-X-P motif. **(C)** The V-X-L-X-C-A-X-V (/ Y)-G motif. The alphabets were generated by submitting the featured amino acid sequences to the Weblogo server, respectively.

Figure S2. See attached separate image "Figure S2. Multiple sequence alignment of PvCRN proteins".

Figure S3. See attached separate image "Figure S3. DNA sequences alignment of *PvCRN18, PvCRN27* and *PvCRN29*".

Figure S4. See attached separate image "Figure S4. DNA sequences alignment of *PvCRN10* and *PvCRN11*".

Figure S5. See attached separate image "Figure S5. Multiple sequence alignment of mature PvCRN proteins by ClustalX-2.1".

Figure S6. See attached separate image "Figure S6. Multiple sequence alignment of CRN proteins from *P. viticola* isolate YL and *P. halstedii* by ClustalX-2.1".

Figure S7. See attached separate image "Figure S7. Some PvCRN proteins were localized at the plasmamembrane, cytoplasm and nucleus".

Figure S8. See attached separate image "Figure S8. Cell death inducing activity of PvCRN proteins on *Nicotiana benthamiana* leaves".

Figure S9. See attached separate image "Figure S9. Cell death inducing activity of PvCRN proteins on *Vitis riparia* leaves".

Figure S10. See attached separate image "Figure S10. Confirmation of the expression of PvCRN-GFP recombinant proteins in *V. riparia* leaves by observation of the GFP fluorescence".

Figure S11. See attached separate image "Figure S11. Cell death suppressing activity of PvCRN proteins on plant cell death induced by INF1 and Bax".

Figure S12. See attached separate image "Figure S12. Some of the cell death suppressing-PvCRN proteins did not show significant effect on the resistance of *N. benthamiana* to *P. capsici.*"

Figure S13. See attached separate image "*PvCRN1* and *PvCRN31* did not enhance the susceptibility of *N. benthamiana* to *P. capsici.*"

2 Supplementary Tables

Table S1. See attached file "Table S1. Primers used in this study".

Table S2. See attached file "Table S2. Sequences information of *PvCRN* genes in *P. viticola* isolate YL".

Table S3. See attached file "Table S3. Gene recombination analysis of *PvCRN* genes in *P. viticola* isolate YL by RDP5".
