# Supplementary material for: Characterization of CRN-Like Genes From Plasmopara viticola: Searching for the Most Virulent Ones
Source: Front Microbiol. 2021 Mar 22;12:632047. doi: 10.3389/fmicb.2021.632047 (PMC8044898; doi:10.3389/fmicb.2021.632047)
Supplement: Supplementary file 5 [file Data_Sheet_2.PDF]

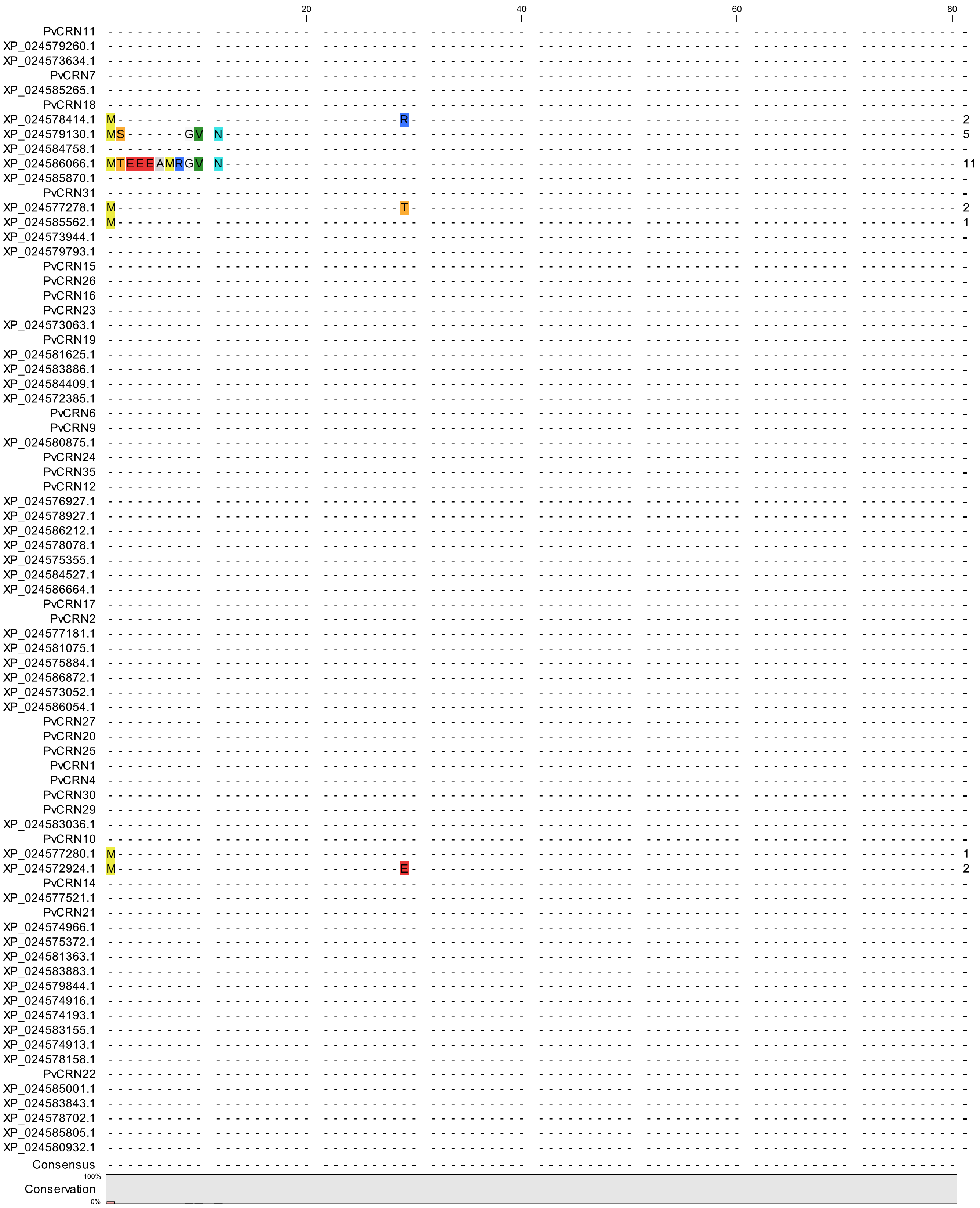

20
40
60
80
PvCRN11
XP_024579260.1
XP_024573634.1
PvCRN7
XP_024585265.1
PvCRN18
XP_024578414.1 M R 2
XP_024579130.1 MS GV N 5
XP_024584758.1
XP_024586066.1 MTEEEAMRGV N 11
XP_024585870.1
PvCRN31
XP_024577278.1 M T 2
XP_024585562.1 M 1
XP_024573944.1
XP_024579793.1
PvCRN15
PvCRN26
PvCRN16
PvCRN23
XP_024573063.1
PvCRN19
XP_024581625.1
XP_024583886.1
XP_024584409.1
XP_024572385.1
PvCRN6
PvCRN9
XP_024580875.1
PvCRN24
PvCRN35
PvCRN12
XP_024576927.1
XP_024578927.1
XP_024586212.1
XP_024578078.1
XP_024575355.1
XP_024584527.1
XP_024586664.1
PvCRN17
PvCRN2
XP_024577181.1
XP_024581075.1
XP_024575884.1
XP_024586872.1
XP_024573052.1
XP_024586054.1
PvCRN27
PvCRN20
PvCRN25
PvCRN1
PvCRN4
PvCRN30
PvCRN29
XP_024583036.1
PvCRN10
XP_024577280.1 M 1
XP_024572924.1 M E 2
PvCRN14
XP_024577521.1
PvCRN21
XP_024574966.1
XP_024575372.1
XP_024581363.1
XP_024583883.1
XP_024579844.1
XP_024574916.1
XP_024574193.1
XP_024583155.1
XP_024574913.1
XP_024578158.1
PvCRN22
XP_024585001.1
XP_024583843.1
XP_024578702.1
XP_024585805.1
XP_024580932.1
Consensus
Conservation
100%
0%

| Sequence | 100 · 120 · 140 · 160 | |
|---|---|---|
| PvCRN11 | ---------- ---------- ---------- ---------- ---------- ---------- ---------- ---------- | - |
| XP_024579260.1 | ---------- ---------- ---------- ---------- ---------- ---------- ---------- ---------- | - |
| XP_024573634.1 | ---------- ---------- ---------- ---------- ---------- ---------- ---------- ---------- | - |
| PvCRN7 | ---------- ---------- ---------- ---------- ---------- ---------- ---------- ---------- | - |
| XP_024585265.1 | ---------- ---------- ---------- ---------- ---------- ---------- ---------- ---------- | - |
| PvCRN18 | ---------- ---------- ---------- ---------- ---------- ---------- ---------- ---------- | - |
| XP_024578414.1 | ---------- ---------- ---------- ---------- ---------- ---------- ---------- ---------- | 2 |
| XP_024579130.1 | ---------- ---------- ---------- ---------- ---------- ---------- ---------- ---------- | 5 |
| XP_024584758.1 | ---------- ---------- ---------- ---------- ---------- ---------- ---------- ---------- | - |
| XP_024586066.1 | ---------- ---------- ---------- ---------- ---------- ---------- ---------- ---------- | 11 |
| XP_024585870.1 | ---------- ---------- ---------- ---------- ---------- ---------- ---------- ---------- | - |
| PvCRN31 | ---------- ---------- ---------- ---------- ---------- ---------- ---------- ---------- | - |
| XP_024577278.1 | ---------- ---------- ---------- ---------- ---------- ---------- ---------- ---------- | 2 |
| XP_024585562.1 | ---------- ---------- ---------- ---------- ---------- ---------- ---------- ---------- | 1 |
| XP_024573944.1 | ---------- ---------- ---------- ---------- ---------- ---------- ---------- ---------- | - |
| XP_024579793.1 | ---------- ---------- ---------- ---------- ---------- ---------- ---------- ---------- | - |
| PvCRN15 | ---------- ---------- ---------- ---------- ---------- ---------- ---------- ---------- | - |
| PvCRN26 | ---------- ---------- ---------- ---------- ---------- ---------- ---------- ---------- | - |
| PvCRN16 | ---------- ---------- ---------- ---------- ---------- ---------- ---------- ---------- | - |
| PvCRN23 | ---------- ---------- ---------- ---------- ---------- ---------- ---------- ---------- | - |
| XP_024573063.1 | ---------- ---------- ---------- ---------- ---------- ---------- ---------- ---------- | - |
| PvCRN19 | ---------- ---------- ---------- ---------- ---------- ---------- ---------- ---------- | - |
| XP_024581625.1 | ---------- ---------- ---------- ---------- ---------- ---------- ---------- ---------- | - |
| XP_024583886.1 | ---------- ---------- ---------- ---------- ---------- ---------- ---------- ---------- | - |
| XP_024584409.1 | ---------- ---------- ---------- ---------- ---------- ---------- ---------- ---------- | - |
| XP_024572385.1 | ---------- ---------- ---------- ---------- ---------- ---------- ---------- ---------- | - |
| PvCRN6 | ---------- ---------- ---------- ---------- ---------- ---------- ---------- ---------- | - |
| PvCRN9 | ---------- ---------- ---------- ---------- ---------- ---------- ---------- ---------- | - |
| XP_024580875.1 | ---------- ---------- ---------- ---------- ---------- ---------- ---------- ---------- | - |
| PvCRN24 | ---------- ---------- ---------- ---------- ---------- ---------- ---------- ---------- | - |
| PvCRN35 | ---------- ---------- ---------- ---------- ---------- ---------- ---------- ---------- | - |
| PvCRN12 | ---------- ---------- ---------- ---------- ---------- ---------- ---------- ---------- | - |
| XP_024576927.1 | ---------- ---------- ---------- ---------- ---------- ---------- ---------- ---------- | - |
| XP_024578927.1 | ---------- ---------- ---------- ---------- ---------- ---------- ---------- ---------- | - |
| XP_024586212.1 | ---------- ---------- ---------- ---------- ---------- ---------- ---------- ---------- | - |
| XP_024578078.1 | ---------- ---------- ---------- ---------- ---------- ---------- ---------- ---------- | - |
| XP_024575355.1 | ---------- ---------- ---------- ---------- ---------- ---------- ---------- ---------- | - |
| XP_024584527.1 | ---------- ---------- ---------- ---------- ---------- ---------- ---------- ---------- | - |
| XP_024586664.1 | ---------- ---------- ---------- ---------- ---------- ---------- ---------- ---------- | - |
| PvCRN17 | ---------- ---------- ---------- ---------- ---------- ---------- ---------- ---------- | - |
| PvCRN2 | ---------- ---------- ---------- ---------- ---------- ---------- ---------- ---------- | - |
| XP_024577181.1 | ---------- ---------- ---------- ---------- ---------- ---------- ---------- ---------- | - |
| XP_024581075.1 | ---------- ---------- ---------- ---------- ---------- ---------- ---------- ---------- | - |
| XP_024575884.1 | ---------- ---------- ---------- ---------- ---------- ---------- ---------- ---------- | - |
| XP_024586872.1 | ---------- ---------- ---------- ---------- ---------- ---------- ---------- ---------- | - |
| XP_024573052.1 | ---------- ---------- ---------- ---------- ---------- ---------- ---------- ---------- | - |
| XP_024586054.1 | ---------- ---------- ---------- ---------- ---------- ---------- ---------- ---------- | - |
| PvCRN27 | ---------- ---------- ---------- ---------- ---------- ---------- ---------- ---------- | - |
| PvCRN20 | ---------- ---------- ---------- ---------- ---------- ---------- ---------- ---------- | - |
| PvCRN25 | ---------- ---------- ---------- ---------- ---------- ---------- ---------- ---------- | - |
| PvCRN1 | ---------- ---------- ---------- ---------- ---------- ---------- ---------- ---------- | - |
| PvCRN4 | ---------- ---------- ---------- ---------- ---------- ---------- ---------- ---------- | - |
| PvCRN30 | ---------- ---------- ---------- ---------- ---------- ---------- ---------- ---------- | - |
| PvCRN29 | ---------- ---------- ---------- ---------- ---------- ---------- ---------- ---------- | - |
| XP_024583036.1 | ---------- ---------- ---------- ---------- ---------- ---------- ---------- ---------- | - |
| PvCRN10 | ---------- ---------- ---------- ---------- ---------- ---------- ---------- ---------- | - |
| XP_024577280.1 | ---------- ---------- ---------- ---------- ---------- ---------- ---------- ---------- | 1 |
| XP_024572924.1 | ---------- ---------- ---------- ---------- ---------- ---------- ---------- ---------- | 2 |
| PvCRN14 | ---------- ---------- ---------- ---------- ---------- ---------- ---------- ---------- | - |
| XP_024577521.1 | ---------- ---------- ---------- ---------- ---------- ---------- ---------- ---------- | - |
| PvCRN21 | ---------- ---------- ---------- ---------- ---------- ---------- ---------- ---------- | - |
| XP_024574966.1 | ---------- ---------- ---------- ---------- ---------- ---------- ---------- ---------- | - |
| XP_024575372.1 | ---------- ---------- ---------- ---------- ---------- ---------- ---------- ---------- | - |
| XP_024581363.1 | ---------- ---------- ---------- ---------- ---------- ---------- ---------- ---------- | - |
| XP_024583883.1 | ---------- ---------- ---------- ---------- ---------- ---------- ---------- ---------- | - |
| XP_024579844.1 | ---------- ---------- ---------- ---------- ---------- ---------- ---------- ---------- | - |
| XP_024574916.1 | ---------- ---------- ---------- ---------- ---------- ---------- ---------- ---------- | - |
| XP_024574193.1 | ---------- ---------- ---------- ---------- ---------- ---------- ---------- ---------- | - |
| XP_024583155.1 | ---------- ---------- ---------- ---------- ---------- ---------- ---------- ---------- | - |
| XP_024574913.1 | ---------- ---------- ---------- ---------- ---------- ---------- ---------- ---------- | - |
| XP_024578158.1 | ---------- ---------- ---------- ---------- ---------- ---------- ---------- ---------- | - |
| PvCRN22 | ---------- ---------- ---------- ---------- ---------- ---------- ---------- ---------- | - |
| XP_024585001.1 | ---------- ---------- ---------- ---------- ---------- ---------- ---------- ---------- | - |
| XP_024583843.1 | ---------- ---------- ---------- ---------- ---------- ---------- ---------- ---------- | - |
| XP_024578702.1 | ---------- ---------- ---------- ---------- ---------- ---------- ---------- ---------- | - |
| XP_024585805.1 | ---------- ---------- ---------- ---------- ---------- ---------- ---------- ---------- | - |
| XP_024580932.1 | ---------- ---------- ---------- ---------- ---------- ---------- ---------- ---------- | - |
| Consensus | ---------- ---------- ---------- ---------- ---------- ---------- ---------- ---------- | |

Conservation 100% 0%

```
                                   180                 200                 220                 240
                                     |                   |                   |                   |
PvCRN11         ---------- ---------- ---------- ---------- ---------- ---------- ---------- ---------- -
XP_024579260.1  ---------- ---------- ---------- ---------- ---------- ---------- ---------- ---------- -
XP_024573634.1  ---------- ---------- ---------- ---------- ---------- ---------- ---------- ---------- -
PvCRN7          ---------- ---------- ---------- ---------- ---------- ---------- ---------- ---------- -
XP_024585265.1  ---------- ---------- ---------- ---------- ---------- ---------- ---------- ---------- -
PvCRN18         ---------- ---------- ---------- ---------- ---------- ---------- ---------- ---------- -
XP_024578414.1  ---------- ---------- ---------- ---------- ---------- ---------- ---------- ---------- 2
XP_024579130.1  ---------- ---------- ---------- ---------- ---------- ---------- ---------- ---------- 5
XP_024584758.1  ---------- ---------- ---------- ---------- ---------- ---------- ---------- ---------- -
XP_024586066.1  ---------- ---------- ---------- ---------- ---------- ---------- ---------- ---------- 11
XP_024585870.1  ---------- ---------- ---------- ---------- ---------- ---------- ---------- ---------- -
PvCRN31         ---------- ---------- ---------- ---------- ---------- ---------- ---------- ---------- -
XP_024577278.1  ---------- ---------- ---------- ---------- ---------- ---------- ---------- ---------- 2
XP_024585562.1  ---------- ---------- ---------- ---------- ---------- ---------- ---------- ---------- 1
XP_024573944.1  ---------- ---------- ---------- ---------- ---------- ---------- ---------- ---------- -
XP_024579793.1  ---------- ---------- ---------- ---------- ---------- ---------- ---------- ---------- -
PvCRN15         ---------- ---------- ---------- ---------- ---------- ---------- ---------- ---------- -
PvCRN26         ---------- ---------- ---------- ---------- ---------- ---------- ---------- ---------- -
PvCRN16         ---------- ---------- ---------- ---------- ---------- ---------- ---------- ---------- -
PvCRN23         ---------- ---------- ---------- ---------- ---------- ---------- ---------- ---------- -
XP_024573063.1  ---------- ---------- ---------- ---------- ---------- ---------- ---------- ---------- -
PvCRN19         ---------- ---------- ---------- ---------- ---------- ---------- ---------- ---------- -
XP_024581625.1  ---------- ---------- ---------- ---------- ---------- ---------- ---------- ---------- -
XP_024583886.1  ---------- ---------- ---------- ---------- ---------- ---------- ---------- ---------- -
XP_024584409.1  ---------- ---------- ---------- ---------- ---------- ---------- ---------- ---------- -
XP_024572385.1  ---------- ---------- ---------- ---------- ---------- ---------- ---------- ---------- -
PvCRN6          ---------- ---------- ---------- ---------- ---------- ---------- ---------- ---------- -
PvCRN9          ---------- ---------- ---------- ---------- ---------- ---------- ---------- ---------- -
XP_024580875.1  ---------- ---------- ---------- ---------- ---------- ---------- ---------- ---------- -
PvCRN24         ---------- ---------- ---------- ---------- ---------- ---------- ---------- ---------- -
PvCRN35         ---------- ---------- ---------- ---------- ---------- ---------- ---------- ---------- -
PvCRN12         ---------- ---------- ---------- ---------- ---------- ---------- ---------- ---------- -
XP_024576927.1  ---------- ---------- ---------- ---------- ---------- ---------- ---------- ---------- -
XP_024578927.1  ---------- ---------- ---------- ---------- ---------- ---------- ---------- ---------- -
XP_024586212.1  ---------- ---------- ---------- ---------- ---------- ---------- ---------- ---------- -
XP_024578078.1  ---------- ---------- ---------- ---------- ---------- ---------- ---------- ---------- -
XP_024575355.1  ---------- ---------- ---------- ---------- ---------- ---------- ---------- ---------- -
XP_024584527.1  ---------- ---------- ---------- ---------- ---------- ---------- ---------- ---------- -
XP_024586664.1  ---------- ---------- ---------- ---------- ---------- ---------- ---------- ---------- -
PvCRN17         ---------- ---------- ---------- ---------- ---------- ---------- ---------- ---------- -
PvCRN2          ---------- ---------- ---------- ---------- ---------- ---------- ---------- ---------- -
XP_024577181.1  ---------- ---------- ---------- ---------- ---------- ---------- ---------- ---------- -
XP_024581075.1  ---------- ---------- ---------- ---------- ---------- ---------- ---------- ---------- -
XP_024575884.1  ---------- ---------- ---------- ---------- ---------- ---------- ---------- ---------- -
XP_024586872.1  ---------- ---------- ---------- ---------- ---------- ---------- ---------- ---------- -
XP_024573052.1  ---------- ---------- ---------- ---------- ---------- ---------- ---------- ---------- -
XP_024586054.1  ---------- ---------- ---------- ---------- ---------- ---------- ---------- ---------- -
PvCRN27         ---------- ---------- ---------- ---------- ---------- ---------- ---------- ---------- -
PvCRN20         ---------- ---------- ---------- ---------- ---------- ---------- ---------- ---------- -
PvCRN25         ---------- ---------- ---------- ---------- ---------- ---------- ---------- ---------- -
PvCRN1          ---------- ---------- ---------- ---------- ---------- ---------- ---------- ---------- -
PvCRN4          ---------- ---------- ---------- ---------- ---------- ---------- ---------- ---------- -
PvCRN30         ---------- ---------- ---------- ---------- ---------- ---------- ---------- ---------- -
PvCRN29         ---------- ---------- ---------- ---------- ---------- ---------- ---------- ---------- -
XP_024583036.1  ---------- ---------- ---------- ---------- ---------- ---------- ---------- ---------- -
PvCRN10         ---------- ---------- ---------- ---------- ---------- ---------- ---------- ---------- -
XP_024577280.1  ---------- ---------- ---------- ---------- ---------- ---------- ---------- ---------- 1
XP_024572924.1  ---------- ---------- ---------- ---------- ---------- ---------- ---------- ---------- 2
PvCRN14         ---------- ---------- ---------- ---------- ---------- ---------- ---------- ---------- -
XP_024577521.1  ---------- ---------- ---------- ---------- ---------- ---------- ---------- ---------- -
PvCRN21         ---------- ---------- ---------- ---------- ---------- ---------- ---------- ---------- -
XP_024574966.1  ---------- ---------- ---------- ---------- ---------- ---------- ---------- ---------- -
XP_024575372.1  ---------- ---------- ---------- ---------- ---------- ---------- ---------- ---------- -
XP_024581363.1  ---------- ---------- ---------- ---------- ---------- ---------- ---------- ---------- -
XP_024583883.1  ---------- ---------- ---------- ---------- ---------- ---------- ---------- ---------- -
XP_024579844.1  ---------- ---------- ---------- ---------- ---------- ---------- ---------- ---------- -
XP_024574916.1  ---------- ---------- ---------- ---------- ---------- ---------- ---------- ---------- -
XP_024574193.1  ---------- ---------- ---------- ---------- ---------- ---------- ---------- ---------- -
XP_024583155.1  ---------- ---------- ---------- ---------- ---------- ---------- ---------- ---------- -
XP_024574913.1  ---------- ---------- ---------- ---------- ---------- ---------- ---------- ---------- -
XP_024578158.1  ---------- ---------- ---------- ---------- ---------- ---------- ---------- ---------- -
PvCRN22         ---------- ---------- ---------- ---------- ---------- ---------- ---------- ---------- -
XP_024585001.1  ---------- ---------- ---------- ---------- ---------- ---------- ---------- ---------- -
XP_024583843.1  ---------- ---------- ---------- ---------- ---------- ---------- ---------- ---------- -
XP_024578702.1  ---------- ---------- ---------- ---------- ---------- ---------- ---------- ---------- -
XP_024585805.1  ---------- ---------- ---------- ---------- ---------- ---------- ---------- ---------- -
XP_024580932.1  ---------- ---------- ---------- ---------- ---------- ---------- ---------- ---------- -
Consensus       ---------- ---------- ---------- ---------- ---------- ---------- ---------- ----------
           100%
Conservation
             0%
```

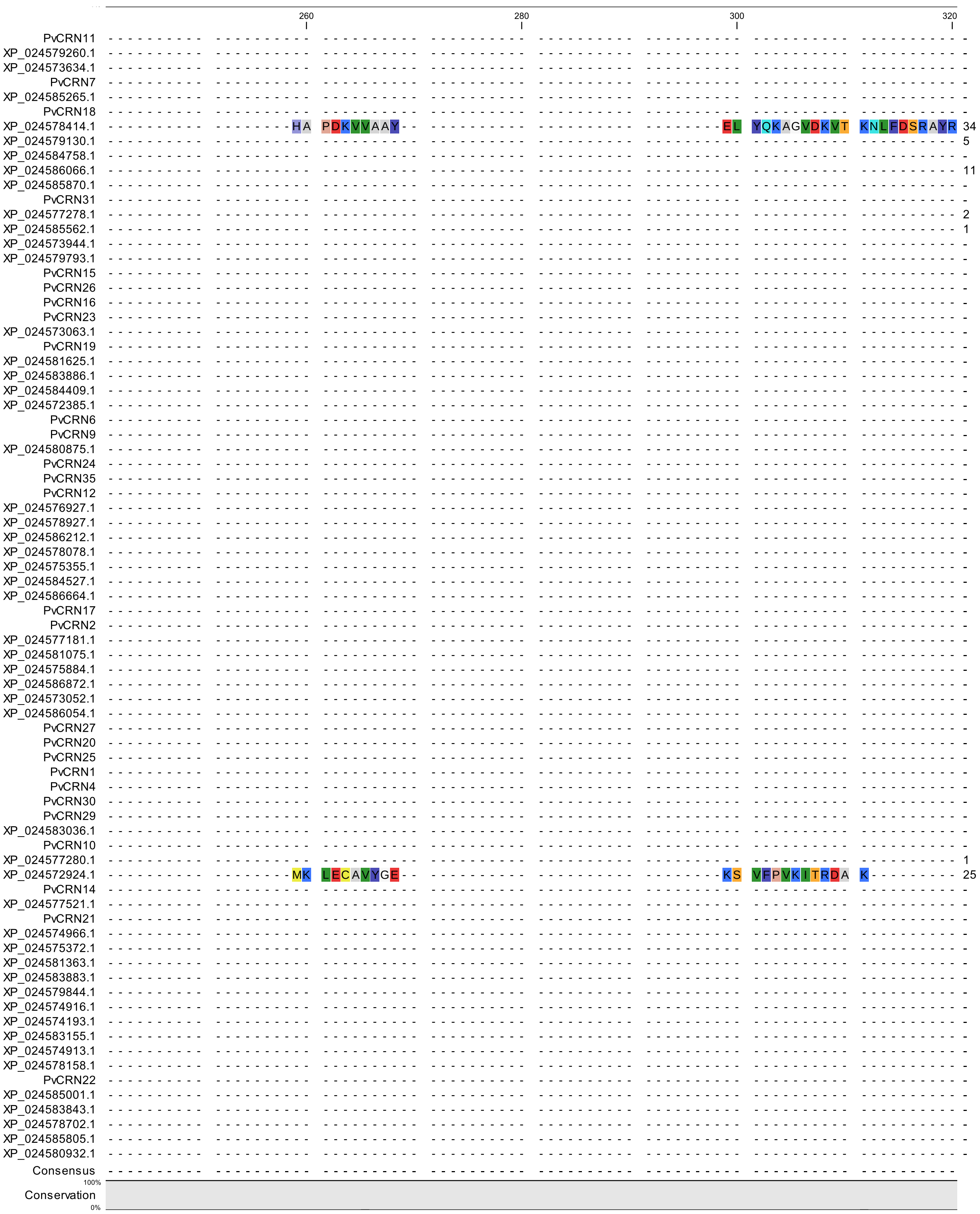
260
280
300
320
PvCRN11
XP_024579260.1
XP_024573634.1
PvCRN7
XP_024585265.1
PvCRN18
XP_024578414.1 HA PDKVVAAY EL YQKAGVDKVT KNLFDSRAYR 34
XP_024579130.1 5
XP_024584758.1
XP_024586066.1 11
XP_024585870.1
PvCRN31
XP_024577278.1 2
XP_024585562.1 1
XP_024573944.1
XP_024579793.1
PvCRN15
PvCRN26
PvCRN16
PvCRN23
XP_024573063.1
PvCRN19
XP_024581625.1
XP_024583886.1
XP_024584409.1
XP_024572385.1
PvCRN6
PvCRN9
XP_024580875.1
PvCRN24
PvCRN35
PvCRN12
XP_024576927.1
XP_024578927.1
XP_024586212.1
XP_024578078.1
XP_024575355.1
XP_024584527.1
XP_024586664.1
PvCRN17
PvCRN2
XP_024577181.1
XP_024581075.1
XP_024575884.1
XP_024586872.1
XP_024573052.1
XP_024586054.1
PvCRN27
PvCRN20
PvCRN25
PvCRN1
PvCRN4
PvCRN30
PvCRN29
XP_024583036.1
PvCRN10
XP_024577280.1 1
XP_024572924.1 MK LECAVYGE KS VFPVKITRDA K 25
PvCRN14
XP_024577521.1
PvCRN21
XP_024574966.1
XP_024575372.1
XP_024581363.1
XP_024583883.1
XP_024579844.1
XP_024574916.1
XP_024574193.1
XP_024583155.1
XP_024574913.1
XP_024578158.1
PvCRN22
XP_024585001.1
XP_024583843.1
XP_024578702.1
XP_024585805.1
XP_024580932.1
Consensus
100%
Conservation
0%

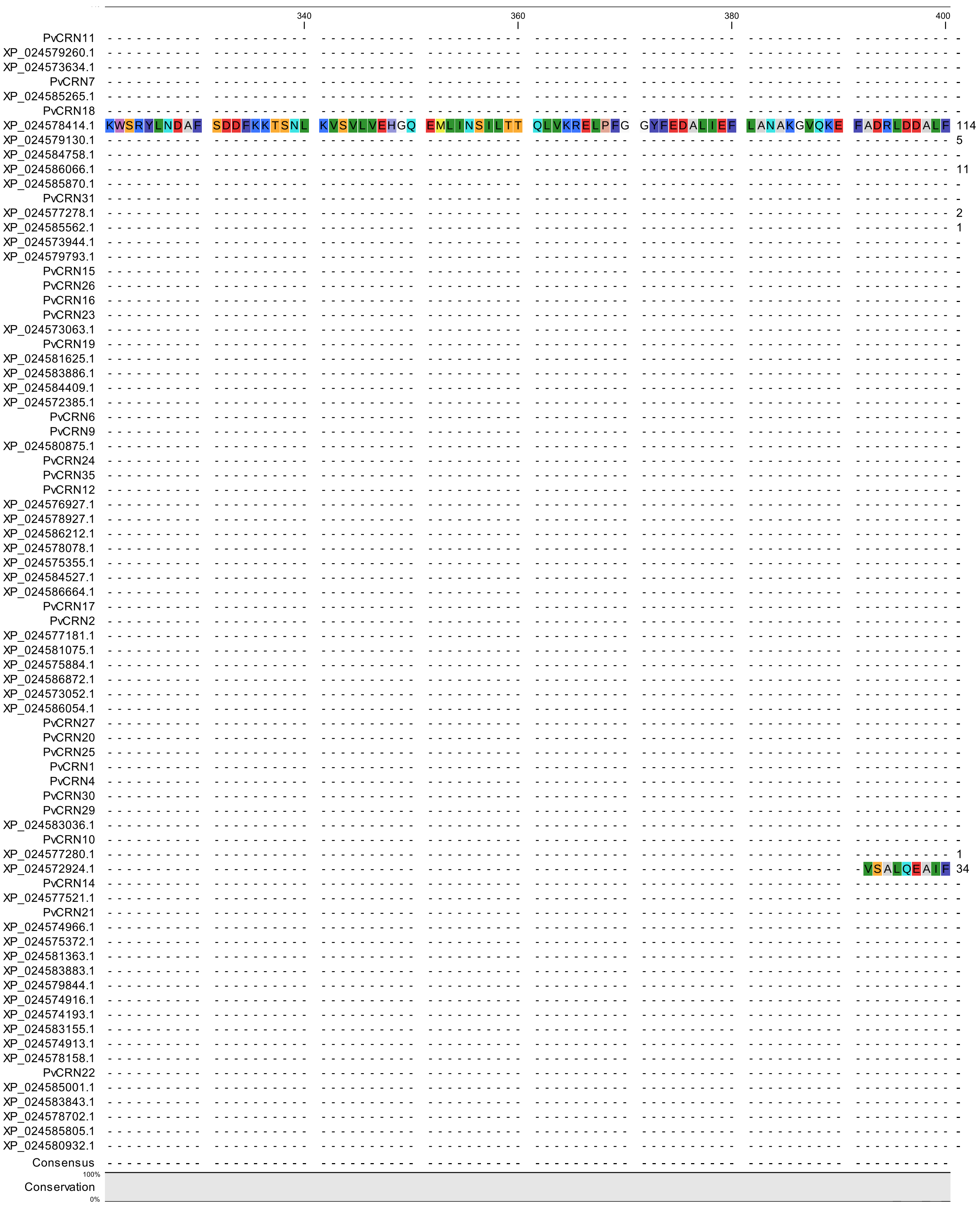
340
360
380
400
PvCRN11
XP_024579260.1
XP_024573634.1
PvCRN7
XP_024585265.1
PvCRN18
XP_024578414.1 KWSRYLNDAF SDDFKKTSNL KVSVLVEHGQ EMLINSILTT QLVKRELPFG GYFEDALIEF LANAKGVQKE FADRLDDALF 114
XP_024579130.1 5
XP_024584758.1
XP_024586066.1 11
XP_024585870.1
PvCRN31
XP_024577278.1 2
XP_024585562.1 1
XP_024573944.1
XP_024579793.1
PvCRN15
PvCRN26
PvCRN16
PvCRN23
XP_024573063.1
PvCRN19
XP_024581625.1
XP_024583886.1
XP_024584409.1
XP_024572385.1
PvCRN6
PvCRN9
XP_024580875.1
PvCRN24
PvCRN35
PvCRN12
XP_024576927.1
XP_024578927.1
XP_024586212.1
XP_024578078.1
XP_024575355.1
XP_024584527.1
XP_024586664.1
PvCRN17
PvCRN2
XP_024577181.1
XP_024581075.1
XP_024575884.1
XP_024586872.1
XP_024573052.1
XP_024586054.1
PvCRN27
PvCRN20
PvCRN25
PvCRN1
PvCRN4
PvCRN30
PvCRN29
XP_024583036.1
PvCRN10
XP_024577280.1 1
XP_024572924.1 VSALQEAIF 34
PvCRN14
XP_024577521.1
PvCRN21
XP_024574966.1
XP_024575372.1
XP_024581363.1
XP_024583883.1
XP_024579844.1
XP_024574916.1
XP_024574193.1
XP_024583155.1
XP_024574913.1
XP_024578158.1
PvCRN22
XP_024585001.1
XP_024583843.1
XP_024578702.1
XP_024585805.1
XP_024580932.1
Consensus
100%
Conservation
0%

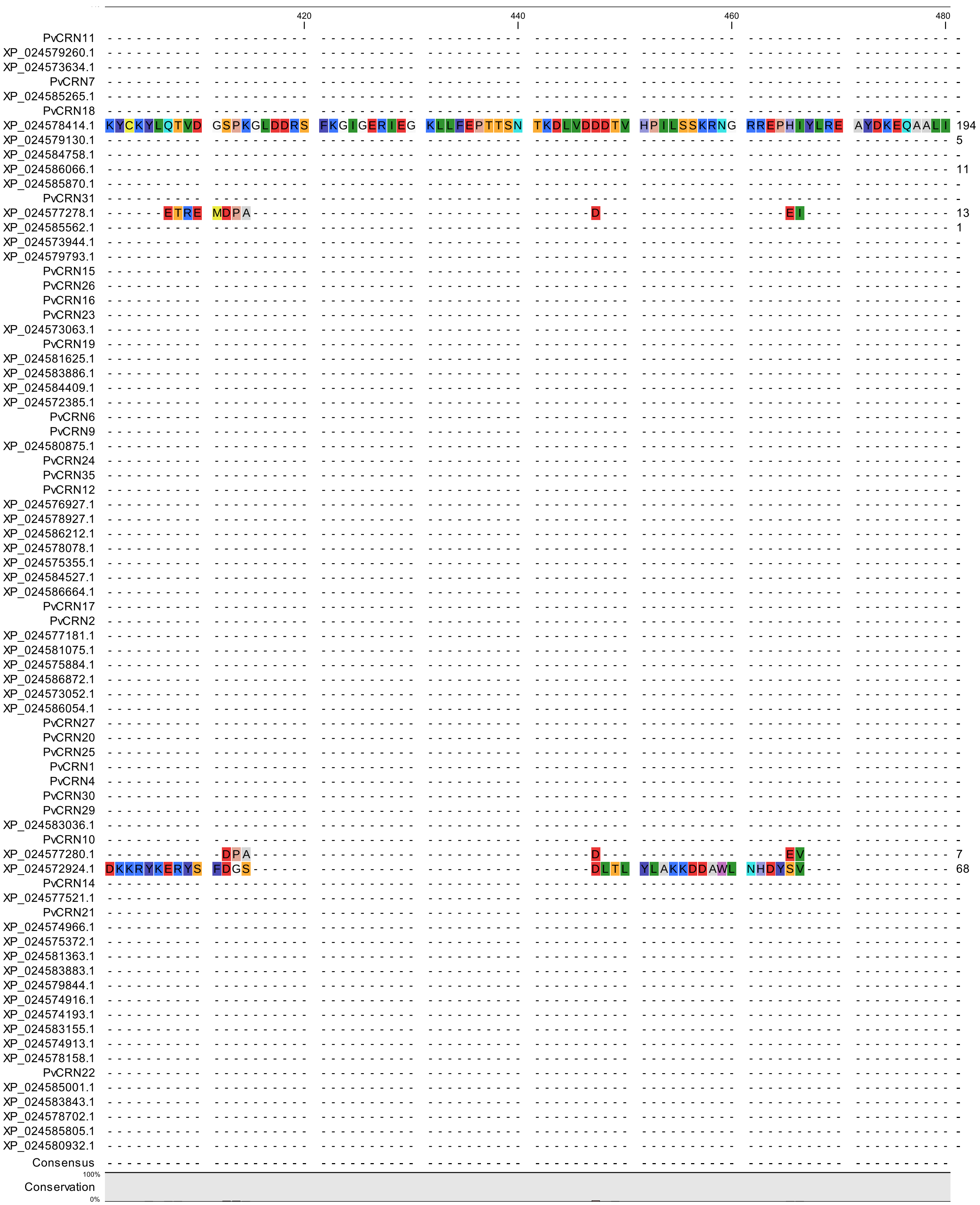
420
440
460
480
PvCRN11
XP_024579260.1
XP_024573634.1
PvCRN7
XP_024585265.1
PvCRN18
XP_024578414.1 KYCKYLQTVD GSPKGLDDRS FKGIGERIEG KLLFEPTTSN TKDLVDDDTV HPILSSKRNG RREPHIYLRE AYDKEQAALI 194
XP_024579130.1 5
XP_024584758.1
XP_024586066.1 11
XP_024585870.1
PvCRN31
XP_024577278.1 ETRE MDPA D EI 13
XP_024585562.1 1
XP_024573944.1
XP_024579793.1
PvCRN15
PvCRN26
PvCRN16
PvCRN23
XP_024573063.1
PvCRN19
XP_024581625.1
XP_024583886.1
XP_024584409.1
XP_024572385.1
PvCRN6
PvCRN9
XP_024580875.1
PvCRN24
PvCRN35
PvCRN12
XP_024576927.1
XP_024578927.1
XP_024586212.1
XP_024578078.1
XP_024575355.1
XP_024584527.1
XP_024586664.1
PvCRN17
PvCRN2
XP_024577181.1
XP_024581075.1
XP_024575884.1
XP_024586872.1
XP_024573052.1
XP_024586054.1
PvCRN27
PvCRN20
PvCRN25
PvCRN1
PvCRN4
PvCRN30
PvCRN29
XP_024583036.1
PvCRN10
XP_024577280.1 DPA D EV 7
XP_024572924.1 DKKRYKERYS FDGS DLTL YLAKKDDAWL NHDYSV 68
PvCRN14
XP_024577521.1
PvCRN21
XP_024574966.1
XP_024575372.1
XP_024581363.1
XP_024583883.1
XP_024579844.1
XP_024574916.1
XP_024574193.1
XP_024583155.1
XP_024574913.1
XP_024578158.1
PvCRN22
XP_024585001.1
XP_024583843.1
XP_024578702.1
XP_024585805.1
XP_024580932.1
Consensus
100%
Conservation
0%

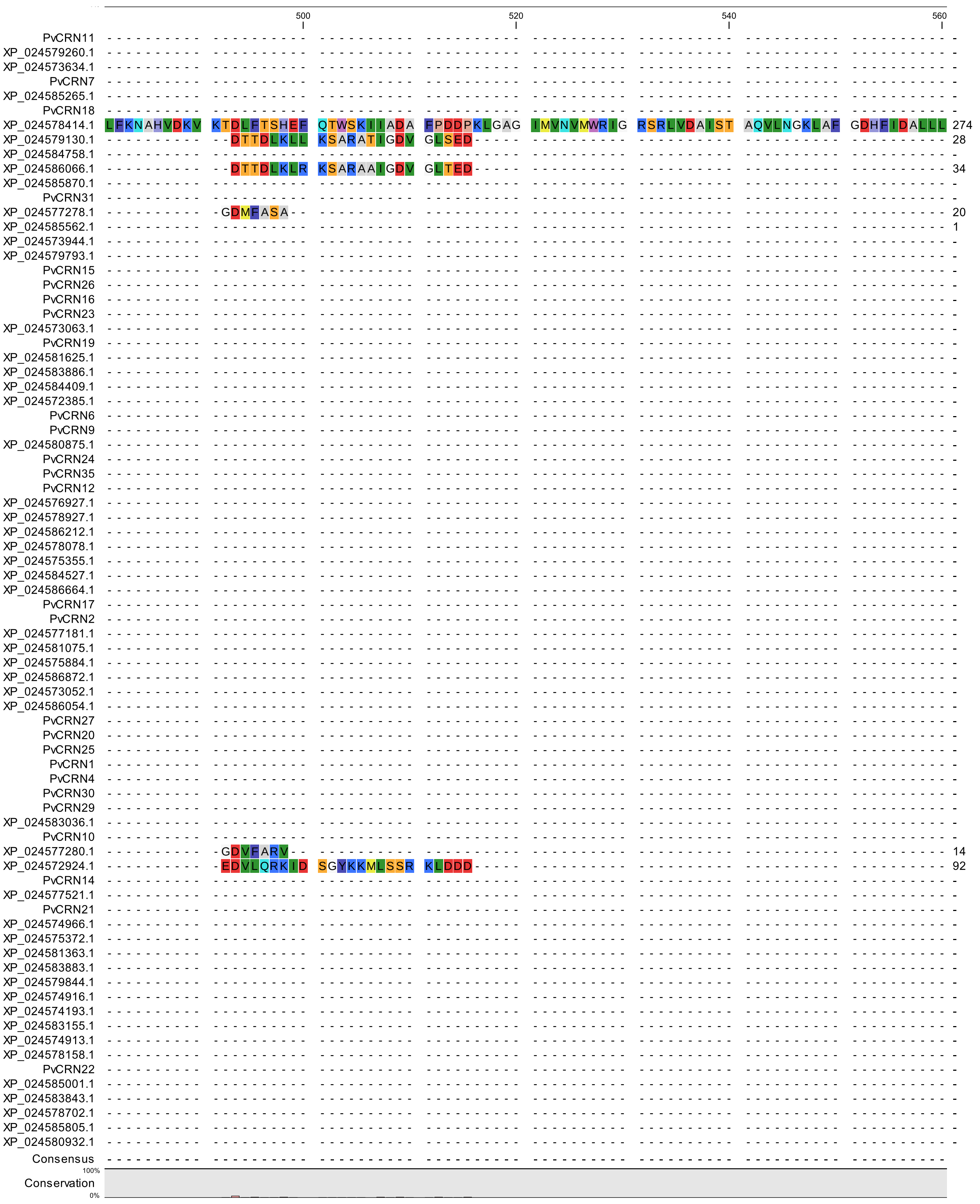

500
520
540
560
XP_024578414.1 LFKNAHVDKV KTDLFTSHEF QTWSKIIADA FPDDPKLGAG IMVNVMWRIG RSRLVDAIST AQVLNGKLAF GDHFIDALLL 274
XP_024579130.1 DTTDLKLL KSARATIGDV GLSED 28
XP_024586066.1 DTTDLKLR KSARAAIGDV GLTED 34
XP_024577278.1 GDMFASA 20
XP_024585562.1 1
XP_024577280.1 GDVFARV 14
XP_024572924.1 EDVLQRKID SGYKKMLSSR KLDDD 92
Consensus
Conservation
100%
0%

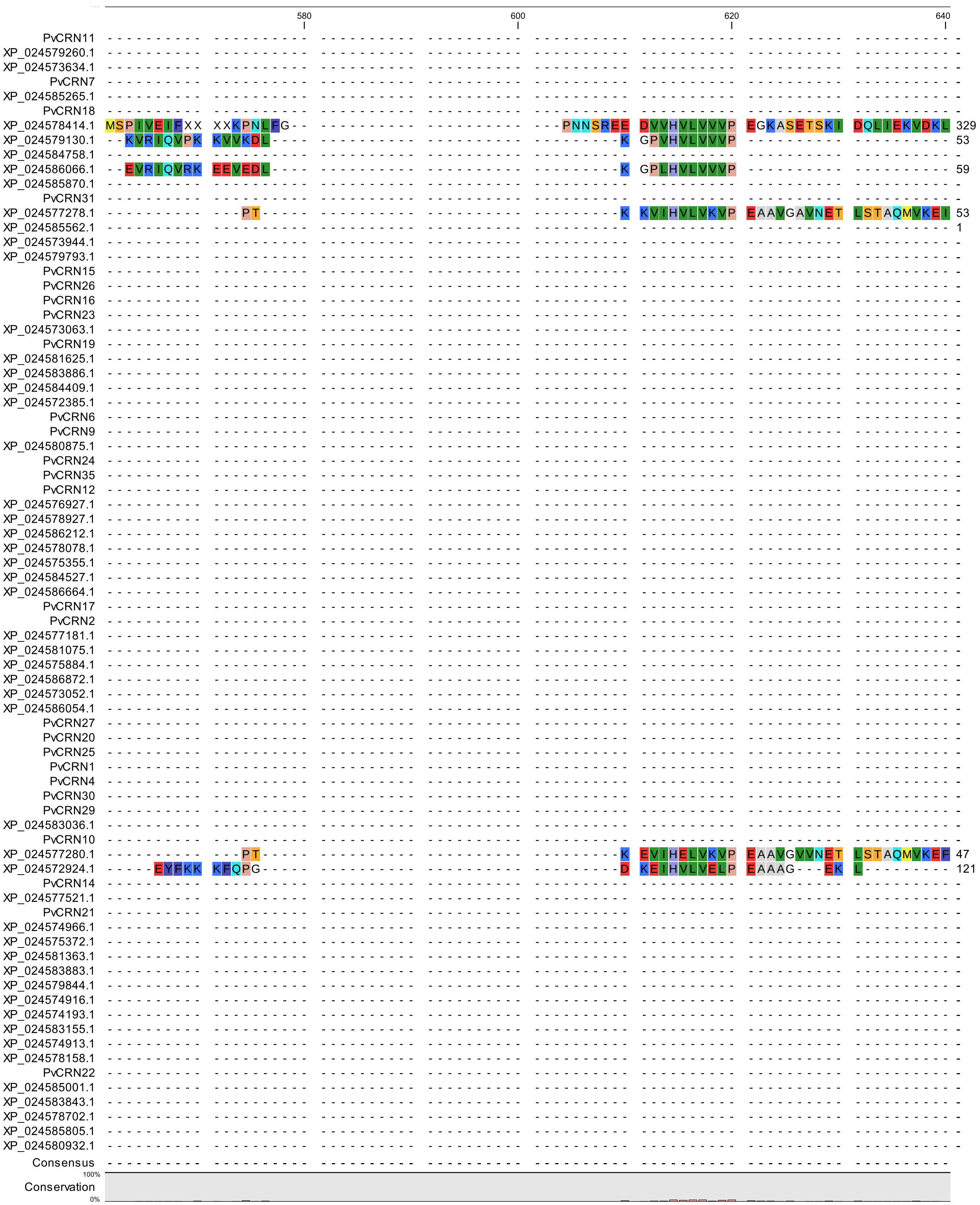

580 600 620 640
XP_024578414.1 MSPIVEIFXX XXKPNLFG PNNSREE DVVHVLVVVP EGKASETSKI DQLIEKVDKL 329
XP_024579130.1 KVRIQVPK KVVKDL K GPVHVLVVVP 53
XP_024586066.1 EVRIQVRK EEVEDL K GPLHVLVVVP 59
XP_024577278.1 PT K KVIHVLVKVP EAAVGAVNET LSTAQMVKEI 53
XP_024585562.1 1
XP_024577280.1 PT K EVIHELVKVP EAAVGVVNET LSTAQMVKEF 47
XP_024572924.1 EYFKK KFQPG D KEIHVLVELP EAAAG EK L 121
Consensus
Conservation
100%
0%

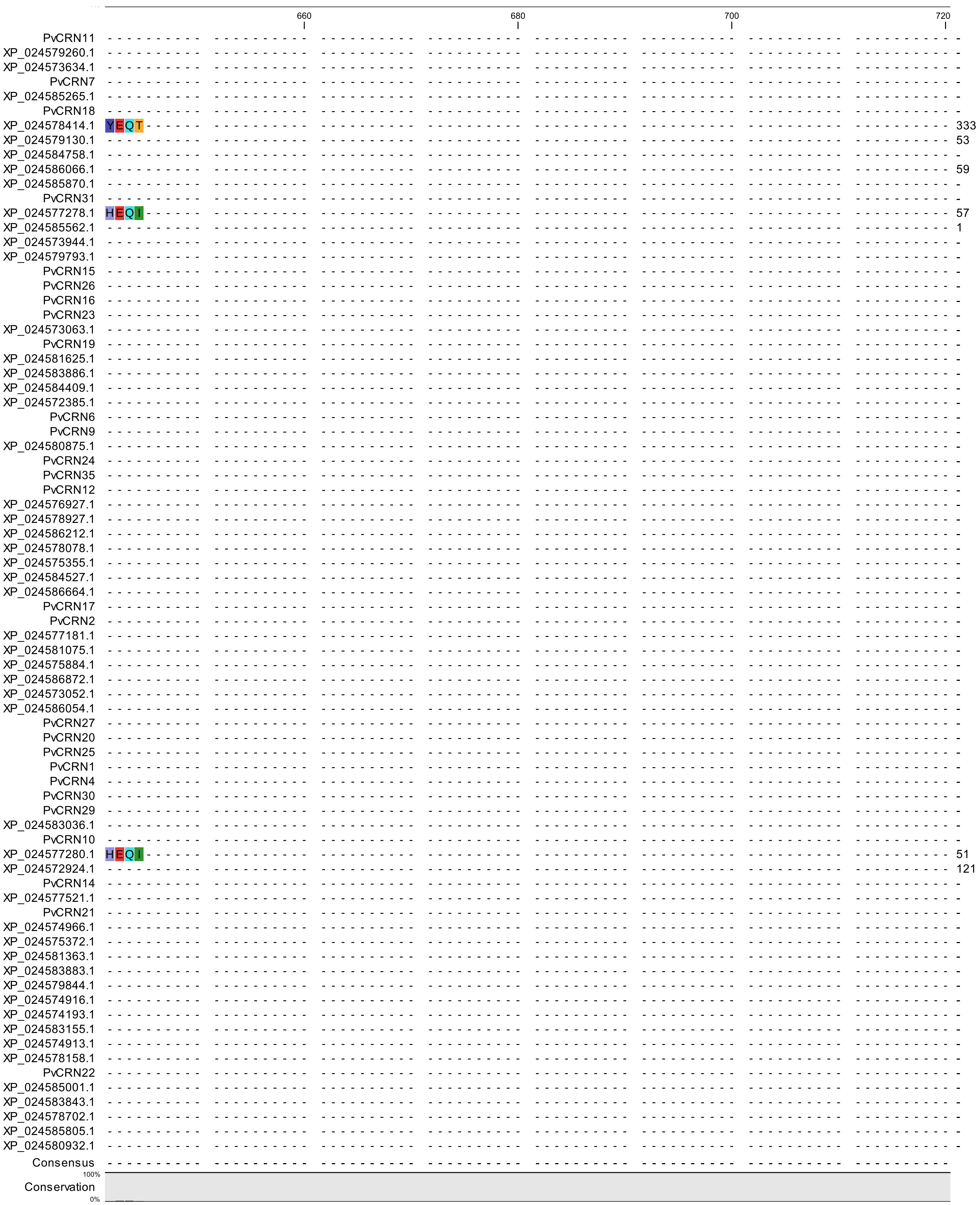
660
680
700
720
PvCRN11
XP_024579260.1
XP_024573634.1
PvCRN7
XP_024585265.1
PvCRN18
XP_024578414.1 YEQT 333
XP_024579130.1 53
XP_024584758.1
XP_024586066.1 59
XP_024585870.1
PvCRN31
XP_024577278.1 HEQI 57
XP_024585562.1 1
XP_024573944.1
XP_024579793.1
PvCRN15
PvCRN26
PvCRN16
PvCRN23
XP_024573063.1
PvCRN19
XP_024581625.1
XP_024583886.1
XP_024584409.1
XP_024572385.1
PvCRN6
PvCRN9
XP_024580875.1
PvCRN24
PvCRN35
PvCRN12
XP_024576927.1
XP_024578927.1
XP_024586212.1
XP_024578078.1
XP_024575355.1
XP_024584527.1
XP_024586664.1
PvCRN17
PvCRN2
XP_024577181.1
XP_024581075.1
XP_024575884.1
XP_024586872.1
XP_024573052.1
XP_024586054.1
PvCRN27
PvCRN20
PvCRN25
PvCRN1
PvCRN4
PvCRN30
PvCRN29
XP_024583036.1
PvCRN10
XP_024577280.1 HEQI 51
XP_024572924.1 121
PvCRN14
XP_024577521.1
PvCRN21
XP_024574966.1
XP_024575372.1
XP_024581363.1
XP_024583883.1
XP_024579844.1
XP_024574916.1
XP_024574193.1
XP_024583155.1
XP_024574913.1
XP_024578158.1
PvCRN22
XP_024585001.1
XP_024583843.1
XP_024578702.1
XP_024585805.1
XP_024580932.1
Consensus
100%
Conservation
0%

```
                                                     740                            760                            780                            800
PvCRN11         ---------- ---------- ---------- ---------- ---------- ---------- ---------- ---------- -
XP_024579260.1  ---------- ---------- ---------- ---------- ---------- ---------- ---------- ---------- -
XP_024573634.1  ---------- ---------- ---------- ---------- ---------- ---------- ---------- ---------- -
PvCRN7          ---------- ---------- ---------- ---------- ---------- ---------- ---------- ---------- -
XP_024585265.1  ---------- ---------- ---------- ---------- ---------- ---------- ---------- ---------- -
PvCRN18         ---------- ---------- ---------- ---------- ---------- ---------- ---------- ---------- -
XP_024578414.1  ---------- ---------- ---------- ---------- ---------- ---------- ---------- ---------- 333
XP_024579130.1  ---------- ---------- ---------- ---------- ---------- ---------- ---------- ---------- 53
XP_024584758.1  ---------- ---------- ---------- ---------- ---------- ---------- ---------- ---------- -
XP_024586066.1  ---------- ---------- ---------- ---------- ---------- ---------- ---------- ---------- 59
XP_024585870.1  ---------- ---------- ---------- ---------- ---------- ---------- ---------- ---------- -
PvCRN31         ---------- ---------- ---------- ---------- ---------- ---------- ---------- ---------- -
XP_024577278.1  ---------- ---------- ---------- ---------- ---------- ---------- ---------- ---------- 57
XP_024585562.1  ---------- ---------- ---------- ---------- ---------- ---------- ---------- ---------- 1
XP_024573944.1  ---------- ---------- ---------- ---------- ---------- ---------- ---------- ---------- -
XP_024579793.1  ---------- ---------- ---------- ---------- ---------- ---------- ---------- ---------- -
PvCRN15         ---------- ---------- ---------- ---------- ---------- ---------- ---------- ---------- -
PvCRN26         ---------- ---------- ---------- ---------- ---------- ---------- ---------- ---------- -
PvCRN16         ---------- ---------- ---------- ---------- ---------- ---------- ---------- ---------- -
PvCRN23         ---------- ---------- ---------- ---------- ---------- ---------- ---------- ---------- -
XP_024573063.1  ---------- ---------- ---------- ---------- ---------- ---------- ---------- ---------- -
PvCRN19         ---------- ---------- ---------- ---------- ---------- ---------- ---------- ---------- -
XP_024581625.1  ---------- ---------- ---------- ---------- ---------- ---------- ---------- ---------- -
XP_024583886.1  ---------- ---------- ---------- ---------- ---------- ---------- ---------- ---------- -
XP_024584409.1  ---------- ---------- ---------- ---------- ---------- ---------- ---------- ---------- -
XP_024572385.1  ---------- ---------- ---------- ---------- ---------- ---------- ---------- ---------- -
PvCRN6          ---------- ---------- ---------- ---------- ---------- ---------- ---------- ---------- -
PvCRN9          ---------- ---------- ---------- ---------- ---------- ---------- ---------- ---------- -
XP_024580875.1  ---------- ---------- ---------- ---------- ---------- ---------- ---------- ---------- -
PvCRN24         ---------- ---------- ---------- ---------- ---------- ---------- ---------- ---------- -
PvCRN35         ---------- ---------- ---------- ---------- ---------- ---------- ---------- ---------- -
PvCRN12         ---------- ---------- ---------- ---------- ---------- ---------- ---------- ---------- -
XP_024576927.1  ---------- ---------- ---------- ---------- ---------- ---------- ---------- ---------- -
XP_024578927.1  ---------- ---------- ---------- ---------- ---------- ---------- ---------- ---------- -
XP_024586212.1  ---------- ---------- ---------- ---------- ---------- ---------- ---------- ---------- -
XP_024578078.1  ---------- ---------- ---------- ---------- ---------- ---------- ---------- ---------- -
XP_024575355.1  ---------- ---------- ---------- ---------- ---------- ---------- ---------- ---------- -
XP_024584527.1  ---------- ---------- ---------- ---------- ---------- ---------- ---------- ---------- -
XP_024586664.1  ---------- ---------- ---------- ---------- ---------- ---------- ---------- ---------- -
PvCRN17         ---------- ---------- ---------- ---------- ---------- ---------- ---------- ---------- -
PvCRN2          ---------- ---------- ---------- ---------- ---------- ---------- ---------- ---------- -
XP_024577181.1  ---------- ---------- ---------- ---------- ---------- ---------- ---------- ---------- -
XP_024581075.1  ---------- ---------- ---------- ---------- ---------- ---------- ---------- ---------- -
XP_024575884.1  ---------- ---------- ---------- ---------- ---------- ---------- ---------- ---------- -
XP_024586872.1  ---------- ---------- ---------- ---------- ---------- ---------- ---------- ---------- -
XP_024573052.1  ---------- ---------- ---------- ---------- ---------- ---------- ---------- ---------- -
XP_024586054.1  ---------- ---------- ---------- ---------- ---------- ---------- ---------- ---------- -
PvCRN27         ---------- ---------- ---------- ---------- ---------- ---------- ---------- ---------- -
PvCRN20         ---------- ---------- ---------- ---------- ---------- ---------- ---------- ---------- -
PvCRN25         ---------- ---------- ---------- ---------- ---------- ---------- ---------- ---------- -
PvCRN1          ---------- ---------- ---------- ---------- ---------- ---------- ---------- ---------- -
PvCRN4          ---------- ---------- ---------- ---------- ---------- ---------- ---------- ---------- -
PvCRN30         ---------- ---------- ---------- ---------- ---------- ---------- ---------- ---------- -
PvCRN29         ---------- ---------- ---------- ---------- ---------- ---------- ---------- ---------- -
XP_024583036.1  ---------- ---------- ---------- ---------- ---------- ---------- ---------- ---------- -
PvCRN10         ---------- ---------- ---------- ---------- ---------- ---------- ---------- ---------- -
XP_024577280.1  ---------- ---------- ---------- ---------- ---------- ---------- ---------- ---------- 51
XP_024572924.1  ---------- ---------- ---------- ---------- ---------- ---------- ---------- ---------- 121
PvCRN14         ---------- ---------- ---------- ---------- ---------- ---------- ---------- ---------- -
XP_024577521.1  ---------- ---------- ---------- ---------- ---------- ---------- ---------- ---------- -
PvCRN21         ---------- ---------- ---------- ---------- ---------- ---------- ---------- ---------- -
XP_024574966.1  ---------- ---------- ---------- ---------- ---------- ---------- ---------- ---------- -
XP_024575372.1  ---------- ---------- ---------- ---------- ---------- ---------- ---------- ---------- -
XP_024581363.1  ---------- ---------- ---------- ---------- ---------- ---------- ---------- ---------- -
XP_024583883.1  ---------- ---------- ---------- ---------- ---------- ---------- ---------- ---------- -
XP_024579844.1  ---------- ---------- ---------- ---------- ---------- ---------- ---------- ---------- -
XP_024574916.1  ---------- ---------- ---------- ---------- ---------- ---------- ---------- ---------- -
XP_024574193.1  ---------- ---------- ---------- ---------- ---------- ---------- ---------- ---------- -
XP_024583155.1  ---------- ---------- ---------- ---------- ---------- ---------- ---------- ---------- -
XP_024574913.1  ---------- ---------- ---------- ---------- ---------- ---------- ---------- ---------- -
XP_024578158.1  ---------- ---------- ---------- ---------- ---------- ---------- ---------- ---------- -
PvCRN22         ---------- ---------- ---------- ---------- ---------- ---------- ---------- ---------- -
XP_024585001.1  ---------- ---------- ---------- ---------- ---------- ---------- ---------- ---------- -
XP_024583843.1  ---------- ---------- ---------- ---------- ---------- ---------- ---------- ---------- -
XP_024578702.1  ---------- ---------- ---------- ---------- ---------- ---------- ---------- ---------- -
XP_024585805.1  ---------- ---------- ---------- ---------- ---------- ---------- ---------- ---------- -
XP_024580932.1  ---------- ---------- ---------- ---------- ---------- ---------- ---------- ---------- -
Consensus       ---------- ---------- ---------- ---------- ---------- ---------- ---------- ----------
            100%
Conservation
              0%
```

```
                          820         840         860         880
                            |           |           |           |
PvCRN11         ---------- ---------- ---------- ---------- ---------- ---------- ---------- ---------- -
XP_024579260.1  ---------- ---------- ---------- ---------- ---------- ---------- ---------- ---------- -
XP_024573634.1  ---------- ---------- ---------- ---------- ---------- ---------- ---------- ---------- -
PvCRN7          ---------- ---------- ---------- ---------- ---------- ---------- ---------- ---------- -
XP_024585265.1  ---------- ---------- ---------- ---------- ---------- ---------- ---------- ---------- -
PvCRN18         ---------- ---------- ---------- ---------- ---------- ---------- ---------- ---------- -
XP_024578414.1  ---------- ---------- ---------- ---------- ---------- ---------- ---------- ---------- 333
XP_024579130.1  ---------- ---------- ---------- ---------- ---------- ---------- ---------- ---------- 53
XP_024584758.1  ---------- ---------- ---------- ---------- ---------- ---------- ---------- ---------- -
XP_024586066.1  ---------- ---------- ---------- ---------- ---------- ---------- ---------- ---------- 59
XP_024585870.1  ---------- ---------- ---------- ---------- ---------- ---------- ---------- ---------- -
PvCRN31         ---------- ---------- ---------- ---------- ---------- ---------- ---------- ---------- -
XP_024577278.1  ---------- ---------- ---------- ---------- ---------- ---------- ---------- ---------- 57
XP_024585562.1  ---------- ---------- ---------- ---------- ---------- ---------- ---------- ---------- 1
XP_024573944.1  ---------- ---------- ---------- ---------- ---------- ---------- ---------- ---------- -
XP_024579793.1  ---------- ---------- ---------- ---------- ---------- ---------- ---------- ---------- -
PvCRN15         ---------- ---------- ---------- ---------- ---------- ---------- ---------- ---------- -
PvCRN26         ---------- ---------- ---------- ---------- ---------- ---------- ---------- ---------- -
PvCRN16         ---------- ---------- ---------- ---------- ---------- ---------- ---------- ---------- -
PvCRN23         ---------- ---------- ---------- ---------- ---------- ---------- ---------- ---------- -
XP_024573063.1  ---------- ---------- ---------- ---------- ---------- ---------- ---------- ---------- -
PvCRN19         ---------- ---------- ---------- ---------- ---------- ---------- ---------- ---------- -
XP_024581625.1  ---------- ---------- ---------- ---------- ---------- ---------- ---------- ---------- -
XP_024583886.1  ---------- ---------- ---------- ---------- ---------- ---------- ---------- ---------- -
XP_024584409.1  ---------- ---------- ---------- ---------- ---------- ---------- ---------- ---------- -
XP_024572385.1  ---------- ---------- ---------- ---------- ---------- ---------- ---------- ---------- -
PvCRN6          ---------- ---------- ---------- ---------- ---------- ---------- ---------- ---------- -
PvCRN9          ---------- ---------- ---------- ---------- ---------- ---------- ---------- ---------- -
XP_024580875.1  ---------- ---------- ---------- ---------- ---------- ---------- ---------- ---------- -
PvCRN24         ---------- ---------- ---------- ---------- ---------- ---------- ---------- ---------- -
PvCRN35         ---------- ---------- ---------- ---------- ---------- ---------- ---------- ---------- -
PvCRN12         ---------- ---------- ---------- ---------- ---------- ---------- ---------- ---------- -
XP_024576927.1  ---------- ---------- ---------- ---------- ---------- ---------- ---------- ---------- -
XP_024578927.1  ---------- ---------- ---------- ---------- ---------- ---------- ---------- ---------- -
XP_024586212.1  ---------- ---------- ---------- ---------- ---------- ---------- ---------- ---------- -
XP_024578078.1  ---------- ---------- ---------- ---------- ---------- ---------- ---------- ---------- -
XP_024575355.1  ---------- ---------- ---------- ---------- ---------- ---------- ---------- ---------- -
XP_024584527.1  ---------- ---------- ---------- ---------- ---------- ---------- ---------- ---------- -
XP_024586664.1  ---------- ---------- ---------- ---------- ---------- ---------- ---------- ---------- -
PvCRN17         ---------- ---------- ---------- ---------- ---------- ---------- ---------- ---------- -
PvCRN2          ---------- ---------- ---------- ---------- ---------- ---------- ---------- ---------- -
XP_024577181.1  ---------- ---------- ---------- ---------- ---------- ---------- ---------- ---------- -
XP_024581075.1  ---------- ---------- ---------- ---------- ---------- ---------- ---------- ---------- -
XP_024575884.1  ---------- ---------- ---------- ---------- ---------- ---------- ---------- ---------- -
XP_024586872.1  ---------- ---------- ---------- ---------- ---------- ---------- ---------- ---------- -
XP_024573052.1  ---------- ---------- ---------- ---------- ---------- ---------- ---------- ---------- -
XP_024586054.1  ---------- ---------- ---------- ---------- ---------- ---------- ---------- ---------- -
PvCRN27         ---------- ---------- ---------- ---------- ---------- ---------- ---------- ---------- -
PvCRN20         ---------- ---------- ---------- ---------- ---------- ---------- ---------- ---------- -
PvCRN25         ---------- ---------- ---------- ---------- ---------- ---------- ---------- ---------- -
PvCRN1          ---------- ---------- ---------- ---------- ---------- ---------- ---------- ---------- -
PvCRN4          ---------- ---------- ---------- ---------- ---------- ---------- ---------- ---------- -
PvCRN30         ---------- ---------- ---------- ---------- ---------- ---------- ---------- ---------- -
PvCRN29         ---------- ---------- ---------- ---------- ---------- ---------- ---------- ---------- -
XP_024583036.1  ---------- ---------- ---------- ---------- ---------- ---------- ---------- ---------- -
PvCRN10         ---------- ---------- ---------- ---------- ---------- ---------- ---------- ---------- -
XP_024577280.1  ---------- ---------- ---------- ---------- ---------- ---------- ---------- ---------- 51
XP_024572924.1  ---------- ---------- ---------- ---------- ---------- ---------- ---------- ---------- 121
PvCRN14         ---------- ---------- ---------- ---------- ---------- ---------- ---------- ---------- -
XP_024577521.1  ---------- ---------- ---------- ---------- ---------- ---------- ---------- ---------- -
PvCRN21         ---------- ---------- ---------- ---------- ---------- ---------- ---------- ---------- -
XP_024574966.1  ---------- ---------- ---------- ---------- ---------- ---------- ---------- ---------- -
XP_024575372.1  ---------- ---------- ---------- ---------- ---------- ---------- ---------- ---------- -
XP_024581363.1  ---------- ---------- ---------- ---------- ---------- ---------- ---------- ---------- -
XP_024583883.1  ---------- ---------- ---------- ---------- ---------- ---------- ---------- ---------- -
XP_024579844.1  ---------- ---------- ---------- ---------- ---------- ---------- ---------- ---------- -
XP_024574916.1  ---------- ---------- ---------- ---------- ---------- ---------- ---------- ---------- -
XP_024574193.1  ---------- ---------- ---------- ---------- ---------- ---------- ---------- ---------- -
XP_024583155.1  ---------- ---------- ---------- ---------- ---------- ---------- ---------- ---------- -
XP_024574913.1  ---------- ---------- ---------- ---------- ---------- ---------- ---------- ---------- -
XP_024578158.1  ---------- ---------- ---------- ---------- ---------- ---------- ---------- ---------- -
PvCRN22         ---------- ---------- ---------- ---------- ---------- ---------- ---------- ---------- -
XP_024585001.1  ---------- ---------- ---------- ---------- ---------- ---------- ---------- ---------- -
XP_024583843.1  ---------- ---------- ---------- ---------- ---------- ---------- ---------- ---------- -
XP_024578702.1  ---------- ---------- ---------- ---------- ---------- ---------- ---------- ---------- -
XP_024585805.1  ---------- ---------- ---------- ---------- ---------- ---------- ---------- ---------- -
XP_024580932.1  ---------- ---------- ---------- ---------- ---------- ---------- ---------- ---------- -
Consensus       ---------- ---------- ---------- ---------- ---------- ---------- ---------- ----------
                100%
Conservation
                0%
```

```
                                           900                 920                 940                 960
                                             |                   |                   |                   |
PvCRN11         ---------- ---------- ---------- ---------- ---------- ---------- ---------- ---------- -
XP_024579260.1  ---------- ---------- ---------- ---------- ---------- ---------- ---------- ---------- -
XP_024573634.1  ---------- ---------- ---------- ---------- ---------- ---------- ---------- ---------- -
PvCRN7          ---------- ---------- ---------- ---------- ---------- ---------- ---------- ---------- -
XP_024585265.1  ---------- ---------- ---------- ---------- ---------- ---------- ---------- ---------- -
PvCRN18         ---------- ---------- ---------- ---------- ---------- ---------- ---------- ---------- -
XP_024578414.1  ---------- ---------- ---------- ---------- ---------- ---------- ---------- ---------- 333
XP_024579130.1  ---------- ---------- ---------- ---------- ---------- ---------- ---------- ---------- 53
XP_024584758.1  ---------- ---------- ---------- ---------- ---------- ---------- ---------- ---------- -
XP_024586066.1  ---------- ---------- ---------- ---------- ---------- ---------- ---------- ---------- 59
XP_024585870.1  ---------- ---------- ---------- ---------- ---------- ---------- ---------- ---------- -
PvCRN31         ---------- ---------- ---------- ---------- ---------- ---------- ---------- ---------- -
XP_024577278.1  ---------- ---------- ---------- ---------- ---------- ---------- ---------- ---------- 57
XP_024585562.1  ---------- ---------- ---------- ---------- ---------- ---------- ---------- ---------- 1
XP_024573944.1  ---------- ---------- ---------- ---------- ---------- ---------- ---------- ---------- -
XP_024579793.1  ---------- ---------- ---------- ---------- ---------- ---------- ---------- ---------- -
PvCRN15         ---------- ---------- ---------- ---------- ---------- ---------- ---------- ---------- -
PvCRN26         ---------- ---------- ---------- ---------- ---------- ---------- ---------- ---------- -
PvCRN16         ---------- ---------- ---------- ---------- ---------- ---------- ---------- ---------- -
PvCRN23         ---------- ---------- ---------- ---------- ---------- ---------- ---------- ---------- -
XP_024573063.1  ---------- ---------- ---------- ---------- ---------- ---------- ---------- ---------- -
PvCRN19         ---------- ---------- ---------- ---------- ---------- ---------- ---------- ---------- -
XP_024581625.1  ---------- ---------- ---------- ---------- ---------- ---------- ---------- ---------- -
XP_024583886.1  ---------- ---------- ---------- ---------- ---------- ---------- ---------- ---------- -
XP_024584409.1  ---------- ---------- ---------- ---------- ---------- ---------- ---------- ---------- -
XP_024572385.1  ---------- ---------- ---------- ---------- ---------- ---------- ---------- ---------- -
PvCRN6          ---------- ---------- ---------- ---------- ---------- ---------- ---------- ---------- -
PvCRN9          ---------- ---------- ---------- ---------- ---------- ---------- ---------- ---------- -
XP_024580875.1  ---------- ---------- ---------- ---------- ---------- ---------- ---------- ---------- -
PvCRN24         ---------- ---------- ---------- ---------- ---------- ---------- ---------- ---------- -
PvCRN35         ---------- ---------- ---------- ---------- ---------- ---------- ---------- ---------- -
PvCRN12         ---------- ---------- ---------- ---------- ---------- ---------- ---------- ---------- -
XP_024576927.1  ---------- ---------- ---------- ---------- ---------- ---------- ---------- ---------- -
XP_024578927.1  ---------- ---------- ---------- ---------- ---------- ---------- ---------- ---------- -
XP_024586212.1  ---------- ---------- ---------- ---------- ---------- ---------- ---------- ---------- -
XP_024578078.1  ---------- ---------- ---------- ---------- ---------- ---------- ---------- ---------- -
XP_024575355.1  ---------- ---------- ---------- ---------- ---------- ---------- ---------- ---------- -
XP_024584527.1  ---------- ---------- ---------- ---------- ---------- ---------- ---------- ---------- -
XP_024586664.1  ---------- ---------- ---------- ---------- ---------- ---------- ---------- ---------- -
PvCRN17         ---------- ---------- ---------- ---------- ---------- ---------- ---------- ---------- -
PvCRN2          ---------- ---------- ---------- ---------- ---------- ---------- ---------- ---------- -
XP_024577181.1  ---------- ---------- ---------- ---------- ---------- ---------- ---------- ---------- -
XP_024581075.1  ---------- ---------- ---------- ---------- ---------- ---------- ---------- ---------- -
XP_024575884.1  ---------- ---------- ---------- ---------- ---------- ---------- ---------- ---------- -
XP_024586872.1  ---------- ---------- ---------- ---------- ---------- ---------- ---------- ---------- -
XP_024573052.1  ---------- ---------- ---------- ---------- ---------- ---------- ---------- ---------- -
XP_024586054.1  ---------- ---------- ---------- ---------- ---------- ---------- ---------- ---------- -
PvCRN27         ---------- ---------- ---------- ---------- ---------- ---------- ---------- ---------- -
PvCRN20         ---------- ---------- ---------- ---------- ---------- ---------- ---------- ---------- -
PvCRN25         ---------- ---------- ---------- ---------- ---------- ---------- ---------- ---------- -
PvCRN1          ---------- ---------- ---------- ---------- ---------- ---------- ---------- ---------- -
PvCRN4          ---------- ---------- ---------- ---------- ---------- ---------- ---------- ---------- -
PvCRN30         ---------- ---------- ---------- ---------- ---------- ---------- ---------- ---------- -
PvCRN29         ---------- ---------- ---------- ---------- ---------- ---------- ---------- ---------- -
XP_024583036.1  ---------- ---------- ---------- ---------- ---------- ---------- ---------- ---------- -
PvCRN10         ---------- ---------- ---------- ---------- ---------- ---------- ---------- ---------- -
XP_024577280.1  ---------- ---------- ---------- ---------- ---------- ---------- ---------- ---------- 51
XP_024572924.1  ---------- ---------- ---------- ---------- ---------- ---------- ---------- ---------- 121
PvCRN14         ---------- ---------- ---------- ---------- ---------- ---------- ---------- ---------- -
XP_024577521.1  ---------- ---------- ---------- ---------- ---------- ---------- ---------- ---------- -
PvCRN21         ---------- ---------- ---------- ---------- ---------- ---------- ---------- ---------- -
XP_024574966.1  ---------- ---------- ---------- ---------- ---------- ---------- ---------- ---------- -
XP_024575372.1  ---------- ---------- ---------- ---------- ---------- ---------- ---------- ---------- -
XP_024581363.1  ---------- ---------- ---------- ---------- ---------- ---------- ---------- ---------- -
XP_024583883.1  ---------- ---------- ---------- ---------- ---------- ---------- ---------- ---------- -
XP_024579844.1  ---------- ---------- ---------- ---------- ---------- ---------- ---------- ---------- -
XP_024574916.1  ---------- ---------- ---------- ---------- ---------- ---------- ---------- ---------- -
XP_024574193.1  ---------- ---------- ---------- ---------- ---------- ---------- ---------- ---------- -
XP_024583155.1  ---------- ---------- ---------- ---------- ---------- ---------- ---------- ---------- -
XP_024574913.1  ---------- ---------- ---------- ---------- ---------- ---------- ---------- ---------- -
XP_024578158.1  ---------- ---------- ---------- ---------- ---------- ---------- ---------- ---------- -
PvCRN22         ---------- ---------- ---------- ---------- ---------- ---------- ---------- ---------- -
XP_024585001.1  ---------- ---------- ---------- ---------- ---------- ---------- ---------- ---------- -
XP_024583843.1  ---------- ---------- ---------- ---------- ---------- ---------- ---------- ---------- -
XP_024578702.1  ---------- ---------- ---------- ---------- ---------- ---------- ---------- ---------- -
XP_024585805.1  ---------- ---------- ---------- ---------- ---------- ---------- ---------- ---------- -
XP_024580932.1  ---------- ---------- ---------- ---------- ---------- ---------- ---------- ---------- -
Consensus       ---------- ---------- ---------- ---------- ---------- ---------- ---------- ----------
           100%
Conservation
             0%
```

```
                 980                 1,000               1,020               1,040
                   |                   |                   |                   |
PvCRN11         ---------- ---------- ---------- ---------- ---------- ---------- ---------- ---------- -
XP_024579260.1  ---------- ---------- ---------- ---------- ---------- ---------- ---------- ---------- -
XP_024573634.1  ---------- ---------- ---------- ---------- ---------- ---------- ---------- ---------- -
PvCRN7          ---------- ---------- ---------- ---------- ---------- ---------- ---------- ---------- -
XP_024585265.1  ---------- ---------- ---------- ---------- ---------- ---------- ---------- ---------- -
PvCRN18         ---------- ---------- ---------- ---------- ---------- ---------- ---------- ---------- -
XP_024578414.1  ---------- ---------- ---------- ---------- ---------- ---------- ---------- ---------- 333
XP_024579130.1  ---------- ---------- ---------- ---------- ---------- ---------- ---------- ---------- 53
XP_024584758.1  ---------- ---------- ---------- ---------- ---------- ---------- ---------- ---------- -
XP_024586066.1  ---------- ---------- ---------- ---------- ---------- ---------- ---------- ---------- 59
XP_024585870.1  ---------- ---------- ---------- ---------- ---------- ---------- ---------- ---------- -
PvCRN31         ---------- ---------- ---------- ---------- ---------- ---------- ---------- ---------- -
XP_024577278.1  ---------- ---------- ---------- ---------- ---------- ---------- ---------- ---------- 57
XP_024585562.1  ---------- ---------- ---------- ---------- ---------- ---------- ---------- ---------- 1
XP_024573944.1  ---------- ---------- ---------- ---------- ---------- ---------- ---------- ---------- -
XP_024579793.1  ---------- ---------- ---------- ---------- ---------- ---------- ---------- ---------- -
PvCRN15         ---------- ---------- ---------- ---------- ---------- ---------- ---------- ---------- -
PvCRN26         ---------- ---------- ---------- ---------- ---------- ---------- ---------- ---------- -
PvCRN16         ---------- ---------- ---------- ---------- ---------- ---------- ---------- ---------- -
PvCRN23         ---------- ---------- ---------- ---------- ---------- ---------- ---------- ---------- -
XP_024573063.1  ---------- ---------- ---------- ---------- ---------- ---------- ---------- ---------- -
PvCRN19         ---------- ---------- ---------- ---------- ---------- ---------- ---------- ---------- -
XP_024581625.1  ---------- ---------- ---------- ---------- ---------- ---------- ---------- ---------- -
XP_024583886.1  ---------- ---------- ---------- ---------- ---------- ---------- ---------- ---------- -
XP_024584409.1  ---------- ---------- ---------- ---------- ---------- ---------- ---------- ---------- -
XP_024572385.1  ---------- ---------- ---------- ---------- ---------- ---------- ---------- ---------- -
PvCRN6          ---------- ---------- ---------- ---------- ---------- ---------- ---------- ---------- -
PvCRN9          ---------- ---------- ---------- ---------- ---------- ---------- ---------- ---------- -
XP_024580875.1  ---------- ---------- ---------- ---------- ---------- ---------- ---------- ---------- -
PvCRN24         ---------- ---------- ---------- ---------- ---------- ---------- ---------- ---------- -
PvCRN35         ---------- ---------- ---------- ---------- ---------- ---------- ---------- ---------- -
PvCRN12         ---------- ---------- ---------- ---------- ---------- ---------- ---------- ---------- -
XP_024576927.1  ---------- ---------- ---------- ---------- ---------- ---------- ---------- ---------- -
XP_024578927.1  ---------- ---------- ---------- ---------- ---------- ---------- ---------- ---------- -
XP_024586212.1  ---------- ---------- ---------- ---------- ---------- ---------- ---------- ---------- -
XP_024578078.1  ---------- ---------- ---------- ---------- ---------- ---------- ---------- ---------- -
XP_024575355.1  ---------- ---------- ---------- ---------- ---------- ---------- ---------- ---------- -
XP_024584527.1  ---------- ---------- ---------- ---------- ---------- ---------- ---------- ---------- -
XP_024586664.1  ---------- ---------- ---------- ---------- ---------- ---------- ---------- ---------- -
PvCRN17         ---------- ---------- ---------- ---------- ---------- ---------- ---------- ---------- -
PvCRN2          ---------- ---------- ---------- ---------- ---------- ---------- ---------- ---------- -
XP_024577181.1  ---------- ---------- ---------- ---------- ---------- ---------- ---------- ---------- -
XP_024581075.1  ---------- ---------- ---------- ---------- ---------- ---------- ---------- ---------- -
XP_024575884.1  ---------- ---------- ---------- ---------- ---------- ---------- ---------- ---------- -
XP_024586872.1  ---------- ---------- ---------- ---------- ---------- ---------- ---------- ---------- -
XP_024573052.1  ---------- ---------- ---------- ---------- ---------- ---------- ---------- ---------- -
XP_024586054.1  ---------- ---------- ---------- ---------- ---------- ---------- ---------- ---------- -
PvCRN27         ---------- ---------- ---------- ---------- ---------- ---------- ---------- ---------- -
PvCRN20         ---------- ---------- ---------- ---------- ---------- ---------- ---------- ---------- -
PvCRN25         ---------- ---------- ---------- ---------- ---------- ---------- ---------- ---------- -
PvCRN1          ---------- ---------- ---------- ---------- ---------- ---------- ---------- ---------- -
PvCRN4          ---------- ---------- ---------- ---------- ---------- ---------- ---------- ---------- -
PvCRN30         ---------- ---------- ---------- ---------- ---------- ---------- ---------- ---------- -
PvCRN29         ---------- ---------- ---------- ---------- ---------- ---------- ---------- ---------- -
XP_024583036.1  ---------- ---------- ---------- ---------- ---------- ---------- ---------- ---------- -
PvCRN10         ---------- ---------- ---------- ---------- ---------- ---------- ---------- ---------- -
XP_024577280.1  ---------- ---------- ---------- ---------- ---------- ---------- ---------- ---------- 51
XP_024572924.1  ---------- ---------- ---------- ---------- ---------- ---------- ---------- ---------- 121
PvCRN14         ---------- ---------- ---------- ---------- ---------- ---------- ---------- ---------- -
XP_024577521.1  ---------- ---------- ---------- ---------- ---------- ---------- ---------- ---------- -
PvCRN21         ---------- ---------- ---------- ---------- ---------- ---------- ---------- ---------- -
XP_024574966.1  ---------- ---------- ---------- ---------- ---------- ---------- ---------- ---------- -
XP_024575372.1  ---------- ---------- ---------- ---------- ---------- ---------- ---------- ---------- -
XP_024581363.1  ---------- ---------- ---------- ---------- ---------- ---------- ---------- ---------- -
XP_024583883.1  ---------- ---------- ---------- ---------- ---------- ---------- ---------- ---------- -
XP_024579844.1  ---------- ---------- ---------- ---------- ---------- ---------- ---------- ---------- -
XP_024574916.1  ---------- ---------- ---------- ---------- ---------- ---------- ---------- ---------- -
XP_024574193.1  ---------- ---------- ---------- ---------- ---------- ---------- ---------- ---------- -
XP_024583155.1  ---------- ---------- ---------- ---------- ---------- ---------- ---------- ---------- -
XP_024574913.1  ---------- ---------- ---------- ---------- ---------- ---------- ---------- ---------- -
XP_024578158.1  ---------- ---------- ---------- ---------- ---------- ---------- ---------- ---------- -
PvCRN22         ---------- ---------- ---------- ---------- ---------- ---------- ---------- ---------- -
XP_024585001.1  ---------- ---------- ---------- ---------- ---------- ---------- ---------- ---------- -
XP_024583843.1  ---------- ---------- ---------- ---------- ---------- ---------- ---------- ---------- -
XP_024578702.1  ---------- ---------- ---------- ---------- ---------- ---------- ---------- ---------- -
XP_024585805.1  ---------- ---------- ---------- ---------- ---------- ---------- ---------- ---------- -
XP_024580932.1  ---------- ---------- ---------- ---------- ---------- ---------- ---------- ---------- -
Consensus       ---------- ---------- ---------- ---------- ---------- ---------- ---------- ----------
      100%
Conservation
        0%
```

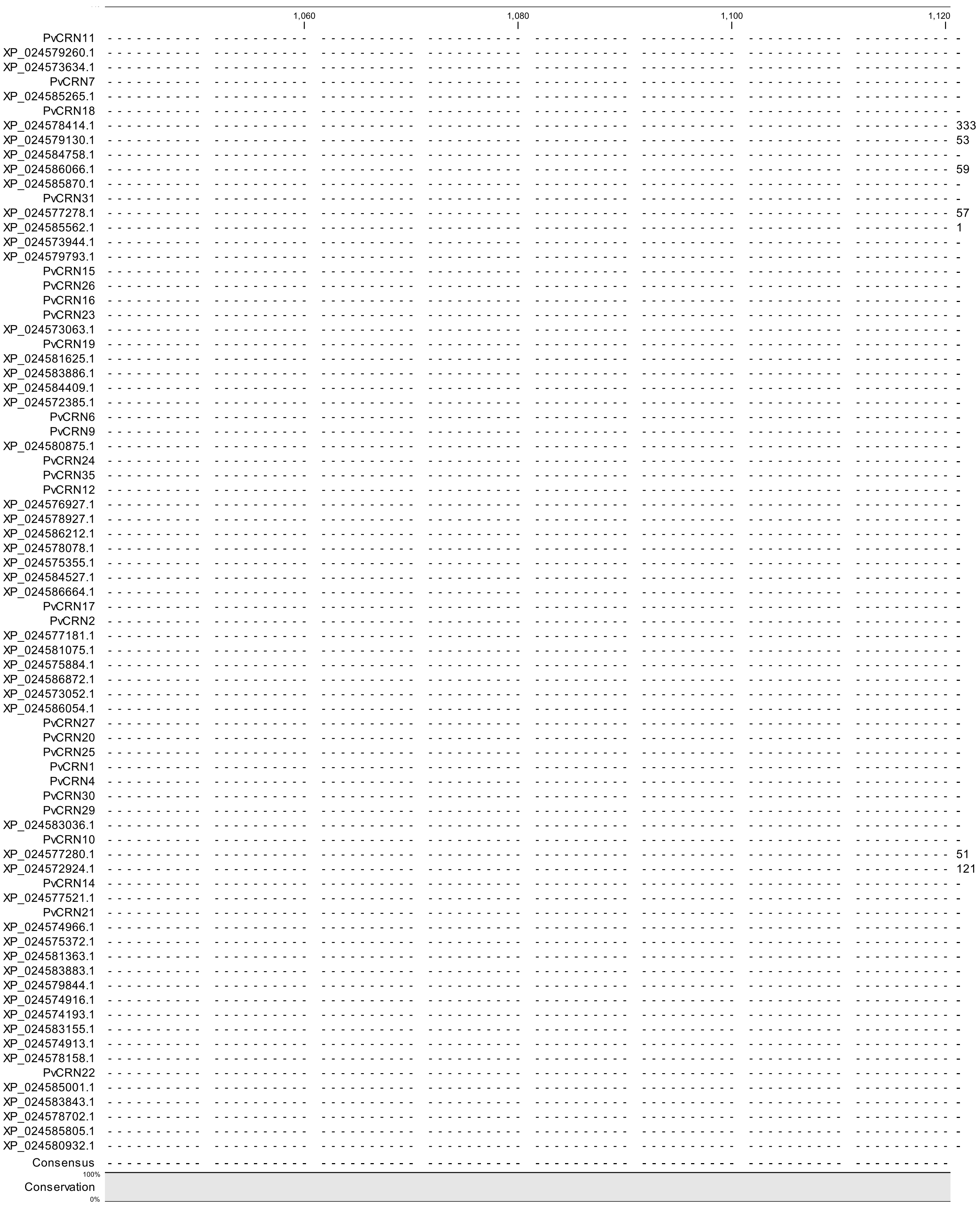
1,060
1,080
1,100
1,120
PvCRN11
XP_024579260.1
XP_024573634.1
PvCRN7
XP_024585265.1
PvCRN18
XP_024578414.1 333
XP_024579130.1 53
XP_024584758.1
XP_024586066.1 59
XP_024585870.1
PvCRN31
XP_024577278.1 57
XP_024585562.1 1
XP_024573944.1
XP_024579793.1
PvCRN15
PvCRN26
PvCRN16
PvCRN23
XP_024573063.1
PvCRN19
XP_024581625.1
XP_024583886.1
XP_024584409.1
XP_024572385.1
PvCRN6
PvCRN9
XP_024580875.1
PvCRN24
PvCRN35
PvCRN12
XP_024576927.1
XP_024578927.1
XP_024586212.1
XP_024578078.1
XP_024575355.1
XP_024584527.1
XP_024586664.1
PvCRN17
PvCRN2
XP_024577181.1
XP_024581075.1
XP_024575884.1
XP_024586872.1
XP_024573052.1
XP_024586054.1
PvCRN27
PvCRN20
PvCRN25
PvCRN1
PvCRN4
PvCRN30
PvCRN29
XP_024583036.1
PvCRN10
XP_024577280.1 51
XP_024572924.1 121
PvCRN14
XP_024577521.1
PvCRN21
XP_024574966.1
XP_024575372.1
XP_024581363.1
XP_024583883.1
XP_024579844.1
XP_024574916.1
XP_024574193.1
XP_024583155.1
XP_024574913.1
XP_024578158.1
PvCRN22
XP_024585001.1
XP_024583843.1
XP_024578702.1
XP_024585805.1
XP_024580932.1
Consensus
100%
Conservation
0%

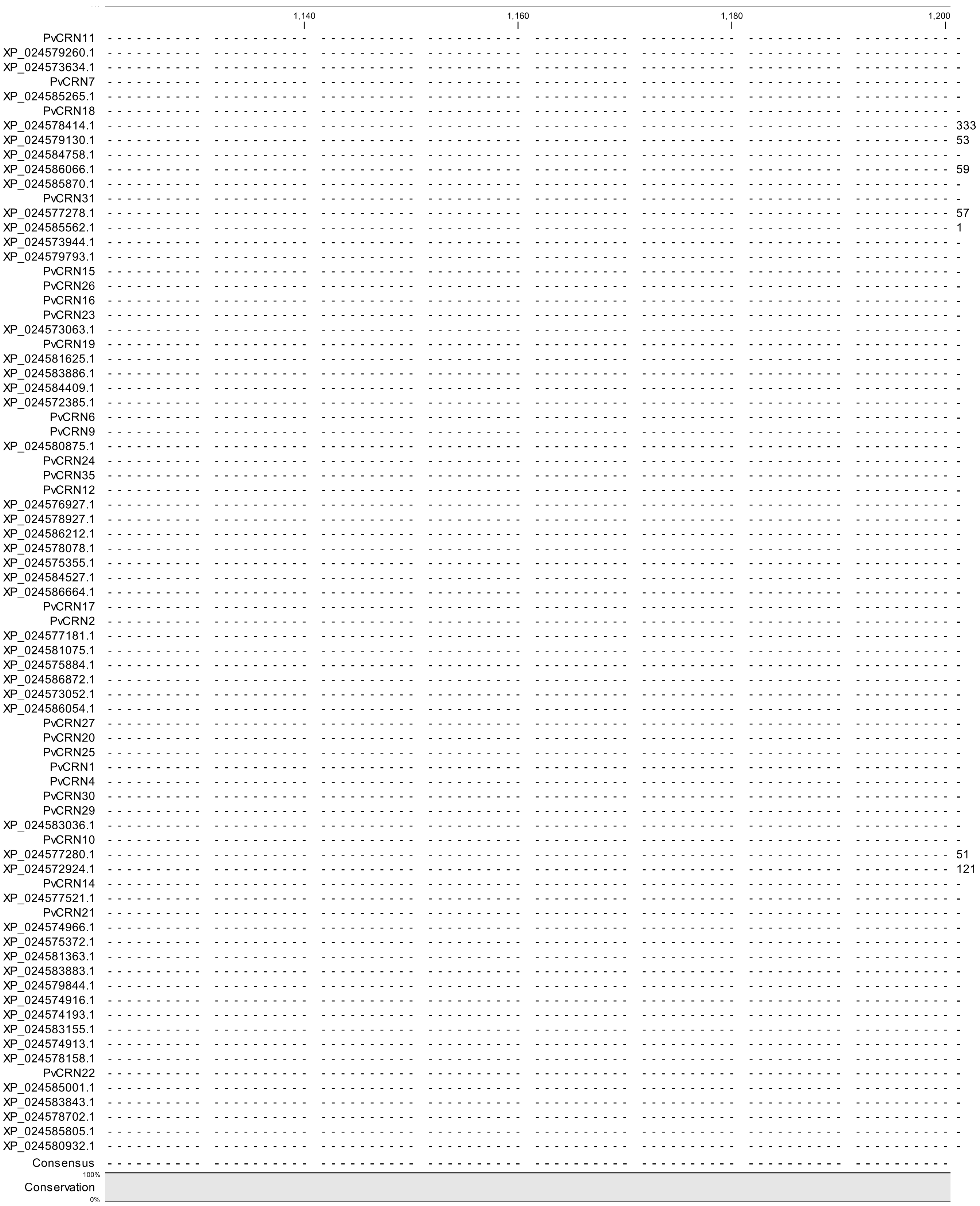
1,140
1,160
1,180
1,200
PvCRN11
XP_024579260.1
XP_024573634.1
PvCRN7
XP_024585265.1
PvCRN18
XP_024578414.1 333
XP_024579130.1 53
XP_024584758.1
XP_024586066.1 59
XP_024585870.1
PvCRN31
XP_024577278.1 57
XP_024585562.1 1
XP_024573944.1
XP_024579793.1
PvCRN15
PvCRN26
PvCRN16
PvCRN23
XP_024573063.1
PvCRN19
XP_024581625.1
XP_024583886.1
XP_024584409.1
XP_024572385.1
PvCRN6
PvCRN9
XP_024580875.1
PvCRN24
PvCRN35
PvCRN12
XP_024576927.1
XP_024578927.1
XP_024586212.1
XP_024578078.1
XP_024575355.1
XP_024584527.1
XP_024586664.1
PvCRN17
PvCRN2
XP_024577181.1
XP_024581075.1
XP_024575884.1
XP_024586872.1
XP_024573052.1
XP_024586054.1
PvCRN27
PvCRN20
PvCRN25
PvCRN1
PvCRN4
PvCRN30
PvCRN29
XP_024583036.1
PvCRN10
XP_024577280.1 51
XP_024572924.1 121
PvCRN14
XP_024577521.1
PvCRN21
XP_024574966.1
XP_024575372.1
XP_024581363.1
XP_024583883.1
XP_024579844.1
XP_024574916.1
XP_024574193.1
XP_024583155.1
XP_024574913.1
XP_024578158.1
PvCRN22
XP_024585001.1
XP_024583843.1
XP_024578702.1
XP_024585805.1
XP_024580932.1
Consensus
100%
Conservation
0%

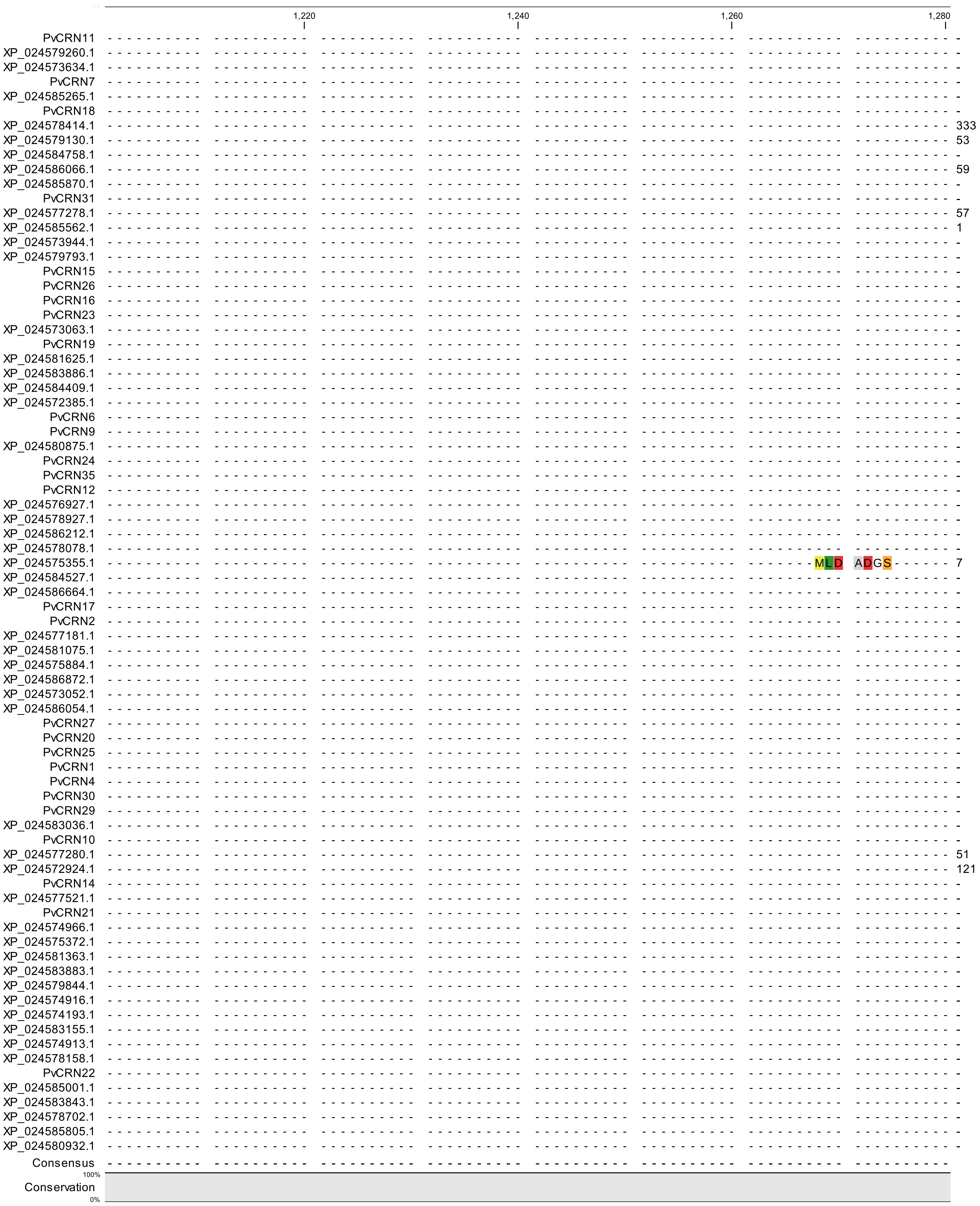
1,220
1,240
1,260
1,280
PvCRN11
XP_024579260.1
XP_024573634.1
PvCRN7
XP_024585265.1
PvCRN18
XP_024578414.1 333
XP_024579130.1 53
XP_024584758.1
XP_024586066.1 59
XP_024585870.1
PvCRN31
XP_024577278.1 57
XP_024585562.1 1
XP_024573944.1
XP_024579793.1
PvCRN15
PvCRN26
PvCRN16
PvCRN23
XP_024573063.1
PvCRN19
XP_024581625.1
XP_024583886.1
XP_024584409.1
XP_024572385.1
PvCRN6
PvCRN9
XP_024580875.1
PvCRN24
PvCRN35
PvCRN12
XP_024576927.1
XP_024578927.1
XP_024586212.1
XP_024578078.1
XP_024575355.1 MLD ADGS 7
XP_024584527.1
XP_024586664.1
PvCRN17
PvCRN2
XP_024577181.1
XP_024581075.1
XP_024575884.1
XP_024586872.1
XP_024573052.1
XP_024586054.1
PvCRN27
PvCRN20
PvCRN25
PvCRN1
PvCRN4
PvCRN30
PvCRN29
XP_024583036.1
PvCRN10
XP_024577280.1 51
XP_024572924.1 121
PvCRN14
XP_024577521.1
PvCRN21
XP_024574966.1
XP_024575372.1
XP_024581363.1
XP_024583883.1
XP_024579844.1
XP_024574916.1
XP_024574193.1
XP_024583155.1
XP_024574913.1
XP_024578158.1
PvCRN22
XP_024585001.1
XP_024583843.1
XP_024578702.1
XP_024585805.1
XP_024580932.1
Consensus
100%
Conservation
0%

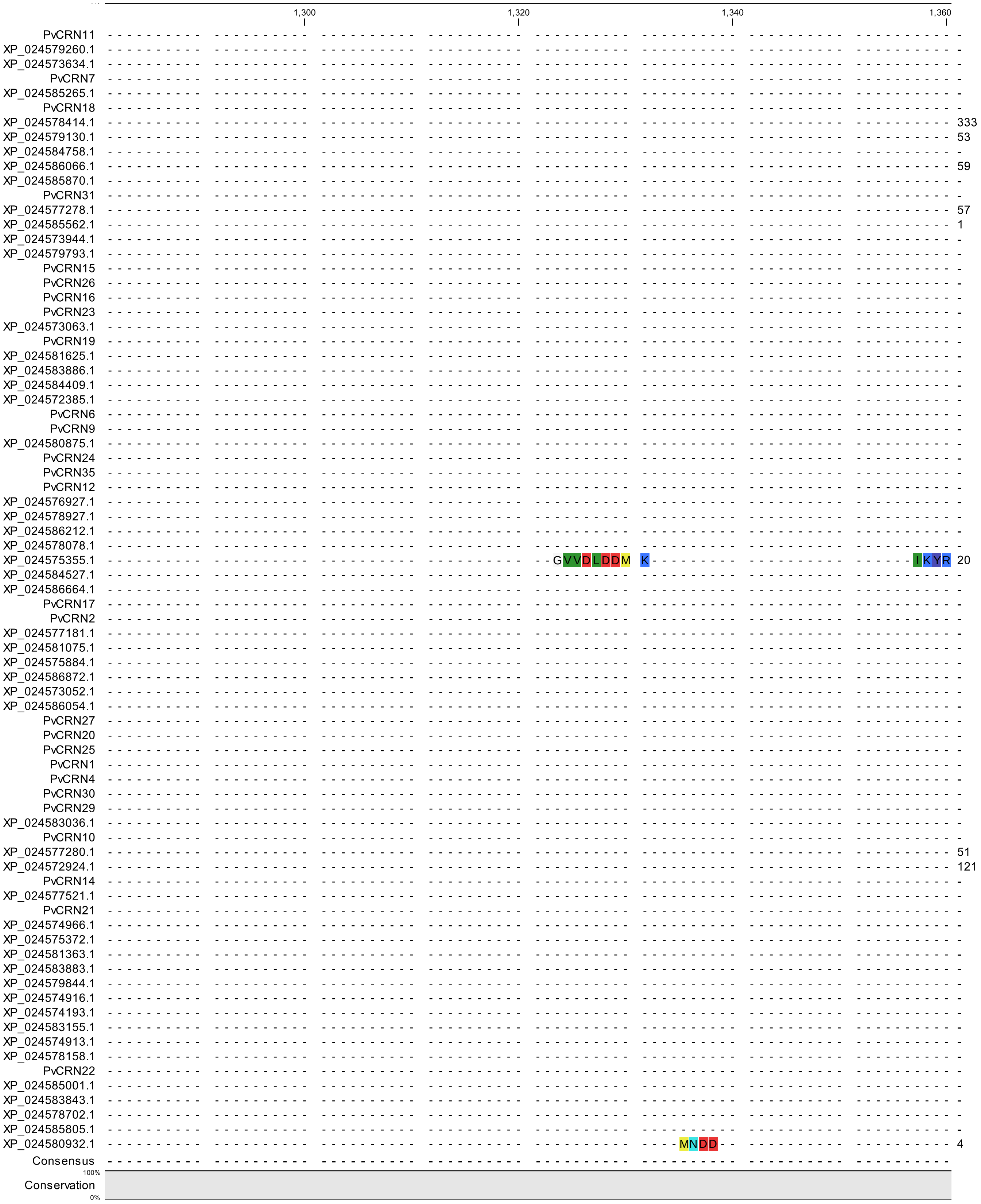
1,300
1,320
1,340
1,360
PvCRN11
XP_024579260.1
XP_024573634.1
PvCRN7
XP_024585265.1
PvCRN18
XP_024578414.1 333
XP_024579130.1 53
XP_024584758.1
XP_024586066.1 59
XP_024585870.1
PvCRN31
XP_024577278.1 57
XP_024585562.1 1
XP_024573944.1
XP_024579793.1
PvCRN15
PvCRN26
PvCRN16
PvCRN23
XP_024573063.1
PvCRN19
XP_024581625.1
XP_024583886.1
XP_024584409.1
XP_024572385.1
PvCRN6
PvCRN9
XP_024580875.1
PvCRN24
PvCRN35
PvCRN12
XP_024576927.1
XP_024578927.1
XP_024586212.1
XP_024578078.1
XP_024575355.1 GVVDLDDM K IKYR 20
XP_024584527.1
XP_024586664.1
PvCRN17
PvCRN2
XP_024577181.1
XP_024581075.1
XP_024575884.1
XP_024586872.1
XP_024573052.1
XP_024586054.1
PvCRN27
PvCRN20
PvCRN25
PvCRN1
PvCRN4
PvCRN30
PvCRN29
XP_024583036.1
PvCRN10
XP_024577280.1 51
XP_024572924.1 121
PvCRN14
XP_024577521.1
PvCRN21
XP_024574966.1
XP_024575372.1
XP_024581363.1
XP_024583883.1
XP_024579844.1
XP_024574916.1
XP_024574193.1
XP_024583155.1
XP_024574913.1
XP_024578158.1
PvCRN22
XP_024585001.1
XP_024583843.1
XP_024578702.1
XP_024585805.1
XP_024580932.1 MNDD 4
Consensus
100%
Conservation
0%

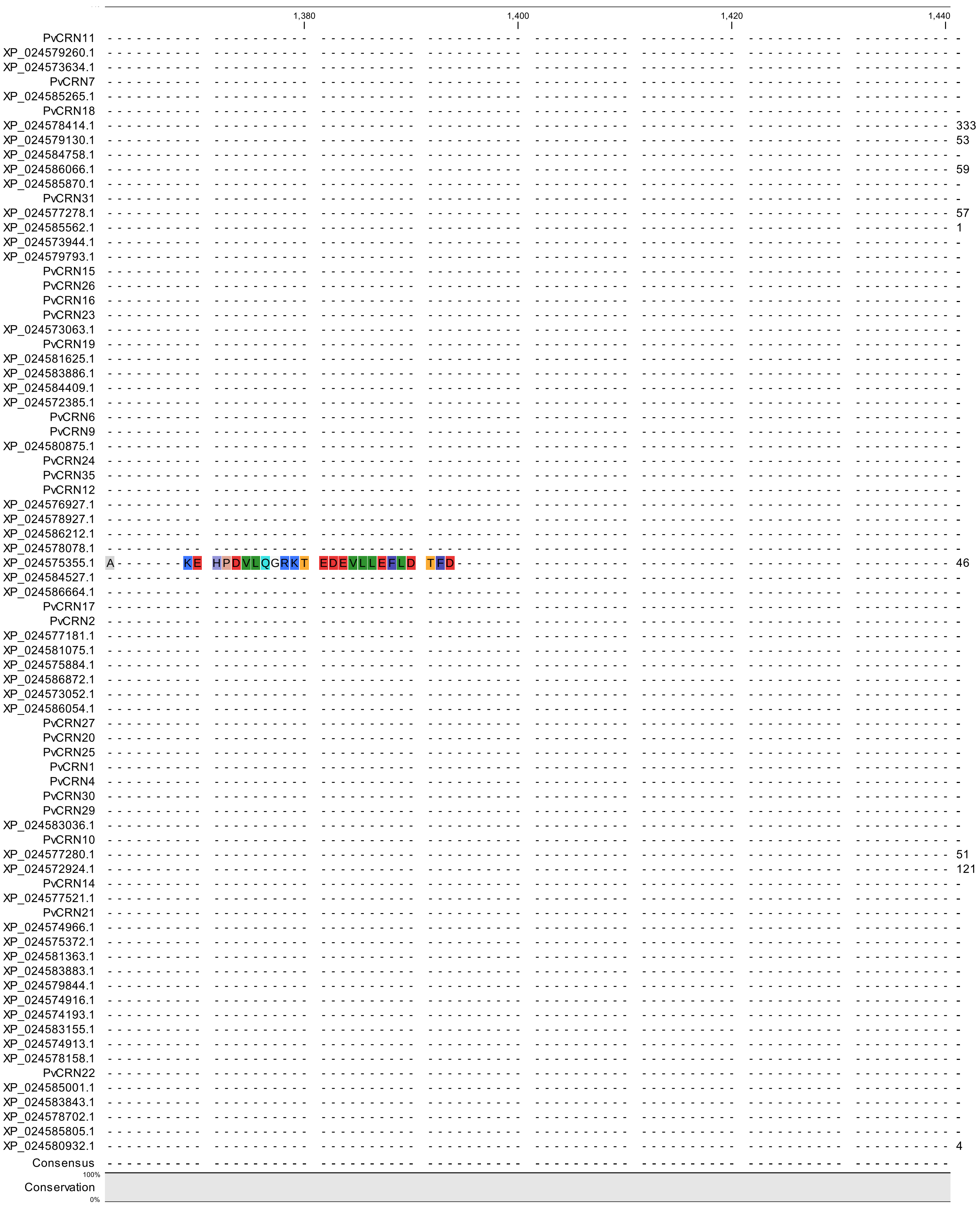
1,380
1,400
1,420
1,440
PvCRN11
XP_024579260.1
XP_024573634.1
PvCRN7
XP_024585265.1
PvCRN18
XP_024578414.1 333
XP_024579130.1 53
XP_024584758.1
XP_024586066.1 59
XP_024585870.1
PvCRN31
XP_024577278.1 57
XP_024585562.1 1
XP_024573944.1
XP_024579793.1
PvCRN15
PvCRN26
PvCRN16
PvCRN23
XP_024573063.1
PvCRN19
XP_024581625.1
XP_024583886.1
XP_024584409.1
XP_024572385.1
PvCRN6
PvCRN9
XP_024580875.1
PvCRN24
PvCRN35
PvCRN12
XP_024576927.1
XP_024578927.1
XP_024586212.1
XP_024578078.1
XP_024575355.1 A KE HPDVLQGRKT EDEVLLEFLD TFD 46
XP_024584527.1
XP_024586664.1
PvCRN17
PvCRN2
XP_024577181.1
XP_024581075.1
XP_024575884.1
XP_024586872.1
XP_024573052.1
XP_024586054.1
PvCRN27
PvCRN20
PvCRN25
PvCRN1
PvCRN4
PvCRN30
PvCRN29
XP_024583036.1
PvCRN10
XP_024577280.1 51
XP_024572924.1 121
PvCRN14
XP_024577521.1
PvCRN21
XP_024574966.1
XP_024575372.1
XP_024581363.1
XP_024583883.1
XP_024579844.1
XP_024574916.1
XP_024574193.1
XP_024583155.1
XP_024574913.1
XP_024578158.1
PvCRN22
XP_024585001.1
XP_024583843.1
XP_024578702.1
XP_024585805.1
XP_024580932.1 4
Consensus
Conservation
100%
0%

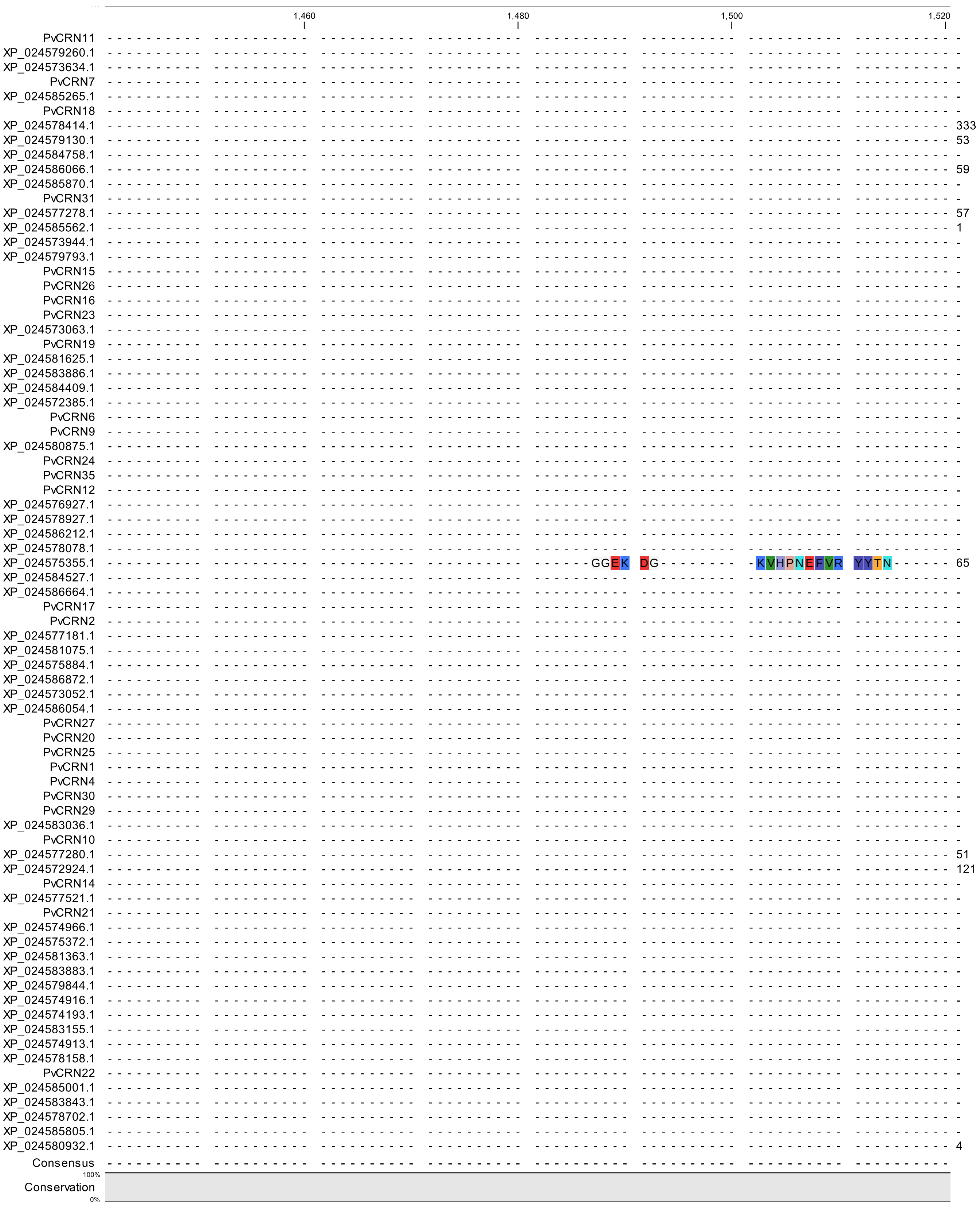
1,460
1,480
1,500
1,520
PvCRN11
XP_024579260.1
XP_024573634.1
PvCRN7
XP_024585265.1
PvCRN18
XP_024578414.1 333
XP_024579130.1 53
XP_024584758.1
XP_024586066.1 59
XP_024585870.1
PvCRN31
XP_024577278.1 57
XP_024585562.1 1
XP_024573944.1
XP_024579793.1
PvCRN15
PvCRN26
PvCRN16
PvCRN23
XP_024573063.1
PvCRN19
XP_024581625.1
XP_024583886.1
XP_024584409.1
XP_024572385.1
PvCRN6
PvCRN9
XP_024580875.1
PvCRN24
PvCRN35
PvCRN12
XP_024576927.1
XP_024578927.1
XP_024586212.1
XP_024578078.1
XP_024575355.1 GGEK DG KVHPNEFVR YYTN 65
XP_024584527.1
XP_024586664.1
PvCRN17
PvCRN2
XP_024577181.1
XP_024581075.1
XP_024575884.1
XP_024586872.1
XP_024573052.1
XP_024586054.1
PvCRN27
PvCRN20
PvCRN25
PvCRN1
PvCRN4
PvCRN30
PvCRN29
XP_024583036.1
PvCRN10
XP_024577280.1 51
XP_024572924.1 121
PvCRN14
XP_024577521.1
PvCRN21
XP_024574966.1
XP_024575372.1
XP_024581363.1
XP_024583883.1
XP_024579844.1
XP_024574916.1
XP_024574193.1
XP_024583155.1
XP_024574913.1
XP_024578158.1
PvCRN22
XP_024585001.1
XP_024583843.1
XP_024578702.1
XP_024585805.1
XP_024580932.1 4
Consensus
100%
Conservation
0%

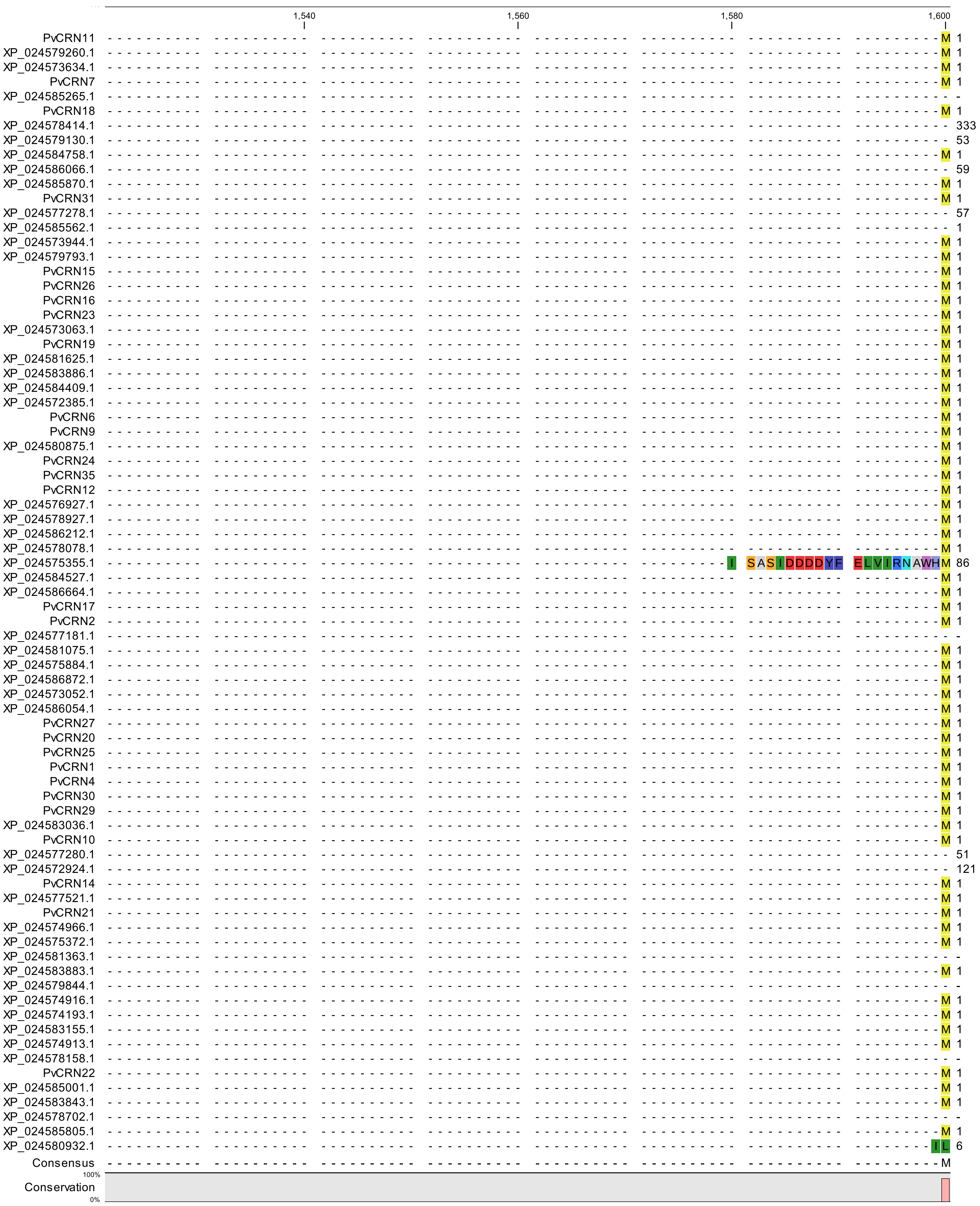
1,540
1,560
1,580
1,600
PvCRN11 M 1
XP_024579260.1 M 1
XP_024573634.1 M 1
PvCRN7 M 1
XP_024585265.1 -
PvCRN18 M 1
XP_024578414.1 333
XP_024579130.1 53
XP_024584758.1 M 1
XP_024586066.1 59
XP_024585870.1 M 1
PvCRN31 M 1
XP_024577278.1 57
XP_024585562.1 1
XP_024573944.1 M 1
XP_024579793.1 M 1
PvCRN15 M 1
PvCRN26 M 1
PvCRN16 M 1
PvCRN23 M 1
XP_024573063.1 M 1
PvCRN19 M 1
XP_024581625.1 M 1
XP_024583886.1 M 1
XP_024584409.1 M 1
XP_024572385.1 M 1
PvCRN6 M 1
PvCRN9 M 1
XP_024580875.1 M 1
PvCRN24 M 1
PvCRN35 M 1
PvCRN12 M 1
XP_024576927.1 M 1
XP_024578927.1 M 1
XP_024586212.1 M 1
XP_024578078.1 M 1
XP_024575355.1 I SASIDDDDYF ELVIRNAWHM 86
XP_024584527.1 M 1
XP_024586664.1 M 1
PvCRN17 M 1
PvCRN2 M 1
XP_024577181.1 -
XP_024581075.1 M 1
XP_024575884.1 M 1
XP_024586872.1 M 1
XP_024573052.1 M 1
XP_024586054.1 M 1
PvCRN27 M 1
PvCRN20 M 1
PvCRN25 M 1
PvCRN1 M 1
PvCRN4 M 1
PvCRN30 M 1
PvCRN29 M 1
XP_024583036.1 M 1
PvCRN10 M 1
XP_024577280.1 51
XP_024572924.1 121
PvCRN14 M 1
XP_024577521.1 M 1
PvCRN21 M 1
XP_024574966.1 M 1
XP_024575372.1 M 1
XP_024581363.1 -
XP_024583883.1 M 1
XP_024579844.1 -
XP_024574916.1 M 1
XP_024574193.1 M 1
XP_024583155.1 M 1
XP_024574913.1 M 1
XP_024578158.1 -
PvCRN22 M 1
XP_024585001.1 M 1
XP_024583843.1 M 1
XP_024578702.1 -
XP_024585805.1 M 1
XP_024580932.1 IL 6
Consensus M
100%
Conservation
0%

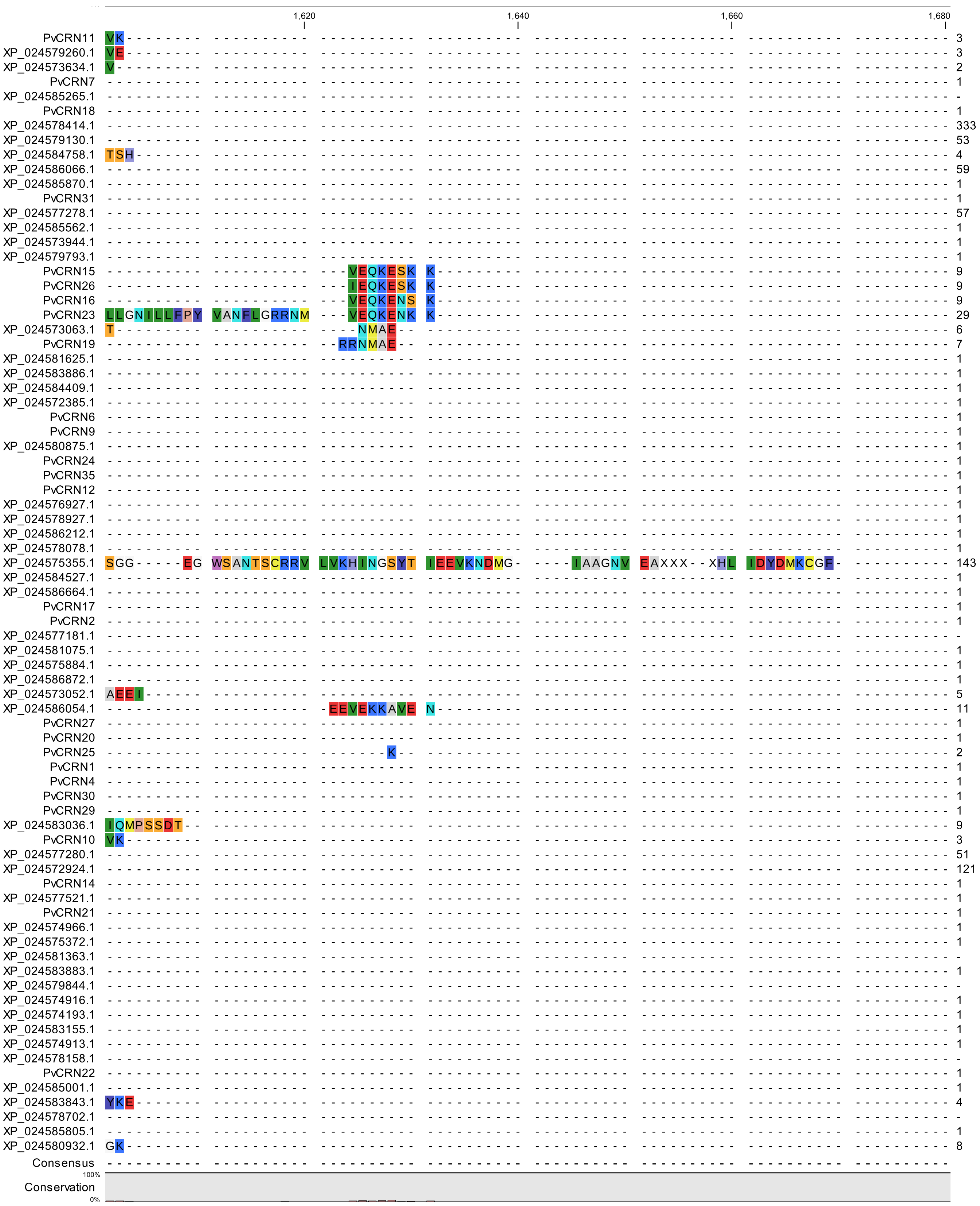
1,620
1,640
1,660
1,680
PvCRN11 VK 3
XP_024579260.1 VE 3
XP_024573634.1 V 2
PvCRN7 1
XP_024585265.1
PvCRN18 1
XP_024578414.1 333
XP_024579130.1 53
XP_024584758.1 TSH 4
XP_024586066.1 59
XP_024585870.1 1
PvCRN31 1
XP_024577278.1 57
XP_024585562.1 1
XP_024573944.1 1
XP_024579793.1 1
PvCRN15 VEQKESK K 9
PvCRN26 IEQKESK K 9
PvCRN16 VEQKENS K 9
PvCRN23 LLGNILLFPY VANFLGRRNM VEQKENK K 29
XP_024573063.1 T NMAE 6
PvCRN19 RRNMAE 7
XP_024581625.1 1
XP_024583886.1 1
XP_024584409.1 1
XP_024572385.1 1
PvCRN6 1
PvCRN9 1
XP_024580875.1 1
PvCRN24 1
PvCRN35 1
PvCRN12 1
XP_024576927.1 1
XP_024578927.1 1
XP_024586212.1 1
XP_024578078.1 1
XP_024575355.1 SGG EG WSANTSCRRV LVKHINGSYT IEEVKNDMG IAAGNV EAXXX XHL IDYDMKCGF 143
XP_024584527.1 1
XP_024586664.1 1
PvCRN17 1
PvCRN2 1
XP_024577181.1
XP_024581075.1 1
XP_024575884.1 1
XP_024586872.1 1
XP_024573052.1 AEEI 5
XP_024586054.1 EEVEKKAVE N 11
PvCRN27 1
PvCRN20 1
PvCRN25 K 2
PvCRN1 1
PvCRN4 1
PvCRN30 1
PvCRN29 1
XP_024583036.1 IQMPSSDT 9
PvCRN10 VK 3
XP_024577280.1 51
XP_024572924.1 121
PvCRN14 1
XP_024577521.1 1
PvCRN21 1
XP_024574966.1 1
XP_024575372.1 1
XP_024581363.1
XP_024583883.1 1
XP_024579844.1
XP_024574916.1 1
XP_024574193.1 1
XP_024583155.1 1
XP_024574913.1 1
XP_024578158.1
PvCRN22 1
XP_024585001.1 1
XP_024583843.1 YKE 4
XP_024578702.1
XP_024585805.1 1
XP_024580932.1 GK 8
Consensus
100%
Conservation
0%

```
                  1,700               1,720               1,740               1,760
PvCRN11          ---------- ---------- ---------- ---------- ---------- ---------- ---------- ---------- 3
XP_024579260.1   ---------- ---------- ---------- ---------- ---------- ---------- ---------- ---------- 3
XP_024573634.1   ---------- ---------- ---------- ---------- ---------- ---------- ---------- ---------- 2
PvCRN7           ---------- ---------- ---------- ---------- ---------- ---------- ---------- ---------- 1
XP_024585265.1   ---------- ---------- ---------- ---------- ---------- ---------- ---------- ---------- -
PvCRN18          ---------- ---------- ---------- ---------- ---------- ---------- ---------- ---------- 1
XP_024578414.1   ---------- ---------- ---------- ---------- ---------- ---------- ---------- ---------- 333
XP_024579130.1   ---------- ---------- ---------- ---------- ---------- ---------- ---------- ---------- 53
XP_024584758.1   ---------- ---------- ---------- ---------- ---------- ---------- ---------- ---------- 4
XP_024586066.1   ---------- ---------- ---------- ---------- ---------- ---------- ---------- ---------- 59
XP_024585870.1   ---------- ---------- ---------- ---------- ---------- ---------- ---------- ---------- 1
PvCRN31          ---------- ---------- ---------- ---------- ---------- ---------- ---------- ---------- 1
XP_024577278.1   ---------- ---------- ---------- ---------- ---------- ---------- ---------- ---------- 57
XP_024585562.1   ---------- ---------- ---------- ---------- ---------- ---------- ---------- ---------- 1
XP_024573944.1   ---------- ---------- ---------- ---------- ---------- ---------- ---------- ---------- 1
XP_024579793.1   ---------- ---------- ---------- ---------- ---------- ---------- ---------- ---------- 1
PvCRN15          ---------- ---------- ---------- ---------- ---------- ---------- ---------- ---------- 9
PvCRN26          ---------- ---------- ---------- ---------- ---------- ---------- ---------- ---------- 9
PvCRN16          ---------- ---------- ---------- ---------- ---------- ---------- ---------- ---------- 9
PvCRN23          ---------- ---------- ---------- ---------- ---------- ---------- ---------- ---------- 29
XP_024573063.1   ---------- ---------- ---------- ---------- ---------- ---------- ---------- ---------- 6
PvCRN19          ---------- ---------- ---------- ---------- ---------- ---------- ---------- ---------- 7
XP_024581625.1   ---------- ---------- ---------- ---------- ---------- ---------- ---------- ---------- 1
XP_024583886.1   ---------- ---------- ---------- ---------- ---------- ---------- ---------- ---------- 1
XP_024584409.1   ---------- ---------- ---------- ---------- ---------- ---------- ---------- ---------- 1
XP_024572385.1   ---------- ---------- ---------- ---------- ---------- ---------- ---------- ---------- 1
PvCRN6           ---------- ---------- ---------- ---------- ---------- ---------- ---------- ---------- 1
PvCRN9           ---------- ---------- ---------- ---------- ---------- ---------- ---------- ---------- 1
XP_024580875.1   ---------- ---------- ---------- ---------- ---------- ---------- ---------- ---------- 1
PvCRN24          ---------- ---------- ---------- ---------- ---------- ---------- ---------- ---------- 1
PvCRN35          ---------- ---------- ---------- ---------- ---------- ---------- ---------- ---------- 1
PvCRN12          ---------- ---------- ---------- ---------- ---------- ---------- ---------- ---------- 1
XP_024576927.1   ---------- ---------- ---------- ---------- ---------- ---------- ---------- ---------- 1
XP_024578927.1   ---------- ---------- ---------- ---------- ---------- ---------- ---------- ---------- 1
XP_024586212.1   ---------- ---------- ---------- ---------- ---------- ---------- ---------- ---------- 1
XP_024578078.1   ---------- ---------- ---------- ---------- ---------- ---------- ---------- ---------- 1
XP_024575355.1   ---------- ---------- ---------- ---------- ---------- ---------- ---------- ---------- 143
XP_024584527.1   ---------- ---------- ---------- ---------- ---------- ---------- ---------- ---------- 1
XP_024586664.1   ---------- ---------- ---------- ---------- ---------- ---------- ---------- ---------- 1
PvCRN17          ---------- ---------- ---------- ---------- ---------- ---------- ---------- ---------- 1
PvCRN2           ---------- ---------- ---------- ---------- ---------- ---------- ---------- ---------- 1
XP_024577181.1   ---------- ---------- ---------- ---------- ---------- ---------- ---------- ---------- -
XP_024581075.1   ---------- ---------- ---------- ---------- ---------- ---------- ---------- ---------- 1
XP_024575884.1   ---------- ---------- ---------- ---------- ---------- ---------- ---------- ---------- 1
XP_024586872.1   ---------- ---------- ---------- ---------- ---------- ---------- ---------- ---------- 1
XP_024573052.1   ---------- ---------- ---------- ---------- ---------- ---------- ---------- ---------- 5
XP_024586054.1   ---------- ---------- ---------- ---------- ---------- ---------- ---------- ---------- 11
PvCRN27          ---------- ---------- ---------- ---------- ---------- ---------- ---------- ---------- 1
PvCRN20          ---------- ---------- ---------- ---------- ---------- ---------- ---------- ---------- 1
PvCRN25          ---------- ---------- ---------- ---------- ---------- ---------- ---------- ---------- 2
PvCRN1           ---------- ---------- ---------- ---------- ---------- ---------- ---------- ---------- 1
PvCRN4           ---------- ---------- ---------- ---------- ---------- ---------- ---------- ---------- 1
PvCRN30          ---------- ---------- ---------- ---------- ---------- ---------- ---------- ---------- 1
PvCRN29          ---------- ---------- ---------- ---------- ---------- ---------- ---------- ---------- 1
XP_024583036.1   ---------- ---------- ---------- ---------- ---------- ---------- ---------- ---------- 9
PvCRN10          ---------- ---------- ---------- ---------- ---------- ---------- ---------- ---------- 3
XP_024577280.1   ---------- ---------- ---------- ---------- ---------- ---------- ---------- ---------- 51
XP_024572924.1   ---------- ---------- ---------- ---------- ---------- ---------- ---------- ---------- 121
PvCRN14          ---------- ---------- ---------- ---------- ---------- ---------- ---------- ---------- 1
XP_024577521.1   ---------- ---------- ---------- ---------- ---------- ---------- ---------- ---------- 1
PvCRN21          ---------- ---------- ---------- ---------- ---------- ---------- ---------- ---------- 1
XP_024574966.1   ---------- ---------- ---------- ---------- ---------- ---------- ---------- ---------- 1
XP_024575372.1   ---------- ---------- ---------- ---------- ---------- ---------- ---------- ---------- 1
XP_024581363.1   ---------- ---------- ---------- ---------- ---------- ---------- ---------- ---------- -
XP_024583883.1   ---------- ---------- ---------- ---------- ---------- ---------- ---------- ---------- 1
XP_024579844.1   ---------- ---------- ---------- ---------- ---------- ---------- ---------- ---------- -
XP_024574916.1   ---------- ---------- ---------- ---------- ---------- ---------- ---------- ---------- 1
XP_024574193.1   ---------- ---------- ---------- ---------- ---------- ---------- ---------- ---------- 1
XP_024583155.1   ---------- ---------- ---------- ---------- ---------- ---------- ---------- ---------- 1
XP_024574913.1   ---------- ---------- ---------- ---------- ---------- ---------- ---------- ---------- 1
XP_024578158.1   ---------- ---------- ---------- ---------- ---------- ---------- ---------- ---------- -
PvCRN22          ---------- ---------- ---------- ---------- ---------- ---------- ---------- ---------- 1
XP_024585001.1   ---------- ---------- ---------- ---------- ---------- ---------- ---------- ---------- 1
XP_024583843.1   ---------- ---------- ---------- ---------- ---------- ---------- ---------- ---------- 4
XP_024578702.1   ---------- ---------- ---------- ---------- ---------- ---------- ---------- ---------- -
XP_024585805.1   ---------- ---------- ---------- ---------- ---------- ---------- ---------- ---------- 1
XP_024580932.1   ---------- ---------- ---------- ---------- ---------- ---------- ---------- ---------- 8
Consensus        ---------- ---------- ---------- ---------- ---------- ---------- ---------- ----------
          100%
Conservation
            0%
```

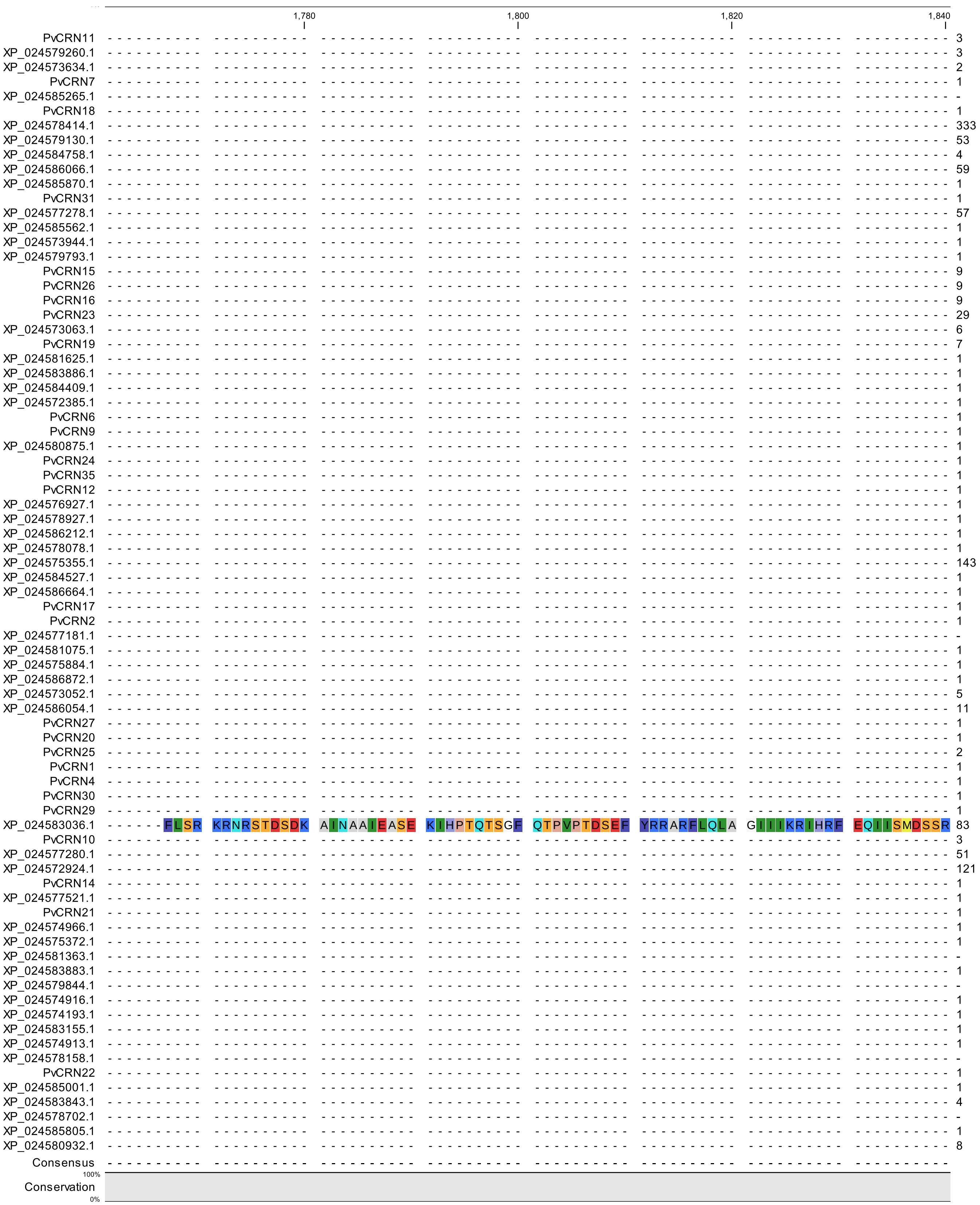

1,780
1,800
1,820
1,840
PvCRN11 3
XP_024579260.1 3
XP_024573634.1 2
PvCRN7 1
XP_024585265.1 -
PvCRN18 1
XP_024578414.1 333
XP_024579130.1 53
XP_024584758.1 4
XP_024586066.1 59
XP_024585870.1 1
PvCRN31 1
XP_024577278.1 57
XP_024585562.1 1
XP_024573944.1 1
XP_024579793.1 1
PvCRN15 9
PvCRN26 9
PvCRN16 9
PvCRN23 29
XP_024573063.1 6
PvCRN19 7
XP_024581625.1 1
XP_024583886.1 1
XP_024584409.1 1
XP_024572385.1 1
PvCRN6 1
PvCRN9 1
XP_024580875.1 1
PvCRN24 1
PvCRN35 1
PvCRN12 1
XP_024576927.1 1
XP_024578927.1 1
XP_024586212.1 1
XP_024578078.1 1
XP_024575355.1 143
XP_024584527.1 1
XP_024586664.1 1
PvCRN17 1
PvCRN2 1
XP_024577181.1 -
XP_024581075.1 1
XP_024575884.1 1
XP_024586872.1 1
XP_024573052.1 5
XP_024586054.1 11
PvCRN27 1
PvCRN20 1
PvCRN25 2
PvCRN1 1
PvCRN4 1
PvCRN30 1
PvCRN29 1
XP_024583036.1 FLSR KRNRSTDSDK AINAAIEASE KIHPTQTSGF QTPVPTDSEF YRRARFLQLA GIIIKRIHRF EQIISMDSSR 83
PvCRN10 3
XP_024577280.1 51
XP_024572924.1 121
PvCRN14 1
XP_024577521.1 1
PvCRN21 1
XP_024574966.1 1
XP_024575372.1 1
XP_024581363.1 -
XP_024583883.1 1
XP_024579844.1 -
XP_024574916.1 1
XP_024574193.1 1
XP_024583155.1 1
XP_024574913.1 1
XP_024578158.1 -
PvCRN22 1
XP_024585001.1 1
XP_024583843.1 4
XP_024578702.1 -
XP_024585805.1 1
XP_024580932.1 8
Consensus
100%
Conservation
0%

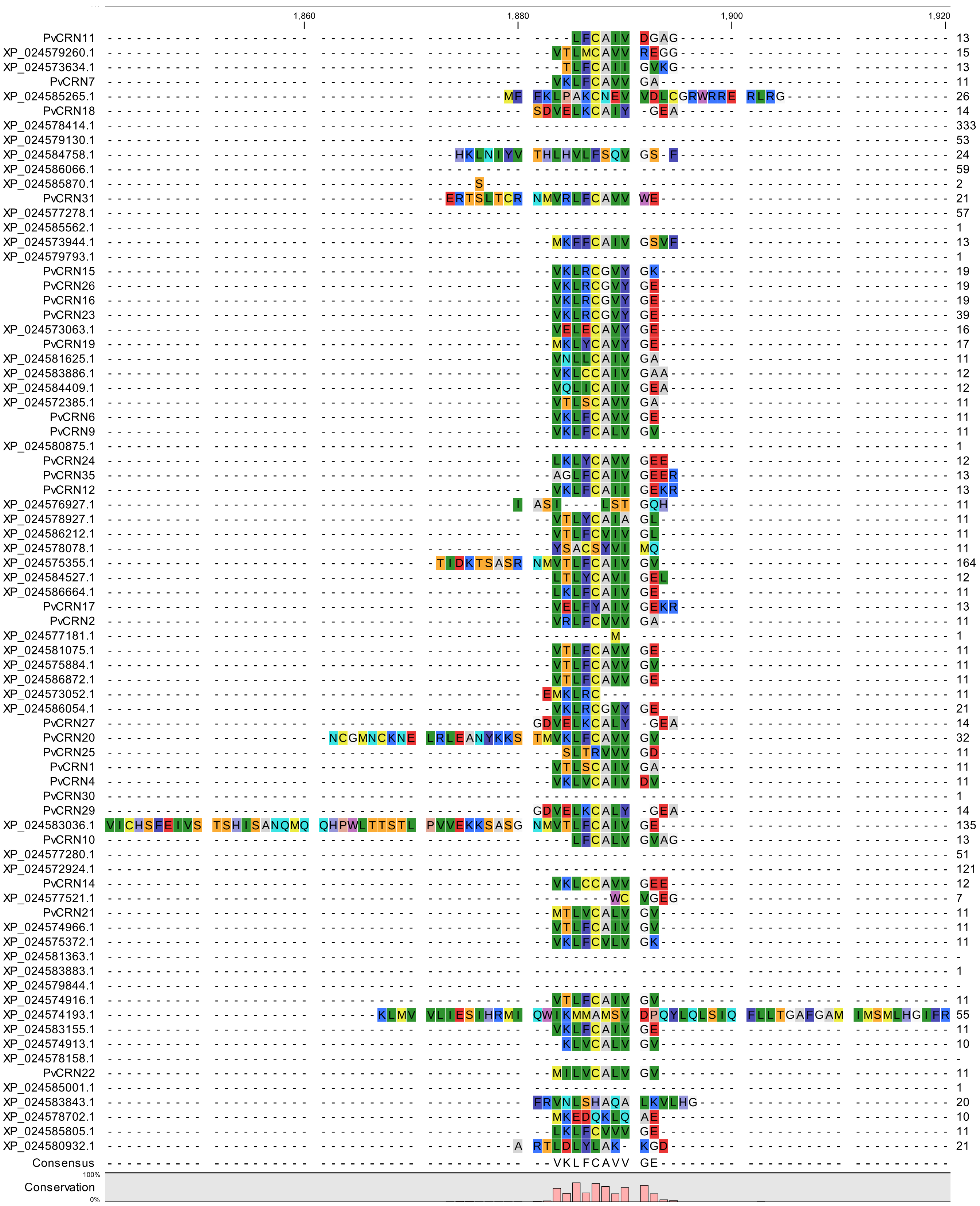

1,860
1,880
1,900
1,920
PvCRN11 LFCAIV DGAG 13
XP_024579260.1 VTLMCAVV REGG 15
XP_024573634.1 TLFCAII GVKG 13
PvCRN7 VKLFCAVV GA 11
XP_024585265.1 MF FKLPAKCNEV VDLCGRWRRE RLRG 26
PvCRN18 SDVELKCAIY -GEA 14
XP_024578414.1 333
XP_024579130.1 53
XP_024584758.1 HKLNIYV THLHVLFSQV GS-F 24
XP_024586066.1 59
XP_024585870.1 S 2
PvCRN31 ERTSLTCR NMVRLFCAVV WE 21
XP_024577278.1 57
XP_024585562.1 1
XP_024573944.1 MKFFCAIV GSVF 13
XP_024579793.1 1
PvCRN15 VKLRCGVY GK 19
PvCRN26 VKLRCGVY GE 19
PvCRN16 VKLRCGVY GE 19
PvCRN23 VKLRCGVY GE 39
XP_024573063.1 VELECAVY GE 16
PvCRN19 MKLYCAVY GE 17
XP_024581625.1 VNLLCAIV GA 11
XP_024583886.1 VKLCCAIV GAA 12
XP_024584409.1 VQLICAIV GEA 12
XP_024572385.1 VTLSCAVV GA 11
PvCRN6 VKLFCAVV GE 11
PvCRN9 VKLFCALV GV 11
XP_024580875.1 1
PvCRN24 LKLYCAVV GEE 12
PvCRN35 AGLFCAIV GEER 13
PvCRN12 VKLFCAII GEKR 13
XP_024576927.1 I ASI----LST GQH 11
XP_024578927.1 VTLYCAIA GL 11
XP_024586212.1 VTLFCVIV GL 11
XP_024578078.1 YSACSYVI MQ 11
XP_024575355.1 TIDKTSASR NMVTLFCAIV GV 164
XP_024584527.1 LTLYCAVI GEL 12
XP_024586664.1 LKLFCAIV GE 11
PvCRN17 VELFYAIV GEKR 13
PvCRN2 VRLFCVVV GA 11
XP_024577181.1 M 1
XP_024581075.1 VTLFCAVV GE 11
XP_024575884.1 VTLFCAVV GV 11
XP_024586872.1 VTLFCAVV GE 11
XP_024573052.1 EMKLRC 11
XP_024586054.1 VKLRCGVY GE 21
PvCRN27 GDVELKCALY -GEA 14
PvCRN20 NCGMNCKNE LRLEANYKKS TMVKLFCAVV GV 32
PvCRN25 SLTRVVV GD 11
PvCRN1 VTLSCAIV GA 11
PvCRN4 VKLVCAIV DV 11
PvCRN30 1
PvCRN29 GDVELKCALY -GEA 14
XP_024583036.1 VICHSFEIVS TSHISANQMQ QHPWLTTSTL PVVEKKSASG NMVTLFCAIV GE 135
PvCRN10 LFCALV GVAG 13
XP_024577280.1 51
XP_024572924.1 121
PvCRN14 VKLCCAVV GEE 12
XP_024577521.1 WC VGEG 7
PvCRN21 MTLVCALV GV 11
XP_024574966.1 VTLFCAIV GV 11
XP_024575372.1 VKLFCVLV GK 11
XP_024581363.1 -
XP_024583883.1 1
XP_024579844.1 -
XP_024574916.1 VTLFCAIV GV 11
XP_024574193.1 KLMV VLIESIHRMI QWIKMMAMSV DPQYLQLSIQ FLLTGAFGAM IMSMLHGIFR 55
XP_024583155.1 VKLFCAIV GE 11
XP_024574913.1 KLVCALV GV 10
XP_024578158.1 -
PvCRN22 MILVCALV GV 11
XP_024585001.1 1
XP_024583843.1 FRVNLSHAQA LKVLHG 20
XP_024578702.1 MKEDQKLQ AE 10
XP_024585805.1 LKLFCVVV GE 11
XP_024580932.1 A RTLDLYLAK- KGD 21
Consensus VKLFCAVV GE
Conservation
100%
0%

| | 1,940 · 1,960 · 1,980 · 2,000 | |
|---|---|---|
| PvCRN11 | ---------- ---------- ---------- ---------- ---------- ---------- ---------- ---------- | 13 |
| XP_024579260.1 | ---------- ---------- ---------- ---------- ---------- ---------- ---------- ---------- | 15 |
| XP_024573634.1 | ---------- ---------- ---------- ---------- ---------- ---------- ---------- ---------- | 13 |
| PvCRN7 | ---------- ---------- ---------- ---------- ---------- ---------- ---------- ---------- | 11 |
| XP_024585265.1 | ---------- ---------- ---------- ---------- ---------- ---------- ---------- ---------- | 26 |
| PvCRN18 | ---------- ---------- ---------- ---------- ---------- ---------- ---------- ---------- | 14 |
| XP_024578414.1 | ---------- ---------- ---------- ---------- ---------- ---------- ---------- ---------- | 333 |
| XP_024579130.1 | ---------- ---------- ---------- ---------- ---------- ---------- ---------- ---------- | 53 |
| XP_024584758.1 | ---------- ---------- ---------- ---------- ---------- ---------- ---------- ---------- | 24 |
| XP_024586066.1 | ---------- ---------- ---------- ---------- ---------- ---------- ---------- ---------- | 59 |
| XP_024585870.1 | ---------- ---------- ---------- ---------- ---------- ---------- ---------- ---------- | 2 |
| PvCRN31 | ---------- ---------- ---------- ---------- ---------- ---------- ---------- ---------- | 21 |
| XP_024577278.1 | ---------- ---------- ---------- ---------- ---------- ---------- ---------- ---------- | 57 |
| XP_024585562.1 | ---------- ---------- ---------- ---------- ---------- ---------- ---------- ---------- | 1 |
| XP_024573944.1 | ---------- ---------- ---------- ---------- ---------- ---------- ---------- ---------- | 13 |
| XP_024579793.1 | ---------- ---------- ---------- ---------- ---------- ---------- ---------- ---------- | 1 |
| PvCRN15 | ---------- ---------- ---------- ---------- ---------- ---------- ---------- ---------- | 19 |
| PvCRN26 | ---------- ---------- ---------- ---------- ---------- ---------- ---------- ---------- | 19 |
| PvCRN16 | ---------- ---------- ---------- ---------- ---------- ---------- ---------- ---------- | 19 |
| PvCRN23 | ---------- ---------- ---------- ---------- ---------- ---------- ---------- ---------- | 39 |
| XP_024573063.1 | ---------- ---------- ---------- ---------- ---------- ---------- ---------- ---------- | 16 |
| PvCRN19 | ---------- ---------- ---------- ---------- ---------- ---------- ---------- ---------- | 17 |
| XP_024581625.1 | ---------- ---------- ---------- ---------- ---------- ---------- ---------- ---------- | 11 |
| XP_024583886.1 | ---------- ---------- ---------- ---------- ---------- ---------- ---------- ---------- | 12 |
| XP_024584409.1 | ---------- ---------- ---------- ---------- ---------- ---------- ---------- ---------- | 12 |
| XP_024572385.1 | ---------- ---------- ---------- ---------- ---------- ---------- ---------- ---------- | 11 |
| PvCRN6 | ---------- ---------- ---------- ---------- ---------- ---------- ---------- ---------- | 11 |
| PvCRN9 | ---------- ---------- ---------- ---------- ---------- ---------- ---------- ---------- | 11 |
| XP_024580875.1 | ---------- ---------- ---------- ---------- ---------- ---------- ---------- ---------- | 1 |
| PvCRN24 | ---------- ---------- ---------- ---------- ---------- ---------- ---------- ---------- | 12 |
| PvCRN35 | ---------- ---------- ---------- ---------- ---------- ---------- ---------- ---------- | 13 |
| PvCRN12 | ---------- ---------- ---------- ---------- ---------- ---------- ---------- ---------- | 13 |
| XP_024576927.1 | ---------- ---------- ---------- ---------- ---------- ---------- ---------- ---------- | 11 |
| XP_024578927.1 | ---------- ---------- ---------- ---------- ---------- ---------- ---------- ---------- | 11 |
| XP_024586212.1 | ---------- ---------- ---------- ---------- ---------- ---------- ---------- ---------- | 11 |
| XP_024578078.1 | ---------- ---------- ---------- ---------- ---------- ---------- ---------- ---------- | 11 |
| XP_024575355.1 | ---------- ---------- ---------- ---------- ---------- ---------- ---------- ---------- | 164 |
| XP_024584527.1 | ---------- ---------- ---------- ---------- ---------- ---------- ---------- ---------- | 12 |
| XP_024586664.1 | ---------- ---------- ---------- ---------- ---------- ---------- ---------- ---------- | 11 |
| PvCRN17 | ---------- ---------- ---------- ---------- ---------- ---------- ---------- ---------- | 13 |
| PvCRN2 | ---------- ---------- ---------- ---------- ---------- ---------- ---------- ---------- | 11 |
| XP_024577181.1 | ---------- ---------- ---------- ---------- ---------- ---------- ---------- ---------- | 1 |
| XP_024581075.1 | ---------- ---------- ---------- ---------- ---------- ---------- ---------- ---------- | 11 |
| XP_024575884.1 | ---------- ---------- ---------- ---------- ---------- ---------- ---------- ---------- | 11 |
| XP_024586872.1 | ---------- ---------- ---------- ---------- ---------- ---------- ---------- ---------- | 11 |
| XP_024573052.1 | ---------- ---------- ---------- ---------- ---------- ---------- ---------- ---------- | 11 |
| XP_024586054.1 | ---------- ---------- ---------- ---------- ---------- ---------- ---------- ---------- | 21 |
| PvCRN27 | ---------- ---------- ---------- ---------- ---------- ---------- ---------- ---------- | 14 |
| PvCRN20 | ---------- ---------- ---------- ---------- ---------- ---------- ---------- ---------- | 32 |
| PvCRN25 | ---------- ---------- ---------- ---------- ---------- ---------- ---------- ---------- | 11 |
| PvCRN1 | ---------- ---------- ---------- ---------- ---------- ---------- ---------- ---------- | 11 |
| PvCRN4 | ---------- ---------- ---------- ---------- ---------- ---------- ---------- ---------- | 11 |
| PvCRN30 | ---------- ---------- ---------- ---------- ---------- ---------- ---------- ---------- | 1 |
| PvCRN29 | ---------- ---------- ---------- ---------- ---------- ---------- ---------- ---------- | 14 |
| XP_024583036.1 | ---------- ---------- ---------- ---------- ---------- ---------- ---------- ---------- | 135 |
| PvCRN10 | ---------- ---------- ---------- ---------- ---------- ---------- ---------- ---------- | 13 |
| XP_024577280.1 | ---------- ---------- ---------- ---------- ---------- ---------- ---------- ---------- | 51 |
| XP_024572924.1 | ---------- ---------- ---------- ---------- ---------- ---------- ---------- ---------- | 121 |
| PvCRN14 | ---------- ---------- ---------- ---------- ---------- ---------- ---------- ---------- | 12 |
| XP_024577521.1 | ---------- ---------- ---------- ---------- ---------- ---------- ---------- ---------- | 7 |
| PvCRN21 | ---------- ---------- ---------- ---------- ---------- ---------- ---------- ---------- | 11 |
| XP_024574966.1 | ---------- ---------- ---------- ---------- ---------- ---------- ---------- ---------- | 11 |
| XP_024575372.1 | ---------- ---------- ---------- ---------- ---------- ---------- ---------- ---------- | 11 |
| XP_024581363.1 | ---------- ---------- ---------- ---------- ---------- ---------- ---------- ---------- | - |
| XP_024583883.1 | ---------- ---------- ---------- ---------- ---------- ---------- ---------- ---------- | 1 |
| XP_024579844.1 | ---------- ---------- ---------- ---------- ---------- ---------- ---------- ---------- | - |
| XP_024574916.1 | ---------- ---------- ---------- ---------- ---------- ---------- ---------- ---------- | 11 |
| XP_024574193.1 | GICV------ ---------- ---------- ---------- ---------- ---------- ---------- ---------- | 59 |
| XP_024583155.1 | ---------- ---------- ---------- ---------- ---------- ---------- ---------- ---------- | 11 |
| XP_024574913.1 | ---------- ---------- ---------- ---------- ---------- ---------- ---------- ---------- | 10 |
| XP_024578158.1 | ---------- ---------- ---------- ---------- ---------- ---------- ---------- ---------- | - |
| PvCRN22 | ---------- ---------- ---------- ---------- ---------- ---------- ---------- ---------- | 11 |
| XP_024585001.1 | ---------- ---------- ---------- ---------- ---------- ---------- ---------- ---------- | 1 |
| XP_024583843.1 | ---------- ---------- ---------- ---------- ---------- ---------- ---------- ---------- | 20 |
| XP_024578702.1 | ---------- ---------- ---------- ---------- ---------- ---------- ---------- ---------- | 10 |
| XP_024585805.1 | ---------- ---------- ---------- ---------- ---------- ---------- ---------- ---------- | 11 |
| XP_024580932.1 | ---------- ---------- ---------- ---------- ---------- ---------- ---------- ---------- | 21 |
| Consensus | ---------- ---------- ---------- ---------- ---------- ---------- ---------- ---------- | |
| Conservation (100% / 0%) | | |

| | 2,020 · 2,040 · 2,060 · 2,080 | |
|---|---|---|
| PvCRN11 | ---------- ---------- ---------- ---------- ---------- ---------- ---------- ---------- | 13 |
| XP_024579260.1 | ---------- ---------- ---------- ---------- ---------- ---------- ---------- ---------- | 15 |
| XP_024573634.1 | ---------- ---------- ---------- ---------- ---------- ---------- ---------- ---------- | 13 |
| PvCRN7 | ---------- ---------- ---------- ---------- ---------- ---------- ---------- ---------- | 11 |
| XP_024585265.1 | ---------- ---------- ---------- ---------- ---------- ---------- ---------- ---------- | 26 |
| PvCRN18 | ---------- ---------- ---------- ---------- ---------- ---------- ---------- ---------- | 14 |
| XP_024578414.1 | ---------- ---------- ---------- ---------- ---------- ---------- ---------- ---------- | 333 |
| XP_024579130.1 | ---------- ---------- ---------- ---------- ---------- ---------- ---------- ---------- | 53 |
| XP_024584758.1 | ---------- ---------- ---------- ---------- ---------- ---------- ---------- ---------- | 24 |
| XP_024586066.1 | ---------- ---------- ---------- ---------- ---------- ---------- ---------- ---------- | 59 |
| XP_024585870.1 | ---------- ---------- ---------- ---------- ---------- ---------- ---------- ---------- | 2 |
| PvCRN31 | ---------- ---------- ---------- ---------- ---------- ---------- ---------- ---------- | 21 |
| XP_024577278.1 | ---------- ---------- ---------- ---------- ---------- ---------- ---------- ---------- | 57 |
| XP_024585562.1 | ---------- ---------- ---------- ---------- ---------- ---------- ---------- ---------- | 1 |
| XP_024573944.1 | ---------- ---------- ---------- ---------- ---------- ---------- ---------- ---------- | 13 |
| XP_024579793.1 | ---------- ---------- ---------- ---------- ---------- ---------- ---------- ---------- | 1 |
| PvCRN15 | ---------- ---------- ---------- ---------- ---------- ---------- ---------- ---------- | 19 |
| PvCRN26 | ---------- ---------- ---------- ---------- ---------- ---------- ---------- ---------- | 19 |
| PvCRN16 | ---------- ---------- ---------- ---------- ---------- ---------- ---------- ---------- | 19 |
| PvCRN23 | ---------- ---------- ---------- ---------- ---------- ---------- ---------- ---------- | 39 |
| XP_024573063.1 | ---------- ---------- ---------- ---------- ---------- ---------- ---------- ---------- | 16 |
| PvCRN19 | ---------- ---------- ---------- ---------- ---------- ---------- ---------- ---------- | 17 |
| XP_024581625.1 | ---------- ---------- ---------- ---------- ---------- ---------- ---------- ---------- | 11 |
| XP_024583886.1 | ---------- ---------- ---------- ---------- ---------- ---------- ---------- ---------- | 12 |
| XP_024584409.1 | ---------- ---------- ---------- ---------- ---------- ---------- ---------- ---------- | 12 |
| XP_024572385.1 | ---------- ---------- ---------- ---------- ---------- ---------- ---------- ---------- | 11 |
| PvCRN6 | ---------- ---------- ---------- ---------- ---------- ---------- ---------- ---------- | 11 |
| PvCRN9 | ---------- ---------- ---------- ---------- ---------- ---------- ---------- ---------- | 11 |
| XP_024580875.1 | ---------- ---------- ---------- ---------- ---------- ---------- ---------- ---------- | 1 |
| PvCRN24 | ---------- ---------- ---------- ---------- ---------- ---------- ---------- ---------- | 12 |
| PvCRN35 | ---------- ---------- ---------- ---------- ---------- ---------- ---------- ---------- | 13 |
| PvCRN12 | ---------- ---------- ---------- ---------- ---------- ---------- ---------- ---------- | 13 |
| XP_024576927.1 | ---------- ---------- ---------- ---------- ---------- ---------- ---------- ---------- | 11 |
| XP_024578927.1 | ---------- ---------- ---------- ---------- ---------- ---------- ---------- ---------- | 11 |
| XP_024586212.1 | ---------- ---------- ---------- ---------- ---------- ---------- ---------- ---------- | 11 |
| XP_024578078.1 | ---------- ---------- ---------- ---------- ---------- ---------- ---------- ---------- | 11 |
| XP_024575355.1 | ---------- ---------- ---------- ---------- ---------- ---------- ---------- ---------- | 164 |
| XP_024584527.1 | ---------- ---------- ---------- ---------- ---------- ---------- ---------- ---------- | 12 |
| XP_024586664.1 | ---------- ---------- ---------- ---------- ---------- ---------- ---------- ---------- | 11 |
| PvCRN17 | ---------- ---------- ---------- ---------- ---------- ---------- ---------- ---------- | 13 |
| PvCRN2 | ---------- ---------- ---------- ---------- ---------- ---------- ---------- ---------- | 11 |
| XP_024577181.1 | ---------- ---------- ---------- ---------- ---------- ---------- ---------- ---------- | 1 |
| XP_024581075.1 | ---------- ---------- ---------- ---------- ---------- ---------- ---------- ---------- | 11 |
| XP_024575884.1 | ---------- ---------- ---------- ---------- ---------- ---------- ---------- ---------- | 11 |
| XP_024586872.1 | ---------- ---------- ---------- ---------- ---------- ---------- ---------- ---------- | 11 |
| XP_024573052.1 | ---------- ---------- ---------- ---------- ---------- ---------- ---------- ---------- | 11 |
| XP_024586054.1 | ---------- ---------- ---------- ---------- ---------- ---------- ---------- ---------- | 21 |
| PvCRN27 | ---------- ---------- ---------- ---------- ---------- ---------- ---------- ---------- | 14 |
| PvCRN20 | ---------- ---------- ---------- ---------- ---------- ---------- ---------- ---------- | 32 |
| PvCRN25 | ---------- ---------- ---------- ---------- ---------- ---------- ---------- ---------- | 11 |
| PvCRN1 | ---------- ---------- ---------- ---------- ---------- ---------- ---------- ---------- | 11 |
| PvCRN4 | ---------- ---------- ---------- ---------- ---------- ---------- ---------- ---------- | 11 |
| PvCRN30 | ---------- ---------- ---------- ---------- ---------- ---------- ---------- ---------- | 1 |
| PvCRN29 | ---------- ---------- ---------- ---------- ---------- ---------- ---------- ---------- | 14 |
| XP_024583036.1 | ---------- ---------- ---------- ---------- ---------- ---------- ---------- ---------- | 135 |
| PvCRN10 | ---------- ---------- ---------- ---------- ---------- ---------- ---------- ---------- | 13 |
| XP_024577280.1 | ---------- ---------- ---------- ---------- ---------- ---------- ---------- ---------- | 51 |
| XP_024572924.1 | ---------- ---------- ---------- ---------- ---------- ---------- ---------- ---------- | 121 |
| PvCRN14 | ---------- ---------- ---------- ---------- ---------- ---------- ---------- ---------- | 12 |
| XP_024577521.1 | ---------- ---------- ---------- ---------- ---------- ---------- ---------- ---------- | 7 |
| PvCRN21 | ---------- ---------- ---------- ---------- ---------- ---------- ---------- ---------- | 11 |
| XP_024574966.1 | ---------- ---------- ---------- ---------- ---------- ---------- ---------- ---------- | 11 |
| XP_024575372.1 | ---------- ---------- ---------- ---------- ---------- ---------- ---------- ---------- | 11 |
| XP_024581363.1 | ---------- ---------- ---------- ---------- ---------- ---------- ---------- ---------- | - |
| XP_024583883.1 | ---------- ---------- ---------- ---------- ---------- ---------- ---------- ---------- | 1 |
| XP_024579844.1 | ---------- ---------- ---------- ---------- ---------- ---------- ---------- ---------- | - |
| XP_024574916.1 | ---------- ---------- ---------- ---------- ---------- ---------- ---------- ---------- | 11 |
| XP_024574193.1 | ---------- ---------- ---------- ---------- ---------- ---------- ---------- ---------- | 59 |
| XP_024583155.1 | ---------- ---------- ---------- ---------- ---------- ---------- ---------- ---------- | 11 |
| XP_024574913.1 | ---------- ---------- ---------- ---------- ---------- ---------- ---------- ---------- | 10 |
| XP_024578158.1 | ---------- ---M------ ---------- ---------- ---------- ---------- ---------- ---------- | 1 |
| PvCRN22 | ---------- ---------- ---------- ---------- ---------- ---------- ---------- ---------- | 11 |
| XP_024585001.1 | ---------- ---------- ---------- ---------- ---------- ---------- ---------- ---------- | 1 |
| XP_024583843.1 | ---------- ---------- ---------- ---------- ---------- ---------- ---------- ---------- | 20 |
| XP_024578702.1 | ---------- ---------- ---------- ---------- ---------- ---------- ---------- ---------- | 10 |
| XP_024585805.1 | ---------- ---------- ---------- ---------- ---------- ---------- ---------- ---------- | 11 |
| XP_024580932.1 | ---------- ---------- ---------- ---------- ---------- ---------- ---------- ---------- | 21 |
| Consensus | ---------- ---------- ---------- ---------- ---------- ---------- ---------- ---------- | |
| Conservation (100% / 0%) | | |

| | 2,100 / 2,120 / 2,140 / 2,160 | |
|---|---|---|
| PvCRN11 | ---------- ---------- ---------- ---------- ---------- ---------- ---------- ---------- | 13 |
| XP_024579260.1 | ---------- ---------- ---------- ---------- ---------- ---------- ---------- ---------- | 15 |
| XP_024573634.1 | ---------- ---------- ---------- ---------- ---------- ---------- ---------- ---------- | 13 |
| PvCRN7 | ---------- ---------- ---------- ---------- ---------- ---------- ---------- ---------- | 11 |
| XP_024585265.1 | ---------- ---------- ---------- ---------- ---------- ---------- ---------- ---------- | 26 |
| PvCRN18 | ---------- ---------- ---------- ---------- ---------- ---------- ---------- ---------- | 14 |
| XP_024578414.1 | ---------- ---------- ---------- ---------- ---------- ---------- ---------- ---------- | 333 |
| XP_024579130.1 | ---------- ---------- ---------- ---------- ---------- ---------- ---------- ---------- | 53 |
| XP_024584758.1 | ---------- ---------- ---------- ---------- ---------- ---------- ---------- ---------- | 24 |
| XP_024586066.1 | ---------- ---------- ---------- ---------- ---------- ---------- ---------- ---------- | 59 |
| XP_024585870.1 | ---------- ---------- ---------- ---------- ---------- ---------- ---------- ---------- | 2 |
| PvCRN31 | ---------- ---------- ---------- ---------- ---------- ---------- ---------- ---------- | 21 |
| XP_024577278.1 | ---------- ---------- ---------- ---------- ---------- ---------- ---------- ---------- | 57 |
| XP_024585562.1 | ---------- ---------- ---------- ---------- ---------- ---------- ---------- ---------- | 1 |
| XP_024573944.1 | ---------- ---------- ---------- ---------- ---------- ---------- ---------- ---------- | 13 |
| XP_024579793.1 | ---------- ---------- ---------- ---------- ---------- ---------- ---------- ---------- | 1 |
| PvCRN15 | ---------- ---------- ---------- ---------- ---------- ---------- ---------- ---------- | 19 |
| PvCRN26 | ---------- ---------- ---------- ---------- ---------- ---------- ---------- ---------- | 19 |
| PvCRN16 | ---------- ---------- ---------- ---------- ---------- ---------- ---------- ---------- | 19 |
| PvCRN23 | ---------- ---------- ---------- ---------- ---------- ---------- ---------- ---------- | 39 |
| XP_024573063.1 | ---------- ---------- ---------- ---------- ---------- ---------- ---------- ---------- | 16 |
| PvCRN19 | ---------- ---------- ---------- ---------- ---------- ---------- ---------- ---------- | 17 |
| XP_024581625.1 | ---------- ---------- ---------- ---------- ---------- ---------- ---------- ---------- | 11 |
| XP_024583886.1 | ---------- ---------- ---------- ---------- ---------- ---------- ---------- ---------- | 12 |
| XP_024584409.1 | ---------- ---------- ---------- ---------- ---------- ---------- ---------- ---------- | 12 |
| XP_024572385.1 | ---------- ---------- ---------- ---------- ---------- ---------- ---------- ---------- | 11 |
| PvCRN6 | ---------- ---------- ---------- ---------- ---------- ---------- ---------- ---------- | 11 |
| PvCRN9 | ---------- ---------- ---------- ---------- ---------- ---------- ---------- ---------- | 11 |
| XP_024580875.1 | ---------- ---------- ---------- ---------- ---------- ---------- ---------- ---------- | 1 |
| PvCRN24 | ---------- ---------- ---------- ---------- ---------- ---------- ---------- ---------- | 12 |
| PvCRN35 | ---------- ---------- ---------- ---------- ---------- ---------- ---------- ---------- | 13 |
| PvCRN12 | ---------- ---------- ---------- ---------- ---------- ---------- ---------- ---------- | 13 |
| XP_024576927.1 | ---------- ---------- ---------- ---------- ---------- ---------- ---------- ---------- | 11 |
| XP_024578927.1 | ---------- ---------- ---------- ---------- ---------- ---------- ---------- ---------- | 11 |
| XP_024586212.1 | ---------- ---------- ---------- ---------- ---------- ---------- ---------- ---------- | 11 |
| XP_024578078.1 | ---------- ---------- ---------- ---------- ---------- ---------- ---------- ---------- | 11 |
| XP_024575355.1 | ---------- ---------- ---------- ---------- ---------- ---------- ---------- ---------- | 164 |
| XP_024584527.1 | ---------- ---------- ---------- ---------- ---------- ---------- ---------- ---------- | 12 |
| XP_024586664.1 | ---------- ---------- ---------- ---------- ---------- ---------- ---------- ---------- | 11 |
| PvCRN17 | ---------- ---------- ---------- ---------- ---------- ---------- ---------- ---------- | 13 |
| PvCRN2 | ---------- ---------- ---------- ---------- ---------- ---------- ---------- ---------- | 11 |
| XP_024577181.1 | ---------- ---------- ---------- ---------- ---------- ---------- ---------- ---------- | 1 |
| XP_024581075.1 | ---------- ---------- ---------- ---------- ---------- ---------- ---------- ---------- | 11 |
| XP_024575884.1 | ---------- ---------- ---------- ---------- ---------- ---------- ---------- ---------- | 11 |
| XP_024586872.1 | ---------- ---------- ---------- ---------- ---------- ---------- ---------- ---------- | 11 |
| XP_024573052.1 | ---------- ---------- ---------- ---------- ---------- ---------- ---------- ---------- | 11 |
| XP_024586054.1 | ---------- ---------- ---------- ---------- ---------- ---------- ---------- ---------- | 21 |
| PvCRN27 | ---------- ---------- ---------- ---------- ---------- ---------- ---------- ---------- | 14 |
| PvCRN20 | ---------- ---------- ---------- ---------- ---------- ---------- ---------- ---------- | 32 |
| PvCRN25 | ---------- ---------- ---------- ---------- ---------- ---------- ---------- ---------- | 11 |
| PvCRN1 | ---------- ---------- ---------- ---------- ---------- ---------- ---------- ---------- | 11 |
| PvCRN4 | ---------- ---------- ---------- ---------- ---------- ---------- ---------- ---------- | 11 |
| PvCRN30 | ---------- ---------- ---------- ---------- ---------- ---------- ---------- ---------- | 1 |
| PvCRN29 | ---------- ---------- ---------- ---------- ---------- ---------- ---------- ---------- | 14 |
| XP_024583036.1 | ---------- ---------- ---------- ---------- ---------- ---------- ---------- ---------- | 135 |
| PvCRN10 | ---------- ---------- ---------- ---------- ---------- ---------- ---------- ---------- | 13 |
| XP_024577280.1 | ---------- ---------- ---------- ---------- ---------- ---------- ---------- ---------- | 51 |
| XP_024572924.1 | ---------- ---------- ---------- ---------- ---------- ---------- ---------- ---------- | 121 |
| PvCRN14 | ---------- ---------- ---------- ---------- ---------- ---------- ---------- ---------- | 12 |
| XP_024577521.1 | ---------- ---------- ---------- ---------- ---------- ---------- ---------- ---------- | 7 |
| PvCRN21 | ---------- ---------- ---------- ---------- ---------- ---------- ---------- ---------- | 11 |
| XP_024574966.1 | ---------- ---------- ---------- ---------- ---------- ---------- ---------- ---------- | 11 |
| XP_024575372.1 | ---------- ---------- ---------- ---------- ---------- ---------- ---------- ---------- | 11 |
| XP_024581363.1 | ---------- ---------- ---------- ---------- ---------- ---------- ---------- ---------- | - |
| XP_024583883.1 | ---------- ---------- ---------- ---------- ---------- ---------- ---------- ---------- | 1 |
| XP_024579844.1 | ---------- ---------- ---------- ---------- ---------- ---------- ---------- ---------- | - |
| XP_024574916.1 | ---------- ---------- ---------- ---------- ---------- ---------- ---------- ---------- | 11 |
| XP_024574193.1 | ---------- ---------- ---------- ---------- ---------- ---------- ---------- ---------- | 59 |
| XP_024583155.1 | ---------- ---------- ---------- ---------- ---------- ---------- ---------- ---------- | 11 |
| XP_024574913.1 | ---------- ---------- ---------- ---------- ---------- ---------- ---------- ---------- | 10 |
| XP_024578158.1 | ---------- ---------- ---------- ---------- ---------- ---------- ---------- ---------- | 1 |
| PvCRN22 | ---------- ---------- ---------- ---------- ---------- ---------- ---------- ---------- | 11 |
| XP_024585001.1 | ---------- ---------- ---------- ---------- ---------- ---------- ---------- ---------- | 1 |
| XP_024583843.1 | ---------- ---------- ---------- ---------- ---------- ---------- ---------- ---------- | 20 |
| XP_024578702.1 | ---------- ---------- ---------- ---------- ---------- ---------- ---------- ---------- | 10 |
| XP_024585805.1 | ---------- ---------- ---------- ---------- ---------- ---------- ---------- ---------- | 11 |
| XP_024580932.1 | ---------- ---------- ---------- ---------- ---------- ---------- ---------- ---------- | 21 |
| Consensus | ---------- ---------- ---------- ---------- ---------- ---------- ---------- ---------- | |

Conservation (100% / 0%)

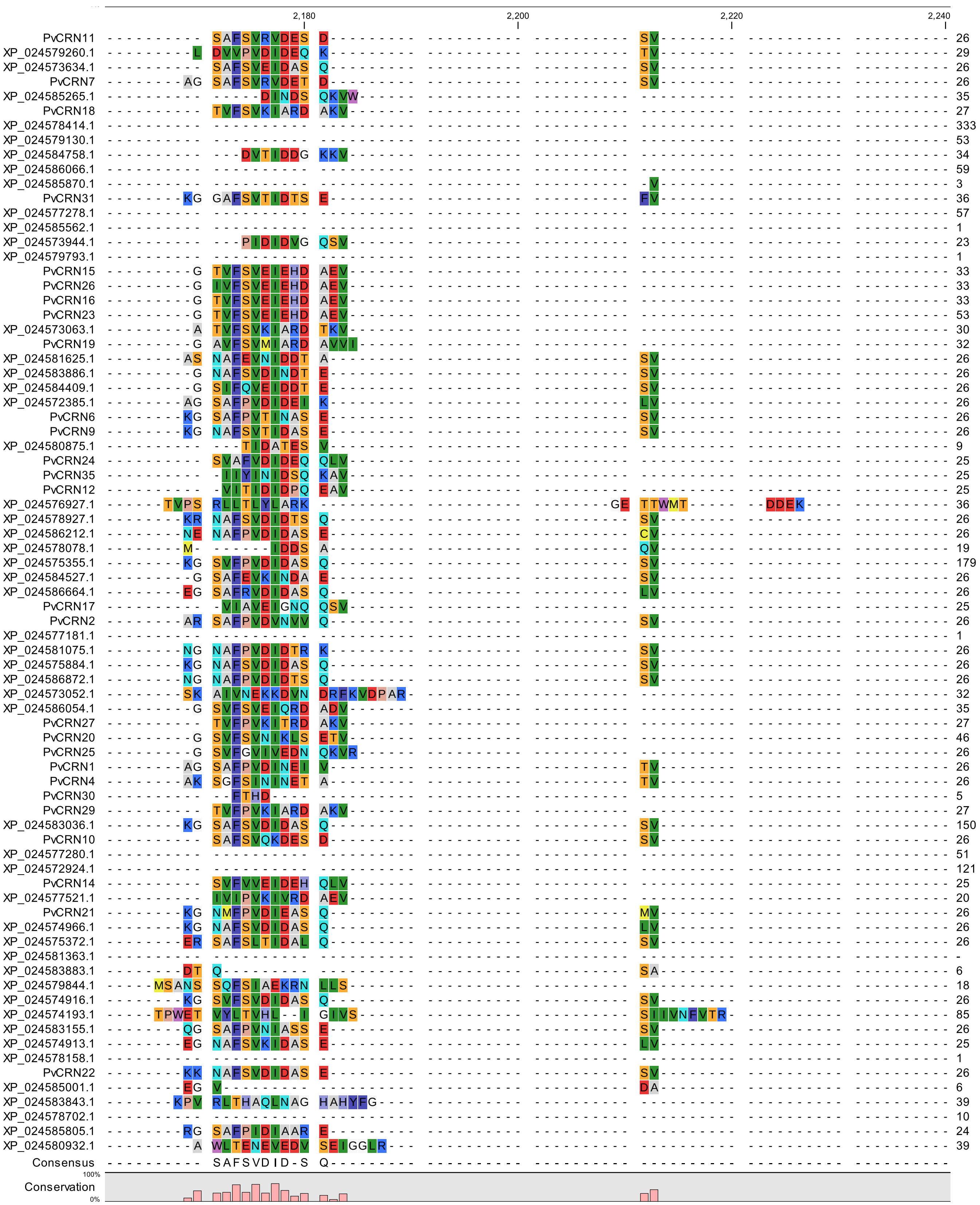
2,180
2,200
2,220
2,240
Consensus
Conservation
100%
0%

| | | |
|---|---|---|
| | 2,260   2,280   2,300   2,320 | |
| PvCRN11 | ---------- ---------- ---------- ---------- ---------- ---------- ---------- ---------- | 26 |
| XP_024579260.1 | ---------- ---------- ---------- ---------- ---------- ---------- ---------- ---------- | 29 |
| XP_024573634.1 | ---------- ---------- ---------- ---------- ---------- ---------- ---------- ---------- | 26 |
| PvCRN7 | ---------- ---------- ---------- ---------- ---------- ---------- ---------- ---------- | 26 |
| XP_024585265.1 | ---------- ---------- ---------- ---------- ---------- ---------- ---------- ---------- | 35 |
| PvCRN18 | ---------- ---------- ---------- ---------- ---------- ---------- ---------- ---------- | 27 |
| XP_024578414.1 | ---------- ---------- ---------- ---------- ---------- ---------- ---------- ---------- | 333 |
| XP_024579130.1 | ---------- ---------- ---------- ---------- ---------- ---------- ---------- ---------- | 53 |
| XP_024584758.1 | ---------- ---------- ---------- ---------- ---------- ---------- ---------- ---------- | 34 |
| XP_024586066.1 | ---------- ---------- ---------- ---------- ---------- ---------- ---------- ---------- | 59 |
| XP_024585870.1 | ---------- ---------- ---------- ---------- ---------- ---------- ---------- ---------- | 3 |
| PvCRN31 | ---------- ---------- ---------- ---------- ---------- ---------- ---------- ---------- | 36 |
| XP_024577278.1 | ---------- ---------- ---------- ---------- ---------- ---------- ---------- ---------- | 57 |
| XP_024585562.1 | ---------- ---------- ---------- ---------- ---------- ---------- ---------- ---------- | 1 |
| XP_024573944.1 | ---------- ---------- ---------- ---------- ---------- ---------- ---------- ---------- | 23 |
| XP_024579793.1 | ---------- ---------- ---------- ---------- ---------- ---------- ---------- ---------- | 1 |
| PvCRN15 | ---------- ---------- ---------- ---------- ---------- ---------- ---------- ---------- | 33 |
| PvCRN26 | ---------- ---------- ---------- ---------- ---------- ---------- ---------- ---------- | 33 |
| PvCRN16 | ---------- ---------- ---------- ---------- ---------- ---------- ---------- ---------- | 33 |
| PvCRN23 | ---------- ---------- ---------- ---------- ---------- ---------- ---------- ---------- | 53 |
| XP_024573063.1 | ---------- ---------- ---------- ---------- ---------- ---------- ---------- ---------- | 30 |
| PvCRN19 | ---------- ---------- ---------- ---------- ---------- ---------- ---------- ---------- | 32 |
| XP_024581625.1 | ---------- ---------- ---------- ---------- ---------- ---------- ---------- ---------- | 26 |
| XP_024583886.1 | ---------- ---------- ---------- ---------- ---------- ---------- ---------- ---------- | 26 |
| XP_024584409.1 | ---------- ---------- ---------- ---------- ---------- ---------- ---------- ---------- | 26 |
| XP_024572385.1 | ---------- ---------- ---------- ---------- ---------- ---------- ---------- ---------- | 26 |
| PvCRN6 | ---------- ---------- ---------- ---------- ---------- ---------- ---------- ---------- | 26 |
| PvCRN9 | ---------- ---------- ---------- ---------- ---------- ---------- ---------- ---------- | 26 |
| XP_024580875.1 | ---------- ---------- ---------- ---------- ---------- ---------- ---------- ---------- | 9 |
| PvCRN24 | ---------- ---------- ---------- ---------- ---------- ---------- ---------- ---------- | 25 |
| PvCRN35 | ---------- ---------- ---------- ---------- ---------- ---------- ---------- ---------- | 25 |
| PvCRN12 | ---------- ---------- ---------- ---------- ---------- ---------- ---------- ---------- | 25 |
| XP_024576927.1 | ---------- ---------- ---------- ---------- ---------- ---------- ---------- ---------- | 36 |
| XP_024578927.1 | ---------- ---------- ---------- ---------- ---------- ---------- ---------- ---------- | 26 |
| XP_024586212.1 | ---------- ---------- ---------- ---------- ---------- ---------- ---------- ---------- | 26 |
| XP_024578078.1 | ---------- ---------- ---------- ---------- ---------- ---------- ---------- ---------- | 19 |
| XP_024575355.1 | ---------- ---------- ---------- ---------- ---------- ---------- ---------- ---------- | 179 |
| XP_024584527.1 | ---------- ---------- ---------- ---------- ---------- ---------- ---------- ---------- | 26 |
| XP_024586664.1 | ---------- ---------- ---------- ---------- ---------- ---------- ---------- ---------- | 26 |
| PvCRN17 | ---------- ---------- ---------- ---------- ---------- ---------- ---------- ---------- | 25 |
| PvCRN2 | ---------- ---------- ---------- ---------- ---------- ---------- ---------- ---------- | 26 |
| XP_024577181.1 | ---------- ---------- ---------- ---------- ---------- ---------- ---------- ---------- | 1 |
| XP_024581075.1 | ---------- ---------- ---------- ---------- ---------- ---------- ---------- ---------- | 26 |
| XP_024575884.1 | ---------- ---------- ---------- ---------- ---------- ---------- ---------- ---------- | 26 |
| XP_024586872.1 | ---------- ---------- ---------- ---------- ---------- ---------- ---------- ---------- | 26 |
| XP_024573052.1 | ---------- ---------- ---------- ---------- ---------- ---------- ---------- ---------- | 32 |
| XP_024586054.1 | ---------- ---------- ---------- ---------- ---------- ---------- ---------- ---------- | 35 |
| PvCRN27 | ---------- ---------- ---------- ---------- ---------- ---------- ---------- ---------- | 27 |
| PvCRN20 | ---------- ---------- ---------- ---------- ---------- ---------- ---------- ---------- | 46 |
| PvCRN25 | ---------- ---------- ---------- ---------- ---------- ---------- ---------- ---------- | 26 |
| PvCRN1 | ---------- ---------- ---------- ---------- ---------- ---------- ---------- ---------- | 26 |
| PvCRN4 | ---------- ---------- ---------- ---------- ---------- ---------- ---------- ---------- | 26 |
| PvCRN30 | ---------- ---------- ---------- ---------- ---------- ---------- ---------- ---------- | 5 |
| PvCRN29 | ---------- ---------- ---------- ---------- ---------- ---------- ---------- ---------- | 27 |
| XP_024583036.1 | ---------- ---------- ---------- ---------- ---------- ---------- ---------- ---------- | 150 |
| PvCRN10 | ---------- ---------- ---------- ---------- ---------- ---------- ---------- ---------- | 26 |
| XP_024577280.1 | ---------- ---------- ---------- ---------- ---------- ---------- ---------- ---------- | 51 |
| XP_024572924.1 | ---------- ---------- ---------- ---------- ---------- ---------- ---------- ---------- | 121 |
| PvCRN14 | ---------- ---------- ---------- ---------- ---------- ---------- ---------- ---------- | 25 |
| XP_024577521.1 | ---------- ---------- ---------- ---------- ---------- ---------- ---------- ---------- | 20 |
| PvCRN21 | ---------- ---------- ---------- ---------- ---------- ---------- ---------- ---------- | 26 |
| XP_024574966.1 | ---------- ---------- ---------- ---------- ---------- ---------- ---------- ---------- | 26 |
| XP_024575372.1 | ---------- ---------- ---------- ---------- ---------- ---------- ---------- ---------- | 26 |
| XP_024581363.1 | ---------- ---------- ---------- ---------- ---------- ---------- ---------- ---------- | - |
| XP_024583883.1 | ---------- ---------- ---------- ---------- ---------- ---------- ---------- ---------- | 6 |
| XP_024579844.1 | ---------- ---------- ---------- ---------- ---------- ---------- ---------- ---------- | 18 |
| XP_024574916.1 | ---------- ---------- ---------- ---------- ---------- ---------- ---------- ---------- | 26 |
| XP_024574193.1 | ---------- ---------- ---------- ---------- ---------- ---------- ---------- ---------- | 85 |
| XP_024583155.1 | ---------- ---------- ---------- ---------- ---------- ---------- ---------- ---------- | 26 |
| XP_024574913.1 | ---------- ---------- ---------- ---------- ---------- ---------- ---------- ---------- | 25 |
| XP_024578158.1 | ---------- ---------- ---------- ---------- ---------- ---------- ---------- ---------- | 1 |
| PvCRN22 | ---------- ---------- ---------- ---------- ---------- ---------- ---------- ---------- | 26 |
| XP_024585001.1 | ---------- ---------- ---------- ---------- ---------- ---------- ---------- ---------- | 6 |
| XP_024583843.1 | ---------- ---------- ---------- ---------- ---------- ---------- ---------- ---------- | 39 |
| XP_024578702.1 | ---------- ---------- ---------- ---------- ---------- ---------- ---------- ---------- | 10 |
| XP_024585805.1 | ---------- ---------- ---------- ---------- ---------- ---------- ---------- ---------- | 24 |
| XP_024580932.1 | ---------- ---------- ---------- ---------- ---------- ---------- ---------- ---------- | 39 |
| Consensus | ---------- ---------- ---------- ---------- ---------- ---------- ---------- ---------- | |
| Conservation (100% / 0%) | | |

| | 2,340 · 2,360 · 2,380 · 2,400 | |
|---|---|---|
| PvCRN11 | ---------- ---------- ---------- ---------- ---------- ---------- ---------- ---------- | 26 |
| XP_024579260.1 | ---------- ---------- ---------- ---------- ---------- ---------- ---------- ---------- | 29 |
| XP_024573634.1 | ---------- ---------- ---------- ---------- ---------- ---------- ---------- ---------- | 26 |
| PvCRN7 | ---------- ---------- ---------- ---------- ---------- ---------- ---------- ---------- | 26 |
| XP_024585265.1 | ---------- ---------- ---------- ---------- ---------- ---------- ---------- ---------- | 35 |
| PvCRN18 | ---------- ---------- ---------- ---------- ---------- ---------- ---------- ---------- | 27 |
| XP_024578414.1 | ---------- ---------- ---------- ---------- ---------- ---------- ---------- ---------- | 333 |
| XP_024579130.1 | ---------- ---------- ---------- ---------- ---------- ---------- ---------- ---------- | 53 |
| XP_024584758.1 | ---------- ---------- ---------- ---------- ---------- ---------- ---------- ---------- | 34 |
| XP_024586066.1 | ---------- ---------- ---------- ---------- ---------- ---------- ---------- ---------- | 59 |
| XP_024585870.1 | ---------- ---------- ---------- ---------- ---------- ---------- ---------- ---------- | 3 |
| PvCRN31 | ---------- ---------- ---------- ---------- ---------- ---------- ---------- ---------- | 36 |
| XP_024577278.1 | ---------- ---------- ---------- ---------- ---------- ---------- ---------- ---------- | 57 |
| XP_024585562.1 | ---------- ---------- ---------- ---------- ---------- ---------- ---------- ---------- | 1 |
| XP_024573944.1 | ---------- ---------- ---------- ---------- ---------- ---------- ---------- ---------- | 23 |
| XP_024579793.1 | ---------- ---------- ---------- ---------- ---------- ---------- ---------- ---------- | 1 |
| PvCRN15 | ---------- ---------- ---------- ---------- ---------- ---------- ---------- ---------- | 33 |
| PvCRN26 | ---------- ---------- ---------- ---------- ---------- ---------- ---------- ---------- | 33 |
| PvCRN16 | ---------- ---------- ---------- ---------- ---------- ---------- ---------- ---------- | 33 |
| PvCRN23 | ---------- ---------- ---------- ---------- ---------- ---------- ---------- ---------- | 53 |
| XP_024573063.1 | ---------- ---------- ---------- ---------- ---------- ---------- ---------- ---------- | 30 |
| PvCRN19 | ---------- ---------- ---------- ---------- ---------- ---------- ---------- ---------- | 32 |
| XP_024581625.1 | ---------- ---------- ---------- ---------- ---------- ---------- ---------- ---------- | 26 |
| XP_024583886.1 | ---------- ---------- ---------- ---------- ---------- ---------- ---------- ---------- | 26 |
| XP_024584409.1 | ---------- ---------- ---------- ---------- ---------- ---------- ---------- ---------- | 26 |
| XP_024572385.1 | ---------- ---------- ---------- ---------- ---------- ---------- ---------- ---------- | 26 |
| PvCRN6 | ---------- ---------- ---------- ---------- ---------- ---------- ---------- ---------- | 26 |
| PvCRN9 | ---------- ---------- ---------- ---------- ---------- ---------- ---------- ---------- | 26 |
| XP_024580875.1 | ---------- ---------- ---------- ---------- ---------- ---------- ---------- ---------- | 9 |
| PvCRN24 | ---------- ---------- ---------- ---------- ---------- ---------- ---------- ---------- | 25 |
| PvCRN35 | ---------- ---------- ---------- ---------- ---------- ---------- ---------- ---------- | 25 |
| PvCRN12 | ---------- ---------- ---------- ---------- ---------- ---------- ---------- ---------- | 25 |
| XP_024576927.1 | ---------- ---------- ---------- ---------- ---------- ---------- ---------- ---------- | 36 |
| XP_024578927.1 | ---------- ---------- ---------- ---------- ---------- ---------- ---------- ---------- | 26 |
| XP_024586212.1 | ---------- ---------- ---------- ---------- ---------- ---------- ---------- ---------- | 26 |
| XP_024578078.1 | ---------- ---------- ---------- ---------- ---------- ---------- ---------- ---------- | 19 |
| XP_024575355.1 | ---------- ---------- ---------- ---------- ---------- ---------- ---------- ---------- | 179 |
| XP_024584527.1 | ---------- ---------- ---------- ---------- ---------- ---------- ---------- ---------- | 26 |
| XP_024586664.1 | ---------- ---------- ---------- ---------- ---------- ---------- ---------- ---------- | 26 |
| PvCRN17 | ---------- ---------- ---------- ---------- ---------- ---------- ---------- ---------- | 25 |
| PvCRN2 | ---------- ---------- ---------- ---------- ---------- ---------- ---------- ---------- | 26 |
| XP_024577181.1 | ---------- ---------- ---------- ---------- ---------- ---------- ---------- ---------- | 1 |
| XP_024581075.1 | ---------- ---------- ---------- ---------- ---------- ---------- ---------- ---------- | 26 |
| XP_024575884.1 | ---------- ---------- ---------- ---------- ---------- ---------- ---------- ---------- | 26 |
| XP_024586872.1 | ---------- ---------- ---------- ---------- ---------- ---------- ---------- ---------- | 26 |
| XP_024573052.1 | ---------- ---------- ---------- ---------- ---------- ---------- ---------- ---------- | 32 |
| XP_024586054.1 | ---------- ---------- ---------- ---------- ---------- ---------- ---------- ---------- | 35 |
| PvCRN27 | ---------- ---------- ---------- ---------- ---------- ---------- ---------- ---------- | 27 |
| PvCRN20 | ---------- ---------- ---------- ---------- ---------- ---------- ---------- ---------- | 46 |
| PvCRN25 | ---------- ---------- ---------- ---------- ---------- ---------- ---------- ---------- | 26 |
| PvCRN1 | ---------- ---------- ---------- ---------- ---------- ---------- ---------- ---------- | 26 |
| PvCRN4 | ---------- ---------- ---------- ---------- ---------- ---------- ---------- ---------- | 26 |
| PvCRN30 | ---------- ---------- ---------- ---------- ---------- ---------- ---------- ---------- | 5 |
| PvCRN29 | ---------- ---------- ---------- ---------- ---------- ---------- ---------- ---------- | 27 |
| XP_024583036.1 | ---------- ---------- ---------- ---------- ---------- ---------- ---------- ---------- | 150 |
| PvCRN10 | ---------- ---------- ---------- ---------- ---------- ---------- ---------- ---------- | 26 |
| XP_024577280.1 | ---------- ---------- ---------- ---------- ---------- ---------- ---------- ---------- | 51 |
| XP_024572924.1 | ---------- ---------- ---------- ---------- ---------- ---------- ---------- ---------- | 121 |
| PvCRN14 | ---------- ---------- ---------- ---------- ---------- ---------- ---------- ---------- | 25 |
| XP_024577521.1 | ---------- ---------- ---------- ---------- ---------- ---------- ---------- ---------- | 20 |
| PvCRN21 | ---------- ---------- ---------- ---------- ---------- ---------- ---------- ---------- | 26 |
| XP_024574966.1 | ---------- ---------- ---------- ---------- ---------- ---------- ---------- ---------- | 26 |
| XP_024575372.1 | ---------- ---------- ---------- ---------- ---------- ---------- ---------- ---------- | 26 |
| XP_024581363.1 | ---------- ---------- ---------- ---------- ---------- ---------- ---------- ---------- | - |
| XP_024583883.1 | ---------- ---------- ---------- ---------- ---------- ---------- ---------- ---------- | 6 |
| XP_024579844.1 | ---------- ---------- ---------- ---------- ---------- ---------- ---------- ---------- | 18 |
| XP_024574916.1 | ---------- ---------- ---------- ---------- ---------- ---------- ---------- ---------- | 26 |
| XP_024574193.1 | ---------- ---------- ---------- ---------- ---------- ---------- ---------- ---------- | 85 |
| XP_024583155.1 | ---------- ---------- ---------- ---------- ---------- ---------- ---------- ---------- | 26 |
| XP_024574913.1 | ---------- ---------- ---------- ---------- ---------- ---------- ---------- ---------- | 25 |
| XP_024578158.1 | ---------- ---------- ---------- ---------- ---------- ---------- ---------- ---------- | 1 |
| PvCRN22 | ---------- ---------- ---------- ---------- ---------- ---------- ---------- ---------- | 26 |
| XP_024585001.1 | ---------- ---------- ---------- ---------- ---------- ---------- ---------- ---------- | 6 |
| XP_024583843.1 | ---------- ---------- ---------- ---------- ---------- ---------- ---------- ---------- | 39 |
| XP_024578702.1 | ---------- ---------- ---------- ---------- ---------- ---------- ---------- ---------- | 10 |
| XP_024585805.1 | ---------- ---------- ---------- ---------- ---------- ---------- ---------- ---------S | 25 |
| XP_024580932.1 | ---------- ---------- ---------- ---------- ---------- ---------- ---------- ---------- | 39 |
| Consensus | ---------- ---------- ---------- ---------- ---------- ---------- ---------- ---------- | |
| Conservation (100% – 0%) | | |

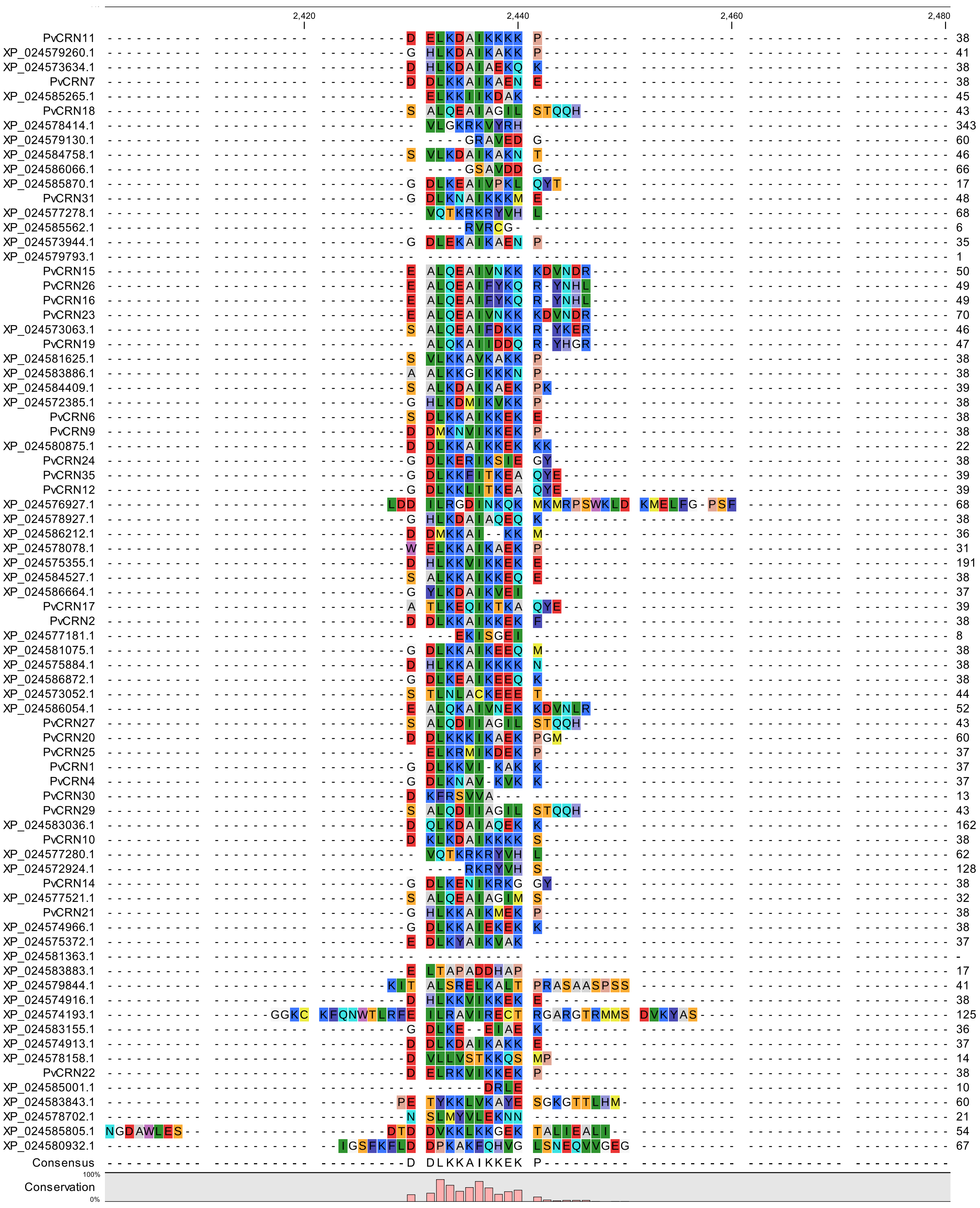

2,420
2,440
2,460
2,480
Consensus
Conservation
100%
0%

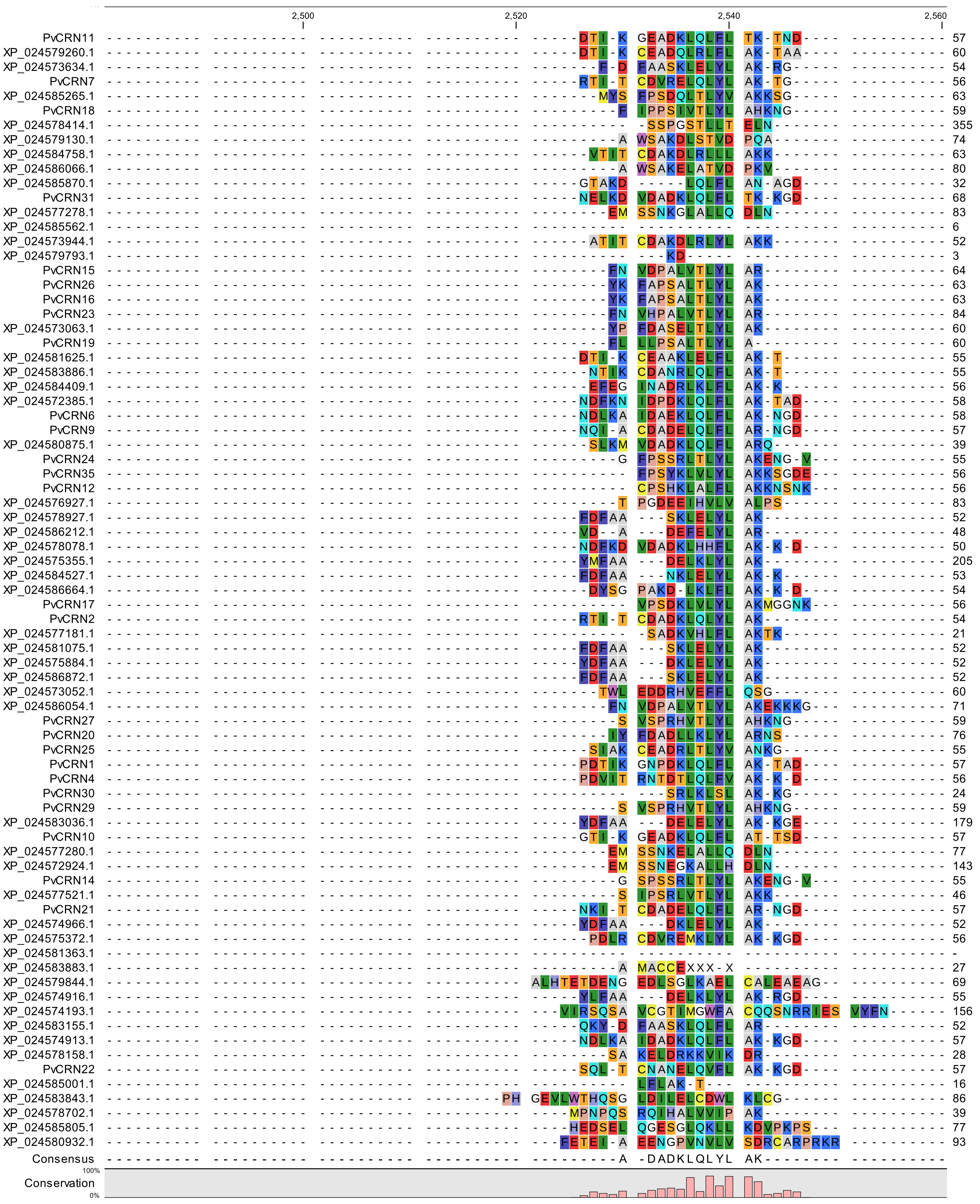

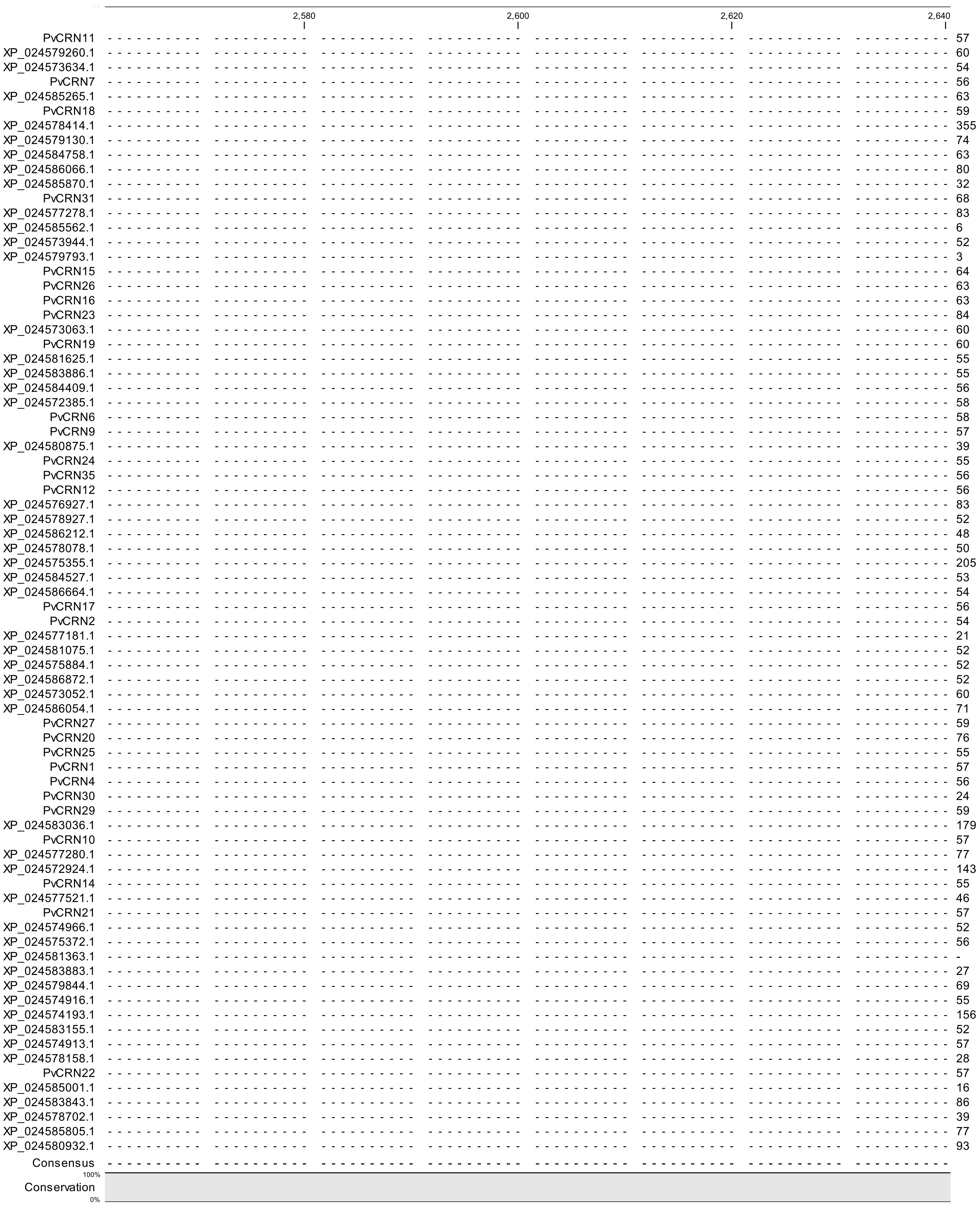
2,580
2,600
2,620
2,640
PvCRN11 57
XP_024579260.1 60
XP_024573634.1 54
PvCRN7 56
XP_024585265.1 63
PvCRN18 59
XP_024578414.1 355
XP_024579130.1 74
XP_024584758.1 63
XP_024586066.1 80
XP_024585870.1 32
PvCRN31 68
XP_024577278.1 83
XP_024585562.1 6
XP_024573944.1 52
XP_024579793.1 3
PvCRN15 64
PvCRN26 63
PvCRN16 63
PvCRN23 84
XP_024573063.1 60
PvCRN19 60
XP_024581625.1 55
XP_024583886.1 55
XP_024584409.1 56
XP_024572385.1 58
PvCRN6 58
PvCRN9 57
XP_024580875.1 39
PvCRN24 55
PvCRN35 56
PvCRN12 56
XP_024576927.1 83
XP_024578927.1 52
XP_024586212.1 48
XP_024578078.1 50
XP_024575355.1 205
XP_024584527.1 53
XP_024586664.1 54
PvCRN17 56
PvCRN2 54
XP_024577181.1 21
XP_024581075.1 52
XP_024575884.1 52
XP_024586872.1 52
XP_024573052.1 60
XP_024586054.1 71
PvCRN27 59
PvCRN20 76
PvCRN25 55
PvCRN1 57
PvCRN4 56
PvCRN30 24
PvCRN29 59
XP_024583036.1 179
PvCRN10 57
XP_024577280.1 77
XP_024572924.1 143
PvCRN14 55
XP_024577521.1 46
PvCRN21 57
XP_024574966.1 52
XP_024575372.1 56
XP_024581363.1 -
XP_024583883.1 27
XP_024579844.1 69
XP_024574916.1 55
XP_024574193.1 156
XP_024583155.1 52
XP_024574913.1 57
XP_024578158.1 28
PvCRN22 57
XP_024585001.1 16
XP_024583843.1 86
XP_024578702.1 39
XP_024585805.1 77
XP_024580932.1 93
Consensus
100%
Conservation
0%

| | 2,660 | 2,680 | 2,700 | 2,720 | |
|---|---|---|---|---|---|
| PvCRN11 | ---------- ---------- | ---------- ---------- | ---------- ---------- | ---------- ---------- | 57 |
| XP_024579260.1 | ---------- ---------- | ---------- ---------- | ---------- ---------- | ---------- ---------- | 60 |
| XP_024573634.1 | ---------- ---------- | ---------- ---------- | ---------- ---------- | ---------- ---------- | 54 |
| PvCRN7 | ---------- ---------- | ---------- ---------- | ---------- ---------- | ---------- ---------- | 56 |
| XP_024585265.1 | ---------- ---------- | ---------- ---------- | ---------- ---------- | ---------- ---------- | 63 |
| PvCRN18 | ---------- ---------- | ---------- ---------- | ---------- ---------- | ---------- ---------- | 59 |
| XP_024578414.1 | ---------- ---------- | ---------- ---------- | ---------- ---------- | ---------- ---------- | 355 |
| XP_024579130.1 | ---------- ---------- | ---------- ---------- | ---------- ---------- | ---------- ---------- | 74 |
| XP_024584758.1 | ---------- ---------- | ---------- ---------- | ---------- ---------- | ---------- ---------- | 63 |
| XP_024586066.1 | ---------- ---------- | ---------- ---------- | ---------- ---------- | ---------- ---------- | 80 |
| XP_024585870.1 | ---------- ---------- | ---------- ---------- | ---------- ---------- | ---------- ---------- | 32 |
| PvCRN31 | ---------- ---------- | ---------- ---------- | ---------- ---------- | ---------- ---------- | 68 |
| XP_024577278.1 | ---------- ---------- | ---------- ---------- | ---------- ---------- | ---------- ---------- | 83 |
| XP_024585562.1 | ---------- ---------- | ---------- ---------- | ---------- ---------- | ---------- ---------- | 6 |
| XP_024573944.1 | ---------- ---------- | ---------- ---------- | ---------- ---------- | ---------- ---------- | 52 |
| XP_024579793.1 | ---------- ---------- | ---------- ---------- | ---------- ---------- | ---------- ---------- | 3 |
| PvCRN15 | ---------- ---------- | ---------- ---------- | ---------- ---------- | ---------- ---------- | 64 |
| PvCRN26 | ---------- ---------- | ---------- ---------- | ---------- ---------- | ---------- ---------- | 63 |
| PvCRN16 | ---------- ---------- | ---------- ---------- | ---------- ---------- | ---------- ---------- | 63 |
| PvCRN23 | ---------- ---------- | ---------- ---------- | ---------- ---------- | ---------- ---------- | 84 |
| XP_024573063.1 | ---------- ---------- | ---------- ---------- | ---------- ---------- | ---------- ---------- | 60 |
| PvCRN19 | ---------- ---------- | ---------- ---------- | ---------- ---------- | ---------- ---------- | 60 |
| XP_024581625.1 | ---------- ---------- | ---------- ---------- | ---------- ---------- | ---------- ---------- | 55 |
| XP_024583886.1 | ---------- ---------- | ---------- ---------- | ---------- ---------- | ---------- ---------- | 55 |
| XP_024584409.1 | ---------- ---------- | ---------- ---------- | ---------- ---------- | ---------- ---------- | 56 |
| XP_024572385.1 | ---------- ---------- | ---------- ---------- | ---------- ---------- | ---------- ---------- | 58 |
| PvCRN6 | ---------- ---------- | ---------- ---------- | ---------- ---------- | ---------- ---------- | 58 |
| PvCRN9 | ---------- ---------- | ---------- ---------- | ---------- ---------- | ---------- ---------- | 57 |
| XP_024580875.1 | ---------- ---------- | ---------- ---------- | ---------- ---------- | ---------- ---------- | 39 |
| PvCRN24 | ---------- ---------- | ---------- ---------- | ---------- ---------- | ---------- ---------- | 55 |
| PvCRN35 | ---------- ---------- | ---------- ---------- | ---------- ---------- | ---------- ---------- | 56 |
| PvCRN12 | ---------- ---------- | ---------- ---------- | ---------- ---------- | ---------- ---------- | 56 |
| XP_024576927.1 | ---------- ---------- | ---------- ---------- | ---------- ---------- | ---------- ---------- | 83 |
| XP_024578927.1 | ---------- ---------- | ---------- ---------- | ---------- ---------- | ---------- ---------- | 52 |
| XP_024586212.1 | ---------- ---------- | ---------- ---------- | ---------- ---------- | ---------- ---------- | 48 |
| XP_024578078.1 | ---------- ---------- | ---------- ---------- | ---------- ---------- | ---------- ---------- | 50 |
| XP_024575355.1 | ---------- ---------- | ---------- ---------- | ---------- ---------- | ---------- ---------- | 205 |
| XP_024584527.1 | ---------- ---------- | ---------- ---------- | ---------- ---------- | ---------- ---------- | 53 |
| XP_024586664.1 | ---------- ---------- | ---------- ---------- | ---------- ---------- | ---------- ---------- | 54 |
| PvCRN17 | ---------- ---------- | ---------- ---------- | ---------- ---------- | ---------- ---------- | 56 |
| PvCRN2 | ---------- ---------- | ---------- ---------- | ---------- ---------- | ---------- ---------- | 54 |
| XP_024577181.1 | ---------- ---------- | ---------- ---------- | ---------- ---------- | ---------- ---------- | 21 |
| XP_024581075.1 | ---------- ---------- | ---------- ---------- | ---------- ---------- | ---------- ---------- | 52 |
| XP_024575884.1 | ---------- ---------- | ---------- ---------- | ---------- ---------- | ---------- ---------- | 52 |
| XP_024586872.1 | ---------- ---------- | ---------- ---------- | ---------- ---------- | ---------- ---------- | 52 |
| XP_024573052.1 | ---------- ---------- | ---------- ---------- | ---------- ---------- | ---------- ---------- | 60 |
| XP_024586054.1 | ---------- ---------- | ---------- ---------- | ---------- ---------- | ---------- ---------- | 71 |
| PvCRN27 | ---------- ---------- | ---------- ---------- | ---------- ---------- | ---------- ---------- | 59 |
| PvCRN20 | ---------- ---------- | ---------- ---------- | ---------- ---------- | ---------- ---------- | 76 |
| PvCRN25 | ---------- ---------- | ---------- ---------- | ---------- ---------- | ---------- ---------- | 55 |
| PvCRN1 | ---------- ---------- | ---------- ---------- | ---------- ---------- | ---------- ---------- | 57 |
| PvCRN4 | ---------- ---------- | ---------- ---------- | ---------- ---------- | ---------- ---------- | 56 |
| PvCRN30 | ---------- ---------- | ---------- ---------- | ---------- ---------- | ---------- ---------- | 24 |
| PvCRN29 | ---------- ---------- | ---------- ---------- | ---------- ---------- | ---------- ---------- | 59 |
| XP_024583036.1 | ---------- ---------- | ---------- ---------- | ---------- ---------- | ---------- ---------- | 179 |
| PvCRN10 | ---------- ---------- | ---------- ---------- | ---------- ---------- | ---------- ---------- | 57 |
| XP_024577280.1 | ---------- ---------- | ---------- ---------- | ---------- ---------- | ---------- ---------- | 77 |
| XP_024572924.1 | ---------- ---------- | ---------- ---------- | ---------- ---------- | ---------- ---------- | 143 |
| PvCRN14 | ---------- ---------- | ---------- ---------- | ---------- ---------- | ---------- ---------- | 55 |
| XP_024577521.1 | ---------- ---------- | ---------- ---------- | ---------- ---------- | ---------- ---------- | 46 |
| PvCRN21 | ---------- ---------- | ---------- ---------- | ---------- ---------- | ---------- ---------- | 57 |
| XP_024574966.1 | ---------- ---------- | ---------- ---------- | ---------- ---------- | ---------- ---------- | 52 |
| XP_024575372.1 | ---------- ---------- | ---------- ---------- | ---------- ---------- | ---------- ---------- | 56 |
| XP_024581363.1 | ---------- ---------- | ---------- ---------- | ---------- ---------- | ---------- ---------- | - |
| XP_024583883.1 | ---------- ---------- | ---------- ---------- | ---------- ---------- | ---------- ---------- | 27 |
| XP_024579844.1 | ---------- ---------- | ---------- ---------- | ---------- ---------- | ---------- ---------- | 69 |
| XP_024574916.1 | ---------- ---------- | ---------- ---------- | ---------- ---------- | ---------- ---------- | 55 |
| XP_024574193.1 | ---------- ---------- | ---------- ---------- | ---------- ---------- | ---------- ---------- | 156 |
| XP_024583155.1 | ---------- ---------- | ---------- ---------- | ---------- ---------- | ---------- ---------- | 52 |
| XP_024574913.1 | ---------- ---------- | ---------- ---------- | ---------- ---------- | ---------- ---------- | 57 |
| XP_024578158.1 | ---------- ---------- | ---------- ---------- | ---------- ---------- | ---------- ---------- | 28 |
| PvCRN22 | ---------- ---------- | ---------- ---------- | ---------- ---------- | ---------- ---------- | 57 |
| XP_024585001.1 | ---------- ---------- | ---------- ---------- | ---------- ---------- | ---------- ---------- | 16 |
| XP_024583843.1 | ---------- ---------- | ---------- ---------- | ---------- ---------- | ---------- ---------- | 86 |
| XP_024578702.1 | ---------- ---------- | ---------- ---------- | ---------- ---------- | ---------- ---------- | 39 |
| XP_024585805.1 | ---------- ---------- | ---------- ---------- | ---------- ---------- | ---------- ---------- | 77 |
| XP_024580932.1 | ---------- ---------- | ---------- ---------- | ---------- ---------- | ---------- ---------- | 93 |
| Consensus | ---------- ---------- | ---------- ---------- | ---------- ---------- | ---------- ---------- | |

100%
Conservation
0%

| | 2,740 · 2,760 · 2,780 · 2,800 | |
|---|---|---|
| PvCRN11 | ---------- ---------- ---------- ---------- ---------- ---------- ---------- ---------- | 57 |
| XP_024579260.1 | ---------- ---------- ---------- ---------- ---------- ---------- ---------- ---------- | 60 |
| XP_024573634.1 | ---------- ---------- ---------- ---------- ---------- ---------- ---------- ---------- | 54 |
| PvCRN7 | ---------- ---------- ---------- ---------- ---------- ---------- ---------- ---------- | 56 |
| XP_024585265.1 | ---------- ---------- ---------- ---------- ---------- ---------- ---------- ---------- | 63 |
| PvCRN18 | ---------- ---------- ---------- ---------- ---------- ---------- ---------- ---------- | 59 |
| XP_024578414.1 | ---------- ---------- ---------- ---------- ---------- ---------- ---------- ---------- | 355 |
| XP_024579130.1 | ---------- ---------- ---------- ---------- ---------- ---------- ---------- ---------- | 74 |
| XP_024584758.1 | ---------- ---------- ---------- ---------- ---------- ---------- ---------- ---------- | 63 |
| XP_024586066.1 | ---------- ---------- ---------- ---------- ---------- ---------- ---------- ---------- | 80 |
| XP_024585870.1 | ---------- ---------- ---------- ---------- ---------- ---------- ---------- ---------- | 32 |
| PvCRN31 | ---------- ---------- ---------- ---------- ---------- ---------- ---------- ---------- | 68 |
| XP_024577278.1 | ---------- ---------- ---------- ---------- ---------- ---------- ---------- ---------- | 83 |
| XP_024585562.1 | ---------- ---------- ---------- ---------- ---------- ---------- ---------- ---------- | 6 |
| XP_024573944.1 | ---------- ---------- ---------- ---------- ---------- ---------- ---------- ---------- | 52 |
| XP_024579793.1 | ---------- ---------- ---------- ---------- ---------- ---------- ---------- ---------- | 3 |
| PvCRN15 | ---------- ---------- ---------- ---------- ---------- ---------- ---------- ---------- | 64 |
| PvCRN26 | ---------- ---------- ---------- ---------- ---------- ---------- ---------- ---------- | 63 |
| PvCRN16 | ---------- ---------- ---------- ---------- ---------- ---------- ---------- ---------- | 63 |
| PvCRN23 | ---------- ---------- ---------- ---------- ---------- ---------- ---------- ---------- | 84 |
| XP_024573063.1 | ---------- ---------- ---------- ---------- ---------- ---------- ---------- ---------- | 60 |
| PvCRN19 | ---------- ---------- ---------- ---------- ---------- ---------- ---------- ---------- | 60 |
| XP_024581625.1 | ---------- ---------- ---------- ---------- ---------- ---------- ---------- ---------- | 55 |
| XP_024583886.1 | ---------- ---------- ---------- ---------- ---------- ---------- ---------- ---------- | 55 |
| XP_024584409.1 | ---------- ---------- ---------- ---------- ---------- ---------- ---------- ---------- | 56 |
| XP_024572385.1 | ---------- ---------- ---------- ---------- ---------- ---------- ---------- ---------- | 58 |
| PvCRN6 | ---------- ---------- ---------- ---------- ---------- ---------- ---------- ---------- | 58 |
| PvCRN9 | ---------- ---------- ---------- ---------- ---------- ---------- ---------- ---------- | 57 |
| XP_024580875.1 | ---------- ---------- ---------- ---------- ---------- ---------- ---------- ---------- | 39 |
| PvCRN24 | ---------- ---------- ---------- ---------- ---------- ---------- ---------- ---------- | 55 |
| PvCRN35 | ---------- ---------- ---------- ---------- ---------- ---------- ---------- ---------- | 56 |
| PvCRN12 | ---------- ---------- ---------- ---------- ---------- ---------- ---------- ---------- | 56 |
| XP_024576927.1 | ---------- ---------- ---------- ---------- ---------- ---------- ---------- ---------- | 83 |
| XP_024578927.1 | ---------- ---------- ---------- ---------- ---------- ---------- ---------- ---------- | 52 |
| XP_024586212.1 | ---------- ---------- ---------- ---------- ---------- ---------- ---------- ---------- | 48 |
| XP_024578078.1 | ---------- ---------- ---------- ---------- ---------- ---------- ---------- ---------- | 50 |
| XP_024575355.1 | ---------- ---------- ---------- ---------- ---------- ---------- ---------- ---------- | 205 |
| XP_024584527.1 | ---------- ---------- ---------- ---------- ---------- ---------- ---------- ---------- | 53 |
| XP_024586664.1 | ---------- ---------- ---------- ---------- ---------- ---------- ---------- ---------- | 54 |
| PvCRN17 | ---------- ---------- ---------- ---------- ---------- ---------- ---------- ---------- | 56 |
| PvCRN2 | ---------- ---------- ---------- ---------- ---------- ---------- ---------- ---------- | 54 |
| XP_024577181.1 | ---------- ---------- ---------- ---------- ---------- ---------- ---------- ---------- | 21 |
| XP_024581075.1 | ---------- ---------- ---------- ---------- ---------- ---------- ---------- ---------- | 52 |
| XP_024575884.1 | ---------- ---------- ---------- ---------- ---------- ---------- ---------- ---------- | 52 |
| XP_024586872.1 | ---------- ---------- ---------- ---------- ---------- ---------- ---------- ---------- | 52 |
| XP_024573052.1 | ---------- ---------- ---------- ---------- ---------- ---------- ---------- ---------- | 60 |
| XP_024586054.1 | ---------- ---------- ---------- ---------- ---------- ---------- ---------- ---------- | 71 |
| PvCRN27 | ---------- ---------- ---------- ---------- ---------- ---------- ---------- ---------- | 59 |
| PvCRN20 | ---------- ---------- ---------- ---------- ---------- ---------- ---------- ---------- | 76 |
| PvCRN25 | ---------- ---------- ---------- ---------- ---------- ---------- ---------- ---------- | 55 |
| PvCRN1 | ---------- ---------- ---------- ---------- ---------- ---------- ---------- ---------- | 57 |
| PvCRN4 | ---------- ---------- ---------- ---------- ---------- ---------- ---------- ---------- | 56 |
| PvCRN30 | ---------- ---------- ---------- ---------- ---------- ---------- ---------- ---------- | 24 |
| PvCRN29 | ---------- ---------- ---------- ---------- ---------- ---------- ---------- ---------- | 59 |
| XP_024583036.1 | ---------- ---------- ---------- ---------- ---------- ---------- ---------- ---------- | 179 |
| PvCRN10 | ---------- ---------- ---------- ---------- ---------- ---------- ---------- ---------- | 57 |
| XP_024577280.1 | ---------- ---------- ---------- ---------- ---------- ---------- ---------- ---------- | 77 |
| XP_024572924.1 | ---------- ---------- ---------- ---------- ---------- ---------- ---------- ---------- | 143 |
| PvCRN14 | ---------- ---------- ---------- ---------- ---------- ---------- ---------- ---------- | 55 |
| XP_024577521.1 | ---------- ---------- ---------- ---------- ---------- ---------- ---------- ---------- | 46 |
| PvCRN21 | ---------- ---------- ---------- ---------- ---------- ---------- ---------- ---------- | 57 |
| XP_024574966.1 | ---------- ---------- ---------- ---------- ---------- ---------- ---------- ---------- | 52 |
| XP_024575372.1 | ---------- ---------- ---------- ---------- ---------- ---------- ---------- ---------- | 56 |
| XP_024581363.1 | ---------- ---------- ---------- ---------- ---------- ---------- ---------- ---------- | - |
| XP_024583883.1 | ---------- ---------- ---------- ---------- ---------- ---------- ---------- ---------- | 27 |
| XP_024579844.1 | ---------- ---------- ---------- ---------- ---------- ---------- ---------- ---------- | 69 |
| XP_024574916.1 | ---------- ---------- ---------- ---------- ---------- ---------- ---------- ---------- | 55 |
| XP_024574193.1 | ---------- ---------- ---------- ---------- ---------- ---------- ---------- ---------- | 156 |
| XP_024583155.1 | ---------- ---------- ---------- ---------- ---------- ---------- ---------- ---------- | 52 |
| XP_024574913.1 | ---------- ---------- ---------- ---------- ---------- ---------- ---------- ---------- | 57 |
| XP_024578158.1 | ---------- ---------- ---------- ---------- ---------- ---------- ---------- ---------- | 28 |
| PvCRN22 | ---------- ---------- ---------- ---------- ---------- ---------- ---------- ---------- | 57 |
| XP_024585001.1 | ---------- ---------- ---------- ---------- ---------- ---------- ---------- ---------- | 16 |
| XP_024583843.1 | ---------- ---------- ---------- ---------- ---------- ---------- ---------- ---------- | 86 |
| XP_024578702.1 | ---------- ---------- ---------- ---------- ---------- ---------- ---------- ---------- | 39 |
| XP_024585805.1 | ---------- ---------- ---------- ---------- ---------- ---------- ---------- ---------- | 77 |
| XP_024580932.1 | ---------- ---------- ---------- ---------- ---------- ---------- ---------- ---------- | 93 |
| Consensus | ---------- ---------- ---------- ---------- ---------- ---------- ---------- ---------- | |
| Conservation (100% / 0%) | | |

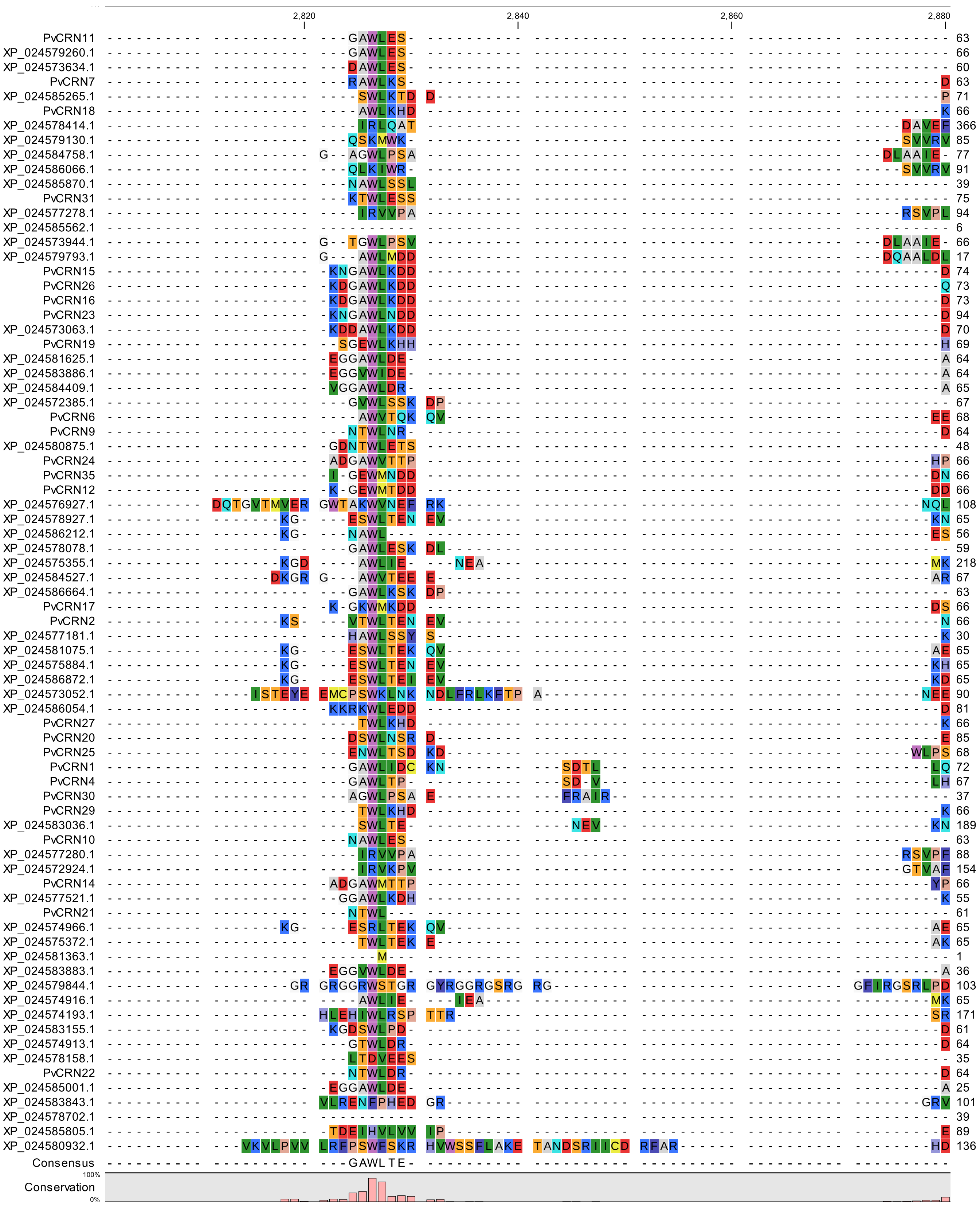

2,820
2,840
2,860
2,880
PvCRN11
XP_024579260.1
XP_024573634.1
PvCRN7
XP_024585265.1
PvCRN18
XP_024578414.1
XP_024579130.1
XP_024584758.1
XP_024586066.1
XP_024585870.1
PvCRN31
XP_024577278.1
XP_024585562.1
XP_024573944.1
XP_024579793.1
PvCRN15
PvCRN26
PvCRN16
PvCRN23
XP_024573063.1
PvCRN19
XP_024581625.1
XP_024583886.1
XP_024584409.1
XP_024572385.1
PvCRN6
PvCRN9
XP_024580875.1
PvCRN24
PvCRN35
PvCRN12
XP_024576927.1
XP_024578927.1
XP_024586212.1
XP_024578078.1
XP_024575355.1
XP_024584527.1
XP_024586664.1
PvCRN17
PvCRN2
XP_024577181.1
XP_024581075.1
XP_024575884.1
XP_024586872.1
XP_024573052.1
XP_024586054.1
PvCRN27
PvCRN20
PvCRN25
PvCRN1
PvCRN4
PvCRN30
PvCRN29
XP_024583036.1
PvCRN10
XP_024577280.1
XP_024572924.1
PvCRN14
XP_024577521.1
PvCRN21
XP_024574966.1
XP_024575372.1
XP_024581363.1
XP_024583883.1
XP_024579844.1
XP_024574916.1
XP_024574193.1
XP_024583155.1
XP_024574913.1
XP_024578158.1
PvCRN22
XP_024585001.1
XP_024583843.1
XP_024578702.1
XP_024585805.1
XP_024580932.1
Consensus
Conservation
100%
0%

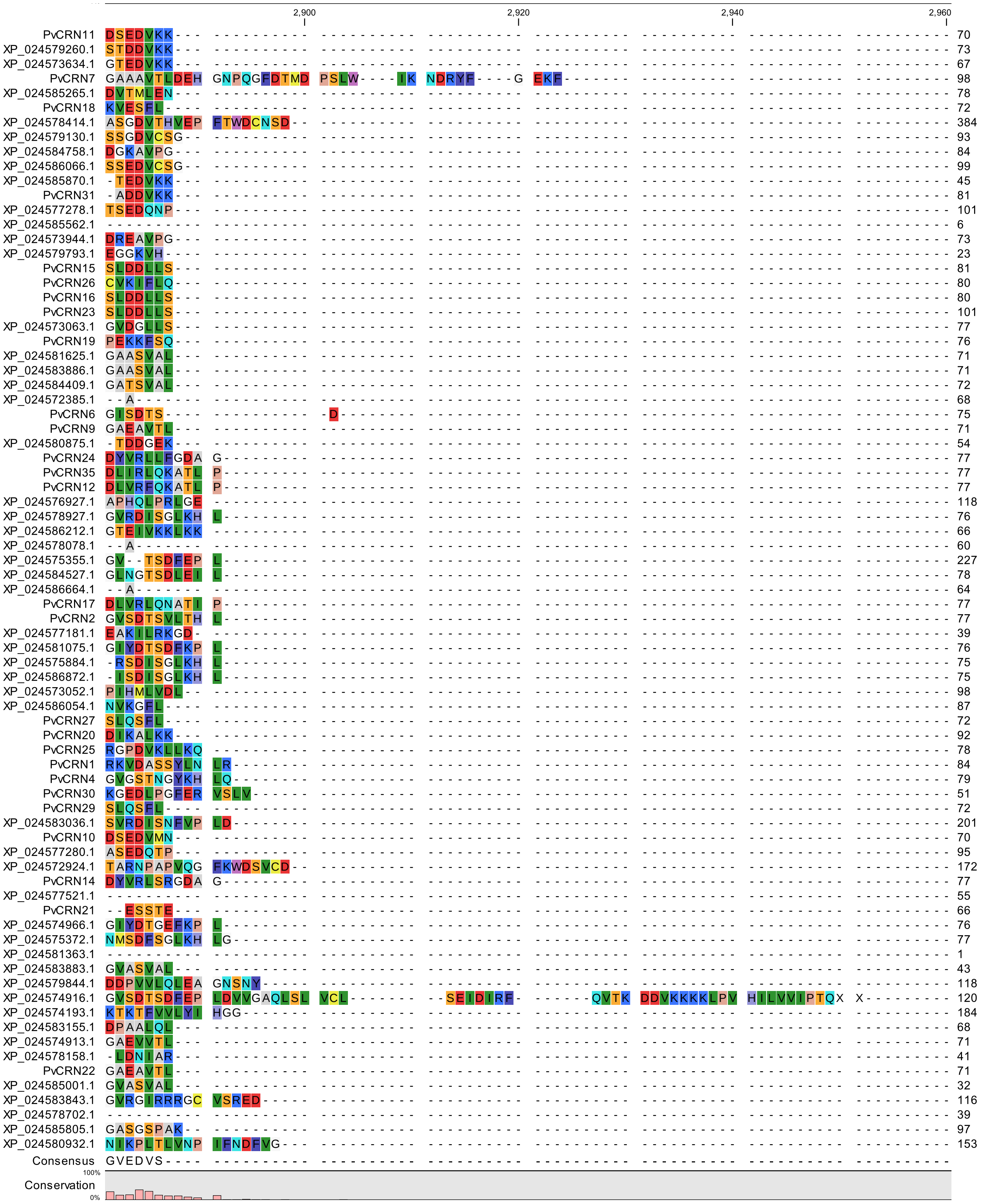
2,900
2,920
2,940
2,960
PvCRN11 DSEDVKK 70
XP_024579260.1 STDDVKK 73
XP_024573634.1 GTEDVKK 67
PvCRN7 GAAAVTLDEH GNPQGFDTMD PSLW IK NDRYF G EKF 98
XP_024585265.1 DVTMLEN 78
PvCRN18 KVESFL 72
XP_024578414.1 ASGDVTHVEP FTWDCNSD 384
XP_024579130.1 SSGDVCSG 93
XP_024584758.1 DGKAVPG 84
XP_024586066.1 SSEDVCSG 99
XP_024585870.1 TEDVKK 45
PvCRN31 ADDVKK 81
XP_024577278.1 TSEDQNP 101
XP_024585562.1 6
XP_024573944.1 DREAVPG 73
XP_024579793.1 EGGKVH 23
PvCRN15 SLDDLLS 81
PvCRN26 CVKIFLQ 80
PvCRN16 SLDDLLS 80
PvCRN23 SLDDLLS 101
XP_024573063.1 GVDGLLS 77
PvCRN19 PEKKFSQ 76
XP_024581625.1 GAASVAL 71
XP_024583886.1 GAASVAL 71
XP_024584409.1 GATSVAL 72
XP_024572385.1 A 68
PvCRN6 GISDTS D 75
PvCRN9 GAEAVTL 71
XP_024580875.1 TDDGEK 54
PvCRN24 DYVRLLFGDA G 77
PvCRN35 DLIRLQKATL P 77
PvCRN12 DLVRFQKATL P 77
XP_024576927.1 APHQLPRLGE 118
XP_024578927.1 GVRDISGLKH L 76
XP_024586212.1 GTEIVKKLKK 66
XP_024578078.1 A 60
XP_024575355.1 GV TSDFEP L 227
XP_024584527.1 GLNGTSDLEI L 78
XP_024586664.1 A 64
PvCRN17 DLVRLQNATI P 77
PvCRN2 GVSDTSVLTH L 77
XP_024577181.1 EAKILRKGD 39
XP_024581075.1 GIYDTSDFKP L 76
XP_024575884.1 RSDISGLKH L 75
XP_024586872.1 ISDISGLKH L 75
XP_024573052.1 PIHMLVDL 98
XP_024586054.1 NVKGFL 87
PvCRN27 SLQSFL 72
PvCRN20 DIKALKK 92
PvCRN25 RGPDVKLLKQ 78
PvCRN1 RKVDASSYLN LR 84
PvCRN4 GVGSTNGYKH LQ 79
PvCRN30 KGEDLPGFER VSLV 51
PvCRN29 SLQSFL 72
XP_024583036.1 SVRDISNFVP LD 201
PvCRN10 DSEDVMN 70
XP_024577280.1 ASEDQTP 95
XP_024572924.1 TARNPAPVQG FKWDSVCD 172
PvCRN14 DYVRLSRGDA G 77
XP_024577521.1 55
PvCRN21 ESSTE 66
XP_024574966.1 GIYDTGEFKP L 76
XP_024575372.1 NMSDFSGLKH LG 77
XP_024581363.1 1
XP_024583883.1 GVASVAL 43
XP_024579844.1 DDPVVLQLEA GNSNY 118
XP_024574916.1 GVSDTSDFEP LDVVGAQLSL VCL SEIDIRF QVTK DDVKKKKLPV HILVVIPTQX X 120
XP_024574193.1 KTKTFVVLYI HGG 184
XP_024583155.1 DPAALQL 68
XP_024574913.1 GAEVVTL 71
XP_024578158.1 LDNIAR 41
PvCRN22 GAEAVTL 71
XP_024585001.1 GVASVAL 32
XP_024583843.1 GVRGIRRRGC VSRED 116
XP_024578702.1 39
XP_024585805.1 GASGSPAK 97
XP_024580932.1 NIKPLTLVNP IFNDFVG 153
Consensus GVEDVS
100%
Conservation
0%

| | 2,980 · 3,000 · 3,020 · 3,040 | |
|---|---|---|
| PvCRN11 | ---------- ---------- ---------- ---------- ---------- ---------- ---------- ---------- | 70 |
| XP_024579260.1 | ---------- ---------- ---------- ---------- ---------- ---------- ---------- ---------- | 73 |
| XP_024573634.1 | ---------- ---------- ---------- ---------- ---------- ---------- ---------- ---------- | 67 |
| PvCRN7 | ---------- ---------- ---------- ---------- ---------- ---------- ---------- ---------- | 98 |
| XP_024585265.1 | ---------- ---------- ---------- ---------- ---------- ---------- ---------- ---------- | 78 |
| PvCRN18 | ---------- ---------- ---------- ---------- ---------- ---------- ---------- ---------- | 72 |
| XP_024578414.1 | ---------- ---------- ---------- ---------- ---------- ---------- ---------- ---------- | 384 |
| XP_024579130.1 | ---------- ---------- ---------- ---------- ---------- ---------- ---------- ---------- | 93 |
| XP_024584758.1 | ---------- ---------- ---------- ---------- ---------- ---------- ---------- ---------- | 84 |
| XP_024586066.1 | ---------- ---------- ---------- ---------- ---------- ---------- ---------- ---------- | 99 |
| XP_024585870.1 | ---------- ---------- ---------- ---------- ---------- ---------- ---------- ---------- | 45 |
| PvCRN31 | ---------- ---------- ---------- ---------- ---------- ---------- ---------- ---------- | 81 |
| XP_024577278.1 | ---------- ---------- ---------- ---------- ---------- ---------- ---------- ---------- | 101 |
| XP_024585562.1 | ---------- ---------- ---------- ---------- ---------- ---------- ---------- ---------- | 6 |
| XP_024573944.1 | ---------- ---------- ---------- ---------- ---------- ---------- ---------- ---------- | 73 |
| XP_024579793.1 | ---------- ---------- ---------- ---------- ---------- ---------- ---------- ---------- | 23 |
| PvCRN15 | ---------- ---------- ---------- ---------- ---------- ---------- ---------- ---------- | 81 |
| PvCRN26 | ---------- ---------- ---------- ---------- ---------- ---------- ---------- ---------- | 80 |
| PvCRN16 | ---------- ---------- ---------- ---------- ---------- ---------- ---------- ---------- | 80 |
| PvCRN23 | ---------- ---------- ---------- ---------- ---------- ---------- ---------- ---------- | 101 |
| XP_024573063.1 | ---------- ---------- ---------- ---------- ---------- ---------- ---------- ---------- | 77 |
| PvCRN19 | ---------- ---------- ---------- ---------- ---------- ---------- ---------- ---------- | 76 |
| XP_024581625.1 | ---------- ---------- ---------- ---------- ---------- ---------- ---------- ---------- | 71 |
| XP_024583886.1 | ---------- ---------- ---------- ---------- ---------- ---------- ---------- ---------- | 71 |
| XP_024584409.1 | ---------- ---------- ---------- ---------- ---------- ---------- ---------- ---------- | 72 |
| XP_024572385.1 | ---------- ---------- ---------- ---------- ---------- ---------- ---------- ---------- | 68 |
| PvCRN6 | ---------- ---------- ---------- ---------- ---------- ---------- ---------- ---------- | 75 |
| PvCRN9 | ---------- ---------- ---------- ---------- ---------- ---------- ---------- ---------- | 71 |
| XP_024580875.1 | ---------- ---------- ---------- ---------- ---------- ---------- ---------- ---------- | 54 |
| PvCRN24 | ---------- ---------- ---------- ---------- ---------- ---------- ---------- ---------- | 77 |
| PvCRN35 | ---------- ---------- ---------- ---------- ---------- ---------- ---------- ---------- | 77 |
| PvCRN12 | ---------- ---------- ---------- ---------- ---------- ---------- ---------- ---------- | 77 |
| XP_024576927.1 | ---------- ---------- ---------- ---------- ---------- ---------- ---------- ---------- | 118 |
| XP_024578927.1 | ---------- ---------- ---------- ---------- ---------- ---------- ---------- ---------- | 76 |
| XP_024586212.1 | ---------- ---------- ---------- ---------- ---------- ---------- ---------- ---------- | 66 |
| XP_024578078.1 | ---------- ---------- ---------- ---------- ---------- ---------- ---------- ---------- | 60 |
| XP_024575355.1 | ---------- ---------- ---------- ---------- ---------- ---------- ---------- ---------- | 227 |
| XP_024584527.1 | ---------- ---------- ---------- ---------- ---------- ---------- ---------- ---------- | 78 |
| XP_024586664.1 | ---------- ---------- ---------- ---------- ---------- ---------- ---------- ---------- | 64 |
| PvCRN17 | ---------- ---------- ---------- ---------- ---------- ---------- ---------- ---------- | 77 |
| PvCRN2 | ---------- ---------- ---------- ---------- ---------- ---------- ---------- ---------- | 77 |
| XP_024577181.1 | ---------- ---------- ---------- ---------- ---------- ---------- ---------- ---------- | 39 |
| XP_024581075.1 | ---------- ---------- ---------- ---------- ---------- ---------- ---------- ---------- | 76 |
| XP_024575884.1 | ---------- ---------- ---------- ---------- ---------- ---------- ---------- ---------- | 75 |
| XP_024586872.1 | ---------- ---------- ---------- ---------- ---------- ---------- ---------- ---------- | 75 |
| XP_024573052.1 | ---------- ---------- ---------- ---------- ---------- ---------- ---------- ---------- | 98 |
| XP_024586054.1 | ---------- ---------- ---------- ---------- ---------- ---------- ---------- ---------- | 87 |
| PvCRN27 | ---------- ---------- ---------- ---------- ---------- ---------- ---------- ---------- | 72 |
| PvCRN20 | ---------- ---------- ---------- ---------- ---------- ---------- ---------- ---------- | 92 |
| PvCRN25 | ---------- ---------- ---------- ---------- ---------- ---------- ---------- ---------- | 78 |
| PvCRN1 | ---------- ---------- ---------- ---------- ---------- ---------- ---------- ---------- | 84 |
| PvCRN4 | ---------- ---------- ---------- ---------- ---------- ---------- ---------- ---------- | 79 |
| PvCRN30 | ---------- ---------- ---------- ---------- ---------- ---------- ---------- ---------- | 51 |
| PvCRN29 | ---------- ---------- ---------- ---------- ---------- ---------- ---------- ---------- | 72 |
| XP_024583036.1 | ---------- ---------- ---------- ---------- ---------- ---------- ---------- ---------- | 201 |
| PvCRN10 | ---------- ---------- ---------- ---------- ---------- ---------- ---------- ---------- | 70 |
| XP_024577280.1 | ---------- ---------- ---------- ---------- ---------- ---------- ---------- ---------- | 95 |
| XP_024572924.1 | ---------- ---------- ---------- ---------- ---------- ---------- ---------- ---------- | 172 |
| PvCRN14 | ---------- ---------- ---------- ---------- ---------- ---------- ---------- ---------- | 77 |
| XP_024577521.1 | ---------- ---------- ---------- ---------- ---------- ---------- ---------- ---------- | 55 |
| PvCRN21 | ---------- ---------- ---------- ---------- ---------- ---------- ---------- ---------- | 66 |
| XP_024574966.1 | ---------- ---------- ---------- ---------- ---------- ---------- ---------- ---------- | 76 |
| XP_024575372.1 | ---------- ---------- ---------- ---------- ---------- ---------- ---------- ---------- | 77 |
| XP_024581363.1 | ---------- ---------- ---------- ---------- ---------- ---------- ---------- ---------- | 1 |
| XP_024583883.1 | ---------- ---------- ---------- ---------- ---------- ---------- ---------- ---------- | 43 |
| XP_024579844.1 | ---------- ---------- ---------- ---------- ---------- ---------- ---------- ---------- | 118 |
| XP_024574916.1 | ---------- ---------- ---------- ---------- ---------- ---------- ---------- ---------- | 120 |
| XP_024574193.1 | ---------- ---------- ---------- ---------- ---------- ---------- ---------- ---------- | 184 |
| XP_024583155.1 | ---------- ---------- ---------- ---------- ---------- ---------- ---------- ---------- | 68 |
| XP_024574913.1 | ---------- ---------- ---------- ---------- ---------- ---------- ---------- ---------- | 71 |
| XP_024578158.1 | ---------- ---------- ---------- ---------- ---------- ---------- ---------- ---------- | 41 |
| PvCRN22 | ---------- ---------- ---------- ---------- ---------- ---------- ---------- ---------- | 71 |
| XP_024585001.1 | ---------- ---------- ---------- ---------- ---------- ---------- ---------- ---------- | 32 |
| XP_024583843.1 | ---------- ---------- ---------- ---------- ---------- ---------- ---------- ---------- | 116 |
| XP_024578702.1 | ---------- ---------- ---------- ---------- ---------- ---------- ---------- ---------- | 39 |
| XP_024585805.1 | ---------- ---------- ---------- ---------- ---------- ---------- ---------- ---------- | 97 |
| XP_024580932.1 | ---------- ---------- ---------- ---------- ---------- ---------- ---------- ---------- | 153 |
| Consensus | ---------- ---------- ---------- ---------- ---------- ---------- ---------- ---------- | |

100%
Conservation
0%

| | 3,060 · 3,080 · 3,100 · 3,120 | |
|---|---|---|
| PvCRN11 | ---------- ---------- ---------- ---------- ---------- ---------- ---------- ---------- | 70 |
| XP_024579260.1 | ---------- ---------- ---------- ---------- ---------- ---------- ---------- ---------- | 73 |
| XP_024573634.1 | ---------- ---------- ---------- ---------- ---------- ---------- ---------- ---------- | 67 |
| PvCRN7 | ---------- ---------- ---------- ---------- ---------- ---------- ---------- ---------- | 98 |
| XP_024585265.1 | ---------- ---------- ---------- ---------- ---------- ---------- ---------- ---------- | 78 |
| PvCRN18 | ---------- ---------- ---------- ---------- ---------- ---------- ---------- ---------- | 72 |
| XP_024578414.1 | ---------- ---------- ---------- ---------- ---------- ---------- ---------- ---------- | 384 |
| XP_024579130.1 | ---------- ---------- ---------- ---------- ---------- ---------- ---------- ---------- | 93 |
| XP_024584758.1 | ---------- ---------- ---------- ---------- ---------- ---------- ---------- ---------- | 84 |
| XP_024586066.1 | ---------- ---------- ---------- ---------- ---------- ---------- ---------- ---------- | 99 |
| XP_024585870.1 | ---------- ---------- ---------- ---------- ---------- ---------- ---------- ---------- | 45 |
| PvCRN31 | ---------- ---------- ---------- ---------- ---------- ---------- ---------- ---------- | 81 |
| XP_024577278.1 | ---------- ---------- ---------- ---------- ---------- ---------- ---------- ---------- | 101 |
| XP_024585562.1 | ---------- ---------- ---------- ---------- ---------- ---------- ---------- ---------- | 6 |
| XP_024573944.1 | ---------- ---------- ---------- ---------- ---------- ---------- ---------- ---------- | 73 |
| XP_024579793.1 | ---------- ---------- ---------- ---------- ---------- ---------- ---------- ---------- | 23 |
| PvCRN15 | ---------- ---------- ---------- ---------- ---------- ---------- ---------- ---------- | 81 |
| PvCRN26 | ---------- ---------- ---------- ---------- ---------- ---------- ---------- ---------- | 80 |
| PvCRN16 | ---------- ---------- ---------- ---------- ---------- ---------- ---------- ---------- | 80 |
| PvCRN23 | ---------- ---------- ---------- ---------- ---------- ---------- ---------- ---------- | 101 |
| XP_024573063.1 | ---------- ---------- ---------- ---------- ---------- ---------- ---------- ---------- | 77 |
| PvCRN19 | ---------- ---------- ---------- ---------- ---------- ---------- ---------- ---------- | 76 |
| XP_024581625.1 | ---------- ---------- ---------- ---------- ---------- ---------- ---------- ---------- | 71 |
| XP_024583886.1 | ---------- ---------- ---------- ---------- ---------- ---------- ---------- ---------- | 71 |
| XP_024584409.1 | ---------- ---------- ---------- ---------- ---------- ---------- ---------- ---------- | 72 |
| XP_024572385.1 | ---------- ---------- ---------- ---------- ---------- ---------- ---------- ---------- | 68 |
| PvCRN6 | ---------- ---------- ---------- ---------- ---------- ---------- ---------- ---------- | 75 |
| PvCRN9 | ---------- ---------- ---------- ---------- ---------- ---------- ---------- ---------- | 71 |
| XP_024580875.1 | ---------- ---------- ---------- ---------- ---------- ---------- ---------- ---------- | 54 |
| PvCRN24 | ---------- ---------- ---------- ---------- ---------- ---------- ---------- ---------- | 77 |
| PvCRN35 | ---------- ---------- ---------- ---------- ---------- ---------- ---------- ---------- | 77 |
| PvCRN12 | ---------- ---------- ---------- ---------- ---------- ---------- ---------- ---------- | 77 |
| XP_024576927.1 | ---------- ---------- ---------- ---------- ---------- ---------- ---------- ---------- | 118 |
| XP_024578927.1 | ---------- ---------- ---------- ---------- ---------- ---------- ---------- ---------- | 76 |
| XP_024586212.1 | ---------- ---------- ---------- ---------- ---------- ---------- ---------- ---------- | 66 |
| XP_024578078.1 | ---------- ---------- ---------- ---------- ---------- ---------- ---------- ---------- | 60 |
| XP_024575355.1 | ---------- ---------- ---------- ---------- ---------- ---------- ---------- ---------- | 227 |
| XP_024584527.1 | ---------- ---------- ---------- ---------- ---------- ---------- ---------- ---------- | 78 |
| XP_024586664.1 | ---------- ---------- ---------- ---------- ---------- ---------- ---------- ---------- | 64 |
| PvCRN17 | ---------- ---------- ---------- ---------- ---------- ---------- ---------- ---------- | 77 |
| PvCRN2 | ---------- ---------- ---------- ---------- ---------- ---------- ---------- ---------- | 77 |
| XP_024577181.1 | ---------- ---------- ---------- ---------- ---------- ---------- ---------- ---------- | 39 |
| XP_024581075.1 | ---------- ---------- ---------- ---------- ---------- ---------- ---------- ---------- | 76 |
| XP_024575884.1 | ---------- ---------- ---------- ---------- ---------- ---------- ---------- ---------- | 75 |
| XP_024586872.1 | ---------- ---------- ---------- ---------- ---------- ---------- ---------- ---------- | 75 |
| XP_024573052.1 | ---------- ---------- ---------- ---------- ---------- ---------- ---------- ---------- | 98 |
| XP_024586054.1 | ---------- ---------- ---------- ---------- ---------- ---------- ---------- ---------- | 87 |
| PvCRN27 | ---------- ---------- ---------- ---------- ---------- ---------- ---------- ---------- | 72 |
| PvCRN20 | ---------- ---------- ---------- ---------- ---------- ---------- ---------- ---------- | 92 |
| PvCRN25 | ---------- ---------- ---------- ---------- ---------- ---------- ---------- ---------- | 78 |
| PvCRN1 | ---------- ---------- ---------- ---------- ---------- ---------- ---------- ---------- | 84 |
| PvCRN4 | ---------- ---------- ---------- ---------- ---------- ---------- ---------- ---------- | 79 |
| PvCRN30 | ---------- ---------- ---------- ---------- ---------- ---------- ---------- ---------- | 51 |
| PvCRN29 | ---------- ---------- ---------- ---------- ---------- ---------- ---------- ---------- | 72 |
| XP_024583036.1 | ---------- ---------- ---------- ---------- ---------- ---------- ---------- ---------- | 201 |
| PvCRN10 | ---------- ---------- ---------- ---------- ---------- ---------- ---------- ---------- | 70 |
| XP_024577280.1 | ---------- ---------- ---------- ---------- ---------- ---------- ---------- ---------- | 95 |
| XP_024572924.1 | ---------- ---------- ---------- ---------- ---------- ---------- ---------- ---------- | 172 |
| PvCRN14 | ---------- ---------- ---------- ---------- ---------- ---------- ---------- ---------- | 77 |
| XP_024577521.1 | ---------- ---------- ---------- ---------- ---------- ---------- ---------- ---------- | 55 |
| PvCRN21 | ---------- ---------- ---------- ---------- ---------- ---------- ---------- ---------- | 66 |
| XP_024574966.1 | ---------- ---------- ---------- ---------- ---------- ---------- ---------- ---------- | 76 |
| XP_024575372.1 | ---------- ---------- ---------- ---------- ---------- ---------- ---------- ---------- | 77 |
| XP_024581363.1 | ---------- ---------- ---------- ---------- ---------- ---------- ---------- ---------- | 1 |
| XP_024583883.1 | ---------- ---------- ---------- ---------- ---------- ---------- ---------- ---------- | 43 |
| XP_024579844.1 | ---------- ---------- ---------- ---------- ---------- ---------- ---------- ---------- | 118 |
| XP_024574916.1 | ---------- ---------- ---------- ---------- ---------- ---------- ---------- ---------- | 120 |
| XP_024574193.1 | ---------- ---------- ---------- ---------- ---------- ---------- ---------- ---------- | 184 |
| XP_024583155.1 | ---------- ---------- ---------- ---------- ---------- ---------- ---------- ---------- | 68 |
| XP_024574913.1 | ---------- ---------- ---------- ---------- ---------- ---------- ---------- ---------- | 71 |
| XP_024578158.1 | ---------- ---------- ---------- ---------- ---------- ---------- ---------- ---------- | 41 |
| PvCRN22 | ---------- ---------- ---------- ---------- ---------- ---------- ---------- ---------- | 71 |
| XP_024585001.1 | ---------- ---------- ---------- ---------- ---------- ---------- ---------- ---------- | 32 |
| XP_024583843.1 | ---------- ---------- ---------- ---------- ---------- ---------- ---------- ---------- | 116 |
| XP_024578702.1 | ---------- ---------- ---------- ---------- ---------- ---------- ---------- ---------- | 39 |
| XP_024585805.1 | ---------- ---------- ---------- ---------- ---------- ---------- ---------- ---------- | 97 |
| XP_024580932.1 | ---------- ---------- ---------- ---------- ---------- ---------- ---------- ---------- | 153 |
| Consensus | ---------- ---------- ---------- ---------- ---------- ---------- ---------- ---------- | |

Conservation (100% – 0%)

| Sequence | 3,140 … 3,160 … 3,180 … 3,200 | |
|---|---|---|
| PvCRN11 | ---------- ---------- ---------- ---------- ---------- ---------- ---------- ---------- | 70 |
| XP_024579260.1 | ---------- ---------- ---------- ---------- ---------- ---------- ---------- ---------- | 73 |
| XP_024573634.1 | ---------- ---------- ---------- ---------- ---------- ---------- ---------- ---------- | 67 |
| PvCRN7 | ---------- ---------- ---------- ---------- ---------- ---------- ---------- ---------- | 98 |
| XP_024585265.1 | ---------- ---------- ---------- ---------- ---------- ---------- ---------- ---------- | 78 |
| PvCRN18 | ---------- ---------- ---------- ---------- ---------- ---------- ---------- ---------- | 72 |
| XP_024578414.1 | ---------- ---------- ---------- ---------- ---------- ---------- ---------- ---------- | 384 |
| XP_024579130.1 | ---------- ---------- ---------- ---------- ---------- ---------- ---------- ---------- | 93 |
| XP_024584758.1 | ---------- ---------- ---------- ---------- ---------- ---------- ---------- ---------- | 84 |
| XP_024586066.1 | ---------- ---------- ---------- ---------- ---------- ---------- ---------- ---------- | 99 |
| XP_024585870.1 | ---------- ---------- ---------- ---------- ---------- ---------- ---------- ---------- | 45 |
| PvCRN31 | ---------- ---------- ---------- ---------- ---------- ---------- ---------- ---------- | 81 |
| XP_024577278.1 | ---------- ---------- ---------- ---------- ---------- ---------- ---------- ---------- | 101 |
| XP_024585562.1 | ---------- ---------- ---------- ---------- ---------- ---------- ---------- ---------- | 6 |
| XP_024573944.1 | ---------- ---------- ---------- ---------- ---------- ---------- ---------- ---------- | 73 |
| XP_024579793.1 | ---------- ---------- ---------- ---------- ---------- ---------- ---------- ---------- | 23 |
| PvCRN15 | ---------- ---------- ---------- ---------- ---------- ---------- ---------- ---------- | 81 |
| PvCRN26 | ---------- ---------- ---------- ---------- ---------- ---------- ---------- ---------- | 80 |
| PvCRN16 | ---------- ---------- ---------- ---------- ---------- ---------- ---------- ---------- | 80 |
| PvCRN23 | ---------- ---------- ---------- ---------- ---------- ---------- ---------- ---------- | 101 |
| XP_024573063.1 | ---------- ---------- ---------- ---------- ---------- ---------- ---------- ---------- | 77 |
| PvCRN19 | ---------- ---------- ---------- ---------- ---------- ---------- ---------- ---------- | 76 |
| XP_024581625.1 | ---------- ---------- ---------- ---------- ---------- ---------- ---------- ---------- | 71 |
| XP_024583886.1 | ---------- ---------- ---------- ---------- ---------- ---------- ---------- ---------- | 71 |
| XP_024584409.1 | ---------- ---------- ---------- ---------- ---------- ---------- ---------- ---------- | 72 |
| XP_024572385.1 | ---------- ---------- ---------- ---------- ---------- ---------- ---------- ---------- | 68 |
| PvCRN6 | ---------- ---------- ---------- ---------- ---------- ---------- ---------- ---------- | 75 |
| PvCRN9 | ---------- ---------- ---------- ---------- ---------- ---------- ---------- ---------- | 71 |
| XP_024580875.1 | ---------- ---------- ---------- ---------- ---------- ---------- ---------- ---------- | 54 |
| PvCRN24 | ---------- ---------- ---------- ---------- ---------- ---------- ---------- ---------- | 77 |
| PvCRN35 | ---------- ---------- ---------- ---------- ---------- ---------- ---------- ---------- | 77 |
| PvCRN12 | ---------- ---------- ---------- ---------- ---------- ---------- ---------- ---------- | 77 |
| XP_024576927.1 | ---------- ---------- ---------- ---------- ---------- ---------- ---------- ---------- | 118 |
| XP_024578927.1 | ---------- ---------- ---------- ---------- ---------- ---------- ---------- ---------- | 76 |
| XP_024586212.1 | ---------- ---------- ---------- ---------- ---------- ---------- ---------- ---------- | 66 |
| XP_024578078.1 | ---------- ---------- ---------- ---------- ---------- ---------- ---------- ---------- | 60 |
| XP_024575355.1 | ---------- ---------- ---------- ---------- ---------- ---------- ---------- ---------- | 227 |
| XP_024584527.1 | ---------- ---------- ---------- ---------- ---------- ---------- ---------- ---------- | 78 |
| XP_024586664.1 | ---------- ---------- ---------- ---------- ---------- ---------- ---------- ---------- | 64 |
| PvCRN17 | ---------- ---------- ---------- ---------- ---------- ---------- ---------- ---------- | 77 |
| PvCRN2 | ---------- ---------- ---------- ---------- ---------- ---------- ---------- ---------- | 77 |
| XP_024577181.1 | ---------- ---------- ---------- ---------- ---------- ---------- ---------- ---------- | 39 |
| XP_024581075.1 | ---------- ---------- ---------- ---------- ---------- ---------- ---------- ---------- | 76 |
| XP_024575884.1 | ---------- ---------- ---------- ---------- ---------- ---------- ---------- ---------- | 75 |
| XP_024586872.1 | ---------- ---------- ---------- ---------- ---------- ---------- ---------- ---------- | 75 |
| XP_024573052.1 | ---------- ---------- ---------- ---------- ---------- ---------- ---------- ---------- | 98 |
| XP_024586054.1 | ---------- ---------- ---------- ---------- ---------- ---------- ---------- ---------- | 87 |
| PvCRN27 | ---------- ---------- ---------- ---------- ---------- ---------- ---------- ---------- | 72 |
| PvCRN20 | ---------- ---------- ---------- ---------- ---------- ---------- ---------- ---------- | 92 |
| PvCRN25 | ---------- ---------- ---------- ---------- ---------- ---------- ---------- ---------- | 78 |
| PvCRN1 | ---------- ---------- ---------- ---------- ---------- ---------- ---------- ---------- | 84 |
| PvCRN4 | ---------- ---------- ---------- ---------- ---------- ---------- ---------- ---------- | 79 |
| PvCRN30 | ---------- ---------- ---------- ---------- ---------- ---------- ---------- ---------- | 51 |
| PvCRN29 | ---------- ---------- ---------- ---------- ---------- ---------- ---------- ---------- | 72 |
| XP_024583036.1 | ---------- ---------- ---------- ---------- ---------- ---------- ---------- ---------- | 201 |
| PvCRN10 | ---------- ---------- ---------- ---------- ---------- ---------- ---------- ---------- | 70 |
| XP_024577280.1 | ---------- ---------- ---------- ---------- ---------- ---------- ---------- ---------- | 95 |
| XP_024572924.1 | ---------- ---------- ---------- ---------- ---------- ---------- ---------- ---------- | 172 |
| PvCRN14 | ---------- ---------- ---------- ---------- ---------- ---------- ---------- ---------- | 77 |
| XP_024577521.1 | ---------- ---------- ---------- ---------- ---------- ---------- ---------- ---------- | 55 |
| PvCRN21 | ---------- ---------- ---------- ---------- ---------- ---------- ---------- ---------- | 66 |
| XP_024574966.1 | ---------- ---------- ---------- ---------- ---------- ---------- ---------- ---------- | 76 |
| XP_024575372.1 | ---------- ---------- ---------- ---------- ---------- ---------- ---------- ---------- | 77 |
| XP_024581363.1 | ---------- ---------- ---------- ---------- ---------- ---------- ---------- ---------- | 1 |
| XP_024583883.1 | ---------- ---------- ---------- ---------- ---------- ---------- ---------- ---------- | 43 |
| XP_024579844.1 | ---------- ---------- ---------- ---------- ---------- ---------- ---------- ---------- | 118 |
| XP_024574916.1 | ---------- ---------- ---------- ---------- ---------- ---------- ---------- ---------- | 120 |
| XP_024574193.1 | ---------- ---------- ---------- ---------- ---------- ---------- ---------- ---------- | 184 |
| XP_024583155.1 | ---------- ---------- ---------- ---------- ---------- ---------- ---------- ---------- | 68 |
| XP_024574913.1 | ---------- ---------- ---------- ---------- ---------- ---------- ---------- ---------- | 71 |
| XP_024578158.1 | ---------- ---------- ---------- ---------- ---------- ---------- ---------- ---------- | 41 |
| PvCRN22 | ---------- ---------- ---------- ---------- ---------- ---------- ---------- ---------- | 71 |
| XP_024585001.1 | ---------- ---------- ---------- ---------- ---------- ---------- ---------- ---------- | 32 |
| XP_024583843.1 | ---------- ---------- ---------- ---------- ---------- ---------- ---------- ---------- | 116 |
| XP_024578702.1 | ---------- ---------- ---------- ---------- ---------- ---------- ---------- ---------- | 39 |
| XP_024585805.1 | ---------- ---------- ---------- ---------- ---------- ---------- ---------- ---------- | 97 |
| XP_024580932.1 | ---------- ---------- ---------- ---------- ---------- ---------- ---------- ---------- | 153 |
| Consensus | ---------- ---------- ---------- ---------- ---------- ---------- ---------- ---------- | |

Conservation (100% – 0%)

| | 3,220 3,240 3,260 3,280 | |
|---|---|---|
| PvCRN11 | ---------- ---------- ---------- ---------- ---------- ---------- ---------- ---------- | 70 |
| XP_024579260.1 | ---------- ---------- ---------- ---------- ---------- ---------- ---------- ---------- | 73 |
| XP_024573634.1 | ---------- ---------- ---------- ---------- ---------- ---------- ---------- ---------- | 67 |
| PvCRN7 | ---------- ---------- ---------- ---------- ---------- ---------- ---------- ---------- | 98 |
| XP_024585265.1 | ---------- ---------- ---------- ---------- ---------- ---------- ---------- ---------- | 78 |
| PvCRN18 | ---------- ---------- ---------- ---------- ---------- ---------- ---------- ---------- | 72 |
| XP_024578414.1 | ---------- ---------- ---------- ---------- ---------- ---------- ---------- ---------- | 384 |
| XP_024579130.1 | ---------- ---------- ---------- ---------- ---------- ---------- ---------- ---------- | 93 |
| XP_024584758.1 | ---------- ---------- ---------- ---------- ---------- ---------- ---------- ---------- | 84 |
| XP_024586066.1 | ---------- ---------- ---------- ---------- ---------- ---------- ---------- ---------- | 99 |
| XP_024585870.1 | ---------- ---------- ---------- ---------- ---------- ---------- ---------- ---------- | 45 |
| PvCRN31 | ---------- ---------- ---------- ---------- ---------- ---------- ---------- ---------- | 81 |
| XP_024577278.1 | ---------- ---------- ---------- ---------- ---------- ---------- ---------- ---------- | 101 |
| XP_024585562.1 | ---------- ---------- ---------- ---------- ---------- ---------- ---------- ---------- | 6 |
| XP_024573944.1 | ---------- ---------- ---------- ---------- ---------- ---------- ---------- ---------- | 73 |
| XP_024579793.1 | ---------- ---------- ---------- ---------- ---------- ---------- ---------- ---------- | 23 |
| PvCRN15 | ---------- ---------- ---------- ---------- ---------- ---------- ---------- ---------- | 81 |
| PvCRN26 | ---------- ---------- ---------- ---------- ---------- ---------- ---------- ---------- | 80 |
| PvCRN16 | ---------- ---------- ---------- ---------- ---------- ---------- ---------- ---------- | 80 |
| PvCRN23 | ---------- ---------- ---------- ---------- ---------- ---------- ---------- ---------- | 101 |
| XP_024573063.1 | ---------- ---------- ---------- ---------- ---------- ---------- ---------- ---------- | 77 |
| PvCRN19 | ---------- ---------- ---------- ---------- ---------- ---------- ---------- ---------- | 76 |
| XP_024581625.1 | ---------- ---------- ---------- ---------- ---------- ---------- ---------- ---------- | 71 |
| XP_024583886.1 | ---------- ---------- ---------- ---------- ---------- ---------- ---------- ---------- | 71 |
| XP_024584409.1 | ---------- ---------- ---------- ---------- ---------- ---------- ---------- ---------- | 72 |
| XP_024572385.1 | ---------- ---------- ---------- ---------- ---------- ---------- ---------- ---------- | 68 |
| PvCRN6 | ---------- ---------- ---------- ---------- ---------- ---------- ---------- ---------- | 75 |
| PvCRN9 | ---------- ---------- ---------- ---------- ---------- ---------- ---------- ---------- | 71 |
| XP_024580875.1 | ---------- ---------- ---------- ---------- ---------- ---------- ---------- ---------- | 54 |
| PvCRN24 | ---------- ---------- ---------- ---------- ---------- ---------- ---------- ---------- | 77 |
| PvCRN35 | ---------- ---------- ---------- ---------- ---------- ---------- ---------- ---------- | 77 |
| PvCRN12 | ---------- ---------- ---------- ---------- ---------- ---------- ---------- ---------- | 77 |
| XP_024576927.1 | ---------- ---------- ---------- ---------- ---------- ---------- ---------- ---------- | 118 |
| XP_024578927.1 | ---------- ---------- ---------- ---------- ---------- ---------- ---------- ---------- | 76 |
| XP_024586212.1 | ---------- ---------- ---------- ---------- ---------- ---------- ---------- ---------- | 66 |
| XP_024578078.1 | ---------- ---------- ---------- ---------- ---------- ---------- ---------- ---------- | 60 |
| XP_024575355.1 | ---------- ---------- ---------- ---------- ---------- ---------- ---------- ---------- | 227 |
| XP_024584527.1 | ---------- ---------- ---------- ---------- ---------- ---------- ---------- ---------- | 78 |
| XP_024586664.1 | ---------- ---------- ---------- ---------- ---------- ---------- ---------- ---------- | 64 |
| PvCRN17 | ---------- ---------- ---------- ---------- ---------- ---------- ---------- ---------- | 77 |
| PvCRN2 | ---------- ---------- ---------- ---------- ---------- ---------- ---------- ---------- | 77 |
| XP_024577181.1 | ---------- ---------- ---------- ---------- ---------- ---------- ---------- ---------- | 39 |
| XP_024581075.1 | ---------- ---------- ---------- ---------- ---------- ---------- ---------- ---------- | 76 |
| XP_024575884.1 | ---------- ---------- ---------- ---------- ---------- ---------- ---------- ---------- | 75 |
| XP_024586872.1 | ---------- ---------- ---------- ---------- ---------- ---------- ---------- ---------- | 75 |
| XP_024573052.1 | ---------- ---------- ---------- ---------- ---------- ---------- ---------- ---------- | 98 |
| XP_024586054.1 | ---------- ---------- ---------- ---------- ---------- ---------- ---------- ---------- | 87 |
| PvCRN27 | ---------- ---------- ---------- ---------- ---------- ---------- ---------- ---------- | 72 |
| PvCRN20 | ---------- ---------- ---------- ---------- ---------- ---------- ---------- ---------- | 92 |
| PvCRN25 | ---------- ---------- ---------- ---------- ---------- ---------- ---------- ---------- | 78 |
| PvCRN1 | ---------- ---------- ---------- ---------- ---------- ---------- ---------- ---------- | 84 |
| PvCRN4 | ---------- ---------- ---------- ---------- ---------- ---------- ---------- ---------- | 79 |
| PvCRN30 | ---------- ---------- ---------- ---------- ---------- ---------- ---------- ---------- | 51 |
| PvCRN29 | ---------- ---------- ---------- ---------- ---------- ---------- ---------- ---------- | 72 |
| XP_024583036.1 | ---------- ---------- ---------- ---------- ---------- ---------- ---------- ---------- | 201 |
| PvCRN10 | ---------- ---------- ---------- ---------- ---------- ---------- ---------- ---------- | 70 |
| XP_024577280.1 | ---------- ---------- ---------- ---------- ---------- ---------- ---------- ---------- | 95 |
| XP_024572924.1 | ---------- ---------- ---------- ---------- ---------- ---------- ---------- ---------- | 172 |
| PvCRN14 | ---------- ---------- ---------- ---------- ---------- ---------- ---------- ---------- | 77 |
| XP_024577521.1 | ---------- ---------- ---------- ---------- ---------- ---------- ---------- ---------- | 55 |
| PvCRN21 | ---------- ---------- ---------- ---------- ---------- ---------- ---------- ---------- | 66 |
| XP_024574966.1 | ---------- ---------- ---------- ---------- ---------- ---------- ---------- ---------- | 76 |
| XP_024575372.1 | ---------- ---------- ---------- ---------- ---------- ---------- ---------- ---------- | 77 |
| XP_024581363.1 | ---------- ---------- ---------- ---------- ---------- ---------- ---------- ---------- | 1 |
| XP_024583883.1 | ---------- ---------- ---------- ---------- ---------- ---------- ---------- ---------- | 43 |
| XP_024579844.1 | ---------- ---------- ---------- ---------- ---------- ---------- ---------- ---------- | 118 |
| XP_024574916.1 | ---------- ---------- ---------- ---------- ---------- ---------- ---------- ---------- | 120 |
| XP_024574193.1 | ---------- ---------- ---------- ---------- ---------- ---------- ---------- ---------- | 184 |
| XP_024583155.1 | ---------- ---------- ---------- ---------- ---------- ---------- ---------- ---------- | 68 |
| XP_024574913.1 | ---------- ---------- ---------- ---------- ---------- ---------- ---------- ---------- | 71 |
| XP_024578158.1 | ---------- ---------- ---------- ---------- ---------- ---------- ---------- ---------- | 41 |
| PvCRN22 | ---------- ---------- ---------- ---------- ---------- ---------- ---------- ---------- | 71 |
| XP_024585001.1 | ---------- ---------- ---------- ---------- ---------- ---------- ---------- ---------- | 32 |
| XP_024583843.1 | ---------- ---------- ---------- ---------- ---------- ---------- ---------- ---------- | 116 |
| XP_024578702.1 | ---------- ---------- ---------- ---------- ---------- ---------- ---------- ---------- | 39 |
| XP_024585805.1 | ---------- ---------- ---------- ---------- ---------- ---------- ---------- ---------- | 97 |
| XP_024580932.1 | ---------- ---------- ---------- ---------- ---------- ---------- ---------- ---------- | 153 |
| Consensus | ---------- ---------- ---------- ---------- ---------- ---------- ---------- ---------- | |
| Conservation (100% / 0%) | | |

| | 3,300 3,320 3,340 3,360 | |
|---|---|---|
| PvCRN11 | ---------- ---------- ---------- ---------- ---------- ---------- ---------- ---------- | 70 |
| XP_024579260.1 | ---------- ---------- ---------- ---------- ---------- ---------- ---------- ---------- | 73 |
| XP_024573634.1 | ---------- ---------- ---------- ---------- ---------- ---------- ---------- ---------- | 67 |
| PvCRN7 | ---------- ---------- ---------- ---------- ---------- ---------- ---------- ---------- | 98 |
| XP_024585265.1 | ---------- ---------- ---------- ---------- ---------- ---------- ---------- ---------- | 78 |
| PvCRN18 | ---------- ---------- ---------- ---------- ---------- ---------- ---------- ---------- | 72 |
| XP_024578414.1 | ---------- ---------- ---------- ---------- ---------- ---------- ---------- ---------- | 384 |
| XP_024579130.1 | ---------- ---------- ---------- ---------- ---------- ---------- ---------- ---------- | 93 |
| XP_024584758.1 | ---------- ---------- ---------- ---------- ---------- ---------- ---------- ---------- | 84 |
| XP_024586066.1 | ---------- ---------- ---------- ---------- ---------- ---------- ---------- ---------- | 99 |
| XP_024585870.1 | ---------- ---------- ---------- ---------- ---------- ---------- ---------- ---------- | 45 |
| PvCRN31 | ---------- ---------- ---------- ---------- ---------- ---------- ---------- ---------- | 81 |
| XP_024577278.1 | ---------- ---------- ---------- ---------- ---------- ---------- ---------- ---------- | 101 |
| XP_024585562.1 | ---------- ---------- ---------- ---------- ---------- ---------- ---------- ---------- | 6 |
| XP_024573944.1 | ---------- ---------- ---------- ---------- ---------- ---------- ---------- ---------- | 73 |
| XP_024579793.1 | ---------- ---------- ---------- ---------- ---------- ---------- ---------- ---------- | 23 |
| PvCRN15 | ---------- ---------- ---------- ---------- ---------- ---------- ---------- ---------- | 81 |
| PvCRN26 | ---------- ---------- ---------- ---------- ---------- ---------- ---------- ---------- | 80 |
| PvCRN16 | ---------- ---------- ---------- ---------- ---------- ---------- ---------- ---------- | 80 |
| PvCRN23 | ---------- ---------- ---------- ---------- ---------- ---------- ---------- ---------- | 101 |
| XP_024573063.1 | ---------- ---------- ---------- ---------- ---------- ---------- ---------- ---------- | 77 |
| PvCRN19 | ---------- ---------- ---------- ---------- ---------- ---------- ---------- ---------- | 76 |
| XP_024581625.1 | ---------- ---------- ---------- ---------- ---------- ---------- ---------- ---------- | 71 |
| XP_024583886.1 | ---------- ---------- ---------- ---------- ---------- ---------- ---------- ---------- | 71 |
| XP_024584409.1 | ---------- ---------- ---------- ---------- ---------- ---------- ---------- ---------- | 72 |
| XP_024572385.1 | ---------- ---------- ---------- ---------- ---------- ---------- ---------- ---------- | 68 |
| PvCRN6 | ---------- ---------- ---------- ---------- ---------- ---------- ---------- ---------- | 75 |
| PvCRN9 | ---------- ---------- ---------- ---------- ---------- ---------- ---------- ---------- | 71 |
| XP_024580875.1 | ---------- ---------- ---------- ---------- ---------- ---------- ---------- ---------- | 54 |
| PvCRN24 | ---------- ---------- ---------- ---------- ---------- ---------- ---------- ---------- | 77 |
| PvCRN35 | ---------- ---------- ---------- ---------- ---------- ---------- ---------- ---------- | 77 |
| PvCRN12 | ---------- ---------- ---------- ---------- ---------- ---------- ---------- ---------- | 77 |
| XP_024576927.1 | ---------- ---------- ---------- ---------- ---------- ---------- ---------- ---------- | 118 |
| XP_024578927.1 | ---------- ---------- ---------- ---------- ---------- ---------- ---------- ---------- | 76 |
| XP_024586212.1 | ---------- ---------- ---------- ---------- ---------- ---------- ---------- ---------- | 66 |
| XP_024578078.1 | ---------- ---------- ---------- ---------- ---------- ---------- ---------- ---------- | 60 |
| XP_024575355.1 | ---------- ---------- ---------- ---------- ---------- ---------- ---------- ---------- | 227 |
| XP_024584527.1 | ---------- ---------- ---------- ---------- ---------- ---------- ---------- ---------- | 78 |
| XP_024586664.1 | ---------- ---------- ---------- ---------- ---------- ---------- ---------- ---------- | 64 |
| PvCRN17 | ---------- ---------- ---------- ---------- ---------- ---------- ---------- ---------- | 77 |
| PvCRN2 | ---------- ---------- ---------- ---------- ---------- ---------- ---------- ---------- | 77 |
| XP_024577181.1 | ---------- ---------- ---------- ---------- ---------- ---------- ---------- ---------- | 39 |
| XP_024581075.1 | ---------- ---------- ---------- ---------- ---------- ---------- ---------- ---------- | 76 |
| XP_024575884.1 | ---------- ---------- ---------- ---------- ---------- ---------- ---------- ---------- | 75 |
| XP_024586872.1 | ---------- ---------- ---------- ---------- ---------- ---------- ---------- ---------- | 75 |
| XP_024573052.1 | ---------- ---------- ---------- ---------- ---------- ---------- ---------- ---------- | 98 |
| XP_024586054.1 | ---------- ---------- ---------- ---------- ---------- ---------- ---------- ---------- | 87 |
| PvCRN27 | ---------- ---------- ---------- ---------- ---------- ---------- ---------- ---------- | 72 |
| PvCRN20 | ---------- ---------- ---------- ---------- ---------- ---------- ---------- ---------- | 92 |
| PvCRN25 | ---------- ---------- ---------- ---------- ---------- ---------- ---------- ---------- | 78 |
| PvCRN1 | ---------- ---------- ---------- ---------- ---------- ---------- ---------- ---------- | 84 |
| PvCRN4 | ---------- ---------- ---------- ---------- ---------- ---------- ---------- ---------- | 79 |
| PvCRN30 | ---------- ---------- ---------- ---------- ---------- ---------- ---------- ---------- | 51 |
| PvCRN29 | ---------- ---------- ---------- ---------- ---------- ---------- ---------- ---------- | 72 |
| XP_024583036.1 | ---------- ---------- ---------- ---------- ---------- ---------- ---------- ---------- | 201 |
| PvCRN10 | ---------- ---------- ---------- ---------- ---------- ---------- ---------- ---------- | 70 |
| XP_024577280.1 | ---------- ---------- ---------- ---------- ---------- ---------- ---------- ---------- | 95 |
| XP_024572924.1 | ---------- ---------- ---------- ---------- ---------- ---------- ---------- ---------- | 172 |
| PvCRN14 | ---------- ---------- ---------- ---------- ---------- ---------- ---------- ---------- | 77 |
| XP_024577521.1 | ---------- ---------- ---------- ---------- ---------- ---------- ---------- ---------- | 55 |
| PvCRN21 | ---------- ---------- ---------- ---------- ---------- ---------- ---------- ---------- | 66 |
| XP_024574966.1 | ---------- ---------- ---------- ---------- ---------- ---------- ---------- ---------- | 76 |
| XP_024575372.1 | ---------- ---------- ---------- ---------- ---------- ---------- ---------- ---------- | 77 |
| XP_024581363.1 | ---------- ---------- ---------- ---------- ---------- ---------- ---------- ---------- | 1 |
| XP_024583883.1 | ---------- ---------- ---------- ---------- ---------- ---------- ---------- ---------- | 43 |
| XP_024579844.1 | ---------- ---------- ---------- ---------- ---------- ---------- ---------- ---------- | 118 |
| XP_024574916.1 | ---------- ---------- ---------- ---------- ---------- ---------- ---------- ---------- | 120 |
| XP_024574193.1 | ---------- ---------- ---------- ---------- ---------- ---------- ---------- ---------- | 184 |
| XP_024583155.1 | ---------- ---------- ---------- ---------- ---------- ---------- ---------- ---------- | 68 |
| XP_024574913.1 | ---------- ---------- ---------- ---------- ---------- ---------- ---------- ---------- | 71 |
| XP_024578158.1 | ---------- ---------- ---------- ---------- ---------- ---------- ---------- ---------- | 41 |
| PvCRN22 | ---------- ---------- ---------- ---------- ---------- ---------- ---------- ---------- | 71 |
| XP_024585001.1 | ---------- ---------- ---------- ---------- ---------- ---------- ---------- ---------- | 32 |
| XP_024583843.1 | ---------- ---------- ---------- ---------- ---------- ---------- ---------- ---------- | 116 |
| XP_024578702.1 | ---------- ---------- ---------- ---------- ---------- ---------- ---------- ---------- | 39 |
| XP_024585805.1 | ---------- ---------- ---------- ---------- ---------- ---------- ---------- ---------- | 97 |
| XP_024580932.1 | ---------- ---------- ---------- ---------- ---------- ---------- ---------- ---------- | 153 |
| Consensus | ---------- ---------- ---------- ---------- ---------- ---------- ---------- ---------- | |
| Conservation (100% / 0%) | | |

| | 3,380 3,400 3,420 3,440 | |
|---|---|---|
| PvCRN11 | ---------- ---------- ---------- ---------- ---------- ---------- ---------- ---------- | 70 |
| XP_024579260.1 | ---------- ---------- ---------- ---------- ---------- ---------- ---------- ---------- | 73 |
| XP_024573634.1 | ---------- ---------- ---------- ---------- ---------- ---------- ---------- ---------- | 67 |
| PvCRN7 | ---------- ---------- ---------- ---------- ---------- ---------- ---------- ---------- | 98 |
| XP_024585265.1 | ---------- ---------- ---------- ---------- ---------- ---------- ---------- ---------- | 78 |
| PvCRN18 | ---------- ---------- ---------- ---------- ---------- ---------- ---------- ---------- | 72 |
| XP_024578414.1 | ---------- ---------- ---------- ---------- ---------- ---------- ---------- ---------- | 384 |
| XP_024579130.1 | ---------- ---------- ---------- ---------- ---------- ---------- ---------- ---------- | 93 |
| XP_024584758.1 | ---------- ---------- ---------- ---------- ---------- ---------- ---------- ---------- | 84 |
| XP_024586066.1 | ---------- ---------- ---------- ---------- ---------- ---------- ---------- ---------- | 99 |
| XP_024585870.1 | ---------- ---------- ---------- ---------- ---------- ---------- ---------- ---------- | 45 |
| PvCRN31 | ---------- ---------- ---------- ---------- ---------- ---------- ---------- ---------- | 81 |
| XP_024577278.1 | ---------- ---------- ---------- ---------- ---------- ---------- ---------- ---------- | 101 |
| XP_024585562.1 | ---------- ---------- ---------- ---------- ---------- ---------- ---------- ---------- | 6 |
| XP_024573944.1 | ---------- ---------- ---------- ---------- ---------- ---------- ---------- ---------- | 73 |
| XP_024579793.1 | ---------- ---------- ---------- ---------- ---------- ---------- ---------- ---------- | 23 |
| PvCRN15 | ---------- ---------- ---------- ---------- ---------- ---------- ---------- ---------- | 81 |
| PvCRN26 | ---------- ---------- ---------- ---------- ---------- ---------- ---------- ---------- | 80 |
| PvCRN16 | ---------- ---------- ---------- ---------- ---------- ---------- ---------- ---------- | 80 |
| PvCRN23 | ---------- ---------- ---------- ---------- ---------- ---------- ---------- ---------- | 101 |
| XP_024573063.1 | ---------- ---------- ---------- ---------- ---------- ---------- ---------- ---------- | 77 |
| PvCRN19 | ---------- ---------- ---------- ---------- ---------- ---------- ---------- ---------- | 76 |
| XP_024581625.1 | ---------- ---------- ---------- ---------- ---------- ---------- ---------- ---------- | 71 |
| XP_024583886.1 | ---------- ---------- ---------- ---------- ---------- ---------- ---------- ---------- | 71 |
| XP_024584409.1 | ---------- ---------- ---------- ---------- ---------- ---------- ---------- ---------- | 72 |
| XP_024572385.1 | ---------- ---------- ---------- ---------- ---------- ---------- ---------- ---------- | 68 |
| PvCRN6 | ---------- ---------- ---------- ---------- ---------- ---------- ---------- ---------- | 75 |
| PvCRN9 | ---------- ---------- ---------- ---------- ---------- ---------- ---------- ---------- | 71 |
| XP_024580875.1 | ---------- ---------- ---------- ---------- ---------- ---------- ---------- ---------- | 54 |
| PvCRN24 | ---------- ---------- ---------- ---------- ---------- ---------- ---------- ---------- | 77 |
| PvCRN35 | ---------- ---------- ---------- ---------- ---------- ---------- ---------- ---------- | 77 |
| PvCRN12 | ---------- ---------- ---------- ---------- ---------- ---------- ---------- ---------- | 77 |
| XP_024576927.1 | ---------- ---------- ---------- ---------- ---------- ---------- ---------- ---------- | 118 |
| XP_024578927.1 | ---------- ---------- ---------- ---------- ---------- ---------- ---------- ---------- | 76 |
| XP_024586212.1 | ---------- ---------- ---------- ---------- ---------- ---------- ---------- ---------- | 66 |
| XP_024578078.1 | ---------- ---------- ---------- ---------- ---------- ---------- ---------- ---------- | 60 |
| XP_024575355.1 | ---------- ---------- ---------- ---------- ---------- ---------- ---------- ---------- | 227 |
| XP_024584527.1 | ---------- ---------- ---------- ---------- ---------- ---------- ---------- ---------- | 78 |
| XP_024586664.1 | ---------- ---------- ---------- ---------- ---------- ---------- ---------- ---------- | 64 |
| PvCRN17 | ---------- ---------- ---------- ---------- ---------- ---------- ---------- ---------- | 77 |
| PvCRN2 | ---------- ---------- ---------- ---------- ---------- ---------- ---------- ---------- | 77 |
| XP_024577181.1 | ---------- ---------- ---------- ---------- ---------- ---------- ---------- ---------- | 39 |
| XP_024581075.1 | ---------- ---------- ---------- ---------- ---------- ---------- ---------- ---------- | 76 |
| XP_024575884.1 | ---------- ---------- ---------- ---------- ---------- ---------- ---------- ---------- | 75 |
| XP_024586872.1 | ---------- ---------- ---------- ---------- ---------- ---------- ---------- ---------- | 75 |
| XP_024573052.1 | ---------- ---------- ---------- ---------- ---------- ---------- ---------- ---------- | 98 |
| XP_024586054.1 | ---------- ---------- ---------- ---------- ---------- ---------- ---------- ---------- | 87 |
| PvCRN27 | ---------- ---------- ---------- ---------- ---------- ---------- ---------- ---------- | 72 |
| PvCRN20 | ---------- ---------- ---------- ---------- ---------- ---------- ---------- ---------- | 92 |
| PvCRN25 | ---------- ---------- ---------- ---------- ---------- ---------- ---------- ---------- | 78 |
| PvCRN1 | ---------- ---------- ---------- ---------- ---------- ---------- ---------- ---------- | 84 |
| PvCRN4 | ---------- ---------- ---------- ---------- ---------- ---------- ---------- ---------- | 79 |
| PvCRN30 | ---------- ---------- ---------- ---------- ---------- ---------- ---------- ---------- | 51 |
| PvCRN29 | ---------- ---------- ---------- ---------- ---------- ---------- ---------- ---------- | 72 |
| XP_024583036.1 | ---------- ---------- ---------- ---------- ---------- ---------- ---------- ---------- | 201 |
| PvCRN10 | ---------- ---------- ---------- ---------- ---------- ---------- ---------- ---------- | 70 |
| XP_024577280.1 | ---------- ---------- ---------- ---------- ---------- ---------- ---------- ---------- | 95 |
| XP_024572924.1 | ---------- ---------- ---------- ---------- ---------- ---------- ---------- ---------- | 172 |
| PvCRN14 | ---------- ---------- ---------- ---------- ---------- ---------- ---------- ---------- | 77 |
| XP_024577521.1 | ---------- ---------- ---------- ---------- ---------- ---------- ---------- ---------- | 55 |
| PvCRN21 | ---------- ---------- ---------- ---------- ---------- ---------- ---------- ---------- | 66 |
| XP_024574966.1 | ---------- ---------- ---------- ---------- ---------- ---------- ---------- ---------- | 76 |
| XP_024575372.1 | ---------- ---------- ---------- ---------- ---------- ---------- ---------- ---------- | 77 |
| XP_024581363.1 | ---------- ---------- ---------- ---------- ---------- ---------- ---------- ---------- | 1 |
| XP_024583883.1 | ---------- ---------- ---------- ---------- ---------- ---------- ---------- ---------- | 43 |
| XP_024579844.1 | ---------- ---------- ---------- ---------- ---------- ---------- ---------- ---------- | 118 |
| XP_024574916.1 | ---------- ---------- ---------- ---------- ---------- ---------- ---------- ---------- | 120 |
| XP_024574193.1 | ---------- ---------- ---------- ---------- ---------- ---------- ---------- ---------- | 184 |
| XP_024583155.1 | ---------- ---------- ---------- ---------- ---------- ---------- ---------- ---------- | 68 |
| XP_024574913.1 | ---------- ---------- ---------- ---------- ---------- ---------- ---------- ---------- | 71 |
| XP_024578158.1 | ---------- ---------- ---------- ---------- ---------- ---------- ---------- ---------- | 41 |
| PvCRN22 | ---------- ---------- ---------- ---------- ---------- ---------- ---------- ---------- | 71 |
| XP_024585001.1 | ---------- ---------- ---------- ---------- ---------- ---------- ---------- ---------- | 32 |
| XP_024583843.1 | ---------- ---------- ---------- ---------- ---------- ---------- ---------- ---------- | 116 |
| XP_024578702.1 | ---------- ---------- ---------- ---------- ---------- ---------- ---------- ---------- | 39 |
| XP_024585805.1 | ---------- ---------- ---------- ---------- ---------- ---------- ---------- ---------- | 97 |
| XP_024580932.1 | ---------- ---------- ---------- ---------- ---------- ---------- ---------- ---------- | 153 |
| Consensus | ---------- ---------- ---------- ---------- ---------- ---------- ---------- ---------- | |
| Conservation (100% / 0%) | | |

| | 3,460 3,480 3,500 3,520 | |
|---|---|---|
| PvCRN11 | ---------- ---------- ---------- ---------- ---------- ---------- ---------- ---------- | 70 |
| XP_024579260.1 | ---------- ---------- ---------- ---------- ---------- ---------- ---------- ---------- | 73 |
| XP_024573634.1 | ---------- ---------- ---------- ---------- ---------- ---------- ---------- ---------- | 67 |
| PvCRN7 | ---------- ---------- ---------- ---------- ---------- ---------- ---------- ---------- | 98 |
| XP_024585265.1 | ---------- ---------- ---------- ---------- ---------- ---------- ---------- ---------- | 78 |
| PvCRN18 | ---------- ---------- ---------- ---------- ---------- ---------- ---------- ---------- | 72 |
| XP_024578414.1 | ---------- ---------- ---------- ---------- ---------- ---------- ---------- ---------- | 384 |
| XP_024579130.1 | ---------- ---------- ---------- ---------- ---------- ---------- ---------- ---------- | 93 |
| XP_024584758.1 | ---------- ---------- ---------- ---------- ---------- ---------- ---------- ---------- | 84 |
| XP_024586066.1 | ---------- ---------- ---------- ---------- ---------- ---------- ---------- ---------- | 99 |
| XP_024585870.1 | ---------- ---------- ---------- ---------- ---------- ---------- ---------- ---------- | 45 |
| PvCRN31 | ---------- ---------- ---------- ---------- ---------- ---------- ---------- ---------- | 81 |
| XP_024577278.1 | ---------- ---------- ---------- ---------- ---------- ---------- ---------- ---------- | 101 |
| XP_024585562.1 | ---------- ---------- ---------- ---------- ---------- ---------- ---------- ---------- | 6 |
| XP_024573944.1 | ---------- ---------- ---------- ---------- ---------- ---------- ---------- ---------- | 73 |
| XP_024579793.1 | ---------- ---------- ---------- ---------- ---------- ---------- ---------- ---------- | 23 |
| PvCRN15 | ---------- ---------- ---------- ---------- ---------- ---------- ---------- ---------- | 81 |
| PvCRN26 | ---------- ---------- ---------- ---------- ---------- ---------- ---------- ---------- | 80 |
| PvCRN16 | ---------- ---------- ---------- ---------- ---------- ---------- ---------- ---------- | 80 |
| PvCRN23 | ---------- ---------- ---------- ---------- ---------- ---------- ---------- ---------- | 101 |
| XP_024573063.1 | ---------- ---------- ---------- ---------- ---------- ---------- ---------- ---------- | 77 |
| PvCRN19 | ---------- ---------- ---------- ---------- ---------- ---------- ---------- ---------- | 76 |
| XP_024581625.1 | ---------- ---------- ---------- ---------- ---------- ---------- ---------- ---------- | 71 |
| XP_024583886.1 | ---------- ---------- ---------- ---------- ---------- ---------- ---------- ---------- | 71 |
| XP_024584409.1 | ---------- ---------- ---------- ---------- ---------- ---------- ---------- ---------- | 72 |
| XP_024572385.1 | ---------- ---------- ---------- ---------- ---------- ---------- ---------- ---------- | 68 |
| PvCRN6 | ---------- ---------- ---------- ---------- ---------- ---------- ---------- ---------- | 75 |
| PvCRN9 | ---------- ---------- ---------- ---------- ---------- ---------- ---------- ---------- | 71 |
| XP_024580875.1 | ---------- ---------- ---------- ---------- ---------- ---------- ---------- ---------- | 54 |
| PvCRN24 | ---------- ---------- ---------- ---------- ---------- ---------- ---------- ---------- | 77 |
| PvCRN35 | ---------- ---------- ---------- ---------- ---------- ---------- ---------- ---------- | 77 |
| PvCRN12 | ---------- ---------- ---------- ---------- ---------- ---------- ---------- ---------- | 77 |
| XP_024576927.1 | ---------- ---------- ---------- ---------- ---------- ---------- ---------- ---------- | 118 |
| XP_024578927.1 | ---------- ---------- ---------- ---------- ---------- ---------- ---------- ---------- | 76 |
| XP_024586212.1 | ---------- ---------- ---------- ---------- ---------- ---------- ---------- ---------- | 66 |
| XP_024578078.1 | ---------- ---------- ---------- ---------- ---------- ---------- ---------- ---------- | 60 |
| XP_024575355.1 | ---------- ---------- ---------- ---------- ---------- ---------- ---------- ---------- | 227 |
| XP_024584527.1 | ---------- ---------- ---------- ---------- ---------- ---------- ---------- ---------- | 78 |
| XP_024586664.1 | ---------- ---------- ---------- ---------- ---------- ---------- ---------- ---------- | 64 |
| PvCRN17 | ---------- ---------- ---------- ---------- ---------- ---------- ---------- ---------- | 77 |
| PvCRN2 | ---------- ---------- ---------- ---------- ---------- ---------- ---------- ---------- | 77 |
| XP_024577181.1 | ---------- ---------- ---------- ---------- ---------- ---------- ---------- ---------- | 39 |
| XP_024581075.1 | ---------- ---------- ---------- ---------- ---------- ---------- ---------- ---------- | 76 |
| XP_024575884.1 | ---------- ---------- ---------- ---------- ---------- ---------- ---------- ---------- | 75 |
| XP_024586872.1 | ---------- ---------- ---------- ---------- ---------- ---------- ---------- ---------- | 75 |
| XP_024573052.1 | ---------- ---------- ---------- ---------- ---------- ---------- ---------- ---------- | 98 |
| XP_024586054.1 | ---------- ---------- ---------- ---------- ---------- ---------- ---------- ---------- | 87 |
| PvCRN27 | ---------- ---------- ---------- ---------- ---------- ---------- ---------- ---------- | 72 |
| PvCRN20 | ---------- ---------- ---------- ---------- ---------- ---------- ---------- ---------- | 92 |
| PvCRN25 | ---------- ---------- ---------- ---------- ---------- ---------- ---------- ---------- | 78 |
| PvCRN1 | ---------- ---------- ---------- ---------- ---------- ---------- ---------- ---------- | 84 |
| PvCRN4 | ---------- ---------- ---------- ---------- ---------- ---------- ---------- ---------- | 79 |
| PvCRN30 | ---------- ---------- ---------- ---------- ---------- ---------- ---------- ---------- | 51 |
| PvCRN29 | ---------- ---------- ---------- ---------- ---------- ---------- ---------- ---------- | 72 |
| XP_024583036.1 | ---------- ---------- ---------- ---------- ---------- ---------- ---------- ---------- | 201 |
| PvCRN10 | ---------- ---------- ---------- ---------- ---------- ---------- ---------- ---------- | 70 |
| XP_024577280.1 | ---------- ---------- ---------- ---------- ---------- ---------- ---------- ---------- | 95 |
| XP_024572924.1 | ---------- ---------- ---------- ---------- ---------- ---------- ---------- ---------- | 172 |
| PvCRN14 | ---------- ---------- ---------- ---------- ---------- ---------- ---------- ---------- | 77 |
| XP_024577521.1 | ---------- ---------- ---------- ---------- ---------- ---------- ---------- ---------- | 55 |
| PvCRN21 | ---------- ---------- ---------- ---------- ---------- ---------- ---------- ---------- | 66 |
| XP_024574966.1 | ---------- ---------- ---------- ---------- ---------- ---------- ---------- ---------- | 76 |
| XP_024575372.1 | ---------- ---------- ---------- ---------- ---------- ---------- ---------- ---------- | 77 |
| XP_024581363.1 | ---------- ---------- ---------- ---------- ---------- ---------- ---------- ---------- | 1 |
| XP_024583883.1 | ---------- ---------- ---------- ---------- ---------- ---------- ---------- ---------- | 43 |
| XP_024579844.1 | ---------- ---------- ---------- ---------- ---------- ---------- ---------- ---------- | 118 |
| XP_024574916.1 | ---------- ---------- ---------- ---------- ---------- ---------- ---------- ---------- | 120 |
| XP_024574193.1 | ---------- ---------- ---------- ---------- ---------- ---------- ---------- ---------- | 184 |
| XP_024583155.1 | ---------- ---------- ---------- ---------- ---------- ---------- ---------- ---------- | 68 |
| XP_024574913.1 | ---------- ---------- ---------- ---------- ---------- ---------- ---------- ---------- | 71 |
| XP_024578158.1 | ---------- ---------- ---------- ---------- ---------- ---------- ---------- ---------- | 41 |
| PvCRN22 | ---------- ---------- ---------- ---------- ---------- ---------- ---------- ---------- | 71 |
| XP_024585001.1 | ---------- ---------- ---------- ---------- ---------- ---------- ---------- ---------- | 32 |
| XP_024583843.1 | ---------- ---------- ---------- ---------- ---------- ---------- ---------- ---------- | 116 |
| XP_024578702.1 | ---------- ---------- ---------- ---------- ---------- ---------- ---------- ---------- | 39 |
| XP_024585805.1 | ---------- ---------- ---------- ---------- ---------- ---------- ---------- ---------- | 97 |
| XP_024580932.1 | ---------- ---------- ---------- ---------- ---------- ---------- ---------- ---------- | 153 |
| Consensus | ---------- ---------- ---------- ---------- ---------- ---------- ---------- ---------- | |
| Conservation (100% – 0%) | | |

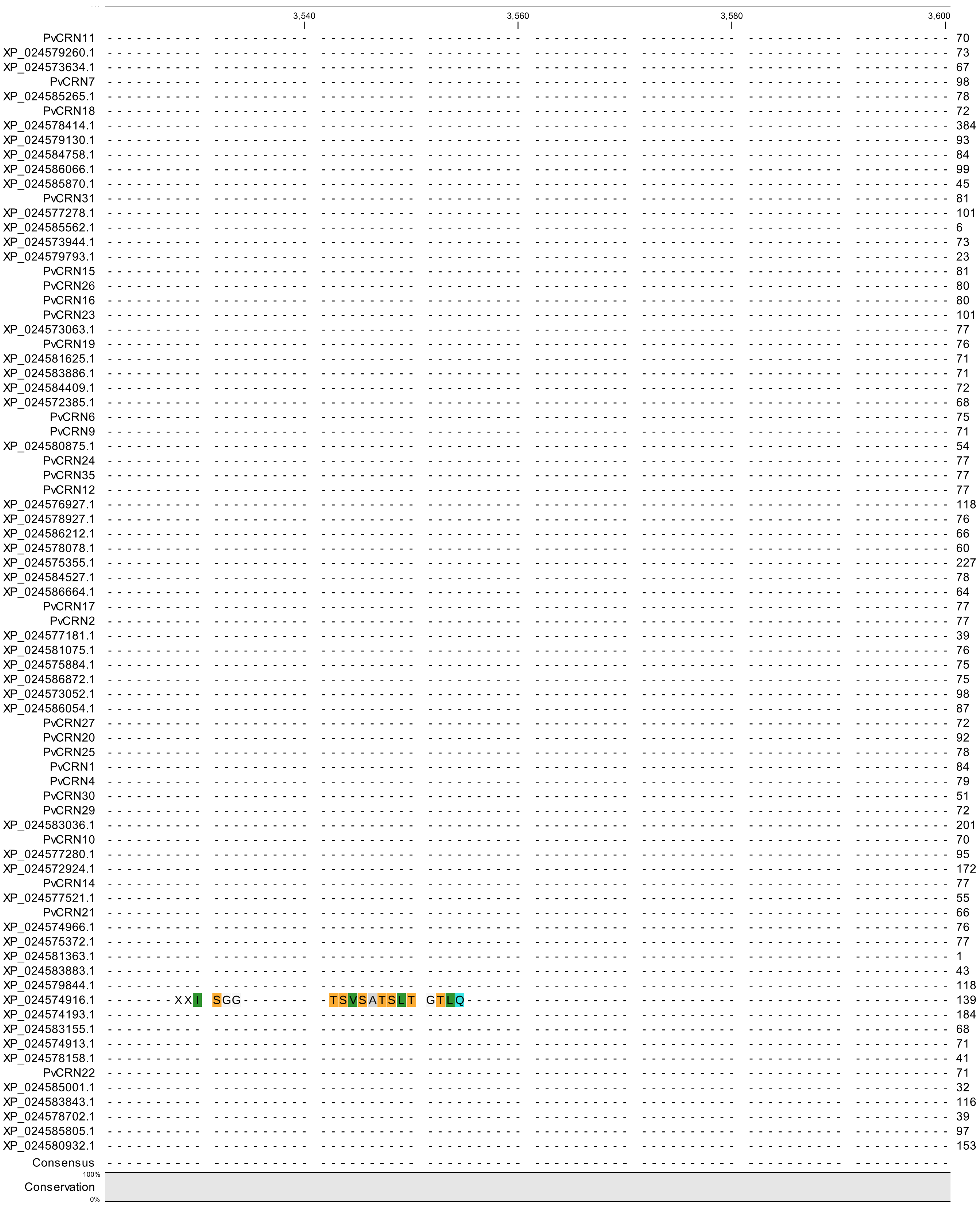
3,540
3,560
3,580
3,600
PvCRN11 70
XP_024579260.1 73
XP_024573634.1 67
PvCRN7 98
XP_024585265.1 78
PvCRN18 72
XP_024578414.1 384
XP_024579130.1 93
XP_024584758.1 84
XP_024586066.1 99
XP_024585870.1 45
PvCRN31 81
XP_024577278.1 101
XP_024585562.1 6
XP_024573944.1 73
XP_024579793.1 23
PvCRN15 81
PvCRN26 80
PvCRN16 80
PvCRN23 101
XP_024573063.1 77
PvCRN19 76
XP_024581625.1 71
XP_024583886.1 71
XP_024584409.1 72
XP_024572385.1 68
PvCRN6 75
PvCRN9 71
XP_024580875.1 54
PvCRN24 77
PvCRN35 77
PvCRN12 77
XP_024576927.1 118
XP_024578927.1 76
XP_024586212.1 66
XP_024578078.1 60
XP_024575355.1 227
XP_024584527.1 78
XP_024586664.1 64
PvCRN17 77
PvCRN2 77
XP_024577181.1 39
XP_024581075.1 76
XP_024575884.1 75
XP_024586872.1 75
XP_024573052.1 98
XP_024586054.1 87
PvCRN27 72
PvCRN20 92
PvCRN25 78
PvCRN1 84
PvCRN4 79
PvCRN30 51
PvCRN29 72
XP_024583036.1 201
PvCRN10 70
XP_024577280.1 95
XP_024572924.1 172
PvCRN14 77
XP_024577521.1 55
PvCRN21 66
XP_024574966.1 76
XP_024575372.1 77
XP_024581363.1 1
XP_024583883.1 43
XP_024579844.1 118
XP_024574916.1 XXI SGG TSVSATSLT GTLQ 139
XP_024574193.1 184
XP_024583155.1 68
XP_024574913.1 71
XP_024578158.1 41
PvCRN22 71
XP_024585001.1 32
XP_024583843.1 116
XP_024578702.1 39
XP_024585805.1 97
XP_024580932.1 153
Consensus
100%
Conservation
0%

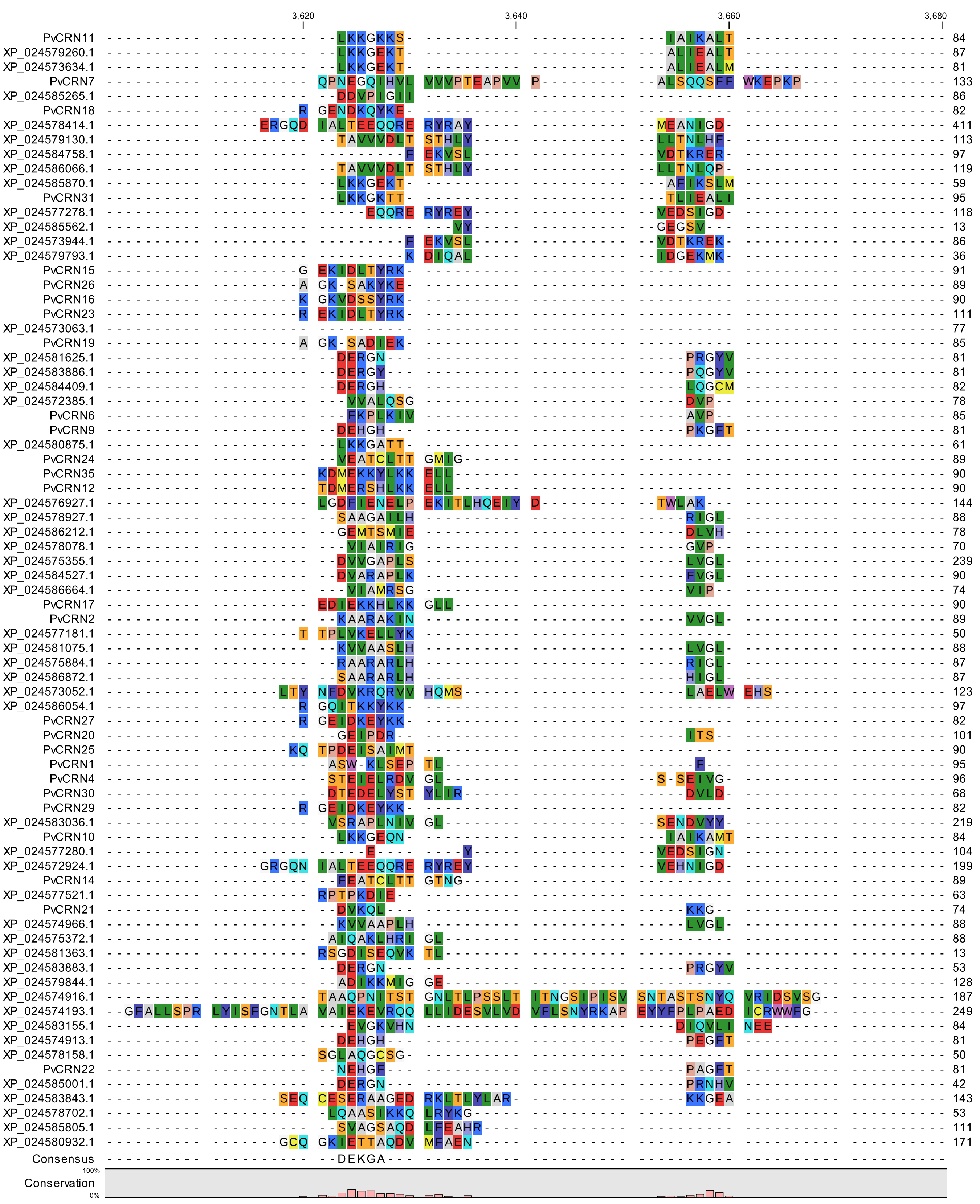

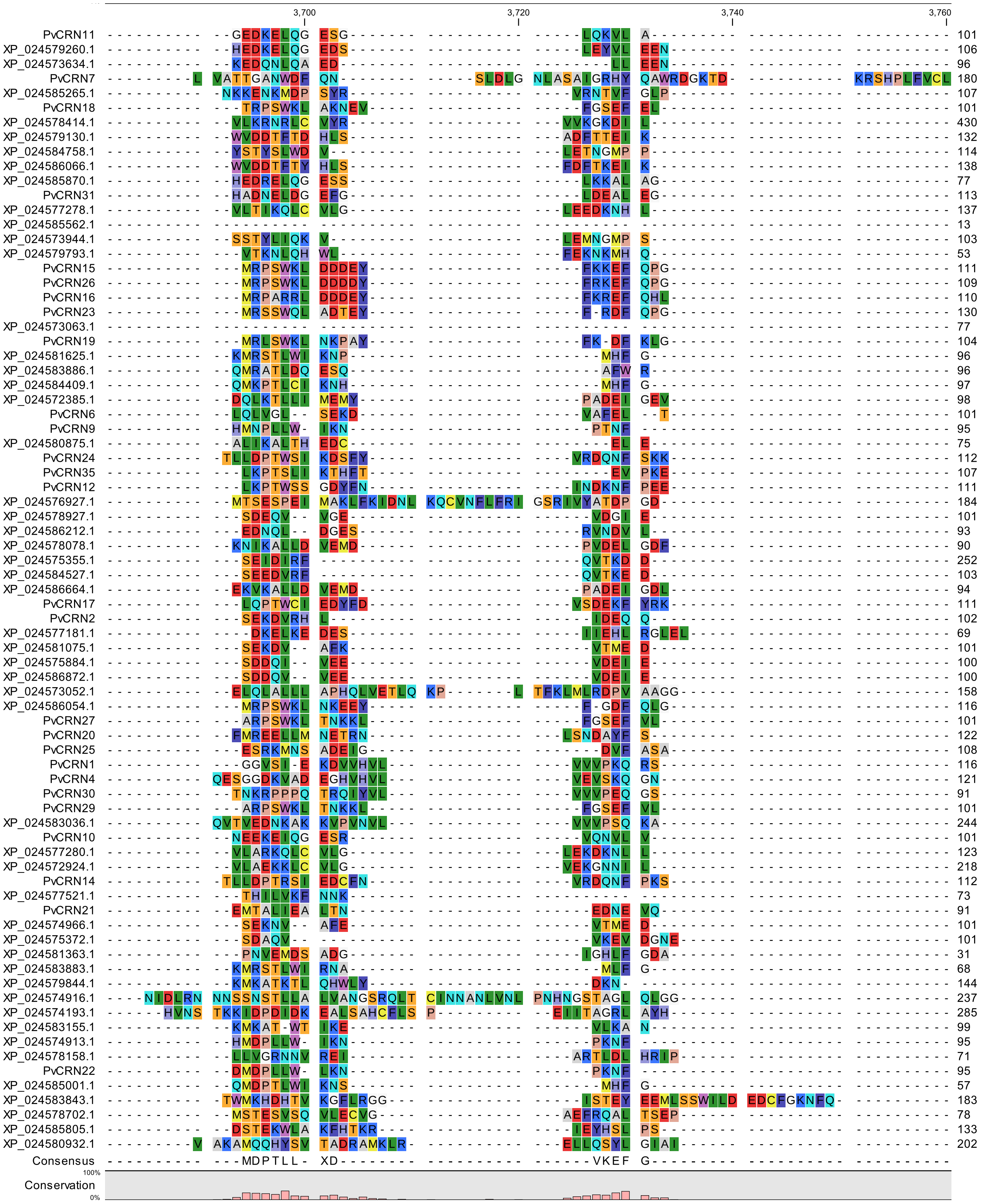
3,700
3,720
3,740
3,760
Consensus
Conservation
100%
0%

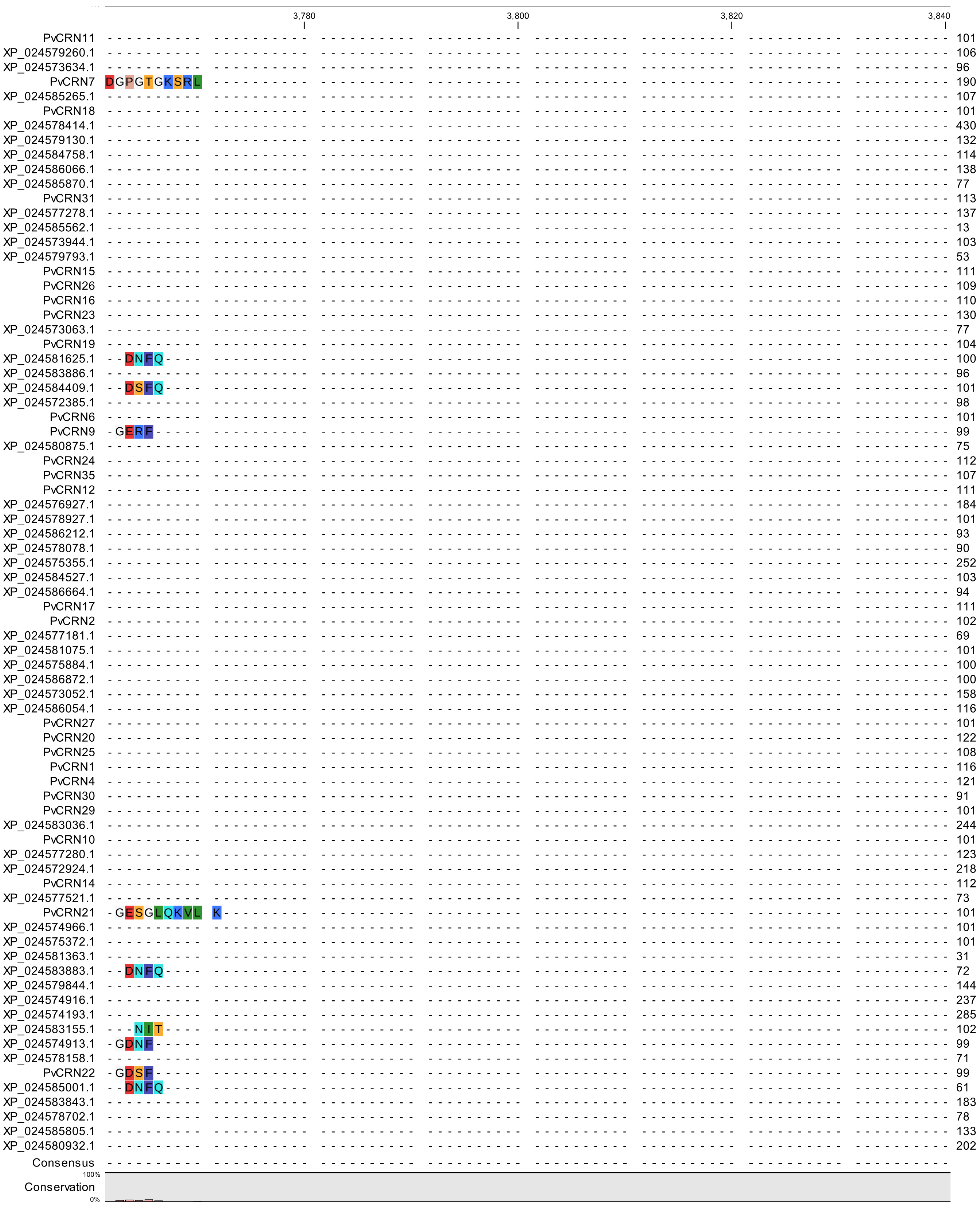

3,780
3,800
3,820
3,840
PvCRN11 101
XP_024579260.1 106
XP_024573634.1 96
PvCRN7 DGPGTGKSRL 190
XP_024585265.1 107
PvCRN18 101
XP_024578414.1 430
XP_024579130.1 132
XP_024584758.1 114
XP_024586066.1 138
XP_024585870.1 77
PvCRN31 113
XP_024577278.1 137
XP_024585562.1 13
XP_024573944.1 103
XP_024579793.1 53
PvCRN15 111
PvCRN26 109
PvCRN16 110
PvCRN23 130
XP_024573063.1 77
PvCRN19 104
XP_024581625.1 DNFQ 100
XP_024583886.1 96
XP_024584409.1 DSFQ 101
XP_024572385.1 98
PvCRN6 101
PvCRN9 GERF 99
XP_024580875.1 75
PvCRN24 112
PvCRN35 107
PvCRN12 111
XP_024576927.1 184
XP_024578927.1 101
XP_024586212.1 93
XP_024578078.1 90
XP_024575355.1 252
XP_024584527.1 103
XP_024586664.1 94
PvCRN17 111
PvCRN2 102
XP_024577181.1 69
XP_024581075.1 101
XP_024575884.1 100
XP_024586872.1 100
XP_024573052.1 158
XP_024586054.1 116
PvCRN27 101
PvCRN20 122
PvCRN25 108
PvCRN1 116
PvCRN4 121
PvCRN30 91
PvCRN29 101
XP_024583036.1 244
PvCRN10 101
XP_024577280.1 123
XP_024572924.1 218
PvCRN14 112
XP_024577521.1 73
PvCRN21 GESGLQKVL K 101
XP_024574966.1 101
XP_024575372.1 101
XP_024581363.1 31
XP_024583883.1 DNFQ 72
XP_024579844.1 144
XP_024574916.1 237
XP_024574193.1 285
XP_024583155.1 NIT 102
XP_024574913.1 GDNF 99
XP_024578158.1 71
PvCRN22 GDSF 99
XP_024585001.1 DNFQ 61
XP_024583843.1 183
XP_024578702.1 78
XP_024585805.1 133
XP_024580932.1 202
Consensus
Conservation
100%
0%

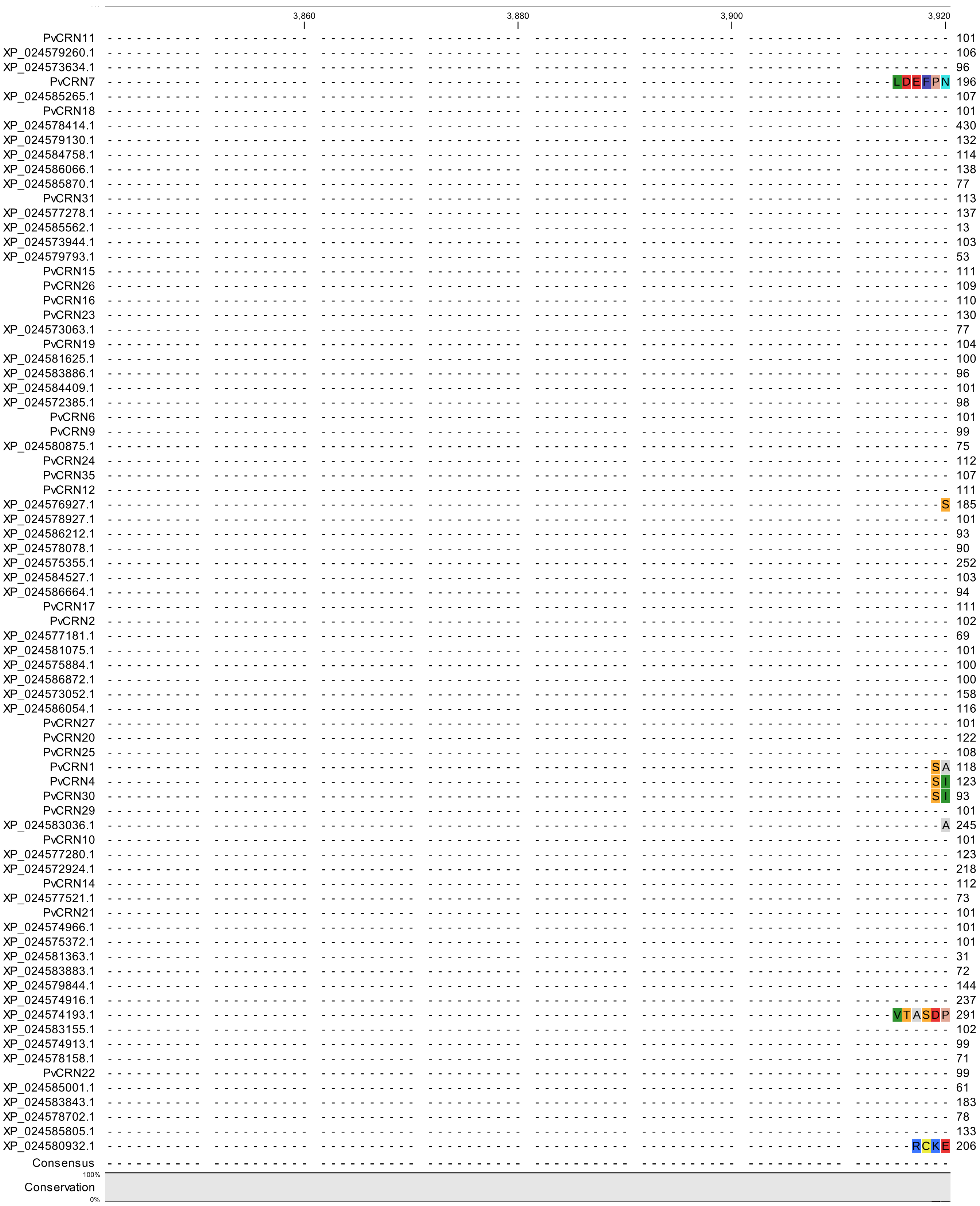

3,860
3,880
3,900
3,920
PvCRN11 101
XP_024579260.1 106
XP_024573634.1 96
PvCRN7 LDEFPN 196
XP_024585265.1 107
PvCRN18 101
XP_024578414.1 430
XP_024579130.1 132
XP_024584758.1 114
XP_024586066.1 138
XP_024585870.1 77
PvCRN31 113
XP_024577278.1 137
XP_024585562.1 13
XP_024573944.1 103
XP_024579793.1 53
PvCRN15 111
PvCRN26 109
PvCRN16 110
PvCRN23 130
XP_024573063.1 77
PvCRN19 104
XP_024581625.1 100
XP_024583886.1 96
XP_024584409.1 101
XP_024572385.1 98
PvCRN6 101
PvCRN9 99
XP_024580875.1 75
PvCRN24 112
PvCRN35 107
PvCRN12 111
XP_024576927.1 S 185
XP_024578927.1 101
XP_024586212.1 93
XP_024578078.1 90
XP_024575355.1 252
XP_024584527.1 103
XP_024586664.1 94
PvCRN17 111
PvCRN2 102
XP_024577181.1 69
XP_024581075.1 101
XP_024575884.1 100
XP_024586872.1 100
XP_024573052.1 158
XP_024586054.1 116
PvCRN27 101
PvCRN20 122
PvCRN25 108
PvCRN1 SA 118
PvCRN4 SI 123
PvCRN30 SI 93
PvCRN29 101
XP_024583036.1 A 245
PvCRN10 101
XP_024577280.1 123
XP_024572924.1 218
PvCRN14 112
XP_024577521.1 73
PvCRN21 101
XP_024574966.1 101
XP_024575372.1 101
XP_024581363.1 31
XP_024583883.1 72
XP_024579844.1 144
XP_024574916.1 237
XP_024574193.1 VTASDP 291
XP_024583155.1 102
XP_024574913.1 99
XP_024578158.1 71
PvCRN22 99
XP_024585001.1 61
XP_024583843.1 183
XP_024578702.1 78
XP_024585805.1 133
XP_024580932.1 RCKE 206
Consensus
100%
Conservation
0%

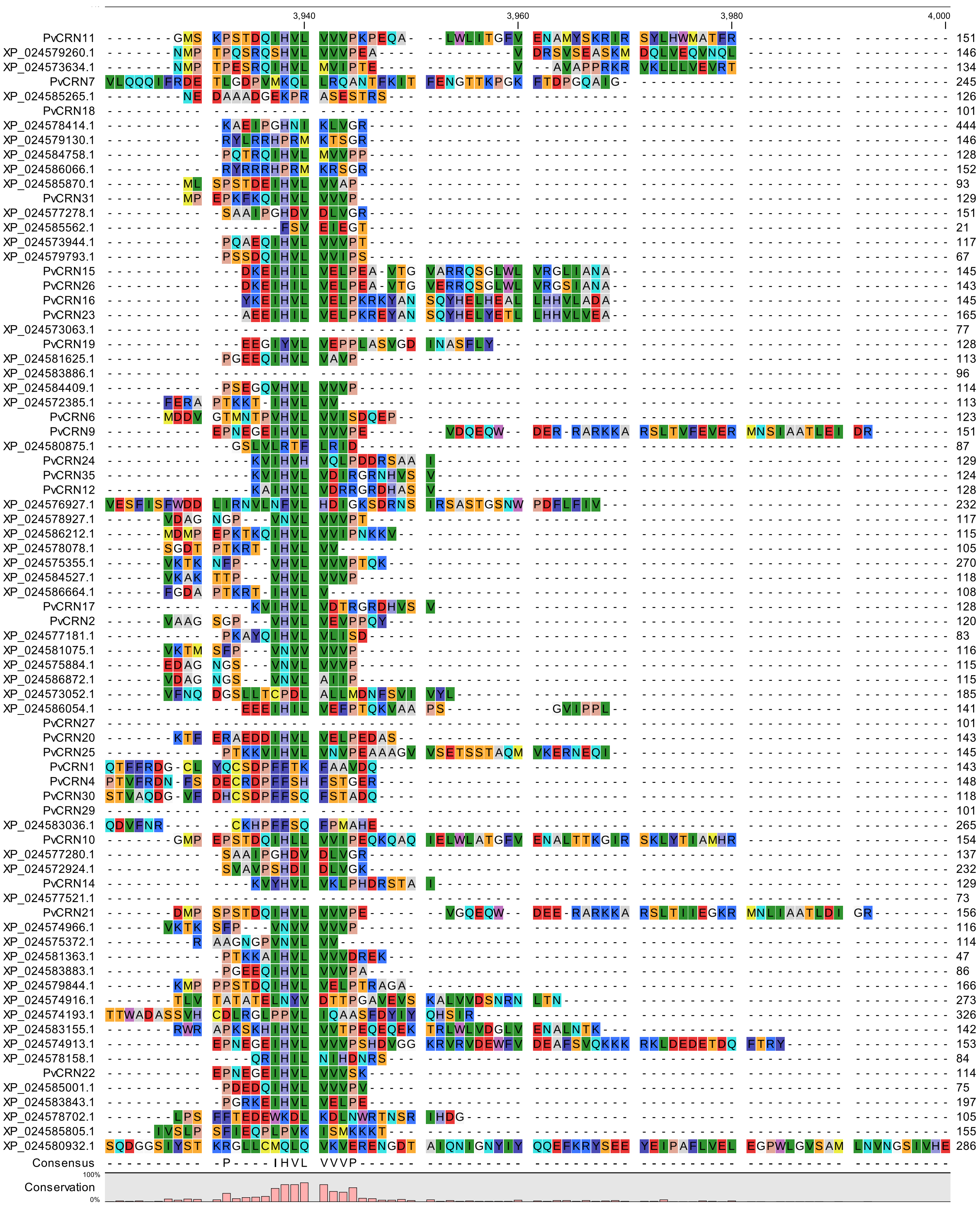

3,940
3,960
3,980
4,000
PvCRN11 151
XP_024579260.1 146
XP_024573634.1 134
PvCRN7 245
XP_024585265.1 126
PvCRN18 101
XP_024578414.1 444
XP_024579130.1 146
XP_024584758.1 128
XP_024586066.1 152
XP_024585870.1 93
PvCRN31 129
XP_024577278.1 151
XP_024585562.1 21
XP_024573944.1 117
XP_024579793.1 67
PvCRN15 145
PvCRN26 143
PvCRN16 145
PvCRN23 165
XP_024573063.1 77
PvCRN19 128
XP_024581625.1 113
XP_024583886.1 96
XP_024584409.1 114
XP_024572385.1 113
PvCRN6 123
PvCRN9 151
XP_024580875.1 87
PvCRN24 129
PvCRN35 124
PvCRN12 128
XP_024576927.1 232
XP_024578927.1 117
XP_024586212.1 115
XP_024578078.1 105
XP_024575355.1 270
XP_024584527.1 118
XP_024586664.1 108
PvCRN17 128
PvCRN2 120
XP_024577181.1 83
XP_024581075.1 116
XP_024575884.1 115
XP_024586872.1 115
XP_024573052.1 185
XP_024586054.1 141
PvCRN27 101
PvCRN20 143
PvCRN25 145
PvCRN1 143
PvCRN4 148
PvCRN30 118
PvCRN29 101
XP_024583036.1 265
PvCRN10 154
XP_024577280.1 137
XP_024572924.1 232
PvCRN14 129
XP_024577521.1 73
PvCRN21 156
XP_024574966.1 116
XP_024575372.1 114
XP_024581363.1 47
XP_024583883.1 86
XP_024579844.1 166
XP_024574916.1 273
XP_024574193.1 326
XP_024583155.1 142
XP_024574913.1 153
XP_024578158.1 84
PvCRN22 114
XP_024585001.1 75
XP_024583843.1 197
XP_024578702.1 105
XP_024585805.1 155
XP_024580932.1 286
Consensus
- P - - - - I H V L   V V V P
Conservation
100%
0%

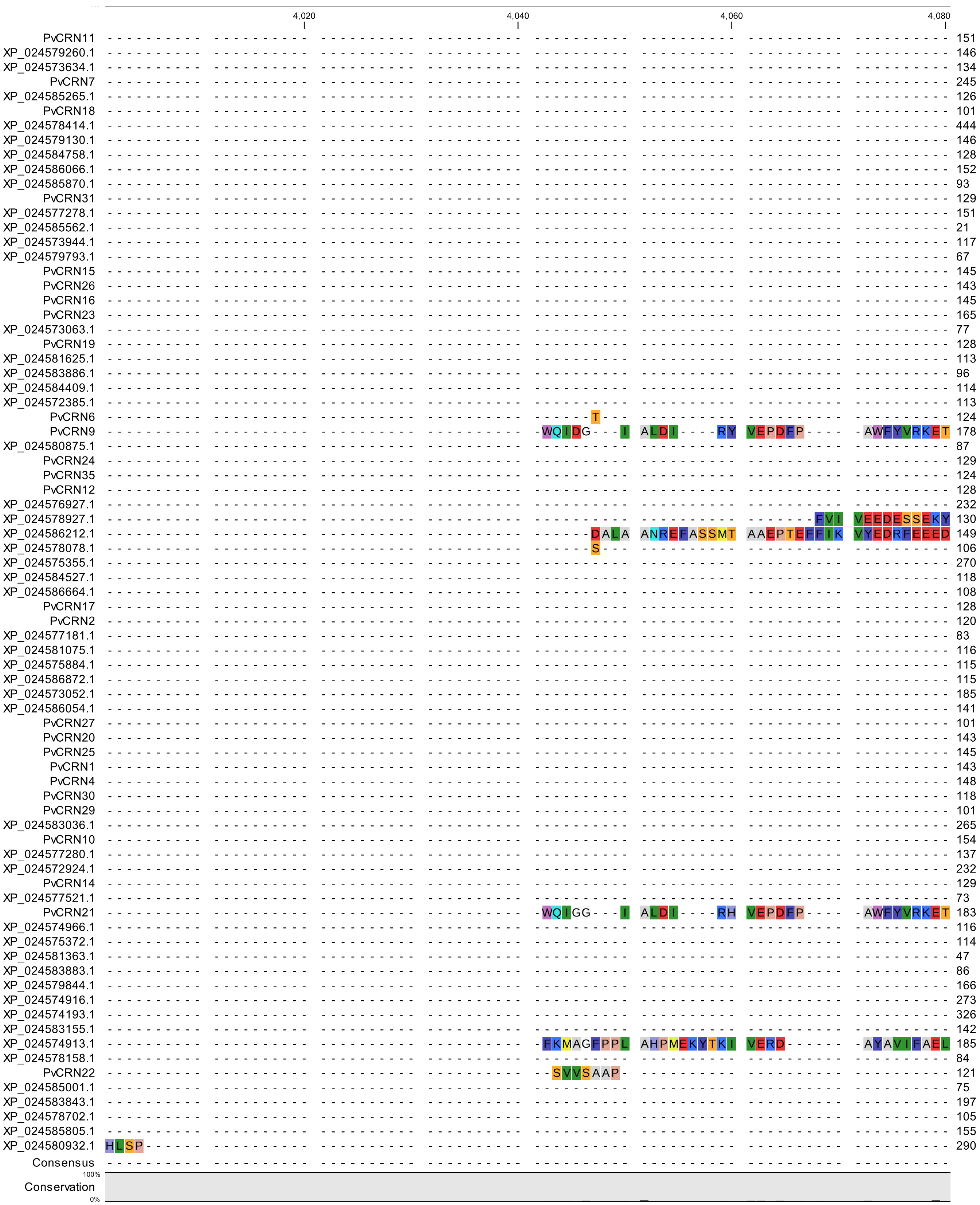
4,020
4,040
4,060
4,080
PvCRN11 151
XP_024579260.1 146
XP_024573634.1 134
PvCRN7 245
XP_024585265.1 126
PvCRN18 101
XP_024578414.1 444
XP_024579130.1 146
XP_024584758.1 128
XP_024586066.1 152
XP_024585870.1 93
PvCRN31 129
XP_024577278.1 151
XP_024585562.1 21
XP_024573944.1 117
XP_024579793.1 67
PvCRN15 145
PvCRN26 143
PvCRN16 145
PvCRN23 165
XP_024573063.1 77
PvCRN19 128
XP_024581625.1 113
XP_024583886.1 96
XP_024584409.1 114
XP_024572385.1 113
PvCRN6 T 124
PvCRN9 WQIDG I ALDI RY VEPDFP AWFYVRKET 178
XP_024580875.1 87
PvCRN24 129
PvCRN35 124
PvCRN12 128
XP_024576927.1 232
XP_024578927.1 FVI VEEDESSEKY 130
XP_024586212.1 DALA ANREFASSMT AAEPTEFFIK VYEDRFEEED 149
XP_024578078.1 S 106
XP_024575355.1 270
XP_024584527.1 118
XP_024586664.1 108
PvCRN17 128
PvCRN2 120
XP_024577181.1 83
XP_024581075.1 116
XP_024575884.1 115
XP_024586872.1 115
XP_024573052.1 185
XP_024586054.1 141
PvCRN27 101
PvCRN20 143
PvCRN25 145
PvCRN1 143
PvCRN4 148
PvCRN30 118
PvCRN29 101
XP_024583036.1 265
PvCRN10 154
XP_024577280.1 137
XP_024572924.1 232
PvCRN14 129
XP_024577521.1 73
PvCRN21 WQIGG I ALDI RH VEPDFP AWFYVRKET 183
XP_024574966.1 116
XP_024575372.1 114
XP_024581363.1 47
XP_024583883.1 86
XP_024579844.1 166
XP_024574916.1 273
XP_024574193.1 326
XP_024583155.1 142
XP_024574913.1 FKMAGFPPL AHPMEKYTKI VERD AYAVIFAEL 185
XP_024578158.1 84
PvCRN22 SVVSAAP 121
XP_024585001.1 75
XP_024583843.1 197
XP_024578702.1 105
XP_024585805.1 155
XP_024580932.1 HLSP 290
Consensus
100%
Conservation
0%

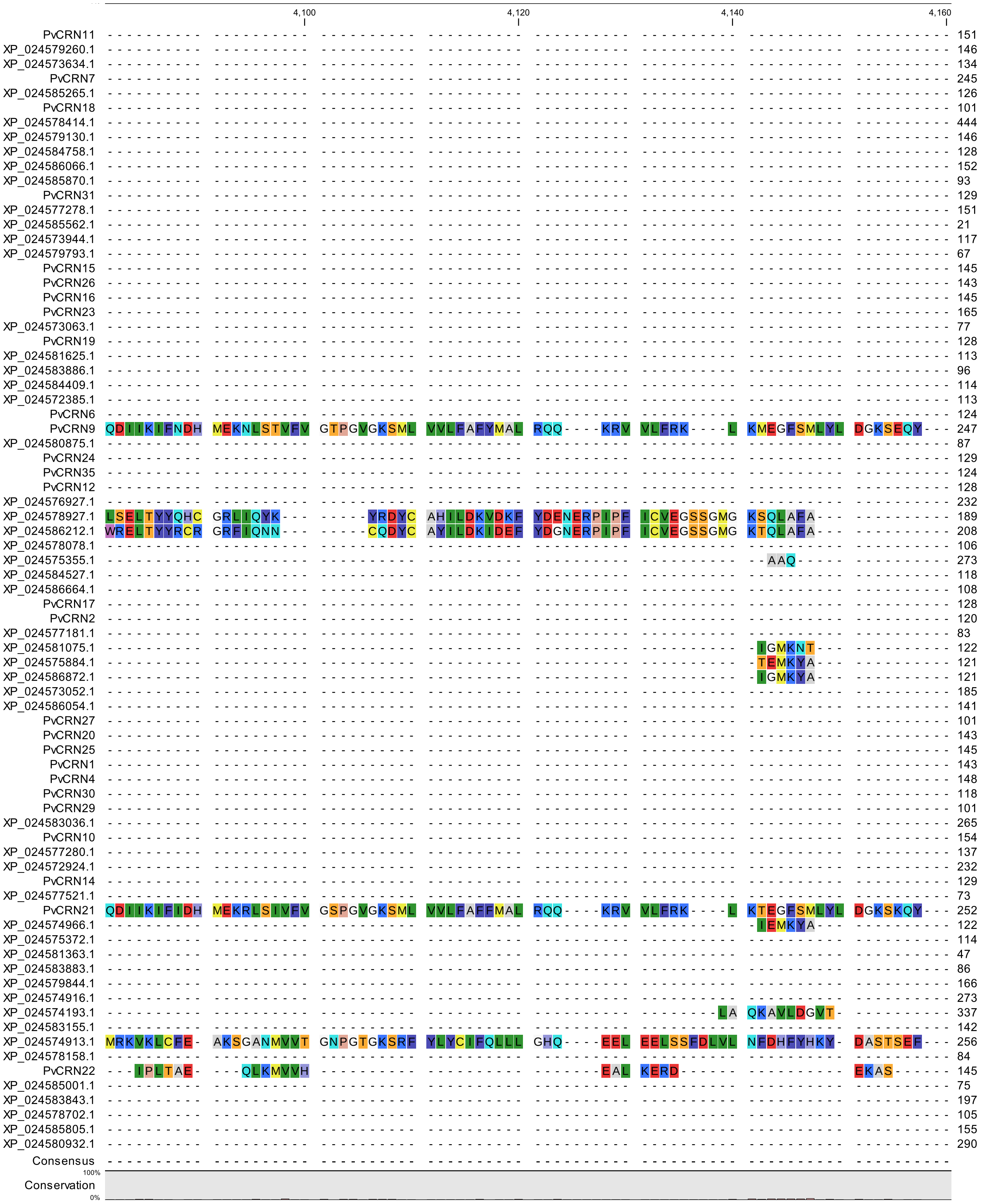
4,100
4,120
4,140
4,160
PvCRN11 151
XP_024579260.1 146
XP_024573634.1 134
PvCRN7 245
XP_024585265.1 126
PvCRN18 101
XP_024578414.1 444
XP_024579130.1 146
XP_024584758.1 128
XP_024586066.1 152
XP_024585870.1 93
PvCRN31 129
XP_024577278.1 151
XP_024585562.1 21
XP_024573944.1 117
XP_024579793.1 67
PvCRN15 145
PvCRN26 143
PvCRN16 145
PvCRN23 165
XP_024573063.1 77
PvCRN19 128
XP_024581625.1 113
XP_024583886.1 96
XP_024584409.1 114
XP_024572385.1 113
PvCRN6 124
PvCRN9 QDIIKIFNDH MEKNLSTVFV GTPGVGKSML VVLFAFYMAL RQQ---KRV VLFRK----L KMEGFSMLYL DGKSEQY--- 247
XP_024580875.1 87
PvCRN24 129
PvCRN35 124
PvCRN12 128
XP_024576927.1 232
XP_024578927.1 LSELTYYQHC GRLIQYK--- -----YRDYC AHILDKVDKF YDENERPIPF ICVEGSSGMG KSQLAFA--- 189
XP_024586212.1 WRELTYYRCR GRFIQNN--- -----CQDYC AYILDKIDEF YDGNERPIPF ICVEGSSGMG KTQLAFA--- 208
XP_024578078.1 106
XP_024575355.1 --AAQ----- 273
XP_024584527.1 118
XP_024586664.1 108
PvCRN17 128
PvCRN2 120
XP_024577181.1 83
XP_024581075.1 -IGMKNT--- 122
XP_024575884.1 -TEMKYA--- 121
XP_024586872.1 -IGMKYA--- 121
XP_024573052.1 185
XP_024586054.1 141
PvCRN27 101
PvCRN20 143
PvCRN25 145
PvCRN1 143
PvCRN4 148
PvCRN30 118
PvCRN29 101
XP_024583036.1 265
PvCRN10 154
XP_024577280.1 137
XP_024572924.1 232
PvCRN14 129
XP_024577521.1 73
PvCRN21 QDIIKIFIDH MEKRLSIVFV GSPGVGKSML VVLFAFFMAL RQQ---KRV VLFRK----L KTEGFSMLYL DGKSKQY--- 252
XP_024574966.1 -IEMKYA--- 122
XP_024575372.1 114
XP_024581363.1 47
XP_024583883.1 86
XP_024579844.1 166
XP_024574916.1 273
XP_024574193.1 --------LA QKAVLDGVT- 337
XP_024583155.1 142
XP_024574913.1 MRKVKLCFE- AKSGANMVVT GNPGTGKSRF YLYCIFQLLL GHQ---EEL EELSSFDLVL NFDHFYHKY- DASTSEF--- 256
XP_024578158.1 84
PvCRN22 ---IPLTAE- ---QLKMVVH ------EAL KERD------ EKAS------ 145
XP_024585001.1 75
XP_024583843.1 197
XP_024578702.1 105
XP_024585805.1 155
XP_024580932.1 290
Consensus
100%
Conservation
0%

| | 4,180 / 4,200 / 4,220 / 4,240 | |
|---|---|---|
| PvCRN11 | ---------- ---------- ---------- ---------- ---------- ---------- ---------- ---------- | 151 |
| XP_024579260.1 | ---------- ---------- ---------- ---------- ---------- ---------- ---------- ---------- | 146 |
| XP_024573634.1 | ---------- ---------- ---------- ---------- ---------- ---------- ---------- ---------- | 134 |
| PvCRN7 | ---------- ---------- ---------- ---------- ---------- ---------- ---------- ---------- | 245 |
| XP_024585265.1 | ---------- ---------- ---------- ---------- ---------- ---------- ---------- ---------- | 126 |
| PvCRN18 | ---------- ---------- ---------- ---------- ---------- ---------- ---------- ---------- | 101 |
| XP_024578414.1 | ---------- ---------- ---------- ---------- ---------- ---------- ---------- ---------- | 444 |
| XP_024579130.1 | ---------- ---------- ---------- ---------- ---------- ---------- ---------- ---------- | 146 |
| XP_024584758.1 | ---------- ---------- ---------- ---------- ---------- ---------- ---------- ---------- | 128 |
| XP_024586066.1 | ---------- ---------- ---------- ---------- ---------- ---------- ---------- ---------- | 152 |
| XP_024585870.1 | ---------- ---------- ---------- ---------- ---------- ---------- ---------- ---------- | 93 |
| PvCRN31 | ---------- ---------- ---------- ---------- ---------- ---------- ---------- ---------- | 129 |
| XP_024577278.1 | ---------- ---------- ---------- ---------- ---------- ---------- ---------- ---------- | 151 |
| XP_024585562.1 | ---------- ---------- ---------- ---------- ---------- ---------- ---------- ---------- | 21 |
| XP_024573944.1 | ---------- ---------- ---------- ---------- ---------- ---------- ---------- ---------- | 117 |
| XP_024579793.1 | ---------- ---------- ---------- ---------- ---------- ---------- ---------- ---------- | 67 |
| PvCRN15 | ---------- ---------- ---------- ---------- ---------- ---------- ---------- ---------- | 145 |
| PvCRN26 | ---------- ---------- ---------- ---------- ---------- ---------- ---------- ---------- | 143 |
| PvCRN16 | ---------- ---------- ---------- ---------- ---------- ---------- ---------- ---------- | 145 |
| PvCRN23 | ---------- ---------- ---------- ---------- ---------- ---------- ---------- ---------- | 165 |
| XP_024573063.1 | ---------- ---------- ---------- ---------- ---------- ---------- ---------- ---------- | 77 |
| PvCRN19 | ---------- ---------- ---------- ---------- ---------- ---------- ---------- ---------- | 128 |
| XP_024581625.1 | ---------- ---------- ---------- ---------- ---------- ---------- ---------- ---------- | 113 |
| XP_024583886.1 | ---------- ---------- ---------- ---------- ---------- ---------- ---------- ---------- | 96 |
| XP_024584409.1 | ---------- ---------- ---------- ---------- ---------- ---------- ---------- ---------- | 114 |
| XP_024572385.1 | ---------- ---------- ---------- ---------- ---------- ---------- ---------- ---------- | 113 |
| PvCRN6 | ---------- ---------- ---------- ---------- ---------- ---------- ---------- ---------- | 124 |
| PvCRN9 | --WRSN---- ---------- ---------- ---------- ---------- ---------- ---------- ---------- | 251 |
| XP_024580875.1 | ---------- ---------- ---------- ---------- ---------- ---------- ---------- ---------- | 87 |
| PvCRN24 | ---------- ---------- ---------- ---------- ---------- ---------- ---------- ---------- | 129 |
| PvCRN35 | ---------- ---------- ---------- ---------- ---------- ---------- ---------- ---------- | 124 |
| PvCRN12 | ---------- ---------- ---------- ---------- ---------- ---------- ---------- ---------- | 128 |
| XP_024576927.1 | ---------- ---------- ---------- ---------- ---------- ---------- ---------- ---------- | 232 |
| XP_024578927.1 | ---------- ---------- ---------- ---------- ---------- ---------- ---------- ---------- | 189 |
| XP_024586212.1 | ---------- ---------- ---------- ---------- ---------- ---------- ---------- ---------- | 208 |
| XP_024578078.1 | ---------- ---------- ---------- ---------- ---------- ---------- ---------- ---------- | 106 |
| XP_024575355.1 | ---------- ---------- ---------- ---------- ---------- ---------- ---------- ---------- | 273 |
| XP_024584527.1 | ---------- ---------- ---------- ---------- ---------- ---------- ---------- ---------- | 118 |
| XP_024586664.1 | ---------- ---------- ---------- ---------- ---------- ---------- ---------- ---------- | 108 |
| PvCRN17 | ---------- ---------- ---------- ---------- ---------- ---------- ---------- ---------- | 128 |
| PvCRN2 | ---------- ---------- ---------- ---------- ---------- ---------- ---------- ---------- | 120 |
| XP_024577181.1 | ---------- ---------- ---------- ---------- ---------- ---------- ---------- ---------- | 83 |
| XP_024581075.1 | ---------- ---------- ---------- ---------- ---------- ---------- ---------- ---------- | 122 |
| XP_024575884.1 | ---------- ---------- ---------- ---------- ---------- ---------- ---------- ---------- | 121 |
| XP_024586872.1 | ---------- ---------- ---------- ---------- ---------- ---------- ---------- ---------- | 121 |
| XP_024573052.1 | ---------- ---------- ---------- ---------- ---------- ---------- ---------- ---------- | 185 |
| XP_024586054.1 | ---------- ---------- ---------- ---------- ---------- ---------- ---------- ---------- | 141 |
| PvCRN27 | ---------- ---------- ---------- ---------- ---------- ---------- ---------- ---------- | 101 |
| PvCRN20 | ---------- ---------- ---------- ---------- ---------- ---------- ---------- ---------- | 143 |
| PvCRN25 | ---------- ---------- ---------- ---------- ---------- ---------- ---------- ---------- | 145 |
| PvCRN1 | ---------- ---------- ---------- ---------- ---------- ---------- ---------- ---------- | 143 |
| PvCRN4 | ---------- ---------- ---------- ---------- ---------- ---------- ---------- ---------- | 148 |
| PvCRN30 | ---------- ---------- ---------- ---------- ---------- ---------- ---------- ---------- | 118 |
| PvCRN29 | ---------- ---------- ---------- ---------- ---------- ---------- ---------- ---------- | 101 |
| XP_024583036.1 | ---------- ---------- ---------- ---------- ---------- ---------- ---------- ---------- | 265 |
| PvCRN10 | ---------- ---------- ---------- ---------- ---------- ---------- ---------- ---------- | 154 |
| XP_024577280.1 | ---------- ---------- ---------- ---------- ---------- ---------- ---------- ---------- | 137 |
| XP_024572924.1 | ---------- ---------- ---------- ---------- ---------- ---------- ---------- ---------- | 232 |
| PvCRN14 | ---------- ---------- ---------- ---------- ---------- ---------- ---------- ---------- | 129 |
| XP_024577521.1 | ---------- ---------- ---------- ---------- ---------- ---------- ---------- ---------- | 73 |
| PvCRN21 | --WRSN---- ---------- ---------- ---------- ---------- ---------- ---------- ---------- | 256 |
| XP_024574966.1 | ---------- ---------- ---------- ---------- ---------- ---------- ---------- ---------- | 122 |
| XP_024575372.1 | ---------- ---------- ---------- ---------- ---------- ---------- ---------- ---------- | 114 |
| XP_024581363.1 | ---------- ---------- ---------- ---------- ---------- ---------- ---------- ---------- | 47 |
| XP_024583883.1 | ---------- ---------- ---------- ---------- ---------- ---------- ---------- ---------- | 86 |
| XP_024579844.1 | ---------- ---------- ---------- ---------- ---------- ---------- ---------- ---------- | 166 |
| XP_024574916.1 | ---------- ---------- ---------- ---------- ---------- LGKVNFTSSD ATQPTG---- ---------- | 289 |
| XP_024574193.1 | ---------- ---------- ---------- ---------- ---------- ---------- ---------- ---------- | 337 |
| XP_024583155.1 | ---------- ---------- ---------- ---------- ---------- ---------- ---------- ---------- | 142 |
| XP_024574913.1 | --LALN---- ---------- ---------- ---------- ---------- ---------- ---------- ---------- | 260 |
| XP_024578158.1 | ---------- ---------- ---------- ---------- ---------- ---------- ---------- ---------- | 84 |
| PvCRN22 | ---------- ---------- ---------- ---------- ---------- ---------- ---------- ---------- | 145 |
| XP_024585001.1 | ---------- ---------- ---------- ---------- ---------- ---------- ---------- ---------- | 75 |
| XP_024583843.1 | ---------- ---------- ---------- ---------- ---------- ---------- ---------- ---------- | 197 |
| XP_024578702.1 | ---------- ---------- ---------- ---------- ---------- ---------- ---------- ---------- | 105 |
| XP_024585805.1 | ---------- ---------- ---------- ---------- ---------- ---------- ---------- ---------- | 155 |
| XP_024580932.1 | ---------- ---------- ---------- ---------- ---------- ---------- ---------- ---------- | 290 |
| Consensus | ---------- ---------- ---------- ---------- ---------- ---------- ---------- ---------- | |

Conservation (100% / 0%)

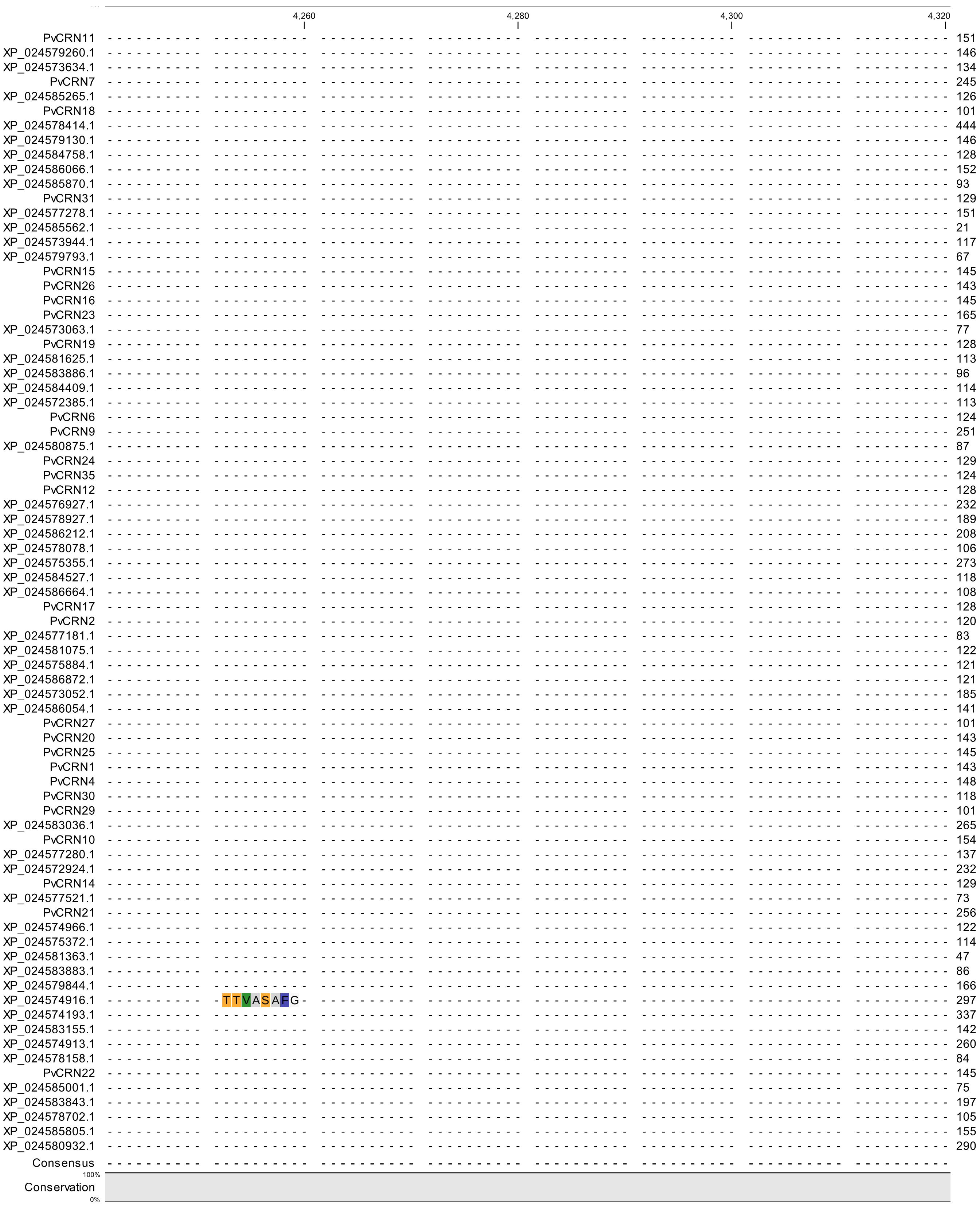
4,260
4,280
4,300
4,320
PvCRN11 151
XP_024579260.1 146
XP_024573634.1 134
PvCRN7 245
XP_024585265.1 126
PvCRN18 101
XP_024578414.1 444
XP_024579130.1 146
XP_024584758.1 128
XP_024586066.1 152
XP_024585870.1 93
PvCRN31 129
XP_024577278.1 151
XP_024585562.1 21
XP_024573944.1 117
XP_024579793.1 67
PvCRN15 145
PvCRN26 143
PvCRN16 145
PvCRN23 165
XP_024573063.1 77
PvCRN19 128
XP_024581625.1 113
XP_024583886.1 96
XP_024584409.1 114
XP_024572385.1 113
PvCRN6 124
PvCRN9 251
XP_024580875.1 87
PvCRN24 129
PvCRN35 124
PvCRN12 128
XP_024576927.1 232
XP_024578927.1 189
XP_024586212.1 208
XP_024578078.1 106
XP_024575355.1 273
XP_024584527.1 118
XP_024586664.1 108
PvCRN17 128
PvCRN2 120
XP_024577181.1 83
XP_024581075.1 122
XP_024575884.1 121
XP_024586872.1 121
XP_024573052.1 185
XP_024586054.1 141
PvCRN27 101
PvCRN20 143
PvCRN25 145
PvCRN1 143
PvCRN4 148
PvCRN30 118
PvCRN29 101
XP_024583036.1 265
PvCRN10 154
XP_024577280.1 137
XP_024572924.1 232
PvCRN14 129
XP_024577521.1 73
PvCRN21 256
XP_024574966.1 122
XP_024575372.1 114
XP_024581363.1 47
XP_024583883.1 86
XP_024579844.1 166
XP_024574916.1 -TTVASAFG- 297
XP_024574193.1 337
XP_024583155.1 142
XP_024574913.1 260
XP_024578158.1 84
PvCRN22 145
XP_024585001.1 75
XP_024583843.1 197
XP_024578702.1 105
XP_024585805.1 155
XP_024580932.1 290
Consensus
100%
Conservation
0%

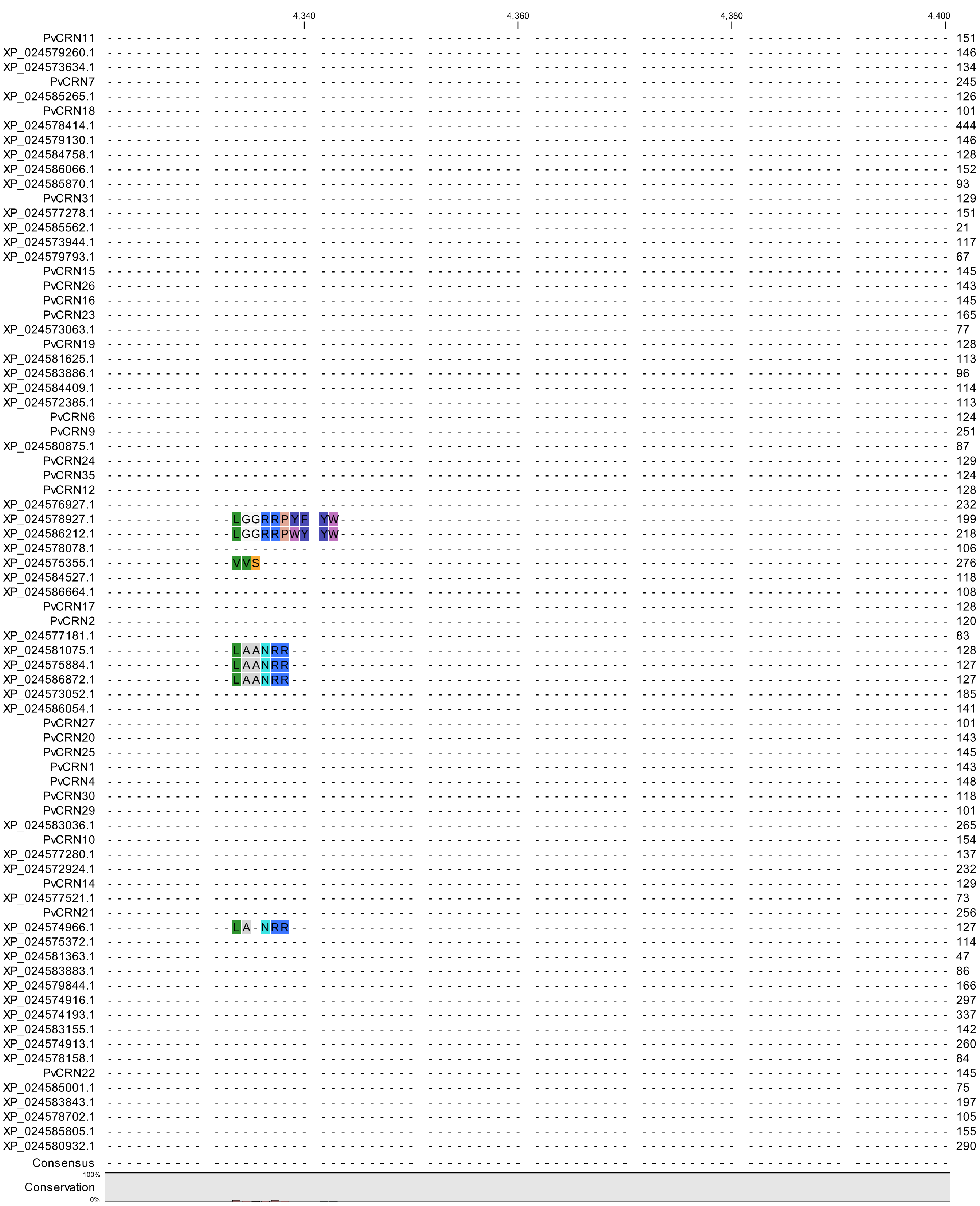
4,340
4,360
4,380
4,400
PvCRN11 151
XP_024579260.1 146
XP_024573634.1 134
PvCRN7 245
XP_024585265.1 126
PvCRN18 101
XP_024578414.1 444
XP_024579130.1 146
XP_024584758.1 128
XP_024586066.1 152
XP_024585870.1 93
PvCRN31 129
XP_024577278.1 151
XP_024585562.1 21
XP_024573944.1 117
XP_024579793.1 67
PvCRN15 145
PvCRN26 143
PvCRN16 145
PvCRN23 165
XP_024573063.1 77
PvCRN19 128
XP_024581625.1 113
XP_024583886.1 96
XP_024584409.1 114
XP_024572385.1 113
PvCRN6 124
PvCRN9 251
XP_024580875.1 87
PvCRN24 129
PvCRN35 124
PvCRN12 128
XP_024576927.1 232
XP_024578927.1 LGGRRPYF YW 199
XP_024586212.1 LGGRRPWY YW 218
XP_024578078.1 106
XP_024575355.1 VVS 276
XP_024584527.1 118
XP_024586664.1 108
PvCRN17 128
PvCRN2 120
XP_024577181.1 83
XP_024581075.1 LAANRR 128
XP_024575884.1 LAANRR 127
XP_024586872.1 LAANRR 127
XP_024573052.1 185
XP_024586054.1 141
PvCRN27 101
PvCRN20 143
PvCRN25 145
PvCRN1 143
PvCRN4 148
PvCRN30 118
PvCRN29 101
XP_024583036.1 265
PvCRN10 154
XP_024577280.1 137
XP_024572924.1 232
PvCRN14 129
XP_024577521.1 73
PvCRN21 256
XP_024574966.1 LA-NRR 127
XP_024575372.1 114
XP_024581363.1 47
XP_024583883.1 86
XP_024579844.1 166
XP_024574916.1 297
XP_024574193.1 337
XP_024583155.1 142
XP_024574913.1 260
XP_024578158.1 84
PvCRN22 145
XP_024585001.1 75
XP_024583843.1 197
XP_024578702.1 105
XP_024585805.1 155
XP_024580932.1 290
Consensus
Conservation
100%
0%

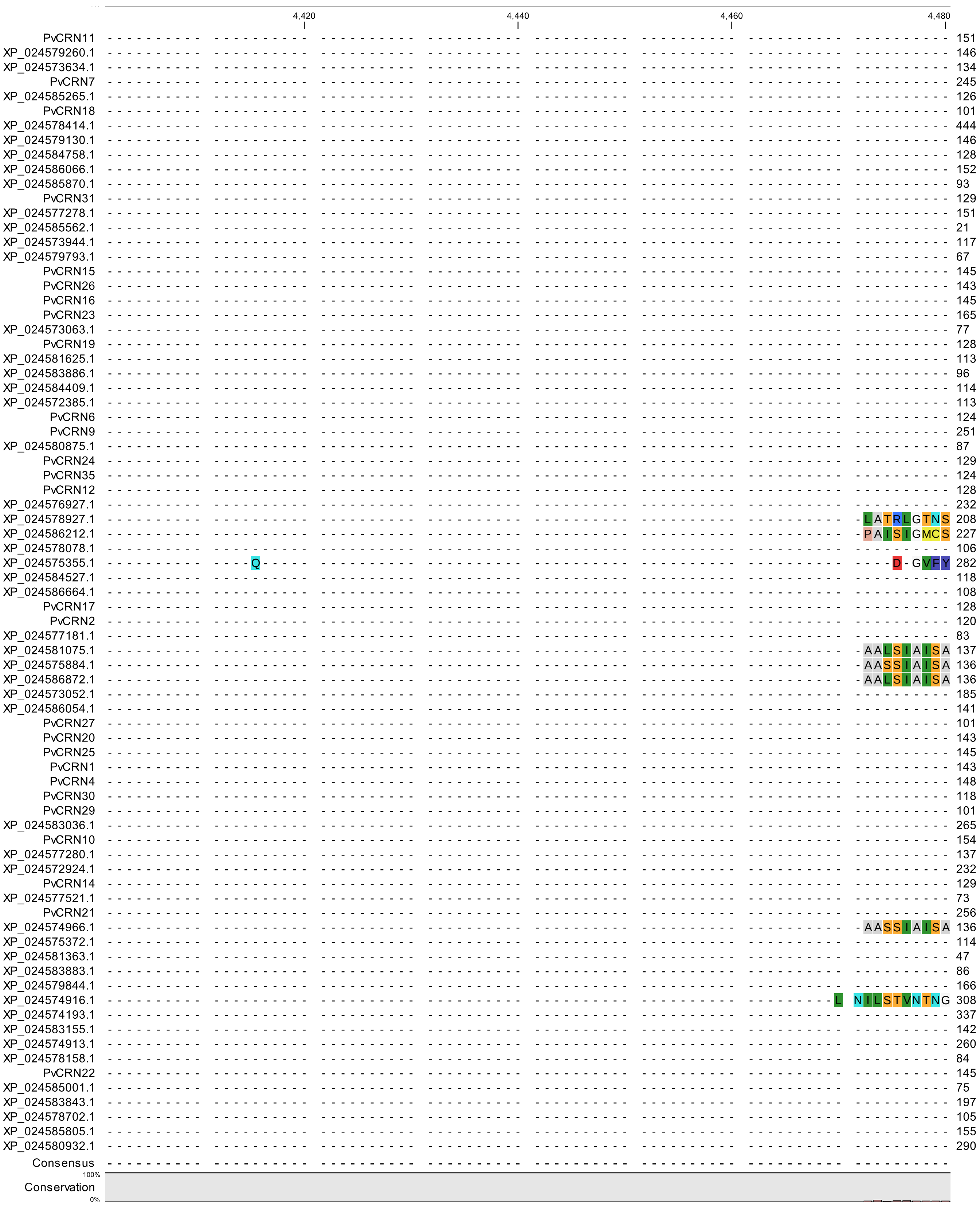
4,420
4,440
4,460
4,480
PvCRN11 151
XP_024579260.1 146
XP_024573634.1 134
PvCRN7 245
XP_024585265.1 126
PvCRN18 101
XP_024578414.1 444
XP_024579130.1 146
XP_024584758.1 128
XP_024586066.1 152
XP_024585870.1 93
PvCRN31 129
XP_024577278.1 151
XP_024585562.1 21
XP_024573944.1 117
XP_024579793.1 67
PvCRN15 145
PvCRN26 143
PvCRN16 145
PvCRN23 165
XP_024573063.1 77
PvCRN19 128
XP_024581625.1 113
XP_024583886.1 96
XP_024584409.1 114
XP_024572385.1 113
PvCRN6 124
PvCRN9 251
XP_024580875.1 87
PvCRN24 129
PvCRN35 124
PvCRN12 128
XP_024576927.1 232
XP_024578927.1 LATRLGTNS 208
XP_024586212.1 PAISIGMCS 227
XP_024578078.1 106
XP_024575355.1 Q D-GVFY 282
XP_024584527.1 118
XP_024586664.1 108
PvCRN17 128
PvCRN2 120
XP_024577181.1 83
XP_024581075.1 AALSIAISA 137
XP_024575884.1 AASSIAISA 136
XP_024586872.1 AALSIAISA 136
XP_024573052.1 185
XP_024586054.1 141
PvCRN27 101
PvCRN20 143
PvCRN25 145
PvCRN1 143
PvCRN4 148
PvCRN30 118
PvCRN29 101
XP_024583036.1 265
PvCRN10 154
XP_024577280.1 137
XP_024572924.1 232
PvCRN14 129
XP_024577521.1 73
PvCRN21 256
XP_024574966.1 AASSIAISA 136
XP_024575372.1 114
XP_024581363.1 47
XP_024583883.1 86
XP_024579844.1 166
XP_024574916.1 L NILSTVNTNG 308
XP_024574193.1 337
XP_024583155.1 142
XP_024574913.1 260
XP_024578158.1 84
PvCRN22 145
XP_024585001.1 75
XP_024583843.1 197
XP_024578702.1 105
XP_024585805.1 155
XP_024580932.1 290
Consensus
Conservation
100%
0%

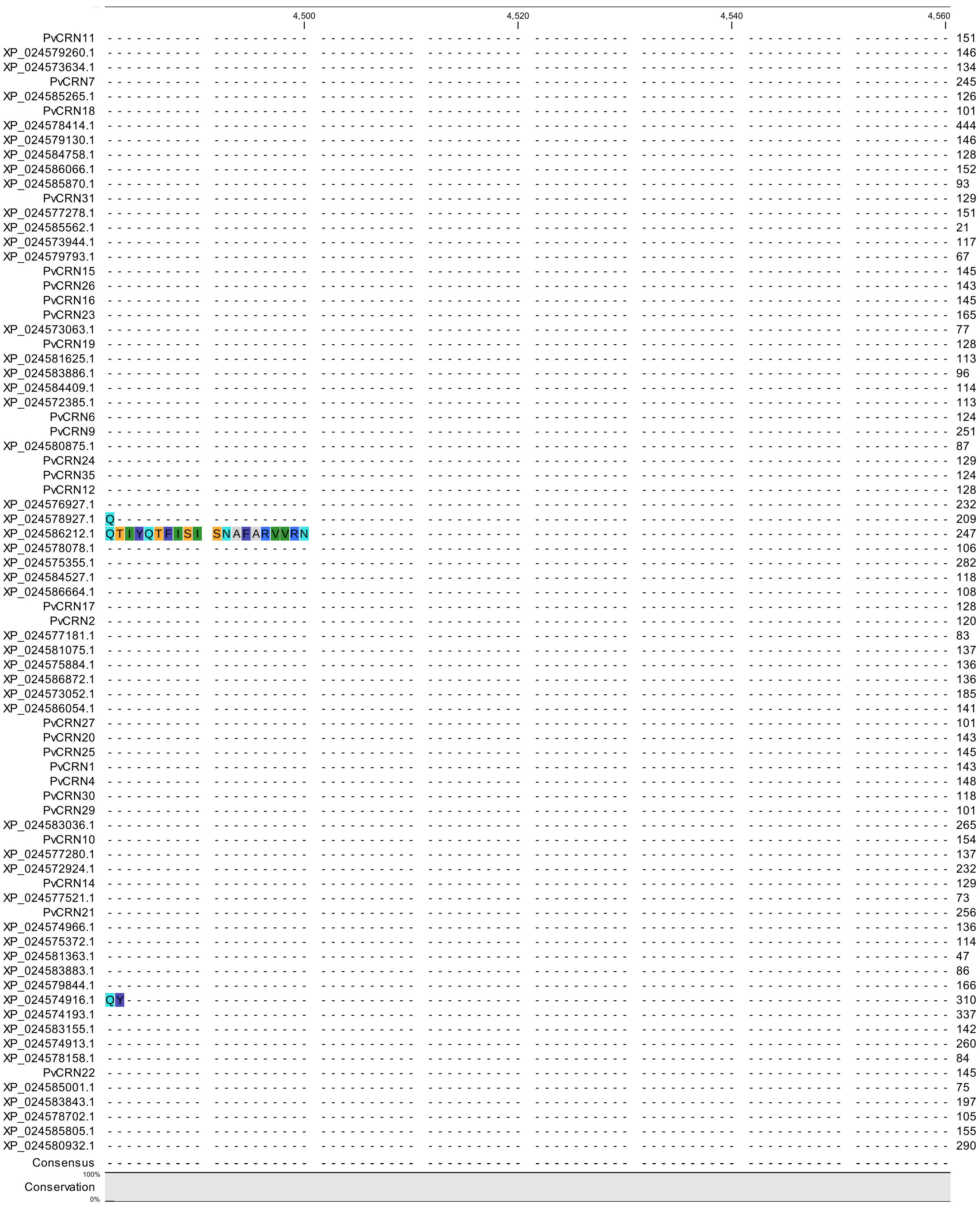
4,500
4,520
4,540
4,560
PvCRN11 151
XP_024579260.1 146
XP_024573634.1 134
PvCRN7 245
XP_024585265.1 126
PvCRN18 101
XP_024578414.1 444
XP_024579130.1 146
XP_024584758.1 128
XP_024586066.1 152
XP_024585870.1 93
PvCRN31 129
XP_024577278.1 151
XP_024585562.1 21
XP_024573944.1 117
XP_024579793.1 67
PvCRN15 145
PvCRN26 143
PvCRN16 145
PvCRN23 165
XP_024573063.1 77
PvCRN19 128
XP_024581625.1 113
XP_024583886.1 96
XP_024584409.1 114
XP_024572385.1 113
PvCRN6 124
PvCRN9 251
XP_024580875.1 87
PvCRN24 129
PvCRN35 124
PvCRN12 128
XP_024576927.1 232
XP_024578927.1 Q 209
XP_024586212.1 QTIYQTFISI SNAFARVVRN 247
XP_024578078.1 106
XP_024575355.1 282
XP_024584527.1 118
XP_024586664.1 108
PvCRN17 128
PvCRN2 120
XP_024577181.1 83
XP_024581075.1 137
XP_024575884.1 136
XP_024586872.1 136
XP_024573052.1 185
XP_024586054.1 141
PvCRN27 101
PvCRN20 143
PvCRN25 145
PvCRN1 143
PvCRN4 148
PvCRN30 118
PvCRN29 101
XP_024583036.1 265
PvCRN10 154
XP_024577280.1 137
XP_024572924.1 232
PvCRN14 129
XP_024577521.1 73
PvCRN21 256
XP_024574966.1 136
XP_024575372.1 114
XP_024581363.1 47
XP_024583883.1 86
XP_024579844.1 166
XP_024574916.1 QY 310
XP_024574193.1 337
XP_024583155.1 142
XP_024574913.1 260
XP_024578158.1 84
PvCRN22 145
XP_024585001.1 75
XP_024583843.1 197
XP_024578702.1 105
XP_024585805.1 155
XP_024580932.1 290
Consensus
100%
Conservation
0%

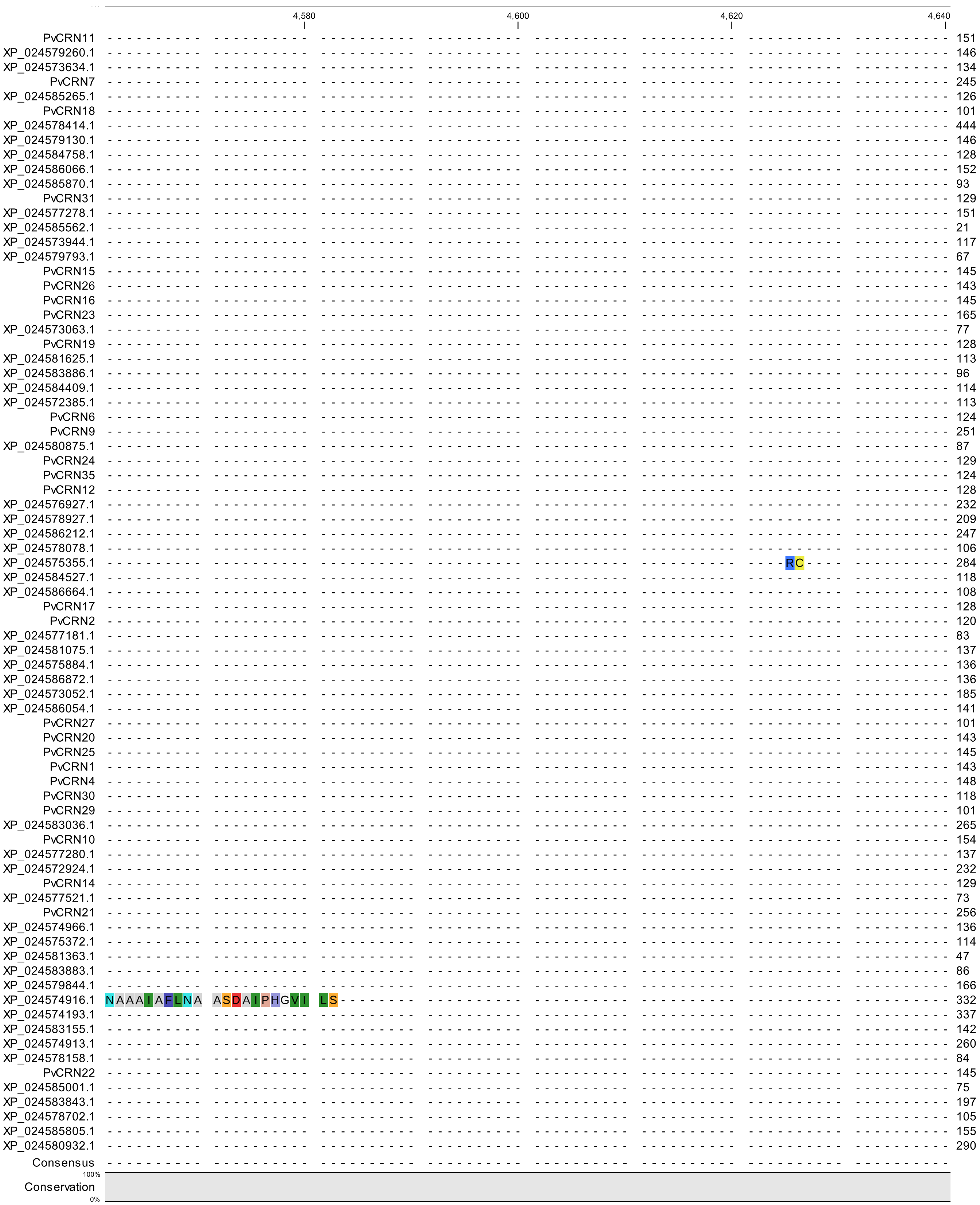
4,580
4,600
4,620
4,640
PvCRN11 151
XP_024579260.1 146
XP_024573634.1 134
PvCRN7 245
XP_024585265.1 126
PvCRN18 101
XP_024578414.1 444
XP_024579130.1 146
XP_024584758.1 128
XP_024586066.1 152
XP_024585870.1 93
PvCRN31 129
XP_024577278.1 151
XP_024585562.1 21
XP_024573944.1 117
XP_024579793.1 67
PvCRN15 145
PvCRN26 143
PvCRN16 145
PvCRN23 165
XP_024573063.1 77
PvCRN19 128
XP_024581625.1 113
XP_024583886.1 96
XP_024584409.1 114
XP_024572385.1 113
PvCRN6 124
PvCRN9 251
XP_024580875.1 87
PvCRN24 129
PvCRN35 124
PvCRN12 128
XP_024576927.1 232
XP_024578927.1 209
XP_024586212.1 247
XP_024578078.1 106
XP_024575355.1 RC 284
XP_024584527.1 118
XP_024586664.1 108
PvCRN17 128
PvCRN2 120
XP_024577181.1 83
XP_024581075.1 137
XP_024575884.1 136
XP_024586872.1 136
XP_024573052.1 185
XP_024586054.1 141
PvCRN27 101
PvCRN20 143
PvCRN25 145
PvCRN1 143
PvCRN4 148
PvCRN30 118
PvCRN29 101
XP_024583036.1 265
PvCRN10 154
XP_024577280.1 137
XP_024572924.1 232
PvCRN14 129
XP_024577521.1 73
PvCRN21 256
XP_024574966.1 136
XP_024575372.1 114
XP_024581363.1 47
XP_024583883.1 86
XP_024579844.1 166
XP_024574916.1 NAAAIAFLNA ASDAIPHGVI LS 332
XP_024574193.1 337
XP_024583155.1 142
XP_024574913.1 260
XP_024578158.1 84
PvCRN22 145
XP_024585001.1 75
XP_024583843.1 197
XP_024578702.1 105
XP_024585805.1 155
XP_024580932.1 290
Consensus
100%
Conservation
0%

| | 4,660 | 4,680 | 4,700 | 4,720 | |
|---|---|---|---|---|---|
| PvCRN11 | ---------- ---------- | ---------- ---------- | ---------- ---------- | ---------- ---------- | 151 |
| XP_024579260.1 | ---------- ---------- | ---------- ---------- | ---------- ---------- | ---------- ---------- | 146 |
| XP_024573634.1 | ---------- ---------- | ---------- ---------- | ---------- ---------- | ---------- ---------- | 134 |
| PvCRN7 | ---------- ---------- | ---------- ---------- | ---------- ---------- | ---------- ---------- | 245 |
| XP_024585265.1 | ---------- ---------- | ---------- ---------- | ---------- ---------- | ---------- ---------- | 126 |
| PvCRN18 | ---------- ---------- | ---------- ---------- | ---------- ---------- | ---------- ---------- | 101 |
| XP_024578414.1 | ---------- ---------- | ---------- ---------- | ---------- ---------- | ---------- ---------- | 444 |
| XP_024579130.1 | ---------- ---------- | ---------- ---------- | ---------- ---------- | ---------- ---------- | 146 |
| XP_024584758.1 | ---------- ---------- | ---------- ---------- | ---------- ---------- | ---------- ---------- | 128 |
| XP_024586066.1 | ---------- ---------- | ---------- ---------- | ---------- ---------- | ---------- ---------- | 152 |
| XP_024585870.1 | ---------- ---------- | ---------- ---------- | ---------- ---------- | ---------- ---------- | 93 |
| PvCRN31 | ---------- ---------- | ---------- ---------- | ---------- ---------- | ---------- ---------- | 129 |
| XP_024577278.1 | ---------- ---------- | ---------- ---------- | ---------- ---------- | ---------- ---------- | 151 |
| XP_024585562.1 | ---------- ---------- | ---------- ---------- | ---------- ---------- | ---------- ---------- | 21 |
| XP_024573944.1 | ---------- ---------- | ---------- ---------- | ---------- ---------- | ---------- ---------- | 117 |
| XP_024579793.1 | ---------- ---------- | ---------- ---------- | ---------- ---------- | ---------- ---------- | 67 |
| PvCRN15 | ---------- ---------- | ---------- ---------- | ---------- ---------- | ---------- ---------- | 145 |
| PvCRN26 | ---------- ---------- | ---------- ---------- | ---------- ---------- | ---------- ---------- | 143 |
| PvCRN16 | ---------- ---------- | ---------- ---------- | ---------- ---------- | ---------- ---------- | 145 |
| PvCRN23 | ---------- ---------- | ---------- ---------- | ---------- ---------- | ---------- ---------- | 165 |
| XP_024573063.1 | ---------- ---------- | ---------- ---------- | ---------- ---------- | ---------- ---------- | 77 |
| PvCRN19 | ---------- ---------- | ---------- ---------- | ---------- ---------- | ---------- ---------- | 128 |
| XP_024581625.1 | ---------- ---------- | ---------- ---------- | ---------- ---------- | ---------- ---------- | 113 |
| XP_024583886.1 | ---------- ---------- | ---------- ---------- | ---------- ---------- | ---------- ---------- | 96 |
| XP_024584409.1 | ---------- ---------- | ---------- ---------- | ---------- ---------- | ---------- ---------- | 114 |
| XP_024572385.1 | ---------- ---------- | ---------- ---------- | ---------- ---------- | ---------- ---------- | 113 |
| PvCRN6 | ---------- ---------- | ---------- ---------- | ---------- ---------- | ---------- ---------- | 124 |
| PvCRN9 | ---------- ---------- | ---------- ---------- | ---------- ---------- | ---------- ---------- | 251 |
| XP_024580875.1 | ---------- ---------- | ---------- ---------- | ---------- ---------- | ---------- ---------- | 87 |
| PvCRN24 | ---------- ---------- | ---------- ---------- | ---------- ---------- | ---------- ---------- | 129 |
| PvCRN35 | ---------- ---------- | ---------- ---------- | ---------- ---------- | ---------- ---------- | 124 |
| PvCRN12 | ---------- ---------- | ---------- ---------- | ---------- ---------- | ---------- ---------- | 128 |
| XP_024576927.1 | ---------- ---------- | ---------- ---------- | ---------- ---------- | ---------- ---------- | 232 |
| XP_024578927.1 | ---------- ---------- | ---------- ---------- | ---------- ---------- | ---------- ---------- | 209 |
| XP_024586212.1 | ---------- ---------- | ---------- ---------- | ---------- ---------- | ---------- ---------- | 247 |
| XP_024578078.1 | ---------- ---------- | ---------- ---------- | ---------- ---------- | ---------- ---------- | 106 |
| XP_024575355.1 | ---------- ---------- | ---------- ---------- | ---------- ---------- | ---------- ---------- | 284 |
| XP_024584527.1 | ---------- ---------- | ---------- ---------- | ---------- ---------- | ---------- ---------- | 118 |
| XP_024586664.1 | ---------- ---------- | ---------- ---------- | ---------- ---------- | ---------- ---------- | 108 |
| PvCRN17 | ---------- ---------- | ---------- ---------- | ---------- ---------- | ---------- ---------- | 128 |
| PvCRN2 | ---------- ---------- | ---------- ---------- | ---------- ---------- | ---------- ---------- | 120 |
| XP_024577181.1 | ---------- ---------- | ---------- ---------- | ---------- ---------- | ---------- ---------- | 83 |
| XP_024581075.1 | ---------- ---------- | ---------- ---------- | ---------- ---------- | ---------- ---------- | 137 |
| XP_024575884.1 | ---------- ---------- | ---------- ---------- | ---------- ---------- | ---------- ---------- | 136 |
| XP_024586872.1 | ---------- ---------- | ---------- ---------- | ---------- ---------- | ---------- ---------- | 136 |
| XP_024573052.1 | ---------- ---------- | ---------- ---------- | ---------- ---------- | ---------- ---------- | 185 |
| XP_024586054.1 | ---------- ---------- | ---------- ---------- | ---------- ---------- | ---------- ---------- | 141 |
| PvCRN27 | ---------- ---------- | ---------- ---------- | ---------- ---------- | ---------- ---------- | 101 |
| PvCRN20 | ---------- ---------- | ---------- ---------- | ---------- ---------- | ---------- ---------- | 143 |
| PvCRN25 | ---------- ---------- | ---------- ---------- | ---------- ---------- | ---------- ---------- | 145 |
| PvCRN1 | ---------- ---------- | ---------- ---------- | ---------- ---------- | ---------- ---------- | 143 |
| PvCRN4 | ---------- ---------- | ---------- ---------- | ---------- ---------- | ---------- ---------- | 148 |
| PvCRN30 | ---------- ---------- | ---------- ---------- | ---------- ---------- | ---------- ---------- | 118 |
| PvCRN29 | ---------- ---------- | ---------- ---------- | ---------- ---------- | ---------- ---------- | 101 |
| XP_024583036.1 | ---------- ---------- | ---------- ---------- | ---------- ---------- | ---------- ---------- | 265 |
| PvCRN10 | ---------- ---------- | ---------- ---------- | ---------- ---------- | ---------- ---------- | 154 |
| XP_024577280.1 | ---------- ---------- | ---------- ---------- | ---------- ---------- | ---------- ---------- | 137 |
| XP_024572924.1 | ---------- ---------- | ---------- ---------- | ---------- ---------- | ---------- ---------- | 232 |
| PvCRN14 | ---------- ---------- | ---------- ---------- | ---------- ---------- | ---------- ---------- | 129 |
| XP_024577521.1 | ---------- ---------- | ---------- ---------- | ---------- ---------- | ---------- ---------- | 73 |
| PvCRN21 | ---------- ---------- | ---------- ---------- | ---------- ---------- | ---------- ---------- | 256 |
| XP_024574966.1 | ---------- ---------- | ---------- ---------- | ---------- ---------- | ---------- ---------- | 136 |
| XP_024575372.1 | ---------- ---------- | ---------- ---------- | ---------- ---------- | ---------- ---------- | 114 |
| XP_024581363.1 | ---------- ---------- | ---------- ---------- | ---------- ---------- | ---------- ---------- | 47 |
| XP_024583883.1 | ---------- ---------- | ---------- ---------- | ---------- ---------- | ---------- ---------- | 86 |
| XP_024579844.1 | ---------- ---------- | ---------- ---------- | ---------- ---------- | ---------- ---------- | 166 |
| XP_024574916.1 | ---------- ---------- | ---------- ---------- | ---------- ---------- | ---------- ---------- | 332 |
| XP_024574193.1 | ---------- ---------- | ---------- ---------- | ---------- ---------- | ---------- ---------- | 337 |
| XP_024583155.1 | ---------- ---------- | ---------- ---------- | ---------- ---------- | ---------- ---------- | 142 |
| XP_024574913.1 | ---------- ---------- | ---------- ---------- | ---------- ---------- | ---------- ---------- | 260 |
| XP_024578158.1 | ---------- ---------- | ---------- ---------- | ---------- ---------- | ---------- ---------- | 84 |
| PvCRN22 | ---------- ---------- | ---------- ---------- | ---------- ---------- | ---------- ---------- | 145 |
| XP_024585001.1 | ---------- ---------- | ---------- ---------- | ---------- ---------- | ---------- ---------- | 75 |
| XP_024583843.1 | ---------- ---------- | ---------- ---------- | ---------- ---------- | ---------- ---------- | 197 |
| XP_024578702.1 | ---------- ---------- | ---------- ---------- | ---------- ---------- | ---------- ---------- | 105 |
| XP_024585805.1 | ---------- ---------- | ---------- ---------- | ---------- ---------- | ---------- ---------- | 155 |
| XP_024580932.1 | ---------- ---------- | ---------- ---------- | ---------- ---------- | ---------- ---------- | 290 |
| Consensus | ---------- ---------- | ---------- ---------- | ---------- ---------- | ---------- ---------- | |
| Conservation (100% / 0%) | | | | | |

| | 4,740 · 4,760 · 4,780 · 4,800 | |
|---|---|---|
| PvCRN11 | ---------- ---------- ---------- ---------- ---------- ---------- ---------- ---------- | 151 |
| XP_024579260.1 | ---------- ---------- ---------- ---------- ---------- ---------- ---------- ---------- | 146 |
| XP_024573634.1 | ---------- ---------- ---------- ---------- ---------- ---------- ---------- ---------- | 134 |
| PvCRN7 | ---------- ---------- ---------- ---------- ---------- ---------- ---------- ---------- | 245 |
| XP_024585265.1 | ---------- ---------- ---------- ---------- ---------- ---------- ---------- ---------- | 126 |
| PvCRN18 | ---------- ---------- ---------- ---------- ---------- ---------- ---------- ---------- | 101 |
| XP_024578414.1 | ---------- ---------- ---------- ---------- ---------- ---------- ---------- ---------- | 444 |
| XP_024579130.1 | ---------- ---------- ---------- ---------- ---------- ---------- ---------- ---------- | 146 |
| XP_024584758.1 | ---------- ---------- ---------- ---------- ---------- ---------- ---------- ---------- | 128 |
| XP_024586066.1 | ---------- ---------- ---------- ---------- ---------- ---------- ---------- ---------- | 152 |
| XP_024585870.1 | ---------- ---------- ---------- ---------- ---------- ---------- ---------- ---------- | 93 |
| PvCRN31 | ---------- ---------- ---------- ---------- ---------- ---------- ---------- ---------- | 129 |
| XP_024577278.1 | ---------- ---------- ---------- ---------- ---------- ---------- ---------- ---------- | 151 |
| XP_024585562.1 | ---------- ---------- ---------- ---------- ---------- ---------- ---------- ---------- | 21 |
| XP_024573944.1 | ---------- ---------- ---------- ---------- ---------- ---------- ---------- ---------- | 117 |
| XP_024579793.1 | ---------- ---------- ---------- ---------- ---------- ---------- ---------- ---------- | 67 |
| PvCRN15 | ---------- ---------- ---------- ---------- ---------- ---------- ---------- ---------- | 145 |
| PvCRN26 | ---------- ---------- ---------- ---------- ---------- ---------- ---------- ---------- | 143 |
| PvCRN16 | ---------- ---------- ---------- ---------- ---------- ---------- ---------- ---------- | 145 |
| PvCRN23 | ---------- ---------- ---------- ---------- ---------- ---------- ---------- ---------- | 165 |
| XP_024573063.1 | ---------- ---------- ---------- ---------- ---------- ---------- ---------- ---------- | 77 |
| PvCRN19 | ---------- ---------- ---------- ---------- ---------- ---------- ---------- ---------- | 128 |
| XP_024581625.1 | ---------- ---------- ---------- ---------- ---------- ---------- ---------- ---------- | 113 |
| XP_024583886.1 | ---------- ---------- ---------- ---------- ---------- ---------- ---------- ---------- | 96 |
| XP_024584409.1 | ---------- ---------- ---------- ---------- ---------- ---------- ---------- ---------- | 114 |
| XP_024572385.1 | ---------- ---------- ---------- ---------- ---------- ---------- ---------- ---------- | 113 |
| PvCRN6 | ---------- ---------- ---------- ---------- ---------- ---------- ---------- ---------- | 124 |
| PvCRN9 | ---------- ---------- ---------- ---------- ---------- ---------- ---------- ---------- | 251 |
| XP_024580875.1 | ---------- ---------- ---------- ---------- ---------- ---------- ---------- ---------- | 87 |
| PvCRN24 | ---------- ---------- ---------- ---------- ---------- ---------- ---------- ---------- | 129 |
| PvCRN35 | ---------- ---------- ---------- ---------- ---------- ---------- ---------- ---------- | 124 |
| PvCRN12 | ---------- ---------- ---------- ---------- ---------- ---------- ---------- ---------- | 128 |
| XP_024576927.1 | ---------- ---------- ---------- ---------- ---------- ---------- ---------- ---------- | 232 |
| XP_024578927.1 | ---------- ---------- ---------- ---------- ---------- ---------- ---------- ---------- | 209 |
| XP_024586212.1 | ---------- ---------- ---------- ---------- ---------- ---------- ---------- ---------- | 247 |
| XP_024578078.1 | ---------- ---------- ---------- ---------- ---------- ---------- ---------- ---------- | 106 |
| XP_024575355.1 | ---------- ---------- ---------- ---------- ---------- ---------- ---------- ---------- | 284 |
| XP_024584527.1 | ---------- ---------- ---------- ---------- ---------- ---------- ---------- ---------- | 118 |
| XP_024586664.1 | ---------- ---------- ---------- ---------- ---------- ---------- ---------- ---------- | 108 |
| PvCRN17 | ---------- ---------- ---------- ---------- ---------- ---------- ---------- ---------- | 128 |
| PvCRN2 | ---------- ---------- ---------- ---------- ---------- ---------- ---------- ---------- | 120 |
| XP_024577181.1 | ---------- ---------- ---------- ---------- ---------- ---------- ---------- ---------- | 83 |
| XP_024581075.1 | ---------- ---------- ---------- ---------- ---------- ---------- ---------- ---------- | 137 |
| XP_024575884.1 | ---------- ---------- ---------- ---------- ---------- ---------- ---------- ---------- | 136 |
| XP_024586872.1 | ---------- ---------- ---------- ---------- ---------- ---------- ---------- ---------- | 136 |
| XP_024573052.1 | ---------- ---------- ---------- ---------- ---------- ---------- ---------- ---------- | 185 |
| XP_024586054.1 | ---------- ---------- ---------- ---------- ---------- ---------- ---------- ---------- | 141 |
| PvCRN27 | ---------- ---------- ---------- ---------- ---------- ---------- ---------- ---------- | 101 |
| PvCRN20 | ---------- ---------- ---------- ---------- ---------- ---------- ---------- ---------- | 143 |
| PvCRN25 | ---------- ---------- ---------- ---------- ---------- ---------- ---------- ---------- | 145 |
| PvCRN1 | ---------- ---------- ---------- ---------- ---------- ---------- ---------- ---------- | 143 |
| PvCRN4 | ---------- ---------- ---------- ---------- ---------- ---------- ---------- ---------- | 148 |
| PvCRN30 | ---------- ---------- ---------- ---------- ---------- ---------- ---------- ---------- | 118 |
| PvCRN29 | ---------- ---------- ---------- ---------- ---------- ---------- ---------- ---------- | 101 |
| XP_024583036.1 | ---------- ---------- ---------- ---------- ---------- ---------- ---------- ---------- | 265 |
| PvCRN10 | ---------- ---------- ---------- ---------- ---------- ---------- ---------- ---------- | 154 |
| XP_024577280.1 | ---------- ---------- ---------- ---------- ---------- ---------- ---------- ---------- | 137 |
| XP_024572924.1 | ---------- ---------- ---------- ---------- ---------- ---------- ---------- ---------- | 232 |
| PvCRN14 | ---------- ---------- ---------- ---------- ---------- ---------- ---------- ---------- | 129 |
| XP_024577521.1 | ---------- ---------- ---------- ---------- ---------- ---------- ---------- ---------- | 73 |
| PvCRN21 | ---------- ---------- ---------- ---------- ---------- ---------- ---------- ---------- | 256 |
| XP_024574966.1 | ---------- ---------- ---------- ---------- ---------- ---------- ---------- ---------- | 136 |
| XP_024575372.1 | ---------- ---------- ---------- ---------- ---------- ---------- ---------- ---------- | 114 |
| XP_024581363.1 | ---------- ---------- ---------- ---------- ---------- ---------- ---------- ---------- | 47 |
| XP_024583883.1 | ---------- ---------- ---------- ---------- ---------- ---------- ---------- ---------- | 86 |
| XP_024579844.1 | ---------- ---------- ---------- ---------- ---------- ---------- ---------- ---------- | 166 |
| XP_024574916.1 | ---------- ---------- ---------- ---------- ---------- ---------- ---------- ---------- | 332 |
| XP_024574193.1 | ---------- ---------- ---------- ---------- ---------- ---------- ---------- ---------- | 337 |
| XP_024583155.1 | ---------- ---------- ---------- ---------- ---------- ---------- ---------- ---------- | 142 |
| XP_024574913.1 | ---------- ---------- ---------- ---------- ---------- ---------- ---------- ---------- | 260 |
| XP_024578158.1 | ---------- ---------- ---------- ---------- ---------- ---------- ---------- ---------- | 84 |
| PvCRN22 | ---------- ---------- ---------- ---------- ---------- ---------- ---------- ---------- | 145 |
| XP_024585001.1 | ---------- ---------- ---------- ---------- ---------- ---------- ---------- ---------- | 75 |
| XP_024583843.1 | ---------- ---------- ---------- ---------- ---------- ---------- ---------- ---------- | 197 |
| XP_024578702.1 | ---------- ---------- ---------- ---------- ---------- ---------- ---------- ---------- | 105 |
| XP_024585805.1 | ---------- ---------- ---------- ---------- ---------- ---------- ---------- ---------- | 155 |
| XP_024580932.1 | ---------- ---------- ---------- ---------- ---------- ---------- ---------- ---------- | 290 |
| Consensus | ---------- ---------- ---------- ---------- ---------- ---------- ---------- ---------- | |
| Conservation (100% / 0%) | | |

| Sequence | 4,801–4,820 | 4,821–4,840 | 4,841–4,860 | 4,861–4,880 | End |
|---|---|---|---|---|---|
| PvCRN11 | ---------- ---------- | ---------- ---------- | ---------- ---------- | ---------- ---------- | 151 |
| XP_024579260.1 | ---------- ---------- | ---------- ---------- | ---------- ---------- | ---------- ---------- | 146 |
| XP_024573634.1 | ---------- ---------- | ---------- ---------- | ---------- ---------- | ---------- ---------- | 134 |
| PvCRN7 | ---------- ---------- | ---------- ---------- | ---------- ---------- | ---------- ---------- | 245 |
| XP_024585265.1 | ---------- ---------- | ---------- ---------- | ---------- ---------- | ---------- ---------- | 126 |
| PvCRN18 | ---------- ---------- | ---------- ---------- | ---------- ---------- | ---------- ---------- | 101 |
| XP_024578414.1 | ---------- ---------- | ---------- ---------- | ---------- ---------- | ---------- ---------- | 444 |
| XP_024579130.1 | ---------- ---------- | ---------- ---------- | ---------- ---------- | ---------- ---------- | 146 |
| XP_024584758.1 | ---------- ---------- | ---------- ---------- | ---------- ---------- | ---------- ---------- | 128 |
| XP_024586066.1 | ---------- ---------- | ---------- ---------- | ---------- ---------- | ---------- ---------- | 152 |
| XP_024585870.1 | ---------- ---------- | ---------- ---------- | ---------- ---------- | ---------- ---------- | 93 |
| PvCRN31 | ---------- ---------- | ---------- ---------- | ---------- ---------- | ---------- ---------- | 129 |
| XP_024577278.1 | ---------- ---------- | ---------- ---------- | ---------- ---------- | ---------- ---------- | 151 |
| XP_024585562.1 | ---------- ---------- | ---------- ---------- | ---------- ---------- | ---------- ---------- | 21 |
| XP_024573944.1 | ---------- ---------- | ---------- ---------- | ---------- ---------- | ---------- ---------- | 117 |
| XP_024579793.1 | ---------- ---------- | ---------- ---------- | ---------- ---------- | ---------- ---------- | 67 |
| PvCRN15 | ---------- ---------- | ---------- ---------- | ---------- ---------- | ---------- ---------- | 145 |
| PvCRN26 | ---------- ---------- | ---------- ---------- | ---------- ---------- | ---------- ---------- | 143 |
| PvCRN16 | ---------- ---------- | ---------- ---------- | ---------- ---------- | ---------- ---------- | 145 |
| PvCRN23 | ---------- ---------- | ---------- ---------- | ---------- ---------- | ---------- ---------- | 165 |
| XP_024573063.1 | ---------- ---------- | ---------- ---------- | ---------- ---------- | ---------- ---------- | 77 |
| PvCRN19 | ---------- ---------- | ---------- ---------- | ---------- ---------- | ---------- ---------- | 128 |
| XP_024581625.1 | ---------- ---------- | ---------- ---------- | ---------- ---------- | ---------- ---------- | 113 |
| XP_024583886.1 | ---------- ---------- | ---------- ---------- | ---------- ---------- | ---------- ---------- | 96 |
| XP_024584409.1 | ---------- ---------- | ---------- ---------- | ---------- ---------- | ---------- ---------- | 114 |
| XP_024572385.1 | ---------- ---------- | ---------- ---------- | ---------- ---------- | ---------- ---------- | 113 |
| PvCRN6 | ---------- ---------- | ---------- ---------- | ---------- ---------- | ---------- ---------- | 124 |
| PvCRN9 | ---------- ---------- | ---------- ---------- | ---------- ---------- | ---------- ---------- | 251 |
| XP_024580875.1 | ---------- ---------- | ---------- ---------- | ---------- ---------- | ---------- ---------- | 87 |
| PvCRN24 | ---------- ---------- | ---------- ---------- | ---------- ---------- | ---------- ---------- | 129 |
| PvCRN35 | ---------- ---------- | ---------- ---------- | ---------- ---------- | ---------- ---------- | 124 |
| PvCRN12 | ---------- ---------- | ---------- ---------- | ---------- ---------- | ---------- ---------- | 128 |
| XP_024576927.1 | ---------- ---------- | ---------- ---------- | ---------- ---------- | ---------- ---------- | 232 |
| XP_024578927.1 | ---------- ---------- | ---------- ---------- | ---------- ---------- | ---------- ---------- | 209 |
| XP_024586212.1 | ---------- ---------- | ---------- ---------- | ---------- ---------- | ---------- ---------- | 247 |
| XP_024578078.1 | ---------- ---------- | ---------- ---------- | ---------- ---------- | ---------- ---------- | 106 |
| XP_024575355.1 | ---------- ---------- | ---------- ---------- | ---------- ---------- | ---------- ---------- | 284 |
| XP_024584527.1 | ---------- ---------- | ---------- ---------- | ---------- ---------- | ---------- ---------- | 118 |
| XP_024586664.1 | ---------- ---------- | ---------- ---------- | ---------- ---------- | ---------- ---------- | 108 |
| PvCRN17 | ---------- ---------- | ---------- ---------- | ---------- ---------- | ---------- ---------- | 128 |
| PvCRN2 | ---------- ---------- | ---------- ---------- | ---------- ---------- | ---------- ---------- | 120 |
| XP_024577181.1 | ---------- ---------- | ---------- ---------- | ---------- ---------- | ---------- ---------- | 83 |
| XP_024581075.1 | ---------- ---------- | ---------- ---------- | ---------- ---------- | ---------- ---------- | 137 |
| XP_024575884.1 | ---------- ---------- | ---------- ---------- | ---------- ---------- | ---------- ---------- | 136 |
| XP_024586872.1 | ---------- ---------- | ---------- ---------- | ---------- ---------- | ---------- ---------- | 136 |
| XP_024573052.1 | ---------- ---------- | ---------- ---------- | ---------- ---------- | ---------- ---------- | 185 |
| XP_024586054.1 | ---------- ---------- | ---------- ---------- | ---------- ---------- | ---------- ---------- | 141 |
| PvCRN27 | ---------- ---------- | ---------- ---------- | ---------- ---------- | ---------- ---------- | 101 |
| PvCRN20 | ---------- ---------- | ---------- ---------- | ---------- ---------- | ---------- ---------- | 143 |
| PvCRN25 | ---------- ---------- | ---------- ---------- | ---------- ---------- | ---------- ---------- | 145 |
| PvCRN1 | ---------- ---------- | ---------- ---------- | ---------- ---------- | ---------- ---------- | 143 |
| PvCRN4 | ---------- ---------- | ---------- ---------- | ---------- ---------- | ---------- ---------- | 148 |
| PvCRN30 | ---------- ---------- | ---------- ---------- | ---------- ---------- | ---------- ---------- | 118 |
| PvCRN29 | ---------- ---------- | ---------- ---------- | ---------- ---------- | ---------- ---------- | 101 |
| XP_024583036.1 | ---------- ---------- | ---------- ---------- | ---------- ---------- | ---------- ---------- | 265 |
| PvCRN10 | ---------- ---------- | ---------- ---------- | ---------- ---------- | ---------- ---------- | 154 |
| XP_024577280.1 | ---------- ---------- | ---------- ---------- | ---------- ---------- | ---------- ---------- | 137 |
| XP_024572924.1 | ---------- ---------- | ---------- ---------- | ---------- ---------- | ---------- ---------- | 232 |
| PvCRN14 | ---------- ---------- | ---------- ---------- | ---------- ---------- | ---------- ---------- | 129 |
| XP_024577521.1 | ---------- ---------- | ---------- ---------- | ---------- ---------- | ---------- ---------- | 73 |
| PvCRN21 | ---------- ---------- | ---------- ---------- | ---------- ---------- | ---------- ---------- | 256 |
| XP_024574966.1 | ---------- ---------- | ---------- ---------- | ---------- ---------- | ---------- ---------- | 136 |
| XP_024575372.1 | ---------- ---------- | ---------- ---------- | ---------- ---------- | ---------- ---------- | 114 |
| XP_024581363.1 | ---------- ---------- | ---------- ---------- | ---------- ---------- | ---------- ---------- | 47 |
| XP_024583883.1 | ---------- ---------- | ---------- ---------- | ---------- ---------- | ---------- ---------- | 86 |
| XP_024579844.1 | ---------- ---------- | ---------- ---------- | ---------- ---------- | ---------- ---------- | 166 |
| XP_024574916.1 | ---------- ---------- | ---------- ---------- | -----QRTAA AAGD---LVF | LTRSATNNLS ---------- | 354 |
| XP_024574193.1 | ---------- ---------- | ---------- ---------- | ---------- ---------- | ---------- ---------- | 337 |
| XP_024583155.1 | ---------- ---------- | ---------- ---------- | ---------- ---------- | ---------- ---------- | 142 |
| XP_024574913.1 | ---------- ---------- | ---------- ---------- | ---------- ---------- | ---------- ---------- | 260 |
| XP_024578158.1 | ---------- ---------- | ---------- ---------- | ---------- ---------- | ---------- ---------- | 84 |
| PvCRN22 | ---------- ---------- | ---------- ---------- | ---------- ---------- | ---------- ---------- | 145 |
| XP_024585001.1 | ---------- ---------- | ---------- ---------- | ---------- ---------- | ---------- ---------- | 75 |
| XP_024583843.1 | ---------- ---------- | ---------- ---------- | ---------- ---------- | ---------- ---------- | 197 |
| XP_024578702.1 | ---------- ---------- | ---------- ---------- | ---------- ---------- | ---------- ---------- | 105 |
| XP_024585805.1 | ---------- ---------- | ---------- ---------- | ---------- ---------- | ---------- ---------- | 155 |
| XP_024580932.1 | ---------- ---------- | ---------- ---------- | ---------- ---------- | ---------- ---------- | 290 |
| Consensus | ---------- ---------- | ---------- ---------- | ---------- ---------- | ---------- ---------- | |

Conservation (100% – 0%)

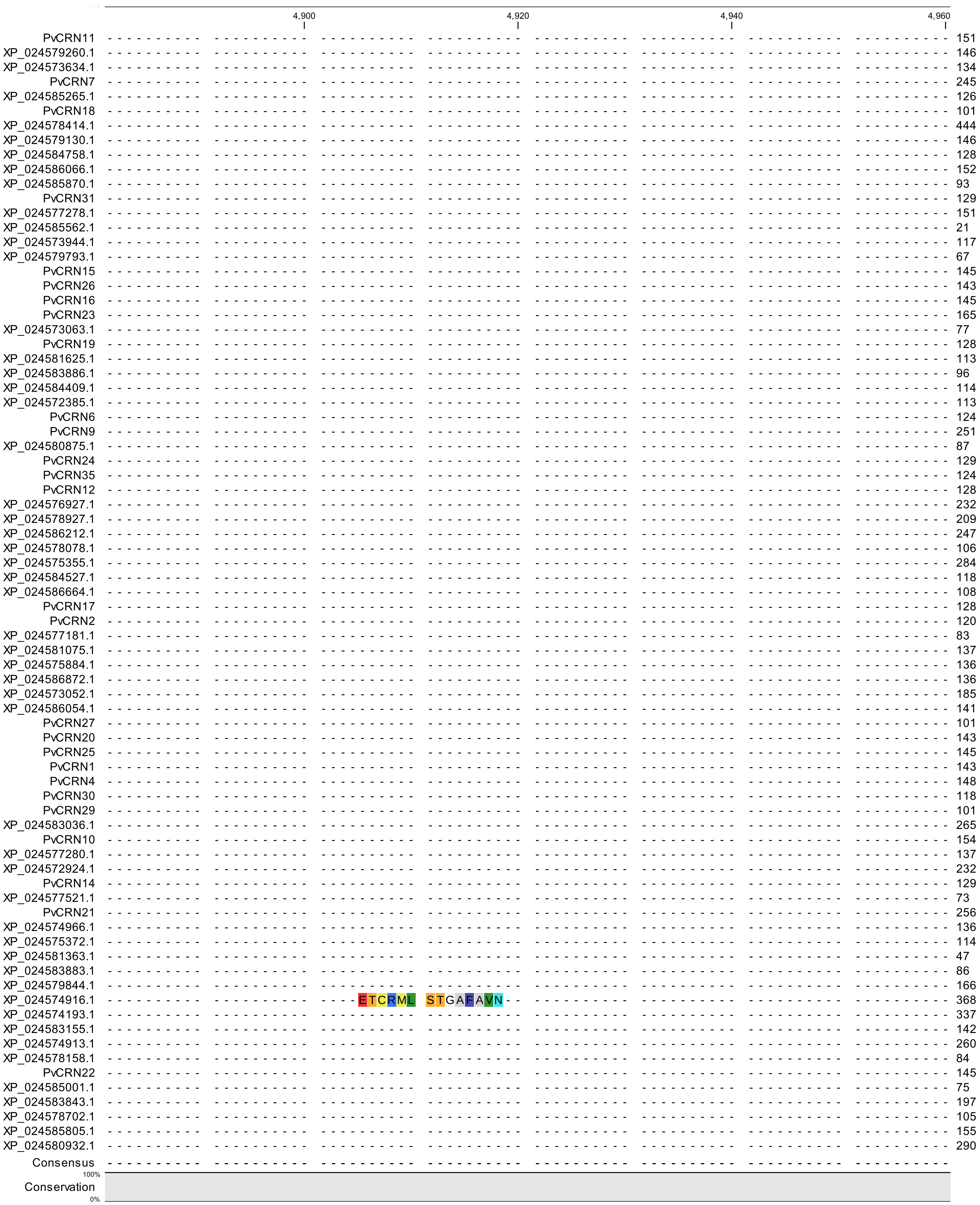
4,900
4,920
4,940
4,960
PvCRN11 151
XP_024579260.1 146
XP_024573634.1 134
PvCRN7 245
XP_024585265.1 126
PvCRN18 101
XP_024578414.1 444
XP_024579130.1 146
XP_024584758.1 128
XP_024586066.1 152
XP_024585870.1 93
PvCRN31 129
XP_024577278.1 151
XP_024585562.1 21
XP_024573944.1 117
XP_024579793.1 67
PvCRN15 145
PvCRN26 143
PvCRN16 145
PvCRN23 165
XP_024573063.1 77
PvCRN19 128
XP_024581625.1 113
XP_024583886.1 96
XP_024584409.1 114
XP_024572385.1 113
PvCRN6 124
PvCRN9 251
XP_024580875.1 87
PvCRN24 129
PvCRN35 124
PvCRN12 128
XP_024576927.1 232
XP_024578927.1 209
XP_024586212.1 247
XP_024578078.1 106
XP_024575355.1 284
XP_024584527.1 118
XP_024586664.1 108
PvCRN17 128
PvCRN2 120
XP_024577181.1 83
XP_024581075.1 137
XP_024575884.1 136
XP_024586872.1 136
XP_024573052.1 185
XP_024586054.1 141
PvCRN27 101
PvCRN20 143
PvCRN25 145
PvCRN1 143
PvCRN4 148
PvCRN30 118
PvCRN29 101
XP_024583036.1 265
PvCRN10 154
XP_024577280.1 137
XP_024572924.1 232
PvCRN14 129
XP_024577521.1 73
PvCRN21 256
XP_024574966.1 136
XP_024575372.1 114
XP_024581363.1 47
XP_024583883.1 86
XP_024579844.1 166
XP_024574916.1 ETCRML STGAFAVN 368
XP_024574193.1 337
XP_024583155.1 142
XP_024574913.1 260
XP_024578158.1 84
PvCRN22 145
XP_024585001.1 75
XP_024583843.1 197
XP_024578702.1 105
XP_024585805.1 155
XP_024580932.1 290
Consensus
100%
Conservation
0%

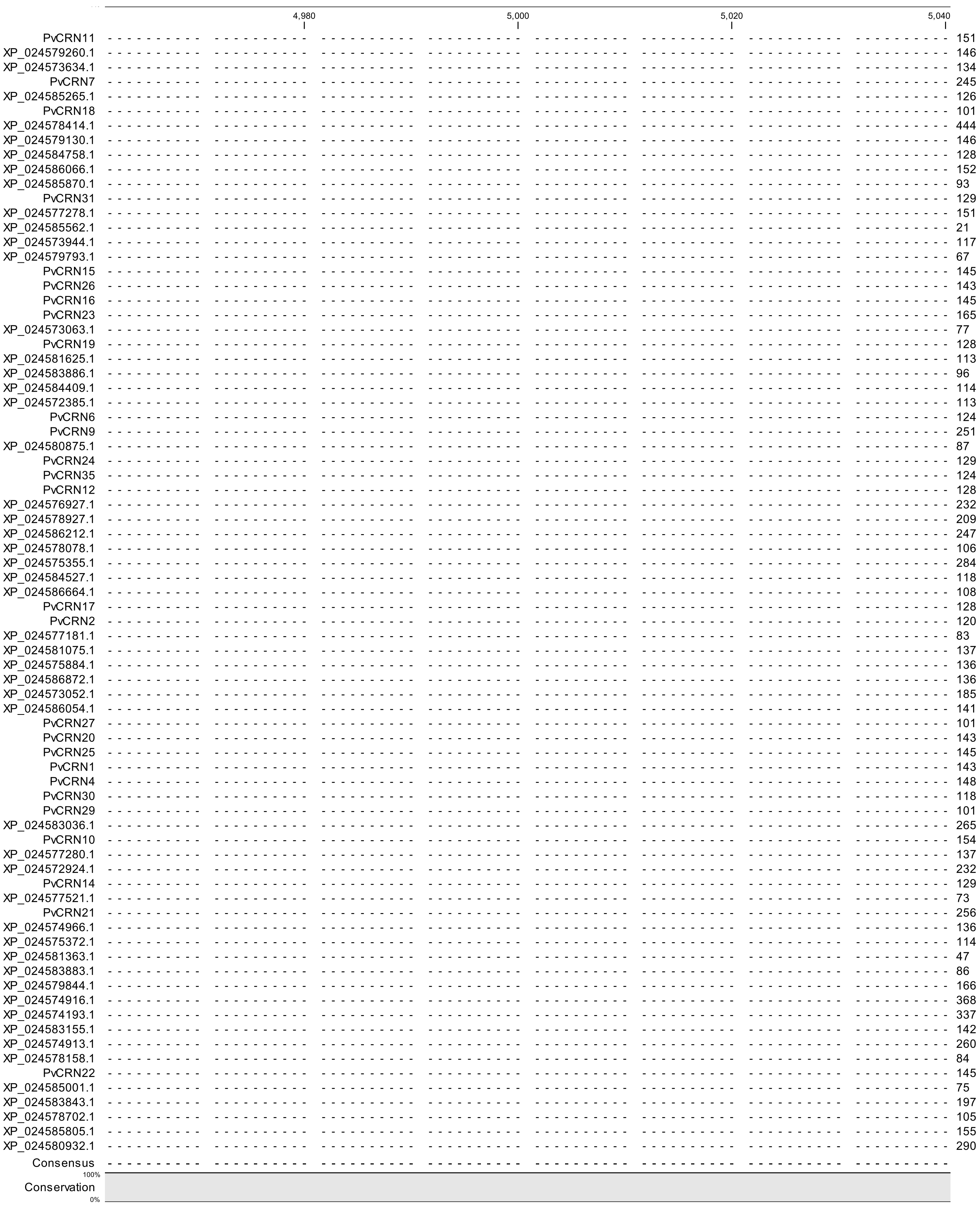
4,980
5,000
5,020
5,040
PvCRN11 151
XP_024579260.1 146
XP_024573634.1 134
PvCRN7 245
XP_024585265.1 126
PvCRN18 101
XP_024578414.1 444
XP_024579130.1 146
XP_024584758.1 128
XP_024586066.1 152
XP_024585870.1 93
PvCRN31 129
XP_024577278.1 151
XP_024585562.1 21
XP_024573944.1 117
XP_024579793.1 67
PvCRN15 145
PvCRN26 143
PvCRN16 145
PvCRN23 165
XP_024573063.1 77
PvCRN19 128
XP_024581625.1 113
XP_024583886.1 96
XP_024584409.1 114
XP_024572385.1 113
PvCRN6 124
PvCRN9 251
XP_024580875.1 87
PvCRN24 129
PvCRN35 124
PvCRN12 128
XP_024576927.1 232
XP_024578927.1 209
XP_024586212.1 247
XP_024578078.1 106
XP_024575355.1 284
XP_024584527.1 118
XP_024586664.1 108
PvCRN17 128
PvCRN2 120
XP_024577181.1 83
XP_024581075.1 137
XP_024575884.1 136
XP_024586872.1 136
XP_024573052.1 185
XP_024586054.1 141
PvCRN27 101
PvCRN20 143
PvCRN25 145
PvCRN1 143
PvCRN4 148
PvCRN30 118
PvCRN29 101
XP_024583036.1 265
PvCRN10 154
XP_024577280.1 137
XP_024572924.1 232
PvCRN14 129
XP_024577521.1 73
PvCRN21 256
XP_024574966.1 136
XP_024575372.1 114
XP_024581363.1 47
XP_024583883.1 86
XP_024579844.1 166
XP_024574916.1 368
XP_024574193.1 337
XP_024583155.1 142
XP_024574913.1 260
XP_024578158.1 84
PvCRN22 145
XP_024585001.1 75
XP_024583843.1 197
XP_024578702.1 105
XP_024585805.1 155
XP_024580932.1 290
Consensus
100%
Conservation
0%

| | 5,060 5,080 5,100 5,120 | |
|---|---|---|
| PvCRN11 | ---------- ---------- ---------- ---------- ---------- ---------- ---------- ---------- | 151 |
| XP_024579260.1 | ---------- ---------- ---------- ---------- ---------- ---------- ---------- ---------- | 146 |
| XP_024573634.1 | ---------- ---------- ---------- ---------- ---------- ---------- ---------- ---------- | 134 |
| PvCRN7 | ---------- ---------- ---------- ---------- ---------- ---------- ---------- ---------- | 245 |
| XP_024585265.1 | ---------- ---------- ---------- ---------- ---------- ---------- ---------- ---------- | 126 |
| PvCRN18 | ---------- ---------- ---------- ---------- ---------- ---------- ---------- ---------- | 101 |
| XP_024578414.1 | ---------- ---------- ---------- ---------- ---------- ---------- ---------- ---------- | 444 |
| XP_024579130.1 | ---------- ---------- ---------- ---------- ---------- ---------- ---------- ---------- | 146 |
| XP_024584758.1 | ---------- ---------- ---------- ---------- ---------- ---------- ---------- ---------- | 128 |
| XP_024586066.1 | ---------- ---------- ---------- ---------- ---------- ---------- ---------- ---------- | 152 |
| XP_024585870.1 | ---------- ---------- ---------- ---------- ---------- ---------- ---------- ---------- | 93 |
| PvCRN31 | ---------- ---------- ---------- ---------- ---------- ---------- ---------- ---------- | 129 |
| XP_024577278.1 | ---------- ---------- ---------- ---------- ---------- ---------- ---------- ---------- | 151 |
| XP_024585562.1 | ---------- ---------- ---------- ---------- ---------- ---------- ---------- ---------- | 21 |
| XP_024573944.1 | ---------- ---------- ---------- ---------- ---------- ---------- ---------- ---------- | 117 |
| XP_024579793.1 | ---------- ---------- ---------- ---------- ---------- ---------- ---------- ---------- | 67 |
| PvCRN15 | ---------- ---------- ---------- ---------- ---------- ---------- ---------- ---------- | 145 |
| PvCRN26 | ---------- ---------- ---------- ---------- ---------- ---------- ---------- ---------- | 143 |
| PvCRN16 | ---------- ---------- ---------- ---------- ---------- ---------- ---------- ---------- | 145 |
| PvCRN23 | ---------- ---------- ---------- ---------- ---------- ---------- ---------- ---------- | 165 |
| XP_024573063.1 | ---------- ---------- ---------- ---------- ---------- ---------- ---------- ---------- | 77 |
| PvCRN19 | ---------- ---------- ---------- ---------- ---------- ---------- ---------- ---------- | 128 |
| XP_024581625.1 | ---------- ---------- ---------- ---------- ---------- ---------- ---------- ---------- | 113 |
| XP_024583886.1 | ---------- ---------- ---------- ---------- ---------- ---------- ---------- ---------- | 96 |
| XP_024584409.1 | ---------- ---------- ---------- ---------- ---------- ---------- ---------- ---------- | 114 |
| XP_024572385.1 | ---------- ---------- ---------- ---------- ---------- ---------- ---------- ---------- | 113 |
| PvCRN6 | ---------- ---------- ---------- ---------- ---------- ---------- ---------- ---------- | 124 |
| PvCRN9 | ---------- ---------- ---------- ---------- ---------- ---------- ---------- ---------- | 251 |
| XP_024580875.1 | ---------- ---------- ---------- ---------- ---------- ---------- ---------- ---------- | 87 |
| PvCRN24 | ---------- ---------- ---------- ---------- ---------- ---------- ---------- ---------- | 129 |
| PvCRN35 | ---------- ---------- ---------- ---------- ---------- ---------- ---------- ---------- | 124 |
| PvCRN12 | ---------- ---------- ---------- ---------- ---------- ---------- ---------- ---------- | 128 |
| XP_024576927.1 | ---------- ---------- ---------- ---------- ---------- ---------- ---------- ---------- | 232 |
| XP_024578927.1 | ---------- ---------- ---------- ---------- ---------- ---------- ---------- ---------- | 209 |
| XP_024586212.1 | ---------- ---------- ---------- ---------- ---------- ---------- ---------- ---------- | 247 |
| XP_024578078.1 | ---------- ---------- ---------- ---------- ---------- ---------- ---------- ---------- | 106 |
| XP_024575355.1 | ---------- ---------- ---------- ---------- ---------- ---------- ---------- ---------- | 284 |
| XP_024584527.1 | ---------- ---------- ---------- ---------- ---------- ---------- ---------- ---------- | 118 |
| XP_024586664.1 | ---------- ---------- ---------- ---------- ---------- ---------- ---------- ---------- | 108 |
| PvCRN17 | ---------- ---------- ---------- ---------- ---------- ---------- ---------- ---------- | 128 |
| PvCRN2 | ---------- ---------- ---------- ---------- ---------- ---------- ---------- ---------- | 120 |
| XP_024577181.1 | ---------- ---------- ---------- ---------- ---------- ---------- ---------- ---------- | 83 |
| XP_024581075.1 | ---------- ---------- ---------- ---------- ---------- ---------- ---------- ---------- | 137 |
| XP_024575884.1 | ---------- ---------- ---------- ---------- ---------- ---------- ---------- ---------- | 136 |
| XP_024586872.1 | ---------- ---------- ---------- ---------- ---------- ---------- ---------- ---------- | 136 |
| XP_024573052.1 | ---------- ---------- ---------- ---------- ---------- ---------- ---------- ---------- | 185 |
| XP_024586054.1 | ---------- ---------- ---------- ---------- ---------- ---------- ---------- ---------- | 141 |
| PvCRN27 | ---------- ---------- ---------- ---------- ---------- ---------- ---------- ---------- | 101 |
| PvCRN20 | ---------- ---------- ---------- ---------- ---------- ---------- ---------- ---------- | 143 |
| PvCRN25 | ---------- ---------- ---------- ---------- ---------- ---------- ---------- ---------- | 145 |
| PvCRN1 | ---------- ---------- ---------- ---------- ---------- ---------- ---------- ---------- | 143 |
| PvCRN4 | ---------- ---------- ---------- ---------- ---------- ---------- ---------- ---------- | 148 |
| PvCRN30 | ---------- ---------- ---------- ---------- ---------- ---------- ---------- ---------- | 118 |
| PvCRN29 | ---------- ---------- ---------- ---------- ---------- ---------- ---------- ---------- | 101 |
| XP_024583036.1 | ---------- ---------- ---------- ---------- ---------- ---------- ---------- ---------- | 265 |
| PvCRN10 | ---------- ---------- ---------- ---------- ---------- ---------- ---------- ---------- | 154 |
| XP_024577280.1 | ---------- ---------- ---------- ---------- ---------- ---------- ---------- ---------- | 137 |
| XP_024572924.1 | ---------- ---------- ---------- ---------- ---------- ---------- ---------- ---------- | 232 |
| PvCRN14 | ---------- ---------- ---------- ---------- ---------- ---------- ---------- ---------- | 129 |
| XP_024577521.1 | ---------- ---------- ---------- ---------- ---------- ---------- ---------- ---------- | 73 |
| PvCRN21 | ---------- ---------- ---------- ---------- ---------- ---------- ---------- ---------- | 256 |
| XP_024574966.1 | ---------- ---------- ---------- ---------- ---------- ---------- ---------- ---------- | 136 |
| XP_024575372.1 | ---------- ---------- ---------- ---------- ---------- ---------- ---------- ---------- | 114 |
| XP_024581363.1 | ---------- ---------- ---------- ---------- ---------- ---------- ---------- ---------- | 47 |
| XP_024583883.1 | ---------- ---------- ---------- ---------- ---------- ---------- ---------- ---------- | 86 |
| XP_024579844.1 | ---------- ---------- ---------- ---------- ---------- ---------- ---------- ---------- | 166 |
| XP_024574916.1 | ---------- ---------- ---------- ---------- ---------- ---------- ---------- ---------- | 368 |
| XP_024574193.1 | ---------- ---------- ---------- ---------- ---------- ---------- ---------- ---------- | 337 |
| XP_024583155.1 | ---------- ---------- ---------- ---------- ---------- ---------- ---------- ---------- | 142 |
| XP_024574913.1 | ---------- ---------- ---------- ---------- ---------- ---------- ---------- ---------- | 260 |
| XP_024578158.1 | ---------- ---------- ---------- ---------- ---------- ---------- ---------- ---------- | 84 |
| PvCRN22 | ---------- ---------- ---------- ---------- ---------- ---------- ---------- ---------- | 145 |
| XP_024585001.1 | ---------- ---------- ---------- ---------- ---------- ---------- ---------- ---------- | 75 |
| XP_024583843.1 | ---------- ---------- ---------- ---------- ---------- ---------- ---------- ---------- | 197 |
| XP_024578702.1 | ---------- ---------- ---------- ---------- ---------- ---------- ---------- ---------- | 105 |
| XP_024585805.1 | ---------- ---------- ---------- ---------- ---------- ---------- ---------- ---------- | 155 |
| XP_024580932.1 | ---------- ---------- ---------- ---------- ---------- ---------- ---------- ---------- | 290 |
| Consensus | ---------- ---------- ---------- ---------- ---------- ---------- ---------- ---------- | |
| Conservation (100% / 0%) | | |

| | 5,140 · 5,160 · 5,180 · 5,200 | |
|---|---|---|
| PvCRN11 | ---------- ---------- ---------- ---------- ---------- ---------- ---------- ---------- | 151 |
| XP_024579260.1 | ---------- ---------- ---------- ---------- ---------- ---------- ---------- ---------- | 146 |
| XP_024573634.1 | ---------- ---------- ---------- ---------- ---------- ---------- ---------- ---------- | 134 |
| PvCRN7 | ---------- ---------- ---------- ---------- ---------- ---------- ---------- ---------- | 245 |
| XP_024585265.1 | ---------- ---------- ---------- ---------- ---------- ---------- ---------- ---------- | 126 |
| PvCRN18 | ---------- ---------- ---------- ---------- ---------- ---------- ---------- ---------- | 101 |
| XP_024578414.1 | ---------- ---------- ---------- ---------- ---------- ---------- ---------- ---------- | 444 |
| XP_024579130.1 | ---------- ---------- ---------- ---------- ---------- ---------- ---------- ---------- | 146 |
| XP_024584758.1 | ---------- ---------- ---------- ---------- ---------- ---------- ---------- ---------- | 128 |
| XP_024586066.1 | ---------- ---------- ---------- ---------- ---------- ---------- ---------- ---------- | 152 |
| XP_024585870.1 | ---------- ---------- ---------- ---------- ---------- ---------- ---------- ---------- | 93 |
| PvCRN31 | ---------- ---------- ---------- ---------- ---------- ---------- ---------- ---------- | 129 |
| XP_024577278.1 | ---------- ---------- ---------- ---------- ---------- ---------- ---------- ---------- | 151 |
| XP_024585562.1 | ---------- ---------- ---------- ---------- ---------- ---------- ---------- ---------- | 21 |
| XP_024573944.1 | ---------- ---------- ---------- ---------- ---------- ---------- ---------- ---------- | 117 |
| XP_024579793.1 | ---------- ---------- ---------- ---------- ---------- ---------- ---------- ---------- | 67 |
| PvCRN15 | ---------- ---------- ---------- ---------- ---------- ---------- ---------- ---------- | 145 |
| PvCRN26 | ---------- ---------- ---------- ---------- ---------- ---------- ---------- ---------- | 143 |
| PvCRN16 | ---------- ---------- ---------- ---------- ---------- ---------- ---------- ---------- | 145 |
| PvCRN23 | ---------- ---------- ---------- ---------- ---------- ---------- ---------- ---------- | 165 |
| XP_024573063.1 | ---------- ---------- ---------- ---------- ---------- ---------- ---------- ---------- | 77 |
| PvCRN19 | ---------- ---------- ---------- ---------- ---------- ---------- ---------- ---------- | 128 |
| XP_024581625.1 | ---------- ---------- ---------- ---------- ---------- ---------- ---------- ---------- | 113 |
| XP_024583886.1 | ---------- ---------- ---------- ---------- ---------- ---------- ---------- ---------- | 96 |
| XP_024584409.1 | ---------- ---------- ---------- ---------- ---------- ---------- ---------- ---------- | 114 |
| XP_024572385.1 | ---------- ---------- ---------- ---------- ---------- ---------- ---------- ---------- | 113 |
| PvCRN6 | ---------- ---------- ---------- ---------- ---------- ---------- ---------- ---------- | 124 |
| PvCRN9 | ---------- ---------- ---------- ---------- ---------- ---------- ---------- ---------- | 251 |
| XP_024580875.1 | ---------- ---------- ---------- ---------- ---------- ---------- ---------- ---------- | 87 |
| PvCRN24 | ---------- ---------- ---------- ---------- ---------- ---------- ---------- ---------- | 129 |
| PvCRN35 | ---------- ---------- ---------- ---------- ---------- ---------- ---------- ---------- | 124 |
| PvCRN12 | ---------- ---------- ---------- ---------- ---------- ---------- ---------- ---------- | 128 |
| XP_024576927.1 | ---------- ---------- ---------- ---------- ---------- ---------- ---------- ---------- | 232 |
| XP_024578927.1 | ---------- ---------- ---------- ---------- ---------- ---------- ---------- ---------- | 209 |
| XP_024586212.1 | ---------- ---------- ---------- ---------- ---------- ---------- ---------- ---------- | 247 |
| XP_024578078.1 | ---------- ---------- ---------- ---------- ---------- ---------- ---------- ---------- | 106 |
| XP_024575355.1 | ---------- ---------- ---------- ---------- ---------- ---------- ---------- ---------- | 284 |
| XP_024584527.1 | ---------- ---------- ---------- ---------- ---------- ---------- ---------- ---------- | 118 |
| XP_024586664.1 | ---------- ---------- ---------- ---------- ---------- ---------- ---------- ---------- | 108 |
| PvCRN17 | ---------- ---------- ---------- ---------- ---------- ---------- ---------- ---------- | 128 |
| PvCRN2 | ---------- ---------- ---------- ---------- ---------- ---------- ---------- ---------- | 120 |
| XP_024577181.1 | ---------- ---------- ---------- ---------- ---------- ---------- ---------- ---------- | 83 |
| XP_024581075.1 | ---------- ---------- ---------- ---------- ---------- ---------- ---------- ---------- | 137 |
| XP_024575884.1 | ---------- ---------- ---------- ---------- ---------- ---------- ---------- ---------- | 136 |
| XP_024586872.1 | ---------- ---------- ---------- ---------- ---------- ---------- ---------- ---------- | 136 |
| XP_024573052.1 | ---------- ---------- ---------- ---------- ---------- ---------- ---------- ---------- | 185 |
| XP_024586054.1 | ---------- ---------- ---------- ---------- ---------- ---------- ---------- ---------- | 141 |
| PvCRN27 | ---------- ---------- ---------- ---------- ---------- ---------- ---------- ---------- | 101 |
| PvCRN20 | ---------- ---------- ---------- ---------- ---------- ---------- ---------- ---------- | 143 |
| PvCRN25 | ---------- ---------- ---------- ---------- ---------- ---------- ---------- ---------- | 145 |
| PvCRN1 | ---------- ---------- ---------- ---------- ---------- ---------- ---------- ---------- | 143 |
| PvCRN4 | ---------- ---------- ---------- ---------- ---------- ---------- ---------- ---------- | 148 |
| PvCRN30 | ---------- ---------- ---------- ---------- ---------- ---------- ---------- ---------- | 118 |
| PvCRN29 | ---------- ---------- ---------- ---------- ---------- ---------- ---------- ---------- | 101 |
| XP_024583036.1 | ---------- ---------- ---------- ---------- ---------- ---------- ---------- ---------- | 265 |
| PvCRN10 | ---------- ---------- ---------- ---------- ---------- ---------- ---------- ---------- | 154 |
| XP_024577280.1 | ---------- ---------- ---------- ---------- ---------- ---------- ---------- ---------- | 137 |
| XP_024572924.1 | ---------- ---------- ---------- ---------- ---------- ---------- ---------- ---------- | 232 |
| PvCRN14 | ---------- ---------- ---------- ---------- ---------- ---------- ---------- ---------- | 129 |
| XP_024577521.1 | ---------- ---------- ---------- ---------- ---------- ---------- ---------- ---------- | 73 |
| PvCRN21 | ---------- ---------- ---------- ---------- ---------- ---------- ---------- ---------- | 256 |
| XP_024574966.1 | ---------- ---------- ---------- ---------- ---------- ---------- ---------- ---------- | 136 |
| XP_024575372.1 | ---------- ---------- ---------- ---------- ---------- ---------- ---------- ---------- | 114 |
| XP_024581363.1 | ---------- ---------- ---------- ---------- ---------- ---------- ---------- ---------- | 47 |
| XP_024583883.1 | ---------- ---------- ---------- ---------- ---------- ---------- ---------- ---------- | 86 |
| XP_024579844.1 | ---------- ---------- ---------- ---------- ---------- ---------- ---------- ---------- | 166 |
| XP_024574916.1 | ---------- ---------- ---------- ---------- ---------- ---------- ---------- ---------- | 368 |
| XP_024574193.1 | ---------- ---------- ---------- ---------- ---------- ---------- ---------- ---------- | 337 |
| XP_024583155.1 | ---------- ---------- ---------- ---------- ---------- ---------- ---------- ---------- | 142 |
| XP_024574913.1 | ---------- ---------- ---------- ---------- ---------- ---------- ---------- ---------- | 260 |
| XP_024578158.1 | ---------- ---------- ---------- ---------- ---------- ---------- ---------- ---------- | 84 |
| PvCRN22 | ---------- ---------- ---------- ---------- ---------- ---------- ---------- ---------- | 145 |
| XP_024585001.1 | ---------- ---------- ---------- ---------- ---------- ---------- ---------- ---------- | 75 |
| XP_024583843.1 | ---------- ---------- ---------- ---------- ---------- ---------- ---------- ---------- | 197 |
| XP_024578702.1 | ---------- ---------- ---------- ---------- ---------- ---------- ---------- ---------- | 105 |
| XP_024585805.1 | ---------- ---------- ---------- ---------- ---------- ---------- ---------- ---------- | 155 |
| XP_024580932.1 | ---------- ---------- ---------- ---------- ---------- ---------- ---------- ---------- | 290 |
| Consensus | ---------- ---------- ---------- ---------- ---------- ---------- ---------- ---------- | |
| Conservation (100% – 0%) | | |

| | 5,220 5,240 5,260 5,280 | |
|---|---|---|
| PvCRN11 | ---------- ---------- ---------- ---------- ---------- ---------- ---------- ---------- | 151 |
| XP_024579260.1 | ---------- ---------- ---------- ---------- ---------- ---------- ---------- ---------- | 146 |
| XP_024573634.1 | ---------- ---------- ---------- ---------- ---------- ---------- ---------- ---------- | 134 |
| PvCRN7 | ---------- ---------- ---------- ---------- ---------- ---------- ---------- ---------- | 245 |
| XP_024585265.1 | ---------- ---------- ---------- ---------- ---------- ---------- ---------- ---------- | 126 |
| PvCRN18 | ---------- ---------- ---------- ---------- ---------- ---------- ---------- ---------- | 101 |
| XP_024578414.1 | ---------- ---------- ---------- ---------- ---------- ---------- ---------- ---------- | 444 |
| XP_024579130.1 | ---------- ---------- ---------- ---------- ---------- ---------- ---------- ---------- | 146 |
| XP_024584758.1 | ---------- ---------- ---------- ---------- ---------- ---------- ---------- ---------- | 128 |
| XP_024586066.1 | ---------- ---------- ---------- ---------- ---------- ---------- ---------- ---------- | 152 |
| XP_024585870.1 | ---------- ---------- ---------- ---------- ---------- ---------- ---------- ---------- | 93 |
| PvCRN31 | ---------- ---------- ---------- ---------- ---------- ---------- ---------- ---------- | 129 |
| XP_024577278.1 | ---------- ---------- ---------- ---------- ---------- ---------- ---------- ---------- | 151 |
| XP_024585562.1 | ---------- ---------- ---------- ---------- ---------- ---------- ---------- ---------- | 21 |
| XP_024573944.1 | ---------- ---------- ---------- ---------- ---------- ---------- ---------- ---------- | 117 |
| XP_024579793.1 | ---------- ---------- ---------- ---------- ---------- ---------- ---------- ---------- | 67 |
| PvCRN15 | ---------- ---------- ---------- ---------- ---------- ---------- ---------- ---------- | 145 |
| PvCRN26 | ---------- ---------- ---------- ---------- ---------- ---------- ---------- ---------- | 143 |
| PvCRN16 | ---------- ---------- ---------- ---------- ---------- ---------- ---------- ---------- | 145 |
| PvCRN23 | ---------- ---------- ---------- ---------- ---------- ---------- ---------- ---------- | 165 |
| XP_024573063.1 | ---------- ---------- ---------- ---------- ---------- ---------- ---------- ---------- | 77 |
| PvCRN19 | ---------- ---------- ---------- ---------- ---------- ---------- ---------- ---------- | 128 |
| XP_024581625.1 | ---------- ---------- ---------- ---------- ---------- ---------- ---------- ---------- | 113 |
| XP_024583886.1 | ---------- ---------- ---------- ---------- ---------- ---------- ---------- ---------- | 96 |
| XP_024584409.1 | ---------- ---------- ---------- ---------- ---------- ---------- ---------- ---------- | 114 |
| XP_024572385.1 | ---------- ---------- ---------- ---------- ---------- ---------- ---------- ---------- | 113 |
| PvCRN6 | ---------- ---------- ---------- ---------- ---------- ---------- ---------- ---------- | 124 |
| PvCRN9 | ---------- ---------- ---------- ---------- ---------- ---------- ---------- ---------- | 251 |
| XP_024580875.1 | ---------- ---------- ---------- ---------- ---------- ---------- ---------- ---------- | 87 |
| PvCRN24 | ---------- ---------- ---------- ---------- ---------- ---------- ---------- ---------- | 129 |
| PvCRN35 | ---------- ---------- ---------- ---------- ---------- ---------- ---------- ---------- | 124 |
| PvCRN12 | ---------- ---------- ---------- ---------- ---------- ---------- ---------- ---------- | 128 |
| XP_024576927.1 | ---------- ---------- ---------- ---------- ---------- ---------- ---------- ---------- | 232 |
| XP_024578927.1 | ---------- ---------- ---------- ---------- ---------- ---------- ---------- ---------- | 209 |
| XP_024586212.1 | ---------- ---------- ---------- ---------- ---------- ---------- ---------- ---------- | 247 |
| XP_024578078.1 | ---------- ---------- ---------- ---------- ---------- ---------- ---------- ---------- | 106 |
| XP_024575355.1 | ---------- ---------- ---------- ---------- ---------- ---------- ---------- ---------- | 284 |
| XP_024584527.1 | ---------- ---------- ---------- ---------- ---------- ---------- ---------- ---------- | 118 |
| XP_024586664.1 | ---------- ---------- ---------- ---------- ---------- ---------- ---------- ---------- | 108 |
| PvCRN17 | ---------- ---------- ---------- ---------- ---------- ---------- ---------- ---------- | 128 |
| PvCRN2 | ---------- ---------- ---------- ---------- ---------- ---------- ---------- ---------- | 120 |
| XP_024577181.1 | ---------- ---------- ---------- ---------- ---------- ---------- ---------- ---------- | 83 |
| XP_024581075.1 | ---------- ---------- ---------- ---------- ---------- ---------- ---------- ---------- | 137 |
| XP_024575884.1 | ---------- ---------- ---------- ---------- ---------- ---------- ---------- ---------- | 136 |
| XP_024586872.1 | ---------- ---------- ---------- ---------- ---------- ---------- ---------- ---------- | 136 |
| XP_024573052.1 | ---------- ---------- ---------- ---------- ---------- ---------- ---------- ---------- | 185 |
| XP_024586054.1 | ---------- ---------- ---------- ---------- ---------- ---------- ---------- ---------- | 141 |
| PvCRN27 | ---------- ---------- ---------- ---------- ---------- ---------- ---------- ---------- | 101 |
| PvCRN20 | ---------- ---------- ---------- ---------- ---------- ---------- ---------- ---------- | 143 |
| PvCRN25 | ---------- ---------- ---------- ---------- ---------- ---------- ---------- ---------- | 145 |
| PvCRN1 | ---------- ---------- ---------- ---------- ---------- ---------- ---------- ---------- | 143 |
| PvCRN4 | ---------- ---------- ---------- ---------- ---------- ---------- ---------- ---------- | 148 |
| PvCRN30 | ---------- ---------- ---------- ---------- ---------- ---------- ---------- ---------- | 118 |
| PvCRN29 | ---------- ---------- ---------- ---------- ---------- ---------- ---------- ---------- | 101 |
| XP_024583036.1 | ---------- ---------- ---------- ---------- ---------- ---------- ---------- ---------- | 265 |
| PvCRN10 | ---------- ---------- ---------- ---------- ---------- ---------- ---------- ---------- | 154 |
| XP_024577280.1 | ---------- ---------- ---------- ---------- ---------- ---------- ---------- ---------- | 137 |
| XP_024572924.1 | ---------- ---------- ---------- ---------- ---------- ---------- ---------- ---------- | 232 |
| PvCRN14 | ---------- ---------- ---------- ---------- ---------- ---------- ---------- ---------- | 129 |
| XP_024577521.1 | ---------- ---------- ---------- ---------- ---------- ---------- ---------- ---------- | 73 |
| PvCRN21 | ---------- ---------- ---------- ---------- ---------- ---------- ---------- ---------- | 256 |
| XP_024574966.1 | ---------- ---------- ---------- ---------- ---------- ---------- ---------- ---------- | 136 |
| XP_024575372.1 | ---------- ---------- ---------- ---------- ---------- ---------- ---------- ---------- | 114 |
| XP_024581363.1 | ---------- ---------- ---------- ---------- ---------- ---------- ---------- ---------- | 47 |
| XP_024583883.1 | ---------- ---------- ---------- ---------- ---------- ---------- ---------- ---------- | 86 |
| XP_024579844.1 | ---------- ---------- ---------- ---------- ---------- ---------- ---------- ---------- | 166 |
| XP_024574916.1 | ---------- ---------- ---------- ---------- ---------- ---------- ---------- ---------- | 368 |
| XP_024574193.1 | ---------- ---------- ---------- ---------- ---------- ---------- ---------- ---------- | 337 |
| XP_024583155.1 | ---------- ---------- ---------- ---------- ---------- ---------- ---------- ---------- | 142 |
| XP_024574913.1 | ---------- ---------- ---------- ---------- ---------- ---------- ---------- ---------- | 260 |
| XP_024578158.1 | ---------- ---------- ---------- ---------- ---------- ---------- ---------- ---------- | 84 |
| PvCRN22 | ---------- ---------- ---------- ---------- ---------- ---------- ---------- ---------- | 145 |
| XP_024585001.1 | ---------- ---------- ---------- ---------- ---------- ---------- ---------- ---------- | 75 |
| XP_024583843.1 | ---------- ---------- ---------- ---------- ---------- ---------- ---------- ---------- | 197 |
| XP_024578702.1 | ---------- ---------- ---------- ---------- ---------- ---------- ---------- ---------- | 105 |
| XP_024585805.1 | ---------- ---------- ---------- ---------- ---------- ---------- ---------- ---------- | 155 |
| XP_024580932.1 | ---------- ---------- ---------- ---------- ---------- ---------- ---------- ---------- | 290 |
| Consensus | ---------- ---------- ---------- ---------- ---------- ---------- ---------- ---------- | |
| Conservation (100% / 0%) | | |

| | 5,300 · 5,320 · 5,340 · 5,360 | |
|---|---|---|
| PvCRN11 | ---------- ---------- ---------- ---------- ---------- ---------- ---------- ---------- | 151 |
| XP_024579260.1 | ---------- ---------- ---------- ---------- ---------- ---------- ---------- ---------- | 146 |
| XP_024573634.1 | ---------- ---------- ---------- ---------- ---------- ---------- ---------- ---------- | 134 |
| PvCRN7 | ---------- ---------- ---------- ---------- ---------- ---------- ---------- ---------- | 245 |
| XP_024585265.1 | ---------- ---------- ---------- ---------- ---------- ---------- ---------- ---------- | 126 |
| PvCRN18 | ---------- ---------- ---------- ---------- ---------- ---------- ---------- ---------- | 101 |
| XP_024578414.1 | ---------- ---------- ---------- ---------- ---------- ---------- ---------- ---------- | 444 |
| XP_024579130.1 | ---------- ---------- ---------- ---------- ---------- ---------- ---------- ---------- | 146 |
| XP_024584758.1 | ---------- ---------- ---------- ---------- ---------- ---------- ---------- ---------- | 128 |
| XP_024586066.1 | ---------- ---------- ---------- ---------- ---------- ---------- ---------- ---------- | 152 |
| XP_024585870.1 | ---------- ---------- ---------- ---------- ---------- ---------- ---------- ---------- | 93 |
| PvCRN31 | ---------- ---------- ---------- ---------- ---------- ---------- ---------- ---------- | 129 |
| XP_024577278.1 | ---------- ---------- ---------- ---------- ---------- ---------- ---------- ---------- | 151 |
| XP_024585562.1 | ---------- ---------- ---------- ---------- ---------- ---------- ---------- ---------- | 21 |
| XP_024573944.1 | ---------- ---------- ---------- ---------- ---------- ---------- ---------- ---------- | 117 |
| XP_024579793.1 | ---------- ---------- ---------- ---------- ---------- ---------- ---------- ---------- | 67 |
| PvCRN15 | ---------- ---------- ---------- ---------- ---------- ---------- ---------- ---------- | 145 |
| PvCRN26 | ---------- ---------- ---------- ---------- ---------- ---------- ---------- ---------- | 143 |
| PvCRN16 | ---------- ---------- ---------- ---------- ---------- ---------- ---------- ---------- | 145 |
| PvCRN23 | ---------- ---------- ---------- ---------- ---------- ---------- ---------- ---------- | 165 |
| XP_024573063.1 | ---------- ---------- ---------- ---------- ---------- ---------- ---------- ---------- | 77 |
| PvCRN19 | ---------- ---------- ---------- ---------- ---------- ---------- ---------- ---------- | 128 |
| XP_024581625.1 | ---------- ---------- ---------- ---------- ---------- ---------- ---------- ---------- | 113 |
| XP_024583886.1 | ---------- ---------- ---------- ---------- ---------- ---------- ---------- ---------- | 96 |
| XP_024584409.1 | ---------- ---------- ---------- ---------- ---------- ---------- ---------- ---------- | 114 |
| XP_024572385.1 | ---------- ---------- ---------- ---------- ---------- ---------- ---------- ---------- | 113 |
| PvCRN6 | ---------- ---------- ---------- ---------- ---------- ---------- ---------- ---------- | 124 |
| PvCRN9 | ---------- ---------- ---------- ---------- ---------- ---------- ---------- ---------- | 251 |
| XP_024580875.1 | ---------- ---------- ---------- ---------- ---------- ---------- ---------- ---------- | 87 |
| PvCRN24 | ---------- ---------- ---------- ---------- ---------- ---------- ---------- ---------- | 129 |
| PvCRN35 | ---------- ---------- ---------- ---------- ---------- ---------- ---------- ---------- | 124 |
| PvCRN12 | ---------- ---------- ---------- ---------- ---------- ---------- ---------- ---------- | 128 |
| XP_024576927.1 | ---------- ---------- ---------- ---------- ---------- ---------- ---------- ---------- | 232 |
| XP_024578927.1 | ---------- ---------- ---------- ---------- ---------- ---------- ---------- ---------- | 209 |
| XP_024586212.1 | ---------- ---------- ---------- ---------- ---------- ---------- ---------- ---------- | 247 |
| XP_024578078.1 | ---------- ---------- ---------- ---------- ---------- ---------- ---------- ---------- | 106 |
| XP_024575355.1 | ---------- ---------- ---------- ---------- ---------- ---------- ---------- ---------- | 284 |
| XP_024584527.1 | ---------- ---------- ---------- ---------- ---------- ---------- ---------- ---------- | 118 |
| XP_024586664.1 | ---------- ---------- ---------- ---------- ---------- ---------- ---------- ---------- | 108 |
| PvCRN17 | ---------- ---------- ---------- ---------- ---------- ---------- ---------- ---------- | 128 |
| PvCRN2 | ---------- ---------- ---------- ---------- ---------- ---------- ---------- ---------- | 120 |
| XP_024577181.1 | ---------- ---------- ---------- ---------- ---------- ---------- ---------- ---------- | 83 |
| XP_024581075.1 | ---------- ---------- ---------- ---------- ---------- ---------- ---------- ---------- | 137 |
| XP_024575884.1 | ---------- ---------- ---------- ---------- ---------- ---------- ---------- ---------- | 136 |
| XP_024586872.1 | ---------- ---------- ---------- ---------- ---------- ---------- ---------- ---------- | 136 |
| XP_024573052.1 | ---------- ---------- ---------- ---------- ---------- ---------- ---------- ---------- | 185 |
| XP_024586054.1 | ---------- ---------- ---------- ---------- ---------- ---------- ---------- ---------- | 141 |
| PvCRN27 | ---------- ---------- ---------- ---------- ---------- ---------- ---------- ---------- | 101 |
| PvCRN20 | ---------- ---------- ---------- ---------- ---------- ---------- ---------- ---------- | 143 |
| PvCRN25 | ---------- ---------- ---------- ---------- ---------- ---------- ---------- ---------- | 145 |
| PvCRN1 | ---------- ---------- ---------- ---------- ---------- ---------- ---------- ---------- | 143 |
| PvCRN4 | ---------- ---------- ---------- ---------- ---------- ---------- ---------- ---------- | 148 |
| PvCRN30 | ---------- ---------- ---------- ---------- ---------- ---------- ---------- ---------- | 118 |
| PvCRN29 | ---------- ---------- ---------- ---------- ---------- ---------- ---------- ---------- | 101 |
| XP_024583036.1 | ---------- ---------- ---------- ---------- ---------- ---------- ---------- ---------- | 265 |
| PvCRN10 | ---------- ---------- ---------- ---------- ---------- ---------- ---------- ---------- | 154 |
| XP_024577280.1 | ---------- ---------- ---------- ---------- ---------- ---------- ---------- ---------- | 137 |
| XP_024572924.1 | ---------- ---------- ---------- ---------- ---------- ---------- ---------- ---------- | 232 |
| PvCRN14 | ---------- ---------- ---------- ---------- ---------- ---------- ---------- ---------- | 129 |
| XP_024577521.1 | ---------- ---------- ---------- ---------- ---------- ---------- ---------- ---------- | 73 |
| PvCRN21 | ---------- ---------- ---------- ---------- ---------- ---------- ---------- ---------- | 256 |
| XP_024574966.1 | ---------- ---------- ---------- ---------- ---------- ---------- ---------- ---------- | 136 |
| XP_024575372.1 | ---------- ---------- ---------- ---------- ---------- ---------- ---------- ---------- | 114 |
| XP_024581363.1 | ---------- ---------- ---------- ---------- ---------- ---------- ---------- ---------- | 47 |
| XP_024583883.1 | ---------- ---------- ---------- ---------- ---------- ---------- ---------- ---------- | 86 |
| XP_024579844.1 | ---------- ---------- ---------- ---------- ---------- ---------- ---------- ---------- | 166 |
| XP_024574916.1 | ---------- ---------- ---------- ---------- ---------- ---------- ---------- ---------- | 368 |
| XP_024574193.1 | ---------- ---------- ---------- ---------- ---------- ---------- ---------- ---------- | 337 |
| XP_024583155.1 | ---------- ---------- ---------- ---------- ---------- ---------- ---------- ---------- | 142 |
| XP_024574913.1 | ---------- ---------- ---------- ---------- ---------- ---------- ---------- ---------- | 260 |
| XP_024578158.1 | ---------- ---------- ---------- ---------- ---------- ---------- ---------- ---------- | 84 |
| PvCRN22 | ---------- ---------- ---------- ---------- ---------- ---------- ---------- ---------- | 145 |
| XP_024585001.1 | ---------- ---------- ---------- ---------- ---------- ---------- ---------- ---------- | 75 |
| XP_024583843.1 | ---------- ---------- ---------- ---------- ---------- ---------- ---------- ---------- | 197 |
| XP_024578702.1 | ---------- ---------- ---------- ---------- ---------- ---------- ---------- ---------- | 105 |
| XP_024585805.1 | ---------- ---------- ---------- ---------- ---------- ---------- ---------- ---------- | 155 |
| XP_024580932.1 | ---------- ---------- ---------- ---------- ---------- ---------- ---------- ---------- | 290 |
| Consensus | ---------- ---------- ---------- ---------- ---------- ---------- ---------- ---------- | |
| Conservation (100% – 0%) | | |

```
                       5,380                5,400                5,420                5,440
                           |                    |                    |                    |
PvCRN11         ---------- ---------- ---------- ---------- ---------- ---------- ---------- ---------- 151
XP_024579260.1  ---------- ---------- ---------- ---------- ---------- ---------- ---------- ---------- 146
XP_024573634.1  ---------- ---------- ---------- ---------- ---------- ---------- ---------- ---------- 134
PvCRN7          ---------- ---------- ---------- ---------- ---------- ---------- ---------- ---------- 245
XP_024585265.1  ---------- ---------- ---------- ---------- ---------- ---------- ---------- ---------- 126
PvCRN18         ---------- ---------- ---------- ---------- ---------- ---------- ---------- ---------- 101
XP_024578414.1  ---------- ---------- ---------- ---------- ---------- ---------- ---------- ---------- 444
XP_024579130.1  ---------- ---------- ---------- ---------- ---------- ---------- ---------- ---------- 146
XP_024584758.1  ---------- ---------- ---------- ---------- ---------- ---------- ---------- ---------- 128
XP_024586066.1  ---------- ---------- ---------- ---------- ---------- ---------- ---------- ---------- 152
XP_024585870.1  ---------- ---------- ---------- ---------- ---------- ---------- ---------- ---------- 93
PvCRN31         ---------- ---------- ---------- ---------- ---------- ---------- ---------- ---------- 129
XP_024577278.1  ---------- ---------- ---------- ---------- ---------- ---------- ---------- ---------- 151
XP_024585562.1  ---------- ---------- ---------- ---------- ---------- ---------- ---------- ---------- 21
XP_024573944.1  ---------- ---------- ---------- ---------- ---------- ---------- ---------- ---------- 117
XP_024579793.1  ---------- ---------- ---------- ---------- ---------- ---------- ---------- ---------- 67
PvCRN15         ---------- ---------- ---------- ---------- ---------- ---------- ---------- ---------- 145
PvCRN26         ---------- ---------- ---------- ---------- ---------- ---------- ---------- ---------- 143
PvCRN16         ---------- ---------- ---------- ---------- ---------- ---------- ---------- ---------- 145
PvCRN23         ---------- ---------- ---------- ---------- ---------- ---------- ---------- ---------- 165
XP_024573063.1  ---------- ---------- ---------- ---------- ---------- ---------- ---------- ---------- 77
PvCRN19         ---------- ---------- ---------- ---------- ---------- ---------- ---------- ---------- 128
XP_024581625.1  ---------- ---------- ---------- ---------- ---------- ---------- ---------- ---------- 113
XP_024583886.1  ---------- ---------- ---------- ---------- ---------- ---------- ---------- ---------- 96
XP_024584409.1  ---------- ---------- ---------- ---------- ---------- ---------- ---------- ---------- 114
XP_024572385.1  ---------- ---------- ---------- ---------- ---------- ---------- ---------- ---------- 113
PvCRN6          ---------- ---------- ---------- ---------- ---------- ---------- ---------- ---------- 124
PvCRN9          ---------- ---------- ---------- ---------- ---------- ---------- ---------- ---------- 251
XP_024580875.1  ---------- ---------- ---------- ---------- ---------- ---------- ---------- ---------- 87
PvCRN24         ---------- ---------- ---------- ---------- ---------- ---------- ---------- ---------- 129
PvCRN35         ---------- ---------- ---------- ---------- ---------- ---------- ---------- ---------- 124
PvCRN12         ---------- ---------- ---------- ---------- ---------- ---------- ---------- ---------- 128
XP_024576927.1  ---------- ---------- ---------- ---------- ---------- ---------- ---------- ---------- 232
XP_024578927.1  ---------- ---------- ---------- ---------- ---------- ---------- ---------- ---------- 209
XP_024586212.1  ---------- ---------- ---------- ---------- ---------- ---------- ---------- ---------- 247
XP_024578078.1  ---------- ---------- ---------- ---------- ---------- ---------- ---------- ---------- 106
XP_024575355.1  ---------- ---------- ---------- ---------- ---------- ---------- ---------- ---------- 284
XP_024584527.1  ---------- ---------- ---------- ---------- ---------- ---------- ---------- ---------- 118
XP_024586664.1  ---------- ---------- ---------- ---------- ---------- ---------- ---------- ---------- 108
PvCRN17         ---------- ---------- ---------- ---------- ---------- ---------- ---------- ---------- 128
PvCRN2          ---------- ---------- ---------- ---------- ---------- ---------- ---------- ---------- 120
XP_024577181.1  ---------- ---------- ---------- ---------- ---------- ---------- ---------- ---------- 83
XP_024581075.1  ---------- ---------- ---------- ---------- ---------- ---------- ---------- ---------- 137
XP_024575884.1  ---------- ---------- ---------- ---------- ---------- ---------- ---------- ---------- 136
XP_024586872.1  ---------- ---------- ---------- ---------- ---------- ---------- ---------- ---------- 136
XP_024573052.1  ---------- ---------- ---------- ---------- ---------- ---------- ---------- ---------- 185
XP_024586054.1  ---------- ---------- ---------- ---------- ---------- ---------- ---------- ---------- 141
PvCRN27         ---------- ---------- ---------- ---------- ---------- ---------- ---------- ---------- 101
PvCRN20         ---------- ---------- ---------- ---------- ---------- ---------- ---------- ---------- 143
PvCRN25         ---------- ---------- ---------- ---------- ---------- ---------- ---------- ---------- 145
PvCRN1          ---------- ---------- ---------- ---------- ---------- ---------- ---------- ---------- 143
PvCRN4          ---------- ---------- ---------- ---------- ---------- ---------- ---------- ---------- 148
PvCRN30         ---------- ---------- ---------- ---------- ---------- ---------- ---------- ---------- 118
PvCRN29         ---------- ---------- ---------- ---------- ---------- ---------- ---------- ---------- 101
XP_024583036.1  ---------- ---------- ---------- ---------- ---------- ---------- ---------- ---------- 265
PvCRN10         ---------- ---------- ---------- ---------- ---------- ---------- ---------- ---------- 154
XP_024577280.1  ---------- ---------- ---------- ---------- ---------- ---------- ---------- ---------- 137
XP_024572924.1  ---------- ---------- ---------- ---------- ---------- ---------- ---------- ---------- 232
PvCRN14         ---------- ---------- ---------- ---------- ---------- ---------- ---------- ---------- 129
XP_024577521.1  ---------- ---------- ---------- ---------- ---------- ---------- ---------- ---------- 73
PvCRN21         ---------- ---------- ---------- ---------- ---------- ---------- ---------- ---------- 256
XP_024574966.1  ---------- ---------- ---------- ---------- ---------- ---------- ---------- ---------- 136
XP_024575372.1  ---------- ---------- ---------- ---------- ---------- ---------- ---------- ---------- 114
XP_024581363.1  ---------- ---------- ---------- ---------- ---------- ---------- ---------- ---------- 47
XP_024583883.1  ---------- ---------- ---------- ---------- ---------- ---------- ---------- ---------- 86
XP_024579844.1  ---------- ---------- ---------- ---------- ---------- ---------- ---------- ---------- 166
XP_024574916.1  ---------- ---------- ---------- ---------- ---------- ---------- ---------- ---------- 368
XP_024574193.1  ---------- ---------- ---------- ---------- ---------- ---------- ---------- ---------- 337
XP_024583155.1  ---------- ---------- ---------- ---------- ---------- ---------- ---------- ---------- 142
XP_024574913.1  ---------- ---------- ---------- ---------- ---------- ---------- ---------- ---------- 260
XP_024578158.1  ---------- ---------- ---------- ---------- ---------- ---------- ---------- ---------- 84
PvCRN22         ---------- ---------- ---------- ---------- ---------- ---------- ---------- ---------- 145
XP_024585001.1  ---------- ---------- ---------- ---------- ---------- ---------- ---------- ---------- 75
XP_024583843.1  ---------- ---------- ---------- ---------- ---------- ---------- ---------- ---------- 197
XP_024578702.1  ---------- ---------- ---------- ---------- ---------- ---------- ---------- ---------- 105
XP_024585805.1  ---------- ---------- ---------- ---------- ---------- ---------- ---------- ---------- 155
XP_024580932.1  ---------- ---------- ---------- ---------- ---------- ---------- ---------- ---------- 290
Consensus       ---------- ---------- ---------- ---------- ---------- ---------- ---------- ----------
           100%
Conservation
             0%
```

```
                 5,460               5,480               5,500               5,520
PvCRN11          ---------- ---------- ---------- ---------- ---------- ---------- ---------- ---------- 151
XP_024579260.1   ---------- ---------- ---------- ---------- ---------- ---------- ---------- ---------- 146
XP_024573634.1   ---------- ---------- ---------- ---------- ---------- ---------- ---------- ---------- 134
PvCRN7           ---------- ---------- ---------- ---------- ---------- ---------- ---------- ---------- 245
XP_024585265.1   ---------- ---------- ---------- ---------- ---------- ---------- ---------- ---------- 126
PvCRN18          ---------- ---------- ---------- ---------- ---------- ---------- ---------- ---------- 101
XP_024578414.1   ---------- ---------- ---------- ---------- ---------- ---------- ---------- ---------- 444
XP_024579130.1   ---------- ---------- ---------- ---------- ---------- ---------- ---------- ---------- 146
XP_024584758.1   ---------- ---------- ---------- ---------- ---------- ---------- ---------- ---------- 128
XP_024586066.1   ---------- ---------- ---------- ---------- ---------- ---------- ---------- ---------- 152
XP_024585870.1   ---------- ---------- ---------- ---------- ---------- ---------- ---------- ---------- 93
PvCRN31          ---------- ---------- ---------- ---------- ---------- ---------- ---------- ---------- 129
XP_024577278.1   ---------- ---------- ---------- ---------- ---------- ---------- ---------- ---------- 151
XP_024585562.1   ---------- ---------- ---------- ---------- ---------- ---------- ---------- ---------- 21
XP_024573944.1   ---------- ---------- ---------- ---------- ---------- ---------- ---------- ---------- 117
XP_024579793.1   ---------- ---------- ---------- ---------- ---------- ---------- ---------- ---------- 67
PvCRN15          ---------- ---------- ---------- ---------- ---------- ---------- ---------- ---------- 145
PvCRN26          ---------- ---------- ---------- ---------- ---------- ---------- ---------- ---------- 143
PvCRN16          ---------- ---------- ---------- ---------- ---------- ---------- ---------- ---------- 145
PvCRN23          ---------- ---------- ---------- ---------- ---------- ---------- ---------- ---------- 165
XP_024573063.1   ---------- ---------- ---------- ---------- ---------- ---------- ---------- ---------- 77
PvCRN19          ---------- ---------- ---------- ---------- ---------- ---------- ---------- ---------- 128
XP_024581625.1   ---------- ---------- ---------- ---------- ---------- ---------- ---------- ---------- 113
XP_024583886.1   ---------- ---------- ---------- ---------- ---------- ---------- ---------- ---------- 96
XP_024584409.1   ---------- ---------- ---------- ---------- ---------- ---------- ---------- ---------- 114
XP_024572385.1   ---------- ---------- ---------- ---------- ---------- ---------- ---------- ---------- 113
PvCRN6           ---------- ---------- ---------- ---------- ---------- ---------- ---------- ---------- 124
PvCRN9           ---------- ---------- ---------- ---------- ---------- ---------- ---------- ---------- 251
XP_024580875.1   ---------- ---------- ---------- ---------- ---------- ---------- ---------- ---------- 87
PvCRN24          ---------- ---------- ---------- ---------- ---------- ---------- ---------- ---------- 129
PvCRN35          ---------- ---------- ---------- ---------- ---------- ---------- ---------- ---------- 124
PvCRN12          ---------- ---------- ---------- ---------- ---------- ---------- ---------- ---------- 128
XP_024576927.1   ---------- ---------- ---------- ---------- ---------- ---------- ---------- ---------- 232
XP_024578927.1   ---------- ---------- ---------- ---------- ---------- ---------- ---------- ---------- 209
XP_024586212.1   ---------- ---------- ---------- ---------- ---------- ---------- ---------- ---------- 247
XP_024578078.1   ---------- ---------- ---------- ---------- ---------- ---------- ---------- ---------- 106
XP_024575355.1   ---------- ---------- ---------- ---------- ---------- ---------- ---------- ---------- 284
XP_024584527.1   ---------- ---------- ---------- ---------- ---------- ---------- ---------- ---------- 118
XP_024586664.1   ---------- ---------- ---------- ---------- ---------- ---------- ---------- ---------- 108
PvCRN17          ---------- ---------- ---------- ---------- ---------- ---------- ---------- ---------- 128
PvCRN2           ---------- ---------- ---------- ---------- ---------- ---------- ---------- ---------- 120
XP_024577181.1   ---------- ---------- ---------- ---------- ---------- ---------- ---------- ---------- 83
XP_024581075.1   ---------- ---------- ---------- ---------- ---------- ---------- ---------- ---------- 137
XP_024575884.1   ---------- ---------- ---------- ---------- ---------- ---------- ---------- ---------- 136
XP_024586872.1   ---------- ---------- ---------- ---------- ---------- ---------- ---------- ---------- 136
XP_024573052.1   ---------- ---------- ---------- ---------- ---------- ---------- ---------- ---------- 185
XP_024586054.1   ---------- ---------- ---------- ---------- ---------- ---------- ---------- ---------- 141
PvCRN27          ---------- ---------- ---------- ---------- ---------- ---------- ---------- ---------- 101
PvCRN20          ---------- ---------- ---------- ---------- ---------- ---------- ---------- ---------- 143
PvCRN25          ---------- ---------- ---------- ---------- ---------- ---------- ---------- ---------- 145
PvCRN1           ---------- ---------- ---------- ---------- ---------- ---------- ---------- ---------- 143
PvCRN4           ---------- ---------- ---------- ---------- ---------- ---------- ---------- ---------- 148
PvCRN30          ---------- ---------- ---------- ---------- ---------- ---------- ---------- ---------- 118
PvCRN29          ---------- ---------- ---------- ---------- ---------- ---------- ---------- ---------- 101
XP_024583036.1   ---------- ---------- ---------- ---------- ---------- ---------- ---------- ---------- 265
PvCRN10          ---------- ---------- ---------- ---------- ---------- ---------- ---------- ---------- 154
XP_024577280.1   ---------- ---------- ---------- ---------- ---------- ---------- ---------- ---------- 137
XP_024572924.1   ---------- ---------- ---------- ---------- ---------- ---------- ---------- ---------- 232
PvCRN14          ---------- ---------- ---------- ---------- ---------- ---------- ---------- ---------- 129
XP_024577521.1   ---------- ---------- ---------- ---------- ---------- ---------- ---------- ---------- 73
PvCRN21          ---------- ---------- ---------- ---------- ---------- ---------- ---------- ---------- 256
XP_024574966.1   ---------- ---------- ---------- ---------- ---------- ---------- ---------- ---------- 136
XP_024575372.1   ---------- ---------- ---------- ---------- ---------- ---------- ---------- ---------- 114
XP_024581363.1   ---------- ---------- ---------- ---------- ---------- ---------- ---------- ---------- 47
XP_024583883.1   ---------- ---------- ---------- ---------- ---------- ---------- ---------- ---------- 86
XP_024579844.1   ---------- ---------- ---------- ---------- ---------- ---------- ---------- ---------- 166
XP_024574916.1   ---------- ---------- ---------- ---------- ---------- ---------- ---------- ---------- 368
XP_024574193.1   ---------- ---------- ---------- ---------- ---------- ---------- ---------- ---------- 337
XP_024583155.1   ---------- ---------- ---------- ---------- ---------- ---------- ---------- ---------- 142
XP_024574913.1   ---------- ---------- ---------- ---------- ---------- ---------- ---------- ---------- 260
XP_024578158.1   ---------- ---------- ---------- ---------- ---------- ---------- ---------- ---------- 84
PvCRN22          ---------- ---------- ---------- ---------- ---------- ---------- ---------- ---------- 145
XP_024585001.1   ---------- ---------- ---------- ---------- ---------- ---------- ---------- ---------- 75
XP_024583843.1   ---------- ---------- ---------- ---------- ---------- ---------- ---------- ---------- 197
XP_024578702.1   ---------- ---------- ---------- ---------- ---------- ---------- ---------- ---------- 105
XP_024585805.1   ---------- ---------- ---------- ---------- ---------- ---------- ---------- ---------- 155
XP_024580932.1   ---------- ---------- ---------- ---------- ---------- ---------- ---------- ---------- 290
Consensus        ---------- ---------- ---------- ---------- ---------- ---------- ---------- ----------
             100%
Conservation
               0%
```

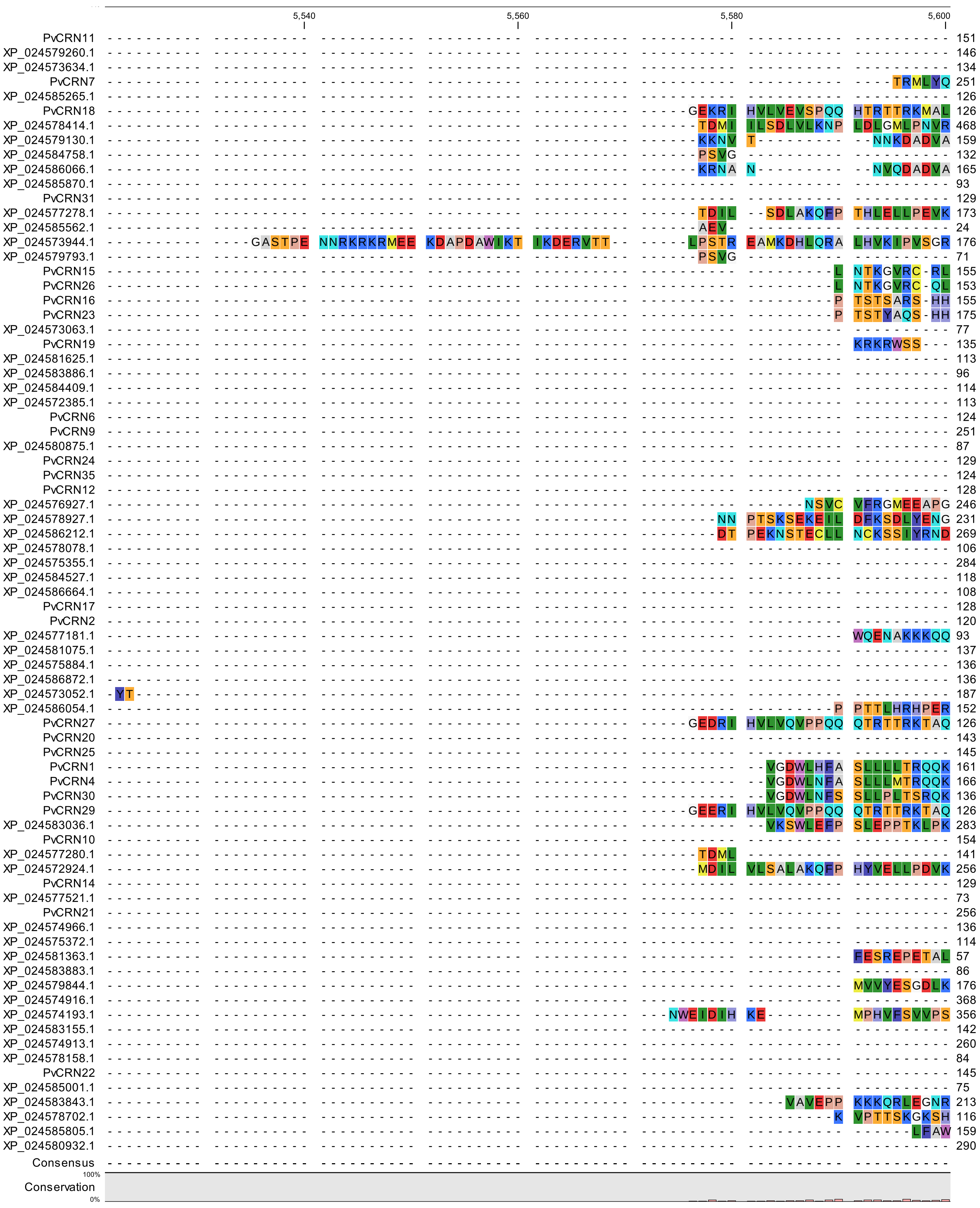
5,540
5,560
5,580
5,600
PvCRN11 151
XP_024579260.1 146
XP_024573634.1 134
PvCRN7 251
XP_024585265.1 126
PvCRN18 126
XP_024578414.1 468
XP_024579130.1 159
XP_024584758.1 132
XP_024586066.1 165
XP_024585870.1 93
PvCRN31 129
XP_024577278.1 173
XP_024585562.1 24
XP_024573944.1 176
XP_024579793.1 71
PvCRN15 155
PvCRN26 153
PvCRN16 155
PvCRN23 175
XP_024573063.1 77
PvCRN19 135
XP_024581625.1 113
XP_024583886.1 96
XP_024584409.1 114
XP_024572385.1 113
PvCRN6 124
PvCRN9 251
XP_024580875.1 87
PvCRN24 129
PvCRN35 124
PvCRN12 128
XP_024576927.1 246
XP_024578927.1 231
XP_024586212.1 269
XP_024578078.1 106
XP_024575355.1 284
XP_024584527.1 118
XP_024586664.1 108
PvCRN17 128
PvCRN2 120
XP_024577181.1 93
XP_024581075.1 137
XP_024575884.1 136
XP_024586872.1 136
XP_024573052.1 187
XP_024586054.1 152
PvCRN27 126
PvCRN20 143
PvCRN25 145
PvCRN1 161
PvCRN4 166
PvCRN30 136
PvCRN29 126
XP_024583036.1 283
PvCRN10 154
XP_024577280.1 141
XP_024572924.1 256
PvCRN14 129
XP_024577521.1 73
PvCRN21 256
XP_024574966.1 136
XP_024575372.1 114
XP_024581363.1 57
XP_024583883.1 86
XP_024579844.1 176
XP_024574916.1 368
XP_024574193.1 356
XP_024583155.1 142
XP_024574913.1 260
XP_024578158.1 84
PvCRN22 145
XP_024585001.1 75
XP_024583843.1 213
XP_024578702.1 116
XP_024585805.1 159
XP_024580932.1 290
Consensus
Conservation
100%
0%

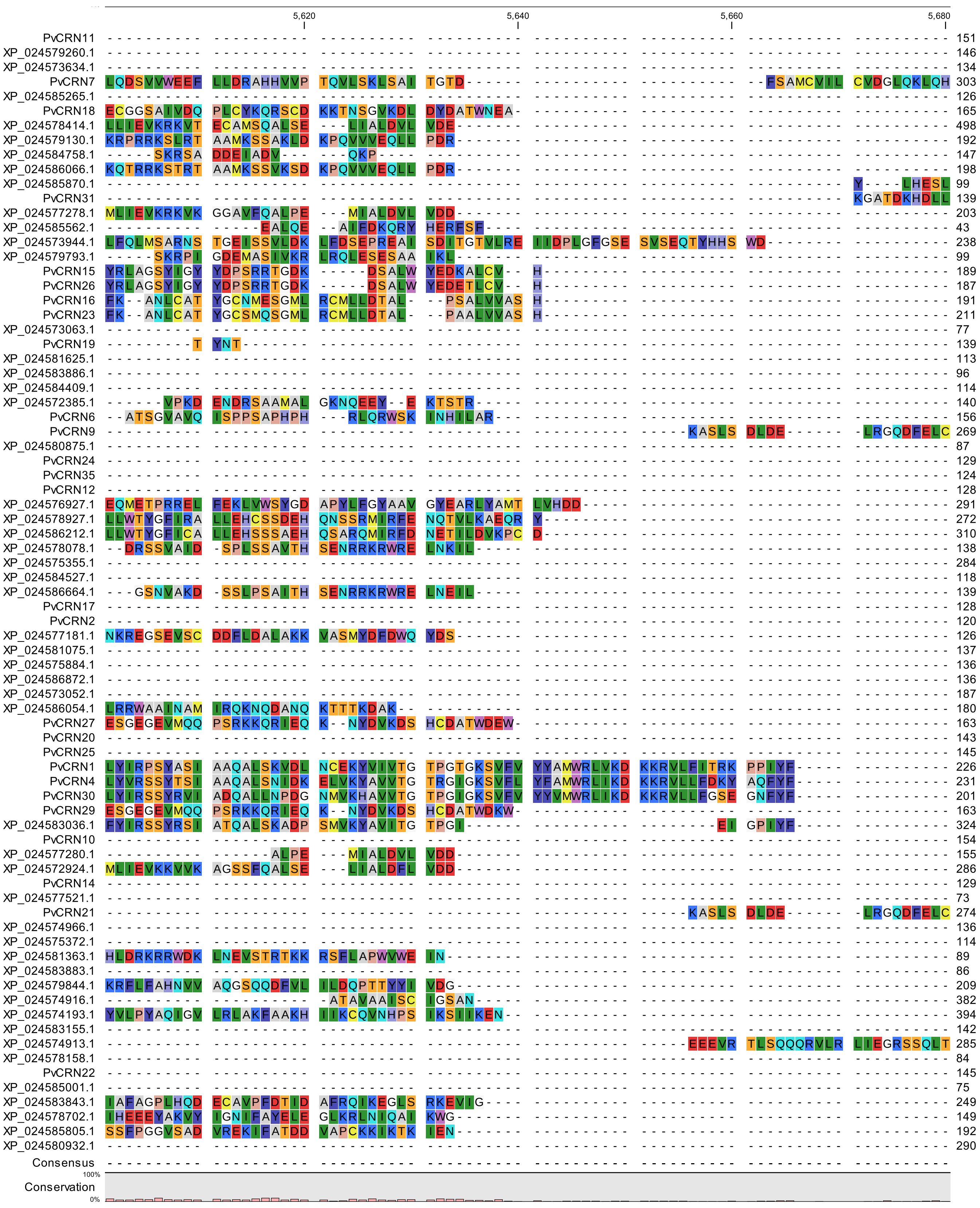

5,620
5,640
5,660
5,680
PvCRN11 151
XP_024579260.1 146
XP_024573634.1 134
PvCRN7 303
XP_024585265.1 126
PvCRN18 165
XP_024578414.1 498
XP_024579130.1 192
XP_024584758.1 147
XP_024586066.1 198
XP_024585870.1 99
PvCRN31 139
XP_024577278.1 203
XP_024585562.1 43
XP_024573944.1 238
XP_024579793.1 99
PvCRN15 189
PvCRN26 187
PvCRN16 191
PvCRN23 211
XP_024573063.1 77
PvCRN19 139
XP_024581625.1 113
XP_024583886.1 96
XP_024584409.1 114
XP_024572385.1 140
PvCRN6 156
PvCRN9 269
XP_024580875.1 87
PvCRN24 129
PvCRN35 124
PvCRN12 128
XP_024576927.1 291
XP_024578927.1 272
XP_024586212.1 310
XP_024578078.1 138
XP_024575355.1 284
XP_024584527.1 118
XP_024586664.1 139
PvCRN17 128
PvCRN2 120
XP_024577181.1 126
XP_024581075.1 137
XP_024575884.1 136
XP_024586872.1 136
XP_024573052.1 187
XP_024586054.1 180
PvCRN27 163
PvCRN20 143
PvCRN25 145
PvCRN1 226
PvCRN4 231
PvCRN30 201
PvCRN29 163
XP_024583036.1 324
PvCRN10 154
XP_024577280.1 155
XP_024572924.1 286
PvCRN14 129
XP_024577521.1 73
PvCRN21 274
XP_024574966.1 136
XP_024575372.1 114
XP_024581363.1 89
XP_024583883.1 86
XP_024579844.1 209
XP_024574916.1 382
XP_024574193.1 394
XP_024583155.1 142
XP_024574913.1 285
XP_024578158.1 84
PvCRN22 145
XP_024585001.1 75
XP_024583843.1 249
XP_024578702.1 149
XP_024585805.1 192
XP_024580932.1 290
Consensus
100%
Conservation
0%

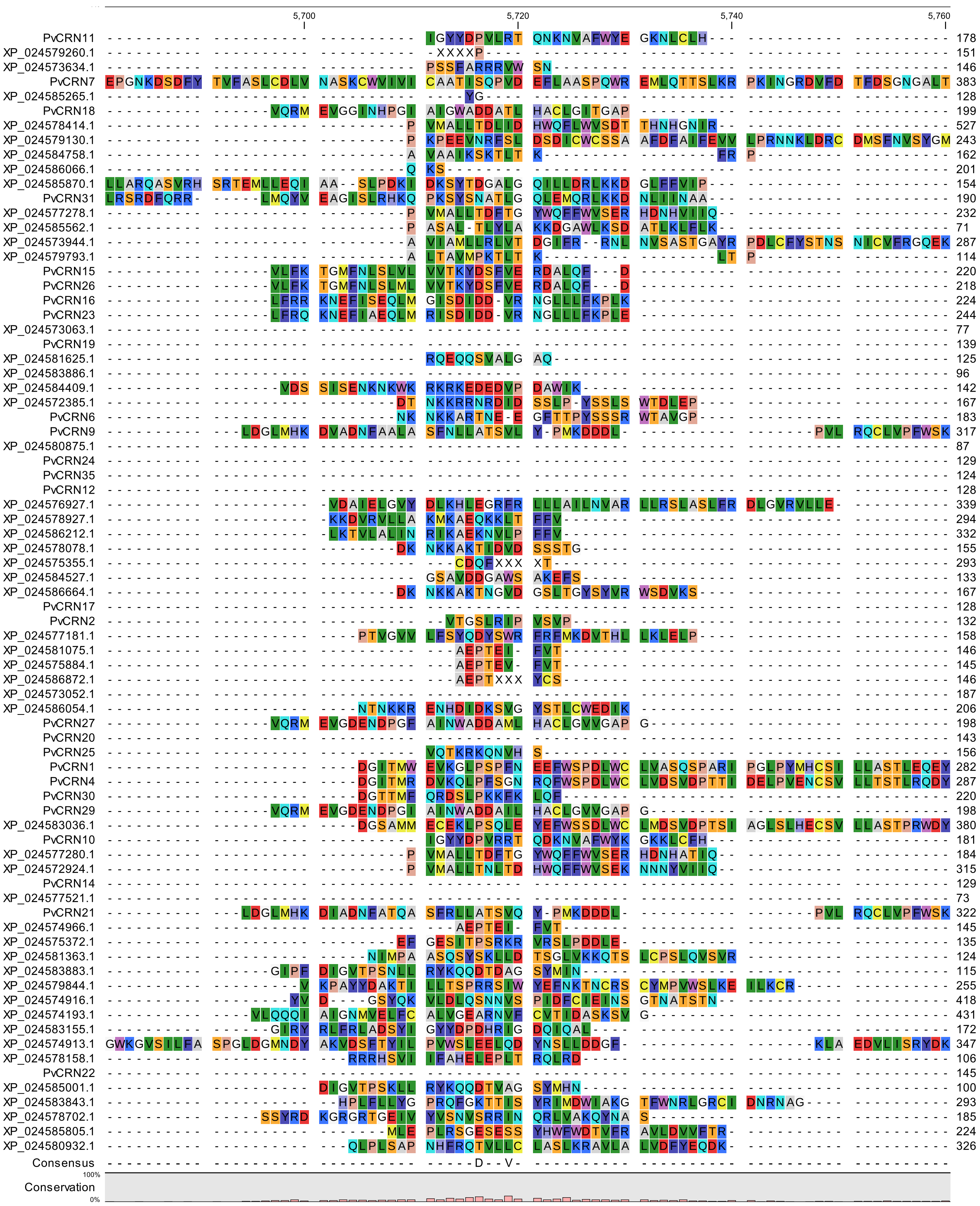
5,700
5,720
5,740
5,760
Consensus
Conservation
100%
0%

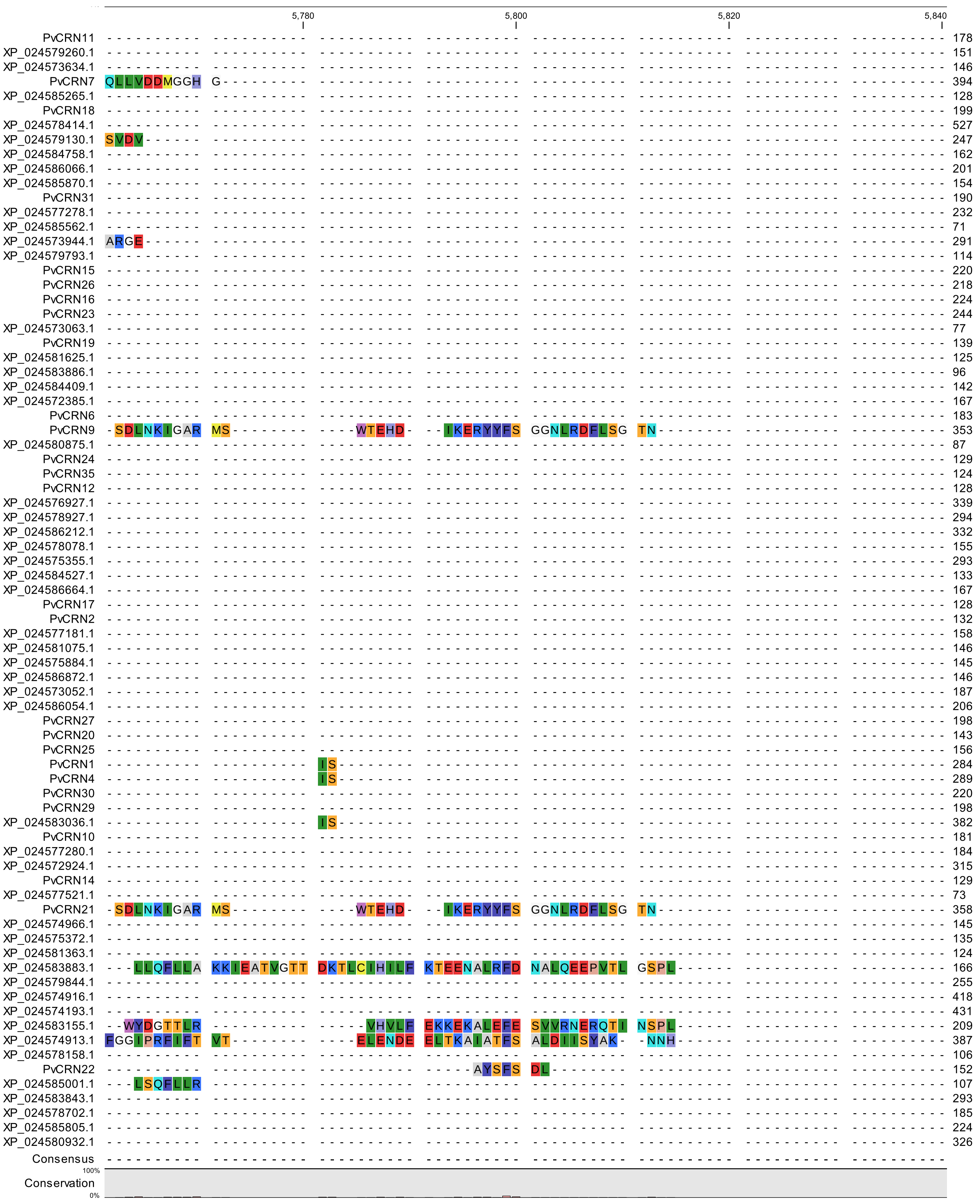
5,780
5,800
5,820
5,840
PvCRN11 178
XP_024579260.1 151
XP_024573634.1 146
PvCRN7 QLLVDDMGGH G 394
XP_024585265.1 128
PvCRN18 199
XP_024578414.1 527
XP_024579130.1 SVDV 247
XP_024584758.1 162
XP_024586066.1 201
XP_024585870.1 154
PvCRN31 190
XP_024577278.1 232
XP_024585562.1 71
XP_024573944.1 ARGE 291
XP_024579793.1 114
PvCRN15 220
PvCRN26 218
PvCRN16 224
PvCRN23 244
XP_024573063.1 77
PvCRN19 139
XP_024581625.1 125
XP_024583886.1 96
XP_024584409.1 142
XP_024572385.1 167
PvCRN6 183
PvCRN9 SDLNKIGAR MS WTEHD IKERYYFS GGNLRDFLSG TN 353
XP_024580875.1 87
PvCRN24 129
PvCRN35 124
PvCRN12 128
XP_024576927.1 339
XP_024578927.1 294
XP_024586212.1 332
XP_024578078.1 155
XP_024575355.1 293
XP_024584527.1 133
XP_024586664.1 167
PvCRN17 128
PvCRN2 132
XP_024577181.1 158
XP_024581075.1 146
XP_024575884.1 145
XP_024586872.1 146
XP_024573052.1 187
XP_024586054.1 206
PvCRN27 198
PvCRN20 143
PvCRN25 156
PvCRN1 IS 284
PvCRN4 IS 289
PvCRN30 220
PvCRN29 198
XP_024583036.1 IS 382
PvCRN10 181
XP_024577280.1 184
XP_024572924.1 315
PvCRN14 129
XP_024577521.1 73
PvCRN21 SDLNKIGAR MS WTEHD IKERYYFS GGNLRDFLSG TN 358
XP_024574966.1 145
XP_024575372.1 135
XP_024581363.1 124
XP_024583883.1 LLQFLLA KKIEATVGTT DKTLCIHILF KTEENALRFD NALQEEPVTL GSPL 166
XP_024579844.1 255
XP_024574916.1 418
XP_024574193.1 431
XP_024583155.1 WYDGTTLR VHVLF EKKEKALEFE SVVRNERQTI NSPL 209
XP_024574913.1 FGGIPRFIFT VT ELENDE ELTKAIATFS ALDIISYAK NNH 387
XP_024578158.1 106
PvCRN22 AYSFS DL 152
XP_024585001.1 LSQFLLR 107
XP_024583843.1 293
XP_024578702.1 185
XP_024585805.1 224
XP_024580932.1 326
Consensus
100%
Conservation
0%

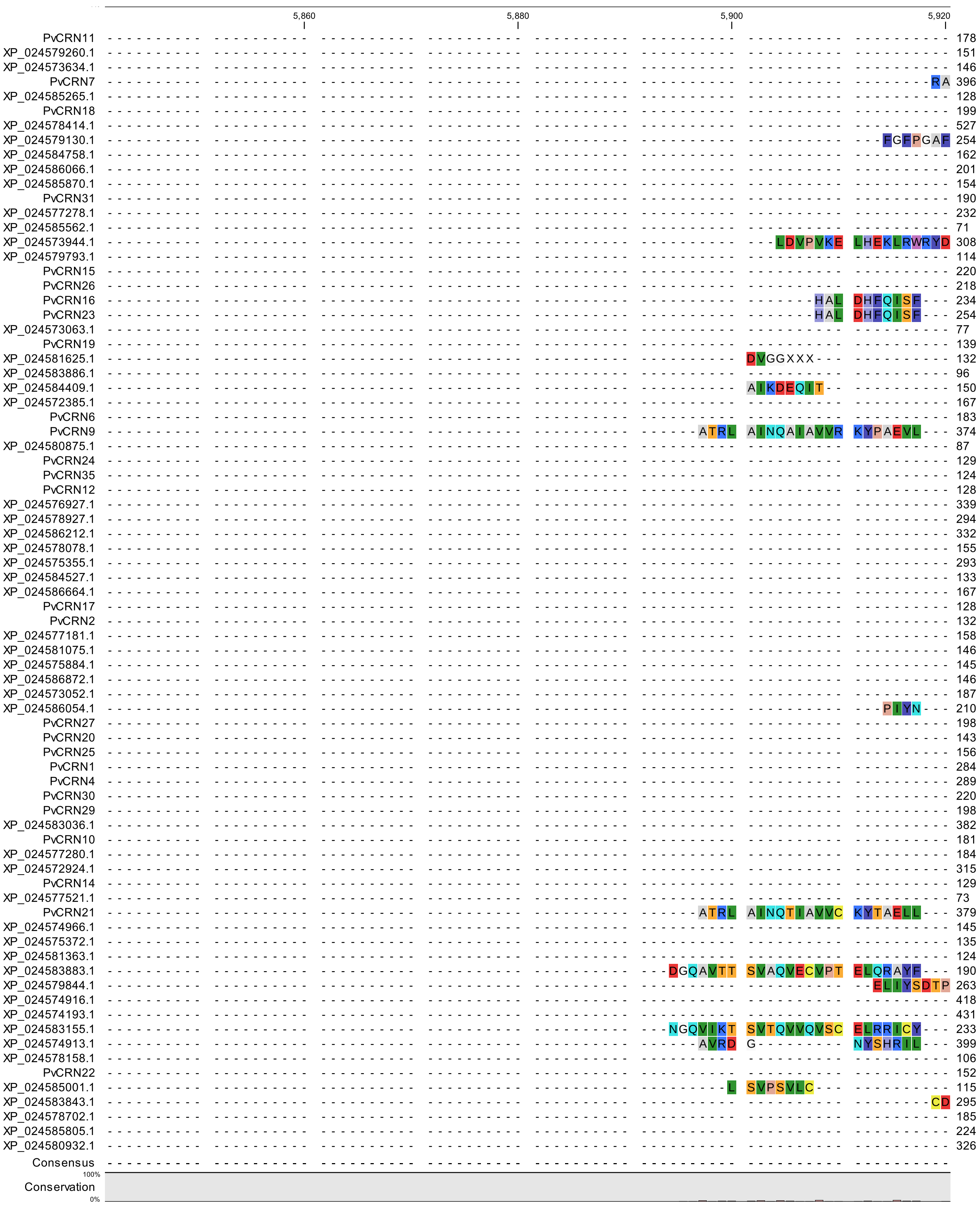
5,860
5,880
5,900
5,920
PvCRN11
XP_024579260.1
XP_024573634.1
PvCRN7
XP_024585265.1
PvCRN18
XP_024578414.1
XP_024579130.1
XP_024584758.1
XP_024586066.1
XP_024585870.1
PvCRN31
XP_024577278.1
XP_024585562.1
XP_024573944.1
XP_024579793.1
PvCRN15
PvCRN26
PvCRN16
PvCRN23
XP_024573063.1
PvCRN19
XP_024581625.1
XP_024583886.1
XP_024584409.1
XP_024572385.1
PvCRN6
PvCRN9
XP_024580875.1
PvCRN24
PvCRN35
PvCRN12
XP_024576927.1
XP_024578927.1
XP_024586212.1
XP_024578078.1
XP_024575355.1
XP_024584527.1
XP_024586664.1
PvCRN17
PvCRN2
XP_024577181.1
XP_024581075.1
XP_024575884.1
XP_024586872.1
XP_024573052.1
XP_024586054.1
PvCRN27
PvCRN20
PvCRN25
PvCRN1
PvCRN4
PvCRN30
PvCRN29
XP_024583036.1
PvCRN10
XP_024577280.1
XP_024572924.1
PvCRN14
XP_024577521.1
PvCRN21
XP_024574966.1
XP_024575372.1
XP_024581363.1
XP_024583883.1
XP_024579844.1
XP_024574916.1
XP_024574193.1
XP_024583155.1
XP_024574913.1
XP_024578158.1
PvCRN22
XP_024585001.1
XP_024583843.1
XP_024578702.1
XP_024585805.1
XP_024580932.1
Consensus
Conservation
100%
0%

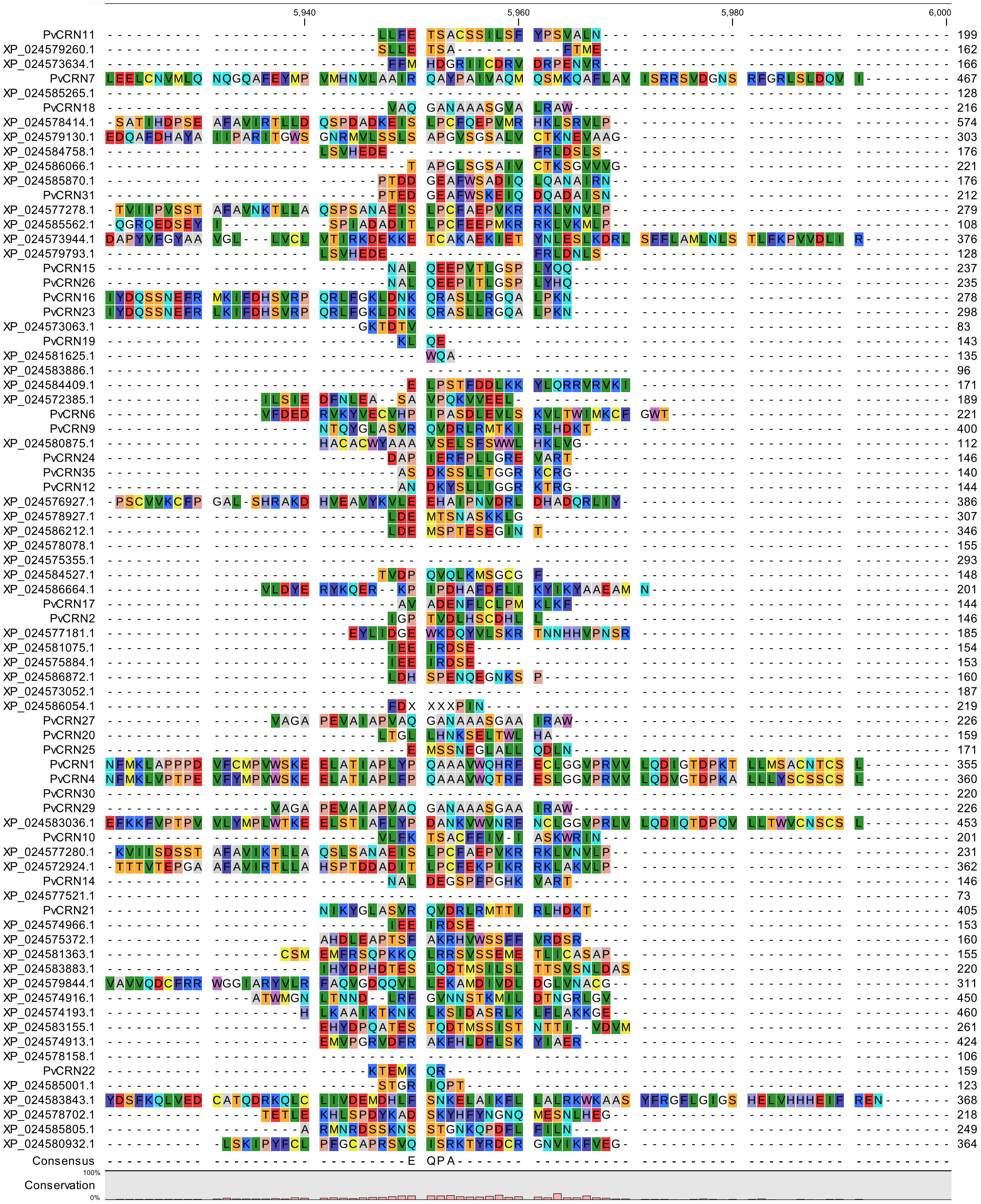
5,940
5,960
5,980
6,000
PvCRN11
XP_024579260.1
XP_024573634.1
PvCRN7
XP_024585265.1
PvCRN18
XP_024578414.1
XP_024579130.1
XP_024584758.1
XP_024586066.1
XP_024585870.1
PvCRN31
XP_024577278.1
XP_024585562.1
XP_024573944.1
XP_024579793.1
PvCRN15
PvCRN26
PvCRN16
PvCRN23
XP_024573063.1
PvCRN19
XP_024581625.1
XP_024583886.1
XP_024584409.1
XP_024572385.1
PvCRN6
PvCRN9
XP_024580875.1
PvCRN24
PvCRN35
PvCRN12
XP_024576927.1
XP_024578927.1
XP_024586212.1
XP_024578078.1
XP_024575355.1
XP_024584527.1
XP_024586664.1
PvCRN17
PvCRN2
XP_024577181.1
XP_024581075.1
XP_024575884.1
XP_024586872.1
XP_024573052.1
XP_024586054.1
PvCRN27
PvCRN20
PvCRN25
PvCRN1
PvCRN4
PvCRN30
PvCRN29
XP_024583036.1
PvCRN10
XP_024577280.1
XP_024572924.1
PvCRN14
XP_024577521.1
PvCRN21
XP_024574966.1
XP_024575372.1
XP_024581363.1
XP_024583883.1
XP_024579844.1
XP_024574916.1
XP_024574193.1
XP_024583155.1
XP_024574913.1
XP_024578158.1
PvCRN22
XP_024585001.1
XP_024583843.1
XP_024578702.1
XP_024585805.1
XP_024580932.1
Consensus
Conservation
100%
0%

| | 6,020 6,040 6,060 6,080 | |
|---|---|---|
| PvCRN11 | ---------- ---------- ---------- ---------- ---------- ---------- ---------- ---------- | 199 |
| XP_024579260.1 | ---------- ---------- ---------- ---------- ---------- ---------- ---------- ---------- | 162 |
| XP_024573634.1 | ---------- ---------- ---------- ---------- ---------- ---------- ---------- ---------- | 166 |
| PvCRN7 | ---------- ---------- ---------- ---------- ---------- ---------- ---------- ---------- | 467 |
| XP_024585265.1 | ---------- ---------- ---------- ---------- ---------- ---------- ---------- ---------- | 128 |
| PvCRN18 | ---------- ---------- ---------- ---------- ---------- ---------- ---------- ---------- | 216 |
| XP_024578414.1 | ---------- ---------- ---------- ---------- ---------- ---------- ---------- ---------- | 574 |
| XP_024579130.1 | ---------- ---------- ---------- ---------- ---------- ---------- ---------- ---------- | 303 |
| XP_024584758.1 | ---------- ---------- ---------- ---------- ---------- ---------- ---------- ---------- | 176 |
| XP_024586066.1 | ---------- ---------- ---------- ---------- ---------- ---------- ---------- ---------- | 221 |
| XP_024585870.1 | ---------- ---------- ---------- ---------- ---------- ---------- ---------- ---------- | 176 |
| PvCRN31 | ---------- ---------- ---------- ---------- ---------- ---------- ---------- ---------- | 212 |
| XP_024577278.1 | ---------- ---------- ---------- ---------- ---------- ---------- ---------- ---------- | 279 |
| XP_024585562.1 | ---------- ---------- ---------- ---------- ---------- ---------- ---------- ---------- | 108 |
| XP_024573944.1 | ---------- ---------- ---------- ---------- ---------- ---------- ---------- ---------- | 376 |
| XP_024579793.1 | ---------- ---------- ---------- ---------- ---------- ---------- ---------- ---------- | 128 |
| PvCRN15 | ---------- ---------- ---------- ---------- ---------- ---------- ---------- ---------- | 237 |
| PvCRN26 | ---------- ---------- ---------- ---------- ---------- ---------- ---------- ---------- | 235 |
| PvCRN16 | ---------- ---------- ---------- ---------- ---------- ---------- ---------- ---------- | 278 |
| PvCRN23 | ---------- ---------- ---------- ---------- ---------- ---------- ---------- ---------- | 298 |
| XP_024573063.1 | ---------- ---------- ---------- ---------- ---------- ---------- ---------- ---------- | 83 |
| PvCRN19 | ---------- ---------- ---------- ---------- ---------- ---------- ---------- ---------- | 143 |
| XP_024581625.1 | ---------- ---------- ---------- ---------- ---------- ---------- ---------- ---------- | 135 |
| XP_024583886.1 | ---------- ---------- ---------- ---------- ---------- ---------- ---------- ---------- | 96 |
| XP_024584409.1 | ---------- ---------- ---------- ---------- ---------- ---------- ---------- ---------- | 171 |
| XP_024572385.1 | ---------- ---------- ---------- ---------- ---------- ---------- ---------- ---------- | 189 |
| PvCRN6 | ---------- ---------- ---------- ---------- ---------- ---------- ---------- ---------- | 221 |
| PvCRN9 | ---------- ---------- ---------- ---------- ---------- ---------- ---------- ---------- | 400 |
| XP_024580875.1 | ---------- ---------- ---------- ---------- ---------- ---------- ---------- ---------- | 112 |
| PvCRN24 | ---------- ---------- ---------- ---------- ---------- ---------- ---------- ---------- | 146 |
| PvCRN35 | ---------- ---------- ---------- ---------- ---------- ---------- ---------- ---------- | 140 |
| PvCRN12 | ---------- ---------- ---------- ---------- ---------- ---------- ---------- ---------- | 144 |
| XP_024576927.1 | ---------- ---------- ---------- ---------- ---------- ---------- ---------- ---------- | 386 |
| XP_024578927.1 | ---------- ---------- ---------- ---------- ---------- ---------- ---------- ---------- | 307 |
| XP_024586212.1 | ---------- ---------- ---------- ---------- ---------- ---------- ---------- ---------- | 346 |
| XP_024578078.1 | ---------- ---------- ---------- ---------- ---------- ---------- ---------- ---------- | 155 |
| XP_024575355.1 | ---------- ---------- ---------- ---------- ---------- ---------- ---------- ---------- | 293 |
| XP_024584527.1 | ---------- ---------- ---------- ---------- ---------- ---------- ---------- ---------- | 148 |
| XP_024586664.1 | ---------- ---------- ---------- ---------- ---------- ---------- ---------- ---------- | 201 |
| PvCRN17 | ---------- ---------- ---------- ---------- ---------- ---------- ---------- ---------- | 144 |
| PvCRN2 | ---------- ---------- ---------- ---------- ---------- ---------- ---------- ---------- | 146 |
| XP_024577181.1 | ---------- ---------- ---------- ---------- ---------- ---------- ---------- ---------- | 185 |
| XP_024581075.1 | ---------- ---------- ---------- ---------- ---------- ---------- ---------- ---------- | 154 |
| XP_024575884.1 | ---------- ---------- ---------- ---------- ---------- ---------- ---------- ---------- | 153 |
| XP_024586872.1 | ---------- ---------- ---------- ---------- ---------- ---------- ---------- ---------- | 160 |
| XP_024573052.1 | ---------- ---------- ---------- ---------- ---------- ---------- ---------- ---------- | 187 |
| XP_024586054.1 | ---------- ---------- ---------- ---------- ---------- ---------- ---------- ---------- | 219 |
| PvCRN27 | ---------- ---------- ---------- ---------- ---------- ---------- ---------- ---------- | 226 |
| PvCRN20 | ---------- ---------- ---------- ---------- ---------- ---------- ---------- ---------- | 159 |
| PvCRN25 | ---------- ---------- ---------- ---------- ---------- ---------- ---------- ---------- | 171 |
| PvCRN1 | ---------- ---------- ---------- ---------- ---------- ---------- ---------- ---------- | 355 |
| PvCRN4 | ---------- ---------- ---------- ---------- ---------- ---------- ---------- ---------- | 360 |
| PvCRN30 | ---------- ---------- ---------- ---------- ---------- ---------- ---------- ---------- | 220 |
| PvCRN29 | ---------- ---------- ---------- ---------- ---------- ---------- ---------- ---------- | 226 |
| XP_024583036.1 | ---------- ---------- ---------- ---------- ---------- ---------- ---------- ---------- | 453 |
| PvCRN10 | ---------- ---------- ---------- ---------- ---------- ---------- ---------- ---------- | 201 |
| XP_024577280.1 | ---------- ---------- ---------- ---------- ---------- ---------- ---------- ---------- | 231 |
| XP_024572924.1 | ---------- ---------- ---------- ---------- ---------- ---------- ---------- ---------- | 362 |
| PvCRN14 | ---------- ---------- ---------- ---------- ---------- ---------- ---------- ---------- | 146 |
| XP_024577521.1 | ---------- ---------- ---------- ---------- ---------- ---------- ---------- ---------- | 73 |
| PvCRN21 | ---------- ---------- ---------- ---------- ---------- ---------- ---------- ---------- | 405 |
| XP_024574966.1 | ---------- ---------- ---------- ---------- ---------- ---------- ---------- ---------- | 153 |
| XP_024575372.1 | ---------- ---------- ---------- ---------- ---------- ---------- ---------- ---------- | 160 |
| XP_024581363.1 | ---------- ---------- ---------- ---------- ---------- ---------- ---------- ---------- | 155 |
| XP_024583883.1 | ---------- ---------- ---------- ---------- ---------- ---------- ---------- ---------- | 220 |
| XP_024579844.1 | ---------- ---------- ---------- ---------- ---------- ---------- ---------- ---------- | 311 |
| XP_024574916.1 | ---------- ---------- ---------- ---------- ---------- ---------- ---------- ---------- | 450 |
| XP_024574193.1 | ---------- ---------- ---------- ---------- ---------- ---------- ---------- ---------- | 460 |
| XP_024583155.1 | ---------- ---------- ---------- ---------- ---------- ---------- ---------- ---------- | 261 |
| XP_024574913.1 | ---------- ---------- ---------- ---------- ---------- ---------- ---------- ---------- | 424 |
| XP_024578158.1 | ---------- ---------- ---------- ---------- ---------- ---------- ---------- ---------- | 106 |
| PvCRN22 | ---------- ---------- ---------- ---------- ---------- ---------- ---------- ---------- | 159 |
| XP_024585001.1 | ---------- ---------- ---------- ---------- ---------- ---------- ---------- ---------- | 123 |
| XP_024583843.1 | ---------- ---------- ---------- ---------- ---------- ---------- ---------- ---------- | 368 |
| XP_024578702.1 | ---------- ---------- ---------- ---------- ---------- ---------- ---------- ---------- | 218 |
| XP_024585805.1 | ---------- ---------- ---------- ---------- ---------- ---------- ---------- ---------- | 249 |
| XP_024580932.1 | ---------- ---------- ---------- ---------- ---------- ---------- ---------- ---------- | 364 |
| Consensus | ---------- ---------- ---------- ---------- ---------- ---------- ---------- ---------- | |
| Conservation (100% – 0%) | | |

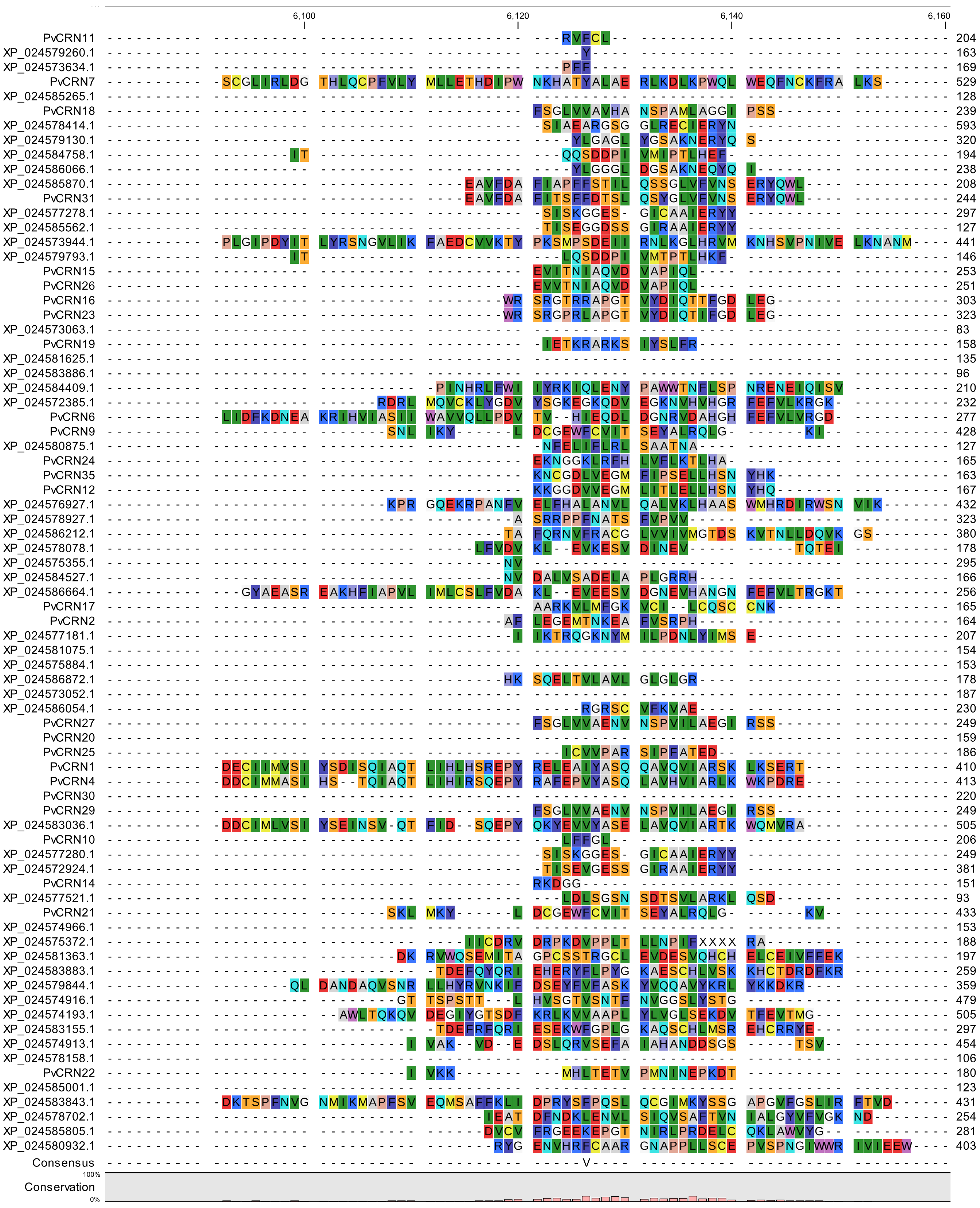
6,100
6,120
6,140
6,160
Consensus
Conservation
100%
0%

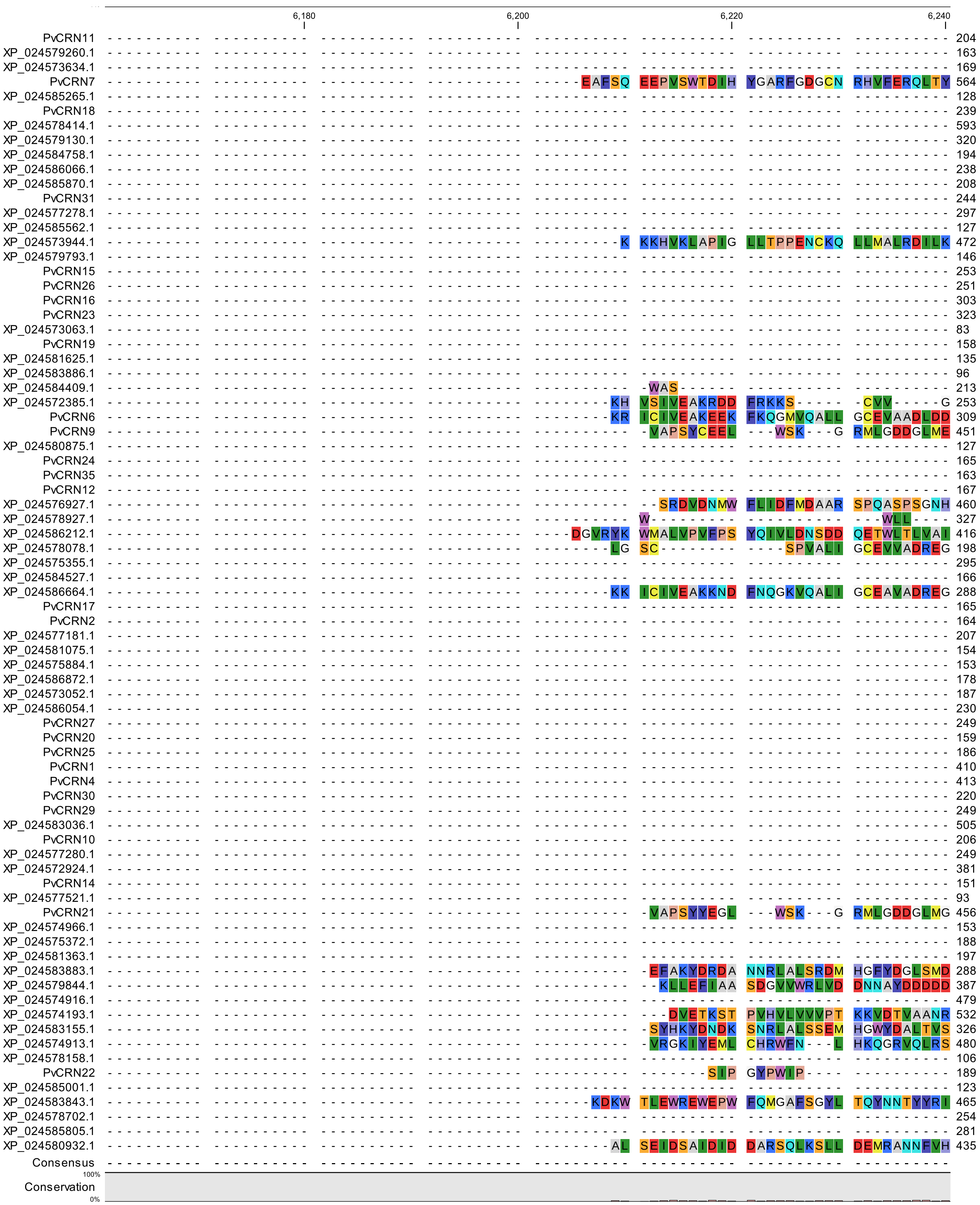
6,180
6,200
6,220
6,240
PvCRN11 204
XP_024579260.1 163
XP_024573634.1 169
PvCRN7 EAFSQ EEPVSWTDIH YGARFGDGCN RHVFERQLTY 564
XP_024585265.1 128
PvCRN18 239
XP_024578414.1 593
XP_024579130.1 320
XP_024584758.1 194
XP_024586066.1 238
XP_024585870.1 208
PvCRN31 244
XP_024577278.1 297
XP_024585562.1 127
XP_024573944.1 K KKHVKLAPIG LLTPPENCKQ LLMALRDILK 472
XP_024579793.1 146
PvCRN15 253
PvCRN26 251
PvCRN16 303
PvCRN23 323
XP_024573063.1 83
PvCRN19 158
XP_024581625.1 135
XP_024583886.1 96
XP_024584409.1 -WAS 213
XP_024572385.1 KH VSIVEAKRDD FRKKS- - - - - -CVV- - - - -G 253
PvCRN6 KR ICIVEAKEEK FKQGMVQALL GCEVAADLDD 309
PvCRN9 -VAPSYCEEL - - -WSK- - -G RMLGDDGLME 451
XP_024580875.1 127
PvCRN24 165
PvCRN35 163
PvCRN12 167
XP_024576927.1 - -SRDVDNMW FLIDFMDAAR SPQASPSGNH 460
XP_024578927.1 W - - -WLL 327
XP_024586212.1 DGVRYK WMALVPVFPS YQIVLDNSDD QETWLTLVAI 416
XP_024578078.1 LG SC - - - -SPVALI GCEVVADREG 198
XP_024575355.1 295
XP_024584527.1 166
XP_024586664.1 KK ICIVEAKKND FNQGKVQALI GCEAVADREG 288
PvCRN17 165
PvCRN2 164
XP_024577181.1 207
XP_024581075.1 154
XP_024575884.1 153
XP_024586872.1 178
XP_024573052.1 187
XP_024586054.1 230
PvCRN27 249
PvCRN20 159
PvCRN25 186
PvCRN1 410
PvCRN4 413
PvCRN30 220
PvCRN29 249
XP_024583036.1 505
PvCRN10 206
XP_024577280.1 249
XP_024572924.1 381
PvCRN14 151
XP_024577521.1 93
PvCRN21 -VAPSYYEGL - - -WSK- - -G RMLGDDGLMG 456
XP_024574966.1 153
XP_024575372.1 188
XP_024581363.1 197
XP_024583883.1 -EFAKYDRDA NNRLALSRDM HGFYDGLSMD 288
XP_024579844.1 - -KLLEFIAA SDGVVWRLVD DNNAYDDDDD 387
XP_024574916.1 479
XP_024574193.1 - - -DVETKST PVHVLVVVPT KKVDTVAANR 532
XP_024583155.1 -SYHKYDNDK SNRLALSSEM HGWYDALTVS 326
XP_024574913.1 -VRGKIYEML CHRWFN- - -L HKQGRVQLRS 480
XP_024578158.1 106
PvCRN22 SIP GYPWIP 189
XP_024585001.1 123
XP_024583843.1 KDKW TLEWREWEPW FQMGAFSGYL TQYNNTYYRI 465
XP_024578702.1 254
XP_024585805.1 281
XP_024580932.1 AL SEIDSAIDID DARSQLKSLL DEMRANNFVH 435
Consensus
100%
Conservation
0%

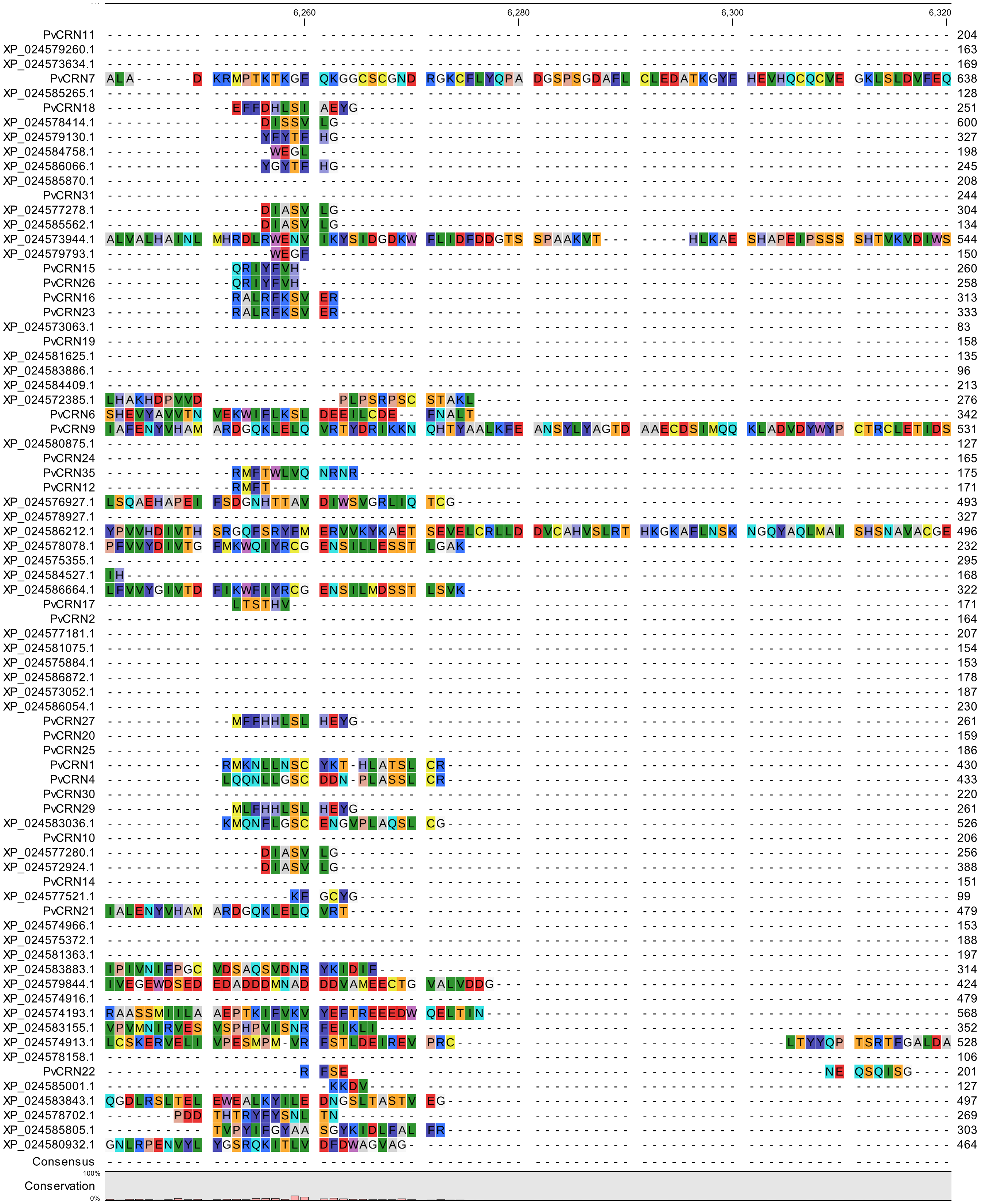
6,260
6,280
6,300
6,320
PvCRN11 204
XP_024579260.1 163
XP_024573634.1 169
PvCRN7 638
XP_024585265.1 128
PvCRN18 251
XP_024578414.1 600
XP_024579130.1 327
XP_024584758.1 198
XP_024586066.1 245
XP_024585870.1 208
PvCRN31 244
XP_024577278.1 304
XP_024585562.1 134
XP_024573944.1 544
XP_024579793.1 150
PvCRN15 260
PvCRN26 258
PvCRN16 313
PvCRN23 333
XP_024573063.1 83
PvCRN19 158
XP_024581625.1 135
XP_024583886.1 96
XP_024584409.1 213
XP_024572385.1 276
PvCRN6 342
PvCRN9 531
XP_024580875.1 127
PvCRN24 165
PvCRN35 175
PvCRN12 171
XP_024576927.1 493
XP_024578927.1 327
XP_024586212.1 496
XP_024578078.1 232
XP_024575355.1 295
XP_024584527.1 168
XP_024586664.1 322
PvCRN17 171
PvCRN2 164
XP_024577181.1 207
XP_024581075.1 154
XP_024575884.1 153
XP_024586872.1 178
XP_024573052.1 187
XP_024586054.1 230
PvCRN27 261
PvCRN20 159
PvCRN25 186
PvCRN1 430
PvCRN4 433
PvCRN30 220
PvCRN29 261
XP_024583036.1 526
PvCRN10 206
XP_024577280.1 256
XP_024572924.1 388
PvCRN14 151
XP_024577521.1 99
PvCRN21 479
XP_024574966.1 153
XP_024575372.1 188
XP_024581363.1 197
XP_024583883.1 314
XP_024579844.1 424
XP_024574916.1 479
XP_024574193.1 568
XP_024583155.1 352
XP_024574913.1 528
XP_024578158.1 106
PvCRN22 201
XP_024585001.1 127
XP_024583843.1 497
XP_024578702.1 269
XP_024585805.1 303
XP_024580932.1 464
Consensus
Conservation
100%
0%

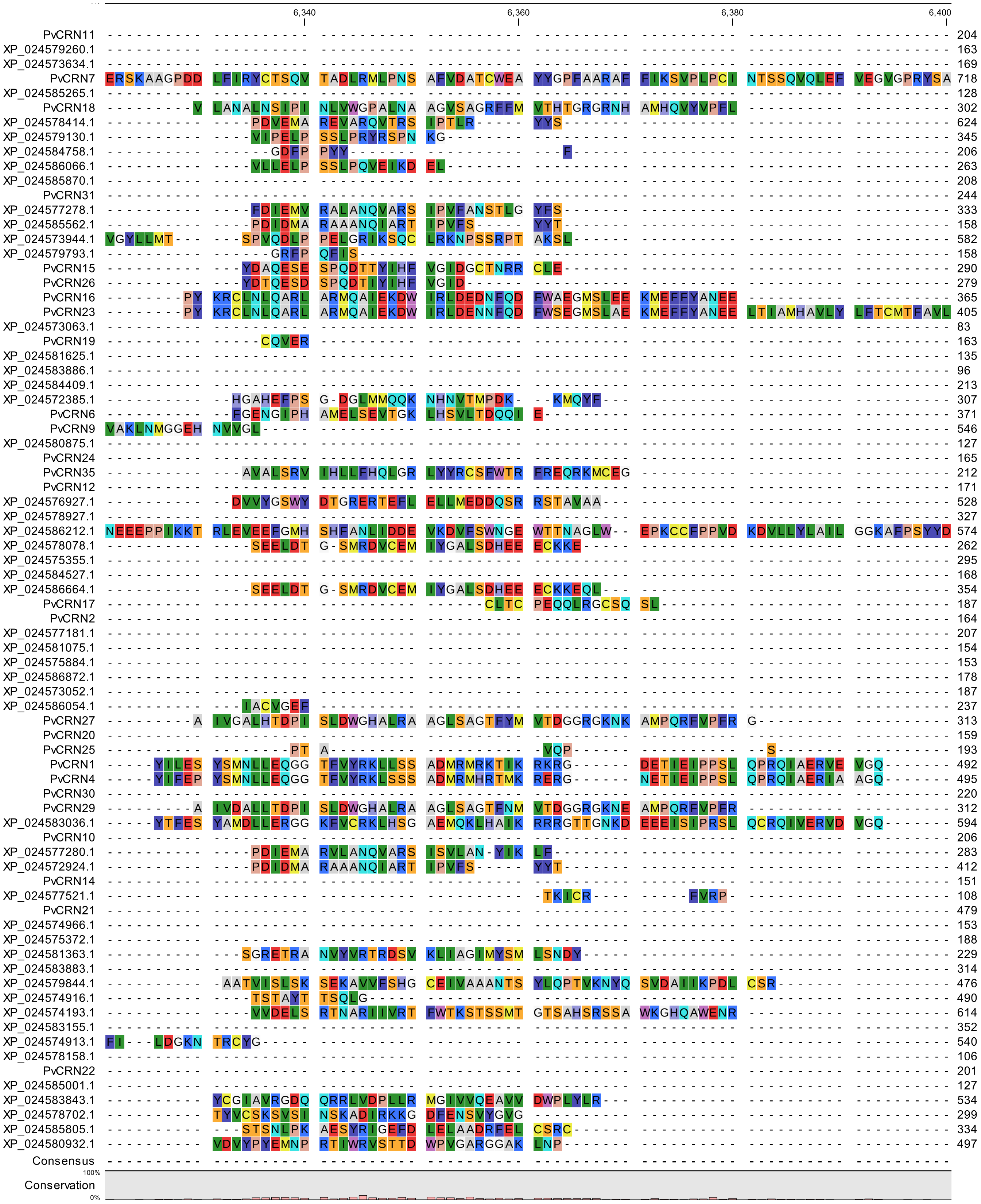
6,340
6,360
6,380
6,400
PvCRN11 204
XP_024579260.1 163
XP_024573634.1 169
PvCRN7 718
XP_024585265.1 128
PvCRN18 302
XP_024578414.1 624
XP_024579130.1 345
XP_024584758.1 206
XP_024586066.1 263
XP_024585870.1 208
PvCRN31 244
XP_024577278.1 333
XP_024585562.1 158
XP_024573944.1 582
XP_024579793.1 158
PvCRN15 290
PvCRN26 279
PvCRN16 365
PvCRN23 405
XP_024573063.1 83
PvCRN19 163
XP_024581625.1 135
XP_024583886.1 96
XP_024584409.1 213
XP_024572385.1 307
PvCRN6 371
PvCRN9 546
XP_024580875.1 127
PvCRN24 165
PvCRN35 212
PvCRN12 171
XP_024576927.1 528
XP_024578927.1 327
XP_024586212.1 574
XP_024578078.1 262
XP_024575355.1 295
XP_024584527.1 168
XP_024586664.1 354
PvCRN17 187
PvCRN2 164
XP_024577181.1 207
XP_024581075.1 154
XP_024575884.1 153
XP_024586872.1 178
XP_024573052.1 187
XP_024586054.1 237
PvCRN27 313
PvCRN20 159
PvCRN25 193
PvCRN1 492
PvCRN4 495
PvCRN30 220
PvCRN29 312
XP_024583036.1 594
PvCRN10 206
XP_024577280.1 283
XP_024572924.1 412
PvCRN14 151
XP_024577521.1 108
PvCRN21 479
XP_024574966.1 153
XP_024575372.1 188
XP_024581363.1 229
XP_024583883.1 314
XP_024579844.1 476
XP_024574916.1 490
XP_024574193.1 614
XP_024583155.1 352
XP_024574913.1 540
XP_024578158.1 106
PvCRN22 201
XP_024585001.1 127
XP_024583843.1 534
XP_024578702.1 299
XP_024585805.1 334
XP_024580932.1 497
Consensus
Conservation
100%
0%

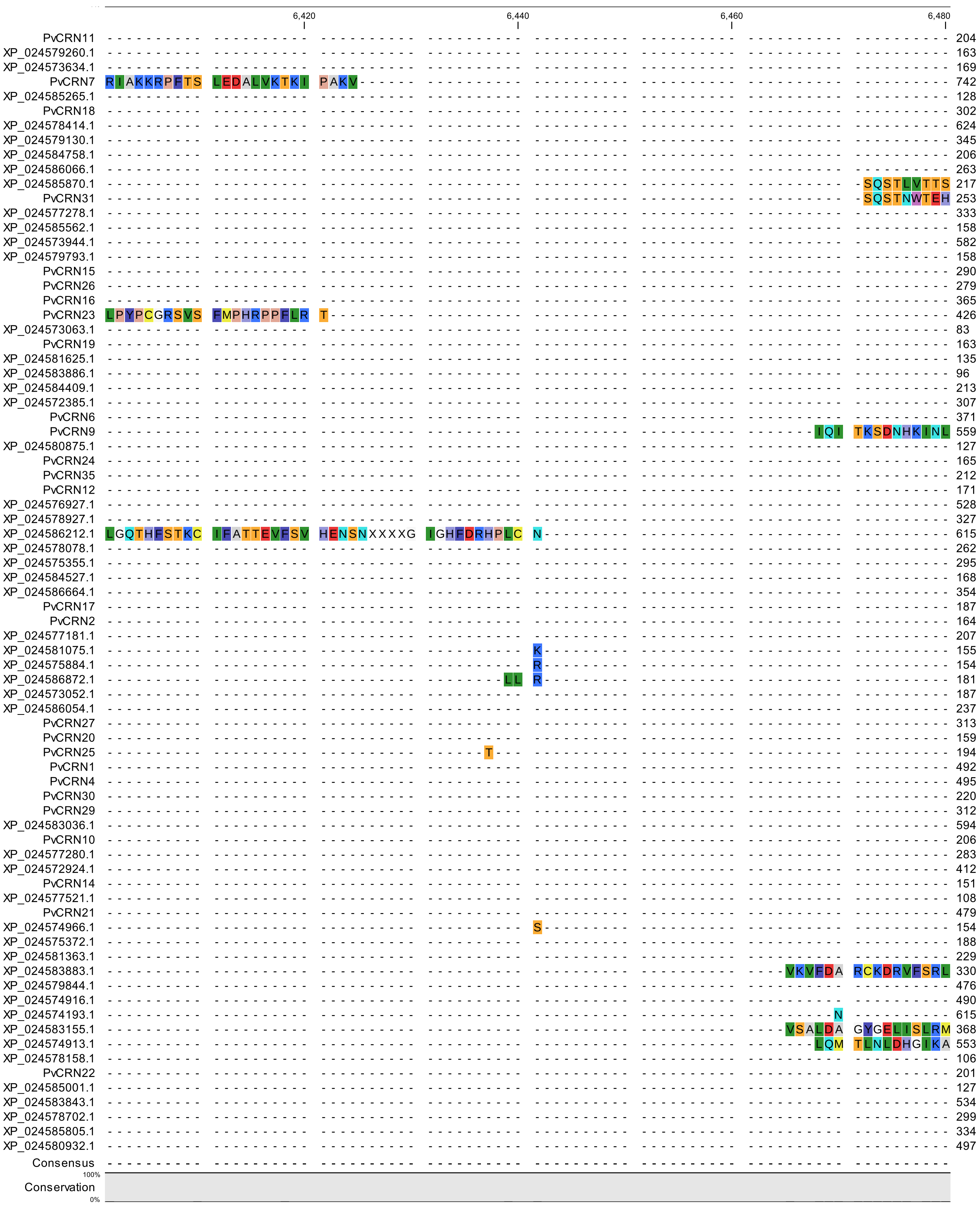
6,420
6,440
6,460
6,480
PvCRN11 204
XP_024579260.1 163
XP_024573634.1 169
PvCRN7 RIAKKRPFTS LEDALVKTKI PAKV 742
XP_024585265.1 128
PvCRN18 302
XP_024578414.1 624
XP_024579130.1 345
XP_024584758.1 206
XP_024586066.1 263
XP_024585870.1 SQSTLVTTS 217
PvCRN31 SQSTNWTEH 253
XP_024577278.1 333
XP_024585562.1 158
XP_024573944.1 582
XP_024579793.1 158
PvCRN15 290
PvCRN26 279
PvCRN16 365
PvCRN23 LPYPCGRSVS FMPHRPPFLR T 426
XP_024573063.1 83
PvCRN19 163
XP_024581625.1 135
XP_024583886.1 96
XP_024584409.1 213
XP_024572385.1 307
PvCRN6 371
PvCRN9 IQI TKSDNHKINL 559
XP_024580875.1 127
PvCRN24 165
PvCRN35 212
PvCRN12 171
XP_024576927.1 528
XP_024578927.1 327
XP_024586212.1 LGQTHFSTKC IFATTEVFSV HENSNXXXXG IGHFDRHPLC N 615
XP_024578078.1 262
XP_024575355.1 295
XP_024584527.1 168
XP_024586664.1 354
PvCRN17 187
PvCRN2 164
XP_024577181.1 207
XP_024581075.1 K 155
XP_024575884.1 R 154
XP_024586872.1 LL R 181
XP_024573052.1 187
XP_024586054.1 237
PvCRN27 313
PvCRN20 159
PvCRN25 T 194
PvCRN1 492
PvCRN4 495
PvCRN30 220
PvCRN29 312
XP_024583036.1 594
PvCRN10 206
XP_024577280.1 283
XP_024572924.1 412
PvCRN14 151
XP_024577521.1 108
PvCRN21 479
XP_024574966.1 S 154
XP_024575372.1 188
XP_024581363.1 229
XP_024583883.1 VKVFDA RCKDRVFSRL 330
XP_024579844.1 476
XP_024574916.1 490
XP_024574193.1 N 615
XP_024583155.1 VSALDA GYGELISLRM 368
XP_024574913.1 LQM TLNLDHGIKA 553
XP_024578158.1 106
PvCRN22 201
XP_024585001.1 127
XP_024583843.1 534
XP_024578702.1 299
XP_024585805.1 334
XP_024580932.1 497
Consensus
100%
Conservation
0%

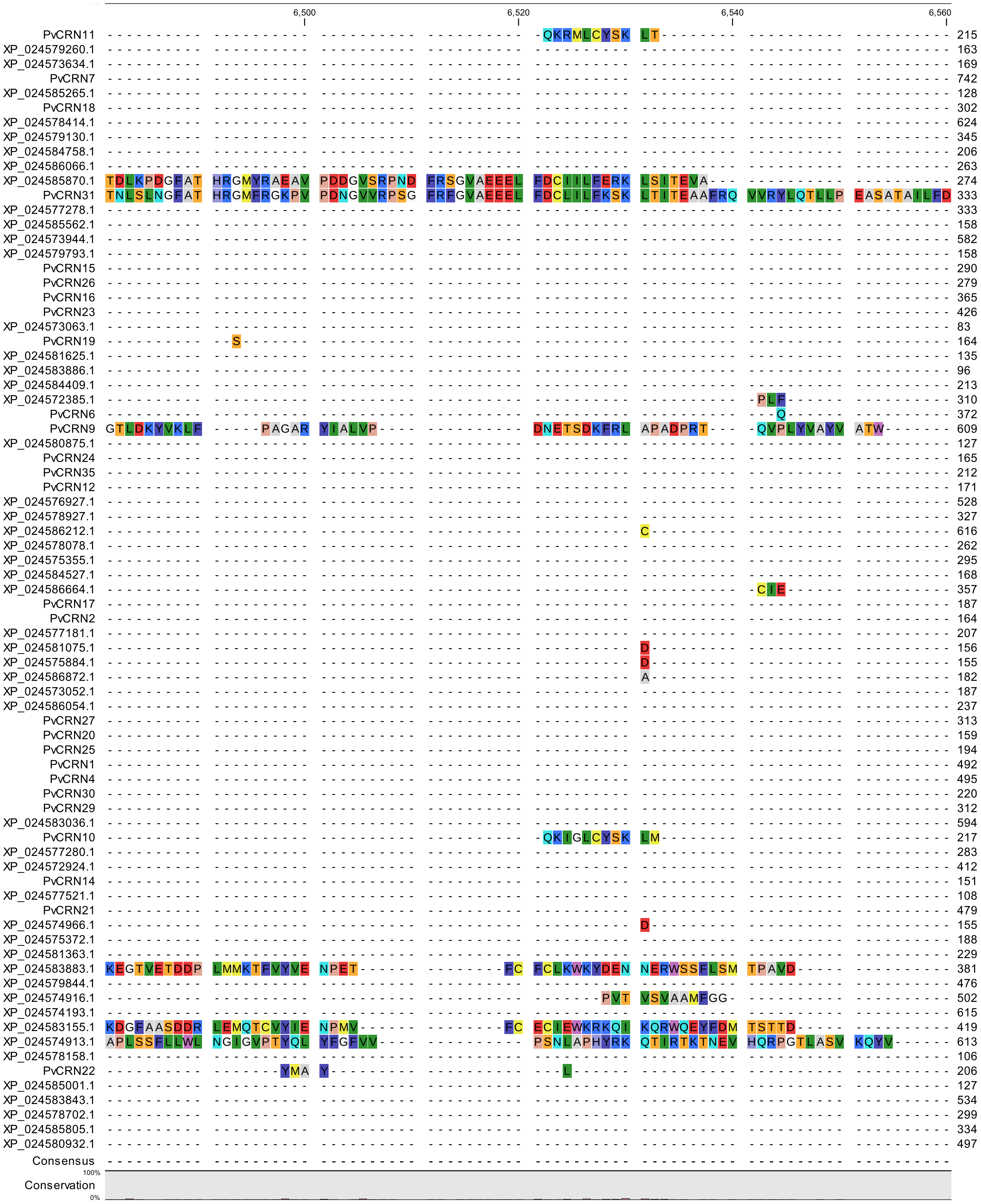
6,500
6,520
6,540
6,560
PvCRN11 QKRMLCYSK LT 215
XP_024579260.1 163
XP_024573634.1 169
PvCRN7 742
XP_024585265.1 128
PvCRN18 302
XP_024578414.1 624
XP_024579130.1 345
XP_024584758.1 206
XP_024586066.1 263
XP_024585870.1 TDLKPDGFAT HRGMYRAEAV PDDGVSRPND FRSGVAEEEL FDCIILFERK LSITEVA 274
PvCRN31 TNLSLNGFAT HRGMFRGKPV PDNGVVRPSG FRFGVAEEEL FDCLILFKSK LTITEAAFRQ VVRYLQTLLP EASATAILFD 333
XP_024577278.1 333
XP_024585562.1 158
XP_024573944.1 582
XP_024579793.1 158
PvCRN15 290
PvCRN26 279
PvCRN16 365
PvCRN23 426
XP_024573063.1 83
PvCRN19 S 164
XP_024581625.1 135
XP_024583886.1 96
XP_024584409.1 213
XP_024572385.1 PLF 310
PvCRN6 Q 372
PvCRN9 GTLDKYVKLF PAGAR YIALVP DNETSDKFRL APADPRT QVPLYVAYV ATW 609
XP_024580875.1 127
PvCRN24 165
PvCRN35 212
PvCRN12 171
XP_024576927.1 528
XP_024578927.1 327
XP_024586212.1 C 616
XP_024578078.1 262
XP_024575355.1 295
XP_024584527.1 168
XP_024586664.1 CIE 357
PvCRN17 187
PvCRN2 164
XP_024577181.1 207
XP_024581075.1 D 156
XP_024575884.1 D 155
XP_024586872.1 A 182
XP_024573052.1 187
XP_024586054.1 237
PvCRN27 313
PvCRN20 159
PvCRN25 194
PvCRN1 492
PvCRN4 495
PvCRN30 220
PvCRN29 312
XP_024583036.1 594
PvCRN10 QKIGLCYSK LM 217
XP_024577280.1 283
XP_024572924.1 412
PvCRN14 151
XP_024577521.1 108
PvCRN21 479
XP_024574966.1 D 155
XP_024575372.1 188
XP_024581363.1 229
XP_024583883.1 KEGTVETDDP LMMKTFVYVE NPET FC FCLKWKYDEN NERWSSFLSM TPAVD 381
XP_024579844.1 476
XP_024574916.1 PVT VSVAAMFGG 502
XP_024574193.1 615
XP_024583155.1 KDGFAASDDR LEMQTCVYIE NPMV FC ECIEWKRKQI KQRWQEYFDM TSTTD 419
XP_024574913.1 APLSSFLLWL NGIGVPTYQL YFGFVV PSNLAPHYRK QTIRTKTNEV HQRPGTLASV KQYV 613
XP_024578158.1 106
PvCRN22 YMA Y L 206
XP_024585001.1 127
XP_024583843.1 534
XP_024578702.1 299
XP_024585805.1 334
XP_024580932.1 497
Consensus
Conservation
100%
0%

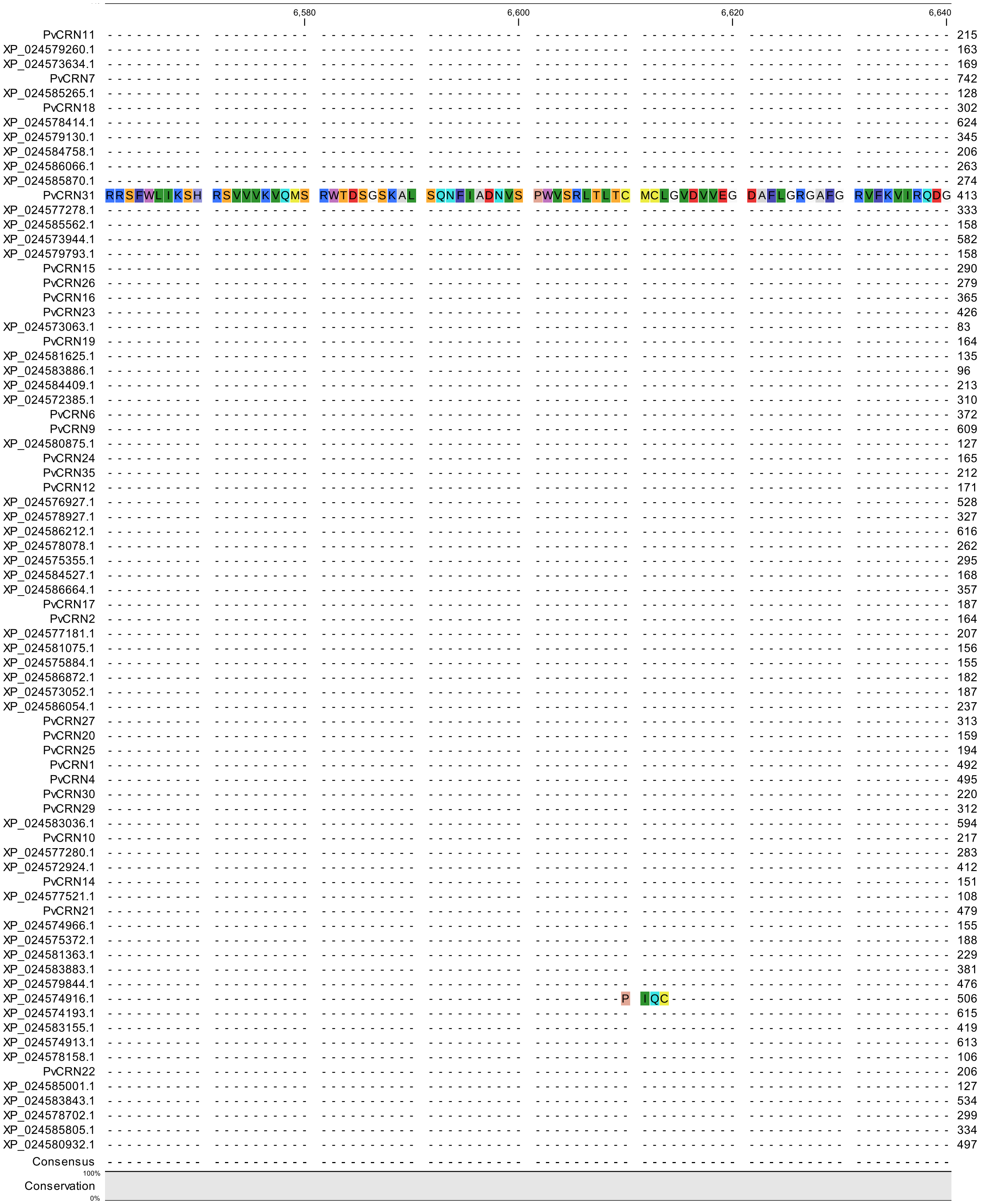
6,580
6,600
6,620
6,640
PvCRN11 215
XP_024579260.1 163
XP_024573634.1 169
PvCRN7 742
XP_024585265.1 128
PvCRN18 302
XP_024578414.1 624
XP_024579130.1 345
XP_024584758.1 206
XP_024586066.1 263
XP_024585870.1 274
PvCRN31 RRSFWLIKSH RSVVVKVQMS RWTDSGSKAL SQNFIADNVS PWVSRLTLTC MCLGVDVVEG DAFLGRGAFG RVFKVIRQDG 413
XP_024577278.1 333
XP_024585562.1 158
XP_024573944.1 582
XP_024579793.1 158
PvCRN15 290
PvCRN26 279
PvCRN16 365
PvCRN23 426
XP_024573063.1 83
PvCRN19 164
XP_024581625.1 135
XP_024583886.1 96
XP_024584409.1 213
XP_024572385.1 310
PvCRN6 372
PvCRN9 609
XP_024580875.1 127
PvCRN24 165
PvCRN35 212
PvCRN12 171
XP_024576927.1 528
XP_024578927.1 327
XP_024586212.1 616
XP_024578078.1 262
XP_024575355.1 295
XP_024584527.1 168
XP_024586664.1 357
PvCRN17 187
PvCRN2 164
XP_024577181.1 207
XP_024581075.1 156
XP_024575884.1 155
XP_024586872.1 182
XP_024573052.1 187
XP_024586054.1 237
PvCRN27 313
PvCRN20 159
PvCRN25 194
PvCRN1 492
PvCRN4 495
PvCRN30 220
PvCRN29 312
XP_024583036.1 594
PvCRN10 217
XP_024577280.1 283
XP_024572924.1 412
PvCRN14 151
XP_024577521.1 108
PvCRN21 479
XP_024574966.1 155
XP_024575372.1 188
XP_024581363.1 229
XP_024583883.1 381
XP_024579844.1 476
XP_024574916.1 P IQC 506
XP_024574193.1 615
XP_024583155.1 419
XP_024574913.1 613
XP_024578158.1 106
PvCRN22 206
XP_024585001.1 127
XP_024583843.1 534
XP_024578702.1 299
XP_024585805.1 334
XP_024580932.1 497
Consensus
100%
Conservation
0%

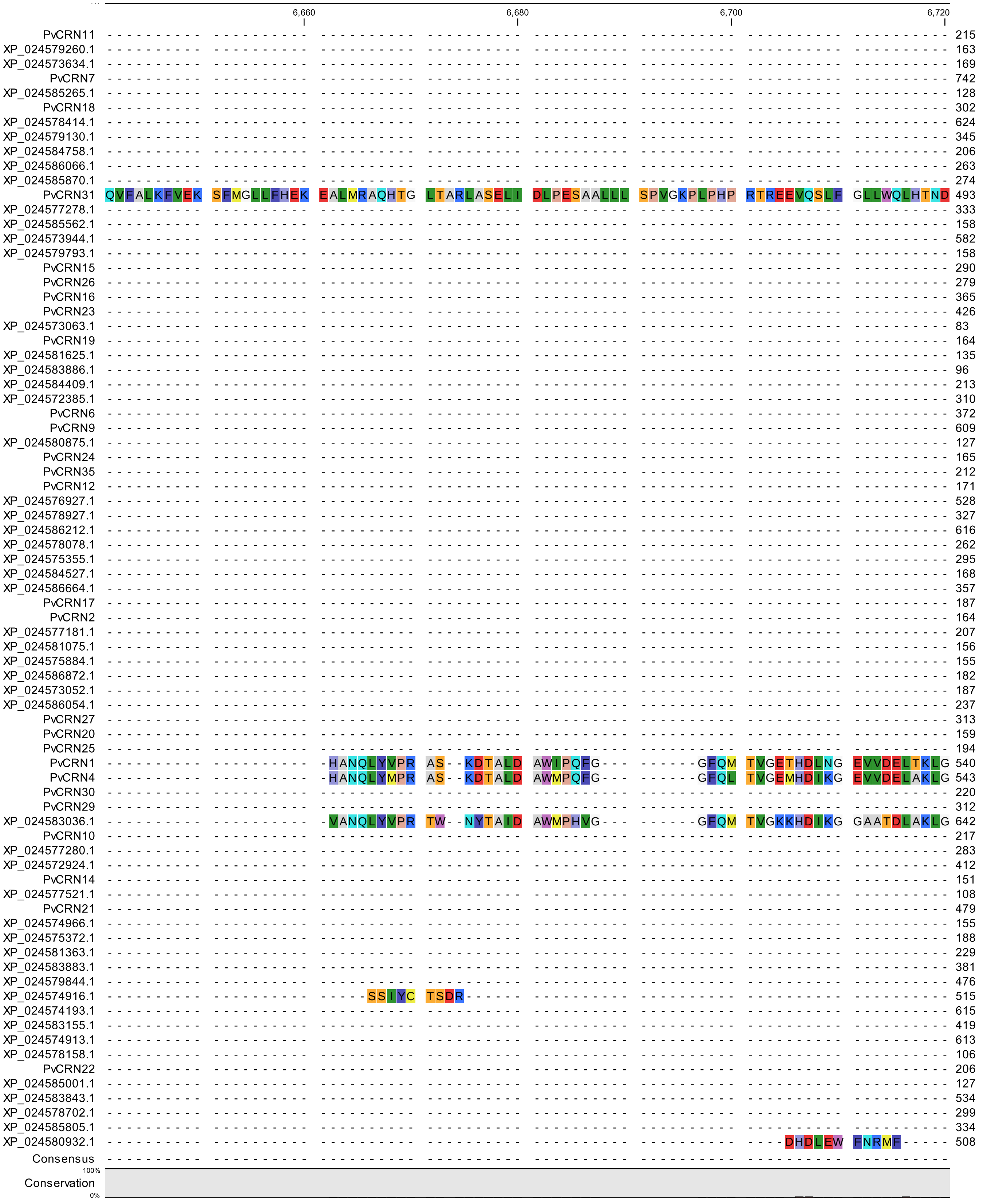

6,660 6,680 6,700 6,720
PvCRN11 ---------- ---------- ---------- ---------- ---------- ---------- ---------- ---------- 215
XP_024579260.1 ---------- ---------- ---------- ---------- ---------- ---------- ---------- ---------- 163
XP_024573634.1 ---------- ---------- ---------- ---------- ---------- ---------- ---------- ---------- 169
PvCRN7 ---------- ---------- ---------- ---------- ---------- ---------- ---------- ---------- 742
XP_024585265.1 ---------- ---------- ---------- ---------- ---------- ---------- ---------- ---------- 128
PvCRN18 ---------- ---------- ---------- ---------- ---------- ---------- ---------- ---------- 302
XP_024578414.1 ---------- ---------- ---------- ---------- ---------- ---------- ---------- ---------- 624
XP_024579130.1 ---------- ---------- ---------- ---------- ---------- ---------- ---------- ---------- 345
XP_024584758.1 ---------- ---------- ---------- ---------- ---------- ---------- ---------- ---------- 206
XP_024586066.1 ---------- ---------- ---------- ---------- ---------- ---------- ---------- ---------- 263
XP_024585870.1 ---------- ---------- ---------- ---------- ---------- ---------- ---------- ---------- 274
PvCRN31 QVFALKFVEK SFMGLLFHEK EALMRAQHTG LTARLASELI DLPESAALLL SPVGKPLPHP RTREEVQSLF GLLWQLHTND 493
XP_024577278.1 ---------- ---------- ---------- ---------- ---------- ---------- ---------- ---------- 333
XP_024585562.1 ---------- ---------- ---------- ---------- ---------- ---------- ---------- ---------- 158
XP_024573944.1 ---------- ---------- ---------- ---------- ---------- ---------- ---------- ---------- 582
XP_024579793.1 ---------- ---------- ---------- ---------- ---------- ---------- ---------- ---------- 158
PvCRN15 ---------- ---------- ---------- ---------- ---------- ---------- ---------- ---------- 290
PvCRN26 ---------- ---------- ---------- ---------- ---------- ---------- ---------- ---------- 279
PvCRN16 ---------- ---------- ---------- ---------- ---------- ---------- ---------- ---------- 365
PvCRN23 ---------- ---------- ---------- ---------- ---------- ---------- ---------- ---------- 426
XP_024573063.1 ---------- ---------- ---------- ---------- ---------- ---------- ---------- ---------- 83
PvCRN19 ---------- ---------- ---------- ---------- ---------- ---------- ---------- ---------- 164
XP_024581625.1 ---------- ---------- ---------- ---------- ---------- ---------- ---------- ---------- 135
XP_024583886.1 ---------- ---------- ---------- ---------- ---------- ---------- ---------- ---------- 96
XP_024584409.1 ---------- ---------- ---------- ---------- ---------- ---------- ---------- ---------- 213
XP_024572385.1 ---------- ---------- ---------- ---------- ---------- ---------- ---------- ---------- 310
PvCRN6 ---------- ---------- ---------- ---------- ---------- ---------- ---------- ---------- 372
PvCRN9 ---------- ---------- ---------- ---------- ---------- ---------- ---------- ---------- 609
XP_024580875.1 ---------- ---------- ---------- ---------- ---------- ---------- ---------- ---------- 127
PvCRN24 ---------- ---------- ---------- ---------- ---------- ---------- ---------- ---------- 165
PvCRN35 ---------- ---------- ---------- ---------- ---------- ---------- ---------- ---------- 212
PvCRN12 ---------- ---------- ---------- ---------- ---------- ---------- ---------- ---------- 171
XP_024576927.1 ---------- ---------- ---------- ---------- ---------- ---------- ---------- ---------- 528
XP_024578927.1 ---------- ---------- ---------- ---------- ---------- ---------- ---------- ---------- 327
XP_024586212.1 ---------- ---------- ---------- ---------- ---------- ---------- ---------- ---------- 616
XP_024578078.1 ---------- ---------- ---------- ---------- ---------- ---------- ---------- ---------- 262
XP_024575355.1 ---------- ---------- ---------- ---------- ---------- ---------- ---------- ---------- 295
XP_024584527.1 ---------- ---------- ---------- ---------- ---------- ---------- ---------- ---------- 168
XP_024586664.1 ---------- ---------- ---------- ---------- ---------- ---------- ---------- ---------- 357
PvCRN17 ---------- ---------- ---------- ---------- ---------- ---------- ---------- ---------- 187
PvCRN2 ---------- ---------- ---------- ---------- ---------- ---------- ---------- ---------- 164
XP_024577181.1 ---------- ---------- ---------- ---------- ---------- ---------- ---------- ---------- 207
XP_024581075.1 ---------- ---------- ---------- ---------- ---------- ---------- ---------- ---------- 156
XP_024575884.1 ---------- ---------- ---------- ---------- ---------- ---------- ---------- ---------- 155
XP_024586872.1 ---------- ---------- ---------- ---------- ---------- ---------- ---------- ---------- 182
XP_024573052.1 ---------- ---------- ---------- ---------- ---------- ---------- ---------- ---------- 187
XP_024586054.1 ---------- ---------- ---------- ---------- ---------- ---------- ---------- ---------- 237
PvCRN27 ---------- ---------- ---------- ---------- ---------- ---------- ---------- ---------- 313
PvCRN20 ---------- ---------- ---------- ---------- ---------- ---------- ---------- ---------- 159
PvCRN25 ---------- ---------- ---------- ---------- ---------- ---------- ---------- ---------- 194
PvCRN1 ---------- ---------- -HANQLYVPR AS--KDTALD AWIPQFG--- ------GFQM TVGETHDLNG EVVDELTKLG 540
PvCRN4 ---------- ---------- -HANQLYMPR AS--KDTALD AWMPQFG--- ------GFQL TVGEMHDIKG EVVDELAKLG 543
PvCRN30 ---------- ---------- ---------- ---------- ---------- ---------- ---------- ---------- 220
PvCRN29 ---------- ---------- ---------- ---------- ---------- ---------- ---------- ---------- 312
XP_024583036.1 ---------- ---------- -VANQLYVPR TW--NYTAID AWMPHVG--- ------GFQM TVGKKHDIKG GAATDLAKLG 642
PvCRN10 ---------- ---------- ---------- ---------- ---------- ---------- ---------- ---------- 217
XP_024577280.1 ---------- ---------- ---------- ---------- ---------- ---------- ---------- ---------- 283
XP_024572924.1 ---------- ---------- ---------- ---------- ---------- ---------- ---------- ---------- 412
PvCRN14 ---------- ---------- ---------- ---------- ---------- ---------- ---------- ---------- 151
XP_024577521.1 ---------- ---------- ---------- ---------- ---------- ---------- ---------- ---------- 108
PvCRN21 ---------- ---------- ---------- ---------- ---------- ---------- ---------- ---------- 479
XP_024574966.1 ---------- ---------- ---------- ---------- ---------- ---------- ---------- ---------- 155
XP_024575372.1 ---------- ---------- ---------- ---------- ---------- ---------- ---------- ---------- 188
XP_024581363.1 ---------- ---------- ---------- ---------- ---------- ---------- ---------- ---------- 229
XP_024583883.1 ---------- ---------- ---------- ---------- ---------- ---------- ---------- ---------- 381
XP_024579844.1 ---------- ---------- ---------- ---------- ---------- ---------- ---------- ---------- 476
XP_024574916.1 ---------- ---------- -----SSIYC TSDR------ ---------- ---------- ---------- ---------- 515
XP_024574193.1 ---------- ---------- ---------- ---------- ---------- ---------- ---------- ---------- 615
XP_024583155.1 ---------- ---------- ---------- ---------- ---------- ---------- ---------- ---------- 419
XP_024574913.1 ---------- ---------- ---------- ---------- ---------- ---------- ---------- ---------- 613
XP_024578158.1 ---------- ---------- ---------- ---------- ---------- ---------- ---------- ---------- 106
PvCRN22 ---------- ---------- ---------- ---------- ---------- ---------- ---------- ---------- 206
XP_024585001.1 ---------- ---------- ---------- ---------- ---------- ---------- ---------- ---------- 127
XP_024583843.1 ---------- ---------- ---------- ---------- ---------- ---------- ---------- ---------- 534
XP_024578702.1 ---------- ---------- ---------- ---------- ---------- ---------- ---------- ---------- 299
XP_024585805.1 ---------- ---------- ---------- ---------- ---------- ---------- ---------- ---------- 334
XP_024580932.1 ---------- ---------- ---------- ---------- ---------- ---------- ----DHDLEW FNRMF----- 508
Consensus ---------- ---------- ---------- ---------- ---------- ---------- ---------- ----------
100%
Conservation
0%

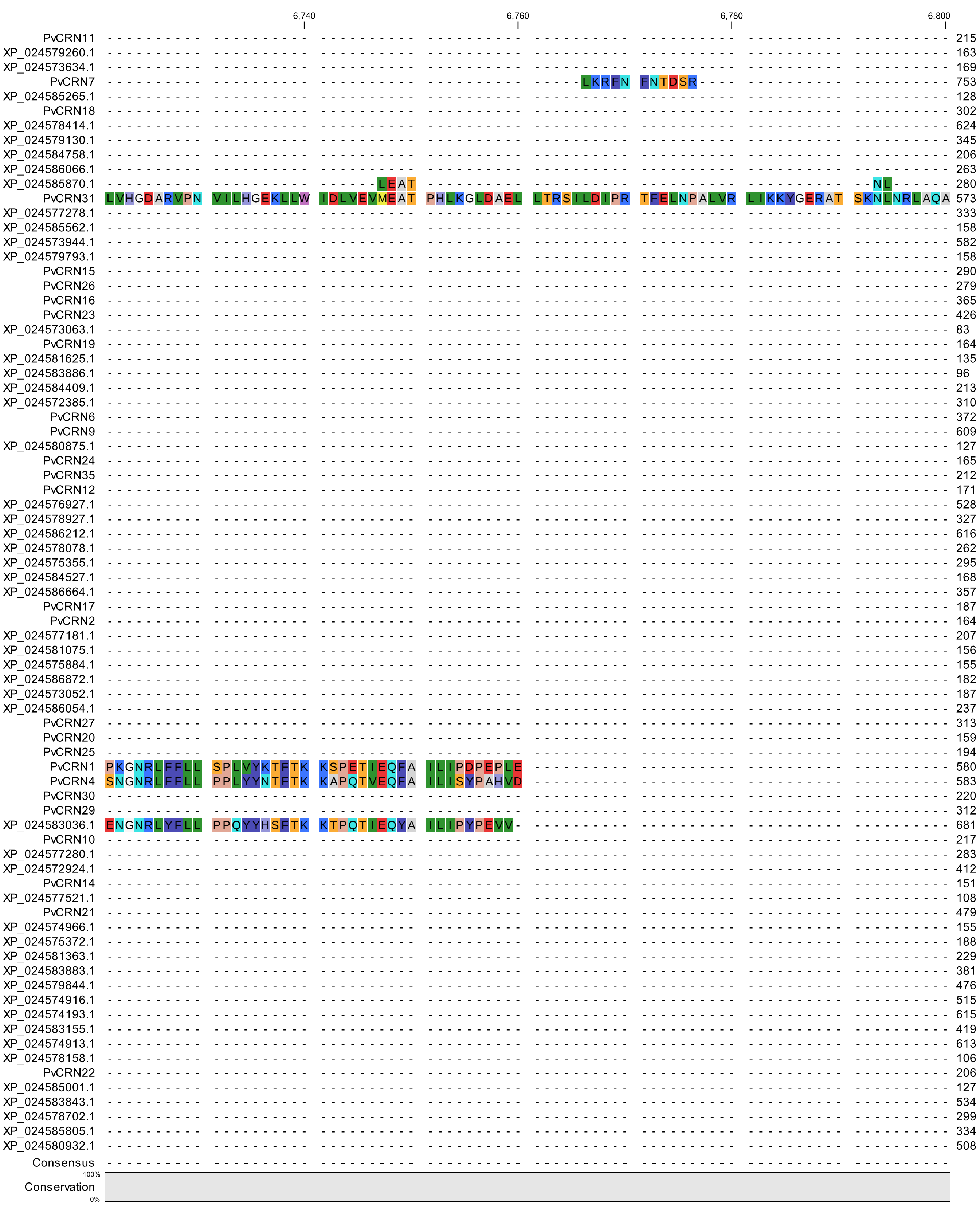
6,740
6,760
6,780
6,800
PvCRN11 215
XP_024579260.1 163
XP_024573634.1 169
PvCRN7 LKRFN FNTDSR 753
XP_024585265.1 128
PvCRN18 302
XP_024578414.1 624
XP_024579130.1 345
XP_024584758.1 206
XP_024586066.1 263
XP_024585870.1 LEAT NL 280
PvCRN31 LVHGDARVPN VILHGEKLLW IDLVEVMEAT PHLKGLDAEL LTRSILDIPR TFELNPALVR LIKKYGERAT SKNLNRLAQA 573
XP_024577278.1 333
XP_024585562.1 158
XP_024573944.1 582
XP_024579793.1 158
PvCRN15 290
PvCRN26 279
PvCRN16 365
PvCRN23 426
XP_024573063.1 83
PvCRN19 164
XP_024581625.1 135
XP_024583886.1 96
XP_024584409.1 213
XP_024572385.1 310
PvCRN6 372
PvCRN9 609
XP_024580875.1 127
PvCRN24 165
PvCRN35 212
PvCRN12 171
XP_024576927.1 528
XP_024578927.1 327
XP_024586212.1 616
XP_024578078.1 262
XP_024575355.1 295
XP_024584527.1 168
XP_024586664.1 357
PvCRN17 187
PvCRN2 164
XP_024577181.1 207
XP_024581075.1 156
XP_024575884.1 155
XP_024586872.1 182
XP_024573052.1 187
XP_024586054.1 237
PvCRN27 313
PvCRN20 159
PvCRN25 194
PvCRN1 PKGNRLFFLL SPLVYKTFTK KSPETIEQFA ILIPDPEPLE 580
PvCRN4 SNGNRLFFLL PPLYYNTFTK KAPQTVEQFA ILISYPAHVD 583
PvCRN30 220
PvCRN29 312
XP_024583036.1 ENGNRLYFLL PPQYYHSFTK KTPQTIEQYA ILIPYPEVV 681
PvCRN10 217
XP_024577280.1 283
XP_024572924.1 412
PvCRN14 151
XP_024577521.1 108
PvCRN21 479
XP_024574966.1 155
XP_024575372.1 188
XP_024581363.1 229
XP_024583883.1 381
XP_024579844.1 476
XP_024574916.1 515
XP_024574193.1 615
XP_024583155.1 419
XP_024574913.1 613
XP_024578158.1 106
PvCRN22 206
XP_024585001.1 127
XP_024583843.1 534
XP_024578702.1 299
XP_024585805.1 334
XP_024580932.1 508
Consensus
100%
Conservation
0%

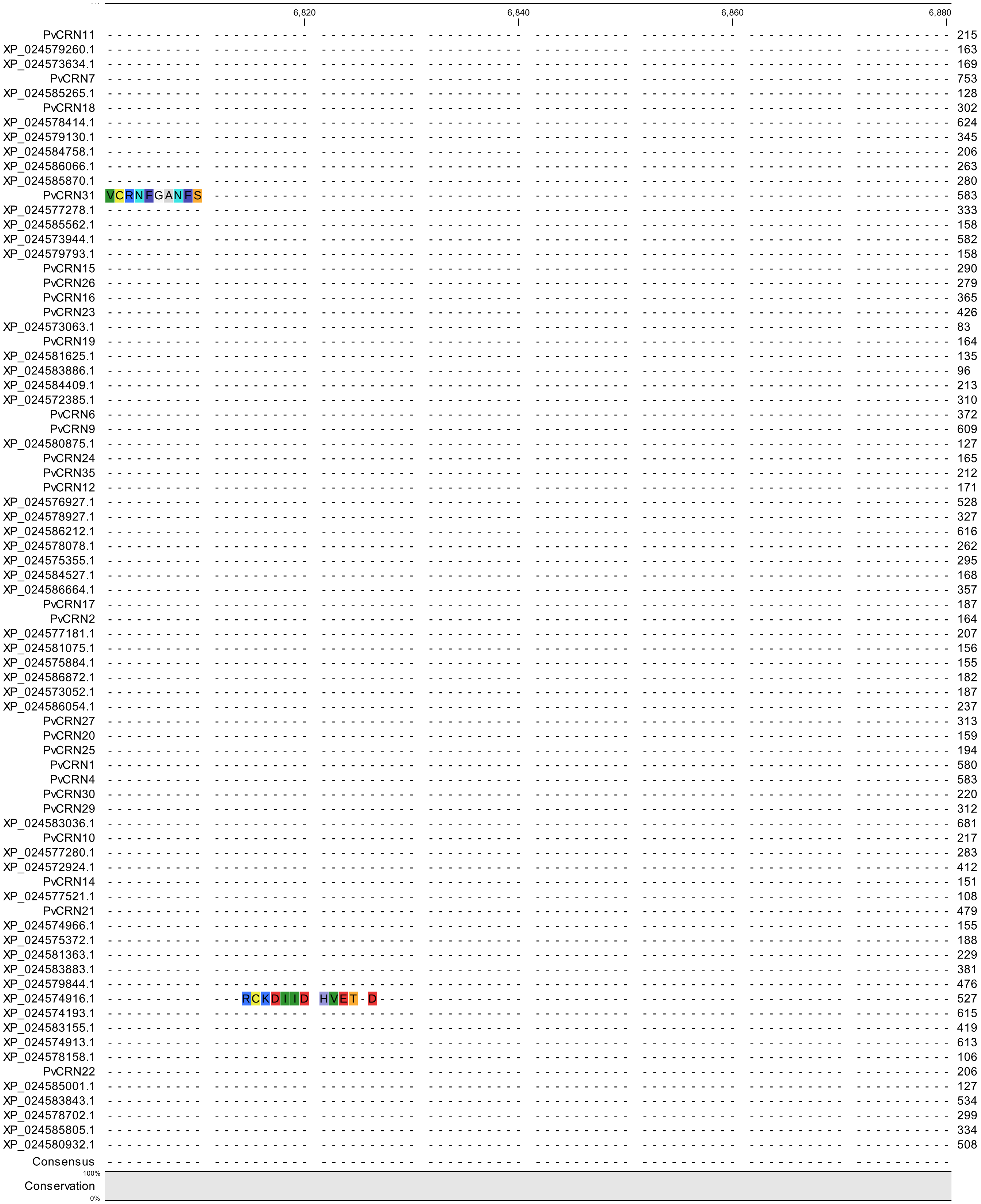

6,820
6,840
6,860
6,880
PvCRN11 215
XP_024579260.1 163
XP_024573634.1 169
PvCRN7 753
XP_024585265.1 128
PvCRN18 302
XP_024578414.1 624
XP_024579130.1 345
XP_024584758.1 206
XP_024586066.1 263
XP_024585870.1 280
PvCRN31 VCRNFGANFS 583
XP_024577278.1 333
XP_024585562.1 158
XP_024573944.1 582
XP_024579793.1 158
PvCRN15 290
PvCRN26 279
PvCRN16 365
PvCRN23 426
XP_024573063.1 83
PvCRN19 164
XP_024581625.1 135
XP_024583886.1 96
XP_024584409.1 213
XP_024572385.1 310
PvCRN6 372
PvCRN9 609
XP_024580875.1 127
PvCRN24 165
PvCRN35 212
PvCRN12 171
XP_024576927.1 528
XP_024578927.1 327
XP_024586212.1 616
XP_024578078.1 262
XP_024575355.1 295
XP_024584527.1 168
XP_024586664.1 357
PvCRN17 187
PvCRN2 164
XP_024577181.1 207
XP_024581075.1 156
XP_024575884.1 155
XP_024586872.1 182
XP_024573052.1 187
XP_024586054.1 237
PvCRN27 313
PvCRN20 159
PvCRN25 194
PvCRN1 580
PvCRN4 583
PvCRN30 220
PvCRN29 312
XP_024583036.1 681
PvCRN10 217
XP_024577280.1 283
XP_024572924.1 412
PvCRN14 151
XP_024577521.1 108
PvCRN21 479
XP_024574966.1 155
XP_024575372.1 188
XP_024581363.1 229
XP_024583883.1 381
XP_024579844.1 476
XP_024574916.1 RCKDIID HVET-D 527
XP_024574193.1 615
XP_024583155.1 419
XP_024574913.1 613
XP_024578158.1 106
PvCRN22 206
XP_024585001.1 127
XP_024583843.1 534
XP_024578702.1 299
XP_024585805.1 334
XP_024580932.1 508
Consensus
100%
Conservation
0%

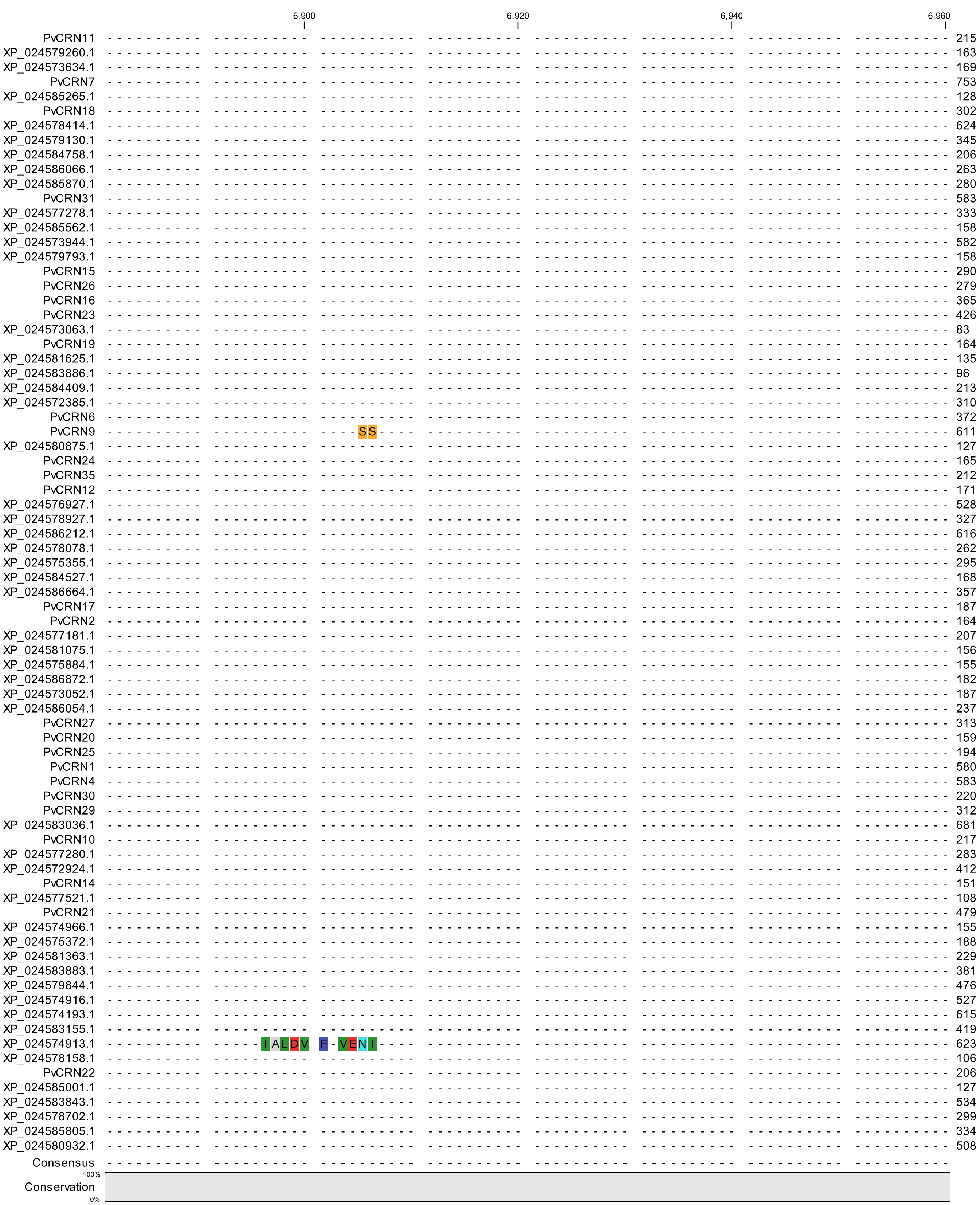
6,900
6,920
6,940
6,960
PvCRN11 215
XP_024579260.1 163
XP_024573634.1 169
PvCRN7 753
XP_024585265.1 128
PvCRN18 302
XP_024578414.1 624
XP_024579130.1 345
XP_024584758.1 206
XP_024586066.1 263
XP_024585870.1 280
PvCRN31 583
XP_024577278.1 333
XP_024585562.1 158
XP_024573944.1 582
XP_024579793.1 158
PvCRN15 290
PvCRN26 279
PvCRN16 365
PvCRN23 426
XP_024573063.1 83
PvCRN19 164
XP_024581625.1 135
XP_024583886.1 96
XP_024584409.1 213
XP_024572385.1 310
PvCRN6 372
PvCRN9 SS 611
XP_024580875.1 127
PvCRN24 165
PvCRN35 212
PvCRN12 171
XP_024576927.1 528
XP_024578927.1 327
XP_024586212.1 616
XP_024578078.1 262
XP_024575355.1 295
XP_024584527.1 168
XP_024586664.1 357
PvCRN17 187
PvCRN2 164
XP_024577181.1 207
XP_024581075.1 156
XP_024575884.1 155
XP_024586872.1 182
XP_024573052.1 187
XP_024586054.1 237
PvCRN27 313
PvCRN20 159
PvCRN25 194
PvCRN1 580
PvCRN4 583
PvCRN30 220
PvCRN29 312
XP_024583036.1 681
PvCRN10 217
XP_024577280.1 283
XP_024572924.1 412
PvCRN14 151
XP_024577521.1 108
PvCRN21 479
XP_024574966.1 155
XP_024575372.1 188
XP_024581363.1 229
XP_024583883.1 381
XP_024579844.1 476
XP_024574916.1 527
XP_024574193.1 615
XP_024583155.1 419
XP_024574913.1 IALDV F-VENI 623
XP_024578158.1 106
PvCRN22 206
XP_024585001.1 127
XP_024583843.1 534
XP_024578702.1 299
XP_024585805.1 334
XP_024580932.1 508
Consensus
100%
Conservation
0%

| | 6,980 · 7,000 · 7,020 · 7,040 | |
|---|---|---|
| PvCRN11 | ---------- ---------- ---------- ---------- ---------- ---------- ---------- ---------- | 215 |
| XP_024579260.1 | ---------- ---------- ---------- ---------- ---------- ---------- ---------- ---------- | 163 |
| XP_024573634.1 | ---------- ---------- ---------- ---------- ---------- ---------- ---------- ---------- | 169 |
| PvCRN7 | ---------- ---------- ---------- ---------- ---------- ---------- ---------- ---------- | 753 |
| XP_024585265.1 | ---------- ---------- ---------- ---------- ---------- ---------- ---------- ---------- | 128 |
| PvCRN18 | ---------- ---------- ---------- ---------- ---------- ---------- ---------- ---------- | 302 |
| XP_024578414.1 | ---------- ---------- ---------- ---------- ---------- ---------- ---------- ---------- | 624 |
| XP_024579130.1 | ---------- ---------- ---------- ---------- ---------- ---------- ---------- ---------- | 345 |
| XP_024584758.1 | ---------- ---------- ---------- ---------- ---------- ---------- ---------- ---------- | 206 |
| XP_024586066.1 | ---------- ---------- ---------- ---------- ---------- ---------- ---------- ---------- | 263 |
| XP_024585870.1 | ---------- ---------- ---------- ---------- ---------- ---------- ---------- ---------- | 280 |
| PvCRN31 | ---------- ---------- ---------- ---------- ---------- ---------- ---------- ---------- | 583 |
| XP_024577278.1 | ---------- ---------- ---------- ---------- ---------- ---------- ---------- ---------- | 333 |
| XP_024585562.1 | ---------- ---------- ---------- ---------- ---------- ---------- ---------- ---------- | 158 |
| XP_024573944.1 | ---------- ---------- ---------- ---------- ---------- ---------- ---------- ---------- | 582 |
| XP_024579793.1 | ---------- ---------- ---------- ---------- ---------- ---------- ---------- ---------- | 158 |
| PvCRN15 | ---------- ---------- ---------- ---------- ---------- ---------- ---------- ---------- | 290 |
| PvCRN26 | ---------- ---------- ---------- ---------- ---------- ---------- ---------- ---------- | 279 |
| PvCRN16 | ---------- ---------- ---------- ---------- ---------- ---------- ---------- ---------- | 365 |
| PvCRN23 | ---------- ---------- ---------- ---------- ---------- ---------- ---------- ---------- | 426 |
| XP_024573063.1 | ---------- ---------- ---------- ---------- ---------- ---------- ---------- ---------- | 83 |
| PvCRN19 | ---------- ---------- ---------- ---------- ---------- ---------- ---------- ---------- | 164 |
| XP_024581625.1 | ---------- ---------- ---------- ---------- ---------- ---------- ---------- ---------- | 135 |
| XP_024583886.1 | ---------- ---------- ---------- ---------- ---------- ---------- ---------- ---------- | 96 |
| XP_024584409.1 | ---------- ---------- ---------- ---------- ---------- ---------- ---------- ---------- | 213 |
| XP_024572385.1 | ---------- ---------- ---------- ---------- ---------- ---------- ---------- ---------- | 310 |
| PvCRN6 | ---------- ---------- ---------- ---------- ---------- ---------- ---------- ---------- | 372 |
| PvCRN9 | ---------- ---------- ---------- ---------- ---------- ---------- ---------- ---------- | 611 |
| XP_024580875.1 | ---------- ---------- ---------- ---------- ---------- ---------- ---------- ---------- | 127 |
| PvCRN24 | ---------- ---------- ---------- ---------- ---------- ---------- ---------- ---------- | 165 |
| PvCRN35 | ---------- ---------- ---------- ---------- ---------- ---------- ---------- ---------- | 212 |
| PvCRN12 | ---------- ---------- ---------- ---------- ---------- ---------- ---------- ---------- | 171 |
| XP_024576927.1 | ---------- ---------- ---------- ---------- ---------- ---------- ---------- ---------- | 528 |
| XP_024578927.1 | ---------- ---------- ---------- ---------- ---------- ---------- ---------- ---------- | 327 |
| XP_024586212.1 | ---------- ---------- ---------- ---------- ---------- ---------- ---------- ---------- | 616 |
| XP_024578078.1 | ---------- ---------- ---------- ---------- ---------- ---------- ---------- ---------- | 262 |
| XP_024575355.1 | ---------- ---------- ---------- ---------- ---------- ---------- ---------- ---------- | 295 |
| XP_024584527.1 | ---------- ---------- ---------- ---------- ---------- ---------- ---------- ---------- | 168 |
| XP_024586664.1 | ---------- ---------- ---------- ---------- ---------- ---------- ---------- ---------- | 357 |
| PvCRN17 | ---------- ---------- ---------- ---------- ---------- ---------- ---------- ---------- | 187 |
| PvCRN2 | ---------- ---------- ---------- ---------- ---------- ---------- ---------- ---------- | 164 |
| XP_024577181.1 | ---------- ---------- ---------- ---------- ---------- ---------- ---------- ---------- | 207 |
| XP_024581075.1 | ---------- ---------- ---------- ---------- ---------- ---------- ---------- ---------- | 156 |
| XP_024575884.1 | ---------- ---------- ---------- ---------- ---------- ---------- ---------- ---------- | 155 |
| XP_024586872.1 | ---------- ---------- ---------- ---------- ---------- ---------- ---------- ---------- | 182 |
| XP_024573052.1 | ---------- ---------- ---------- ---------- ---------- ---------- ---------- ---------- | 187 |
| XP_024586054.1 | ---------- ---------- ---------- ---------- ---------- ---------- ---------- ---------- | 237 |
| PvCRN27 | ---------- ---------- ---------- ---------- ---------- ---------- ---------- ---------- | 313 |
| PvCRN20 | ---------- ---------- ---------- ---------- ---------- ---------- ---------- ---------- | 159 |
| PvCRN25 | ---------- ---------- ---------- ---------- ---------- ---------- ---------- ---------- | 194 |
| PvCRN1 | ---------- ---------- ---------- ---------- ---------- ---------- ---------- ---------- | 580 |
| PvCRN4 | ---------- ---------- ---------- ---------- ---------- ---------- ---------- ---------- | 583 |
| PvCRN30 | ---------- ---------- ---------- ---------- ---------- ---------- ---------- ---------- | 220 |
| PvCRN29 | ---------- ---------- ---------- ---------- ---------- ---------- ---------- ---------- | 312 |
| XP_024583036.1 | ---------- ---------- ---------- ---------- ---------- ---------- ---------- ---------- | 681 |
| PvCRN10 | ---------- ---------- ---------- ---------- ---------- ---------- ---------- ---------- | 217 |
| XP_024577280.1 | ---------- ---------- ---------- ---------- ---------- ---------- ---------- ---------- | 283 |
| XP_024572924.1 | ---------- ---------- ---------- ---------- ---------- ---------- ---------- ---------- | 412 |
| PvCRN14 | ---------- ---------- ---------- ---------- ---------- ---------- ---------- ---------- | 151 |
| XP_024577521.1 | ---------- ---------- ---------- ---------- ---------- ---------- ---------- ---------- | 108 |
| PvCRN21 | ---------- ---------- ---------- ---------- ---------- ---------- ---------- ---------- | 479 |
| XP_024574966.1 | ---------- ---------- ---------- ---------- ---------- ---------- ---------- ---------- | 155 |
| XP_024575372.1 | ---------- ---------- ---------- ---------- ---------- ---------- ---------- ---------- | 188 |
| XP_024581363.1 | ---------- ---------- ---------- ---------- ---------- ---------- ---------- ---------- | 229 |
| XP_024583883.1 | ---------- ---------- ---------- ---------- ---------- ---------- ---------- ---------- | 381 |
| XP_024579844.1 | ---------- ---------- ---------- ---------- ---------- ---------- ---------- ---------- | 476 |
| XP_024574916.1 | ---------- ---------- ---------- ---------- ---------- ---------- ---------- ---------- | 527 |
| XP_024574193.1 | ---------- ---------- ---------- ---------- ---------- ---------- ---------- ---------- | 615 |
| XP_024583155.1 | ---------- ---------- ---------- ---------- ---------- ---------- ---------- ---------- | 419 |
| XP_024574913.1 | ---------- ---------- ---------- ---------- ---------- ---------- ---------- ---------- | 623 |
| XP_024578158.1 | ---------- ---------- ---------- ---------- ---------- ---------- ---------- ---------- | 106 |
| PvCRN22 | ---------- ---------- ---------- ---------- ---------- ---------- ---------- ---------- | 206 |
| XP_024585001.1 | ---------- ---------- ---------- ---------- ---------- ---------- ---------- ---------- | 127 |
| XP_024583843.1 | ---------- ---------- ---------- ---------- ---------- ---------- ---------- ---------- | 534 |
| XP_024578702.1 | ---------- ---------- ---------- ---------- ---------- ---------- ---------- ---------- | 299 |
| XP_024585805.1 | ---------- ---------- ---------- ---------- ---------- ---------- ---------- ---------- | 334 |
| XP_024580932.1 | ---------- ---------- ---------- ---------- ---------- ---------- ---------- ---------- | 508 |
| Consensus | ---------- ---------- ---------- ---------- ---------- ---------- ---------- ---------- | |

Conservation (100% – 0%)

| | 7,060 · 7,080 · 7,100 · 7,120 | |
|---|---|---|
| PvCRN11 | ---------- ---------- ---------- ---------- ---------- ---------- ---------- ---------- | 215 |
| XP_024579260.1 | ---------- ---------- ---------- ---------- ---------- ---------- ---------- ---------- | 163 |
| XP_024573634.1 | ---------- ---------- ---------- ---------- ---------- ---------- ---------- ---------- | 169 |
| PvCRN7 | ---------- ---------- ---------- ---------- ---------- ---------- ---------- ---------- | 753 |
| XP_024585265.1 | ---------- ---------- ---------- ---------- ---------- ---------- ---------- ---------- | 128 |
| PvCRN18 | ---------- ---------- ---------- ---------- ---------- ---------- ---------- ---------- | 302 |
| XP_024578414.1 | ---------- ---------- ---------- ---------- ---------- ---------- ---------- ---------- | 624 |
| XP_024579130.1 | ---------- ---------- ---------- ---------- ---------- ---------- ---------- ---------- | 345 |
| XP_024584758.1 | ---------- ---------- ---------- ---------- ---------- ---------- ---------- ---------- | 206 |
| XP_024586066.1 | ---------- ---------- ---------- ---------- ---------- ---------- ---------- ---------- | 263 |
| XP_024585870.1 | ---------- ---------- ---------- ---------- ---------- ---------- ---------- ---------- | 280 |
| PvCRN31 | ---------- ---------- ---------- ---------- ---------- ---------- ---------- ---------- | 583 |
| XP_024577278.1 | ---------- ---------- ---------- ---------- ---------- ---------- ---------- ---------- | 333 |
| XP_024585562.1 | ---------- ---------- ---------- ---------- ---------- ---------- ---------- ---------- | 158 |
| XP_024573944.1 | ---------- ---------- ---------- ---------- ---------- ---------- ---------- ---------- | 582 |
| XP_024579793.1 | ---------- ---------- ---------- ---------- ---------- ---------- ---------- ---------- | 158 |
| PvCRN15 | ---------- ---------- ---------- ---------- ---------- ---------- ---------- ---------- | 290 |
| PvCRN26 | ---------- ---------- ---------- ---------- ---------- ---------- ---------- ---------- | 279 |
| PvCRN16 | ---------- ---------- ---------- ---------- ---------- ---------- ---------- ---------- | 365 |
| PvCRN23 | ---------- ---------- ---------- ---------- ---------- ---------- ---------- ---------- | 426 |
| XP_024573063.1 | ---------- ---------- ---------- ---------- ---------- ---------- ---------- ---------- | 83 |
| PvCRN19 | ---------- ---------- ---------- ---------- ---------- ---------- ---------- ---------- | 164 |
| XP_024581625.1 | ---------- ---------- ---------- ---------- ---------- ---------- ---------- ---------- | 135 |
| XP_024583886.1 | ---------- ---------- ---------- ---------- ---------- ---------- ---------- ---------- | 96 |
| XP_024584409.1 | ---------- ---------- ---------- ---------- ---------- ---------- ---------- ---------- | 213 |
| XP_024572385.1 | ---------- ---------- ---------- ---------- ---------- ---------- ---------- ---------- | 310 |
| PvCRN6 | ---------- ---------- ---------- ---------- ---------- ---------- ---------- ---------- | 372 |
| PvCRN9 | ---------- ---------- ---------- ---------- ---------- ---------- ---------- ---------- | 611 |
| XP_024580875.1 | ---------- ---------- ---------- ---------- ---------- ---------- ---------- ---------- | 127 |
| PvCRN24 | ---------- ---------- ---------- ---------- ---------- ---------- ---------- ---------- | 165 |
| PvCRN35 | ---------- ---------- ---------- ---------- ---------- ---------- ---------- ---------- | 212 |
| PvCRN12 | ---------- ---------- ---------- ---------- ---------- ---------- ---------- ---------- | 171 |
| XP_024576927.1 | ---------- ---------- ---------- ---------- ---------- ---------- ---------- ---------- | 528 |
| XP_024578927.1 | ---------- ---------- ---------- ---------- ---------- ---------- ---------- ---------- | 327 |
| XP_024586212.1 | ---------- ---------- ---------- ---------- ---------- ---------- ---------- ---------- | 616 |
| XP_024578078.1 | ---------- ---------- ---------- ---------- ---------- ---------- ---------- ---------- | 262 |
| XP_024575355.1 | ---------- ---------- ---------- ---------- ---------- ---------- ---------- ---------- | 295 |
| XP_024584527.1 | ---------- ---------- ---------- ---------- ---------- ---------- ---------- ---------- | 168 |
| XP_024586664.1 | ---------- ---------- ---------- ---------- ---------- ---------- ---------- ---------- | 357 |
| PvCRN17 | ---------- ---------- ---------- ---------- ---------- ---------- ---------- ---------- | 187 |
| PvCRN2 | ---------- ---------- ---------- ---------- ---------- ---------- ---------- ---------- | 164 |
| XP_024577181.1 | ---------- ---------- ---------- ---------- ---------- ---------- ---------- ---------- | 207 |
| XP_024581075.1 | ---------- ---------- ---------- ---------- ---------- ---------- ---------- ---------- | 156 |
| XP_024575884.1 | ---------- ---------- ---------- ---------- ---------- ---------- ---------- ---------- | 155 |
| XP_024586872.1 | ---------- ---------- ---------- ---------- ---------- ---------- ---------- ---------- | 182 |
| XP_024573052.1 | ---------- ---------- ---------- ---------- ---------- ---------- ---------- ---------- | 187 |
| XP_024586054.1 | ---------- ---------- ---------- ---------- ---------- ---------- ---------- ---------- | 237 |
| PvCRN27 | ---------- ---------- ---------- ---------- ---------- ---------- ---------- ---------- | 313 |
| PvCRN20 | ---------- ---------- ---------- ---------- ---------- ---------- ---------- ---------- | 159 |
| PvCRN25 | ---------- ---------- ---------- ---------- ---------- ---------- ---------- ---------- | 194 |
| PvCRN1 | ---------- ---------- ---------- ---------- ---------- ---------- ---------- ---------- | 580 |
| PvCRN4 | ---------- ---------- ---------- ---------- ---------- ---------- ---------- ---------- | 583 |
| PvCRN30 | ---------- ---------- ---------- ---------- ---------- ---------- ---------- ---------- | 220 |
| PvCRN29 | ---------- ---------- ---------- ---------- ---------- ---------- ---------- ---------- | 312 |
| XP_024583036.1 | ---------- ---------- ---------- ---------- ---------- ---------- ---------- ---------- | 681 |
| PvCRN10 | ---------- ---------- ---------- ---------- ---------- ---------- ---------- ---------- | 217 |
| XP_024577280.1 | ---------- ---------- ---------- ---------- ---------- ---------- ---------- ---------- | 283 |
| XP_024572924.1 | ---------- ---------- ---------- ---------- ---------- ---------- ---------- ---------- | 412 |
| PvCRN14 | ---------- ---------- ---------- ---------- ---------- ---------- ---------- ---------- | 151 |
| XP_024577521.1 | ---------- ---------- ---------- ---------- ---------- ---------- ---------- ---------- | 108 |
| PvCRN21 | ---------- ---------- ---------- ---------- ---------- ---------- ---------- ---------- | 479 |
| XP_024574966.1 | ---------- ---------- ---------- ---------- ---------- ---------- ---------- ---------- | 155 |
| XP_024575372.1 | ---------- ---------- ---------- ---------- ---------- ---------- ---------- ---------- | 188 |
| XP_024581363.1 | ---------- ---------- ---------- ---------- ---------- ---------- ---------- ---------- | 229 |
| XP_024583883.1 | ---------- ---------- ---------- ---------- ---------- ---------- ---------- ---------- | 381 |
| XP_024579844.1 | ---------- ---------- ---------- ---------- ---------- ---------- ---------- ---------- | 476 |
| XP_024574916.1 | ---------- ---------- ---------- ---------- ---------- ---------- ---------- ---------- | 527 |
| XP_024574193.1 | ---------- ---------- ---------- ---------- ---------- ---------- ---------- ---------- | 615 |
| XP_024583155.1 | ---------- ---------- ---------- ---------- ---------- ---------- ---------- ---------- | 419 |
| XP_024574913.1 | ---------- ---------- ---------- ---------- ---------- ---------- ---------- ---------- | 623 |
| XP_024578158.1 | ---------- ---------- ---------- ---------- ---------- ---------- ---------- ---------- | 106 |
| PvCRN22 | ---------- ---------- ---------- ---------- ---------- ---------- ---------- ---------- | 206 |
| XP_024585001.1 | ---------- ---------- ---------- ---------- ---------- ---------- ---------- ---------- | 127 |
| XP_024583843.1 | ---------- ---------- ---------- ---------- ---------- ---------- ---------- ---------- | 534 |
| XP_024578702.1 | ---------- ---------- ---------- ---------- ---------- ---------- ---------- ---------- | 299 |
| XP_024585805.1 | ---------- ---------- ---------- ---------- ---------- ---------- ---------- ---------- | 334 |
| XP_024580932.1 | ---------- ---------- ---------- ---------- ---------- ---------- ---------- ---------- | 508 |
| Consensus | ---------- ---------- ---------- ---------- ---------- ---------- ---------- ---------- | |
| Conservation (100% / 0%) | | |

```
                  7,140                7,160                7,180                7,200
                    |                    |                    |                    |
PvCRN11         ---------- ---------- ---------- ---------- ---------- ---------- ---------- ---------- 215
XP_024579260.1  ---------- ---------- ---------- ---------- ---------- ---------- ---------- ---------- 163
XP_024573634.1  ---------- ---------- ---------- ---------- ---------- ---------- ---------- ---------- 169
PvCRN7          ---------- ---------- ---------- ---------- ---------- ---------- ---------- ---------- 753
XP_024585265.1  ---------- ---------- ---------- ---------- ---------- ---------- ---------- ---------- 128
PvCRN18         ---------- ---------- ---------- ---------- ---------- ---------- ---------- ---------- 302
XP_024578414.1  ---------- ---------- ---------- ---------- ---------- ---------- ---------- ---------- 624
XP_024579130.1  ---------- ---------- ---------- ---------- ---------- ---------- ---------- ---------- 345
XP_024584758.1  ---------- ---------- ---------- ---------- ---------- ---------- ---------- ---------- 206
XP_024586066.1  ---------- ---------- ---------- ---------- ---------- ---------- ---------- ---------- 263
XP_024585870.1  ---------- ---------- ---------- ---------- ---------- ---------- ---------- ---------- 280
PvCRN31         ---------- ---------- ---------- ---------- ---------- ---------- ---------- ---------- 583
XP_024577278.1  ---------- ---------- ---------- ---------- ---------- ---------- ---------- ---------- 333
XP_024585562.1  ---------- ---------- ---------- ---------- ---------- ---------- ---------- ---------- 158
XP_024573944.1  ---------- ---------- ---------- ---------- ---------- ---------- ---------- ---------- 582
XP_024579793.1  ---------- ---------- ---------- ---------- ---------- ---------- ---------- ---------- 158
PvCRN15         ---------- ---------- ---------- ---------- ---------- ---------- ---------- ---------- 290
PvCRN26         ---------- ---------- ---------- ---------- ---------- ---------- ---------- ---------- 279
PvCRN16         ---------- ---------- ---------- ---------- ---------- ---------- ---------- ---------- 365
PvCRN23         ---------- ---------- ---------- ---------- ---------- ---------- ---------- ---------- 426
XP_024573063.1  ---------- ---------- ---------- ---------- ---------- ---------- ---------- ---------- 83
PvCRN19         ---------- ---------- ---------- ---------- ---------- ---------- ---------- ---------- 164
XP_024581625.1  ---------- ---------- ---------- ---------- ---------- ---------- ---------- ---------- 135
XP_024583886.1  ---------- ---------- ---------- ---------- ---------- ---------- ---------- ---------- 96
XP_024584409.1  ---------- ---------- ---------- ---------- ---------- ---------- ---------- ---------- 213
XP_024572385.1  ---------- ---------- ---------- ---------- ---------- ---------- ---------- ---------- 310
PvCRN6          ---------- ---------- ---------- ---------- ---------- ---------- ---------- ---------- 372
PvCRN9          ---------- ---------- ---------- ---------- ---------- ---------- ---------- ---------- 611
XP_024580875.1  ---------- ---------- ---------- ---------- ---------- ---------- ---------- ---------- 127
PvCRN24         ---------- ---------- ---------- ---------- ---------- ---------- ---------- ---------- 165
PvCRN35         ---------- ---------- ---------- ---------- ---------- ---------- ---------- ---------- 212
PvCRN12         ---------- ---------- ---------- ---------- ---------- ---------- ---------- ---------- 171
XP_024576927.1  ---------- ---------- ---------- ---------- ---------- ---------- ---------- ---------- 528
XP_024578927.1  ---------- ---------- ---------- ---------- ---------- ---------- ---------- ---------- 327
XP_024586212.1  ---------- ---------- ---------- ---------- ---------- ---------- ---------- ---------- 616
XP_024578078.1  ---------- ---------- ---------- ---------- ---------- ---------- ---------- ---------- 262
XP_024575355.1  ---------- ---------- ---------- ---------- ---------- ---------- ---------- ---------- 295
XP_024584527.1  ---------- ---------- ---------- ---------- ---------- ---------- ---------- ---------- 168
XP_024586664.1  ---------- ---------- ---------- ---------- ---------- ---------- ---------- ---------- 357
PvCRN17         ---------- ---------- ---------- ---------- ---------- ---------- ---------- ---------- 187
PvCRN2          ---------- ---------- ---------- ---------- ---------- ---------- ---------- ---------- 164
XP_024577181.1  ---------- ---------- ---------- ---------- ---------- ---------- ---------- ---------- 207
XP_024581075.1  ---------- ---------- ---------- ---------- ---------- ---------- ---------- ---------- 156
XP_024575884.1  ---------- ---------- ---------- ---------- ---------- ---------- ---------- ---------- 155
XP_024586872.1  ---------- ---------- ---------- ---------- ---------- ---------- ---------- ---------- 182
XP_024573052.1  ---------- ---------- ---------- ---------- ---------- ---------- ---------- ---------- 187
XP_024586054.1  ---------- ---------- ---------- ---------- ---------- ---------- ---------- ---------- 237
PvCRN27         ---------- ---------- ---------- ---------- ---------- ---------- ---------- ---------- 313
PvCRN20         ---------- ---------- ---------- ---------- ---------- ---------- ---------- ---------- 159
PvCRN25         ---------- ---------- ---------- ---------- ---------- ---------- ---------- ---------- 194
PvCRN1          ---------- ---------- ---------- ---------- ---------- ---------- ---------- ---------- 580
PvCRN4          ---------- ---------- ---------- ---------- ---------- ---------- ---------- ---------- 583
PvCRN30         ---------- ---------- ---------- ---------- ---------- ---------- ---------- ---------- 220
PvCRN29         ---------- ---------- ---------- ---------- ---------- ---------- ---------- ---------- 312
XP_024583036.1  ---------- ---------- ---------- ---------- ---------- ---------- ---------- ---------- 681
PvCRN10         ---------- ---------- ---------- ---------- ---------- ---------- ---------- ---------- 217
XP_024577280.1  ---------- ---------- ---------- ---------- ---------- ---------- ---------- ---------- 283
XP_024572924.1  ---------- ---------- ---------- ---------- ---------- ---------- ---------- ---------- 412
PvCRN14         ---------- ---------- ---------- ---------- ---------- ---------- ---------- ---------- 151
XP_024577521.1  ---------- ---------- ---------- ---------- ---------- ---------- ---------- ---------- 108
PvCRN21         ---------- ---------- ---------- ---------- ---------- ---------- ---------- ---------- 479
XP_024574966.1  ---------- ---------- ---------- ---------- ---------- ---------- ---------- ---------- 155
XP_024575372.1  ---------- ---------- ---------- ---------- ---------- ---------- ---------- ---------- 188
XP_024581363.1  ---------- ---------- ---------- ---------- ---------- ---------- ---------- ---------- 229
XP_024583883.1  ---------- ---------- ---------- ---------- ---------- ---------- ---------- ---------- 381
XP_024579844.1  ---------- ---------- ---------- ---------- ---------- ---------- ---------- ---------- 476
XP_024574916.1  ---------- ---------- ---------- ---------- ---------- ---------- ---------- ---------- 527
XP_024574193.1  ---------- ---------- ---------- ---------- ---------- ---------- ---------- ---------- 615
XP_024583155.1  ---------- ---------- ---------- ---------- ---------- ---------- ---------- ---------- 419
XP_024574913.1  ---------- ---------- ---------- ---------- ---------- ---------- ---------- ---------- 623
XP_024578158.1  ---------- ---------- ---------- ---------- ---------- ---------- ---------- ---------- 106
PvCRN22         ---------- ---------- ---------- ---------- ---------- ---------- ---------- ---------- 206
XP_024585001.1  ---------- ---------- ---------- ---------- ---------- ---------- ---------- ---------- 127
XP_024583843.1  ---------- ---------- ---------- ---------- ---------- ---------- ---------- ---------- 534
XP_024578702.1  ---------- ---------- ---------- ---------- ---------- ---------- ---------- ---------- 299
XP_024585805.1  ---------- ---------- ---------- ---------- ---------- ---------- ---------- ---------- 334
XP_024580932.1  ---------- ---------- ---------- ---------- ---------- ---------- ---------- ---------- 508
Consensus       ---------- ---------- ---------- ---------- ---------- ---------- ---------- ----------
           100%
Conservation
             0%
```

| | 7,220 7,240 7,260 7,280 | |
|---|---|---|
| PvCRN11 | ---------- ---------- ---------- ---------- ---------- ---------- ---------- ---------- | 215 |
| XP_024579260.1 | ---------- ---------- ---------- ---------- ---------- ---------- ---------- ---------- | 163 |
| XP_024573634.1 | ---------- ---------- ---------- ---------- ---------- ---------- ---------- ---------- | 169 |
| PvCRN7 | ---------- ---------- ---------- ---------- ---------- ---------- ---------- ---------- | 753 |
| XP_024585265.1 | ---------- ---------- ---------- ---------- ---------- ---------- ---------- ---------- | 128 |
| PvCRN18 | ---------- ---------- ---------- ---------- ---------- ---------- ---------- ---------- | 302 |
| XP_024578414.1 | ---------- ---------- ---------- ---------- ---------- ---------- ---------- ---------- | 624 |
| XP_024579130.1 | ---------- ---------- ---------- ---------- ---------- ---------- ---------- ---------- | 345 |
| XP_024584758.1 | ---------- ---------- ---------- ---------- ---------- ---------- ---------- ---------- | 206 |
| XP_024586066.1 | ---------- ---------- ---------- ---------- ---------- ---------- ---------- ---------- | 263 |
| XP_024585870.1 | ---------- ---------- ---------- ---------- ---------- ---------- ---------- ---------- | 280 |
| PvCRN31 | ---------- ---------- ---------- ---------- ---------- ---------- ---------- ---------- | 583 |
| XP_024577278.1 | ---------- ---------- ---------- ---------- ---------- ---------- ---------- ---------- | 333 |
| XP_024585562.1 | ---------- ---------- ---------- ---------- ---------- ---------- ---------- ---------- | 158 |
| XP_024573944.1 | ---------- ---------- ---------- ---------- ---------- ---------- ---------- ---------- | 582 |
| XP_024579793.1 | ---------- ---------- ---------- ---------- ---------- ---------- ---------- ---------- | 158 |
| PvCRN15 | ---------- ---------- ---------- ---------- ---------- ---------- ---------- ---------- | 290 |
| PvCRN26 | ---------- ---------- ---------- ---------- ---------- ---------- ---------- ---------- | 279 |
| PvCRN16 | ---------- ---------- ---------- ---------- ---------- ---------- ---------- ---------- | 365 |
| PvCRN23 | ---------- ---------- ---------- ---------- ---------- ---------- ---------- ---------- | 426 |
| XP_024573063.1 | ---------- ---------- ---------- ---------- ---------- ---------- ---------- ---------- | 83 |
| PvCRN19 | ---------- ---------- ---------- ---------- ---------- ---------- ---------- ---------- | 164 |
| XP_024581625.1 | ---------- ---------- ---------- ---------- ---------- ---------- ---------- ---------- | 135 |
| XP_024583886.1 | ---------- ---------- ---------- ---------- ---------- ---------- ---------- ---------- | 96 |
| XP_024584409.1 | ---------- ---------- ---------- ---------- ---------- ---------- ---------- ---------- | 213 |
| XP_024572385.1 | ---------- ---------- ---------- ---------- ---------- ---------- ---------- ---------- | 310 |
| PvCRN6 | ---------- ---------- ---------- ---------- ---------- ---------- ---------- ---------- | 372 |
| PvCRN9 | ---------- ---------- ---------- ---------- ---------- ---------- ---------- ---------- | 611 |
| XP_024580875.1 | ---------- ---------- ---------- ---------- ---------- ---------- ---------- ---------- | 127 |
| PvCRN24 | ---------- ---------- ---------- ---------- ---------- ---------- ---------- ---------- | 165 |
| PvCRN35 | ---------- ---------- ---------- ---------- ---------- ---------- ---------- ---------- | 212 |
| PvCRN12 | ---------- ---------- ---------- ---------- ---------- ---------- ---------- ---------- | 171 |
| XP_024576927.1 | ---------- ---------- ---------- ---------- ---------- ---------- ---------- ---------- | 528 |
| XP_024578927.1 | ---------- ---------- ---------- ---------- ---------- ---------- ---------- ---------- | 327 |
| XP_024586212.1 | ---------- ---------- ---------- ---------- ---------- ---------- ---------- ---------- | 616 |
| XP_024578078.1 | ---------- ---------- ---------- ---------- ---------- ---------- ---------- ---------- | 262 |
| XP_024575355.1 | ---------- ---------- ---------- ---------- ---------- ---------- ---------- ---------- | 295 |
| XP_024584527.1 | ---------- ---------- ---------- ---------- ---------- ---------- ---------- ---------- | 168 |
| XP_024586664.1 | ---------- ---------- ---------- ---------- ---------- ---------- ---------- ---------- | 357 |
| PvCRN17 | ---------- ---------- ---------- ---------- ---------- ---------- ---------- ---------- | 187 |
| PvCRN2 | ---------- ---------- ---------- ---------- ---------- ---------- ---------- ---------- | 164 |
| XP_024577181.1 | ---------- ---------- ---------- ---------- ---------- ---------- ---------- ---------- | 207 |
| XP_024581075.1 | ---------- ---------- ---------- ---------- ---------- ---------- ---------- ---------- | 156 |
| XP_024575884.1 | ---------- ---------- ---------- ---------- ---------- ---------- ---------- ---------- | 155 |
| XP_024586872.1 | ---------- ---------- ---------- ---------- ---------- ---------- ---------- ---------- | 182 |
| XP_024573052.1 | ---------- ---------- ---------- ---------- ---------- ---------- ---------- ---------- | 187 |
| XP_024586054.1 | ---------- ---------- ---------- ---------- ---------- ---------- ---------- ---------- | 237 |
| PvCRN27 | ---------- ---------- ---------- ---------- ---------- ---------- ---------- ---------- | 313 |
| PvCRN20 | ---------- ---------- ---------- ---------- ---------- ---------- ---------- ---------- | 159 |
| PvCRN25 | ---------- ---------- ---------- ---------- ---------- ---------- ---------- ---------- | 194 |
| PvCRN1 | ---------- ---------- ---------- ---------- ---------- ---------- ---------- ---------- | 580 |
| PvCRN4 | ---------- ---------- ---------- ---------- ---------- ---------- ---------- ---------- | 583 |
| PvCRN30 | ---------- ---------- ---------- ---------- ---------- ---------- ---------- ---------- | 220 |
| PvCRN29 | ---------- ---------- ---------- ---------- ---------- ---------- ---------- ---------- | 312 |
| XP_024583036.1 | ---------- ---------- ---------- ---------- ---------- ---------- ---------- ---------- | 681 |
| PvCRN10 | ---------- ---------- ---------- ---------- ---------- ---------- ---------- ---------- | 217 |
| XP_024577280.1 | ---------- ---------- ---------- ---------- ---------- ---------- ---------- ---------- | 283 |
| XP_024572924.1 | ---------- ---------- ---------- ---------- ---------- ---------- ---------- ---------- | 412 |
| PvCRN14 | ---------- ---------- ---------- ---------- ---------- ---------- ---------- ---------- | 151 |
| XP_024577521.1 | ---------- ---------- ---------- ---------- ---------- ---------- ---------- ---------- | 108 |
| PvCRN21 | ---------- ---------- ---------- ---------- ---------- ---------- ---------- ---------- | 479 |
| XP_024574966.1 | ---------- ---------- ---------- ---------- ---------- ---------- ---------- ---------- | 155 |
| XP_024575372.1 | ---------- ---------- ---------- ---------- ---------- ---------- ---------- ---------- | 188 |
| XP_024581363.1 | ---------- ---------- ---------- ---------- ---------- ---------- ---------- ---------- | 229 |
| XP_024583883.1 | ---------- ---------- ---------- ---------- ---------- ---------- ---------- ---------- | 381 |
| XP_024579844.1 | ---------- ---------- ---------- ---------- ---------- ---------- ---------- ---------- | 476 |
| XP_024574916.1 | ---------- ---------- ---------- ---------- ---------- ---------- ---------- ---------- | 527 |
| XP_024574193.1 | ---------- ---------- ---------- ---------- ---------- ---------- ---------- ---------- | 615 |
| XP_024583155.1 | ---------- ---------- ---------- ---------- ---------- ---------- ---------- ---------- | 419 |
| XP_024574913.1 | ---------- ---------- ---------- ---------- ---------- ---------- ---------- ---------- | 623 |
| XP_024578158.1 | ---------- ---------- ---------- ---------- ---------- ---------- ---------- ---------- | 106 |
| PvCRN22 | ---------- ---------- ---------- ---------- ---------- ---------- ---------- ---------- | 206 |
| XP_024585001.1 | ---------- ---------- ---------- ---------- ---------- ---------- ---------- ---------- | 127 |
| XP_024583843.1 | ---------- ---------- ---------- ---------- ---------- ---------- ---------- ---------- | 534 |
| XP_024578702.1 | ---------- ---------- ---------- ---------- ---------- ---------- ---------- ---------- | 299 |
| XP_024585805.1 | ---------- ---------- ---------- ---------- ---------- ---------- ---------- ---------- | 334 |
| XP_024580932.1 | ---------- ---------- ---------- ---------- ---------- ---------- ---------- ---------- | 508 |
| Consensus | ---------- ---------- ---------- ---------- ---------- ---------- ---------- ---------- | |
| Conservation (100% / 0%) | | |

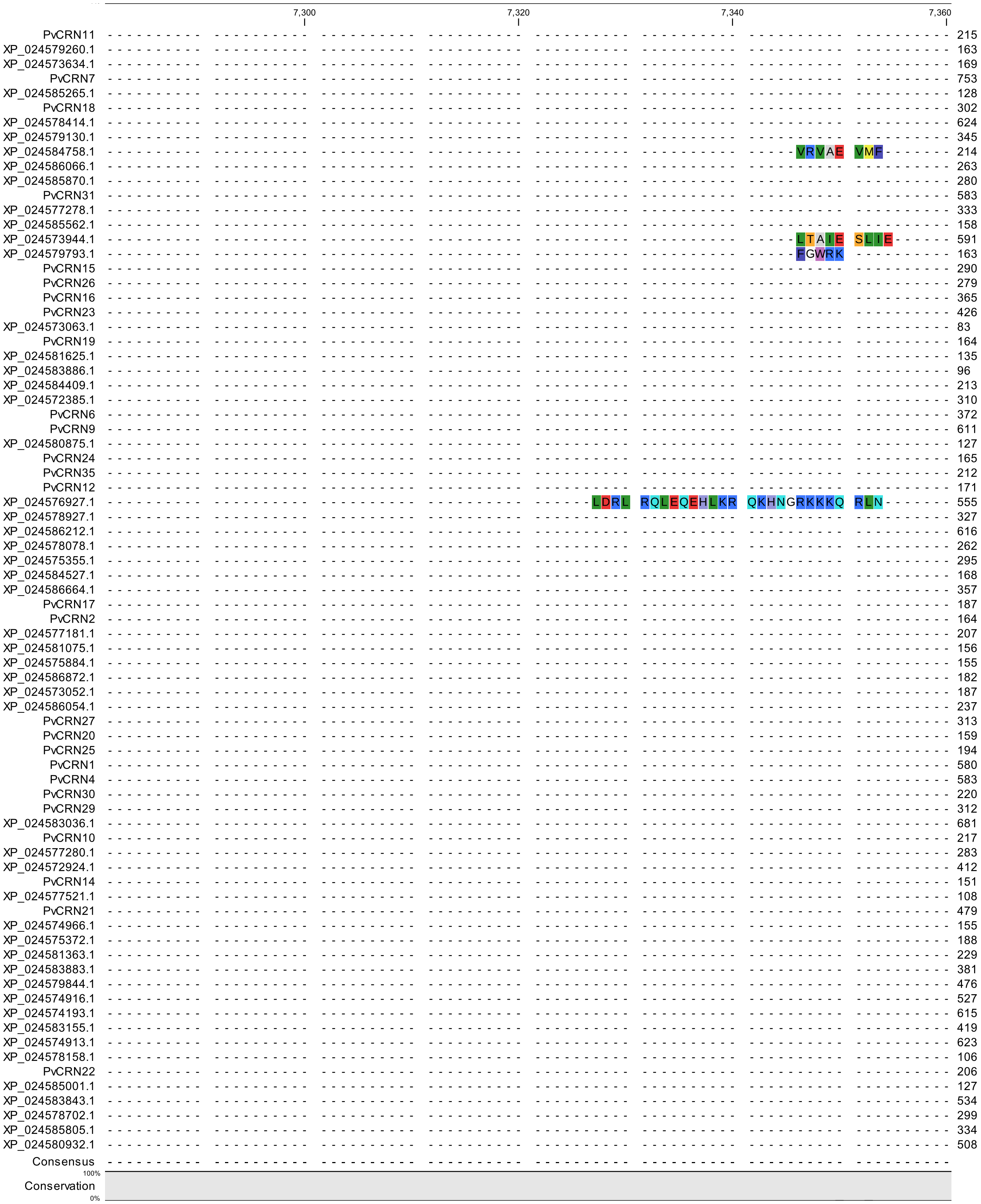
7,300
7,320
7,340
7,360
PvCRN11 215
XP_024579260.1 163
XP_024573634.1 169
PvCRN7 753
XP_024585265.1 128
PvCRN18 302
XP_024578414.1 624
XP_024579130.1 345
XP_024584758.1 VRVAE VMF 214
XP_024586066.1 263
XP_024585870.1 280
PvCRN31 583
XP_024577278.1 333
XP_024585562.1 158
XP_024573944.1 LTAIE SLIE 591
XP_024579793.1 FGWRK 163
PvCRN15 290
PvCRN26 279
PvCRN16 365
PvCRN23 426
XP_024573063.1 83
PvCRN19 164
XP_024581625.1 135
XP_024583886.1 96
XP_024584409.1 213
XP_024572385.1 310
PvCRN6 372
PvCRN9 611
XP_024580875.1 127
PvCRN24 165
PvCRN35 212
PvCRN12 171
XP_024576927.1 LDRL RQLEQEHLKR QKHNGRKKKQ RLN 555
XP_024578927.1 327
XP_024586212.1 616
XP_024578078.1 262
XP_024575355.1 295
XP_024584527.1 168
XP_024586664.1 357
PvCRN17 187
PvCRN2 164
XP_024577181.1 207
XP_024581075.1 156
XP_024575884.1 155
XP_024586872.1 182
XP_024573052.1 187
XP_024586054.1 237
PvCRN27 313
PvCRN20 159
PvCRN25 194
PvCRN1 580
PvCRN4 583
PvCRN30 220
PvCRN29 312
XP_024583036.1 681
PvCRN10 217
XP_024577280.1 283
XP_024572924.1 412
PvCRN14 151
XP_024577521.1 108
PvCRN21 479
XP_024574966.1 155
XP_024575372.1 188
XP_024581363.1 229
XP_024583883.1 381
XP_024579844.1 476
XP_024574916.1 527
XP_024574193.1 615
XP_024583155.1 419
XP_024574913.1 623
XP_024578158.1 106
PvCRN22 206
XP_024585001.1 127
XP_024583843.1 534
XP_024578702.1 299
XP_024585805.1 334
XP_024580932.1 508
Consensus
100%
Conservation
0%

| | 7,380 | 7,400 | 7,420 | 7,440 |
|---|---|---|---|---|

| Sequence | Alignment | |
|---|---|---|
| PvCRN11 | ---------- ---------- ---------- ---------- ---------- ---------- ---------- ---------- | 215 |
| XP_024579260.1 | ---------- ---------- ---------- ---------- ---------- ---------- ---------- ---------- | 163 |
| XP_024573634.1 | ---------- ---------- ---------- ---------- ---------- ---------- ---------- ---------- | 169 |
| PvCRN7 | ---------- ---------- ---------- ---------- ---------- ---------- ---------- ---------- | 753 |
| XP_024585265.1 | ---------- ---------- ---------- ---------- ---------- ---------- ---------- ---------- | 128 |
| PvCRN18 | ---------- ---------- ---------- ---------- ---------- ---------- ---------- ---------- | 302 |
| XP_024578414.1 | ---------- ---------- ---------- ---------- ---------- ---------- ---------- ---------- | 624 |
| XP_024579130.1 | ---------- ---------- ---------- ---------- ---------- ---------- ---------- ---------- | 345 |
| XP_024584758.1 | ---------- ---------- ---------- ---------- ---------- ---------- ---------- ---------- | 214 |
| XP_024586066.1 | ---------- ---------- ---------- ---------- ---------- ---------- ---------- ---------- | 263 |
| XP_024585870.1 | ---------- ---------- ---------- ---------- ---------- ---------- ---------- ---------- | 280 |
| PvCRN31 | ---------- ---------- ---------- ---------- ---------- ---------- ---------- ---------- | 583 |
| XP_024577278.1 | ---------- ---------- ---------- ---------- ---------- ---------- ---------- ---------- | 333 |
| XP_024585562.1 | ---------- ---------- ---------- ---------- ---------- ---------- ---------- ---------- | 158 |
| XP_024573944.1 | ---------- -S-------- ---------- ---------- ---------- ---------- ---------- ---------- | 592 |
| XP_024579793.1 | ---------- ---------- ---------- ---------- ---------- ---------- ---------- ---------- | 163 |
| PvCRN15 | ---------- ---------- ---------- ---------- ---------- ---------- ---------- ---------- | 290 |
| PvCRN26 | ---------- ---------- ---------- ---------- ---------- ---------- ---------- ---------- | 279 |
| PvCRN16 | ---------- ---------- ---------- ---------- ---------- ---------- ---------- ---------- | 365 |
| PvCRN23 | ---------- ---------- ---------- ---------- ---------- ---------- ---------- ---------- | 426 |
| XP_024573063.1 | ---------- ---------- ---------- ---------- ---------- ---------- ---------- ---------- | 83 |
| PvCRN19 | ---------- ---------- ---------- ---------- ---------- ---------- ---------- ---------- | 164 |
| XP_024581625.1 | ---------- ---------- ---------- ---------- ---------- ---------- ---------- ---------- | 135 |
| XP_024583886.1 | ---------- ---------- ---------- ---------- ---------- ---------- ---------- ---------- | 96 |
| XP_024584409.1 | ---------- ---------- ---------- ---------- ---------- ---------- ---------- ---------- | 213 |
| XP_024572385.1 | ---------- ---------- ---------- ---------- ---------- ---------- ---------- ---------- | 310 |
| PvCRN6 | ---------- ---------- ---------- ---------- ---------- ---------- ---------- ---------- | 372 |
| PvCRN9 | ---------- ---------- ---------- ---------- ---------- ---------- ---------- ---------- | 611 |
| XP_024580875.1 | ---------- ---------- ---------- ---------- ---------- ---------- ---------- ---------- | 127 |
| PvCRN24 | ---------- ---------- ---------- ---------- ---------- ---------- ---------- ---------- | 165 |
| PvCRN35 | ---------- ---------- ---------- ---------- ---------- ---------- ---------- ---------- | 212 |
| PvCRN12 | ---------- ---------- ---------- ---------- ---------- ---------- ---------- ---------- | 171 |
| XP_024576927.1 | ---------- ---------- ---------- ---------- ---------- ---------- ---------- ---------- | 555 |
| XP_024578927.1 | ---------- ---------- ---------- ---------- ---------- ---------- ---------- ---------- | 327 |
| XP_024586212.1 | ---------- ---------- ---------- ---------- ---------- ---------- ---------- ---------- | 616 |
| XP_024578078.1 | ---------- ---------- ---------- ---------- ---------- ---------- ---------- ---------- | 262 |
| XP_024575355.1 | ---------- ---------- ---------- ---------- ---------- ---------- ---------- ---------- | 295 |
| XP_024584527.1 | ---------- ---------- ---------- ---------- ---------- ---------- ---------- ---------- | 168 |
| XP_024586664.1 | ---------- ---------- ---------- ---------- ---------- ---------- ---------- ---------- | 357 |
| PvCRN17 | ---------- ---------- ---------- ---------- ---------- ---------- ---------- ---------- | 187 |
| PvCRN2 | ---------- ---------- ---------- ---------- ---------- ---------- ---------- ---------- | 164 |
| XP_024577181.1 | ---------- ---------- ---------- ---------- ---------- ---------- ---------- ---------- | 207 |
| XP_024581075.1 | ---------- ---------- ---------- ---------- ---------- ---------- ---------- ---------- | 156 |
| XP_024575884.1 | ---------- ---------- ---------- ---------- ---------- ---------- ---------- ---------- | 155 |
| XP_024586872.1 | ---------- ---------- ---------- ---------- ---------- ---------- ---------- ---------- | 182 |
| XP_024573052.1 | ---------- ---------- ---------- ---------- ---------- ---------- ---------- ---------- | 187 |
| XP_024586054.1 | ---------- ---------- ---------- ---------- ---------- ---------- ---------- ---------- | 237 |
| PvCRN27 | ---------- ---------- ---------- ---------- ---------- ---------- ---------- ---------- | 313 |
| PvCRN20 | ---------- ---------- ---------- ---------- ---------- ---------- ---------- ---------- | 159 |
| PvCRN25 | ---------- ---------- ---------- ---------- ---------- ---------- ---------- ---------- | 194 |
| PvCRN1 | ---------- ---------- ---------- ---------- ---------- ---------- ---------- ---------- | 580 |
| PvCRN4 | ---------- ---------- ---------- ---------- ---------- ---------- ---------- ---------- | 583 |
| PvCRN30 | ---------- ---------- ---------- ---------- ---------- ---------- ---------- ---------- | 220 |
| PvCRN29 | ---------- ---------- ---------- ---------- ---------- ---------- ---------- ---------- | 312 |
| XP_024583036.1 | ---------- ---------- ---------- ---------- ---------- ---------- ---------- ---------- | 681 |
| PvCRN10 | ---------- ---------- ---------- ---------- ---------- ---------- ---------- ---------- | 217 |
| XP_024577280.1 | ---------- ---------- ---------- ---------- ---------- ---------- ---------- ---------- | 283 |
| XP_024572924.1 | ---------- ---------- ---------- ---------- ---------- ---------- ---------- ---------- | 412 |
| PvCRN14 | ---------- ---------- ---------- ---------- ---------- ---------- ---------- ---------- | 151 |
| XP_024577521.1 | ---------- ---------- ---------- ---------- ---------- ---------- ---------- ---------- | 108 |
| PvCRN21 | ---------- ---------- ---------- ---------- ---------- ---------- ---------- ---------- | 479 |
| XP_024574966.1 | ---------- ---------- ---------- ---------- ---------- ---------- ---------- ---------- | 155 |
| XP_024575372.1 | ---------- ---------- ---------- ---------- ---------- ---------- ---------- ---------- | 188 |
| XP_024581363.1 | ---------- ---------- ---------- ---------- ---------- ---------- ---------- ---------- | 229 |
| XP_024583883.1 | ---------- ---------- ---------- ---------- ---------- ---------- ---------- ---------- | 381 |
| XP_024579844.1 | ---------- ---------- ---------- ---------- ---------- ---------- ---------- ---------- | 476 |
| XP_024574916.1 | ---------- ---------- ---------- ---------- ---------- ---------- ---------- ---------- | 527 |
| XP_024574193.1 | ---------- ---------- ---------- ---------- ---------- ---------- ---------- ---------- | 615 |
| XP_024583155.1 | ---------- ---------- ---------- ---------- ---------- ---------- ---------- ---------- | 419 |
| XP_024574913.1 | ---------- ---------- ---------- ---------- ---------- ---------- ---------- ---------- | 623 |
| XP_024578158.1 | ---------- ---------- ---------- ---------- ---------- ---------- ---------- ---------- | 106 |
| PvCRN22 | ---------- ---------- ---------- ---------- ---------- ---------- ---------- ---------- | 206 |
| XP_024585001.1 | ---------- ---------- ---------- ---------- ---------- ---------- ---------- ---------- | 127 |
| XP_024583843.1 | ---------- ---------- ---------- ---------- ---------- ---------- ---------- ---------- | 534 |
| XP_024578702.1 | ---------- ---------- ---------- ---------- ---------- ---------- ---------- ---------- | 299 |
| XP_024585805.1 | ---------- ---------- ---------- ---------- ---------- ---------- ---------- ---------- | 334 |
| XP_024580932.1 | ---------- ---------- ---------- ---------- ---------- ---------- ---------- ---------- | 508 |
| Consensus | ---------- ---------- ---------- ---------- ---------- ---------- ---------- ---------- | |
| Conservation (100% / 0%) | | |

```
                                 7,460                7,480                7,500                7,520
                                     |                    |                    |                    |
PvCRN11         ---------- ---------- ---------- ---------- ---------- ---------- ---------- ---------- 215
XP_024579260.1  ---------- ---------- ---------- ---------- ---------- ---------- ---------- ---------- 163
XP_024573634.1  ---------- ---------- ---------- ---------- ---------- ---------- ---------- ---------- 169
PvCRN7          ---------- ---------- ---------- ---------- ---------- ---------- ---------- ---------- 753
XP_024585265.1  ---------- ---------- ---------- ---------- ---------- ---------- ---------- ---------- 128
PvCRN18         ---------- ---------- ---------- ---------- ---------- ---------- ---------- ---------- 302
XP_024578414.1  ---------- ---------- ---------- ---------- ---------- ---------- ---------- ---------- 624
XP_024579130.1  ---------- ---------- ---------- ---------- ---------- ---------- ---------- ---------- 345
XP_024584758.1  ---------- ---------- ---------- ---------- ---------- ---------- ---------- ---------- 214
XP_024586066.1  ---------- ---------- ---------- ---------- ---------- ---------- ---------- ---------- 263
XP_024585870.1  ---------- ---------- ---------- ---------- ---------- ---------- ---------- ---------- 280
PvCRN31         ---------- ---------- ---------- ---------- ---------- ---------- ---------- ---------- 583
XP_024577278.1  ---------- ---------- ---------- ---------- ---------- ---------- ---------- ---------- 333
XP_024585562.1  ---------- ---------- ---------- ---------- ---------- ---------- ---------- ---------- 158
XP_024573944.1  ---------- ---------- ---------- ---------- ---------- ---------- ---------- ---------- 592
XP_024579793.1  ---------- ---------- ---------- ---------- ---------- ---------- ---------- ---------- 163
PvCRN15         ---------- ---------- ---------- ---------- ---------- ---------- ---------- ---------- 290
PvCRN26         ---------- ---------- ---------- ---------- ---------- ---------- ---------- ---------- 279
PvCRN16         ---------- ---------- ---------- ---------- ---------- ---------- ---------- ---------- 365
PvCRN23         ---------- ---------- ---------- ---------- ---------- ---------- ---------- ---------- 426
XP_024573063.1  ---------- ---------- ---------- ---------- ---------- ---------- ---------- ---------- 83
PvCRN19         ---------- ---------- ---------- ---------- ---------- ---------- ---------- ---------- 164
XP_024581625.1  ---------- ---------- ---------- ---------- ---------- ---------- ---------- ---------- 135
XP_024583886.1  ---------- ---------- ---------- ---------- ---------- ---------- ---------- ---------- 96
XP_024584409.1  ---------- ---------- ---------- ---------- ---------- ---------- ---------- ---------- 213
XP_024572385.1  ---------- ---------- ---------- ---------- ---------- ---------- ---------- ---------- 310
PvCRN6          ---------- ---------- ---------- ---------- ---------- ---------- ---------- ---------- 372
PvCRN9          ---------- ---------- ---------- ---------- ---------- ---------- ---------- ---------- 611
XP_024580875.1  ---------- ---------- ---------- ---------- ---------- ---------- ---------- ---------- 127
PvCRN24         ---------- ---------- ---------- ---------- ---------- ---------- ---------- ---------- 165
PvCRN35         ---------- ---------- ---------- ---------- ---------- ---------- ---------- ---------- 212
PvCRN12         ---------- ---------- ---------- ---------- ---------- ---------- ---------- ---------- 171
XP_024576927.1  ---------- ---------- ---------- ---------- ---------- ---------- ---------- ---------- 555
XP_024578927.1  ---------- ---------- ---------- ---------- ---------- ---------- ---------- ---------- 327
XP_024586212.1  ---------- ---------- ---------- ---------- ---------- ---------- ---------- ---------- 616
XP_024578078.1  ---------- ---------- ---------- ---------- ---------- ---------- ---------- ---------- 262
XP_024575355.1  ---------- ---------- ---------- ---------- ---------- ---------- ---------- ---------- 295
XP_024584527.1  ---------- ---------- ---------- ---------- ---------- ---------- ---------- ---------- 168
XP_024586664.1  ---------- ---------- ---------- ---------- ---------- ---------- ---------- ---------- 357
PvCRN17         ---------- ---------- ---------- ---------- ---------- ---------- ---------- ---------- 187
PvCRN2          ---------- ---------- ---------- ---------- ---------- ---------- ---------- -ILYQ----- 168
XP_024577181.1  ---------- ---------- ---------- ---------- ---------- ---------- ---------- ---------- 207
XP_024581075.1  ---------- ---------- ---------- ---------- ---------- ---------- ---------- ---------- 156
XP_024575884.1  ---------- ---------- ---------- ---------- ---------- ---------- ---------- ---------- 155
XP_024586872.1  ---------- ---------- ---------- ---------- ---------- ---------- ---------- ---------- 182
XP_024573052.1  ---------- ---------- ---------- ---------- ---------- ---------- ---------- ---------- 187
XP_024586054.1  ---------- ---------- ---------- ---------- ---------- ---------- ---------- ---------- 237
PvCRN27         ---------- ---------- ---------- ---------- ---------- ---------- ---------- ---------- 313
PvCRN20         ---------- ---------- ---------- ---------- ---------- ---------- ---------- ---------- 159
PvCRN25         ---------- ---------- ---------- ---------- ---------- ---------- ---------- ---------- 194
PvCRN1          ---------- ---------- ---------- ---------- ---------- ---------- ---------- ---------- 580
PvCRN4          ---------- ---------- ---------- ---------- ---------- ---------- ---------- ---------- 583
PvCRN30         ---------- ---------- ---------- ---------- ---------- ---------- ---------- ---------- 220
PvCRN29         ---------- ---------- ---------- ---------- ---------- ---------- ---------- ---------- 312
XP_024583036.1  ---------- ---------- ---------- ---------- ---------- ---------- ---------- ---------- 681
PvCRN10         ---------- ---------- ---------- ---------- ---------- ---------- ---------- ---------- 217
XP_024577280.1  ---------- ---------- ---------- ---------- ---------- ---------- ---------- ---------- 283
XP_024572924.1  ---------- ---------- ---------- ---------- ---------- ---------- ---------- ---------- 412
PvCRN14         ---------- ---------- ---------- ---------- ---------- ---------- ---------- ---------- 151
XP_024577521.1  ---------- ---------- ---------- ---------- ---------- ---------- ---------- ---------- 108
PvCRN21         ---------- ---------- ---------- ---------- ---------- ---------- ---------- ---------- 479
XP_024574966.1  ---------- ---------- ---------- ---------- ---------- ---------- ---------- ---------- 155
XP_024575372.1  ---------- ---------- ---------- ---------- ---------- ---------- ---------- ---------- 188
XP_024581363.1  ---------- ---------- ---------- ---------- ---------- ---------- ---------- ---------- 229
XP_024583883.1  ---------- ---------- ---------- ---------- ---------- ---------- ---------- ---------- 381
XP_024579844.1  ---------- ---------- ---------- ---------- ---------- ---------- ---------- ---------- 476
XP_024574916.1  ---------- ---------- ---------- ---------- ---------- ---------- ---------- ---------- 527
XP_024574193.1  ---------- ---------- ---------- ---------- ---------- ---------- ---------- ---------- 615
XP_024583155.1  ---------- ---------- ---------- ---------- ---------- ---------- ---------- ---------- 419
XP_024574913.1  ---------- ---------- ---------- ---------- ---------- ---------- ---------- ---------- 623
XP_024578158.1  ---------- ---------- ---------- ---------- ---------- ---------- ---------- ---------- 106
PvCRN22         ---------- ---------- ---------- ---------- ---------- ---------- ---------- ---------- 206
XP_024585001.1  ---------- ---------- ---------- ---------- ---------- ---------- ---------- ---------- 127
XP_024583843.1  ---------- ---------- ---------- ---------- ---------- ---------- ---------- ---------- 534
XP_024578702.1  ---------- ---------- ---------- ---------- ---------- ---------- ---------- ---------- 299
XP_024585805.1  ---------- ---------- ---------- ---------- ---------- ---------- ---------- ---------- 334
XP_024580932.1  ---------- ---------- ---------- ---------- ---------- ---------- ---------- ---------- 508
Consensus       ---------- ---------- ---------- ---------- ---------- ---------- ---------- ----------
           100%
Conservation
             0%
```

```
                                    7,540                7,560                7,580                7,600
PvCRN11         ---------- ---------- ---------- ---------- ---------- ---------- ---------- ---------- 215
XP_024579260.1  ---------- ---------- ---------- ---------- ---------- ---------- ---------- ---------- 163
XP_024573634.1  ---------- ---------- ---------- ---------- ---------- ---------- ---------- ---------- 169
PvCRN7          ---------- ---------- ---------- ---------- ---------- ---------- ---------- ---------- 753
XP_024585265.1  ---------- ---------- ---------- ---------- ---------- ---------- ---------- ---------- 128
PvCRN18         ---------- ---------- ---------- ---------- ---------- ---------- ---------- ---------- 302
XP_024578414.1  ---------- ---------- ---------- ---------- ---------- ---------- ---------- ---------- 624
XP_024579130.1  ---------- ---------- ---------- ---------- ---------- ---------- ---------- ---------- 345
XP_024584758.1  ---------- ---------- ---------- ---------- ---------- ---------- ---------- ---------- 214
XP_024586066.1  ---------- ---------- ---------- ---------- ---------- ---------- ---------- ---------- 263
XP_024585870.1  ---------- ---------- ---------- ---------- ---------- ---------- ---------- ---------- 280
PvCRN31         ---------- ---------- ---------- ---------- ---------- ---------- ---------- ---------- 583
XP_024577278.1  ---------- ---------- ---------- ---------- ---------- ---------- ---------- ---------- 333
XP_024585562.1  ---------- ---------- ---------- ---------- ---------- ---------- ---------- ---------- 158
XP_024573944.1  ---------- ---------- ---------- ---------- ---------- ---------- ---------- ---------- 592
XP_024579793.1  ---------- ---------- ---------- ---------- ---------- ---------- ---------- ---------- 163
PvCRN15         ---------- ---------- ---------- ---------- ---------- ---------- ---------- ---------- 290
PvCRN26         ---------- ---------- ---------- ---------- ---------- ---------- ---------- ---------- 279
PvCRN16         ---------- ---------- ---------- ---------- ---------- ---------- ---------- ---------- 365
PvCRN23         ---------- ---------- ---------- ---------- ---------- ---------- ---------- ---------- 426
XP_024573063.1  ---------- ---------- ---------- ---------- ---------- ---------- ---------- ---------- 83
PvCRN19         ---------- ---------- ---------- ---------- ---------- ---------- ---------- ---------- 164
XP_024581625.1  ---------- ---------- ---------- ---------- ---------- ---------- ---------- ---------- 135
XP_024583886.1  ---------- ---------- ---------- ---------- ---------- ---------- ---------- ---------- 96
XP_024584409.1  ---------- ---------- ---------- ---------- ---------- ---------- ---------- ---------- 213
XP_024572385.1  ---------- ---------- ---------- ---------- ---------- ---------- ---------- ---------- 310
PvCRN6          ---------- ---------- ---------- ---------- ---------- ---------- ---------- ---------- 372
PvCRN9          ---------- ---------- ---------- ---------- ---------- ---------- ---------- ---------- 611
XP_024580875.1  ---------- ---------- ---------- ---------- ---------- ---------- ---------- ---------- 127
PvCRN24         ---------- ---------- ---------- ---------- ---------- ---------- ---------- ---------- 165
PvCRN35         ---------- ---------- ---------- ---------- ---------- ---------- ---------- ---------- 212
PvCRN12         ---------- ---------- ---------- ---------- ---------- ---------- ---------- ---------- 171
XP_024576927.1  ---------- ---------- ---------- ---------- ---------- ---------- ---------- ---------- 555
XP_024578927.1  ---------- ---------- ---------- ---------- ---------- ---------- ---------- ---------- 327
XP_024586212.1  ---------- ---------- ---------- ---------- ---------- ---------- ---------- ---------- 616
XP_024578078.1  ---------- ---------- ---------- ---------- ---------- ---------- ---------- ---------- 262
XP_024575355.1  ---------- ---------- ---------- ---------- ---------- ---------- ---------- ---------- 295
XP_024584527.1  ---------- ---------- ---------- ---------- ---------- ---------- ---------- ---------- 168
XP_024586664.1  ---------- ---------- ---------- ---------- ---------- ---------- ---------- ---------- 357
PvCRN17         ---------- ---------- ---------- ---------- ---------- ---------- ---------- ---------- 187
PvCRN2          ---------- ---------- ---------- ---------- ---------- ---------- ---------- ---------- 168
XP_024577181.1  ---------- ---------- ---------- ---------- ---------- ---------- ---------- ---------- 207
XP_024581075.1  ---------- ---------- ---------- ---------- ---------- ---------- ---------- ---------- 156
XP_024575884.1  ---------- ---------- ---------- ---------- ---------- ---------- ---------- ---------- 155
XP_024586872.1  ---------- ---------- ---------- ---------- ---------- ---------- ---------- ---------- 182
XP_024573052.1  ---------- ---------- ---------- ---------- ---------- ---------- ---------- ---------- 187
XP_024586054.1  ---------- ---------- ---------- ---------- ---------- ---------- ---------- ---------- 237
PvCRN27         ---------- ---------- ---------- ---------- ---------- ---------- ---------- ---------- 313
PvCRN20         ---------- ---------- ---------- ---------- ---------- ---------- ---------- ---------- 159
PvCRN25         ---------- ---------- ---------- ---------- ---------- ---------- ---------- ---------- 194
PvCRN1          ---------- ---------- ---------- ---------- ---------- ---------- ---------- ---------- 580
PvCRN4          ---------- ---------- ---------- ---------- ---------- ---------- ---------- ---------- 583
PvCRN30         ---------- ---------- ---------- ---------- ---------- ---------- ---------- ---------- 220
PvCRN29         ---------- ---------- ---------- ---------- ---------- ---------- ---------- ---------- 312
XP_024583036.1  ---------- ---------- ---------- ---------- ---------- ---------- ---------- ---------- 681
PvCRN10         ---------- ---------- ---------- ---------- ---------- ---------- ---------- ---------- 217
XP_024577280.1  ---------- ---------- ---------- ---------- ---------- ---------- ---------- ---------- 283
XP_024572924.1  ---------- ---------- ---------- ---------- ---------- ---------- ---------- ---------- 412
PvCRN14         ---------- ---------- ---------- ---------- ---------- ---------- ---------- ---------- 151
XP_024577521.1  ---------- ---------- ---------- ---------- ---------- ---------- ---------- ---------- 108
PvCRN21         ---------- ---------- ---------- ---------- ---------- ---------- ---------- ---------- 479
XP_024574966.1  ---------- ---------- ---------- ---------- ---------- ---------- ---------- ---------- 155
XP_024575372.1  ---------- ---------- ---------- ---------- ---------- ---------- ---------- ---------- 188
XP_024581363.1  ---------- ---------- ---------- ---------- ---------- ---------- ---------- ---------- 229
XP_024583883.1  ---------- ---------- ---------- ---------- ---------- ---------- ---------- ---------- 381
XP_024579844.1  ---------- ---------- ---------- ---------- ---------- ---------- ---------- ---------- 476
XP_024574916.1  ---------- ---------- ---------- ---------- ---------- ---------- ---------- ---------- 527
XP_024574193.1  ---------- ---------- ---------- ---------- ---------- ---------- ---------- ---------- 615
XP_024583155.1  ---------- ---------- ---------- ---------- ---------- ---------- ---------- ---------- 419
XP_024574913.1  ---------- ---------- ---------- ---------- ---------- ---------- ---------- ---------- 623
XP_024578158.1  ---------- ---------- ---------- ---------- ---------- ---------- ---------- ---------- 106
PvCRN22         ---------- ---------- ---------- ---------- ---------- ---------- ---------- ---------- 206
XP_024585001.1  ---------- ---------- ---------- ---------- ---------- ---------- ---------- ---------- 127
XP_024583843.1  ---------- ---------- ---------- ---------- ---------- ---------- ---------- ---------- 534
XP_024578702.1  ---------- ---------- ---------- ---------- ---------- ---------- ---------- ---------- 299
XP_024585805.1  ---------- ---------- ---------- ---------- ---------- ---------- ---------- ---------- 334
XP_024580932.1  ---------- ---------- ---------- ---------- ---------- ---------- ---------- ---------- 508
Consensus       ---------- ---------- ---------- ---------- ---------- ---------- ---------- ----------
100%
Conservation
0%
```

| | 7,620 7,640 7,660 7,680 | |
|---|---|---|
| PvCRN11 | ---------- ---------- ---------- ---------- ---------- ---------- ---------- ---------- | 215 |
| XP_024579260.1 | ---------- ---------- ---------- ---------- ---------- ---------- ---------- ---------- | 163 |
| XP_024573634.1 | ---------- ---------- ---------- ---------- ---------- ---------- ---------- ---------- | 169 |
| PvCRN7 | ---------- ---------- ---------- ---------- ---------- ---------- ---------- ---------- | 753 |
| XP_024585265.1 | ---------- ---------- ---------- ---------- ---------- ---------- ---------- ---------- | 128 |
| PvCRN18 | ---------- ---------- ---------- ---------- ---------- ---------- ---------- ---------- | 302 |
| XP_024578414.1 | ---------- ---------- ---------- ---------- ---------- ---------- ---------- ---------- | 624 |
| XP_024579130.1 | ---------- ---------- ---------- ---------- ---------- ---------- ---------- ---------- | 345 |
| XP_024584758.1 | ---------- ---------- ---------- ---------- ---------- ---------- ---------- ---------- | 214 |
| XP_024586066.1 | ---------- ---------- ---------- ---------- ---------- ---------- ---------- ---------- | 263 |
| XP_024585870.1 | ---------- ---------- ---------- ---------- ---------- ---------- ---------- ---------- | 280 |
| PvCRN31 | ---------- ---------- ---------- ---------- ---------- ---------- ---------- ---------- | 583 |
| XP_024577278.1 | ---------- ---------- ---------- ---------- ---------- ---------- ---------- ---------- | 333 |
| XP_024585562.1 | ---------- ---------- ---------- ---------- ---------- ---------- ---------- ---------- | 158 |
| XP_024573944.1 | ---------- ---------- ---------- ---------- ---------- ---------- ---------- ---------- | 592 |
| XP_024579793.1 | ---------- ---------- ---------- ---------- ---------- ---------- ---------- ---------- | 163 |
| PvCRN15 | ---------- ---------- ---------- ---------- ---------- ---------- ---------- ---------- | 290 |
| PvCRN26 | ---------- ---------- ---------- ---------- ---------- ---------- ---------- ---------- | 279 |
| PvCRN16 | ---------- ---------- ---------- ---------- ---------- ---------- ---------- ---------- | 365 |
| PvCRN23 | ---------- ---------- ---------- ---------- ---------- ---------- ---------- ---------- | 426 |
| XP_024573063.1 | ---------- ---------- ---------- ---------- ---------- ---------- ---------- ---------- | 83 |
| PvCRN19 | ---------- ---------- ---------- ---------- ---------- ---------- ---------- ---------- | 164 |
| XP_024581625.1 | ---------- ---------- ---------- ---------- ---------- ---------- ---------- ---------- | 135 |
| XP_024583886.1 | ---------- ---------- ---------- ---------- ---------- ---------- ---------- ---------- | 96 |
| XP_024584409.1 | ---------- ---------- ---------- ---------- ---------- ---------- ---------- ---------- | 213 |
| XP_024572385.1 | ---------- ---------- ---------- ---------- ---------- ---------- ---------- ---------- | 310 |
| PvCRN6 | ---------- ---------- ---------- ---------- ---------- ---------- ---------- ---------- | 372 |
| PvCRN9 | ---------- ---------- ---------- ---------- ---------- ---------- ---------- ---------- | 611 |
| XP_024580875.1 | ---------- ---------- ---------- ---------- ---------- ---------- ---------- ---------- | 127 |
| PvCRN24 | ---------- ---------- ---------- ---------- ---------- ---------- ---------- ---------- | 165 |
| PvCRN35 | ---------- ---------- ---------- ---------- ---------- ---------- ---------- ---------- | 212 |
| PvCRN12 | ---------- ---------- ---------- ---------- ---------- ---------- ---------- ---------- | 171 |
| XP_024576927.1 | ---------- ---------- ---------- ---------- ---------- ---------- ---------- ---------- | 555 |
| XP_024578927.1 | ---------- ---------- ---------- ---------- ---------- ---------- ---------- ---------- | 327 |
| XP_024586212.1 | ---------- ---------- ---------- ---------- ---------- ---------- ---------- ---------- | 616 |
| XP_024578078.1 | ---------- ---------- ---------- ---------- ---------- ---------- ---------- ---------- | 262 |
| XP_024575355.1 | ---------- ---------- ---------- ---------- ---------- ---------- ---------- ---------- | 295 |
| XP_024584527.1 | ---------- ---------- ---------- ---------- ---------- ---------- ---------- ---------- | 168 |
| XP_024586664.1 | ---------- ---------- ---------- ---------- ---------- ---------- ---------- ---------- | 357 |
| PvCRN17 | ---------- ---------- ---------- ---------- ---------- ---------- ---------- ---------- | 187 |
| PvCRN2 | ---------- ---------- ---------- ---------- ---------- ---------- ---------- ---------- | 168 |
| XP_024577181.1 | ---------- ---------- ---------- ---------- ---------- ---------- ---------- ---------- | 207 |
| XP_024581075.1 | ---------- ---------- ---------- ---------- ---------- ---------- ---------- ---------- | 156 |
| XP_024575884.1 | ---------- ---------- ---------- ---------- ---------- ---------- ---------- ---------- | 155 |
| XP_024586872.1 | ---------- ---------- ---------- ---------- ---------- ---------- ---------- ---------- | 182 |
| XP_024573052.1 | ---------- ---------- ---------- ---------- ---------- ---------- ---------- ---------- | 187 |
| XP_024586054.1 | ---------- ---------- ---------- ---------- ---------- ---------- ---------- ---------- | 237 |
| PvCRN27 | ---------- ---------- ---------- ---------- ---------- ---------- ---------- ---------- | 313 |
| PvCRN20 | ---------- ---------- ---------- ---------- ---------- ---------- ---------- ---------- | 159 |
| PvCRN25 | ---------- ---------- ---------- ---------- ---------- ---------- ---------- ---------- | 194 |
| PvCRN1 | ---------- ---------- ---------- ---------- ---------- ---------- ---------- ---------- | 580 |
| PvCRN4 | ---------- ---------- ---------- ---------- ---------- ---------- ---------- ---------- | 583 |
| PvCRN30 | ---------- ---------- ---------- ---------- ---------- ---------- ---------- ---------- | 220 |
| PvCRN29 | ---------- ---------- ---------- ---------- ---------- ---------- ---------- ---------- | 312 |
| XP_024583036.1 | ---------- ---------- ---------- ---------- ---------- ---------- ---------- ---------- | 681 |
| PvCRN10 | ---------- ---------- ---------- ---------- ---------- ---------- ---------- ---------- | 217 |
| XP_024577280.1 | ---------- ---------- ---------- ---------- ---------- ---------- ---------- ---------- | 283 |
| XP_024572924.1 | ---------- ---------- ---------- ---------- ---------- ---------- ---------- ---------- | 412 |
| PvCRN14 | ---------- ---------- ---------- ---------- ---------- ---------- ---------- ---------- | 151 |
| XP_024577521.1 | ---------- ---------- ---------- ---------- ---------- ---------- ---------- ---------- | 108 |
| PvCRN21 | ---------- ---------- ---------- ---------- ---------- ---------- ---------- ---------- | 479 |
| XP_024574966.1 | ---------- ---------- ---------- ---------- ---------- ---------- ---------- ---------- | 155 |
| XP_024575372.1 | ---------- ---------- ---------- ---------- ---------- ---------- ---------- ---------- | 188 |
| XP_024581363.1 | ---------- ---------- ---------- ---------- ---------- ---------- ---------- ---------- | 229 |
| XP_024583883.1 | ---------- ---------- ---------- ---------- ---------- ---------- ---------- ---------- | 381 |
| XP_024579844.1 | ---------- ---------- ---------- ---------- ---------- ---------- ---------- ---------- | 476 |
| XP_024574916.1 | ---------- ---------- ---------- ---------- ---------- ---------- ---------- ---------- | 527 |
| XP_024574193.1 | ---------- ---------- ---------- ---------- ---------- ---------- ---------- ---------- | 615 |
| XP_024583155.1 | ---------- ---------- ---------- ---------- ---------- ---------- ---------- ---------- | 419 |
| XP_024574913.1 | ---------- ---------- ---------- ---------- ---------- ---------- ---------- ---------- | 623 |
| XP_024578158.1 | ---------- ---------- ---------- ---------- ---------- ---------- ---------- ---------- | 106 |
| PvCRN22 | ---------- ---------- ---------- ---------- ---------- ---------- ---------- ---------- | 206 |
| XP_024585001.1 | ---------- ---------- ---------- ---------- ---------- ---------- ---------- ---------- | 127 |
| XP_024583843.1 | ---------- ---------- ---------- ---------- ---------- ---------- ---------- ---------- | 534 |
| XP_024578702.1 | ---------- ---------- ---------- ---------- ---------- ---------- ---------- ---------- | 299 |
| XP_024585805.1 | ---------- ---------- ---------- ---------- ---------- ---------- ---------- ---------- | 334 |
| XP_024580932.1 | ---------- ---------- ---------- ---------- ---------- ---------- ---------- ---------- | 508 |
| Consensus | ---------- ---------- ---------- ---------- ---------- ---------- ---------- ---------- | |
| Conservation (100% – 0%) | | |

```
                 7,700                7,720                7,740                7,760
PvCRN11        ---------- ---------- ---------- ---------- ---------- ---------- ---------- ---------- 215
XP_024579260.1 ---------- ---------- ---------- ---------- ---------- ---------- ---------- ---------- 163
XP_024573634.1 ---------- ---------- ---------- ---------- ---------- ---------- ---------- ---------- 169
PvCRN7         ---------- ---------- ---------- ---------- ---------- ---------- ---------- ---------- 753
XP_024585265.1 ---------- ---------- ---------- ---------- ---------- ---------- ---------- ---------- 128
PvCRN18        ---------- ---------- ---------- ---------- ---------- ---------- ---------- ---------- 302
XP_024578414.1 ---------- ---------- ---------- ---------- ---------- ---------- ---------- ---------- 624
XP_024579130.1 ---------- ---------- ---------- ---------- ---------- ---------- ---------- ---------- 345
XP_024584758.1 ---------- ---------- ---------- ---------- ---------- ---------- ---------- ---------- 214
XP_024586066.1 ---------- ---------- ---------- ---------- ---------- ---------- ---------- ---------- 263
XP_024585870.1 ---------- ---------- ---------- ---------- ---------- ---------- ---------- ---------- 280
PvCRN31        ---------- ---------- ---------- ---------- ---------- ---------- ---------- ---------- 583
XP_024577278.1 ---------- ---------- ---------- ---------- ---------- ---------- ---------- ---------- 333
XP_024585562.1 ---------- ---------- ---------- ---------- ---------- ---------- ---------- ---------- 158
XP_024573944.1 ---------- ---------- ---------- ---------- ---------- ---------- ---------- ---------- 592
XP_024579793.1 ---------- ---------- ---------- ---------- ---------- ---------- ---------- ---------- 163
PvCRN15        ---------- ---------- ---------- ---------- ---------- ---------- ---------- ---------- 290
PvCRN26        ---------- ---------- ---------- ---------- ---------- ---------- ---------- ---------- 279
PvCRN16        ---------- ---------- ---------- ---------- ---------- ---------- ---------- ---------- 365
PvCRN23        ---------- ---------- ---------- ---------- ---------- ---------- ---------- ---------- 426
XP_024573063.1 ---------- ---------- ---------- ---------- ---------- ---------- ---------- ---------- 83
PvCRN19        ---------- ---------- ---------- ---------- ---------- ---------- ---------- ---------- 164
XP_024581625.1 ---------- ---------- ---------- ---------- ---------- ---------- ---------- ---------- 135
XP_024583886.1 ---------- ---------- ---------- ---------- ---------- ---------- ---------- ---------- 96
XP_024584409.1 ---------- ---------- ---------- ---------- ---------- ---------- ---------- ---------- 213
XP_024572385.1 ---------- ---------- ---------- ---------- ---------- ---------- ---------- ---------- 310
PvCRN6         ---------- ---------- ---------- ---------- ---------- ---------- ---------- ---------- 372
PvCRN9         ---------- ---------- ---------- ---------- ---------- ---------- ---------- ---------- 611
XP_024580875.1 ---------- ---------- ---------- ---------- ---------- ---------- ---------- ---------- 127
PvCRN24        ---------- ---------- ---------- ---------- ---------- ---------- ---------- ---------- 165
PvCRN35        ---------- ---------- ---------- ---------- ---------- ---------- ---------- ---------- 212
PvCRN12        ---------- ---------- ---------- ---------- ---------- ---------- ---------- ---------- 171
XP_024576927.1 ---------- ---------- ---------- ---------- ---------- ---------- ---------- ---------- 555
XP_024578927.1 ---------- ---------- ---------- ---------- ---------- ---------- ---------- ---------- 327
XP_024586212.1 ---------- ---------- ---------- ---------- ---------- ---------- ---------- ---------- 616
XP_024578078.1 ---------- ---------- ---------- ---------- ---------- ---------- ---------- ---------- 262
XP_024575355.1 ---------- ---------- ---------- ---------- ---------- ---------- ---------- ---------- 295
XP_024584527.1 ---------- ---------- ---------- ---------- ---------- ---------- ---------- ---------- 168
XP_024586664.1 ---------- ---------- ---------- ---------- ---------- ---------- ---------- ---------- 357
PvCRN17        ---------- ---------- ---------- ---------- ---------- ---------- ---------- ---------- 187
PvCRN2         ---------- ---------- ---------- ---------- ---------- ---------- ---------- ---------- 168
XP_024577181.1 ---------- ---------- ---------- ---------- ---------- ---------- ---------- ---------- 207
XP_024581075.1 ---------- ---------- ---------- ---------- ---------- ---------- ---------- ---------- 156
XP_024575884.1 ---------- ---------- ---------- ---------- ---------- ---------- ---------- ---------- 155
XP_024586872.1 ---------- ---------- ---------- ---------- ---------- ---------- ---------- ---------- 182
XP_024573052.1 ---------- ---------- ---------- ---------- ---------- ---------- ---------- ---------- 187
XP_024586054.1 ---------- ---------- ---------- ---------- ---------- ---------- ---------- ---------- 237
PvCRN27        ---------- ---------- ---------- ---------- ---------- ---------- ---------- ---------- 313
PvCRN20        ---------- ---------- ---------- ---------- ---------- ---------- ---------- ---------- 159
PvCRN25        ---------- ---------- ---------- ---------- ---------- ---------- ---------- ---------- 194
PvCRN1         ---------- ---------- ---------- ---------- ---------- ---------- ---------- ---------- 580
PvCRN4         ---------- ---------- ---------- ---------- ---------- ---------- ---------- ---------- 583
PvCRN30        ---------- ---------- ---------- ---------- ---------- ---------- ---------- ---------- 220
PvCRN29        ---------- ---------- ---------- ---------- ---------- ---------- ---------- ---------- 312
XP_024583036.1 ---------- ---------- ---------- ---------- ---------- ---------- ---------- ---------- 681
PvCRN10        ---------- ---------- ---------- ---------- ---------- ---------- ---------- ---------- 217
XP_024577280.1 ---------- ---------- ---------- ---------- ---------- ---------- ---------- ---------- 283
XP_024572924.1 ---------- ---------- ---------- ---------- ---------- ---------- ---------- ---------- 412
PvCRN14        ---------- ---------- ---------- ---------- ---------- ---------- ---------- ---------- 151
XP_024577521.1 ---------- ---------- ---------- ---------- ---------- ---------- ---------- ---------- 108
PvCRN21        ---------- ---------- ---------- ---------- ---------- ---------- ---------- ---------- 479
XP_024574966.1 ---------- ---------- ---------- ---------- ---------- ---------- ---------- ---------- 155
XP_024575372.1 ---------- ---------- ---------- ---------- ---------- ---------- ---------- ---------- 188
XP_024581363.1 ---------- ---------- ---------- ---------- ---------- ---------- ---------- ---------- 229
XP_024583883.1 ---------- ---------- ---------- ---------- ---------- ---------- ---------- ---------- 381
XP_024579844.1 ---------- ---------- ---------- ---------- ---------- ---------- ---------- ---------- 476
XP_024574916.1 ---------- ---------- ---------- ---------- ---------- ---------- ---------- ---------- 527
XP_024574193.1 ---------- ---------- ---------- ---------- ---------- ---------- ---------- ---------- 615
XP_024583155.1 ---------- ---------- ---------- ---------- ---------- ---------- ---------- ---------- 419
XP_024574913.1 ---------- ---------- ---------- ---------- ---------- ---------- ---------- ---------- 623
XP_024578158.1 ---------- ---------- ---------- ---------- ---------- ---------- ---------- ---------- 106
PvCRN22        ---------- ---------- ---------- ---------- ---------- ---------- ---------- ---------- 206
XP_024585001.1 ---------- ---------- ---------- ---------- ---------- ---------- ---------- ---------- 127
XP_024583843.1 ---------- ---------- ---------- ---------- ---------- ---------- ---------- ---------- 534
XP_024578702.1 ---------- ---------- ---------- ---------- ---------- ---------- ---------- ---------- 299
XP_024585805.1 ---------- ---------- ---------- ---------- ---------- ---------- ---------- ---------- 334
XP_024580932.1 ---------- ---------- ---------- ---------- ---------- ---------- ---------- ---------- 508
Consensus      ---------- ---------- ---------- ---------- ---------- ---------- ---------- ----------
          100%
Conservation
            0%
```

| | 7,780 7,800 7,820 7,840 | |
|---|---|---|
| PvCRN11 | ---------- ---------- ---------- ---------- ---------- ---------- ---------- ---------- | 215 |
| XP_024579260.1 | ---------- ---------- ---------- ---------- ---------- ---------- ---------- ---------- | 163 |
| XP_024573634.1 | ---------- ---------- ---------- ---------- ---------- ---------- ---------- ---------- | 169 |
| PvCRN7 | ---------- ---------- ---------- ---------- ---------- ---------- ---------- ---------- | 753 |
| XP_024585265.1 | ---------- ---------- ---------- ---------- ---------- ---------- ---------- ---------- | 128 |
| PvCRN18 | ---------- ---------- ---------- ---------- ---------- ---------- ---------- ---------- | 302 |
| XP_024578414.1 | ---------- ---------- ---------- ---------- ---------- ---------- ---------- ---------- | 624 |
| XP_024579130.1 | ---------- ---------- ---------- ---------- ---------- ---------- ---------- ---------- | 345 |
| XP_024584758.1 | ---------- ---------- ---------- ---------- ---------- ---------- ---------- ---------- | 214 |
| XP_024586066.1 | ---------- ---------- ---------- ---------- ---------- ---------- ---------- ---------- | 263 |
| XP_024585870.1 | ---------- ---------- ---------- ---------- ---------- ---------- ---------- ---------- | 280 |
| PvCRN31 | ---------- ---------- ---------- ---------- ---------- ---------- ---------- ---------- | 583 |
| XP_024577278.1 | ---------- ---------- ---------- ---------- ---------- ---------- ---------- ---------- | 333 |
| XP_024585562.1 | ---------- ---------- ---------- ---------- ---------- ---------- ---------- ---------- | 158 |
| XP_024573944.1 | ---------- ---------- ---------- ---------- ---------- ---------- ---------- ---------- | 592 |
| XP_024579793.1 | ---------- ---------- ---------- ---------- ---------- ---------- ---------- ---------- | 163 |
| PvCRN15 | ---------- ---------- ---------- ---------- ---------- ---------- ---------- ---------- | 290 |
| PvCRN26 | ---------- ---------- ---------- ---------- ---------- ---------- ---------- ---------- | 279 |
| PvCRN16 | ---------- ---------- ---------- ---------- ---------- ---------- ---------- ---------- | 365 |
| PvCRN23 | ---------- ---------- ---------- ---------- ---------- ---------- ---------- ---------- | 426 |
| XP_024573063.1 | ---------- ---------- ---------- ---------- ---------- ---------- ---------- ---------- | 83 |
| PvCRN19 | ---------- ---------- ---------- ---------- ---------- ---------- ---------- ---------- | 164 |
| XP_024581625.1 | ---------- ---------- ---------- ---------- ---------- ---------- ---------- ---------- | 135 |
| XP_024583886.1 | ---------- ---------- ---------- ---------- ---------- ---------- ---------- ---------- | 96 |
| XP_024584409.1 | ---------- ---------- ---------- ---------- ---------- ---------- ---------- ---------- | 213 |
| XP_024572385.1 | ---------- ---------- ---------- ---------- ---------- ---------- ---------- ---------- | 310 |
| PvCRN6 | ---------- ---------- ---------- ---------- ---------- ---------- ---------- ---------- | 372 |
| PvCRN9 | ---------- ---------- ---------- ---------- ---------- ---------- ---------- ---------- | 611 |
| XP_024580875.1 | ---------- ---------- ---------- ---------- ---------- ---------- ---------- ---------- | 127 |
| PvCRN24 | ---------- ---------- ---------- ---------- ---------- ---------- ---------- ---------- | 165 |
| PvCRN35 | ---------- ---------- ---------- ---------- ---------- ---------- ---------- ---------- | 212 |
| PvCRN12 | ---------- ---------- ---------- ---------- ---------- ---------- ---------- ---------- | 171 |
| XP_024576927.1 | ---------- ---------- ---------- ---------- ---------- ---------- ---------- ---------- | 555 |
| XP_024578927.1 | ---------- ---------- ---------- ---------- ---------- ---------- ---------- ---------- | 327 |
| XP_024586212.1 | ---------- ---------- ---------- ---------- ---------- ---------- ---------- ---------- | 616 |
| XP_024578078.1 | ---------- ---------- ---------- ---------- ---------- ---------- ---------- ---------- | 262 |
| XP_024575355.1 | ---------- ---------- ---------- ---------- ---------- ---------- ---------- ---------- | 295 |
| XP_024584527.1 | ---------- ---------- ---------- ---------- ---------- ---------- ---------- ---------- | 168 |
| XP_024586664.1 | ---------- ---------- ---------- ---------- ---------- ---------- ---------- ---------- | 357 |
| PvCRN17 | ---------- ---------- ---------- ---------- ---------- ---------- ---------- ---------- | 187 |
| PvCRN2 | ---------- ---------- ---------- ---------- ---------- ---------- ---------- ---------- | 168 |
| XP_024577181.1 | ---------- ---------- ---------- ---------- ---------- ---------- ---------- ---------- | 207 |
| XP_024581075.1 | ---------- ---------- ---------- ---------- ---------- ---------- ---------- ---------- | 156 |
| XP_024575884.1 | ---------- ---------- ---------- ---------- ---------- ---------- ---------- ---------- | 155 |
| XP_024586872.1 | ---------- ---------- ---------- ---------- ---------- ---------- ---------- ---------- | 182 |
| XP_024573052.1 | ---------- ---------- ---------- ---------- ---------- ---------- ---------- ---------- | 187 |
| XP_024586054.1 | ---------- ---------- ---------- ---------- ---------- ---------- ---------- ---------- | 237 |
| PvCRN27 | ---------- ---------- ---------- ---------- ---------- ---------- ---------- ---------- | 313 |
| PvCRN20 | ---------- ---------- ---------- ---------- ---------- ---------- ---------- ---------- | 159 |
| PvCRN25 | ---------- ---------- ---------- ---------- ---------- ---------- ---------- ---------- | 194 |
| PvCRN1 | ---------- ---------- ---------- ---------- ---------- ---------- ---------- ---------- | 580 |
| PvCRN4 | ---------- ---------- ---------- ---------- ---------- ---------- ---------- ---------- | 583 |
| PvCRN30 | ---------- ---------- ---------- ---------- ---------- ---------- ---------- ---------- | 220 |
| PvCRN29 | ---------- ---------- ---------- ---------- ---------- ---------- ---------- ---------- | 312 |
| XP_024583036.1 | ---------- ---------- ---------- ---------- ---------- ---------- ---------- ---------- | 681 |
| PvCRN10 | ---------- ---------- ---------- ---------- ---------- ---------- ---------- ---------- | 217 |
| XP_024577280.1 | ---------- ---------- ---------- ---------- ---------- ---------- ---------- ---------- | 283 |
| XP_024572924.1 | ---------- ---------- ---------- ---------- ---------- ---------- ---------- ---------- | 412 |
| PvCRN14 | ---------- ---------- ---------- ---------- ---------- ---------- ---------- ---------- | 151 |
| XP_024577521.1 | ---------- ---------- ---------- ---------- ---------- ---------- ---------- ---------- | 108 |
| PvCRN21 | ---------- ---------- ---------- ---------- ---------- ---------- ---------- ---------- | 479 |
| XP_024574966.1 | ---------- ---------- ---------- ---------- ---------- ---------- ---------- ---------- | 155 |
| XP_024575372.1 | ---------- ---------- ---------- ---------- ---------- ---------- ---------- ---------- | 188 |
| XP_024581363.1 | ---------- ---------- ---------- ---------- ---------- ---------- ---------- ---------- | 229 |
| XP_024583883.1 | ---------- ---------- ---------- ---------- ---------- ---------- ---------- ---------- | 381 |
| XP_024579844.1 | ---------- ---------- ---------- ---------- ---------- ---------- ---------- ---------- | 476 |
| XP_024574916.1 | ---------- ---------- ---------- ---------- ---------- ---------- ---------- ---------- | 527 |
| XP_024574193.1 | ---------- ---------- ---------- ---------- ---------- ---------- ---------- ---------- | 615 |
| XP_024583155.1 | ---------- ---------- ---------- ---------- ---------- ---------- ---------- ---------- | 419 |
| XP_024574913.1 | ---------- ---------- ---------- ---------- ---------- ---------- ---------- ---------- | 623 |
| XP_024578158.1 | ---------- ---------- ---------- ---------- ---------- ---------- ---------- ---------- | 106 |
| PvCRN22 | ---------- ---------- ---------- ---------- ---------- ---------- ---------- ---------- | 206 |
| XP_024585001.1 | ---------- ---------- ---------- ---------- ---------- ---------- ---------- ---------- | 127 |
| XP_024583843.1 | ---------- ---------- ---------- ---------- ---------- ---------- ---------- ---------- | 534 |
| XP_024578702.1 | ---------- ---------- ---------- ---------- ---------- ---------- ---------- ---------- | 299 |
| XP_024585805.1 | ---------- ---------- ---------- ---------- ---------- ---------- ---------- ---------- | 334 |
| XP_024580932.1 | ---------- ---------- ---------- ---------- ---------- ---------- ---------- ---------- | 508 |
| Consensus | ---------- ---------- ---------- ---------- ---------- ---------- ---------- ---------- | |

100%
Conservation
0%

| | 7,860 · 7,880 · 7,900 · 7,920 | |
|---|---|---|
| PvCRN11 | ---------- ---------- ---------- ---------- ---------- ---------- ---------- ---------- | 215 |
| XP_024579260.1 | ---------- ---------- ---------- ---------- ---------- ---------- ---------- ---------- | 163 |
| XP_024573634.1 | ---------- ---------- ---------- ---------- ---------- ---------- ---------- ---------- | 169 |
| PvCRN7 | ---------- ---------- ---------- ---------- ---------- ---------- ---------- ---------- | 753 |
| XP_024585265.1 | ---------- ---------- ---------- ---------- ---------- ---------- ---------- ---------- | 128 |
| PvCRN18 | ---------- ---------- ---------- ---------- ---------- ---------- ---------- ---------- | 302 |
| XP_024578414.1 | ---------- ---------- ---------- ---------- ---------- ---------- ---------- ---------- | 624 |
| XP_024579130.1 | ---------- ---------- ---------- ---------- ---------- ---------- ---------- ---------- | 345 |
| XP_024584758.1 | ---------- ---------- ---------- ---------- ---------- ---------- ---------- ---------- | 214 |
| XP_024586066.1 | ---------- ---------- ---------- ---------- ---------- ---------- ---------- ---------- | 263 |
| XP_024585870.1 | ---------- ---------- ---------- ---------- ---------- ---------- ---------- ---------- | 280 |
| PvCRN31 | ---------- ---------- ---------- ---------- ---------- ---------- ---------- ---------- | 583 |
| XP_024577278.1 | ---------- ---------- ---------- ---------- ---------- ---------- ---------- ---------- | 333 |
| XP_024585562.1 | ---------- ---------- ---------- ---------- ---------- ---------- ---------- ---------- | 158 |
| XP_024573944.1 | ---------- ---------- ---------- ---------- ---------- ---------- ---------- ---------- | 592 |
| XP_024579793.1 | ---------- ---------- ---------- ---------- ---------- ---------- ---------- ---------- | 163 |
| PvCRN15 | ---------- ---------- ---------- ---------- ---------- ---------- ---------- ---------- | 290 |
| PvCRN26 | ---------- ---------- ---------- ---------- ---------- ---------- ---------- ---------- | 279 |
| PvCRN16 | ---------- ---------- ---------- ---------- ---------- ---------- ---------- ---------- | 365 |
| PvCRN23 | ---------- ---------- ---------- ---------- ---------- ---------- ---------- ---------- | 426 |
| XP_024573063.1 | ---------- ---------- ---------- ---------- ---------- ---------- ---------- ---------- | 83 |
| PvCRN19 | ---------- ---------- ---------- ---------- ---------- ---------- ---------- ---------- | 164 |
| XP_024581625.1 | ---------- ---------- ---------- ---------- ---------- ---------- ---------- ---------- | 135 |
| XP_024583886.1 | ---------- ---------- ---------- ---------- ---------- ---------- ---------- ---------- | 96 |
| XP_024584409.1 | ---------- ---------- ---------- ---------- ---------- ---------- ---------- ---------- | 213 |
| XP_024572385.1 | ---------- ---------- ---------- ---------- ---------- ---------- ---------- ---------- | 310 |
| PvCRN6 | ---------- ---------- ---------- ---------- ---------- ---------- ---------- ---------- | 372 |
| PvCRN9 | ---------- ---------- ---------- ---------- ---------- ---------- ---------- ---------- | 611 |
| XP_024580875.1 | ---------- ---------- ---------- ---------- ---------- ---------- ---------- ---------- | 127 |
| PvCRN24 | ---------- ---------- ---------- ---------- ---------- ---------- ---------- ---------- | 165 |
| PvCRN35 | ---------- ---------- ---------- ---------- ---------- ---------- ---------- ---------- | 212 |
| PvCRN12 | ---------- ---------- ---------- ---------- ---------- ---------- ---------- ---------- | 171 |
| XP_024576927.1 | ---------- ---------- ---------- ---------- ---------- ---------- ---------- ---------- | 555 |
| XP_024578927.1 | ---------- ---------- ---------- ---------- ---------- ---------- ---------- ---------- | 327 |
| XP_024586212.1 | ---------- ---------- ---------- ---------- ---------- ---------- ---------- ---------- | 616 |
| XP_024578078.1 | ---------- ---------- ---------- ---------- ---------- ---------- ---------- ---------- | 262 |
| XP_024575355.1 | ---------- ---------- ---------- ---------- ---------- ---------- ---------- ---------- | 295 |
| XP_024584527.1 | ---------- ---------- ---------- ---------- ---------- ---------- ---------- ---------- | 168 |
| XP_024586664.1 | ---------- ---------- ---------- ---------- ---------- ---------- ---------- ---------- | 357 |
| PvCRN17 | ---------- ---------- ---------- ---------- ---------- ---------- ---------- ---------- | 187 |
| PvCRN2 | ---------- ------ES-L EFRLAGRE-- ---------- ---------- ---------- ---------- ---------- | 179 |
| XP_024577181.1 | ---------- ---------- ---------- ---------- ---------- ---------- ---------- ---------- | 207 |
| XP_024581075.1 | ---------- ---------- ---------- ---------- ---------- ---------- ---------- ---------- | 156 |
| XP_024575884.1 | ---------- ---------- ---------- ---------- ---------- ---------- ---------- ---------- | 155 |
| XP_024586872.1 | ---------- ---------- ---------- ---------- ---------- ---------- ---------- ---------- | 182 |
| XP_024573052.1 | ---------- ---------- ---------- ---------- ---------- ---------- ---------- ---------- | 187 |
| XP_024586054.1 | ---------- ---------- ---------- ---------- ---------- ---------- ---------- ---------- | 237 |
| PvCRN27 | ---------- ---------- ---------- ---------- ---------- ---------- ---------- ---------- | 313 |
| PvCRN20 | ---------- ---------- ---------- ---------- ---------- ---------- ---------- ---------- | 159 |
| PvCRN25 | ---------- ---------- ---------- ---------- ---------- ---------- ---------- ---------- | 194 |
| PvCRN1 | ---------- ---------- ---------- ---------- ---------- ---------- ---------- ---------- | 580 |
| PvCRN4 | ---------- ---------- ---------- ---------- ---------- ---------- ---------- ---------- | 583 |
| PvCRN30 | ---------- ---------- ---------- ---------- ---------- ---------- ---------- ---------- | 220 |
| PvCRN29 | ---------- ---------- ---------- ---------- ---------- ---------- ---------- ---------- | 312 |
| XP_024583036.1 | ---------- ---------- ---------- ---------- ---------- ---------- ---------- ---------- | 681 |
| PvCRN10 | ---------- ---------- ---------- ---------- ---------- ---------- ---------- ---------- | 217 |
| XP_024577280.1 | ---------- ---------- ---------- ---------- ---------- ---------- ---------- ---------- | 283 |
| XP_024572924.1 | ---------- ---------- ---------- ---------- ---------- ---------- ---------- ---------- | 412 |
| PvCRN14 | ---------- ---------- ---------- ---------- ---------- ---------- ---------- ---------- | 151 |
| XP_024577521.1 | ---------- ---------- ---------- ---------- ---------- ---------- ---------- ---------- | 108 |
| PvCRN21 | ---------- ---------- ---------- ---------- ---------- ---------- ---------- ---------- | 479 |
| XP_024574966.1 | ---------- ---------- ---------- ---------- ---------- ---------- ---------- ---------- | 155 |
| XP_024575372.1 | ---------- ---------- ---------- ---------- ---------- ---------- ---------- ---------- | 188 |
| XP_024581363.1 | ---------- ---------- ---------- ---------- ---------- ---------- ---------- ---------- | 229 |
| XP_024583883.1 | ---------- ---------- ---------- ---------- ---------- ---------- ---------- ---------- | 381 |
| XP_024579844.1 | ---------- ---------- ---------- ---------- ---------- ---------- ---------- ---------- | 476 |
| XP_024574916.1 | ---------- ---------Y CDKFYDLNVY EYKYKGSDET IPKIGFISQD LYAAGYINLL SMTPNENLKK VNEDDIEGYQ | 588 |
| XP_024574193.1 | ---------- ---------- ---------- ---------- ---------- ---------- ---------- ---------- | 615 |
| XP_024583155.1 | ---------- ---------- ---------- ---------- ---------- ---------- ---------- ---------- | 419 |
| XP_024574913.1 | ---------- ---------- ---------- ---------- ---------- ---------- ---------- ---------- | 623 |
| XP_024578158.1 | ---------- ---------- ---------- ---------- ---------- ---------- ---------- ---------- | 106 |
| PvCRN22 | ---------- ---------- ---------- ---------- ---------- ---------- ---------- ---------- | 206 |
| XP_024585001.1 | ---------- ---------- ---------- ---------- ---------- ---------- ---------- ---------- | 127 |
| XP_024583843.1 | ---------- ---------- ---------- ---------- ---------- ---------- ---------- ---------- | 534 |
| XP_024578702.1 | ---------- ---------- ---------- ---------- ---------- ---------- ---------- ---------- | 299 |
| XP_024585805.1 | ---------- ---------- ---------- ---------- ---------- ---------- ---------- ---------- | 334 |
| XP_024580932.1 | ---------- ---------- ---------- ---------- ---------- ---------- ---------- ---------- | 508 |
| Consensus | ---------- ---------- ---------- ---------- ---------- ---------- ---------- ---------- | |
| Conservation (100% / 0%) | | |

```
                7,940                7,960                7,980                8,000
PvCRN11         ---------- ---------- ---------- ---------- ---------- ---------- ---------- ---------- 215
XP_024579260.1  ---------- ---------- ---------- ---------- ---------- ---------- ---------- ---------- 163
XP_024573634.1  ---------- ---------- ---------- ---------- ---------- ---------- ---------- ---------- 169
PvCRN7          ---------- ---------- ---------- ---------- ---------- ---------- ---------- ---------- 753
XP_024585265.1  ---------- ---------- ---------- ---------- ---------- ---------- ---------- ---------- 128
PvCRN18         ---------- ---------- ---------- ---------- ---------- ---------- ---------- ---------- 302
XP_024578414.1  ---------- ---------- ---------- ---------- ---------- ---------- ---------- ---------- 624
XP_024579130.1  ---------- ---------- ---------- ---------- ---------- ---------- ---------- ---------- 345
XP_024584758.1  ---------- ---------- ---------- ---------- ---------- ---------- ---------- ---------- 214
XP_024586066.1  ---------- ---------- ---------- ---------- ---------- ---------- ---------- ---------- 263
XP_024585870.1  ---------- ---------- ---------- ---------- ---------- ---------- ---------- ---------- 280
PvCRN31         ---------- ---------- ---------- ---------- ---------- ---------- ---------- ---------- 583
XP_024577278.1  ---------- ---------- ---------- ---------- ---------- ---------- ---------- ---------- 333
XP_024585562.1  ---------- ---------- ---------- ---------- ---------- ---------- ---------- ---------- 158
XP_024573944.1  ---------- ---------- ---------- ---------- ---------- ---------- ---------- ---------- 592
XP_024579793.1  ---------- ---------- ---------- ---------- ---------- ---------- ---------- ---------- 163
PvCRN15         ---------- ---------- ---------- ---------- ---------- ---------- ---------- ---------- 290
PvCRN26         ---------- ---------- ---------- ---------- ---------- ---------- ---------- ---------- 279
PvCRN16         ---------- ---------- ---------- ---------- ---------- ---------- ---------- ---------- 365
PvCRN23         ---------- ---------- ---------- ---------- ---------- ---------- ---------- ---------- 426
XP_024573063.1  ---------- ---------- ---------- ---------- ---------- ---------- ---------- ---------- 83
PvCRN19         ---------- ---------- ---------- ---------- ---------- ---------- ---------- ---------- 164
XP_024581625.1  ---------- ---------- ---------- ---------- ---------- ---------- ---------- ---------- 135
XP_024583886.1  ---------- ---------- ---------- ---------- ---------- ---------- ---------- ---------- 96
XP_024584409.1  ---------- ---------- ---------- ---------- ---------- ---------- ---------- ---------- 213
XP_024572385.1  ---------- ---------- ---------- ---------- ---------- ---------- ---------- ---------- 310
PvCRN6          ---------- ---------- ---------- ---------- ---------- ---------- ---------- ---------- 372
PvCRN9          ---------- ---------- ---------- ---------- ---------- ---------- ---------- ---------- 611
XP_024580875.1  ---------- ---------- ---------- ---------- ---------- ---------- ---------- ---------- 127
PvCRN24         ---------- ---------- ---------- ---------- ---------- ---------- ---------- ---------- 165
PvCRN35         ---------- ---------- ---------- ---------- ---------- ---------- ---------- ---------- 212
PvCRN12         ---------- ---------- ---------- ---------- ---------- ---------- ---------- ---------- 171
XP_024576927.1  ---------- ---------- ---------- ---------- ---------- ---------- ---------- ---------- 555
XP_024578927.1  ---------- ---------- ---------- ---------- ---------- ---------- ---------- ---------- 327
XP_024586212.1  ---------- ---------- ---------- ---------- ---------- ---------- ---------- ---------- 616
XP_024578078.1  ---------- ---------- ---------- ---------- ---------- ---------- ---------- ---------- 262
XP_024575355.1  ---------- ---------- ---------- ---------- ---------- ---------- ---------- ---------- 295
XP_024584527.1  ---------- ---------- ---------- ---------- ---------- ---------- ---------- ---------- 168
XP_024586664.1  ---------- ---------- ---------- ---------- ---------- ---------- ---------- ---------- 357
PvCRN17         ---------- ---------- ---------- ---------- ---------- ---------- ---------- ---------- 187
PvCRN2          ---------- ---------- ---------- ---------- ---------- ---------- ---------- ---------- 179
XP_024577181.1  ---------- ---------- ---------- ---------- ---------- ---------- ---------- ---------- 207
XP_024581075.1  ---------- ---------- ---------- ---------- ---------- ---------- ---------- ---------- 156
XP_024575884.1  ---------- ---------- ---------- ---------- ---------- ---------- ---------- ---------- 155
XP_024586872.1  ---------- ---------- ---------- ---------- ---------- ---------- ---------- ---------- 182
XP_024573052.1  ---------- ---------- ---------- ---------- ---------- ---------- ---------- ---------- 187
XP_024586054.1  ---------- ---------- ---------- ---------- ---------- ---------- ---------- ---------- 237
PvCRN27         ---------- ---------- ---------- ---------- ---------- ---------- ---------- ---------- 313
PvCRN20         ---------- ---------- ---------- ---------- ---------- ---------- ---------- ---------- 159
PvCRN25         ---------- ---------- ---------- ---------- ---------- ---------- ---------- ---------- 194
PvCRN1          ---------- ---------- ---------- ---------- ---------- ---------- ---------- ---------- 580
PvCRN4          ---------- ---------- ---------- ---------- ---------- ---------- ---------- ---------- 583
PvCRN30         ---------- ---------- ---------- ---------- ---------- ---------- ---------- ---------- 220
PvCRN29         ---------- ---------- ---------- ---------- ---------- ---------- ---------- ---------- 312
XP_024583036.1  ---------- ---------- ---------- ---------- ---------- ---------- ---------- ---------- 681
PvCRN10         ---------- ---------- ---------- ---------- ---------- ---------- ---------- ---------- 217
XP_024577280.1  ---------- ---------- ---------- ---------- ---------- ---------- ---------- ---------- 283
XP_024572924.1  ---------- ---------- ---------- ---------- ---------- ---------- ---------- ---------- 412
PvCRN14         ---------- ---------- ---------- ---------- ---------- ---------- ---------- ---------- 151
XP_024577521.1  ---------- ---------- ---------- ---------- ---------- ---------- ---------- ---------- 108
PvCRN21         ---------- ---------- ---------- ---------- ---------- ---------- ---------- ---------- 479
XP_024574966.1  ---------- ---------- ---------- ---------- ---------- ---------- ---------- ---------- 155
XP_024575372.1  ---------- ---------- ---------- ---------- ---------- ---------- ---------- ---------- 188
XP_024581363.1  ---------- ---------- ---------- ---------- ---------- ---------- ---------- ---------- 229
XP_024583883.1  ---------- ---------- ---------- ---------- ---------- ---------- ---------- ---------- 381
XP_024579844.1  ---------- ---------- ---------- ---------- ---------- ---------- ---------- ---------- 476
XP_024574916.1  M--------- ---------- ---------- ---------- ---------- ---------- ---------- ---------- 589
XP_024574193.1  ---------- ---------- ---------- ---------- ---------- ---------- ---------- ---------- 615
XP_024583155.1  ---------- ---------- ---------- ---------- ---------- ---------- ---------- ---------- 419
XP_024574913.1  ---------- ---------- ---------- ---------- ---------- ---------- ---------- ---------- 623
XP_024578158.1  ---------- ---------- ---------- ---------- ---------- ---------- ---------- ---------- 106
PvCRN22         ---------- ---------- ---------- ---------- ---------- ---------- ---------- ---------- 206
XP_024585001.1  ---------- ---------- ---------- ---------- ---------- ---------- ---------- ---------- 127
XP_024583843.1  ---------- ---------- ---------- ---------- ---------- ---------- ---------- ---------- 534
XP_024578702.1  ---------- ---------- ---------- ---------- ---------- ---------- ---------- ---------- 299
XP_024585805.1  ---------- ---------- ---------- ---------- ---------- ---------- ---------- ---------- 334
XP_024580932.1  ---------- ---------- ---------- ---------- ---------- ---------- ---------- ---------- 508
Consensus       ---------- ---------- ---------- ---------- ---------- ---------- ---------- ----------
                100%
Conservation
                0%
```

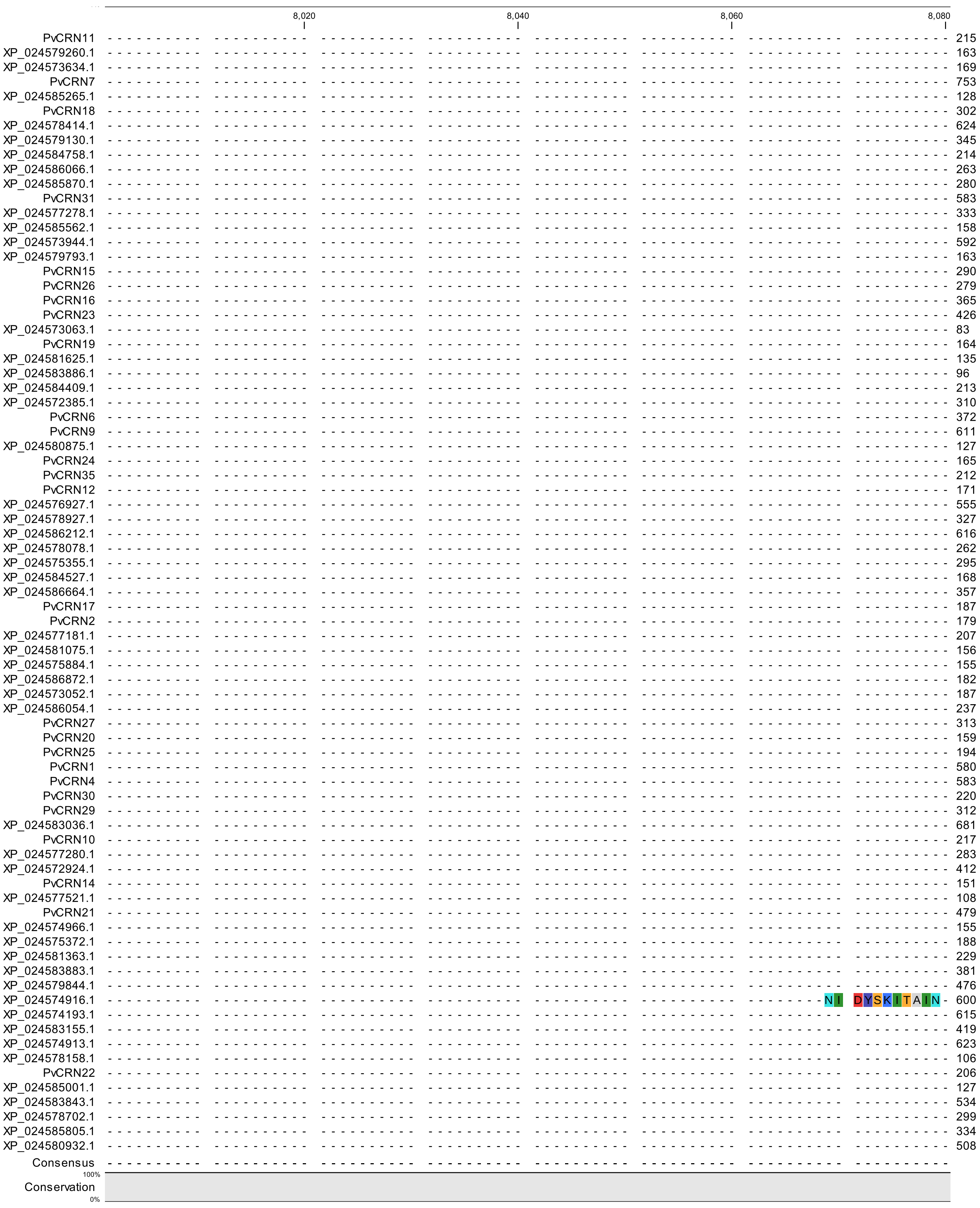
8,020
8,040
8,060
8,080
PvCRN11 215
XP_024579260.1 163
XP_024573634.1 169
PvCRN7 753
XP_024585265.1 128
PvCRN18 302
XP_024578414.1 624
XP_024579130.1 345
XP_024584758.1 214
XP_024586066.1 263
XP_024585870.1 280
PvCRN31 583
XP_024577278.1 333
XP_024585562.1 158
XP_024573944.1 592
XP_024579793.1 163
PvCRN15 290
PvCRN26 279
PvCRN16 365
PvCRN23 426
XP_024573063.1 83
PvCRN19 164
XP_024581625.1 135
XP_024583886.1 96
XP_024584409.1 213
XP_024572385.1 310
PvCRN6 372
PvCRN9 611
XP_024580875.1 127
PvCRN24 165
PvCRN35 212
PvCRN12 171
XP_024576927.1 555
XP_024578927.1 327
XP_024586212.1 616
XP_024578078.1 262
XP_024575355.1 295
XP_024584527.1 168
XP_024586664.1 357
PvCRN17 187
PvCRN2 179
XP_024577181.1 207
XP_024581075.1 156
XP_024575884.1 155
XP_024586872.1 182
XP_024573052.1 187
XP_024586054.1 237
PvCRN27 313
PvCRN20 159
PvCRN25 194
PvCRN1 580
PvCRN4 583
PvCRN30 220
PvCRN29 312
XP_024583036.1 681
PvCRN10 217
XP_024577280.1 283
XP_024572924.1 412
PvCRN14 151
XP_024577521.1 108
PvCRN21 479
XP_024574966.1 155
XP_024575372.1 188
XP_024581363.1 229
XP_024583883.1 381
XP_024579844.1 476
XP_024574916.1 NI DYSKITAIN- 600
XP_024574193.1 615
XP_024583155.1 419
XP_024574913.1 623
XP_024578158.1 106
PvCRN22 206
XP_024585001.1 127
XP_024583843.1 534
XP_024578702.1 299
XP_024585805.1 334
XP_024580932.1 508
Consensus
100%
Conservation
0%

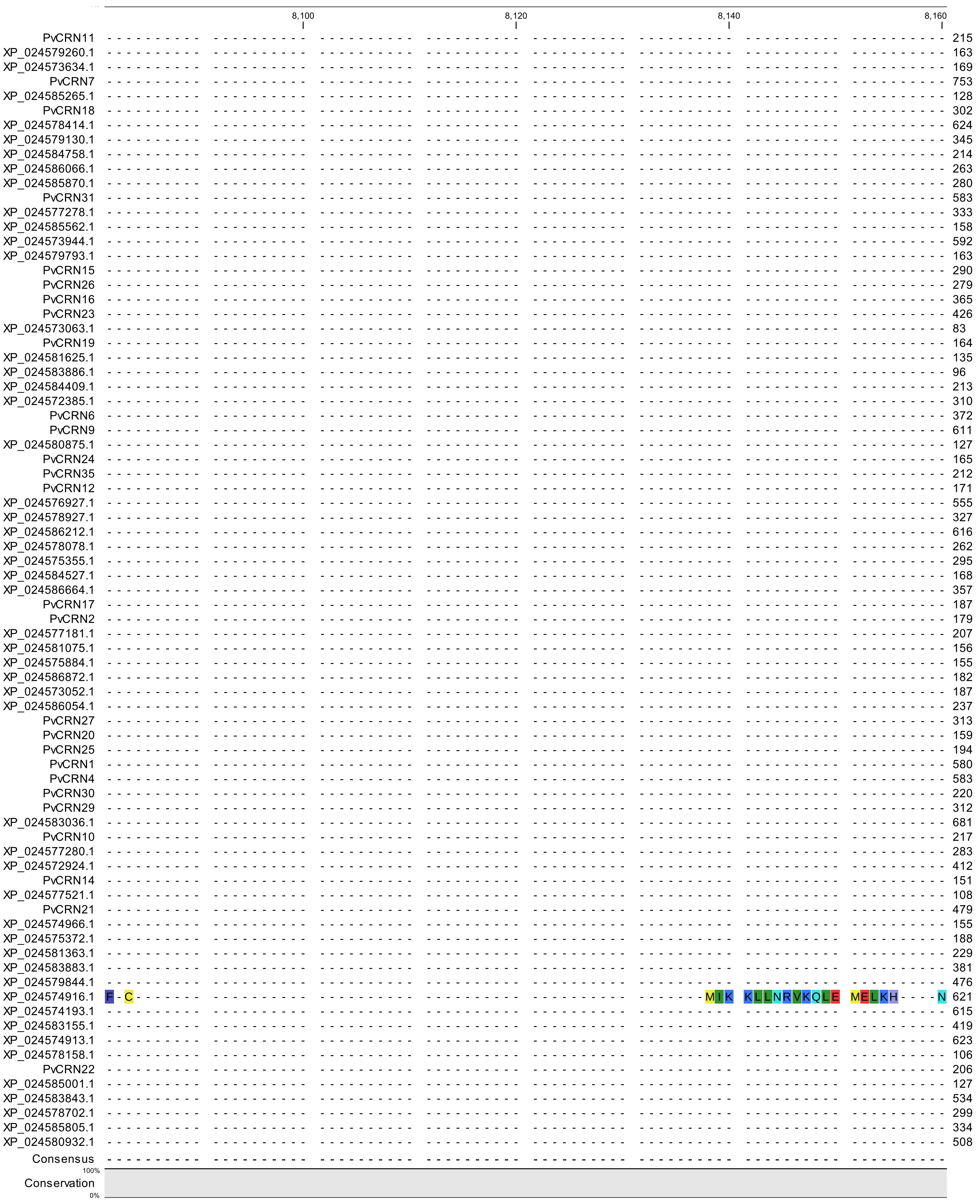

8,100
8,120
8,140
8,160
PvCRN11 ---------- ---------- ---------- ---------- ---------- ---------- ---------- ---------- 215
XP_024579260.1 ---------- ---------- ---------- ---------- ---------- ---------- ---------- ---------- 163
XP_024573634.1 ---------- ---------- ---------- ---------- ---------- ---------- ---------- ---------- 169
PvCRN7 ---------- ---------- ---------- ---------- ---------- ---------- ---------- ---------- 753
XP_024585265.1 ---------- ---------- ---------- ---------- ---------- ---------- ---------- ---------- 128
PvCRN18 ---------- ---------- ---------- ---------- ---------- ---------- ---------- ---------- 302
XP_024578414.1 ---------- ---------- ---------- ---------- ---------- ---------- ---------- ---------- 624
XP_024579130.1 ---------- ---------- ---------- ---------- ---------- ---------- ---------- ---------- 345
XP_024584758.1 ---------- ---------- ---------- ---------- ---------- ---------- ---------- ---------- 214
XP_024586066.1 ---------- ---------- ---------- ---------- ---------- ---------- ---------- ---------- 263
XP_024585870.1 ---------- ---------- ---------- ---------- ---------- ---------- ---------- ---------- 280
PvCRN31 ---------- ---------- ---------- ---------- ---------- ---------- ---------- ---------- 583
XP_024577278.1 ---------- ---------- ---------- ---------- ---------- ---------- ---------- ---------- 333
XP_024585562.1 ---------- ---------- ---------- ---------- ---------- ---------- ---------- ---------- 158
XP_024573944.1 ---------- ---------- ---------- ---------- ---------- ---------- ---------- ---------- 592
XP_024579793.1 ---------- ---------- ---------- ---------- ---------- ---------- ---------- ---------- 163
PvCRN15 ---------- ---------- ---------- ---------- ---------- ---------- ---------- ---------- 290
PvCRN26 ---------- ---------- ---------- ---------- ---------- ---------- ---------- ---------- 279
PvCRN16 ---------- ---------- ---------- ---------- ---------- ---------- ---------- ---------- 365
PvCRN23 ---------- ---------- ---------- ---------- ---------- ---------- ---------- ---------- 426
XP_024573063.1 ---------- ---------- ---------- ---------- ---------- ---------- ---------- ---------- 83
PvCRN19 ---------- ---------- ---------- ---------- ---------- ---------- ---------- ---------- 164
XP_024581625.1 ---------- ---------- ---------- ---------- ---------- ---------- ---------- ---------- 135
XP_024583886.1 ---------- ---------- ---------- ---------- ---------- ---------- ---------- ---------- 96
XP_024584409.1 ---------- ---------- ---------- ---------- ---------- ---------- ---------- ---------- 213
XP_024572385.1 ---------- ---------- ---------- ---------- ---------- ---------- ---------- ---------- 310
PvCRN6 ---------- ---------- ---------- ---------- ---------- ---------- ---------- ---------- 372
PvCRN9 ---------- ---------- ---------- ---------- ---------- ---------- ---------- ---------- 611
XP_024580875.1 ---------- ---------- ---------- ---------- ---------- ---------- ---------- ---------- 127
PvCRN24 ---------- ---------- ---------- ---------- ---------- ---------- ---------- ---------- 165
PvCRN35 ---------- ---------- ---------- ---------- ---------- ---------- ---------- ---------- 212
PvCRN12 ---------- ---------- ---------- ---------- ---------- ---------- ---------- ---------- 171
XP_024576927.1 ---------- ---------- ---------- ---------- ---------- ---------- ---------- ---------- 555
XP_024578927.1 ---------- ---------- ---------- ---------- ---------- ---------- ---------- ---------- 327
XP_024586212.1 ---------- ---------- ---------- ---------- ---------- ---------- ---------- ---------- 616
XP_024578078.1 ---------- ---------- ---------- ---------- ---------- ---------- ---------- ---------- 262
XP_024575355.1 ---------- ---------- ---------- ---------- ---------- ---------- ---------- ---------- 295
XP_024584527.1 ---------- ---------- ---------- ---------- ---------- ---------- ---------- ---------- 168
XP_024586664.1 ---------- ---------- ---------- ---------- ---------- ---------- ---------- ---------- 357
PvCRN17 ---------- ---------- ---------- ---------- ---------- ---------- ---------- ---------- 187
PvCRN2 ---------- ---------- ---------- ---------- ---------- ---------- ---------- ---------- 179
XP_024577181.1 ---------- ---------- ---------- ---------- ---------- ---------- ---------- ---------- 207
XP_024581075.1 ---------- ---------- ---------- ---------- ---------- ---------- ---------- ---------- 156
XP_024575884.1 ---------- ---------- ---------- ---------- ---------- ---------- ---------- ---------- 155
XP_024586872.1 ---------- ---------- ---------- ---------- ---------- ---------- ---------- ---------- 182
XP_024573052.1 ---------- ---------- ---------- ---------- ---------- ---------- ---------- ---------- 187
XP_024586054.1 ---------- ---------- ---------- ---------- ---------- ---------- ---------- ---------- 237
PvCRN27 ---------- ---------- ---------- ---------- ---------- ---------- ---------- ---------- 313
PvCRN20 ---------- ---------- ---------- ---------- ---------- ---------- ---------- ---------- 159
PvCRN25 ---------- ---------- ---------- ---------- ---------- ---------- ---------- ---------- 194
PvCRN1 ---------- ---------- ---------- ---------- ---------- ---------- ---------- ---------- 580
PvCRN4 ---------- ---------- ---------- ---------- ---------- ---------- ---------- ---------- 583
PvCRN30 ---------- ---------- ---------- ---------- ---------- ---------- ---------- ---------- 220
PvCRN29 ---------- ---------- ---------- ---------- ---------- ---------- ---------- ---------- 312
XP_024583036.1 ---------- ---------- ---------- ---------- ---------- ---------- ---------- ---------- 681
PvCRN10 ---------- ---------- ---------- ---------- ---------- ---------- ---------- ---------- 217
XP_024577280.1 ---------- ---------- ---------- ---------- ---------- ---------- ---------- ---------- 283
XP_024572924.1 ---------- ---------- ---------- ---------- ---------- ---------- ---------- ---------- 412
PvCRN14 ---------- ---------- ---------- ---------- ---------- ---------- ---------- ---------- 151
XP_024577521.1 ---------- ---------- ---------- ---------- ---------- ---------- ---------- ---------- 108
PvCRN21 ---------- ---------- ---------- ---------- ---------- ---------- ---------- ---------- 479
XP_024574966.1 ---------- ---------- ---------- ---------- ---------- ---------- ---------- ---------- 155
XP_024575372.1 ---------- ---------- ---------- ---------- ---------- ---------- ---------- ---------- 188
XP_024581363.1 ---------- ---------- ---------- ---------- ---------- ---------- ---------- ---------- 229
XP_024583883.1 ---------- ---------- ---------- ---------- ---------- ---------- ---------- ---------- 381
XP_024579844.1 ---------- ---------- ---------- ---------- ---------- ---------- ---------- ---------- 476
XP_024574916.1 F-C------- ---------- ---------- ---------- ---------- -------MIK KLLNRVKQLE MELKH----N 621
XP_024574193.1 ---------- ---------- ---------- ---------- ---------- ---------- ---------- ---------- 615
XP_024583155.1 ---------- ---------- ---------- ---------- ---------- ---------- ---------- ---------- 419
XP_024574913.1 ---------- ---------- ---------- ---------- ---------- ---------- ---------- ---------- 623
XP_024578158.1 ---------- ---------- ---------- ---------- ---------- ---------- ---------- ---------- 106
PvCRN22 ---------- ---------- ---------- ---------- ---------- ---------- ---------- ---------- 206
XP_024585001.1 ---------- ---------- ---------- ---------- ---------- ---------- ---------- ---------- 127
XP_024583843.1 ---------- ---------- ---------- ---------- ---------- ---------- ---------- ---------- 534
XP_024578702.1 ---------- ---------- ---------- ---------- ---------- ---------- ---------- ---------- 299
XP_024585805.1 ---------- ---------- ---------- ---------- ---------- ---------- ---------- ---------- 334
XP_024580932.1 ---------- ---------- ---------- ---------- ---------- ---------- ---------- ---------- 508
Consensus ---------- ---------- ---------- ---------- ---------- ---------- ---------- ----------
100%
Conservation
0%

| Sequence | 8,180 · 8,200 · 8,220 · 8,240 | |
|---|---|---|
| PvCRN11 | ---------- ---------- ---------- ---------- ---------- ---------- ---------- ---------- | 215 |
| XP_024579260.1 | ---------- ---------- ---------- ---------- ---------- ---------- ---------- ---------- | 163 |
| XP_024573634.1 | ---------- ---------- ---------- ---------- ---------- ---------- ---------- ---------- | 169 |
| PvCRN7 | ---------- ---------- ---------- ---------- ---------- ---------- ---------- ---------- | 753 |
| XP_024585265.1 | ---------- ---------- ---------- ---------- ---------- ---------- ---------- ---------- | 128 |
| PvCRN18 | ---------- ---------- ---------- ---------- ---------- ---------- ---------- ---------- | 302 |
| XP_024578414.1 | ---------- ---------- ---------- ---------- ---------- ---------- ---------- ---------- | 624 |
| XP_024579130.1 | ---------- ---------- ---------- ---------- ---------- ---------- ---------- ---------- | 345 |
| XP_024584758.1 | ---------- ---------- ---------- ---------- ---------- ---------- ---------- ---------- | 214 |
| XP_024586066.1 | ---------- ---------- ---------- ---------- ---------- ---------- ---------- ---------- | 263 |
| XP_024585870.1 | ---------- ---------- ---------- ---------- ---------- ---------- ---------- ---------- | 280 |
| PvCRN31 | ---------- ---------- ---------- ---------- ---------- ---------- ---------- ---------- | 583 |
| XP_024577278.1 | ---------- ---------- ---------- ---------- ---------- ---------- ---------- ---------- | 333 |
| XP_024585562.1 | ---------- ---------- ---------- ---------- ---------- ---------- ---------- ---------- | 158 |
| XP_024573944.1 | ---------- ---------- ---------- ---------- ---------- ---------- ---------- ---------- | 592 |
| XP_024579793.1 | ---------- ---------- ---------- ---------- ---------- ---------- ---------- ---------- | 163 |
| PvCRN15 | ---------- ---------- ---------- ---------- ---------- ---------- ---------- ---------- | 290 |
| PvCRN26 | ---------- ---------- ---------- ---------- ---------- ---------- ---------- ---------- | 279 |
| PvCRN16 | ---------- ---------- ---------- ---------- ---------- ---------- ---------- ---------- | 365 |
| PvCRN23 | ---------- ---------- ---------- ---------- ---------- ---------- ---------- ---------- | 426 |
| XP_024573063.1 | ---------- ---------- ---------- ---------- ---------- ---------- ---------- ---------- | 83 |
| PvCRN19 | ---------- ---------- ---------- ---------- ---------- ---------- ---------- ---------- | 164 |
| XP_024581625.1 | ---------- ---------- ---------- ---------- ---------- ---------- ---------- ---------- | 135 |
| XP_024583886.1 | ---------- ---------- ---------- ---------- ---------- ---------- ---------- ---------- | 96 |
| XP_024584409.1 | ---------- ---------- ---------- ---------- ---------- ---------- ---------- ---------- | 213 |
| XP_024572385.1 | ---------- ---------- ---------- ---------- ---------- ---------- ---------- ---------- | 310 |
| PvCRN6 | ---------- ---------- ---------- ---------- ---------- ---------- ---------- ---------- | 372 |
| PvCRN9 | ---------- ---------- ---------- ---------- ---------- ---------- ---------- ---------- | 611 |
| XP_024580875.1 | ---------- ---------- ---------- ---------- ---------- ---------- ---------- ---------- | 127 |
| PvCRN24 | ---------- ---------- ---------- ---------- ---------- ---------- ---------- ---------- | 165 |
| PvCRN35 | ---------- ---------- ---------- ---------- ---------- ---------- ---------- ---------- | 212 |
| PvCRN12 | ---------- ---------- ---------- ---------- ---------- ---------- ---------- ---------- | 171 |
| XP_024576927.1 | ---------- ---------- ---------- ---------- ---------- ---------- ---------- ---------- | 555 |
| XP_024578927.1 | ---------- ---------- ---------- ---------- ---------- ---------- ---------- ---------- | 327 |
| XP_024586212.1 | ---------- ---------- ---------- ---------- ---------- ---------- ---------- ---------- | 616 |
| XP_024578078.1 | ---------- ---------- ---------- ---------- ---------- ---------- ---------- ---------- | 262 |
| XP_024575355.1 | ---------- ---------- ---------- ---------- ---------- ---------- ---------- ---------- | 295 |
| XP_024584527.1 | ---------- ---------- ---------- ---------- ---------- ---------- ---------- ---------- | 168 |
| XP_024586664.1 | ---------- ---------- ---------- ---------- ---------- ---------- ---------- ---------- | 357 |
| PvCRN17 | ---------- ---------- ---------- ---------- ---------- ---------- ---------- ---------- | 187 |
| PvCRN2 | ---------- ---------- ---------- ---------- ---------- ---------- ---------- ---------- | 179 |
| XP_024577181.1 | ---------- ---------- ---------- ---------- ---------- ---------- ---------- ---------- | 207 |
| XP_024581075.1 | ---------- ---------- ---------- ---------- ---------- ---------- ---------- ---------- | 156 |
| XP_024575884.1 | ---------- ---------- ---------- ---------- ---------- ---------- ---------- ---------- | 155 |
| XP_024586872.1 | ---------- ---------- ---------- ---------- ---------- ---------- ---------- ---------- | 182 |
| XP_024573052.1 | ---------- ---------- ---------- ---------- ---------- ---------- ---------- ---------- | 187 |
| XP_024586054.1 | ---------- ---------- ---------- ---------- ---------- ---------- ---------- ---------- | 237 |
| PvCRN27 | ---------- ---------- ---------- ---------- ---------- ---------- ---------- ---------- | 313 |
| PvCRN20 | ---------- ---------- ---------- ---------- ---------- ---------- ---------- ---------- | 159 |
| PvCRN25 | ---------- ---------- ---------- ---------- ---------- ---------- ---------- ---------- | 194 |
| PvCRN1 | ---------- ---------- ---------- ---------- ---------- ---------- ---------- ---------- | 580 |
| PvCRN4 | ---------- ---------- ---------- ---------- ---------- ---------- ---------- ---------- | 583 |
| PvCRN30 | ---------- ---------- ---------- ---------- ---------- ---------- ---------- ---------- | 220 |
| PvCRN29 | ---------- ---------- ---------- ---------- ---------- ---------- ---------- ---------- | 312 |
| XP_024583036.1 | ---------- ---------- ---------- ---------- ---------- ---------- ---------- ---------- | 681 |
| PvCRN10 | ---------- ---------- ---------- ---------- ---------- ---------- ---------- ---------- | 217 |
| XP_024577280.1 | ---------- ---------- ---------- ---------- ---------- ---------- ---------- ---------- | 283 |
| XP_024572924.1 | ---------- ---------- ---------- ---------- ---------- ---------- ---------- ---------- | 412 |
| PvCRN14 | ---------- ---------- ---------- ---------- ---------- ---------- ---------- ---------- | 151 |
| XP_024577521.1 | ---------- ---------- ---------- ---------- ---------- ---------- ---------- ---------- | 108 |
| PvCRN21 | ---------- ---------- ---------- ---------- ---------- ---------- ---------- ---------- | 479 |
| XP_024574966.1 | ---------- ---------- ---------- ---------- ---------- ---------- ---------- ---------- | 155 |
| XP_024575372.1 | ---------- ---------- ---------- ---------- ---------- ---------- ---------- ---------- | 188 |
| XP_024581363.1 | ---------- ---------- ---------- ---------- ---------- ---------- ---------- ---------- | 229 |
| XP_024583883.1 | ---------- ---------- ---------- ---------- ---------- ---------- ---------- ---------- | 381 |
| XP_024579844.1 | ---------- ---------- ---------- ---------- ---------- ---------- ---------- ---------- | 476 |
| XP_024574916.1 | ---------- ---------- ---------- ---------- ---------- ---------- ---------- ---------- | 621 |
| XP_024574193.1 | ---------- ---------- ---------- ---------- ---------- ---------- ---------- ---------- | 615 |
| XP_024583155.1 | ---------- ---------- ---------- ---------- ---------- ---------- ---------- ---------- | 419 |
| XP_024574913.1 | ---------- ---------- ---------- ---------- ---------- ---------- ---------- ---------- | 623 |
| XP_024578158.1 | ---------- ---------- ---------- ---------- ---------- ---------- ---------- ---------- | 106 |
| PvCRN22 | ---------- ---------- ---------- ---------- ---------- ---------- ---------- ---------- | 206 |
| XP_024585001.1 | ---------- ---------- ---------- ---------- ---------- ---------- ---------- ---------- | 127 |
| XP_024583843.1 | ---------- ---------- ---------- ---------- ---------- ---------- ---------- ---------- | 534 |
| XP_024578702.1 | ---------- ---------- ---------- ---------- ---------- ---------- ---------- ---------- | 299 |
| XP_024585805.1 | ---------- ---------- ---------- ---------- ---------- ---------- ---------- ---------- | 334 |
| XP_024580932.1 | ---------- ---------- ---------- ---------- ---------- ---------- ---------- ---------- | 508 |
| Consensus | ---------- ---------- ---------- ---------- ---------- ---------- ---------- ---------- | |
| Conservation (100% / 0%) | | |

| Sequence | 8,260 · 8,280 · 8,300 · 8,320 | |
|---|---|---|
| PvCRN11 | ---------- ---------- ---------- ---------- ---------- ---------- ---------- ---------- | 215 |
| XP_024579260.1 | ---------- ---------- ---------- ---------- ---------- ---------- ---------- ---------- | 163 |
| XP_024573634.1 | ---------- ---------- ---------- ---------- ---------- ---------- ---------- ---------- | 169 |
| PvCRN7 | ---------- ---------- ---------- ---------- ---------- ---------- ---------- ---------- | 753 |
| XP_024585265.1 | ---------- ---------- ---------- ---------- ---------- ---------- ---------- ---------- | 128 |
| PvCRN18 | ---------- ---------- ---------- ---------- ---------- ---------- ---------- ---------- | 302 |
| XP_024578414.1 | ---------- ---------- ---------- ---------- ---------- ---------- ---------- ---------- | 624 |
| XP_024579130.1 | ---------- ---------- ---------- ---------- ---------- ---------- ---------- ---------- | 345 |
| XP_024584758.1 | ---------- ---------- ---------- ---------- ---------- ---------- ---------- ---------- | 214 |
| XP_024586066.1 | ---------- ---------- ---------- ---------- ---------- ---------- ---------- ---------- | 263 |
| XP_024585870.1 | ---------- ---------- ---------- ---------- ---------- ---------- ---------- ---------- | 280 |
| PvCRN31 | ---------- ---------- ---------- ---------- ---------- ---------- ---------- ---------- | 583 |
| XP_024577278.1 | ---------- ---------- ---------- ---------- ---------- ---------- ---------- ---------- | 333 |
| XP_024585562.1 | ---------- ---------- ---------- ---------- ---------- ---------- ---------- ---------- | 158 |
| XP_024573944.1 | ---------- ---------- ---------- ---------- ---------- ---------- ---------- ---------- | 592 |
| XP_024579793.1 | ---------- ---------- ---------- ---------- ---------- ---------- ---------- ---------- | 163 |
| PvCRN15 | ---------- ---------- ---------- ---------- ---------- ---------- ---------- ---------- | 290 |
| PvCRN26 | ---------- ---------- ---------- ---------- ---------- ---------- ---------- ---------- | 279 |
| PvCRN16 | ---------- ---------- ---------- ---------- ---------- ---------- ---------- ---------- | 365 |
| PvCRN23 | ---------- ---------- ---------- ---------- ---------- ---------- ---------- ---------- | 426 |
| XP_024573063.1 | ---------- ---------- ---------- ---------- ---------- ---------- ---------- ---------- | 83 |
| PvCRN19 | ---------- ---------- ---------- ---------- ---------- ---------- ---------- ---------- | 164 |
| XP_024581625.1 | ---------- ---------- ---------- ---------- ---------- ---------- ---------- ---------- | 135 |
| XP_024583886.1 | ---------- ---------- ---------- ---------- ---------- ---------- ---------- ---------- | 96 |
| XP_024584409.1 | ---------- ---------- ---------- ---------- ---------- ---------- ---------- ---------- | 213 |
| XP_024572385.1 | ---------- ---------- ---------- ---------- ---------- ---------- ---------- ---------- | 310 |
| PvCRN6 | ---------- ---------- ---------- ---------- ---------- ---------- ---------- ---------- | 372 |
| PvCRN9 | ---------- ---------- ---------- ---------- ---------- ---------- ---------- ---------- | 611 |
| XP_024580875.1 | ---------- ---------- ---------- ---------- ---------- ---------- ---------- ---------- | 127 |
| PvCRN24 | ---------- ---------- ---------- ---------- ---------- ---------- ---------- ---------- | 165 |
| PvCRN35 | ---------- ---------- ---------- ---------- ---------- ---------- ---------- ---------- | 212 |
| PvCRN12 | ---------- ---------- ---------- ---------- ---------- ---------- ---------- ---------- | 171 |
| XP_024576927.1 | ---------- ---------- ---------- ---------- ---------- ---------- ---------- ---------- | 555 |
| XP_024578927.1 | ---------- ---------- ---------- ---------- ---------- ---------- ---------- ---------- | 327 |
| XP_024586212.1 | ---------- ---------- ---------- ---------- ---------- ---------- ---------- ---------- | 616 |
| XP_024578078.1 | ---------- ---------- ---------- ---------- ---------- ---------- ---------- ---------- | 262 |
| XP_024575355.1 | ---------- ---------- ---------- ---------- ---------- ---------- ---------- ---------- | 295 |
| XP_024584527.1 | ---------- ---------- ---------- ---------- ---------- ---------- ---------- ---------- | 168 |
| XP_024586664.1 | ---------- ---------- ---------- ---------- ---------- ---------- ---------- ---------- | 357 |
| PvCRN17 | ---------- ---------- ---------- ---------- ---------- ---------- ---------- ---------- | 187 |
| PvCRN2 | ---------- ---------- ---------- ---------- ---------- ---------- ---------- ---------- | 179 |
| XP_024577181.1 | ---------- ---------- ---------- ---------- ---------- ---------- ---------- ---------- | 207 |
| XP_024581075.1 | ---------- ---------- ---------- ---------- ---------- ---------- ---------- ---------- | 156 |
| XP_024575884.1 | ---------- ---------- ---------- ---------- ---------- ---------- ---------- ---------- | 155 |
| XP_024586872.1 | ---------- ---------- ---------- ---------- ---------- ---------- ---------- ---------- | 182 |
| XP_024573052.1 | ---------- ---------- ---------- ---------- ---------- ---------- ---------- ---------- | 187 |
| XP_024586054.1 | ---------- ---------- ---------- ---------- ---------- ---------- ---------- ---------- | 237 |
| PvCRN27 | ---------- ---------- ---------- ---------- ---------- ---------- ---------- ---------- | 313 |
| PvCRN20 | ---------- ---------- ---------- ---------- ---------- ---------- ---------- ---------- | 159 |
| PvCRN25 | ---------- ---------- ---------- ---------- ---------- ---------- ---------- ---------- | 194 |
| PvCRN1 | ---------- ---------- ---------- ---------- ---------- ---------- ---------- ---------- | 580 |
| PvCRN4 | ---------- ---------- ---------- ---------- ---------- ---------- ---------- ---------- | 583 |
| PvCRN30 | ---------- ---------- ---------- ---------- ---------- ---------- ---------- ---------- | 220 |
| PvCRN29 | ---------- ---------- ---------- ---------- ---------- ---------- ---------- ---------- | 312 |
| XP_024583036.1 | ---------- ---------- ---------- ---------- ---------- ---------- ---------- ---------- | 681 |
| PvCRN10 | ---------- ---------- ---------- ---------- ---------- ---------- ---------- ---------- | 217 |
| XP_024577280.1 | ---------- ---------- ---------- ---------- ---------- ---------- ---------- ---------- | 283 |
| XP_024572924.1 | ---------- ---------- ---------- ---------- ---------- ---------- ---------- ---------- | 412 |
| PvCRN14 | ---------- ---------- ---------- ---------- ---------- ---------- ---------- ---------- | 151 |
| XP_024577521.1 | ---------- ---------- ---------- ---------- ---------- ---------- ---------- ---------- | 108 |
| PvCRN21 | ---------- ---------- ---------- ---------- ---------- ---------- ---------- ---------- | 479 |
| XP_024574966.1 | ---------- ---------- ---------- ---------- ---------- ---------- ---------- ---------- | 155 |
| XP_024575372.1 | ---------- ---------- ---------- ---------- ---------- ---------- ---------- ---------- | 188 |
| XP_024581363.1 | ---------- ---------- ---------- ---------- ---------- ---------- ---------- ---------- | 229 |
| XP_024583883.1 | ---------- ---------- ---------- ---------- ---------- ---------- ---------- ---------- | 381 |
| XP_024579844.1 | ---------- ---------- ---------- ---------- ---------- ---------- ---------- ---------- | 476 |
| XP_024574916.1 | ---------- ---------- ---------- ---------- ---------- ---------- ---------- ---------- | 621 |
| XP_024574193.1 | ---------- ---------- ---------- ---------- ---------- ---------- ---------- ---------- | 615 |
| XP_024583155.1 | ---------- ---------- ---------- ---------- ---------- ---------- ---------- ---------- | 419 |
| XP_024574913.1 | ---------- ---------- ---------- ---------- ---------- ---------- ---------- ---------- | 623 |
| XP_024578158.1 | ---------- ---------- ---------- ---------- ---------- ---------- ---------- ---------- | 106 |
| PvCRN22 | ---------- ---------- ---------- ---------- ---------- ---------- ---------- ---------- | 206 |
| XP_024585001.1 | ---------- ---------- ---------- ---------- ---------- ---------- ---------- ---------- | 127 |
| XP_024583843.1 | ---------- ---------- ---------- ---------- ---------- ---------- ---------- ---------- | 534 |
| XP_024578702.1 | ---------- ---------- ---------- ---------- ---------- ---------- ---------- ---------- | 299 |
| XP_024585805.1 | ---------- ---------- ---------- ---------- ---------- ---------- ---------- ---------- | 334 |
| XP_024580932.1 | ---------- ---------- ---------- ---------- ---------- ---------- ---------- ---------- | 508 |
| Consensus | ---------- ---------- ---------- ---------- ---------- ---------- ---------- ---------- | |
| Conservation (100% / 0%) | | |

| | 8,340 8,360 8,380 8,400 | |
|---|---|---|
| PvCRN11 | ---------- ---------- ---------- ---------- ---------- ---------- ---------- ---------- | 215 |
| XP_024579260.1 | ---------- ---------- ---------- ---------- ---------- ---------- ---------- ---------- | 163 |
| XP_024573634.1 | ---------- ---------- ---------- ---------- ---------- ---------- ---------- ---------- | 169 |
| PvCRN7 | ---------- ---------- ---------- ---------- ---------- ---------- ---------- ---------- | 753 |
| XP_024585265.1 | ---------- ---------- ---------- ---------- ---------- ---------- ---------- ---------- | 128 |
| PvCRN18 | ---------- ---------- ---------- ---------- ---------- ---------- ---------- ---------- | 302 |
| XP_024578414.1 | ---------- ---------- ---------- ---------- ---------- ---------- ---------- ---------- | 624 |
| XP_024579130.1 | ---------- ---------- ---------- ---------- ---------- ---------- ---------- ---------- | 345 |
| XP_024584758.1 | ---------- ---------- ---------- ---------- ---------- ---------- ---------- ---------- | 214 |
| XP_024586066.1 | ---------- ---------- ---------- ---------- ---------- ---------- ---------- ---------- | 263 |
| XP_024585870.1 | ---------- ---------- ---------- ---------- ---------- ---------- ---------- ---------- | 280 |
| PvCRN31 | ---------- ---------- ---------- ---------- ---------- ---------- ---------- ---------- | 583 |
| XP_024577278.1 | ---------- ---------- ---------- ---------- ---------- ---------- ---------- ---------- | 333 |
| XP_024585562.1 | ---------- ---------- ---------- ---------- ---------- ---------- ---------- ---------- | 158 |
| XP_024573944.1 | ---------- ---------- ---------- ---------- ---------- ---------- ---------- ---------- | 592 |
| XP_024579793.1 | ---------- ---------- ---------- ---------- ---------- ---------- ---------- ---------- | 163 |
| PvCRN15 | ---------- ---------- ---------- ---------- ---------- ---------- ---------- ---------- | 290 |
| PvCRN26 | ---------- ---------- ---------- ---------- ---------- ---------- ---------- ---------- | 279 |
| PvCRN16 | ---------- ---------- ---------- ---------- ---------- ---------- ---------- ---------- | 365 |
| PvCRN23 | ---------- ---------- ---------- ---------- ---------- ---------- ---------- ---------- | 426 |
| XP_024573063.1 | ---------- ---------- ---------- ---------- ---------- ---------- ---------- ---------- | 83 |
| PvCRN19 | ---------- ---------- ---------- ---------- ---------- ---------- ---------- ---------- | 164 |
| XP_024581625.1 | ---------- ---------- ---------- ---------- ---------- ---------- ---------- ---------- | 135 |
| XP_024583886.1 | ---------- ---------- ---------- ---------- ---------- ---------- ---------- ---------- | 96 |
| XP_024584409.1 | ---------- ---------- ---------- ---------- ---------- ---------- ---------- ---------- | 213 |
| XP_024572385.1 | ---------- ---------- ---------- ---------- ---------- ---------- ---------- ---------- | 310 |
| PvCRN6 | ---------- ---------- ---------- ---------- ---------- ---------- ---------- ---------- | 372 |
| PvCRN9 | ---------- ---------- ---------- ---------- ---------- ---------- ---------- ---------- | 611 |
| XP_024580875.1 | ---------- ---------- ---------- ---------- ---------- ---------- ---------- ---------- | 127 |
| PvCRN24 | ---------- ---------- ---------- ---------- ---------- ---------- ---------- ---------- | 165 |
| PvCRN35 | ---------- ---------- ---------- ---------- ---------- ---------- ---------- ---------- | 212 |
| PvCRN12 | ---------- ---------- ---------- ---------- ---------- ---------- ---------- ---------- | 171 |
| XP_024576927.1 | ---------- ---------- ---------- ---------- ---------- ---------- ---------- ---------- | 555 |
| XP_024578927.1 | ---------- ---------- ---------- ---------- ---------- ---------- ---------- ---------- | 327 |
| XP_024586212.1 | ---------- ---------- ---------- ---------- ---------- ---------- ---------- ---------- | 616 |
| XP_024578078.1 | ---------- ---------- ---------- ---------- ---------- ---------- ---------- ---------- | 262 |
| XP_024575355.1 | ---------- ---------- ---------- ---------- ---------- ---------- ---------- ---------- | 295 |
| XP_024584527.1 | ---------- ---------- ---------- ---------- ---------- ---------- ---------- ---------- | 168 |
| XP_024586664.1 | ---------- ---------- ---------- ---------- ---------- ---------- ---------- ---------- | 357 |
| PvCRN17 | ---------- ---------- ---------- ---------- ---------- ---------- ---------- ---------- | 187 |
| PvCRN2 | ---------- ---------- ---------- ---------- ---------- ---------- ---------- ---------- | 179 |
| XP_024577181.1 | ---------- ---------- ---------- ---------- ---------- ---------- ---------- ---------- | 207 |
| XP_024581075.1 | ---------- ---------- ---------- ---------- ---------- ---------- ---------- ---------- | 156 |
| XP_024575884.1 | ---------- ---------- ---------- ---------- ---------- ---------- ---------- ---------- | 155 |
| XP_024586872.1 | ---------- ---------- ---------- ---------- ---------- ---------- ---------- ---------- | 182 |
| XP_024573052.1 | ---------- ---------- ---------- ---------- ---------- ---------- ---------- ---------- | 187 |
| XP_024586054.1 | ---------- ---------- ---------- ---------- ---------- ---------- ---------- ---------- | 237 |
| PvCRN27 | ---------- ---------- ---------- ---------- ---------- ---------- ---------- ---------- | 313 |
| PvCRN20 | ---------- ---------- ---------- ---------- ---------- ---------- ---------- ---------- | 159 |
| PvCRN25 | ---------- ---------- ---------- ---------- ---------- ---------- ---------- ---------- | 194 |
| PvCRN1 | ---------- ---------- ---------- ---------- ---------- ---------- ---------- ---------- | 580 |
| PvCRN4 | ---------- ---------- ---------- ---------- ---------- ---------- ---------- ---------- | 583 |
| PvCRN30 | ---------- ---------- ---------- ---------- ---------- ---------- ---------- ---------- | 220 |
| PvCRN29 | ---------- ---------- ---------- ---------- ---------- ---------- ---------- ---------- | 312 |
| XP_024583036.1 | ---------- ---------- ---------- ---------- ---------- ---------- ---------- ---------- | 681 |
| PvCRN10 | ---------- ---------- ---------- ---------- ---------- ---------- ---------- ---------- | 217 |
| XP_024577280.1 | ---------- ---------- ---------- ---------- ---------- ---------- ---------- ---------- | 283 |
| XP_024572924.1 | ---------- ---------- ---------- ---------- ---------- ---------- ---------- ---------- | 412 |
| PvCRN14 | ---------- ---------- ---------- ---------- ---------- ---------- ---------- ---------- | 151 |
| XP_024577521.1 | ---------- ---------- ---------- ---------- ---------- ---------- ---------- ---------- | 108 |
| PvCRN21 | ---------- ---------- ---------- ---------- ---------- ---------- ---------- ---------- | 479 |
| XP_024574966.1 | ---------- ---------- ---------- ---------- ---------- ---------- ---------- ---------- | 155 |
| XP_024575372.1 | ---------- ---------- ---------- ---------- ---------- ---------- ---------- ---------- | 188 |
| XP_024581363.1 | ---------- ---------- ---------- ---------- ---------- ---------- ---------- ---------- | 229 |
| XP_024583883.1 | ---------- ---------- ---------- ---------- ---------- ---------- ---------- ---------- | 381 |
| XP_024579844.1 | ---------- ---------- ---------- ---------- ---------- ---------- ---------- ---------- | 476 |
| XP_024574916.1 | ---------- ---------- ---------- ---------- ---------- ---------- ---------- ---------- | 621 |
| XP_024574193.1 | ---------- ---------- ---------- ---------- ---------- ---------- ---------- ---------- | 615 |
| XP_024583155.1 | ---------- ---------- ---------- ---------- ---------- ---------- ---------- ---------- | 419 |
| XP_024574913.1 | ---------- ---------- ---------- ---------- ---------- ---------- ---------- ---------- | 623 |
| XP_024578158.1 | ---------- ---------- ---------- ---------- ---------- ---------- ---------- ---------- | 106 |
| PvCRN22 | ---------- ---------- ---------- ---------- ---------- ---------- ---------- ---------- | 206 |
| XP_024585001.1 | ---------- ---------- ---------- ---------- ---------- ---------- ---------- ---------- | 127 |
| XP_024583843.1 | ---------- ---------- ---------- ---------- ---------- ---------- ---------- ---------- | 534 |
| XP_024578702.1 | ---------- ---------- ---------- ---------- ---------- ---------- ---------- ---------- | 299 |
| XP_024585805.1 | ---------- ---------- ---------- ---------- ---------- ---------- ---------- ---------- | 334 |
| XP_024580932.1 | ---------- ---------- ---------- ---------- ---------- ---------- ---------- ---------- | 508 |
| Consensus | ---------- ---------- ---------- ---------- ---------- ---------- ---------- ---------- | |
| Conservation (100% / 0%) | | |

| | 8,420 | 8,440 | 8,460 | 8,480 | |
|---|---|---|---|---|---|
| PvCRN11 | ---------- ---------- | ---------- ---------- | ---------- ---------- | ---------- ---------- | 215 |
| XP_024579260.1 | ---------- ---------- | ---------- ---------- | ---------- ---------- | ---------- ---------- | 163 |
| XP_024573634.1 | ---------- ---------- | ---------- ---------- | ---------- ---------- | ---------- ---------- | 169 |
| PvCRN7 | ---------- ---------- | ---------- ---------- | ---------- ---------- | ---------- ---------- | 753 |
| XP_024585265.1 | ---------- ---------- | ---------- ---------- | ---------- ---------- | ---------- ---------- | 128 |
| PvCRN18 | ---------- ---------- | ---------- ---------- | ---------- ---------- | ---------- ---------- | 302 |
| XP_024578414.1 | ---------- ---------- | ---------- ---------- | ---------- ---------- | ---------- ---------- | 624 |
| XP_024579130.1 | ---------- ---------- | ---------- ---------- | ---------- ---------- | ---------- ---------- | 345 |
| XP_024584758.1 | ---------- ---------- | ---------- ---------- | ---------- ---------- | ---------- ---------- | 214 |
| XP_024586066.1 | ---------- ---------- | ---------- ---------- | ---------- ---------- | ---------- ---------- | 263 |
| XP_024585870.1 | ---------- ---------- | ---------- ---------- | ---------- ---------- | ---------- ---------- | 280 |
| PvCRN31 | ---------- ---------- | ---------- ---------- | ---------- ---------- | ---------- ---------- | 583 |
| XP_024577278.1 | ---------- ---------- | ---------- ---------- | ---------- ---------- | ---------- ---------- | 333 |
| XP_024585562.1 | ---------- ---------- | ---------- ---------- | ---------- ---------- | ---------- ---------- | 158 |
| XP_024573944.1 | ---------- ---------- | ---------- ---------- | ---------- ---------- | ---------- ---------- | 592 |
| XP_024579793.1 | ---------- ---------- | ---------- ---------- | ---------- ---------- | ---------- ---------- | 163 |
| PvCRN15 | ---------- ---------- | ---------- ---------- | ---------- ---------- | ---------- ---------- | 290 |
| PvCRN26 | ---------- ---------- | ---------- ---------- | ---------- ---------- | ---------- ---------- | 279 |
| PvCRN16 | ---------- ---------- | ---------- ---------- | ---------- ---------- | ---------- ---------- | 365 |
| PvCRN23 | ---------- ---------- | ---------- ---------- | ---------- ---------- | ---------- ---------- | 426 |
| XP_024573063.1 | ---------- ---------- | ---------- ---------- | ---------- ---------- | ---------- ---------- | 83 |
| PvCRN19 | ---------- ---------- | ---------- ---------- | ---------- ---------- | ---------- ---------- | 164 |
| XP_024581625.1 | ---------- ---------- | ---------- ---------- | ---------- ---------- | ---------- ---------- | 135 |
| XP_024583886.1 | ---------- ---------- | ---------- ---------- | ---------- ---------- | ---------- ---------- | 96 |
| XP_024584409.1 | ---------- ---------- | ---------- ---------- | ---------- ---------- | ---------- ---------- | 213 |
| XP_024572385.1 | ---------- ---------- | ---------- ---------- | ---------- ---------- | ---------- ---------- | 310 |
| PvCRN6 | ---------- ---------- | ---------- ---------- | ---------- ---------- | ---------- ---------- | 372 |
| PvCRN9 | ---------- ---------- | ---------- ---------- | ---------- ---------- | ---------- ---------- | 611 |
| XP_024580875.1 | ---------- ---------- | ---------- ---------- | ---------- ---------- | ---------- ---------- | 127 |
| PvCRN24 | ---------- ---------- | ---------- ---------- | ---------- ---------- | ---------- ---------- | 165 |
| PvCRN35 | ---------- ---------- | ---------- ---------- | ---------- ---------- | ---------- ---------- | 212 |
| PvCRN12 | ---------- ---------- | ---------- ---------- | ---------- ---------- | ---------- ---------- | 171 |
| XP_024576927.1 | ---------- ---------- | ---------- ---------- | ---------- ---------- | ---------- ---------- | 555 |
| XP_024578927.1 | ---------- ---------- | ---------- ---------- | ---------- ---------- | ---------- ---------- | 327 |
| XP_024586212.1 | ---------- ---------- | ---------- ---------- | ---------- ---------- | ---------- ---------- | 616 |
| XP_024578078.1 | ---------- ---------- | ---------- ---------- | ---------- ---------- | ---------- ---------- | 262 |
| XP_024575355.1 | ---------- ---------- | ---------- ---------- | ---------- ---------- | ---------- ---------- | 295 |
| XP_024584527.1 | ---------- ---------- | ---------- ---------- | ---------- ---------- | ---------- ---------- | 168 |
| XP_024586664.1 | ---------- ---------- | ---------- ---------- | ---------- ---------- | ---------- ---------- | 357 |
| PvCRN17 | ---------- ---------- | ---------- ---------- | ---------- ---------- | ---------- ---------- | 187 |
| PvCRN2 | ---------- ---------- | ---------- ---------- | ---------- ---------- | ---------- ---------- | 179 |
| XP_024577181.1 | ---------- ---------- | ---------- ---------- | ---------- ---------- | ---------- ---------- | 207 |
| XP_024581075.1 | ---------- ---------- | ---------- ---------- | ---------- ---------- | ---------- ---------- | 156 |
| XP_024575884.1 | ---------- ---------- | ---------- ---------- | ---------- ---------- | ---------- ---------- | 155 |
| XP_024586872.1 | ---------- ---------- | ---------- ---------- | ---------- ---------- | ---------- ---------- | 182 |
| XP_024573052.1 | ---------- ---------- | ---------- ---------- | ---------- ---------- | ---------- ---------- | 187 |
| XP_024586054.1 | ---------- ---------- | ---------- ---------- | ---------- ---------- | ---------- ---------- | 237 |
| PvCRN27 | ---------- ---------- | ---------- ---------- | ---------- ---------- | ---------- ---------- | 313 |
| PvCRN20 | ---------- ---------- | ---------- ---------- | ---------- ---------- | ---------- ---------- | 159 |
| PvCRN25 | ---------- ---------- | ---------- ---------- | ---------- ---------- | ---------- ---------- | 194 |
| PvCRN1 | ---------- ---------- | ---------- ---------- | ---------- ---------- | ---------- ---------- | 580 |
| PvCRN4 | ---------- ---------- | ---------- ---------- | ---------- ---------- | ---------- ---------- | 583 |
| PvCRN30 | ---------- ---------- | ---------- ---------- | ---------- ---------- | ---------- ---------- | 220 |
| PvCRN29 | ---------- ---------- | ---------- ---------- | ---------- ---------- | ---------- ---------- | 312 |
| XP_024583036.1 | ---------- ---------- | ---------- ---------- | ---------- ---------- | ---------- ---------- | 681 |
| PvCRN10 | ---------- ---------- | ---------- ---------- | ---------- ---------- | ---------- ---------- | 217 |
| XP_024577280.1 | ---------- ---------- | ---------- ---------- | ---------- ---------- | ---------- ---------- | 283 |
| XP_024572924.1 | ---------- ---------- | ---------- ---------- | ---------- ---------- | ---------- ---------- | 412 |
| PvCRN14 | ---------- ---------- | ---------- ---------- | ---------- ---------- | ---------- ---------- | 151 |
| XP_024577521.1 | ---------- ---------- | ---------- ---------- | ---------- ---------- | ---------- ---------- | 108 |
| PvCRN21 | ---------- ---------- | ---------- ---------- | ---------- ---------- | ---------- ---------- | 479 |
| XP_024574966.1 | ---------- ---------- | ---------- ---------- | ---------- ---------- | ---------- ---------- | 155 |
| XP_024575372.1 | ---------- ---------- | ---------- ---------- | ---------- ---------- | ---------- ---------- | 188 |
| XP_024581363.1 | ---------- ---------- | ---------- ---------- | ---------- ---------- | ---------- ---------- | 229 |
| XP_024583883.1 | ---------- ---------- | ---------- ---------- | ---------- ---------- | ---------- ---------- | 381 |
| XP_024579844.1 | ---------- ---------- | ---------- ---------- | ---------- ---------- | ---------- ---------- | 476 |
| XP_024574916.1 | ---------- ---------- | ---------- ---------- | ---------- ---------- | ---------- ---------- | 621 |
| XP_024574193.1 | ---------- ---------- | ---------- ---------- | ---------- ---------- | ---------- ---------- | 615 |
| XP_024583155.1 | ---------- ---------- | ---------- ---------- | ---------- ---------- | ---------- ---------- | 419 |
| XP_024574913.1 | ---------- ---------- | ---------- ---------- | ---------- ---------- | ---------- ---------- | 623 |
| XP_024578158.1 | ---------- ---------- | ---------- ---------- | ---------- ---------- | ---------- ---------- | 106 |
| PvCRN22 | ---------- ---------- | ---------- ---------- | ---------- ---------- | ---------- ---------- | 206 |
| XP_024585001.1 | ---------- ---------- | ---------- ---------- | ---------- ---------- | ---------- ---------- | 127 |
| XP_024583843.1 | ---------- ---------- | ---------- ---------- | ---------- ---------- | ---------- ---------- | 534 |
| XP_024578702.1 | ---------- ---------- | ---------- ---------- | ---------- ---------- | ---------- ---------- | 299 |
| XP_024585805.1 | ---------- ---------- | ---------- ---------- | ---------- ---------- | ---------- ---------- | 334 |
| XP_024580932.1 | ---------- ---------- | ---------- ---------- | ---------- ---------- | ---------- ---------- | 508 |
| Consensus | ---------- ---------- | ---------- ---------- | ---------- ---------- | ---------- ---------- | |
| Conservation (100% / 0%) | | | | | |

```
                    8,500                8,520                8,540                8,560
                        |                    |                    |                    |
      PvCRN11 ---------- ---------- ---------- ---------- ---------- ---------- ---------- ---------- 215
XP_024579260.1 ---------- ---------- ---------- ---------- ---------- ---------- ---------- ---------- 163
XP_024573634.1 ---------- ---------- ---------- ---------- ---------- ---------- ---------- ---------- 169
       PvCRN7 ---------- ---------- ---------- ---------- ---------- ---------- ---------- ---------- 753
XP_024585265.1 ---------- ---------- ---------- ---------- ---------- ---------- ---------- ---------- 128
      PvCRN18 ---------- ---------- ---------- ---------- ---------- ---------- ---------- ---------- 302
XP_024578414.1 ---------- ---------- ---------- ---------- ---------- ---------- ---------- ---------- 624
XP_024579130.1 ---------- ---------- ---------- ---------- ---------- ---------- ---------- ---------- 345
XP_024584758.1 ---------- ---------- ---------- ---------- ---------- ---------- ---------- ---------- 214
XP_024586066.1 ---------- ---------- ---------- ---------- ---------- ---------- ---------- ---------- 263
XP_024585870.1 ---------- ---------- ---------- ---------- ---------- ---------- ---------- ---------- 280
      PvCRN31 ---------- ---------- ---------- ---------- ---------- ---------- ---------- ---------- 583
XP_024577278.1 ---------- ---------- ---------- ---------- ---------- ---------- ---------- ---------- 333
XP_024585562.1 ---------- ---------- ---------- ---------- ---------- ---------- ---------- ---------- 158
XP_024573944.1 ---------- ---------- ---------- ---------- ---------- ---------- ---------- ---------- 592
XP_024579793.1 ---------- ---------- ---------- ---------- ---------- ---------- ---------- ---------- 163
      PvCRN15 ---------- ---------- ---------- ---------- ---------- ---------- ---------- ---------- 290
      PvCRN26 ---------- ---------- ---------- ---------- ---------- ---------- ---------- ---------- 279
      PvCRN16 ---------- ---------- ---------- ---------- ---------- ---------- ---------- ---------- 365
      PvCRN23 ---------- ---------- ---------- ---------- ---------- ---------- ---------- ---------- 426
XP_024573063.1 ---------- ---------- ---------- ---------- ---------- ---------- ---------- ---------- 83
      PvCRN19 ---------- ---------- ---------- ---------- ---------- ---------- ---------- ---------- 164
XP_024581625.1 ---------- ---------- ---------- ---------- ---------- ---------- ---------- ---------- 135
XP_024583886.1 ---------- ---------- ---------- ---------- ---------- ---------- ---------- ---------- 96
XP_024584409.1 ---------- ---------- ---------- ---------- ---------- ---------- ---------- ---------- 213
XP_024572385.1 ---------- ---------- ---------- ---------- ---------- ---------- ---------- ---------- 310
       PvCRN6 ---------- ---------- ---------- ---------- ---------- ---------- ---------- ---------- 372
       PvCRN9 ---------- ---------- ---------- ---------- ---------- ---------- ---------- ---------- 611
XP_024580875.1 ---------- ---------- ---------- ---------- ---------- ---------- ---------- ---------- 127
      PvCRN24 ---------- ---------- ---------- ---------- ---------- ---------- ---------- ---------- 165
      PvCRN35 ---------- ---------- ---------- ---------- ---------- ---------- ---------- ---------- 212
      PvCRN12 ---------- ---------- ---------- ---------- ---------- ---------- ---------- ---------- 171
XP_024576927.1 ---------- ---------- ---------- ---------- ---------- ---------- ---------- ---------- 555
XP_024578927.1 ---------- ---------- ---------- ---------- ---------- ---------- ---------- ---------- 327
XP_024586212.1 ---------- ---------- ---------- ---------- ---------- ---------- ---------- ---------- 616
XP_024578078.1 ---------- ---------- ---------- ---------- ---------- ---------- ---------- ---------- 262
XP_024575355.1 ---------- ---------- ---------- ---------- ---------- ---------- ---------- ---------- 295
XP_024584527.1 ---------- ---------- ---------- ---------- ---------- ---------- ---------- ---------- 168
XP_024586664.1 ---------- ---------- ---------- ---------- ---------- ---------- ---------- ---------- 357
      PvCRN17 ---------- ---------- ---------- ---------- ---------- ---------- ---------- ---------- 187
       PvCRN2 ---------- ---------- ---------- ---------- ---------- ---------- ---------- ---------- 179
XP_024577181.1 ---------- ---------- ---------- ---------- ---------- ---------- ---------- ---------- 207
XP_024581075.1 ---------- ---------- ---------- ---------- ---------- ---------- ---------- ---------- 156
XP_024575884.1 ---------- ---------- ---------- ---------- ---------- ---------- ---------- ---------- 155
XP_024586872.1 ---------- ---------- ---------- ---------- ---------- ---------- ---------- ---------- 182
XP_024573052.1 ---------- ---------- ---------- ---------- ---------- ---------- ---------- ---------- 187
XP_024586054.1 ---------- ---------- ---------- ---------- ---------- ---------- ---------- ---------- 237
      PvCRN27 ---------- ---------- ---------- ---------- ---------- ---------- ---------- ---------- 313
      PvCRN20 ---------- ---------- ---------- ---------- ---------- ---------- ---------- ---------- 159
      PvCRN25 ---------- ---------- ---------- ---------- ---------- ---------- ---------- ---------- 194
       PvCRN1 ---------- ---------- ---------- ---------- ---------- ---------- ---------- ---------- 580
       PvCRN4 ---------- ---------- ---------- ---------- ---------- ---------- ---------- ---------- 583
      PvCRN30 ---------- ---------- ---------- ---------- ---------- ---------- ---------- ---------- 220
      PvCRN29 ---------- ---------- ---------- ---------- ---------- ---------- ---------- ---------- 312
XP_024583036.1 ---------- ---------- ---------- ---------- ---------- ---------- ---------- ---------- 681
      PvCRN10 ---------- ---------- ---------- ---------- ---------- ---------- ---------- ---------- 217
XP_024577280.1 ---------- ---------- ---------- ---------- ---------- ---------- ---------- ---------- 283
XP_024572924.1 ---------- ---------- ---------- ---------- ---------- ---------- ---------- ---------- 412
      PvCRN14 ---------- ---------- ---------- ---------- ---------- ---------- ---------- ---------- 151
XP_024577521.1 ---------- ---------- ---------- ---------- ---------- ---------- ---------- ---------- 108
      PvCRN21 ---------- ---------- ---------- ---------- ---------- ---------- ---------- ---------- 479
XP_024574966.1 ---------- ---------- ---------- ---------- ---------- ---------- ---------- ---------- 155
XP_024575372.1 ---------- ---------- ---------- ---------- ---------- ---------- ---------- ---------- 188
XP_024581363.1 ---------- ---------- ---------- ---------- ---------- ---------- ---------- ---------- 229
XP_024583883.1 ---------- ---------- ---------- ---------- ---------- ---------- ---------- ---------- 381
XP_024579844.1 ---------- ---------- ---------- ---------- ---------- ---------- ---------- ---------- 476
XP_024574916.1 ---------- ---------- ---------- ---------- ---------- ---------- ---------- ---------- 621
XP_024574193.1 ---------- ---------- ---------- ---------- ---------- ---------- ---------- ---------- 615
XP_024583155.1 ---------- ---------- ---------- ---------- ---------- ---------- ---------- ---------- 419
XP_024574913.1 ---------- ---------- ---------- ---------- ---------- ---------- ---------- ---------- 623
XP_024578158.1 ---------- ---------- ---------- ---------- ---------- ---------- ---------- ---------- 106
      PvCRN22 ---------- ---------- ---------- ---------- ---------- ---------- ---------- ---------- 206
XP_024585001.1 ---------- ---------- ---------- ---------- ---------- ---------- ---------- ---------- 127
XP_024583843.1 ---------- ---------- ---------- ---------- ---------- ---------- ---------- ---------- 534
XP_024578702.1 ---------- ---------- ---------- ---------- ---------- ---------- ---------- ---------- 299
XP_024585805.1 ---------- ---------- ---------- ---------- ---------- ---------- ---------- ---------- 334
XP_024580932.1 ---------- ---------- ---------- ---------- ---------- ---------- ---------- ---------- 508
    Consensus ---------- ---------- ---------- ---------- ---------- ---------- ---------- ----------
         100%
 Conservation
           0%
```

| | 8,580 | 8,600 | 8,620 | 8,640 |
|---|---|---|---|---|
| PvCRN11 | ---------- ---------- | ---------- ---------- | ---------- ---------- | ---------- ---------- 215 |
| XP_024579260.1 | ---------- ---------- | ---------- ---------- | ---------- ---------- | ---------- ---------- 163 |
| XP_024573634.1 | ---------- ---------- | ---------- ---------- | ---------- ---------- | ---------- ---------- 169 |
| PvCRN7 | ---------- ---------- | ---------- ---------- | ---------- ---------- | ---------- ---------- 753 |
| XP_024585265.1 | ---------- ---------- | ---------- ---------- | ---------- ---------- | ---------- ---------- 128 |
| PvCRN18 | ---------- ---------- | ---------- ---------- | ---------- ---------- | ---------- ---------- 302 |
| XP_024578414.1 | ---------- ---------- | ---------- ---------- | ---------- ---------- | ---------- ---------- 624 |
| XP_024579130.1 | ---------- ---------- | ---------- ---------- | ---------- ---------- | ---------- ---------- 345 |
| XP_024584758.1 | ---------- ---------- | ---------- ---------- | ---------- ---------- | ---------- ---------- 214 |
| XP_024586066.1 | ---------- ---------- | ---------- ---------- | ---------- ---------- | ---------- ---------- 263 |
| XP_024585870.1 | ---------- ---------- | ---------- ---------- | ---------- ---------- | ---------- ---------- 280 |
| PvCRN31 | ---------- ---------- | ---------- ---------- | ---------- ---------- | ---------- ---------- 583 |
| XP_024577278.1 | ---------- ---------- | ---------- ---------- | ---------- ---------- | ---------- ---------- 333 |
| XP_024585562.1 | ---------- ---------- | ---------- ---------- | ---------- ---------- | ---------- ---------- 158 |
| XP_024573944.1 | ---------- ---------- | ---------- ---------- | ---------- ---------- | ---------- ---------- 592 |
| XP_024579793.1 | ---------- ---------- | ---------- ---------- | ---------- ---------- | ---------- ---------- 163 |
| PvCRN15 | ---------- ---------- | ---------- ---------- | ---------- ---------- | ---------- ---------- 290 |
| PvCRN26 | ---------- ---------- | ---------- ---------- | ---------- ---------- | ---------- ---------- 279 |
| PvCRN16 | ---------- ---------- | ---------- ---------- | ---------- ---------- | ---------- ---------- 365 |
| PvCRN23 | ---------- ---------- | ---------- ---------- | ---------- ---------- | ---------- ---------- 426 |
| XP_024573063.1 | ---------- ---------- | ---------- ---------- | ---------- ---------- | ---------- ---------- 83 |
| PvCRN19 | ---------- ---------- | ---------- ---------- | ---------- ---------- | ---------- ---------- 164 |
| XP_024581625.1 | ---------- ---------- | ---------- ---------- | ---------- ---------- | ---------- ---------- 135 |
| XP_024583886.1 | ---------- ---------- | ---------- ---------- | ---------- ---------- | ---------- ---------- 96 |
| XP_024584409.1 | ---------- ---------- | ---------- ---------- | ---------- ---------- | ---------- ---------- 213 |
| XP_024572385.1 | ---------- ---------- | ---------- ---------- | ---------- ---------- | ---------- ---------- 310 |
| PvCRN6 | ---------- ---------- | ---------- ---------- | ---------- ---------- | ---------- ---------- 372 |
| PvCRN9 | ---------- ---------- | ---------- ---------- | ---------- ---------- | ---------- ---------- 611 |
| XP_024580875.1 | ---------- ---------- | ---------- ---------- | ---------- ---------- | ---------- ---------- 127 |
| PvCRN24 | ---------- ---------- | ---------- ---------- | ---------- ---------- | ---------- ---------- 165 |
| PvCRN35 | ---------- ---------- | ---------- ---------- | ---------- ---------- | ---------- ---------- 212 |
| PvCRN12 | ---------- ---------- | ---------- ---------- | ---------- ---------- | ---------- ---------- 171 |
| XP_024576927.1 | ---------- ---------- | ---------- ---------- | ---------- ---------- | ---------- ---------- 555 |
| XP_024578927.1 | ---------- ---------- | ---------- ---------- | ---------- ---------- | ---------- ---------- 327 |
| XP_024586212.1 | ---------- ---------- | ---------- ---------- | ---------- ---------- | ---------- ---------- 616 |
| XP_024578078.1 | ---------- ---------- | ---------- ---------- | ---------- ---------- | ---------- ---------- 262 |
| XP_024575355.1 | ---------- ---------- | ---------- ---------- | ---------- ---------- | ---------- ---------- 295 |
| XP_024584527.1 | ---------- ---------- | ---------- ---------- | ---------- ---------- | ---------- ---------- 168 |
| XP_024586664.1 | ---------- ---------- | ---------- ---------- | ---------- ---------- | ---------- ---------- 357 |
| PvCRN17 | ---------- ---------- | ---------- ---------- | ---------- ---------- | ---------- ---------- 187 |
| PvCRN2 | ---------- ---------- | ---------- ---------- | ---------- ---------- | ---------- ---------- 179 |
| XP_024577181.1 | ---------- ---------- | ---------- ---------- | ---------- ---------- | ---------- ---------- 207 |
| XP_024581075.1 | ---------- ---------- | ---------- ---------- | ---------- ---------- | ---------- ---------- 156 |
| XP_024575884.1 | ---------- ---------- | ---------- ---------- | ---------- ---------- | ---------- ---------- 155 |
| XP_024586872.1 | ---------- ---------- | ---------- ---------- | ---------- ---------- | ---------- ---------- 182 |
| XP_024573052.1 | ---------- ---------- | ---------- ---------- | ---------- ---------- | ---------- ---------- 187 |
| XP_024586054.1 | ---------- ---------- | ---------- ---------- | ---------- ---------- | ---------- ---------- 237 |
| PvCRN27 | ---------- ---------- | ---------- ---------- | ---------- ---------- | ---------- ---------- 313 |
| PvCRN20 | ---------- ---------- | ---------- ---------- | ---------- ---------- | ---------- ---------- 159 |
| PvCRN25 | ---------- ---------- | ---------- ---------- | ---------- ---------- | ---------- ---------- 194 |
| PvCRN1 | ---------- ---------- | ---------- ---------- | ---------- ---------- | ---------- ---------- 580 |
| PvCRN4 | ---------- ---------- | ---------- ---------- | ---------- ---------- | ---------- ---------- 583 |
| PvCRN30 | ---------- ---------- | ---------- ---------- | ---------- ---------- | ---------- ---------- 220 |
| PvCRN29 | ---------- ---------- | ---------- ---------- | ---------- ---------- | ---------- ---------- 312 |
| XP_024583036.1 | ---------- ---------- | ---------- ---------- | ---------- ---------- | ---------- ---------- 681 |
| PvCRN10 | ---------- ---------- | ---------- ---------- | ---------- ---------- | ---------- ---------- 217 |
| XP_024577280.1 | ---------- ---------- | ---------- ---------- | ---------- ---------- | ---------- ---------- 283 |
| XP_024572924.1 | ---------- ---------- | ---------- ---------- | ---------- ---------- | ---------- ---------- 412 |
| PvCRN14 | ---------- ---------- | ---------- ---------- | ---------- ---------- | ---------- ---------- 151 |
| XP_024577521.1 | ---------- ---------- | ---------- ---------- | ---------- ---------- | ---------- ---------- 108 |
| PvCRN21 | ---------- ---------- | ---------- ---------- | ---------- ---------- | ---------- ---------- 479 |
| XP_024574966.1 | ---------- ---------- | ---------- ---------- | ---------- ---------- | ---------- ---------- 155 |
| XP_024575372.1 | ---------- ---------- | ---------- ---------- | ---------- ---------- | ---------- ---------- 188 |
| XP_024581363.1 | ---------- ---------- | ---------- ---------- | ---------- ---------- | ---------- ---------- 229 |
| XP_024583883.1 | ---------- ---------- | ---------- ---------- | ---------- ---------- | ---------- ---------- 381 |
| XP_024579844.1 | ---------- ---------- | ---------- ---------- | ---------- ---------- | ---------- ---------- 476 |
| XP_024574916.1 | ---------- ---------- | ---------- ---------- | ---------- ---------- | ---------- ---------- 621 |
| XP_024574193.1 | ---------- ---------- | ---------- ---------- | ---------- ---------- | ---------- ---------- 615 |
| XP_024583155.1 | ---------- ---------- | ---------- ---------- | ---------- ---------- | ---------- ---------- 419 |
| XP_024574913.1 | ---------- ---------- | ---------- ---------- | ---------- ---------- | ---------- ---------- 623 |
| XP_024578158.1 | ---------- ---------- | ---------- ---------- | ---------- ---------- | ---------- ---------- 106 |
| PvCRN22 | ---------- ---------- | ---------- ---------- | ---------- ---------- | ---------- ---------- 206 |
| XP_024585001.1 | ---------- ---------- | ---------- ---------- | ---------- ---------- | ---------- ---------- 127 |
| XP_024583843.1 | ---------- ---------- | ---------- ---------- | ---------- ---------- | ---------- ---------- 534 |
| XP_024578702.1 | ---------- ---------- | ---------- ---------- | ---------- ---------- | ---------- ---------- 299 |
| XP_024585805.1 | ---------- ---------- | ---------- ---------- | ---------- ---------- | ---------- ---------- 334 |
| XP_024580932.1 | ---------- ---------- | ---------- ---------- | ---------- ---------- | ---------- ---------- 508 |
| Consensus | ---------- ---------- | ---------- ---------- | ---------- ---------- | ---------- ---------- |
| Conservation (100% / 0%) | | | | |

| | 8,660 8,680 8,700 8,720 | |
|---|---|---|
| PvCRN11 | ---------- ---------- ---------- ---------- ---------- ---------- ---------- ---------- | 215 |
| XP_024579260.1 | ---------- ---------- ---------- ---------- ---------- ---------- ---------- ---------- | 163 |
| XP_024573634.1 | ---------- ---------- ---------- ---------- ---------- ---------- ---------- ---------- | 169 |
| PvCRN7 | ---------- ---------- ---------- ---------- ---------- ---------- ---------- ---------- | 753 |
| XP_024585265.1 | ---------- ---------- ---------- ---------- ---------- ---------- ---------- ---------- | 128 |
| PvCRN18 | ---------- ---------- ---------- ---------- ---------- ---------- ---------- ---------- | 302 |
| XP_024578414.1 | ---------- ---------- ---------- ---------- ---------- ---------- ---------- ---------- | 624 |
| XP_024579130.1 | ---------- ---------- ---------- ---------- ---------- ---------- ---------- ---------- | 345 |
| XP_024584758.1 | ---------- ---------- ---------- ---------- ---------- ---------- ---------- ---------- | 214 |
| XP_024586066.1 | ---------- ---------- ---------- ---------- ---------- ---------- ---------- ---------- | 263 |
| XP_024585870.1 | ---------- ---------- ---------- ---------- ---------- ---------- ---------- ---------- | 280 |
| PvCRN31 | ---------- ---------- ---------- ---------- ---------- ---------- ---------- ---------- | 583 |
| XP_024577278.1 | ---------- ---------- ---------- ---------- ---------- ---------- ---------- ---------- | 333 |
| XP_024585562.1 | ---------- ---------- ---------- ---------- ---------- ---------- ---------- ---------- | 158 |
| XP_024573944.1 | ---------- ---------- ---------- ---------- ---------- ---------- ---------- ---------- | 592 |
| XP_024579793.1 | ---------- ---------- ---------- ---------- ---------- ---------- ---------- ---------- | 163 |
| PvCRN15 | ---------- ---------- ---------- ---------- ---------- ---------- ---------- ---------- | 290 |
| PvCRN26 | ---------- ---------- ---------- ---------- ---------- ---------- ---------- ---------- | 279 |
| PvCRN16 | ---------- ---------- ---------- ---------- ---------- ---------- ---------- ---------- | 365 |
| PvCRN23 | ---------- ---------- ---------- ---------- ---------- ---------- ---------- ---------- | 426 |
| XP_024573063.1 | ---------- ---------- ---------- ---------- ---------- ---------- ---------- ---------- | 83 |
| PvCRN19 | ---------- ---------- ---------- ---------- ---------- ---------- ---------- ---------- | 164 |
| XP_024581625.1 | ---------- ---------- ---------- ---------- ---------- ---------- ---------- ---------- | 135 |
| XP_024583886.1 | ---------- ---------- ---------- ---------- ---------- ---------- ---------- ---------- | 96 |
| XP_024584409.1 | ---------- ---------- ---------- ---------- ---------- ---------- ---------- ---------- | 213 |
| XP_024572385.1 | ---------- ---------- ---------- ---------- ---------- ---------- ---------- ---------- | 310 |
| PvCRN6 | ---------- ---------- ---------- ---------- ---------- ---------- ---------- ---------- | 372 |
| PvCRN9 | ---------- ---------- ---------- ---------- ---------- ---------- ---------- ---------- | 611 |
| XP_024580875.1 | ---------- ---------- ---------- ---------- ---------- ---------- ---------- ---------- | 127 |
| PvCRN24 | ---------- ---------- ---------- ---------- ---------- ---------- ---------- ---------- | 165 |
| PvCRN35 | ---------- ---------- ---------- ---------- ---------- ---------- ---------- ---------- | 212 |
| PvCRN12 | ---------- ---------- ---------- ---------- ---------- ---------- ---------- ---------- | 171 |
| XP_024576927.1 | ---------- ---------- ---------- ---------- ---------- ---------- ---------- ---------- | 555 |
| XP_024578927.1 | ---------- ---------- ---------- ---------- ---------- ---------- ---------- ---------- | 327 |
| XP_024586212.1 | ---------- ---------- ---------- ---------- ---------- ---------- ---------- ---------- | 616 |
| XP_024578078.1 | ---------- ---------- ---------- ---------- ---------- ---------- ---------- ---------- | 262 |
| XP_024575355.1 | ---------- ---------- ---------- ---------- ---------- ---------- ---------- ---------- | 295 |
| XP_024584527.1 | ---------- ---------- ---------- ---------- ---------- ---------- ---------- ---------- | 168 |
| XP_024586664.1 | ---------- ---------- ---------- ---------- ---------- ---------- ---------- ---------- | 357 |
| PvCRN17 | ---------- ---------- ---------- ---------- ---------- ---------- ---------- ---------- | 187 |
| PvCRN2 | ---------- ---------- ---------- ---------- ---------- ---------- ---------- ---------- | 179 |
| XP_024577181.1 | ---------- ---------- ---------- ---------- ---------- ---------- ---------- ---------- | 207 |
| XP_024581075.1 | ---------- ---------- ---------- ---------- ---------- ---------- ---------- ---------- | 156 |
| XP_024575884.1 | ---------- ---------- ---------- ---------- ---------- ---------- ---------- ---------- | 155 |
| XP_024586872.1 | ---------- ---------- ---------- ---------- ---------- ---------- ---------- ---------- | 182 |
| XP_024573052.1 | ---------- ---------- ---------- ---------- ---------- ---------- ---------- ---------- | 187 |
| XP_024586054.1 | ---------- ---------- ---------- ---------- ---------- ---------- ---------- ---------- | 237 |
| PvCRN27 | ---------- ---------- ---------- ---------- ---------- ---------- ---------- ---------- | 313 |
| PvCRN20 | ---------- ---------- ---------- ---------- ---------- ---------- ---------- ---------- | 159 |
| PvCRN25 | ---------- ---------- ---------- ---------- ---------- ---------- ---------- ---------- | 194 |
| PvCRN1 | ---------- ---------- ---------- ---------- ---------- ---------- ---------- ---------- | 580 |
| PvCRN4 | ---------- ---------- ---------- ---------- ---------- ---------- ---------- ---------- | 583 |
| PvCRN30 | ---------- ---------- ---------- ---------- ---------- ---------- ---------- ---------- | 220 |
| PvCRN29 | ---------- ---------- ---------- ---------- ---------- ---------- ---------- ---------- | 312 |
| XP_024583036.1 | ---------- ---------- ---------- ---------- ---------- ---------- ---------- ---------- | 681 |
| PvCRN10 | ---------- ---------- ---------- ---------- ---------- ---------- ---------- ---------- | 217 |
| XP_024577280.1 | ---------- ---------- ---------- ---------- ---------- ---------- ---------- ---------- | 283 |
| XP_024572924.1 | ---------- ---------- ---------- ---------- ---------- ---------- ---------- ---------- | 412 |
| PvCRN14 | ---------- ---------- ---------- ---------- ---------- ---------- ---------- ---------- | 151 |
| XP_024577521.1 | ---------- ---------- ---------- ---------- ---------- ---------- ---------- ---------- | 108 |
| PvCRN21 | ---------- ---------- ---------- ---------- ---------- ---------- ---------- ---------- | 479 |
| XP_024574966.1 | ---------- ---------- ---------- ---------- ---------- ---------- ---------- ---------- | 155 |
| XP_024575372.1 | ---------- ---------- ---------- ---------- ---------- ---------- ---------- ---------- | 188 |
| XP_024581363.1 | ---------- ---------- ---------- ---------- ---------- ---------- ---------- ---------- | 229 |
| XP_024583883.1 | ---------- ---------- ---------- ---------- ---------- ---------- ---------- ---------- | 381 |
| XP_024579844.1 | ---------- ---------- ---------- ---------- ---------- ---------- ---------- ---------- | 476 |
| XP_024574916.1 | ---------- ---------- ---------- ---------- ---------- ---------- ---------- ---------- | 621 |
| XP_024574193.1 | ---------- ---------- ---------- ---------- ---------- ---------- ---------- ---------- | 615 |
| XP_024583155.1 | ---------- ---------- ---------- ---------- ---------- ---------- ---------- ---------- | 419 |
| XP_024574913.1 | ---------- ---------- ---------- ---------- ---------- ---------- ---------- ---------- | 623 |
| XP_024578158.1 | ---------- ---------- ---------- ---------- ---------- ---------- ---------- ---------- | 106 |
| PvCRN22 | ---------- ---------- ---------- ---------- ---------- ---------- ---------- ---------- | 206 |
| XP_024585001.1 | ---------- ---------- ---------- ---------- ---------- ---------- ---------- ---------- | 127 |
| XP_024583843.1 | ---------- ---------- ---------- ---------- ---------- ---------- ---------- ---------- | 534 |
| XP_024578702.1 | ---------- ---------- ---------- ---------- ---------- ---------- ---------- ---------- | 299 |
| XP_024585805.1 | ---------- ---------- ---------- ---------- ---------- ---------- ---------- ---------- | 334 |
| XP_024580932.1 | ---------- ---------- ---------- ---------- ---------- ---------- ---------- ---------- | 508 |
| Consensus | ---------- ---------- ---------- ---------- ---------- ---------- ---------- ---------- | |
| Conservation (100% / 0%) | | |

| | 8,740 · 8,760 · 8,780 · 8,800 | |
|---|---|---|
| PvCRN11 | ---------- ---------- ---------- ---------- ---------- ---------- ---------- ---------- | 215 |
| XP_024579260.1 | ---------- ---------- ---------- ---------- ---------- ---------- ---------- ---------- | 163 |
| XP_024573634.1 | ---------- ---------- ---------- ---------- ---------- ---------- ---------- ---------- | 169 |
| PvCRN7 | ---------- ---------- ---------- ---------- ---------- ---------- ---------- ---------- | 753 |
| XP_024585265.1 | ---------- ---------- ---------- ---------- ---------- ---------- ---------- ---------- | 128 |
| PvCRN18 | ---------- ---------- ---------- ---------- ---------- ---------- ---------- ---------- | 302 |
| XP_024578414.1 | ---------- ---------- ---------- ---------- ---------- ---------- ---------- ---------- | 624 |
| XP_024579130.1 | ---------- ---------- ---------- ---------- ---------- ---------- ---------- ---------- | 345 |
| XP_024584758.1 | ---------- ---------- ---------- ---------- ---------- ---------- ---------- ---------- | 214 |
| XP_024586066.1 | ---------- ---------- ---------- ---------- ---------- ---------- ---------- ---------- | 263 |
| XP_024585870.1 | ---------- ---------- ---------- ---------- ---------- ---------- ---------- ---------- | 280 |
| PvCRN31 | ---------- ---------- ---------- ---------- ---------- ---------- ---------- ---------- | 583 |
| XP_024577278.1 | ---------- ---------- ---------- ---------- ---------- ---------- ---------- ---------- | 333 |
| XP_024585562.1 | ---------- ---------- ---------- ---------- ---------- ---------- ---------- ---------- | 158 |
| XP_024573944.1 | ---------- ---------- ---------- ---------- ---------- ---------- ---------- ---------- | 592 |
| XP_024579793.1 | ---------- ---------- ---------- ---------- ---------- ---------- ---------- ---------- | 163 |
| PvCRN15 | ---------- ---------- ---------- ---------- ---------- ---------- ---------- ---------- | 290 |
| PvCRN26 | ---------- ---------- ---------- ---------- ---------- ---------- ---------- ---------- | 279 |
| PvCRN16 | ---------- ---------- ---------- ---------- ---------- ---------- ---------- ---------- | 365 |
| PvCRN23 | ---------- ---------- ---------- ---------- ---------- ---------- ---------- ---------- | 426 |
| XP_024573063.1 | ---------- ---------- ---------- ---------- ---------- ---------- ---------- ---------- | 83 |
| PvCRN19 | ---------- ---------- ---------- ---------- ---------- ---------- ---------- ---------- | 164 |
| XP_024581625.1 | ---------- ---------- ---------- ---------- ---------- ---------- ---------- ---------- | 135 |
| XP_024583886.1 | ---------- ---------- ---------- ---------- ---------- ---------- ---------- ---------- | 96 |
| XP_024584409.1 | ---------- ---------- ---------- ---------- ---------- ---------- ---------- ---------- | 213 |
| XP_024572385.1 | ---------- ---------- ---------- ---------- ---------- ---------- ---------- ---------- | 310 |
| PvCRN6 | ---------- ---------- ---------- ---------- ---------- ---------- ---------- ---------- | 372 |
| PvCRN9 | ---------- ---------- ---------- ---------- ---------- ---------- ---------- ---------- | 611 |
| XP_024580875.1 | ---------- ---------- ---------- ---------- ---------- ---------- ---------- ---------- | 127 |
| PvCRN24 | ---------- ---------- ---------- ---------- ---------- ---------- ---------- ---------- | 165 |
| PvCRN35 | ---------- ---------- ---------- ---------- ---------- ---------- ---------- ---------- | 212 |
| PvCRN12 | ---------- ---------- ---------- ---------- ---------- ---------- ---------- ---------- | 171 |
| XP_024576927.1 | ---------- ---------- ---------- ---------- ---------- ---------- ---------- ---------- | 555 |
| XP_024578927.1 | ---------- ---------- ---------- ---------- ---------- ---------- ---------- ---------- | 327 |
| XP_024586212.1 | ---------- ---------- ---------- ---------- ---------- ---------- ---------- ---------- | 616 |
| XP_024578078.1 | ---------- ---------- ---------- ---------- ---------- ---------- ---------- ---------- | 262 |
| XP_024575355.1 | ---------- ---------- ---------- ---------- ---------- ---------- ---------- ---------- | 295 |
| XP_024584527.1 | ---------- ---------- ---------- ---------- ---------- ---------- ---------- ---------- | 168 |
| XP_024586664.1 | ---------- ---------- ---------- ---------- ---------- ---------- ---------- ---------- | 357 |
| PvCRN17 | ---------- ---------- ---------- ---------- ---------- ---------- ---------- ---------- | 187 |
| PvCRN2 | ---------- ---------- ---------- ---------- ---------- ---------- ---------- ---------- | 179 |
| XP_024577181.1 | ---------- ---------- ---------- ---------- ---------- ---------- ---------- ---------- | 207 |
| XP_024581075.1 | ---------- ---------- ---------- ---------- ---------- ---------- ---------- ---------- | 156 |
| XP_024575884.1 | ---------- ---------- ---------- ---------- ---------- ---------- ---------- ---------- | 155 |
| XP_024586872.1 | ---------- ---------- ---------- ---------- ---------- ---------- ---------- ---------- | 182 |
| XP_024573052.1 | ---------- ---------- ---------- ---------- ---------- ---------- ---------- ---------- | 187 |
| XP_024586054.1 | ---------- ---------- ---------- ---------- ---------- ---------- ---------- ---------- | 237 |
| PvCRN27 | ---------- ---------- ---------- ---------- ---------- ---------- ---------- ---------- | 313 |
| PvCRN20 | ---------- ---------- ---------- ---------- ---------- ---------- ---------- ---------- | 159 |
| PvCRN25 | ---------- ---------- ---------- ---------- ---------- ---------- ---------- ---------- | 194 |
| PvCRN1 | ---------- ---------- ---------- ---------- ---------- ---------- ---------- ---------- | 580 |
| PvCRN4 | ---------- ---------- ---------- ---------- ---------- ---------- ---------- ---------- | 583 |
| PvCRN30 | ---------- ---------- ---------- ---------- ---------- ---------- ---------- ---------- | 220 |
| PvCRN29 | ---------- ---------- ---------- ---------- ---------- ---------- ---------- ---------- | 312 |
| XP_024583036.1 | ---------- ---------- ---------- ---------- ---------- ---------- ---------- ---------- | 681 |
| PvCRN10 | ---------- ---------- ---------- ---------- ---------- ---------- ---------- ---------- | 217 |
| XP_024577280.1 | ---------- ---------- ---------- ---------- ---------- ---------- ---------- ---------- | 283 |
| XP_024572924.1 | ---------- ---------- ---------- ---------- ---------- ---------- ---------- ---------- | 412 |
| PvCRN14 | ---------- ---------- ---------- ---------- ---------- ---------- ---------- ---------- | 151 |
| XP_024577521.1 | ---------- ---------- ---------- ---------- ---------- ---------- ---------- ---------- | 108 |
| PvCRN21 | ---------- ---------- ---------- ---------- ---------- ---------- ---------- ---------- | 479 |
| XP_024574966.1 | ---------- ---------- ---------- ---------- ---------- ---------- ---------- ---------- | 155 |
| XP_024575372.1 | ---------- ---------- ---------- ---------- ---------- ---------- ---------- ---------- | 188 |
| XP_024581363.1 | ---------- ---------- ---------- ---------- ---------- ---------- ---------- ---------- | 229 |
| XP_024583883.1 | ---------- ---------- ---------- ---------- ---------- ---------- ---------- ---------- | 381 |
| XP_024579844.1 | ---------- ---------- ---------- ---------- ---------- ---------- ---------- ---------- | 476 |
| XP_024574916.1 | ---------- ---------- ---------- ---------- ---------- ---------- ---------- ---------- | 621 |
| XP_024574193.1 | ---------- ---------- ---------- ---------- ---------- ---------- ---------- ---------- | 615 |
| XP_024583155.1 | ---------- ---------- ---------- ---------- ---------- ---------- ---------- ---------- | 419 |
| XP_024574913.1 | ---------- ---------- ---------- ---------- ---------- ---------- ---------- ---------- | 623 |
| XP_024578158.1 | ---------- ---------- ---------- ---------- ---------- ---------- ---------- ---------- | 106 |
| PvCRN22 | ---------- ---------- ---------- ---------- ---------- ---------- ---------- ---------- | 206 |
| XP_024585001.1 | ---------- ---------- ---------- ---------- ---------- ---------- ---------- ---------- | 127 |
| XP_024583843.1 | ---------- ---------- ---------- ---------- ---------- ---------- ---------- ---------- | 534 |
| XP_024578702.1 | ---------- ---------- ---------- ---------- ---------- ---------- ---------- ---------- | 299 |
| XP_024585805.1 | ---------- ---------- ---------- ---------- ---------- ---------- ---------- ---------- | 334 |
| XP_024580932.1 | ---------- ---------- ---------- ---------- ---------- ---------- ---------- ---------- | 508 |
| Consensus | ---------- ---------- ---------- ---------- ---------- ---------- ---------- ---------- | |
| Conservation (100% / 0%) | | |

| | 8,820 · 8,840 · 8,860 · 8,880 | |
|---|---|---|
| PvCRN11 | ---------- ---------- ---------- ---------- ---------- ---------- ---------- ---------- | 215 |
| XP_024579260.1 | ---------- ---------- ---------- ---------- ---------- ---------- ---------- ---------- | 163 |
| XP_024573634.1 | ---------- ---------- ---------- ---------- ---------- ---------- ---------- ---------- | 169 |
| PvCRN7 | ---------- ---------- ---------- ---------- ---------- ---------- ---------- ---------- | 753 |
| XP_024585265.1 | ---------- ---------- ---------- ---------- ---------- ---------- ---------- ---------- | 128 |
| PvCRN18 | ---------- ---------- ---------- ---------- ---------- ---------- ---------- ---------- | 302 |
| XP_024578414.1 | ---------- ---------- ---------- ---------- ---------- ---------- ---------- ---------- | 624 |
| XP_024579130.1 | ---------- ---------- ---------- ---------- ---------- ---------- ---------- ---------- | 345 |
| XP_024584758.1 | ---------- ---------- ---------- ---------- ---------- ---------- ---------- ---------- | 214 |
| XP_024586066.1 | ---------- ---------- ---------- ---------- ---------- ---------- ---------- ---------- | 263 |
| XP_024585870.1 | ---------- ---------- ---------- ---------- ---------- ---------- ---------- ---------- | 280 |
| PvCRN31 | ---------- ---------- ---------- ---------- ---------- ---------- ---------- ---------- | 583 |
| XP_024577278.1 | ---------- ---------- ---------- ---------- ---------- ---------- ---------- ---------- | 333 |
| XP_024585562.1 | ---------- ---------- ---------- ---------- ---------- ---------- ---------- ---------- | 158 |
| XP_024573944.1 | ---------- ---------- ---------- ---------- ---------- ---------- ---------- ---------- | 592 |
| XP_024579793.1 | ---------- ---------- ---------- ---------- ---------- ---------- ---------- ---------- | 163 |
| PvCRN15 | ---------- ---------- ---------- ---------- ---------- ---------- ---------- ---------- | 290 |
| PvCRN26 | ---------- ---------- ---------- ---------- ---------- ---------- ---------- ---------- | 279 |
| PvCRN16 | ---------- ---------- ---------- ---------- ---------- ---------- ---------- ---------- | 365 |
| PvCRN23 | ---------- ---------- ---------- ---------- ---------- ---------- ---------- ---------- | 426 |
| XP_024573063.1 | ---------- ---------- ---------- ---------- ---------- ---------- ---------- ---------- | 83 |
| PvCRN19 | ---------- ---------- ---------- ---------- ---------- ---------- ---------- ---------- | 164 |
| XP_024581625.1 | ---------- ---------- ---------- ---------- ---------- ---------- ---------- ---------- | 135 |
| XP_024583886.1 | ---------- ---------- ---------- ---------- ---------- ---------- ---------- ---------- | 96 |
| XP_024584409.1 | ---------- ---------- ---------- ---------- ---------- ---------- ---------- ---------- | 213 |
| XP_024572385.1 | ---------- ---------- ---------- ---------- ---------- ---------- ---------- ---------- | 310 |
| PvCRN6 | ---------- ---------- ---------- ---------- ---------- ---------- ---------- ---------- | 372 |
| PvCRN9 | ---------- ---------- ---------- ---------- ---------- ---------- ---------- ---------- | 611 |
| XP_024580875.1 | ---------- ---------- ---------- ---------- ---------- ---------- ---------- ---------- | 127 |
| PvCRN24 | ---------- ---------- ---------- ---------- ---------- ---------- ---------- ---------- | 165 |
| PvCRN35 | ---------- ---------- ---------- ---------- ---------- ---------- ---------- ---------- | 212 |
| PvCRN12 | ---------- ---------- ---------- ---------- ---------- ---------- ---------- ---------- | 171 |
| XP_024576927.1 | ---------- ---------- ---------- ---------- ---------- ---------- ---------- ---------- | 555 |
| XP_024578927.1 | ---------- ---------- ---------- ---------- ---------- ---------- ---------- ---------- | 327 |
| XP_024586212.1 | ---------- ---------- ---------- ---------- ---------- ---------- ---------- ---------- | 616 |
| XP_024578078.1 | ---------- ---------- ---------- ---------- ---------- ---------- ---------- ---------- | 262 |
| XP_024575355.1 | ---------- ---------- ---------- ---------- ---------- ---------- ---------- ---------- | 295 |
| XP_024584527.1 | ---------- ---------- ---------- ---------- ---------- ---------- ---------- ---------- | 168 |
| XP_024586664.1 | ---------- ---------- ---------- ---------- ---------- ---------- ---------- ---------- | 357 |
| PvCRN17 | ---------- ---------- ---------- ---------- ---------- ---------- ---------- ---------- | 187 |
| PvCRN2 | ---------- ---------- ---------- ---------- ---------- ---------- ---------- ---------- | 179 |
| XP_024577181.1 | ---------- ---------- ---------- ---------- ---------- ---------- ---------- ---------- | 207 |
| XP_024581075.1 | ---------- ---------- ---------- ---------- ---------- ---------- ---------- ---------- | 156 |
| XP_024575884.1 | ---------- ---------- ---------- ---------- ---------- ---------- ---------- ---------- | 155 |
| XP_024586872.1 | ---------- ---------- ---------- ---------- ---------- ---------- ---------- ---------- | 182 |
| XP_024573052.1 | ---------- ---------- ---------- ---------- ---------- ---------- ---------- ---------- | 187 |
| XP_024586054.1 | ---------- ---------- ---------- ---------- ---------- ---------- ---------- ---------- | 237 |
| PvCRN27 | ---------- ---------- ---------- ---------- ---------- ---------- ---------- ---------- | 313 |
| PvCRN20 | ---------- ---------- ---------- ---------- ---------- ---------- ---------- ---------- | 159 |
| PvCRN25 | ---------- ---------- ---------- ---------- ---------- ---------- ---------- ---------- | 194 |
| PvCRN1 | ---------- ---------- ---------- ---------- ---------- ---------- ---------- ---------- | 580 |
| PvCRN4 | ---------- ---------- ---------- ---------- ---------- ---------- ---------- ---------- | 583 |
| PvCRN30 | ---------- ---------- ---------- ---------- ---------- ---------- ---------- ---------- | 220 |
| PvCRN29 | ---------- ---------- ---------- ---------- ---------- ---------- ---------- ---------- | 312 |
| XP_024583036.1 | ---------- ---------- ---------- ---------- ---------- ---------- ---------- ---------- | 681 |
| PvCRN10 | ---------- ---------- ---------- ---------- ---------- ---------- ---------- ---------- | 217 |
| XP_024577280.1 | ---------- ---------- ---------- ---------- ---------- ---------- ---------- ---------- | 283 |
| XP_024572924.1 | ---------- ---------- ---------- ---------- ---------- ---------- ---------- ---------- | 412 |
| PvCRN14 | ---------- ---------- ---------- ---------- ---------- ---------- ---------- ---------- | 151 |
| XP_024577521.1 | ---------- ---------- ---------- ---------- ---------- ---------- ---------- ---------- | 108 |
| PvCRN21 | ---------- ---------- ---------- ---------- ---------- ---------- ---------- ---------- | 479 |
| XP_024574966.1 | ---------- ---------- ---------- ---------- ---------- ---------- ---------- ---------- | 155 |
| XP_024575372.1 | ---------- ---------- ---------- ---------- ---------- ---------- ---------- ---------- | 188 |
| XP_024581363.1 | ---------- ---------- ---------- ---------- ---------- ---------- ---------- ---------- | 229 |
| XP_024583883.1 | ---------- ---------- ---------- ---------- ---------- ---------- ---------- ---------- | 381 |
| XP_024579844.1 | ---------- ---------- ---------- ---------- ---------- ---------- ---------- ---------- | 476 |
| XP_024574916.1 | ---------- ---------- ---------- ---------- ---------- ---------- ---------- ---------- | 621 |
| XP_024574193.1 | ---------- ---------- ---------- ---------- ---------- ---------- ---------- ---------- | 615 |
| XP_024583155.1 | ---------- ---------- ---------- ---------- ---------- ---------- ---------- ---------- | 419 |
| XP_024574913.1 | ---------- ---------- ---------- ---------- ---------- ---------- ---------- ---------- | 623 |
| XP_024578158.1 | ---------- ---------- ---------- ---------- ---------- ---------- ---------- ---------- | 106 |
| PvCRN22 | ---------- ---------- ---------- ---------- ---------- ---------- ---------- ---------- | 206 |
| XP_024585001.1 | ---------- ---------- ---------- ---------- ---------- ---------- ---------- ---------- | 127 |
| XP_024583843.1 | ---------- ---------- ---------- ---------- ---------- ---------- ---------- ---------- | 534 |
| XP_024578702.1 | ---------- ---------- ---------- ---------- ---------- ---------- ---------- ---------- | 299 |
| XP_024585805.1 | ---------- ---------- ---------- ---------- ---------- ---------- ---------- ---------- | 334 |
| XP_024580932.1 | ---------- ---------- ---------- ---------- ---------- ---------- ---------- ---------- | 508 |
| Consensus | ---------- ---------- ---------- ---------- ---------- ---------- ---------- ---------- | |
| Conservation (100% – 0%) | | |

| | 8,900 · 8,920 · 8,940 · 8,960 | |
|---|---|---|
| PvCRN11 | ---------- ---------- ---------- ---------- ---------- ---------- ---------- ---------- | 215 |
| XP_024579260.1 | ---------- ---------- ---------- ---------- ---------- ---------- ---------- ---------- | 163 |
| XP_024573634.1 | ---------- ---------- ---------- ---------- ---------- ---------- ---------- ---------- | 169 |
| PvCRN7 | ---------- ---------- ---------- ---------- ---------- ---------- ---------- ---------- | 753 |
| XP_024585265.1 | ---------- ---------- ---------- ---------- ---------- ---------- ---------- ---------- | 128 |
| PvCRN18 | ---------- ---------- ---------- ---------- ---------- ---------- ---------- ---------- | 302 |
| XP_024578414.1 | ---------- ---------- ---------- ---------- ---------- ---------- ---------- ---------- | 624 |
| XP_024579130.1 | ---------- ---------- ---------- ---------- ---------- ---------- ---------- ---------- | 345 |
| XP_024584758.1 | ---------- ---------- ---------- ---------- ---------- ---------- ---------- ---------- | 214 |
| XP_024586066.1 | ---------- ---------- ---------- ---------- ---------- ---------- ---------- ---------- | 263 |
| XP_024585870.1 | ---------- ---------- ---------- ---------- ---------- ---------- ---------- ---------- | 280 |
| PvCRN31 | ---------- ---------- ---------- ---------- ---------- ---------- ---------- ---------- | 583 |
| XP_024577278.1 | ---------- ---------- ---------- ---------- ---------- ---------- ---------- ---------- | 333 |
| XP_024585562.1 | ---------- ---------- ---------- ---------- ---------- ---------- ---------- ---------- | 158 |
| XP_024573944.1 | ---------- ---------- ---------- ---------- ---------- ---------- ---------- ---------- | 592 |
| XP_024579793.1 | ---------- ---------- ---------- ---------- ---------- ---------- ---------- ---------- | 163 |
| PvCRN15 | ---------- ---------- ---------- ---------- ---------- ---------- ---------- ---------- | 290 |
| PvCRN26 | ---------- ---------- ---------- ---------- ---------- ---------- ---------- ---------- | 279 |
| PvCRN16 | ---------- ---------- ---------- ---------- ---------- ---------- ---------- ---------- | 365 |
| PvCRN23 | ---------- ---------- ---------- ---------- ---------- ---------- ---------- ---------- | 426 |
| XP_024573063.1 | ---------- ---------- ---------- ---------- ---------- ---------- ---------- ---------- | 83 |
| PvCRN19 | ---------- ---------- ---------- ---------- ---------- ---------- ---------- ---------- | 164 |
| XP_024581625.1 | ---------- ---------- ---------- ---------- ---------- ---------- ---------- ---------- | 135 |
| XP_024583886.1 | ---------- ---------- ---------- ---------- ---------- ---------- ---------- ---------- | 96 |
| XP_024584409.1 | ---------- ---------- ---------- ---------- ---------- ---------- ---------- ---------- | 213 |
| XP_024572385.1 | ---------- ---------- ---------- ---------- ---------- ---------- ---------- ---------- | 310 |
| PvCRN6 | ---------- ---------- ---------- ---------- ---------- ---------- ---------- ---------- | 372 |
| PvCRN9 | ---------- ---------- ---------- ---------- ---------- ---------- ---------- ---------- | 611 |
| XP_024580875.1 | ---------- ---------- ---------- ---------- ---------- ---------- ---------- ---------- | 127 |
| PvCRN24 | ---------- ---------- ---------- ---------- ---------- ---------- ---------- ---------- | 165 |
| PvCRN35 | ---------- ---------- ---------- ---------- ---------- ---------- ---------- ---------- | 212 |
| PvCRN12 | ---------- ---------- ---------- ---------- ---------- ---------- ---------- ---------- | 171 |
| XP_024576927.1 | ---------- ---------- ---------- ---------- ---------- ---------- ---------- ---------- | 555 |
| XP_024578927.1 | ---------- ---------- ---------- ---------- ---------- ---------- ---------- ---------- | 327 |
| XP_024586212.1 | ---------- ---------- ---------- ---------- ---------- ---------- ---------- ---------- | 616 |
| XP_024578078.1 | ---------- ---------- ---------- ---------- ---------- ---------- ---------- ---------- | 262 |
| XP_024575355.1 | ---------- ---------- ---------- ---------- ---------- ---------- ---------- ---------- | 295 |
| XP_024584527.1 | ---------- ---------- ---------- ---------- ---------- ---------- ---------- ---------- | 168 |
| XP_024586664.1 | ---------- ---------- ---------- ---------- ---------- ---------- ---------- ---------- | 357 |
| PvCRN17 | ---------- ---------- ---------- ---------- ---------- ---------- ---------- ---------- | 187 |
| PvCRN2 | ---------- ---------- ---------- ---------- ---------- ---------- ---------- ---------- | 179 |
| XP_024577181.1 | ---------- ---------- ---------- ---------- ---------- ---------- ---------- ---------- | 207 |
| XP_024581075.1 | ---------- ---------- ---------- ---------- ---------- ---------- ---------- ---------- | 156 |
| XP_024575884.1 | ---------- ---------- ---------- ---------- ---------- ---------- ---------- ---------- | 155 |
| XP_024586872.1 | ---------- ---------- ---------- ---------- ---------- ---------- ---------- ---------- | 182 |
| XP_024573052.1 | ---------- ---------- ---------- ---------- ---------- ---------- ---------- ---------- | 187 |
| XP_024586054.1 | ---------- ---------- ---------- ---------- ---------- ---------- ---------- ---------- | 237 |
| PvCRN27 | ---------- ---------- ---------- ---------- ---------- ---------- ---------- ---------- | 313 |
| PvCRN20 | ---------- ---------- ---------- ---------- ---------- ---------- ---------- ---------- | 159 |
| PvCRN25 | ---------- ---------- ---------- ---------- ---------- ---------- ---------- ---------- | 194 |
| PvCRN1 | ---------- ---------- ---------- ---------- ---------- ---------- ---------- ---------- | 580 |
| PvCRN4 | ---------- ---------- ---------- ---------- ---------- ---------- ---------- ---------- | 583 |
| PvCRN30 | ---------- ---------- ---------- ---------- ---------- ---------- ---------- ---------- | 220 |
| PvCRN29 | ---------- ---------- ---------- ---------- ---------- ---------- ---------- ---------- | 312 |
| XP_024583036.1 | ---------- ---------- ---------- ---------- ---------- ---------- ---------- ---------- | 681 |
| PvCRN10 | ---------- ---------- ---------- ---------- ---------- ---------- ---------- ---------- | 217 |
| XP_024577280.1 | ---------- ---------- ---------- ---------- ---------- ---------- ---------- ---------- | 283 |
| XP_024572924.1 | ---------- ---------- ---------- ---------- ---------- ---------- ---------- ---------- | 412 |
| PvCRN14 | ---------- ---------- ---------- ---------- ---------- ---------- ---------- ---------- | 151 |
| XP_024577521.1 | ---------- ---------- ---------- ---------- ---------- ---------- ---------- ---------- | 108 |
| PvCRN21 | ---------- ---------- ---------- ---------- ---------- ---------- ---------- ---------- | 479 |
| XP_024574966.1 | ---------- ---------- ---------- ---------- ---------- ---------- ---------- ---------- | 155 |
| XP_024575372.1 | ---------- ---------- ---------- ---------- ---------- ---------- ---------- ---------- | 188 |
| XP_024581363.1 | ---------- ---------- ---------- ---------- ---------- ---------- ---------- ---------- | 229 |
| XP_024583883.1 | ---------- ---------- ---------- ---------- ---------- ---------- ---------- ---------- | 381 |
| XP_024579844.1 | ---------- ---------- ---------- ---------- ---------- ---------- ---------- ---------- | 476 |
| XP_024574916.1 | ---------- ---------- ---------- ---------- ---------- ---------- ---------- ---------- | 621 |
| XP_024574193.1 | ---------- ---------- ---------- ---------- ---------- ---------- ---------- ---------- | 615 |
| XP_024583155.1 | ---------- ---------- ---------- ---------- ---------- ---------- ---------- ---------- | 419 |
| XP_024574913.1 | ---------- ---------- ---------- ---------- ---------- ---------- ---------- ---------- | 623 |
| XP_024578158.1 | ---------- ---------- ---------- ---------- ---------- ---------- ---------- ---------- | 106 |
| PvCRN22 | ---------- ---------- ---------- ---------- ---------- ---------- ---------- ---------- | 206 |
| XP_024585001.1 | ---------- ---------- ---------- ---------- ---------- ---------- ---------- ---------- | 127 |
| XP_024583843.1 | ---------- ---------- ---------- ---------- ---------- ---------- ---------- ---------- | 534 |
| XP_024578702.1 | ---------- ---------- ---------- ---------- ---------- ---------- ---------- ---------- | 299 |
| XP_024585805.1 | ---------- ---------- ---------- ---------- ---------- ---------- ---------- ---------- | 334 |
| XP_024580932.1 | ---------- ---------- ---------- ---------- ---------- ---------- ---------- ---------- | 508 |
| Consensus | ---------- ---------- ---------- ---------- ---------- ---------- ---------- ---------- | |
| Conservation (100% / 0%) | | |

| | 8,980 · 9,000 · 9,020 · 9,040 | |
|---|---|---|
| PvCRN11 | ---------- ---------- ---------- ---------- ---------- ---------- ---------- ---------- | 215 |
| XP_024579260.1 | ---------- ---------- ---------- ---------- ---------- ---------- ---------- ---------- | 163 |
| XP_024573634.1 | ---------- ---------- ---------- ---------- ---------- ---------- ---------- ---------- | 169 |
| PvCRN7 | ---------- ---------- ---------- ---------- ---------- ---------- ---------- ---------- | 753 |
| XP_024585265.1 | ---------- ---------- ---------- ---------- ---------- ---------- ---------- ---------- | 128 |
| PvCRN18 | ---------- ---------- ---------- ---------- ---------- ---------- ---------- ---------- | 302 |
| XP_024578414.1 | ---------- ---------- ---------- ---------- ---------- ---------- ---------- ---------- | 624 |
| XP_024579130.1 | ---------- ---------- ---------- ---------- ---------- ---------- ---------- ---------- | 345 |
| XP_024584758.1 | ---------- ---------- ---------- ---------- ---------- ---------- ---------- ---------- | 214 |
| XP_024586066.1 | ---------- ---------- ---------- ---------- ---------- ---------- ---------- ---------- | 263 |
| XP_024585870.1 | ---------- ---------- ---------- ---------- ---------- ---------- ---------- ---------- | 280 |
| PvCRN31 | ---------- ---------- ---------- ---------- ---------- ---------- ---------- ---------- | 583 |
| XP_024577278.1 | ---------- ---------- ---------- ---------- ---------- ---------- ---------- ---------- | 333 |
| XP_024585562.1 | ---------- ---------- ---------- ---------- ---------- ---------- ---------- ---------- | 158 |
| XP_024573944.1 | ---------- ---------- ---------- ---------- ---------- ---------- ---------- ---------- | 592 |
| XP_024579793.1 | ---------- ---------- ---------- ---------- ---------- ---------- ---------- ---------- | 163 |
| PvCRN15 | ---------- ---------- ---------- ---------- ---------- ---------- ---------- ---------- | 290 |
| PvCRN26 | ---------- ---------- ---------- ---------- ---------- ---------- ---------- ---------- | 279 |
| PvCRN16 | ---------- ---------- ---------- ---------- ---------- ---------- ---------- ---------- | 365 |
| PvCRN23 | ---------- ---------- ---------- ---------- ---------- ---------- ---------- ---------- | 426 |
| XP_024573063.1 | ---------- ---------- ---------- ---------- ---------- ---------- ---------- ---------- | 83 |
| PvCRN19 | ---------- ---------- ---------- ---------- ---------- ---------- ---------- ---------- | 164 |
| XP_024581625.1 | ---------- ---------- ---------- ---------- ---------- ---------- ---------- ---------- | 135 |
| XP_024583886.1 | ---------- ---------- ---------- ---------- ---------- ---------- ---------- ---------- | 96 |
| XP_024584409.1 | ---------- ---------- ---------- ---------- ---------- ---------- ---------- ---------- | 213 |
| XP_024572385.1 | ---------- ---------- ---------- ---------- ---------- ---------- ---------- ---------- | 310 |
| PvCRN6 | ---------- ---------- ---------- ---------- ---------- ---------- ---------- ---------- | 372 |
| PvCRN9 | ---------- ---------- ---------- ---------- ---------- ---------- ---------- ---------- | 611 |
| XP_024580875.1 | ---------- ---------- ---------- ---------- ---------- ---------- ---------- ---------- | 127 |
| PvCRN24 | ---------- ---------- ---------- ---------- ---------- ---------- ---------- ---------- | 165 |
| PvCRN35 | ---------- ---------- ---------- ---------- ---------- ---------- ---------- ---------- | 212 |
| PvCRN12 | ---------- ---------- ---------- ---------- ---------- ---------- ---------- ---------- | 171 |
| XP_024576927.1 | ---------- ---------- ---------- ---------- ---------- ---------- ---------- ---------- | 555 |
| XP_024578927.1 | ---------- ---------- ---------- ---------- ---------- ---------- ---------- ---------- | 327 |
| XP_024586212.1 | ---------- ---------- ---------- ---------- ---------- ---------- ---------- ---------- | 616 |
| XP_024578078.1 | ---------- ---------- ---------- ---------- ---------- ---------- ---------- ---------- | 262 |
| XP_024575355.1 | ---------- ---------- ---------- ---------- ---------- ---------- ---------- ---------- | 295 |
| XP_024584527.1 | ---------- ---------- ---------- ---------- ---------- ---------- ---------- ---------- | 168 |
| XP_024586664.1 | ---------- ---------- ---------- ---------- ---------- ---------- ---------- ---------- | 357 |
| PvCRN17 | ---------- ---------- ---------- ---------- ---------- ---------- ---------- ---------- | 187 |
| PvCRN2 | ---------- ---------- ---------- ---------- ---------- ---------- ---------- ---------- | 179 |
| XP_024577181.1 | ---------- ---------- ---------- ---------- ---------- ---------- ---------- ---------- | 207 |
| XP_024581075.1 | ---------- ---------- ---------- ---------- ---------- ---------- ---------- ---------- | 156 |
| XP_024575884.1 | ---------- ---------- ---------- ---------- ---------- ---------- ---------- ---------- | 155 |
| XP_024586872.1 | ---------- ---------- ---------- ---------- ---------- ---------- ---------- ---------- | 182 |
| XP_024573052.1 | ---------- ---------- ---------- ---------- ---------- ---------- ---------- ---------- | 187 |
| XP_024586054.1 | ---------- ---------- ---------- ---------- ---------- ---------- ---------- ---------- | 237 |
| PvCRN27 | ---------- ---------- ---------- ---------- ---------- ---------- ---------- ---------- | 313 |
| PvCRN20 | ---------- ---------- ---------- ---------- ---------- ---------- ---------- ---------- | 159 |
| PvCRN25 | ---------- ---------- ---------- ---------- ---------- ---------- ---------- ---------- | 194 |
| PvCRN1 | ---------- ---------- ---------- ---------- ---------- ---------- ---------- ---------- | 580 |
| PvCRN4 | ---------- ---------- ---------- ---------- ---------- ---------- ---------- ---------- | 583 |
| PvCRN30 | ---------- ---------- ---------- ---------- ---------- ---------- ---------- ---------- | 220 |
| PvCRN29 | ---------- ---------- ---------- ---------- ---------- ---------- ---------- ---------- | 312 |
| XP_024583036.1 | ---------- ---------- ---------- ---------- ---------- ---------- ---------- ---------- | 681 |
| PvCRN10 | ---------- ---------- ---------- ---------- ---------- ---------- ---------- ---------- | 217 |
| XP_024577280.1 | ---------- ---------- ---------- ---------- ---------- ---------- ---------- ---------- | 283 |
| XP_024572924.1 | ---------- ---------- ---------- ---------- ---------- ---------- ---------- ---------- | 412 |
| PvCRN14 | ---------- ---------- ---------- ---------- ---------- ---------- ---------- ---------- | 151 |
| XP_024577521.1 | ---------- ---------- ---------- ---------- ---------- ---------- ---------- ---------- | 108 |
| PvCRN21 | ---------- ---------- ---------- ---------- ---------- ---------- ---------- ---------- | 479 |
| XP_024574966.1 | ---------- ---------- ---------- ---------- ---------- ---------- ---------- ---------- | 155 |
| XP_024575372.1 | ---------- ---------- ---------- ---------- ---------- ---------- ---------- ---------- | 188 |
| XP_024581363.1 | ---------- ---------- ---------- ---------- ---------- ---------- ---------- ---------- | 229 |
| XP_024583883.1 | ---------- ---------- ---------- ---------- ---------- ---------- ---------- ---------- | 381 |
| XP_024579844.1 | ---------- ---------- ---------- ---------- ---------- ---------- ---------- ---------- | 476 |
| XP_024574916.1 | ---------- ---------- ---------- ---------- ---------- ---------- ---------- ---------- | 621 |
| XP_024574193.1 | ---------- ---------- ---------- ---------- ---------- ---------- ---------- ---------- | 615 |
| XP_024583155.1 | ---------- ---------- ---------- ---------- ---------- ---------- ---------- ---------- | 419 |
| XP_024574913.1 | ---------- ---------- ---------- ---------- ---------- ---------- ---------- ---------- | 623 |
| XP_024578158.1 | ---------- ---------- ---------- ---------- ---------- ---------- ---------- ---------- | 106 |
| PvCRN22 | ---------- ---------- ---------- ---------- ---------- ---------- ---------- ---------- | 206 |
| XP_024585001.1 | ---------- ---------- ---------- ---------- ---------- ---------- ---------- ---------- | 127 |
| XP_024583843.1 | ---------- ---------- ---------- ---------- ---------- ---------- ---------- ---------- | 534 |
| XP_024578702.1 | ---------- ---------- ---------- ---------- ---------- ---------- ---------- ---------- | 299 |
| XP_024585805.1 | ---------- ---------- ---------- ---------- ---------- ---------- ---------- ---------- | 334 |
| XP_024580932.1 | ---------- ---------- ---------- ---------- ---------- ---------- ---------- ---------- | 508 |
| Consensus | ---------- ---------- ---------- ---------- ---------- ---------- ---------- ---------- | |
| Conservation (100% / 0%) | | |

```
                    9,060              9,080              9,100              9,120
PvCRN11         ---------- ---------- ---------- ---------- ---------- ---------- ---------- ---------- 215
XP_024579260.1  ---------- ---------- ---------- ---------- ---------- ---------- ---------- ---------- 163
XP_024573634.1  ---------- ---------- ---------- ---------- ---------- ---------- ---------- ---------- 169
PvCRN7          ---------- ---------- ---------- ---------- ---------- ---------- ---------- ---------- 753
XP_024585265.1  ---------- ---------- ---------- ---------- ---------- ---------- ---------- ---------- 128
PvCRN18         ---------- ---------- ---------- ---------- ---------- ---------- ---------- ---------- 302
XP_024578414.1  ---------- ---------- ---------- ---------- ---------- ---------- ---------- ---------- 624
XP_024579130.1  ---------- ---------- ---------- ---------- ---------- ---------- ---------- ---------- 345
XP_024584758.1  ---------- ---------- ---------- ---------- ---------- ---------- ---------- ---------- 214
XP_024586066.1  ---------- ---------- ---------- ---------- ---------- ---------- ---------- ---------- 263
XP_024585870.1  ---------- ---------- ---------- ---------- ---------- ---------- ---------- ---------- 280
PvCRN31         ---------- ---------- ---------- ---------- ---------- ---------- ---------- ---------- 583
XP_024577278.1  ---------- ---------- ---------- ---------- ---------- ---------- ---------- ---------- 333
XP_024585562.1  ---------- ---------- ---------- ---------- ---------- ---------- ---------- ---------- 158
XP_024573944.1  ---------- ---------- ---------- ---------- ---------- ---------- ---------- ---------- 592
XP_024579793.1  ---------- ---------- ---------- ---------- ---------- ---------- ---------- ---------- 163
PvCRN15         ---------- ---------- ---------- ---------- ---------- ---------- ---------- ---------- 290
PvCRN26         ---------- ---------- ---------- ---------- ---------- ---------- ---------- ---------- 279
PvCRN16         ---------- ---------- ---------- ---------- ---------- ---------- ---------- ---------- 365
PvCRN23         ---------- ---------- ---------- ---------- ---------- ---------- ---------- ---------- 426
XP_024573063.1  ---------- ---------- ---------- ---------- ---------- ---------- ---------- ---------- 83
PvCRN19         ---------- ---------- ---------- ---------- ---------- ---------- ---------- ---------- 164
XP_024581625.1  ---------- ---------- ---------- ---------- ---------- ---------- ---------- ---------- 135
XP_024583886.1  ---------- ---------- ---------- ---------- ---------- ---------- ---------- ---------- 96
XP_024584409.1  ---------- ---------- ---------- ---------- ---------- ---------- ---------- ---------- 213
XP_024572385.1  ---------- ---------- ---------- ---------- ---------- ---------- ---------- ---------- 310
PvCRN6          ---------- ---------- ---------- ---------- ---------- ---------- ---------- ---------- 372
PvCRN9          ---------- ---------- ---------- ---------- ---------- ---------- ---------- ---------- 611
XP_024580875.1  ---------- ---------- ---------- ---------- ---------- ---------- ---------- ---------- 127
PvCRN24         ---------- ---------- ---------- ---------- ---------- ---------- ---------- ---------- 165
PvCRN35         ---------- ---------- ---------- ---------- ---------- ---------- ---------- ---------- 212
PvCRN12         ---------- ---------- ---------- ---------- ---------- ---------- ---------- ---------- 171
XP_024576927.1  ---------- ---------- ---------- ---------- ---------- ---------- ---------- ---------- 555
XP_024578927.1  ---------- ---------- ---------- ---------- ---------- ---------- ---------- ---------- 327
XP_024586212.1  ---------- ---------- ---------- ---------- ---------- ---------- ---------- ---------- 616
XP_024578078.1  ---------- ---------- ---------- ---------- ---------- ---------- ---------- ---------- 262
XP_024575355.1  ---------- ---------- ---------- ---------- ---------- ---------- ---------- ---------- 295
XP_024584527.1  ---------- ---------- ---------- ---------- ---------- ---------- ---------- ---------- 168
XP_024586664.1  ---------- ---------- ---------- ---------- ---------- ---------- ---------- ---------- 357
PvCRN17         ---------- ---------- ---------- ---------- ---------- ---------- ---------- ---------- 187
PvCRN2          ---------- ---------- ---------- ---------- ---------- ---------- ---------- ---------- 179
XP_024577181.1  ---------- ---------- ---------- ---------- ---------- ---------- ---------- ---------- 207
XP_024581075.1  ---------- ---------- ---------- ---------- ---------- ---------- ---------- ---------- 156
XP_024575884.1  ---------- ---------- ---------- ---------- ---------- ---------- ---------- ---------- 155
XP_024586872.1  ---------- ---------- ---------- ---------- ---------- ---------- ---------- ---------- 182
XP_024573052.1  ---------- ---------- ---------- ---------- ---------- ---------- ---------- ---------- 187
XP_024586054.1  ---------- ---------- ---------- ---------- ---------- ---------- ---------- ---------- 237
PvCRN27         ---------- ---------- ---------- ---------- ---------- ---------- ---------- ---------- 313
PvCRN20         ---------- ---------- ---------- ---------- ---------- ---------- ---------- ---------- 159
PvCRN25         ---------- ---------- ---------- ---------- ---------- ---------- ---------- ---------- 194
PvCRN1          ---------- ---------- ---------- ---------- ---------- ---------- ---------- ---------- 580
PvCRN4          ---------- ---------- ---------- ---------- ---------- ---------- ---------- ---------- 583
PvCRN30         ---------- ---------- ---------- ---------- ---------- ---------- ---------- ---------- 220
PvCRN29         ---------- ---------- ---------- ---------- ---------- ---------- ---------- ---------- 312
XP_024583036.1  ---------- ---------- ---------- ---------- ---------- ---------- ---------- ---------- 681
PvCRN10         ---------- ---------- ---------- ---------- ---------- ---------- ---------- ---------- 217
XP_024577280.1  ---------- ---------- ---------- ---------- ---------- ---------- ---------- ---------- 283
XP_024572924.1  ---------- ---------- ---------- ---------- ---------- ---------- ---------- ---------- 412
PvCRN14         ---------- ---------- ---------- ---------- ---------- ---------- ---------- ---------- 151
XP_024577521.1  ---------- ---------- ---------- ---------- ---------- ---------- ---------- ---------- 108
PvCRN21         ---------- ---------- ---------- ---------- ---------- ---------- ---------- ---------- 479
XP_024574966.1  ---------- ---------- ---------- ---------- ---------- ---------- ---------- ---------- 155
XP_024575372.1  ---------- ---------- ---------- ---------- ---------- ---------- ---------- ---------- 188
XP_024581363.1  ---------- ---------- ---------- ---------- ---------- ---------- ---------- ---------- 229
XP_024583883.1  ---------- ---------- ---------- ---------- ---------- ---------- ---------- ---------- 381
XP_024579844.1  ---------- ---------- ---------- ---------- ---------- ---------- ---------- ---------- 476
XP_024574916.1  ---------- ---------- ---------- ---------- ---------- ---------- ---------- ---------- 621
XP_024574193.1  ---------- ---------- ---------- ---------- ---------- ---------- ---------- ---------- 615
XP_024583155.1  ---------- ---------- ---------- ---------- ---------- ---------- ---------- ---------- 419
XP_024574913.1  ---------- ---------- ---------- ---------- ---------- ---------- ---------- ---------- 623
XP_024578158.1  ---------- ---------- ---------- ---------- ---------- ---------- ---------- ---------- 106
PvCRN22         ---------- ---------- ---------- ---------- ---------- ---------- ---------- ---------- 206
XP_024585001.1  ---------- ---------- ---------- ---------- ---------- ---------- ---------- ---------- 127
XP_024583843.1  ---------- ---------- ---------- ---------- ---------- ---------- ---------- ---------- 534
XP_024578702.1  ---------- ---------- ---------- ---------- ---------- ---------- ---------- ---------- 299
XP_024585805.1  ---------- ---------- ---------- ---------- ---------- ---------- ---------- ---------- 334
XP_024580932.1  ---------- ---------- ---------- ---------- ---------- ---------- ---------- ---------- 508
Consensus       ---------- ---------- ---------- ---------- ---------- ---------- ---------- ----------
100%
Conservation
0%
```

| | 9,140 · 9,160 · 9,180 · 9,200 | |
|---|---|---|
| PvCRN11 | ---------- ---------- ---------- ---------- ---------- ---------- ---------- ---------- | 215 |
| XP_024579260.1 | ---------- ---------- ---------- ---------- ---------- ---------- ---------- ---------- | 163 |
| XP_024573634.1 | ---------- ---------- ---------- ---------- ---------- ---------- ---------- ---------- | 169 |
| PvCRN7 | ---------- ---------- ---------- ---------- ---------- ---------- ---------- ---------- | 753 |
| XP_024585265.1 | ---------- ---------- ---------- ---------- ---------- ---------- ---------- ---------- | 128 |
| PvCRN18 | ---------- ---------- ---------- ---------- ---------- ---------- ---------- ---------- | 302 |
| XP_024578414.1 | ---------- ---------- ---------- ---------- ---------- ---------- ---------- ---------- | 624 |
| XP_024579130.1 | ---------- ---------- ---------- ---------- ---------- ---------- ---------- ---------- | 345 |
| XP_024584758.1 | ---------- ---------- ---------- ---------- ---------- ---------- ---------- ---------- | 214 |
| XP_024586066.1 | ---------- ---------- ---------- ---------- ---------- ---------- ---------- ---------- | 263 |
| XP_024585870.1 | ---------- ---------- ---------- ---------- ---------- ---------- ---------- ---------- | 280 |
| PvCRN31 | ---------- ---------- ---------- ---------- ---------- ---------- ---------- ---------- | 583 |
| XP_024577278.1 | ---------- ---------- ---------- ---------- ---------- ---------- ---------- ---------- | 333 |
| XP_024585562.1 | ---------- ---------- ---------- ---------- ---------- ---------- ---------- ---------- | 158 |
| XP_024573944.1 | ---------- ---------- ---------- ---------- ---------- ---------- ---------- ---------- | 592 |
| XP_024579793.1 | ---------- ---------- ---------- ---------- ---------- ---------- ---------- ---------- | 163 |
| PvCRN15 | ---------- ---------- ---------- ---------- ---------- ---------- ---------- ---------- | 290 |
| PvCRN26 | ---------- ---------- ---------- ---------- ---------- ---------- ---------- ---------- | 279 |
| PvCRN16 | ---------- ---------- ---------- ---------- ---------- ---------- ---------- ---------- | 365 |
| PvCRN23 | ---------- ---------- ---------- ---------- ---------- ---------- ---------- ---------- | 426 |
| XP_024573063.1 | ---------- ---------- ---------- ---------- ---------- ---------- ---------- ---------- | 83 |
| PvCRN19 | ---------- ---------- ---------- ---------- ---------- ---------- ---------- ---------- | 164 |
| XP_024581625.1 | ---------- ---------- ---------- ---------- ---------- ---------- ---------- ---------- | 135 |
| XP_024583886.1 | ---------- ---------- ---------- ---------- ---------- ---------- ---------- ---------- | 96 |
| XP_024584409.1 | ---------- ---------- ---------- ---------- ---------- ---------- ---------- ---------- | 213 |
| XP_024572385.1 | ---------- ---------- ---------- ---------- ---------- ---------- ---------- ---------- | 310 |
| PvCRN6 | ---------- ---------- ---------- ---------- ---------- ---------- ---------- ---------- | 372 |
| PvCRN9 | ---------- ---------- ---------- ---------- ---------- ---------- ---------- ---------- | 611 |
| XP_024580875.1 | ---------- ---------- ---------- ---------- ---------- ---------- ---------- ---------- | 127 |
| PvCRN24 | ---------- ---------- ---------- ---------- ---------- ---------- ---------- ---------- | 165 |
| PvCRN35 | ---------- ---------- ---------- ---------- ---------- ---------- ---------- ---------- | 212 |
| PvCRN12 | ---------- ---------- ---------- ---------- ---------- ---------- ---------- ---------- | 171 |
| XP_024576927.1 | ---------- ---------- ---------- ---------- ---------- ---------- ---------- ---------- | 555 |
| XP_024578927.1 | ---------- ---------- ---------- ---------- ---------- ---------- ---------- ---------- | 327 |
| XP_024586212.1 | ---------- ---------- ---------- ---------- ---------- ---------- ---------- ---------- | 616 |
| XP_024578078.1 | ---------- ---------- ---------- ---------- ---------- ---------- ---------- ---------- | 262 |
| XP_024575355.1 | ---------- ---------- ---------- ---------- ---------- ---------- ---------- ---------- | 295 |
| XP_024584527.1 | ---------- ---------- ---------- ---------- ---------- ---------- ---------- ---------- | 168 |
| XP_024586664.1 | ---------- ---------- ---------- ---------- ---------- ---------- ---------- ---------- | 357 |
| PvCRN17 | ---------- ---------- ---------- ---------- ---------- ---------- ---------- ---------- | 187 |
| PvCRN2 | ---------- ---------- ---------- ---------- ---------- ---------- ---------- ---------- | 179 |
| XP_024577181.1 | ---------- ---------- ---------- ---------- ---------- ---------- ---------- ---------- | 207 |
| XP_024581075.1 | ---------- ---------- ---------- ---------- ---------- ---------- ---------- ---------- | 156 |
| XP_024575884.1 | ---------- ---------- ---------- ---------- ---------- ---------- ---------- ---------- | 155 |
| XP_024586872.1 | ---------- ---------- ---------- ---------- ---------- ---------- ---------- ---------- | 182 |
| XP_024573052.1 | ---------- ---------- ---------- ---------- ---------- ---------- ---------- ---------- | 187 |
| XP_024586054.1 | ---------- ---------- ---------- ---------- ---------- ---------- ---------- ---------- | 237 |
| PvCRN27 | ---------- ---------- ---------- ---------- ---------- ---------- ---------- ---------- | 313 |
| PvCRN20 | ---------- ---------- ---------- ---------- ---------- ---------- ---------- ---------- | 159 |
| PvCRN25 | ---------- ---------- ---------- ---------- ---------- ---------- ---------- ---------- | 194 |
| PvCRN1 | ---------- ---------- ---------- ---------- ---------- ---------- ---------- ---------- | 580 |
| PvCRN4 | ---------- ---------- ---------- ---------- ---------- ---------- ---------- ---------- | 583 |
| PvCRN30 | ---------- ---------- ---------- ---------- ---------- ---------- ---------- ---------- | 220 |
| PvCRN29 | ---------- ---------- ---------- ---------- ---------- ---------- ---------- ---------- | 312 |
| XP_024583036.1 | ---------- ---------- ---------- ---------- ---------- ---------- ---------- ---------- | 681 |
| PvCRN10 | ---------- ---------- ---------- ---------- ---------- ---------- ---------- ---------- | 217 |
| XP_024577280.1 | ---------- ---------- ---------- ---------- ---------- ---------- ---------- ---------- | 283 |
| XP_024572924.1 | ---------- ---------- ---------- ---------- ---------- ---------- ---------- ---------- | 412 |
| PvCRN14 | ---------- ---------- ---------- ---------- ---------- ---------- ---------- ---------- | 151 |
| XP_024577521.1 | ---------- ---------- ---------- ---------- ---------- ---------- ---------- ---------- | 108 |
| PvCRN21 | ---------- ---------- ---------- ---------- ---------- ---------- ---------- ---------- | 479 |
| XP_024574966.1 | ---------- ---------- ---------- ---------- ---------- ---------- ---------- ---------- | 155 |
| XP_024575372.1 | ---------- ---------- ---------- ---------- ---------- ---------- ---------- ---------- | 188 |
| XP_024581363.1 | ---------- ---------- ---------- ---------- ---------- ---------- ---------- ---------- | 229 |
| XP_024583883.1 | ---------- ---------- ---------- ---------- ---------- ---------- ---------- ---------- | 381 |
| XP_024579844.1 | ---------- ---------- ---------- ---------- ---------- ---------- ---------- ---------- | 476 |
| XP_024574916.1 | ---------- ---------- ---------- ---------- ---------- ---------- ---------- ---------- | 621 |
| XP_024574193.1 | ---------- ---------- ---------- ---------- ---------- ---------- ---------- ---------- | 615 |
| XP_024583155.1 | ---------- ---------- ---------- ---------- ---------- ---------- ---------- ---------- | 419 |
| XP_024574913.1 | ---------- ---------- ---------- ---------- ---------- ---------- ---------- ---------- | 623 |
| XP_024578158.1 | ---------- ---------- ---------- ---------- ---------- ---------- ---------- ---------- | 106 |
| PvCRN22 | ---------- ---------- ---------- ---------- ---------- ---------- ---------- ---------- | 206 |
| XP_024585001.1 | ---------- ---------- ---------- ---------- ---------- ---------- ---------- ---------- | 127 |
| XP_024583843.1 | ---------- ---------- ---------- ---------- ---------- ---------- ---------- ---------- | 534 |
| XP_024578702.1 | ---------- ---------- ---------- ---------- ---------- ---------- ---------- ---------- | 299 |
| XP_024585805.1 | ---------- ---------- ---------- ---------- ---------- ---------- ---------- ---------- | 334 |
| XP_024580932.1 | ---------- ---------- ---------- ---------- ---------- ---------- ---------- ---------- | 508 |
| Consensus | ---------- ---------- ---------- ---------- ---------- ---------- ---------- ---------- | |
| Conservation (100% – 0%) | | |

| | 9,220 9,240 9,260 9,280 | |
|---|---|---|
| PvCRN11 | ---------- ---------- ---------- ---------- ---------- ---------- ---------- ---------- | 215 |
| XP_024579260.1 | ---------- ---------- ---------- ---------- ---------- ---------- ---------- ---------- | 163 |
| XP_024573634.1 | ---------- ---------- ---------- ---------- ---------- ---------- ---------- ---------- | 169 |
| PvCRN7 | ---------- ---------- ---------- ---------- ---------- ---------- ---------- ---------- | 753 |
| XP_024585265.1 | ---------- ---------- ---------- ---------- ---------- ---------- ---------- ---------- | 128 |
| PvCRN18 | ---------- ---------- ---------- ---------- ---------- ---------- ---------- ---------- | 302 |
| XP_024578414.1 | ---------- ---------- ---------- ---------- ---------- ---------- ---------- ---------- | 624 |
| XP_024579130.1 | ---------- ---------- ---------- ---------- ---------- ---------- ---------- ---------- | 345 |
| XP_024584758.1 | ---------- ---------- ---------- ---------- ---------- ---------- ---------- ---------- | 214 |
| XP_024586066.1 | ---------- ---------- ---------- ---------- ---------- ---------- ---------- ---------- | 263 |
| XP_024585870.1 | ---------- ---------- ---------- ---------- ---------- ---------- ---------- ---------- | 280 |
| PvCRN31 | ---------- ---------- ---------- ---------- ---------- ---------- ---------- ---------- | 583 |
| XP_024577278.1 | ---------- ---------- ---------- ---------- ---------- ---------- ---------- ---------- | 333 |
| XP_024585562.1 | ---------- ---------- ---------- ---------- ---------- ---------- ---------- ---------- | 158 |
| XP_024573944.1 | ---------- ---------- ---------- ---------- ---------- ---------- ---------- ---------- | 592 |
| XP_024579793.1 | ---------- ---------- ---------- ---------- ---------- ---------- ---------- ---------- | 163 |
| PvCRN15 | ---------- ---------- ---------- ---------- ---------- ---------- ---------- ---------- | 290 |
| PvCRN26 | ---------- ---------- ---------- ---------- ---------- ---------- ---------- ---------- | 279 |
| PvCRN16 | ---------- ---------- ---------- ---------- ---------- ---------- ---------- ---------- | 365 |
| PvCRN23 | ---------- ---------- ---------- ---------- ---------- ---------- ---------- ---------- | 426 |
| XP_024573063.1 | ---------- ---------- ---------- ---------- ---------- ---------- ---------- ---------- | 83 |
| PvCRN19 | ---------- ---------- ---------- ---------- ---------- ---------- ---------- ---------- | 164 |
| XP_024581625.1 | ---------- ---------- ---------- ---------- ---------- ---------- ---------- ---------- | 135 |
| XP_024583886.1 | ---------- ---------- ---------- ---------- ---------- ---------- ---------- ---------- | 96 |
| XP_024584409.1 | ---------- ---------- ---------- ---------- ---------- ---------- ---------- ---------- | 213 |
| XP_024572385.1 | ---------- ---------- ---------- ---------- ---------- ---------- ---------- ---------- | 310 |
| PvCRN6 | ---------- ---------- ---------- ---------- ---------- ---------- ---------- ---------- | 372 |
| PvCRN9 | ---------- ---------- ---------- ---------- ---------- ---------- ---------- ---------- | 611 |
| XP_024580875.1 | ---------- ---------- ---------- ---------- ---------- ---------- ---------- ---------- | 127 |
| PvCRN24 | ---------- ---------- ---------- ---------- ---------- ---------- ---------- ---------- | 165 |
| PvCRN35 | ---------- ---------- ---------- ---------- ---------- ---------- ---------- ---------- | 212 |
| PvCRN12 | ---------- ---------- ---------- ---------- ---------- ---------- ---------- ---------- | 171 |
| XP_024576927.1 | ---------- ---------- ---------- ---------- ---------- ---------- ---------- ---------- | 555 |
| XP_024578927.1 | ---------- ---------- ---------- ---------- ---------- ---------- ---------- ---------- | 327 |
| XP_024586212.1 | ---------- ---------- ---------- ---------- ---------- ---------- ---------- ---------- | 616 |
| XP_024578078.1 | ---------- ---------- ---------- ---------- ---------- ---------- ---------- ---------- | 262 |
| XP_024575355.1 | ---------- ---------- ---------- ---------- ---------- ---------- ---------- ---------- | 295 |
| XP_024584527.1 | ---------- ---------- ---------- ---------- ---------- ---------- ---------- ---------- | 168 |
| XP_024586664.1 | ---------- ---------- ---------- ---------- ---------- ---------- ---------- ---------- | 357 |
| PvCRN17 | ---------- ---------- ---------- ---------- ---------- ---------- ---------- ---------- | 187 |
| PvCRN2 | ---------- ---------- ---------- ---------- ---------- ---------- ---------- ---------- | 179 |
| XP_024577181.1 | ---------- ---------- ---------- ---------- ---------- ---------- ---------- ---------- | 207 |
| XP_024581075.1 | ---------- ---------- ---------- ---------- ---------- ---------- ---------- ---------- | 156 |
| XP_024575884.1 | ---------- ---------- ---------- ---------- ---------- ---------- ---------- ---------- | 155 |
| XP_024586872.1 | ---------- ---------- ---------- ---------- ---------- ---------- ---------- ---------- | 182 |
| XP_024573052.1 | ---------- ---------- ---------- ---------- ---------- ---------- ---------- ---------- | 187 |
| XP_024586054.1 | ---------- ---------- ---------- ---------- ---------- ---------- ---------- ---------- | 237 |
| PvCRN27 | ---------- ---------- ---------- ---------- ---------- ---------- ---------- ---------- | 313 |
| PvCRN20 | ---------- ---------- ---------- ---------- ---------- ---------- ---------- ---------- | 159 |
| PvCRN25 | ---------- ---------- ---------- ---------- ---------- ---------- ---------- ---------- | 194 |
| PvCRN1 | ---------- ---------- ---------- ---------- ---------- ---------- ---------- ---------- | 580 |
| PvCRN4 | ---------- ---------- ---------- ---------- ---------- ---------- ---------- ---------- | 583 |
| PvCRN30 | ---------- ---------- ---------- ---------- ---------- ---------- ---------- ---------- | 220 |
| PvCRN29 | ---------- ---------- ---------- ---------- ---------- ---------- ---------- ---------- | 312 |
| XP_024583036.1 | ---------- ---------- ---------- ---------- ---------- ---------- ---------- ---------- | 681 |
| PvCRN10 | ---------- ---------- ---------- ---------- ---------- ---------- ---------- ---------- | 217 |
| XP_024577280.1 | ---------- ---------- ---------- ---------- ---------- ---------- ---------- ---------- | 283 |
| XP_024572924.1 | ---------- ---------- ---------- ---------- ---------- ---------- ---------- ---------- | 412 |
| PvCRN14 | ---------- ---------- ---------- ---------- ---------- ---------- ---------- ---------- | 151 |
| XP_024577521.1 | ---------- ---------- ---------- ---------- ---------- ---------- ---------- ---------- | 108 |
| PvCRN21 | ---------- ---------- ---------- ---------- ---------- ---------- ---------- ---------- | 479 |
| XP_024574966.1 | ---------- ---------- ---------- ---------- ---------- ---------- ---------- ---------- | 155 |
| XP_024575372.1 | ---------- ---------- ---------- ---------- ---------- ---------- ---------- ---------- | 188 |
| XP_024581363.1 | ---------- ---------- ---------- ---------- ---------- ---------- ---------- ---------- | 229 |
| XP_024583883.1 | ---------- ---------- ---------- ---------- ---------- ---------- ---------- ---------- | 381 |
| XP_024579844.1 | ---------- ---------- ---------- ---------- ---------- ---------- ---------- ---------- | 476 |
| XP_024574916.1 | ---------- ---------- ---------- ---------- ---------- ---------- ---------- ---------- | 621 |
| XP_024574193.1 | ---------- ---------- ---------- ---------- ---------- ---------- ---------- ---------- | 615 |
| XP_024583155.1 | ---------- ---------- ---------- ---------- ---------- ---------- ---------- ---------- | 419 |
| XP_024574913.1 | ---------- ---------- ---------- ---------- ---------- ---------- ---------- ---------- | 623 |
| XP_024578158.1 | ---------- ---------- ---------- ---------- ---------- ---------- ---------- ---------- | 106 |
| PvCRN22 | ---------- ---------- ---------- ---------- ---------- ---------- ---------- ---------- | 206 |
| XP_024585001.1 | ---------- ---------- ---------- ---------- ---------- ---------- ---------- ---------- | 127 |
| XP_024583843.1 | ---------- ---------- ---------- ---------- ---------- ---------- ---------- ---------- | 534 |
| XP_024578702.1 | ---------- ---------- ---------- ---------- ---------- ---------- ---------- ---------- | 299 |
| XP_024585805.1 | ---------- ---------- ---------- ---------- ---------- ---------- ---------- ---------- | 334 |
| XP_024580932.1 | ---------- ---------- ---------- ---------- ---------- ---------- ---------- ---------- | 508 |
| Consensus | ---------- ---------- ---------- ---------- ---------- ---------- ---------- ---------- | |
| Conservation (100% – 0%) | | |

| | 9,300 · 9,320 · 9,340 · 9,360 | |
|---|---|---|
| PvCRN11 | ---------- ---------- ---------- ---------- ---------- ---------- ---------- ---------- | 215 |
| XP_024579260.1 | ---------- ---------- ---------- ---------- ---------- ---------- ---------- ---------- | 163 |
| XP_024573634.1 | ---------- ---------- ---------- ---------- ---------- ---------- ---------- ---------- | 169 |
| PvCRN7 | ---------- ---------- ---------- ---------- ---------- ---------- ---------- ---------- | 753 |
| XP_024585265.1 | ---------- ---------- ---------- ---------- ---------- ---------- ---------- ---------- | 128 |
| PvCRN18 | ---------- ---------- ---------- ---------- ---------- ---------- ---------- ---------- | 302 |
| XP_024578414.1 | ---------- ---------- ---------- ---------- ---------- ---------- ---------- ---------- | 624 |
| XP_024579130.1 | ---------- ---------- ---------- ---------- ---------- ---------- ---------- ---------- | 345 |
| XP_024584758.1 | ---------- ---------- ---------- ---------- ---------- ---------- ---------- ---------- | 214 |
| XP_024586066.1 | ---------- ---------- ---------- ---------- ---------- ---------- ---------- ---------- | 263 |
| XP_024585870.1 | ---------- ---------- ---------- ---------- ---------- ---------- ---------- ---------- | 280 |
| PvCRN31 | ---------- ---------- ---------- ---------- ---------- ---------- ---------- ---------- | 583 |
| XP_024577278.1 | ---------- ---------- ---------- ---------- ---------- ---------- ---------- ---------- | 333 |
| XP_024585562.1 | ---------- ---------- ---------- ---------- ---------- ---------- ---------- ---------- | 158 |
| XP_024573944.1 | ---------- ---------- ---------- ---------- ---------- ---------- ---------- ---------- | 592 |
| XP_024579793.1 | ---------- ---------- ---------- ---------- ---------- ---------- ---------- ---------- | 163 |
| PvCRN15 | ---------- ---------- ---------- ---------- ---------- ---------- ---------- ---------- | 290 |
| PvCRN26 | ---------- ---------- ---------- ---------- ---------- ---------- ---------- ---------- | 279 |
| PvCRN16 | ---------- ---------- ---------- ---------- ---------- ---------- ---------- ---------- | 365 |
| PvCRN23 | ---------- ---------- ---------- ---------- ---------- ---------- ---------- ---------- | 426 |
| XP_024573063.1 | ---------- ---------- ---------- ---------- ---------- ---------- ---------- ---------- | 83 |
| PvCRN19 | ---------- ---------- ---------- ---------- ---------- ---------- ---------- ---------- | 164 |
| XP_024581625.1 | ---------- ---------- ---------- ---------- ---------- ---------- ---------- ---------- | 135 |
| XP_024583886.1 | ---------- ---------- ---------- ---------- ---------- ---------- ---------- ---------- | 96 |
| XP_024584409.1 | ---------- ---------- ---------- ---------- ---------- ---------- ---------- ---------- | 213 |
| XP_024572385.1 | ---------- ---------- ---------- ---------- ---------- ---------- ---------- ---------- | 310 |
| PvCRN6 | ---------- ---------- ---------- ---------- ---------- ---------- ---------- ---------- | 372 |
| PvCRN9 | ---------- ---------- ---------- ---------- ---------- ---------- ---------- ---------- | 611 |
| XP_024580875.1 | ---------- ---------- ---------- ---------- ---------- ---------- ---------- ---------- | 127 |
| PvCRN24 | ---------- ---------- ---------- ---------- ---------- ---------- ---------- ---------- | 165 |
| PvCRN35 | ---------- ---------- ---------- ---------- ---------- ---------- ---------- ---------- | 212 |
| PvCRN12 | ---------- ---------- ---------- ---------- ---------- ---------- ---------- ---------- | 171 |
| XP_024576927.1 | ---------- ---------- ---------- ---------- ---------- ---------- ---------- ---------- | 555 |
| XP_024578927.1 | ---------- ---------- ---------- ---------- ---------- ---------- ---------- ---------- | 327 |
| XP_024586212.1 | ---------- ---------- ---------- ---------- ---------- ---------- ---------- ---------- | 616 |
| XP_024578078.1 | ---------- ---------- ---------- ---------- ---------- ---------- ---------- ---------- | 262 |
| XP_024575355.1 | ---------- ---------- ---------- ---------- ---------- ---------- ---------- ---------- | 295 |
| XP_024584527.1 | ---------- ---------- ---------- ---------- ---------- ---------- ---------- ---------- | 168 |
| XP_024586664.1 | ---------- ---------- ---------- ---------- ---------- ---------- ---------- ---------- | 357 |
| PvCRN17 | ---------- ---------- ---------- ---------- ---------- ---------- ---------- ---------- | 187 |
| PvCRN2 | ---------- ---------- ---------- ---------- ---------- ---------- ---------- ---------- | 179 |
| XP_024577181.1 | ---------- ---------- ---------- ---------- ---------- ---------- ---------- ---------- | 207 |
| XP_024581075.1 | ---------- ---------- ---------- ---------- ---------- ---------- ---------- ---------- | 156 |
| XP_024575884.1 | ---------- ---------- ---------- ---------- ---------- ---------- ---------- ---------- | 155 |
| XP_024586872.1 | ---------- ---------- ---------- ---------- ---------- ---------- ---------- ---------- | 182 |
| XP_024573052.1 | ---------- ---------- ---------- ---------- ---------- ---------- ---------- ---------- | 187 |
| XP_024586054.1 | ---------- ---------- ---------- ---------- ---------- ---------- ---------- ---------- | 237 |
| PvCRN27 | ---------- ---------- ---------- ---------- ---------- ---------- ---------- ---------- | 313 |
| PvCRN20 | ---------- ---------- ---------- ---------- ---------- ---------- ---------- ---------- | 159 |
| PvCRN25 | ---------- ---------- ---------- ---------- ---------- ---------- ---------- ---------- | 194 |
| PvCRN1 | ---------- ---------- ---------- ---------- ---------- ---------- ---------- ---------- | 580 |
| PvCRN4 | ---------- ---------- ---------- ---------- ---------- ---------- ---------- ---------- | 583 |
| PvCRN30 | ---------- ---------- ---------- ---------- ---------- ---------- ---------- ---------- | 220 |
| PvCRN29 | ---------- ---------- ---------- ---------- ---------- ---------- ---------- ---------- | 312 |
| XP_024583036.1 | ---------- ---------- ---------- ---------- ---------- ---------- ---------- ---------- | 681 |
| PvCRN10 | ---------- ---------- ---------- ---------- ---------- ---------- ---------- ---------- | 217 |
| XP_024577280.1 | ---------- ---------- ---------- ---------- ---------- ---------- ---------- ---------- | 283 |
| XP_024572924.1 | ---------- ---------- ---------- ---------- ---------- ---------- ---------- ---------- | 412 |
| PvCRN14 | ---------- ---------- ---------- ---------- ---------- ---------- ---------- ---------- | 151 |
| XP_024577521.1 | ---------- ---------- ---------- ---------- ---------- ---------- ---------- ---------- | 108 |
| PvCRN21 | ---------- ---------- ---------- ---------- ---------- ---------- ---------- ---------- | 479 |
| XP_024574966.1 | ---------- ---------- ---------- ---------- ---------- ---------- ---------- ---------- | 155 |
| XP_024575372.1 | ---------- ---------- ---------- ---------- ---------- ---------- ---------- ---------- | 188 |
| XP_024581363.1 | ---------- ---------- ---------- ---------- ---------- ---------- ---------- ---------- | 229 |
| XP_024583883.1 | ---------- ---------- ---------- ---------- ---------- ---------- ---------- ---------- | 381 |
| XP_024579844.1 | ---------- ---------- ---------- ---------- ---------- ---------- ---------- ---------- | 476 |
| XP_024574916.1 | ---------- ---------- ---------- ---------- ---------- ---------- ---------- ---------- | 621 |
| XP_024574193.1 | ---------- ---------- ---------- ---------- ---------- ---------- ---------- ---------- | 615 |
| XP_024583155.1 | ---------- ---------- ---------- ---------- ---------- ---------- ---------- ---------- | 419 |
| XP_024574913.1 | ---------- ---------- ---------- ---------- ---------- ---------- ---------- ---------- | 623 |
| XP_024578158.1 | ---------- ---------- ---------- ---------- ---------- ---------- ---------- ---------- | 106 |
| PvCRN22 | ---------- ---------- ---------- ---------- ---------- ---------- ---------- ---------- | 206 |
| XP_024585001.1 | ---------- ---------- ---------- ---------- ---------- ---------- ---------- ---------- | 127 |
| XP_024583843.1 | ---------- ---------- ---------- ---------- ---------- ---------- ---------- ---------- | 534 |
| XP_024578702.1 | ---------- ---------- ---------- ---------- ---------- ---------- ---------- ---------- | 299 |
| XP_024585805.1 | ---------- ---------- ---------- ---------- ---------- ---------- ---------- ---------- | 334 |
| XP_024580932.1 | ---------- ---------- ---------- ---------- ---------- ---------- ---------- ---------- | 508 |
| Consensus | ---------- ---------- ---------- ---------- ---------- ---------- ---------- ---------- | |
| Conservation (100% / 0%) | | |

| | 9,380 9,400 9,420 9,440 | |
|---|---|---|
| PvCRN11 | ---------- ---------- ---------- ---------- ---------- ---------- ---------- ---------- | 215 |
| XP_024579260.1 | ---------- ---------- ---------- ---------- ---------- ---------- ---------- ---------- | 163 |
| XP_024573634.1 | ---------- ---------- ---------- ---------- ---------- ---------- ---------- ---------- | 169 |
| PvCRN7 | ---------- ---------- ---------- ---------- ---------- ---------- ---------- ---------- | 753 |
| XP_024585265.1 | ---------- ---------- ---------- ---------- ---------- ---------- ---------- ---------- | 128 |
| PvCRN18 | ---------- ---------- ---------- ---------- ---------- ---------- ---------- ---------- | 302 |
| XP_024578414.1 | ---------- ---------- ---------- ---------- ---------- ---------- ---------- ---------- | 624 |
| XP_024579130.1 | ---------- ---------- ---------- ---------- ---------- ---------- ---------- ---------- | 345 |
| XP_024584758.1 | ---------- ---------- ---------- ---------- ---------- ---------- ---------- ---------- | 214 |
| XP_024586066.1 | ---------- ---------- ---------- ---------- ---------- ---------- ---------- ---------- | 263 |
| XP_024585870.1 | ---------- ---------- ---------- ---------- ---------- ---------- ---------- ---------- | 280 |
| PvCRN31 | ---------- ---------- ---------- ---------- ---------- ---------- ---------- ---------- | 583 |
| XP_024577278.1 | ---------- ---------- ---------- ---------- ---------- ---------- ---------- ---------- | 333 |
| XP_024585562.1 | ---------- ---------- ---------- ---------- ---------- ---------- ---------- ---------- | 158 |
| XP_024573944.1 | ---------- ---------- ---------- ---------- ---------- ---------- ---------- ---------- | 592 |
| XP_024579793.1 | ---------- ---------- ---------- ---------- ---------- ---------- ---------- ---------- | 163 |
| PvCRN15 | ---------- ---------- ---------- ---------- ---------- ---------- ---------- ---------- | 290 |
| PvCRN26 | ---------- ---------- ---------- ---------- ---------- ---------- ---------- ---------- | 279 |
| PvCRN16 | ---------- ---------- ---------- ---------- ---------- ---------- ---------- ---------- | 365 |
| PvCRN23 | ---------- ---------- ---------- ---------- ---------- ---------- ---------- ---------- | 426 |
| XP_024573063.1 | ---------- ---------- ---------- ---------- ---------- ---------- ---------- ---------- | 83 |
| PvCRN19 | ---------- ---------- ---------- ---------- ---------- ---------- ---------- ---------- | 164 |
| XP_024581625.1 | ---------- ---------- ---------- ---------- ---------- ---------- ---------- ---------- | 135 |
| XP_024583886.1 | ---------- ---------- ---------- ---------- ---------- ---------- ---------- ---------- | 96 |
| XP_024584409.1 | ---------- ---------- ---------- ---------- ---------- ---------- ---------- ---------- | 213 |
| XP_024572385.1 | ---------- ---------- ---------- ---------- ---------- ---------- ---------- ---------- | 310 |
| PvCRN6 | ---------- ---------- ---------- ---------- ---------- ---------- ---------- ---------- | 372 |
| PvCRN9 | ---------- ---------- ---------- ---------- ---------- ---------- ---------- ---------- | 611 |
| XP_024580875.1 | ---------- ---------- ---------- ---------- ---------- ---------- ---------- ---------- | 127 |
| PvCRN24 | ---------- ---------- ---------- ---------- ---------- ---------- ---------- ---------- | 165 |
| PvCRN35 | ---------- ---------- ---------- ---------- ---------- ---------- ---------- ---------- | 212 |
| PvCRN12 | ---------- ---------- ---------- ---------- ---------- ---------- ---------- ---------- | 171 |
| XP_024576927.1 | ---------- ---------- ---------- ---------- ---------- ---------- ---------- ---------- | 555 |
| XP_024578927.1 | ---------- ---------- ---------- ---------- ---------- ---------- ---------- ---------- | 327 |
| XP_024586212.1 | ---------- ---------- ---------- ---------- ---------- ---------- ---------- ---------- | 616 |
| XP_024578078.1 | ---------- ---------- ---------- ---------- ---------- ---------- ---------- ---------- | 262 |
| XP_024575355.1 | ---------- ---------- ---------- ---------- ---------- ---------- ---------- ---------- | 295 |
| XP_024584527.1 | ---------- ---------- ---------- ---------- ---------- ---------- ---------- ---------- | 168 |
| XP_024586664.1 | ---------- ---------- ---------- ---------- ---------- ---------- ---------- ---------- | 357 |
| PvCRN17 | ---------- ---------- ---------- ---------- ---------- ---------- ---------- ---------- | 187 |
| PvCRN2 | ---------- ---------- ---------- ---------- ---------- ---------- ---------- ---------- | 179 |
| XP_024577181.1 | ---------- ---------- ---------- ---------- ---------- ---------- ---------- ---------- | 207 |
| XP_024581075.1 | ---------- ---------- ---------- ---------- ---------- ---------- ---------- ---------- | 156 |
| XP_024575884.1 | ---------- ---------- ---------- ---------- ---------- ---------- ---------- ---------- | 155 |
| XP_024586872.1 | ---------- ---------- ---------- ---------- ---------- ---------- ---------- ---------- | 182 |
| XP_024573052.1 | ---------- ---------- ---------- ---------- ---------- ---------- ---------- ---------- | 187 |
| XP_024586054.1 | ---------- ---------- ---------- ---------- ---------- ---------- ---------- ---------- | 237 |
| PvCRN27 | ---------- ---------- ---------- ---------- ---------- ---------- ---------- ---------- | 313 |
| PvCRN20 | ---------- ---------- ---------- ---------- ---------- ---------- ---------- ---------- | 159 |
| PvCRN25 | ---------- ---------- ---------- ---------- ---------- ---------- ---------- ---------- | 194 |
| PvCRN1 | ---------- ---------- ---------- ---------- ---------- ---------- ---------- ---------- | 580 |
| PvCRN4 | ---------- ---------- ---------- ---------- ---------- ---------- ---------- ---------- | 583 |
| PvCRN30 | ---------- ---------- ---------- ---------- ---------- ---------- ---------- ---------- | 220 |
| PvCRN29 | ---------- ---------- ---------- ---------- ---------- ---------- ---------- ---------- | 312 |
| XP_024583036.1 | ---------- ---------- ---------- ---------- ---------- ---------- ---------- ---------- | 681 |
| PvCRN10 | ---------- ---------- ---------- ---------- ---------- ---------- ---------- ---------- | 217 |
| XP_024577280.1 | ---------- ---------- ---------- ---------- ---------- ---------- ---------- ---------- | 283 |
| XP_024572924.1 | ---------- ---------- ---------- ---------- ---------- ---------- ---------- ---------- | 412 |
| PvCRN14 | ---------- ---------- ---------- ---------- ---------- ---------- ---------- ---------- | 151 |
| XP_024577521.1 | ---------- ---------- ---------- ---------- ---------- ---------- ---------- ---------- | 108 |
| PvCRN21 | ---------- ---------- ---------- ---------- ---------- ---------- ---------- ---------- | 479 |
| XP_024574966.1 | ---------- ---------- ---------- ---------- ---------- ---------- ---------- ---------- | 155 |
| XP_024575372.1 | ---------- ---------- ---------- ---------- ---------- ---------- ---------- ---------- | 188 |
| XP_024581363.1 | ---------- ---------- ---------- ---------- ---------- ---------- ---------- ---------- | 229 |
| XP_024583883.1 | ---------- ---------- ---------- ---------- ---------- ---------- ---------- ---------- | 381 |
| XP_024579844.1 | ---------- ---------- ---------- ---------- ---------- ---------- ---------- ---------- | 476 |
| XP_024574916.1 | ---------- ---------- ---------- ---------- ---------- ---------- ---------- ---------- | 621 |
| XP_024574193.1 | ---------- ---------- ---------- ---------- ---------- ---------- ---------- ---------- | 615 |
| XP_024583155.1 | ---------- ---------- ---------- ---------- ---------- ---------- ---------- ---------- | 419 |
| XP_024574913.1 | ---------- ---------- ---------- ---------- ---------- ---------- ---------- ---------- | 623 |
| XP_024578158.1 | ---------- ---------- ---------- ---------- ---------- ---------- ---------- ---------- | 106 |
| PvCRN22 | ---------- ---------- ---------- ---------- ---------- ---------- ---------- ---------- | 206 |
| XP_024585001.1 | ---------- ---------- ---------- ---------- ---------- ---------- ---------- ---------- | 127 |
| XP_024583843.1 | ---------- ---------- ---------- ---------- ---------- ---------- ---------- ---------- | 534 |
| XP_024578702.1 | ---------- ---------- ---------- ---------- ---------- ---------- ---------- ---------- | 299 |
| XP_024585805.1 | ---------- ---------- ---------- ---------- ---------- ---------- ---------- ---------- | 334 |
| XP_024580932.1 | ---------- ---------- ---------- ---------- ---------- ---------- ---------- ---------- | 508 |
| Consensus | ---------- ---------- ---------- ---------- ---------- ---------- ---------- ---------- | |
| Conservation (100% / 0%) | | |

```
                         9,460               9,480               9,500               9,520
                             |                   |                   |                   |
PvCRN11         ---------- ---------- ---------- ---------- ---------- ---------- ---------- ---------- 215
XP_024579260.1  ---------- ---------- ---------- ---------- ---------- ---------- ---------- ---------- 163
XP_024573634.1  ---------- ---------- ---------- ---------- ---------- ---------- ---------- ---------- 169
PvCRN7          ---------- ---------- ---------- ---------- ---------- ---------- ---------- ---------- 753
XP_024585265.1  ---------- ---------- ---------- ---------- ---------- ---------- ---------- ---------- 128
PvCRN18         ---------- ---------- ---------- ---------- ---------- ---------- ---------- ---------- 302
XP_024578414.1  ---------- ---------- ---------- ---------- ---------- ---------- ---------- ---------- 624
XP_024579130.1  ---------- ---------- ---------- ---------- ---------- ---------- ---------- ---------- 345
XP_024584758.1  ---------- ---------- ---------- ---------- ---------- ---------- ---------- ---------- 214
XP_024586066.1  ---------- ---------- ---------- ---------- ---------- ---------- ---------- ---------- 263
XP_024585870.1  ---------- ---------- ---------- ---------- ---------- ---------- ---------- ---------- 280
PvCRN31         ---------- ---------- ---------- ---------- ---------- ---------- ---------- ---------- 583
XP_024577278.1  ---------- ---------- ---------- ---------- ---------- ---------- ---------- ---------- 333
XP_024585562.1  ---------- ---------- ---------- ---------- ---------- ---------- ---------- ---------- 158
XP_024573944.1  ---------- ---------- ---------- ---------- ---------- ---------- ---------- ---------- 592
XP_024579793.1  ---------- ---------- ---------- ---------- ---------- ---------- ---------- ---------- 163
PvCRN15         ---------- ---------- ---------- ---------- ---------- ---------- ---------- ---------- 290
PvCRN26         ---------- ---------- ---------- ---------- ---------- ---------- ---------- ---------- 279
PvCRN16         ---------- ---------- ---------- ---------- ---------- ---------- ---------- ---------- 365
PvCRN23         ---------- ---------- ---------- ---------- ---------- ---------- ---------- ---------- 426
XP_024573063.1  ---------- ---------- ---------- ---------- ---------- ---------- ---------- ---------- 83
PvCRN19         ---------- ---------- ---------- ---------- ---------- ---------- ---------- ---------- 164
XP_024581625.1  ---------- ---------- ---------- ---------- ---------- ---------- ---------- ---------- 135
XP_024583886.1  ---------- ---------- ---------- ---------- ---------- ---------- ---------- ---------- 96
XP_024584409.1  ---------- ---------- ---------- ---------- ---------- ---------- ---------- ---------- 213
XP_024572385.1  ---------- ---------- ---------- ---------- ---------- ---------- ---------- ---------- 310
PvCRN6          ---------- ---------- ---------- ---------- ---------- ---------- ---------- ---------- 372
PvCRN9          ---------- ---------- ---------- ---------- ---------- ---------- ---------- ---------- 611
XP_024580875.1  ---------- ---------- ---------- ---------- ---------- ---------- ---------- ---------- 127
PvCRN24         ---------- ---------- ---------- ---------- ---------- ---------- ---------- ---------- 165
PvCRN35         ---------- ---------- ---------- ---------- ---------- ---------- ---------- ---------- 212
PvCRN12         ---------- ---------- ---------- ---------- ---------- ---------- ---------- ---------- 171
XP_024576927.1  ---------- ---------- ---------- ---------- ---------- ---------- ---------- ---------- 555
XP_024578927.1  ---------- ---------- ---------- ---------- ---------- ---------- ---------- ---------- 327
XP_024586212.1  ---------- ---------- ---------- ---------- ---------- ---------- ---------- ---------- 616
XP_024578078.1  ---------- ---------- ---------- ---------- ---------- ---------- ---------- ---------- 262
XP_024575355.1  ---------- ---------- ---------- ---------- ---------- ---------- ---------- ---------- 295
XP_024584527.1  ---------- ---------- ---------- ---------- ---------- ---------- ---------- ---------- 168
XP_024586664.1  ---------- ---------- ---------- ---------- ---------- ---------- ---------- ---------- 357
PvCRN17         ---------- ---------- ---------- ---------- ---------- ---------- ---------- ---------- 187
PvCRN2          ---------- ---------- ---------- ---------- ---------- ---------- ---------- ---------- 179
XP_024577181.1  ---------- ---------- ---------- ---------- ---------- ---------- ---------- ---------- 207
XP_024581075.1  ---------- ---------- ---------- ---------- ---------- ---------- ---------- ---------- 156
XP_024575884.1  ---------- ---------- ---------- ---------- ---------- ---------- ---------- ---------- 155
XP_024586872.1  ---------- ---------- ---------- ---------- ---------- ---------- ---------- ---------- 182
XP_024573052.1  ---------- ---------- ---------- ---------- ---------- ---------- ---------- ---------- 187
XP_024586054.1  ---------- ---------- ---------- ---------- ---------- ---------- ---------- ---------- 237
PvCRN27         ---------- ---------- ---------- ---------- ---------- ---------- ---------- ---------- 313
PvCRN20         ---------- ---------- ---------- ---------- ---------- ---------- ---------- ---------- 159
PvCRN25         ---------- ---------- ---------- ---------- ---------- ---------- ---------- ---------- 194
PvCRN1          ---------- ---------- ---------- ---------- ---------- ---------- ---------- ---------- 580
PvCRN4          ---------- ---------- ---------- ---------- ---------- ---------- ---------- ---------- 583
PvCRN30         ---------- ---------- ---------- ---------- ---------- ---------- ---------- ---------- 220
PvCRN29         ---------- ---------- ---------- ---------- ---------- ---------- ---------- ---------- 312
XP_024583036.1  ---------- ---------- ---------- ---------- ---------- ---------- ---------- ---------- 681
PvCRN10         ---------- ---------- ---------- ---------- ---------- ---------- ---------- ---------- 217
XP_024577280.1  ---------- ---------- ---------- ---------- ---------- ---------- ---------- ---------- 283
XP_024572924.1  ---------- ---------- ---------- ---------- ---------- ---------- ---------- ---------- 412
PvCRN14         ---------- ---------- ---------- ---------- ---------- ---------- ---------- ---------- 151
XP_024577521.1  ---------- ---------- ---------- ---------- ---------- ---------- ---------- ---------- 108
PvCRN21         ---------- ---------- ---------- ---------- ---------- ---------- ---------- ---------- 479
XP_024574966.1  ---------- ---------- ---------- ---------- ---------- ---------- ---------- ---------- 155
XP_024575372.1  ---------- ---------- ---------- ---------- ---------- ---------- ---------- ---------- 188
XP_024581363.1  ---------- ---------- ---------- ---------- ---------- ---------- ---------- ---------- 229
XP_024583883.1  ---------- ---------- ---------- ---------- ---------- ---------- ---------- ---------- 381
XP_024579844.1  ---------- ---------- ---------- ---------- ---------- ---------- ---------- ---------- 476
XP_024574916.1  ---------- ---------- ---------- ---------- ---------- ---------- ---------- ---------- 621
XP_024574193.1  ---------- ---------- ---------- ---------- ---------- ---------- ---------- ---------- 615
XP_024583155.1  ---------- ---------- ---------- ---------- ---------- ---------- ---------- ---------- 419
XP_024574913.1  ---------- ---------- ---------- ---------- ---------- ---------- ---------- ---------- 623
XP_024578158.1  ---------- ---------- ---------- ---------- ---------- ---------- ---------- ---------- 106
PvCRN22         ---------- ---------- ---------- ---------- ---------- ---------- ---------- ---------- 206
XP_024585001.1  ---------- ---------- ---------- ---------- ---------- ---------- ---------- ---------- 127
XP_024583843.1  ---------- ---------- ---------- ---------- ---------- ---------- ---------- ---------- 534
XP_024578702.1  ---------- ---------- ---------- ---------- ---------- ---------- ---------- ---------- 299
XP_024585805.1  ---------- ---------- ---------- ---------- ---------- ---------- ---------- ---------- 334
XP_024580932.1  ---------- ---------- ---------- ---------- ---------- ---------- ---------- ---------- 508
Consensus       ---------- ---------- ---------- ---------- ---------- ---------- ---------- ----------
100%
Conservation
0%
```

```
                  9,540               9,560               9,580               9,600
                      |                   |                   |                   |
PvCRN11        ---------- ---------- ---------- ---------- ---------- ---------- ---------- ---------- 215
XP_024579260.1 ---------- ---------- ---------- ---------- ---------- ---------- ---------- ---------- 163
XP_024573634.1 ---------- ---------- ---------- ---------- ---------- ---------- ---------- ---------- 169
PvCRN7         ---------- ---------- ---------- ---------- ---------- ---------- ---------- ---------- 753
XP_024585265.1 ---------- ---------- ---------- ---------- ---------- ---------- ---------- ---------- 128
PvCRN18        ---------- ---------- ---------- ---------- ---------- ---------- ---------- ---------- 302
XP_024578414.1 ---------- ---------- ---------- ---------- ---------- ---------- ---------- ---------- 624
XP_024579130.1 ---------- ---------- ---------- ---------- ---------- ---------- ---------- ---------- 345
XP_024584758.1 ---------- ---------- ---------- ---------- ---------- ---------- ---------- ---------- 214
XP_024586066.1 ---------- ---------- ---------- ---------- ---------- ---------- ---------- ---------- 263
XP_024585870.1 ---------- ---------- ---------- ---------- ---------- ---------- ---------- ---------- 280
PvCRN31        ---------- ---------- ---------- ---------- ---------- ---------- ---------- ---------- 583
XP_024577278.1 ---------- ---------- ---------- ---------- ---------- ---------- ---------- ---------- 333
XP_024585562.1 ---------- ---------- ---------- ---------- ---------- ---------- ---------- ---------- 158
XP_024573944.1 ---------- ---------- ---------- ---------- ---------- ---------- ---------- ---------- 592
XP_024579793.1 ---------- ---------- ---------- ---------- ---------- ---------- ---------- ---------- 163
PvCRN15        ---------- ---------- ---------- ---------- ---------- ---------- ---------- ---------- 290
PvCRN26        ---------- ---------- ---------- ---------- ---------- ---------- ---------- ---------- 279
PvCRN16        ---------- ---------- ---------- ---------- ---------- ---------- ---------- ---------- 365
PvCRN23        ---------- ---------- ---------- ---------- ---------- ---------- ---------- ---------- 426
XP_024573063.1 ---------- ---------- ---------- ---------- ---------- ---------- ---------- ---------- 83
PvCRN19        ---------- ---------- ---------- ---------- ---------- ---------- ---------- ---------- 164
XP_024581625.1 ---------- ---------- ---------- ---------- ---------- ---------- ---------- ---------- 135
XP_024583886.1 ---------- ---------- ---------- ---------- ---------- ---------- ---------- ---------- 96
XP_024584409.1 ---------- ---------- ---------- ---------- ---------- ---------- ---------- ---------- 213
XP_024572385.1 ---------- ---------- ---------- ---------- ---------- ---------- ---------- ---------- 310
PvCRN6         ---------- ---------- ---------- ---------- ---------- ---------- ---------- ---------- 372
PvCRN9         ---------- ---------- ---------- ---------- ---------- ---------- ---------- ---------- 611
XP_024580875.1 ---------- ---------- ---------- ---------- ---------- ---------- ---------- ---------- 127
PvCRN24        ---------- ---------- ---------- ---------- ---------- ---------- ---------- ---------- 165
PvCRN35        ---------- ---------- ---------- ---------- ---------- ---------- ---------- ---------- 212
PvCRN12        ---------- ---------- ---------- ---------- ---------- ---------- ---------- ---------- 171
XP_024576927.1 ---------- ---------- ---------- ---------- ---------- ---------- ---------- ---------- 555
XP_024578927.1 ---------- ---------- ---------- ---------- ---------- ---------- ---------- ---------- 327
XP_024586212.1 ---------- ---------- ---------- ---------- ---------- ---------- ---------- ---------- 616
XP_024578078.1 ---------- ---------- ---------- ---------- ---------- ---------- ---------- ---------- 262
XP_024575355.1 ---------- ---------- ---------- ---------- ---------- ---------- ---------- ---------- 295
XP_024584527.1 ---------- ---------- ---------- ---------- ---------- ---------- ---------- ---------- 168
XP_024586664.1 ---------- ---------- ---------- ---------- ---------- ---------- ---------- ---------- 357
PvCRN17        ---------- ---------- ---------- ---------- ---------- ---------- ---------- ---------- 187
PvCRN2         ---------- ---------- ---------- ---------- ---------- ---------- ---------- ---------- 179
XP_024577181.1 ---------- ---------- ---------- ---------- ---------- ---------- ---------- ---------- 207
XP_024581075.1 ---------- ---------- ---------- ---------- ---------- ---------- ---------- ---------- 156
XP_024575884.1 ---------- ---------- ---------- ---------- ---------- ---------- ---------- ---------- 155
XP_024586872.1 ---------- ---------- ---------- ---------- ---------- ---------- ---------- ---------- 182
XP_024573052.1 ---------- ---------- ---------- ---------- ---------- ---------- ---------- ---------- 187
XP_024586054.1 ---------- ---------- ---------- ---------- ---------- ---------- ---------- ---------- 237
PvCRN27        ---------- ---------- ---------- ---------- ---------- ---------- ---------- ---------- 313
PvCRN20        ---------- ---------- ---------- ---------- ---------- ---------- ---------- ---------- 159
PvCRN25        ---------- ---------- ---------- ---------- ---------- ---------- ---------- ---------- 194
PvCRN1         ---------- ---------- ---------- ---------- ---------- ---------- ---------- ---------- 580
PvCRN4         ---------- ---------- ---------- ---------- ---------- ---------- ---------- ---------- 583
PvCRN30        ---------- ---------- ---------- ---------- ---------- ---------- ---------- ---------- 220
PvCRN29        ---------- ---------- ---------- ---------- ---------- ---------- ---------- ---------- 312
XP_024583036.1 ---------- ---------- ---------- ---------- ---------- ---------- ---------- ---------- 681
PvCRN10        ---------- ---------- ---------- ---------- ---------- ---------- ---------- ---------- 217
XP_024577280.1 ---------- ---------- ---------- ---------- ---------- ---------- ---------- ---------- 283
XP_024572924.1 ---------- ---------- ---------- ---------- ---------- ---------- ---------- ---------- 412
PvCRN14        ---------- ---------- ---------- ---------- ---------- ---------- ---------- ---------- 151
XP_024577521.1 ---------- ---------- ---------- ---------- ---------- ---------- ---------- ---------- 108
PvCRN21        ---------- ---------- ---------- ---------- ---------- ---------- ---------- ---------- 479
XP_024574966.1 ---------- ---------- ---------- ---------- ---------- ---------- ---------- ---------- 155
XP_024575372.1 ---------- ---------- ---------- ---------- ---------- ---------- ---------- ---------- 188
XP_024581363.1 ---------- ---------- ---------- ---------- ---------- ---------- ---------- ---------- 229
XP_024583883.1 ---------- ---------- ---------- ---------- ---------- ---------- ---------- ---------- 381
XP_024579844.1 ---------- ---------- ---------- ---------- ---------- ---------- ---------- ---------- 476
XP_024574916.1 ---------- ---------- ---------- ---------- ---------- ---------- ---------- ---------- 621
XP_024574193.1 ---------- ---------- ---------- ---------- ---------- ---------- ---------- ---------- 615
XP_024583155.1 ---------- ---------- ---------- ---------- ---------- ---------- ---------- ---------- 419
XP_024574913.1 ---------- ---------- ---------- ---------- ---------- ---------- ---------- ---------- 623
XP_024578158.1 ---------- ---------- ---------- ---------- ---------- ---------- ---------- ---------- 106
PvCRN22        ---------- ---------- ---------- ---------- ---------- ---------- ---------- ---------- 206
XP_024585001.1 ---------- ---------- ---------- ---------- ---------- ---------- ---------- ---------- 127
XP_024583843.1 ---------- ---------- ---------- ---------- ---------- ---------- ---------- ---------- 534
XP_024578702.1 ---------- ---------- ---------- ---------- ---------- ---------- ---------- ---------- 299
XP_024585805.1 ---------- ---------- ---------- ---------- ---------- ---------- ---------- ---------- 334
XP_024580932.1 ---------- ---------- ---------- ---------- ---------- ---------- ---------- ---------- 508
Consensus      ---------- ---------- ---------- ---------- ---------- ---------- ---------- ----------
         100%
Conservation
           0%
```

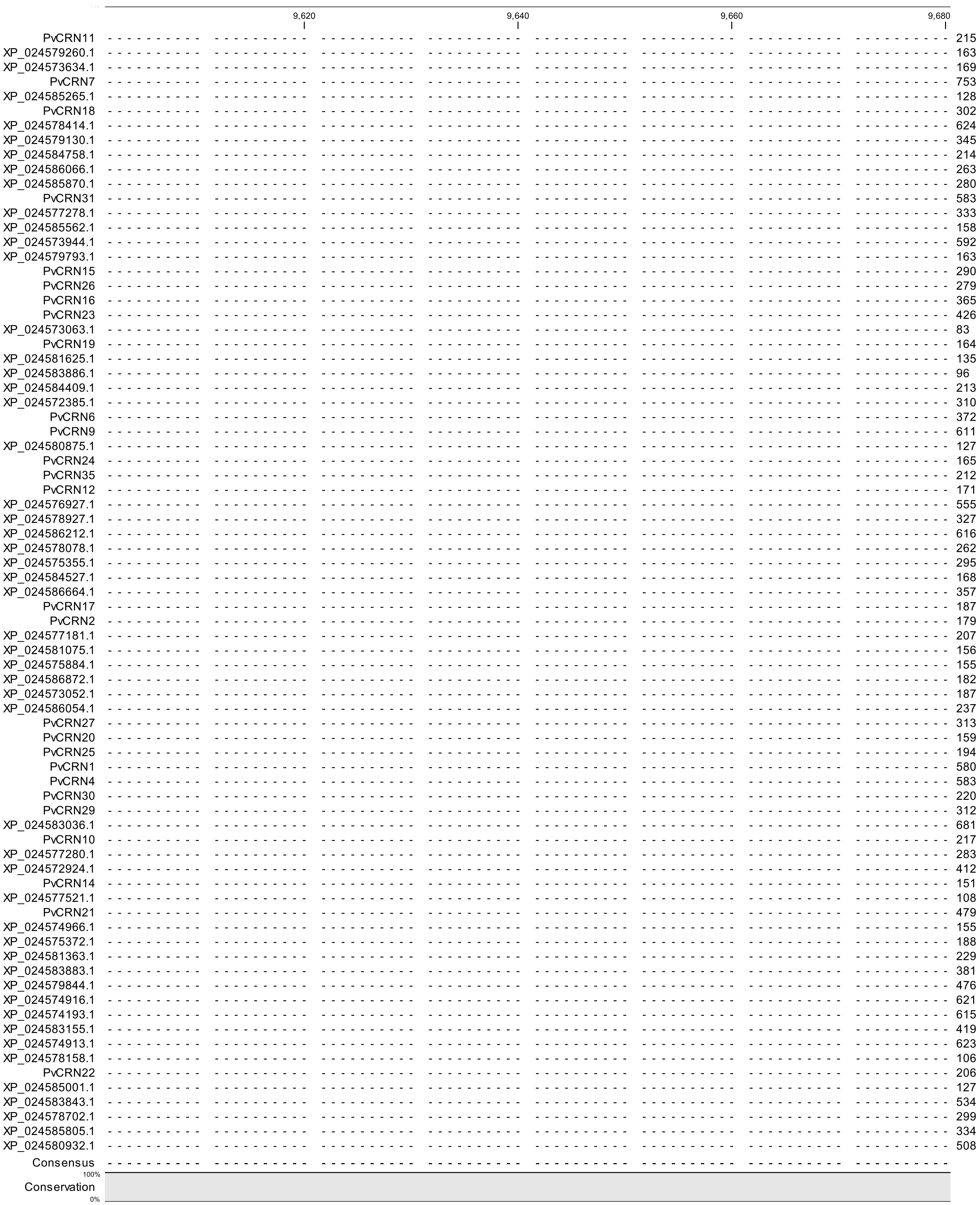
9,620
9,640
9,660
9,680
PvCRN11 215
XP_024579260.1 163
XP_024573634.1 169
PvCRN7 753
XP_024585265.1 128
PvCRN18 302
XP_024578414.1 624
XP_024579130.1 345
XP_024584758.1 214
XP_024586066.1 263
XP_024585870.1 280
PvCRN31 583
XP_024577278.1 333
XP_024585562.1 158
XP_024573944.1 592
XP_024579793.1 163
PvCRN15 290
PvCRN26 279
PvCRN16 365
PvCRN23 426
XP_024573063.1 83
PvCRN19 164
XP_024581625.1 135
XP_024583886.1 96
XP_024584409.1 213
XP_024572385.1 310
PvCRN6 372
PvCRN9 611
XP_024580875.1 127
PvCRN24 165
PvCRN35 212
PvCRN12 171
XP_024576927.1 555
XP_024578927.1 327
XP_024586212.1 616
XP_024578078.1 262
XP_024575355.1 295
XP_024584527.1 168
XP_024586664.1 357
PvCRN17 187
PvCRN2 179
XP_024577181.1 207
XP_024581075.1 156
XP_024575884.1 155
XP_024586872.1 182
XP_024573052.1 187
XP_024586054.1 237
PvCRN27 313
PvCRN20 159
PvCRN25 194
PvCRN1 580
PvCRN4 583
PvCRN30 220
PvCRN29 312
XP_024583036.1 681
PvCRN10 217
XP_024577280.1 283
XP_024572924.1 412
PvCRN14 151
XP_024577521.1 108
PvCRN21 479
XP_024574966.1 155
XP_024575372.1 188
XP_024581363.1 229
XP_024583883.1 381
XP_024579844.1 476
XP_024574916.1 621
XP_024574193.1 615
XP_024583155.1 419
XP_024574913.1 623
XP_024578158.1 106
PvCRN22 206
XP_024585001.1 127
XP_024583843.1 534
XP_024578702.1 299
XP_024585805.1 334
XP_024580932.1 508
Consensus
100%
Conservation
0%

| | 9,700 9,720 9,740 9,760 | |
|---|---|---|
| PvCRN11 | ---------- ---------- ---------- ---------- ---------- ---------- ---------- ---------- | 215 |
| XP_024579260.1 | ---------- ---------- ---------- ---------- ---------- ---------- ---------- ---------- | 163 |
| XP_024573634.1 | ---------- ---------- ---------- ---------- ---------- ---------- ---------- ---------- | 169 |
| PvCRN7 | ---------- ---------- ---------- ---------- ---------- ---------- ---------- ---------- | 753 |
| XP_024585265.1 | ---------- ---------- ---------- ---------- ---------- ---------- ---------- ---------- | 128 |
| PvCRN18 | ---------- ---------- ---------- ---------- ---------- ---------- ---------- ---------- | 302 |
| XP_024578414.1 | ---------- ---------- ---------- ---------- ---------- ---------- ---------- ---------- | 624 |
| XP_024579130.1 | ---------- ---------- ---------- ---------- ---------- ---------- ---------- ---------- | 345 |
| XP_024584758.1 | ---------- ---------- ---------- ---------- ---------- ---------- ---------- ---------- | 214 |
| XP_024586066.1 | ---------- ---------- ---------- ---------- ---------- ---------- ---------- ---------- | 263 |
| XP_024585870.1 | ---------- ---------- ---------- ---------- ---------- ---------- ---------- ---------- | 280 |
| PvCRN31 | ---------- ---------- ---------- ---------- ---------- ---------- ---------- ---------- | 583 |
| XP_024577278.1 | ---------- ---------- ---------- ---------- ---------- ---------- ---------- ---------- | 333 |
| XP_024585562.1 | ---------- ---------- ---------- ---------- ---------- ---------- ---------- ---------- | 158 |
| XP_024573944.1 | ---------- ---------- ---------- ---------- ---------- ---------- ---------- ---------- | 592 |
| XP_024579793.1 | ---------- ---------- ---------- ---------- ---------- ---------- ---------- ---------- | 163 |
| PvCRN15 | ---------- ---------- ---------- ---------- ---------- ---------- ---------- ---------- | 290 |
| PvCRN26 | ---------- ---------- ---------- ---------- ---------- ---------- ---------- ---------- | 279 |
| PvCRN16 | ---------- ---------- ---------- ---------- ---------- ---------- ---------- ---------- | 365 |
| PvCRN23 | ---------- ---------- ---------- ---------- ---------- ---------- ---------- ---------- | 426 |
| XP_024573063.1 | ---------- ---------- ---------- ---------- ---------- ---------- ---------- ---------- | 83 |
| PvCRN19 | ---------- ---------- ---------- ---------- ---------- ---------- ---------- ---------- | 164 |
| XP_024581625.1 | ---------- ---------- ---------- ---------- ---------- ---------- ---------- ---------- | 135 |
| XP_024583886.1 | ---------- ---------- ---------- ---------- ---------- ---------- ---------- ---------- | 96 |
| XP_024584409.1 | ---------- ---------- ---------- ---------- ---------- ---------- ---------- ---------- | 213 |
| XP_024572385.1 | ---------- ---------- ---------- ---------- ---------- ---------- ---------- ---------- | 310 |
| PvCRN6 | ---------- ---------- ---------- ---------- ---------- ---------- ---------- ---------- | 372 |
| PvCRN9 | ---------- ---------- ---------- ---------- ---------- ---------- ---------- ---------- | 611 |
| XP_024580875.1 | ---------- ---------- ---------- ---------- ---------- ---------- ---------- ---------- | 127 |
| PvCRN24 | ---------- ---------- ---------- ---------- ---------- ---------- ---------- ---------- | 165 |
| PvCRN35 | ---------- ---------- ---------- ---------- ---------- ---------- ---------- ---------- | 212 |
| PvCRN12 | ---------- ---------- ---------- ---------- ---------- ---------- ---------- ---------- | 171 |
| XP_024576927.1 | ---------- ---------- ---------- ---------- ---------- ---------- ---------- ---------- | 555 |
| XP_024578927.1 | ---------- ---------- ---------- ---------- ---------- ---------- ---------- ---------- | 327 |
| XP_024586212.1 | ---------- ---------- ---------- ---------- ---------- ---------- ---------- ---------- | 616 |
| XP_024578078.1 | ---------- ---------- ---------- ---------- ---------- ---------- ---------- ---------- | 262 |
| XP_024575355.1 | ---------- ---------- ---------- ---------- ---------- ---------- ---------- ---------- | 295 |
| XP_024584527.1 | ---------- ---------- ---------- ---------- ---------- ---------- ---------- ---------- | 168 |
| XP_024586664.1 | ---------- ---------- ---------- ---------- ---------- ---------- ---------- ---------- | 357 |
| PvCRN17 | ---------- ---------- ---------- ---------- ---------- ---------- ---------- ---------- | 187 |
| PvCRN2 | ---------- ---------- ---------- ---------- ---------- ---------- ---------- ---------- | 179 |
| XP_024577181.1 | ---------- ---------- ---------- ---------- ---------- ---------- ---------- ---------- | 207 |
| XP_024581075.1 | ---------- ---------- ---------- ---------- ---------- ---------- ---------- ---------- | 156 |
| XP_024575884.1 | ---------- ---------- ---------- ---------- ---------- ---------- ---------- ---------- | 155 |
| XP_024586872.1 | ---------- ---------- ---------- ---------- ---------- ---------- ---------- ---------- | 182 |
| XP_024573052.1 | ---------- ---------- ---------- ---------- ---------- ---------- ---------- ---------- | 187 |
| XP_024586054.1 | ---------- ---------- ---------- ---------- ---------- ---------- ---------- ---------- | 237 |
| PvCRN27 | ---------- ---------- ---------- ---------- ---------- ---------- ---------- ---------- | 313 |
| PvCRN20 | ---------- ---------- ---------- ---------- ---------- ---------- ---------- ---------- | 159 |
| PvCRN25 | ---------- ---------- ---------- ---------- ---------- ---------- ---------- ---------- | 194 |
| PvCRN1 | ---------- ---------- ---------- ---------- ---------- ---------- ---------- ---------- | 580 |
| PvCRN4 | ---------- ---------- ---------- ---------- ---------- ---------- ---------- ---------- | 583 |
| PvCRN30 | ---------- ---------- ---------- ---------- ---------- ---------- ---------- ---------- | 220 |
| PvCRN29 | ---------- ---------- ---------- ---------- ---------- ---------- ---------- ---------- | 312 |
| XP_024583036.1 | ---------- ---------- ---------- ---------- ---------- ---------- ---------- ---------- | 681 |
| PvCRN10 | ---------- ---------- ---------- ---------- ---------- ---------- ---------- ---------- | 217 |
| XP_024577280.1 | ---------- ---------- ---------- ---------- ---------- ---------- ---------- ---------- | 283 |
| XP_024572924.1 | ---------- ---------- ---------- ---------- ---------- ---------- ---------- ---------- | 412 |
| PvCRN14 | ---------- ---------- ---------- ---------- ---------- ---------- ---------- ---------- | 151 |
| XP_024577521.1 | ---------- ---------- ---------- ---------- ---------- ---------- ---------- ---------- | 108 |
| PvCRN21 | ---------- ---------- ---------- ---------- ---------- ---------- ---------- ---------- | 479 |
| XP_024574966.1 | ---------- ---------- ---------- ---------- ---------- ---------- ---------- ---------- | 155 |
| XP_024575372.1 | ---------- ---------- ---------- ---------- ---------- ---------- ---------- ---------- | 188 |
| XP_024581363.1 | ---------- ---------- ---------- ---------- ---------- ---------- ---------- ---------- | 229 |
| XP_024583883.1 | ---------- ---------- ---------- ---------- ---------- ---------- ---------- ---------- | 381 |
| XP_024579844.1 | ---------- ---------- ---------- ---------- ---------- ---------- ---------- ---------- | 476 |
| XP_024574916.1 | ---------- ---------- ---------- ---------- ---------- ---------- ---------- ---------- | 621 |
| XP_024574193.1 | ---------- ---------- ---------- ---------- ---------- ---------- ---------- ---------- | 615 |
| XP_024583155.1 | ---------- ---------- ---------- ---------- ---------- ---------- ---------- ---------- | 419 |
| XP_024574913.1 | ---------- ---------- ---------- ---------- ---------- ---------- ---------- ---------- | 623 |
| XP_024578158.1 | ---------- ---------- ---------- ---------- ---------- ---------- ---------- ---------- | 106 |
| PvCRN22 | ---------- ---------- ---------- ---------- ---------- ---------- ---------- ---------- | 206 |
| XP_024585001.1 | ---------- ---------- ---------- ---------- ---------- ---------- ---------- ---------- | 127 |
| XP_024583843.1 | ---------- ---------- ---------- ---------- ---------- ---------- ---------- ---------- | 534 |
| XP_024578702.1 | ---------- ---------- ---------- ---------- ---------- ---------- ---------- ---------- | 299 |
| XP_024585805.1 | ---------- ---------- ---------- ---------- ---------- ---------- ---------- ---------- | 334 |
| XP_024580932.1 | ---------- ---------- ---------- ---------- ---------- ---------- ---------- ---------- | 508 |
| Consensus | ---------- ---------- ---------- ---------- ---------- ---------- ---------- ---------- | |
| Conservation (100% – 0%) | | |

| Sequence | 9,780 · 9,800 · 9,820 · 9,840 | End |
|---|---|---|
| PvCRN11 | ---------- ---------- ---------- ---------- ---------- ---------- ---------- ---------- | 215 |
| XP_024579260.1 | ---------- ---------- ---------- ---------- ---------- ---------- ---------- ---------- | 163 |
| XP_024573634.1 | ---------- ---------- ---------- ---------- ---------- ---------- ---------- ---------- | 169 |
| PvCRN7 | ---------- ---------- ---------- ---------- ---------- ---------- ---------- ---------- | 753 |
| XP_024585265.1 | ---------- ---------- ---------- ---------- ---------- ---------- ---------- ---------- | 128 |
| PvCRN18 | ---------- ---------- ---------- ---------- ---------- ---------- ---------- ---------- | 302 |
| XP_024578414.1 | ---------- ---------- ---------- ---------- ---------- ---------- ---------- ---------- | 624 |
| XP_024579130.1 | ---------- ---------- ---------- ---------- ---------- ---------- ---------- ---------- | 345 |
| XP_024584758.1 | ---------- ---------- ---------- ---------- ---------- ---------- ---------- ---------- | 214 |
| XP_024586066.1 | ---------- ---------- ---------- ---------- ---------- ---------- ---------- ---------- | 263 |
| XP_024585870.1 | ---------- ---------- ---------- ---------- ---------- ---------- ---------- ---------- | 280 |
| PvCRN31 | ---------- ---------- ---------- ---------- ---------- ---------- ---------- ---------- | 583 |
| XP_024577278.1 | ---------- ---------- ---------- ---------- ---------- ---------- ---------- ---------- | 333 |
| XP_024585562.1 | ---------- ---------- ---------- ---------- ---------- ---------- ---------- ---------- | 158 |
| XP_024573944.1 | ---------- ---------- ---------- ---------- ---------- ---------- ---------- ---------- | 592 |
| XP_024579793.1 | ---------- ---------- ---------- ---------- ---------- ---------- ---------- ---------- | 163 |
| PvCRN15 | ---------- ---------- ---------- ---------- ---------- ---------- ---------- ---------- | 290 |
| PvCRN26 | ---------- ---------- ---------- ---------- ---------- ---------- ---------- ---------- | 279 |
| PvCRN16 | ---------- ---------- ---------- ---------- ---------- ---------- ---------- ---------- | 365 |
| PvCRN23 | ---------- ---------- ---------- ---------- ---------- ---------- ---------- ---------- | 426 |
| XP_024573063.1 | ---------- ---------- ---------- ---------- ---------- ---------- ---------- ---------- | 83 |
| PvCRN19 | ---------- ---------- ---------- ---------- ---------- ---------- ---------- ---------- | 164 |
| XP_024581625.1 | ---------- ---------- ---------- ---------- ---------- ---------- ---------- ---------- | 135 |
| XP_024583886.1 | ---------- ---------- ---------- ---------- ---------- ---------- ---------- ---------- | 96 |
| XP_024584409.1 | ---------- ---------- ---------- ---------- ---------- ---------- ---------- ---------- | 213 |
| XP_024572385.1 | ---------- ---------- ---------- ---------- ---------- ---------- ---------- ---------- | 310 |
| PvCRN6 | ---------- ---------- ---------- ---------- ---------- ---------- ---------- ---------- | 372 |
| PvCRN9 | ---------- ---------- ---------- ---------- ---------- ---------- ---------- ---------- | 611 |
| XP_024580875.1 | ---------- ---------- ---------- ---------- ---------- ---------- ---------- ---------- | 127 |
| PvCRN24 | ---------- ---------- ---------- ---------- ---------- ---------- ---------- ---------- | 165 |
| PvCRN35 | ---------- ---------- ---------- ---------- ---------- ---------- ---------- ---------- | 212 |
| PvCRN12 | ---------- ---------- ---------- ---------- ---------- ---------- ---------- ---------- | 171 |
| XP_024576927.1 | ---------- ---------- ---------- ---------- ---------- ---------- ---------- ---------- | 555 |
| XP_024578927.1 | ---------- ---------- ---------- ---------- ---------- ---------- ---------- ---------- | 327 |
| XP_024586212.1 | ---------- ---------- ---------- ---------- ---------- ---------- ---------- ---------- | 616 |
| XP_024578078.1 | ---------- ---------- ---------- ---------- ---------- ---------- ---------- ---------- | 262 |
| XP_024575355.1 | ---------- ---------- ---------- ---------- ---------- ---------- ---------- ---------- | 295 |
| XP_024584527.1 | ---------- ---------- ---------- ---------- ---------- ---------- ---------- ---------- | 168 |
| XP_024586664.1 | ---------- ---------- ---------- ---------- ---------- ---------- ---------- ---------- | 357 |
| PvCRN17 | ---------- ---------- ---------- ---------- ---------- ---------- ---------- ---------- | 187 |
| PvCRN2 | ---------- ---------- ---------- ---------- ---------- ---------- ---------- ---------- | 179 |
| XP_024577181.1 | ---------- ---------- ---------- ---------- ---------- ---------- ---------- ---------- | 207 |
| XP_024581075.1 | ---------- ---------- ---------- ---------- ---------- ---------- ---------- ---------- | 156 |
| XP_024575884.1 | ---------- ---------- ---------- ---------- ---------- ---------- ---------- ---------- | 155 |
| XP_024586872.1 | ---------- ---------- ---------- ---------- ---------- ---------- ---------- ---------- | 182 |
| XP_024573052.1 | ---------- ---------- ---------- ---------- ---------- ---------- ---------- ---------- | 187 |
| XP_024586054.1 | ---------- ---------- ---------- ---------- ---------- ---------- ---------- ---------- | 237 |
| PvCRN27 | ---------- ---------- ---------- ---------- ---------- ---------- ---------- ---------- | 313 |
| PvCRN20 | ---------- ---------- ---------- ---------- ---------- ---------- ---------- ---------- | 159 |
| PvCRN25 | ---------- ---------- ---------- ---------- ---------- ---------- ---------- ---------- | 194 |
| PvCRN1 | ---------- ---------- ---------- ---------- ---------- ---------- ---------- ---------- | 580 |
| PvCRN4 | ---------- ---------- ---------- ---------- ---------- ---------- ---------- ---------- | 583 |
| PvCRN30 | ---------- ---------- ---------- ---------- ---------- ---------- ---------- ---------- | 220 |
| PvCRN29 | ---------- ---------- ---------- ---------- ---------- ---------- ---------- ---------- | 312 |
| XP_024583036.1 | ---------- ---------- ---------- ---------- ---------- ---------- ---------- ---------- | 681 |
| PvCRN10 | ---------- ---------- ---------- ---------- ---------- ---------- ---------- ---------- | 217 |
| XP_024577280.1 | ---------- ---------- ---------- ---------- ---------- ---------- ---------- ---------- | 283 |
| XP_024572924.1 | ---------- ---------- ---------- ---------- ---------- ---------- ---------- ---------- | 412 |
| PvCRN14 | ---------- ---------- ---------- ---------- ---------- ---------- ---------- ---------- | 151 |
| XP_024577521.1 | ---------- ---------- ---------- ---------- ---------- ---------- ---------- ---------- | 108 |
| PvCRN21 | ---------- ---------- ---------- ---------- ---------- ---------- ---------- ---------- | 479 |
| XP_024574966.1 | ---------- ---------- ---------- ---------- ---------- ---------- ---------- ---------- | 155 |
| XP_024575372.1 | ---------- ---------- ---------- ---------- ---------- ---------- ---------- ---------- | 188 |
| XP_024581363.1 | ---------- ---------- ---------- ---------- ---------- ---------- ---------- ---------- | 229 |
| XP_024583883.1 | ---------- ---------- ---------- ---------- ---------- ---------- ---------- ---------- | 381 |
| XP_024579844.1 | ---------- ---------- ---------- ---------- ---------- ---------- ---------- ---------- | 476 |
| XP_024574916.1 | ---------- ---------- ---------- ---------- ---------- ---------- ---------- ---------- | 621 |
| XP_024574193.1 | ---------- ---------- ---------- ---------- ---------- ---------- ---------- ---------- | 615 |
| XP_024583155.1 | ---------- ---------- ---------- ---------- ---------- ---------- ---------- ---------- | 419 |
| XP_024574913.1 | ---------- ---------- ---------- ---------- ---------- ---------- ---------- ---------- | 623 |
| XP_024578158.1 | ---------- ---------- ---------- ---------- ---------- ---------- ---------- ---------- | 106 |
| PvCRN22 | ---------- ---------- ---------- ---------- ---------- ---------- ---------- ---------- | 206 |
| XP_024585001.1 | ---------- ---------- ---------- ---------- ---------- ---------- ---------- ---------- | 127 |
| XP_024583843.1 | ---------- ---------- ---------- ---------- ---------- ---------- ---------- ---------- | 534 |
| XP_024578702.1 | ---------- ---------- ---------- ---------- ---------- ---------- ---------- ---------- | 299 |
| XP_024585805.1 | ---------- ---------- ---------- ---------- ---------- ---------- ---------- ---------- | 334 |
| XP_024580932.1 | ---------- ---------- ---------- ---------- ---------- ---------- ---------- ---------- | 508 |
| Consensus | ---------- ---------- ---------- ---------- ---------- ---------- ---------- ---------- | |
| Conservation (100% – 0%) | | |

| | 9,860 · 9,880 · 9,900 · 9,920 | |
|---|---|---|
| PvCRN11 | ---------- ---------- ---------- ---------- ---------- ---------- ---------- ---------- | 215 |
| XP_024579260.1 | ---------- ---------- ---------- ---------- ---------- ---------- ---------- ---------- | 163 |
| XP_024573634.1 | ---------- ---------- ---------- ---------- ---------- ---------- ---------- ---------- | 169 |
| PvCRN7 | ---------- ---------- ---------- ---------- ---------- ---------- ---------- ---------- | 753 |
| XP_024585265.1 | ---------- ---------- ---------- ---------- ---------- ---------- ---------- ---------- | 128 |
| PvCRN18 | ---------- ---------- ---------- ---------- ---------- ---------- ---------- ---------- | 302 |
| XP_024578414.1 | ---------- ---------- ---------- ---------- ---------- ---------- ---------- ---------- | 624 |
| XP_024579130.1 | ---------- ---------- ---------- ---------- ---------- ---------- ---------- ---------- | 345 |
| XP_024584758.1 | ---------- ---------- ---------- ---------- ---------- ---------- ---------- ---------- | 214 |
| XP_024586066.1 | ---------- ---------- ---------- ---------- ---------- ---------- ---------- ---------- | 263 |
| XP_024585870.1 | ---------- ---------- ---------- ---------- ---------- ---------- ---------- ---------- | 280 |
| PvCRN31 | ---------- ---------- ---------- ---------- ---------- ---------- ---------- ---------- | 583 |
| XP_024577278.1 | ---------- ---------- ---------- ---------- ---------- ---------- ---------- ---------- | 333 |
| XP_024585562.1 | ---------- ---------- ---------- ---------- ---------- ---------- ---------- ---------- | 158 |
| XP_024573944.1 | ---------- ---------- ---------- ---------- ---------- ---------- ---------- ---------- | 592 |
| XP_024579793.1 | ---------- ---------- ---------- ---------- ---------- ---------- ---------- ---------- | 163 |
| PvCRN15 | ---------- ---------- ---------- ---------- ---------- ---------- ---------- ---------- | 290 |
| PvCRN26 | ---------- ---------- ---------- ---------- ---------- ---------- ---------- ---------- | 279 |
| PvCRN16 | ---------- ---------- ---------- ---------- ---------- ---------- ---------- ---------- | 365 |
| PvCRN23 | ---------- ---------- ---------- ---------- ---------- ---------- ---------- ---------- | 426 |
| XP_024573063.1 | ---------- ---------- ---------- ---------- ---------- ---------- ---------- ---------- | 83 |
| PvCRN19 | ---------- ---------- ---------- ---------- ---------- ---------- ---------- ---------- | 164 |
| XP_024581625.1 | ---------- ---------- ---------- ---------- ---------- ---------- ---------- ---------- | 135 |
| XP_024583886.1 | ---------- ---------- ---------- ---------- ---------- ---------- ---------- ---------- | 96 |
| XP_024584409.1 | ---------- ---------- ---------- ---------- ---------- ---------- ---------- ---------- | 213 |
| XP_024572385.1 | ---------- ---------- ---------- ---------- ---------- ---------- ---------- ---------- | 310 |
| PvCRN6 | ---------- ---------- ---------- ---------- ---------- ---------- ---------- ---------- | 372 |
| PvCRN9 | ---------- ---------- ---------- ---------- ---------- ---------- ---------- ---------- | 611 |
| XP_024580875.1 | ---------- ---------- ---------- ---------- ---------- ---------- ---------- ---------- | 127 |
| PvCRN24 | ---------- ---------- ---------- ---------- ---------- ---------- ---------- ---------- | 165 |
| PvCRN35 | ---------- ---------- ---------- ---------- ---------- ---------- ---------- ---------- | 212 |
| PvCRN12 | ---------- ---------- ---------- ---------- ---------- ---------- ---------- ---------- | 171 |
| XP_024576927.1 | ---------- ---------- ---------- ---------- ---------- ---------- ---------- ---------- | 555 |
| XP_024578927.1 | ---------- ---------- ---------- ---------- ---------- ---------- ---------- ---------- | 327 |
| XP_024586212.1 | ---------- ---------- ---------- ---------- ---------- ---------- ---------- ---------- | 616 |
| XP_024578078.1 | ---------- ---------- ---------- ---------- ---------- ---------- ---------- ---------- | 262 |
| XP_024575355.1 | ---------- ---------- ---------- ---------- ---------- ---------- ---------- ---------- | 295 |
| XP_024584527.1 | ---------- ---------- ---------- ---------- ---------- ---------- ---------- ---------- | 168 |
| XP_024586664.1 | ---------- ---------- ---------- ---------- ---------- ---------- ---------- ---------- | 357 |
| PvCRN17 | ---------- ---------- ---------- ---------- ---------- ---------- ---------- ---------- | 187 |
| PvCRN2 | ---------- ---------- ---------- ---------- ---------- ---------- ---------- ---------- | 179 |
| XP_024577181.1 | ---------- ---------- ---------- ---------- ---------- ---------- ---------- ---------- | 207 |
| XP_024581075.1 | ---------- ---------- ---------- ---------- ---------- ---------- ---------- ---------- | 156 |
| XP_024575884.1 | ---------- ---------- ---------- ---------- ---------- ---------- ---------- ---------- | 155 |
| XP_024586872.1 | ---------- ---------- ---------- ---------- ---------- ---------- ---------- ---------- | 182 |
| XP_024573052.1 | ---------- ---------- ---------- ---------- ---------- ---------- ---------- ---------- | 187 |
| XP_024586054.1 | ---------- ---------- ---------- ---------- ---------- ---------- ---------- ---------- | 237 |
| PvCRN27 | ---------- ---------- ---------- ---------- ---------- ---------- ---------- ---------- | 313 |
| PvCRN20 | ---------- ---------- ---------- ---------- ---------- ---------- ---------- ---------- | 159 |
| PvCRN25 | ---------- ---------- ---------- ---------- ---------- ---------- ---------- ---------- | 194 |
| PvCRN1 | ---------- ---------- ---------- ---------- ---------- ---------- ---------- ---------- | 580 |
| PvCRN4 | ---------- ---------- ---------- ---------- ---------- ---------- ---------- ---------- | 583 |
| PvCRN30 | ---------- ---------- ---------- ---------- ---------- ---------- ---------- ---------- | 220 |
| PvCRN29 | ---------- ---------- ---------- ---------- ---------- ---------- ---------- ---------- | 312 |
| XP_024583036.1 | ---------- ---------- ---------- ---------- ---------- ---------- ---------- ---------- | 681 |
| PvCRN10 | ---------- ---------- ---------- ---------- ---------- ---------- ---------- ---------- | 217 |
| XP_024577280.1 | ---------- ---------- ---------- ---------- ---------- ---------- ---------- ---------- | 283 |
| XP_024572924.1 | ---------- ---------- ---------- ---------- ---------- ---------- ---------- ---------- | 412 |
| PvCRN14 | ---------- ---------- ---------- ---------- ---------- ---------- ---------- ---------- | 151 |
| XP_024577521.1 | ---------- ---------- ---------- ---------- ---------- ---------- ---------- ---------- | 108 |
| PvCRN21 | ---------- ---------- ---------- ---------- ---------- ---------- ---------- ---------- | 479 |
| XP_024574966.1 | ---------- ---------- ---------- ---------- ---------- ---------- ---------- ---------- | 155 |
| XP_024575372.1 | ---------- ---------- ---------- ---------- ---------- ---------- ---------- ---------- | 188 |
| XP_024581363.1 | ---------- ---------- ---------- ---------- ---------- ---------- ---------- ---------- | 229 |
| XP_024583883.1 | ---------- ---------- ---------- ---------- ---------- ---------- ---------- ---------- | 381 |
| XP_024579844.1 | ---------- ---------- ---------- ---------- ---------- ---------- ---------- ---------- | 476 |
| XP_024574916.1 | ---------- ---------- ---------- ---------- ---------- ---------- ---------- ---------- | 621 |
| XP_024574193.1 | ---------- ---------- ---------- ---------- ---------- ---------- ---------- ---------- | 615 |
| XP_024583155.1 | ---------- ---------- ---------- ---------- ---------- ---------- ---------- ---------- | 419 |
| XP_024574913.1 | ---------- ---------- ---------- ---------- ---------- ---------- ---------- ---------- | 623 |
| XP_024578158.1 | ---------- ---------- ---------- ---------- ---------- ---------- ---------- ---------- | 106 |
| PvCRN22 | ---------- ---------- ---------- ---------- ---------- ---------- ---------- ---------- | 206 |
| XP_024585001.1 | ---------- ---------- ---------- ---------- ---------- ---------- ---------- ---------- | 127 |
| XP_024583843.1 | ---------- ---------- ---------- ---------- ---------- ---------- ---------- ---------- | 534 |
| XP_024578702.1 | ---------- ---------- ---------- ---------- ---------- ---------- ---------- ---------- | 299 |
| XP_024585805.1 | ---------- ---------- ---------- ---------- ---------- ---------- ---------- ---------- | 334 |
| XP_024580932.1 | ---------- ---------- ---------- ---------- ---------- ---------- ---------- ---------- | 508 |
| Consensus | ---------- ---------- ---------- ---------- ---------- ---------- ---------- ---------- | |
| Conservation (100% / 0%) | | |

| | 9,940 9,960 9,980 10,000 | |
|---|---|---|
| PvCRN11 | --------- --------- --------- --------- --------- --------- --------- --------- | 215 |
| XP_024579260.1 | --------- --------- --------- --------- --------- --------- --------- --------- | 163 |
| XP_024573634.1 | --------- --------- --------- --------- --------- --------- --------- --------- | 169 |
| PvCRN7 | --------- --------- --------- --------- --------- --------- --------- --------- | 753 |
| XP_024585265.1 | --------- --------- --------- --------- --------- --------- --------- --------- | 128 |
| PvCRN18 | --------- --------- --------- --------- --------- --------- --------- --------- | 302 |
| XP_024578414.1 | --------- --------- --------- --------- --------- --------- --------- --------- | 624 |
| XP_024579130.1 | --------- --------- --------- --------- --------- --------- --------- --------- | 345 |
| XP_024584758.1 | --------- --------- --------- --------- --------- --------- --------- --------- | 214 |
| XP_024586066.1 | --------- --------- --------- --------- --------- --------- --------- --------- | 263 |
| XP_024585870.1 | --------- --------- --------- --------- --------- --------- --------- --------- | 280 |
| PvCRN31 | --------- --------- --------- --------- --------- --------- --------- --------- | 583 |
| XP_024577278.1 | --------- --------- --------- --------- --------- --------- --------- --------- | 333 |
| XP_024585562.1 | --------- --------- --------- --------- --------- --------- --------- --------- | 158 |
| XP_024573944.1 | --------- --------- --------- --------- --------- --------- --------- --------- | 592 |
| XP_024579793.1 | --------- --------- --------- --------- --------- --------- --------- --------- | 163 |
| PvCRN15 | --------- --------- --------- --------- --------- --------- --------- --------- | 290 |
| PvCRN26 | --------- --------- --------- --------- --------- --------- --------- --------- | 279 |
| PvCRN16 | --------- --------- --------- --------- --------- --------- --------- --------- | 365 |
| PvCRN23 | --------- --------- --------- --------- --------- --------- --------- --------- | 426 |
| XP_024573063.1 | --------- --------- --------- --------- --------- --------- --------- --------- | 83 |
| PvCRN19 | --------- --------- --------- --------- --------- --------- --------- --------- | 164 |
| XP_024581625.1 | --------- --------- --------- --------- --------- --------- --------- --------- | 135 |
| XP_024583886.1 | --------- --------- --------- --------- --------- --------- --------- --------- | 96 |
| XP_024584409.1 | --------- --------- --------- --------- --------- --------- --------- --------- | 213 |
| XP_024572385.1 | --------- --------- --------- --------- --------- --------- --------- --------- | 310 |
| PvCRN6 | --------- --------- --------- --------- --------- --------- --------- --------- | 372 |
| PvCRN9 | --------- --------- --------- --------- --------- --------- --------- --------- | 611 |
| XP_024580875.1 | --------- --------- --------- --------- --------- --------- --------- --------- | 127 |
| PvCRN24 | --------- --------- --------- --------- --------- --------- --------- --------- | 165 |
| PvCRN35 | --------- --------- --------- --------- --------- --------- --------- --------- | 212 |
| PvCRN12 | --------- --------- --------- --------- --------- --------- --------- --------- | 171 |
| XP_024576927.1 | --------- --------- --------- --------- --------- --------- --------- --------- | 555 |
| XP_024578927.1 | --------- --------- --------- --------- --------- --------- --------- --------- | 327 |
| XP_024586212.1 | --------- --------- --------- --------- --------- --------- --------- --------- | 616 |
| XP_024578078.1 | --------- --------- --------- --------- --------- --------- --------- --------- | 262 |
| XP_024575355.1 | --------- --------- --------- --------- --------- --------- --------- --------- | 295 |
| XP_024584527.1 | --------- --------- --------- --------- --------- --------- --------- --------- | 168 |
| XP_024586664.1 | --------- --------- --------- --------- --------- --------- --------- --------- | 357 |
| PvCRN17 | --------- --------- --------- --------- --------- --------- --------- --------- | 187 |
| PvCRN2 | --------- --------- --------- --------- --------- --------- --------- --------- | 179 |
| XP_024577181.1 | --------- --------- --------- --------- --------- --------- --------- --------- | 207 |
| XP_024581075.1 | --------- --------- --------- --------- --------- --------- --------- --------- | 156 |
| XP_024575884.1 | --------- --------- --------- --------- --------- --------- --------- --------- | 155 |
| XP_024586872.1 | --------- --------- --------- --------- --------- --------- --------- --------- | 182 |
| XP_024573052.1 | --------- --------- --------- --------- --------- --------- --------- --------- | 187 |
| XP_024586054.1 | --------- --------- --------- --------- --------- --------- --------- --------- | 237 |
| PvCRN27 | --------- --------- --------- --------- --------- --------- --------- --------- | 313 |
| PvCRN20 | --------- --------- --------- --------- --------- --------- --------- --------- | 159 |
| PvCRN25 | --------- --------- --------- --------- --------- --------- --------- --------- | 194 |
| PvCRN1 | --------- --------- --------- --------- --------- --------- --------- --------- | 580 |
| PvCRN4 | --------- --------- --------- --------- --------- --------- --------- --------- | 583 |
| PvCRN30 | --------- --------- --------- --------- --------- --------- --------- --------- | 220 |
| PvCRN29 | --------- --------- --------- --------- --------- --------- --------- --------- | 312 |
| XP_024583036.1 | --------- --------- --------- --------- --------- --------- --------- --------- | 681 |
| PvCRN10 | --------- --------- --------- --------- --------- --------- --------- --------- | 217 |
| XP_024577280.1 | --------- --------- --------- --------- --------- --------- --------- --------- | 283 |
| XP_024572924.1 | --------- --------- --------- --------- --------- --------- --------- --------- | 412 |
| PvCRN14 | --------- --------- --------- --------- --------- --------- --------- --------- | 151 |
| XP_024577521.1 | --------- --------- --------- --------- --------- --------- --------- --------- | 108 |
| PvCRN21 | --------- --------- --------- --------- --------- --------- --------- --------- | 479 |
| XP_024574966.1 | --------- --------- --------- --------- --------- --------- --------- --------- | 155 |
| XP_024575372.1 | --------- --------- --------- --------- --------- --------- --------- --------- | 188 |
| XP_024581363.1 | --------- --------- --------- --------- --------- --------- --------- --------- | 229 |
| XP_024583883.1 | --------- --------- --------- --------- --------- --------- --------- --------- | 381 |
| XP_024579844.1 | --------- --------- --------- --------- --------- --------- --------- --------- | 476 |
| XP_024574916.1 | --------- --------- --------- --------- --------- --------- --------- --------- | 621 |
| XP_024574193.1 | --------- --------- --------- --------- --------- --------- --------- --------- | 615 |
| XP_024583155.1 | --------- --------- --------- --------- --------- --------- --------- --------- | 419 |
| XP_024574913.1 | --------- --------- --------- --------- --------- --------- --------- --------- | 623 |
| XP_024578158.1 | --------- --------- --------- --------- --------- --------- --------- --------- | 106 |
| PvCRN22 | --------- --------- --------- --------- --------- --------- --------- --------- | 206 |
| XP_024585001.1 | --------- --------- --------- --------- --------- --------- --------- --------- | 127 |
| XP_024583843.1 | --------- --------- --------- --------- --------- --------- --------- --------- | 534 |
| XP_024578702.1 | --------- --------- --------- --------- --------- --------- --------- --------- | 299 |
| XP_024585805.1 | --------- --------- --------- --------- --------- --------- --------- --------- | 334 |
| XP_024580932.1 | --------- --------- --------- --------- --------- --------- --------- --------- | 508 |
| Consensus | --------- --------- --------- --------- --------- --------- --------- --------- | |

Conservation (100% / 0%)

| | 10,020 | |
|---|---|---|
| PvCRN11 | ---------- ---------- | 215 |
| XP_024579260.1 | ---------- ---------- | 163 |
| XP_024573634.1 | ---------- ---------- | 169 |
| PvCRN7 | ---------- ---------- | 753 |
| XP_024585265.1 | ---------- ---------- | 128 |
| PvCRN18 | ---------- ---------- | 302 |
| XP_024578414.1 | ---------- ---------- | 624 |
| XP_024579130.1 | ---------- ---------- | 345 |
| XP_024584758.1 | ---------- ---------- | 214 |
| XP_024586066.1 | ---------- ---------- | 263 |
| XP_024585870.1 | ---------- ---------- | 280 |
| PvCRN31 | ---------- ---------- | 583 |
| XP_024577278.1 | ---------- ---------- | 333 |
| XP_024585562.1 | ---------- ---------- | 158 |
| XP_024573944.1 | ---------- ---------- | 592 |
| XP_024579793.1 | ---------- ---------- | 163 |
| PvCRN15 | ---------- ---------- | 290 |
| PvCRN26 | ---------- ---------- | 279 |
| PvCRN16 | ---------- ---------- | 365 |
| PvCRN23 | ---------- ---------- | 426 |
| XP_024573063.1 | ---------- ---------- | 83 |
| PvCRN19 | ---------- ---------- | 164 |
| XP_024581625.1 | ---------- ---------- | 135 |
| XP_024583886.1 | ---------- ---------- | 96 |
| XP_024584409.1 | ---------- ---------- | 213 |
| XP_024572385.1 | ---------- ---------- | 310 |
| PvCRN6 | ---------- ---------- | 372 |
| PvCRN9 | ---------- ---------- | 611 |
| XP_024580875.1 | ---------- ---------- | 127 |
| PvCRN24 | ---------- ---------- | 165 |
| PvCRN35 | ---------- ---------- | 212 |
| PvCRN12 | ---------- ---------- | 171 |
| XP_024576927.1 | ---------- ---------- | 555 |
| XP_024578927.1 | ---------- ---------- | 327 |
| XP_024586212.1 | ---------- ---------- | 616 |
| XP_024578078.1 | ---------- ---------- | 262 |
| XP_024575355.1 | ---------- ---------- | 295 |
| XP_024584527.1 | ---------- ---------- | 168 |
| XP_024586664.1 | ---------- ---------- | 357 |
| PvCRN17 | ---------- ---------- | 187 |
| PvCRN2 | ---------- ---------- | 179 |
| XP_024577181.1 | ---------- ---------- | 207 |
| XP_024581075.1 | ---------- ---------- | 156 |
| XP_024575884.1 | ---------- ---------- | 155 |
| XP_024586872.1 | ---------- ---------- | 182 |
| XP_024573052.1 | ---------- ---------- | 187 |
| XP_024586054.1 | ---------- ---------- | 237 |
| PvCRN27 | ---------- ---------- | 313 |
| PvCRN20 | ---------- ---------- | 159 |
| PvCRN25 | ---------- ---------- | 194 |
| PvCRN1 | ---------- ---------- | 580 |
| PvCRN4 | ---------- ---------- | 583 |
| PvCRN30 | ---------- ---------- | 220 |
| PvCRN29 | ---------- ---------- | 312 |
| XP_024583036.1 | ---------- ---------- | 681 |
| PvCRN10 | ---------- ---------- | 217 |
| XP_024577280.1 | ---------- ---------- | 283 |
| XP_024572924.1 | ---------- ---------- | 412 |
| PvCRN14 | ---------- ---------- | 151 |
| XP_024577521.1 | ---------- ---------- | 108 |
| PvCRN21 | ---------- ---------- | 479 |
| XP_024574966.1 | ---------- ---------- | 155 |
| XP_024575372.1 | ---------- ---------- | 188 |
| XP_024581363.1 | ---------- ---------- | 229 |
| XP_024583883.1 | ---------- ---------- | 381 |
| XP_024579844.1 | ---------- ---------- | 476 |
| XP_024574916.1 | ---------- ---------- | 621 |
| XP_024574193.1 | ---------- ---------- | 615 |
| XP_024583155.1 | ---------- ---------- | 419 |
| XP_024574913.1 | ---------- ---------- | 623 |
| XP_024578158.1 | ---------- ---------- | 106 |
| PvCRN22 | ---------- ---------- | 206 |
| XP_024585001.1 | ---------- ---------- | 127 |
| XP_024583843.1 | ---------- ---------- | 534 |
| XP_024578702.1 | ---------- ---------- | 299 |
| XP_024585805.1 | ---------- ---------- | 334 |
| XP_024580932.1 | ---------- ---------- | 508 |
| Consensus | ---------- ---------- | |
| Conservation (100% / 0%) | | |
