# Supplementary figures and images for: Characterization of CRN-Like Genes From Plasmopara viticola: Searching for the Most Virulent Ones
Source: Front Microbiol. 2021 Mar 22;12:632047. doi: 10.3389/fmicb.2021.632047 (PMC8044898; doi:10.3389/fmicb.2021.632047)

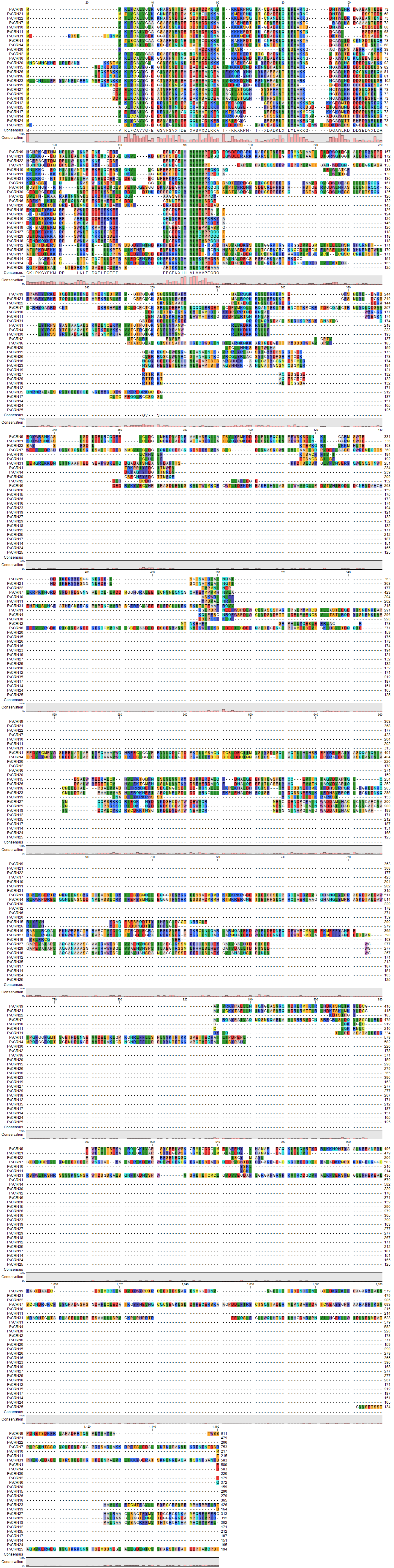

Supplement: Supplementary file 6 [file Image_1.PNG]

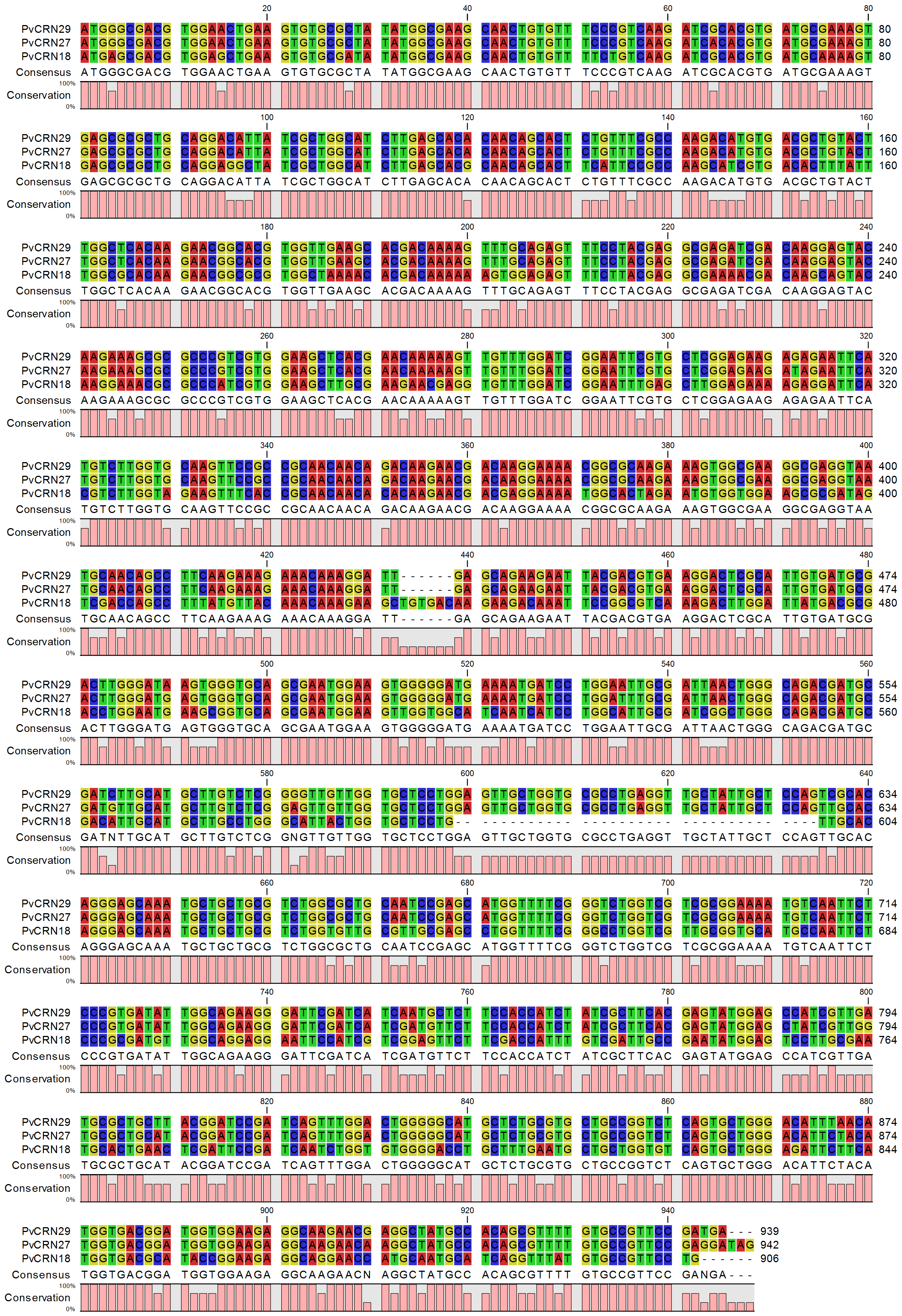

Supplement: Supplementary file 7 [file Image_2.PNG]

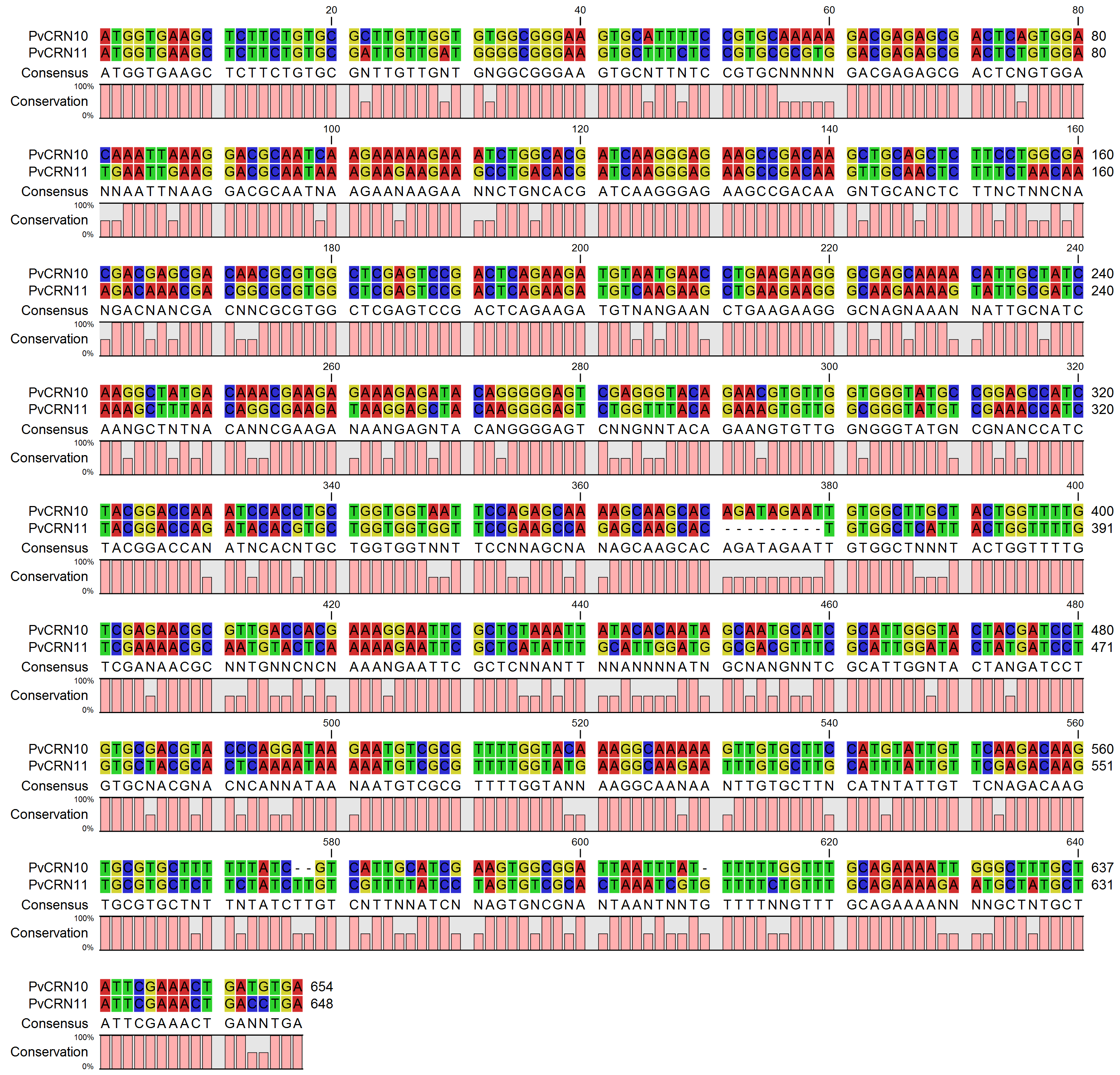

Supplement: Supplementary file 8 [file Image_3.PNG]

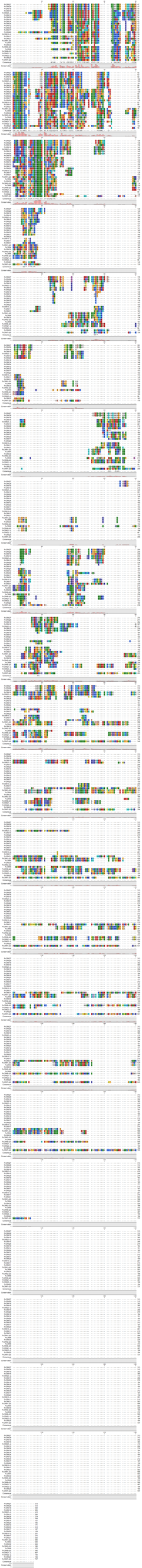

Supplement: Supplementary file 9 [file Image_4.PNG]

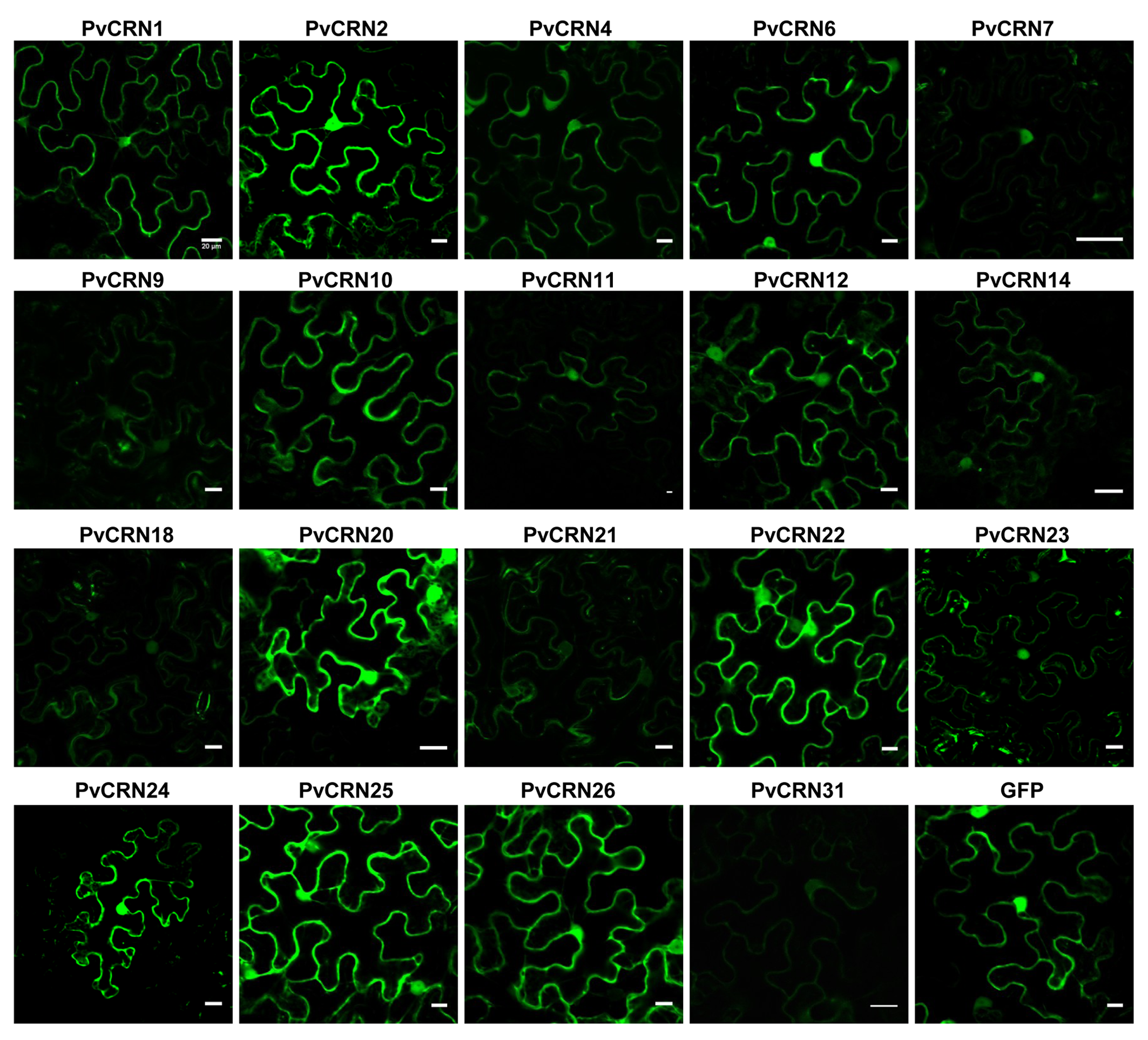

Supplement: Supplementary file 10 [file Image_5.TIF]

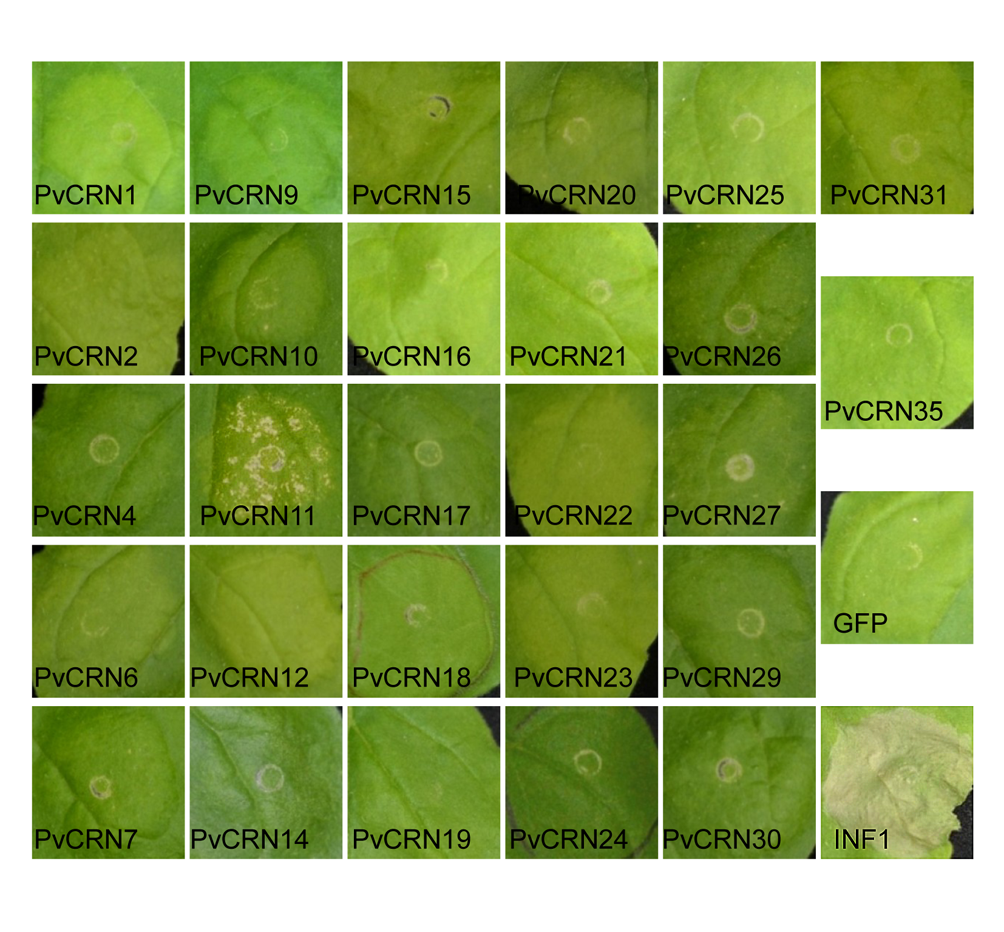

Supplement: Supplementary file 11 [file Image_6.TIF]

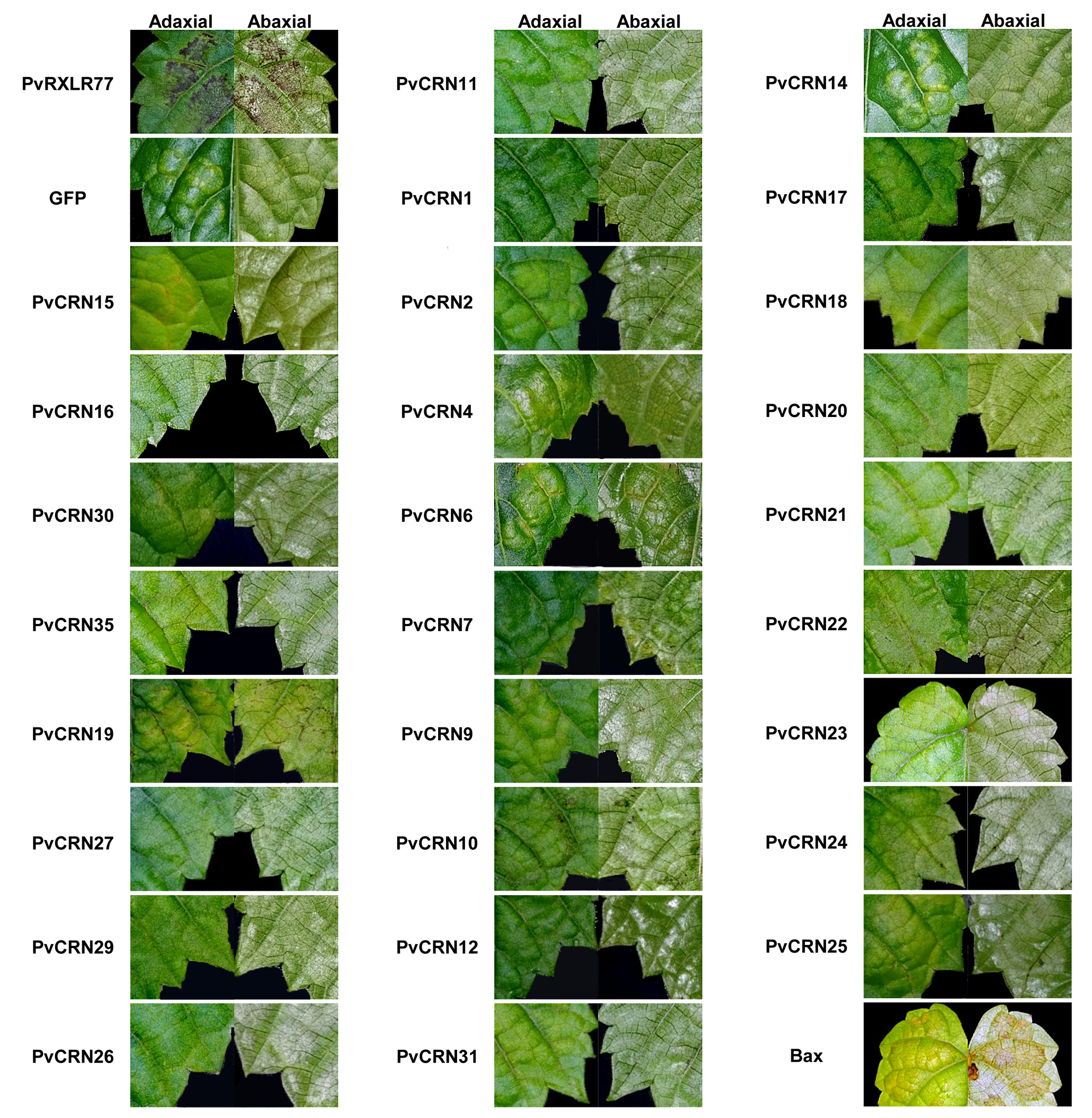

Supplement: Supplementary file 12 [file Image_7.TIF]

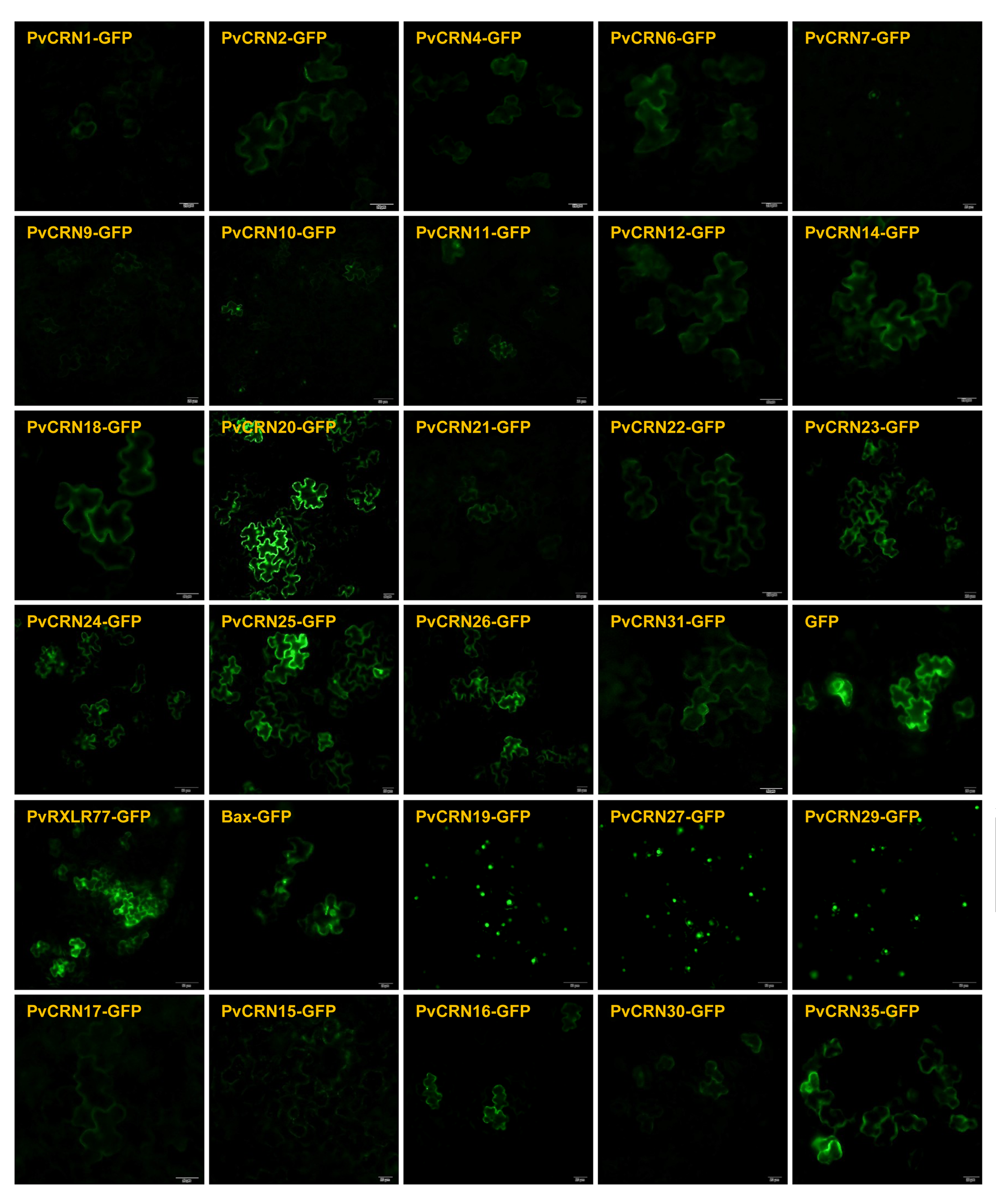

Supplement: Supplementary file 13 [file Image_8.TIF]

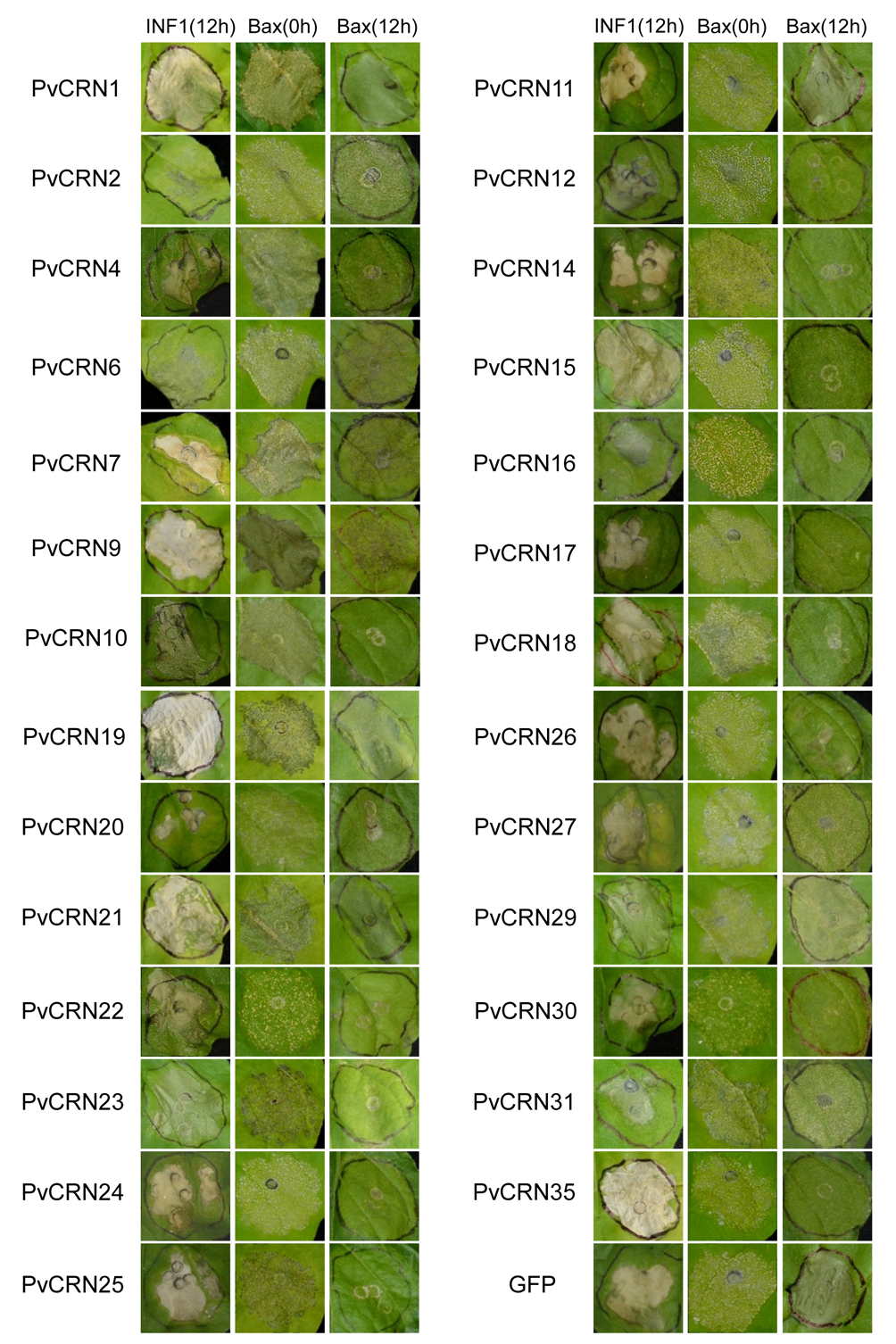

Supplement: Supplementary file 14 [file Image_9.TIF]

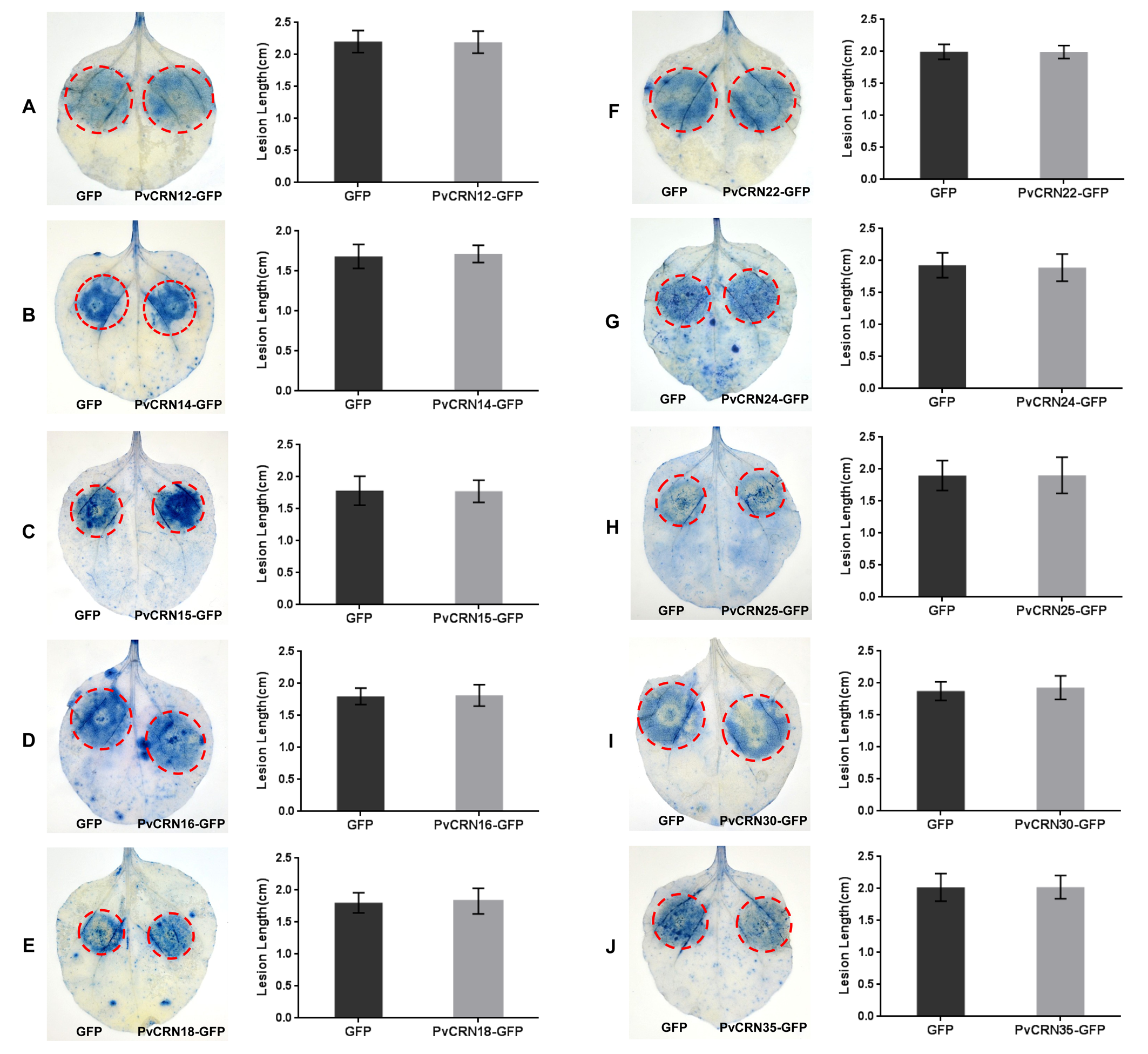

Supplement: Supplementary file 15 [file Image_10.TIF]

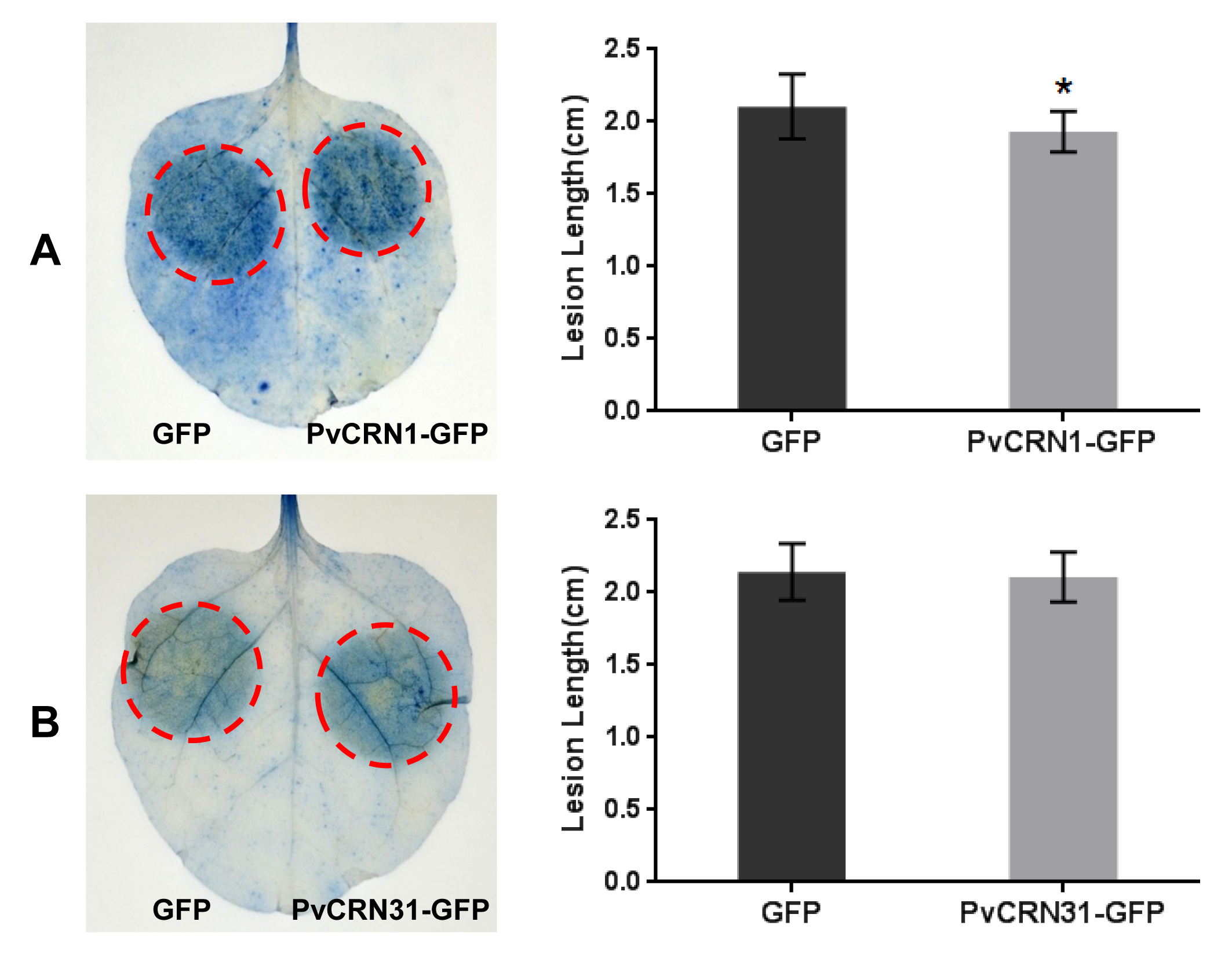

Supplement: Supplementary file 16 [file Image_11.TIF]
